# Identification of potential drug targets for diabetic polyneuropathy through Mendelian randomization analysis

**DOI:** 10.1186/s13578-024-01323-4

**Published:** 2024-12-05

**Authors:** Xiaokun Chen, Guohua Jiang, Tianjing Zhao, Nian Sun, Shanshan Liu, Hao Guo, Canjun Zeng, Yijun Liu

**Affiliations:** 1 Department of Orthopaedic Surgery, Shanghai Ninth People’s Hospital, Shanghai, China; 2 Department of Foot and Ankle Surgery, Center for Orthopedic Surgery, The Third Affiliated Hospital of Southern Medical University, Guangzhou, China; 3 Orthopedic Hospital of Guangdong Province, Guangzhou, China; 4 Zhujiang Hospital of Southern Medical University, 253 Gongye Middle Avenue, Guangzhou, 510280 China

**Keywords:** Diabetic polyneuropathy, Complications-nerves, Proteomics, Mendelian randomization, Drug development

## Abstract

**Background:**

Diabetic polyneuropathy (DPN) is a common diabetes complication with limited treatment options. We aimed to identify circulating plasma proteins as potential therapeutic targets for DPN using Mendelian Randomization (MR).

**Methods:**

The protein quantitative trait loci (pQTLs) utilized in this study were derived from seven previously published genome-wide association studies (GWASs) on plasma proteomics. The DPN data were obtained from the IEU OpenGWAS project. This study employed two-sample MR using MR-Egger and inverse-variance weighted methods to evaluate the causal relationship between plasma proteins and DPN risk, with Cochran’s Q test, and I^2^ statistics, among other methods, used to validate the robustness of the results.

**Results:**

Using cis-pQTLs as genetic instruments, we identified 62 proteins associated with DPN, with 33 increasing the risk and 29 decreasing the risk of DPN. Using cis-pQTLs + trans-pQTLs, we identified 116 proteins associated with DPN, with 44 increasing the risk and 72 decreasing the risk of DPN. Steiger directionality tests indicated that the causal relationships between circulating plasma proteins and DPN were consistent with expected directions.

**Conclusion:**

This study identified 96 circulating plasma proteins with genetically determined levels that affect the risk of DPN, providing new potential targets for DPN drug development, particularly ITM2B, CREG1, CD14, and PLXNA4.

**Supplementary Information:**

The online version contains supplementary material available at 10.1186/s13578-024-01323-4.

## Background

Diabetic Neuropathy (DN) refers to a range of nerve disorders caused by diabetes, including focal, autonomic, and peripheral neuropathies, affecting up to 50% of diabetic patients during their lifetime [1]. Diabetic Polyneuropathy (DPN), the most common form of DN, specifically involves multiple peripheral nerves, leading to sensory and motor dysfunction, and is generally considered the most disabling form of DN, often leading to lower limb ulcers and potentially resulting in amputation [2–4]. The prevalence of DPN varies widely, ranging from 26 to 50%, with painful DPN affecting 8–30% of patients. Currently, the main treatments for DPN include strict blood glucose control and pain management [2, 5, 6]. However, these methods are limited in effectiveness and cannot prevent or reverse the progressive axonal damage in patients with neuropathy. Therefore, identifying effective therapeutic targets to overcome the treatment challenges of DPN is crucial.

Proteins play a vital role in a range of biological processes and represent the largest class of drug targets [7]. Previous proteomics studies have identified certain proteins as biomarkers for DPN [8]. Recent research indicates that various plasma proteins (such as HLA-DRA) are associated with the risk of developing type 2 diabetes complications [9], and plasma proteins (such as plasma fibrinogen) are related to neurophysiological changes in type 2 diabetic peripheral neuropathy [10]. However, few studies have reported on the relationship between circulating plasma proteins and the risk of DPN. Moreover, current research mainly focuses on the association between single or a few circulating plasma proteins and DPN, often based on observational studies, which have limitations in inferring causal relationships [11]. Hence, more effective analytical methods are needed to explore the causal relationship between DPN and circulating plasma proteins.

Mendelian Randomization (MR) provides an alternative approach for exploring disease risk. By leveraging genetic variations within collected data, MR reveals causal relationships between risk factors and common diseases, thus minimizing the impact of confounding factors and reducing the potential for reverse causation [12, 13]. In recent years, genome-wide association studies (GWAS) have significantly contributed to understanding the etiology and identifying drug targets for diseases such as diabetes, cardiovascular diseases, and cancer [9, 14, 15]. Song et al. [16] identified causal associations between eight plasma proteins and peripheral neuropathy through MR, with DN being among these peripheral neuropathies. Notably, since DN includes all types of nerve damage caused by diabetes, Song et al. [16] analyzed the relationship between plasma proteins and DN from a broad perspective. Based on these results, DPN can be selected as the outcome variable in future studies to enhance the specificity of the findings, providing a comprehensive perspective for research on plasma proteins and DPN. To our knowledge, there is still a lack of MR studies on plasma proteins and DPN.

In this study, we aimed to identify circulating plasma proteins as potential therapeutic targets for DPN. Based on pQTLs from seven published GWAS used MR to identify potential pathogenic plasma proteins for DPN and further validated the causal direction through Steiger filtering. This study aims to explore the causal relationship between different circulating plasma proteins and DPN from a genetic perspective. This study helps clarify potential therapeutic targets for DPN and provides evidence to support the optimization of DPN prevention and treatment strategies.

## Methods

### Study design

**Fig. 1:**
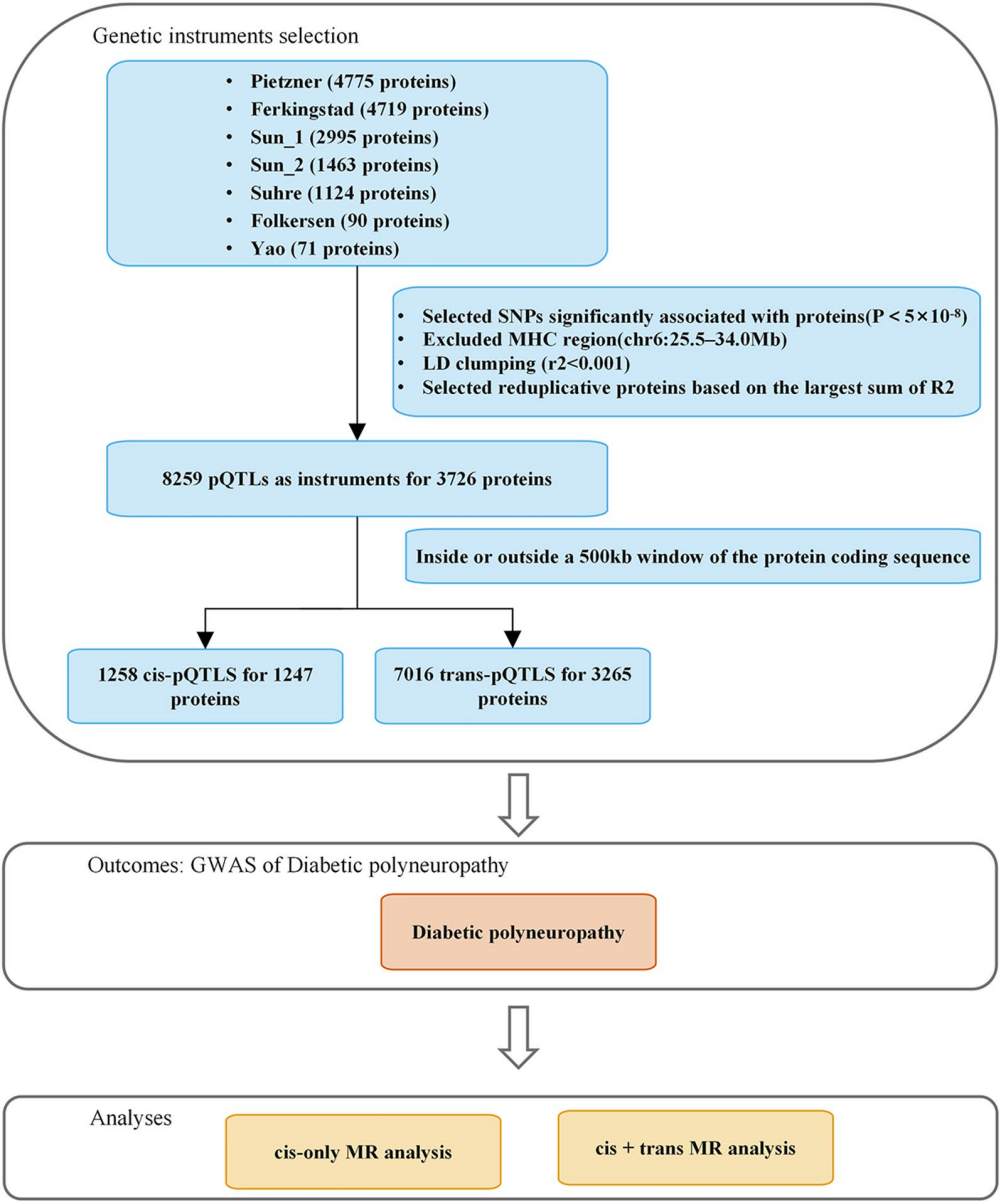
The framework flowchart of MR analysis evaluating the impact of plasma proteins on diabetic polyneuropathy

This study design is illustrated in Fig. 1. We used specific criteria to select eligible SNPs from the IEU OpenGWAS project dataset as instrumental variables for the pQTL-DPN causal relationship. As shown in the figure, our MR study design satisfied three necessary assumptions: (1) there is a strong association between the instrument and the exposure (relevance); (2) the genetic variation is independent of confounders of the exposure-outcome association (independence); (3) the instrumental variable affects the outcome only through the exposure (exclusivity), and it also adhered to the STROBE-MR guidelines[17].

### Data source and SNP identification

Genetic associations with DPN were derived from the IEU OpenGWAS project, including 358 cases and 217,377 controls (GWAS ID: finn-b-DM_POLYNEURO), all of European ancestry. The data were accessed on March 15, 2024. Case inclusion and exclusion were based on ICD10 chapter diagnostic criteria [18]. All included GWAS had received ethical approval and informed consent from the original databases.

Protein quantitative trait loci (pQTLs) were derived from seven published plasma proteome-wide GWASs [7, 19–24] (Table 1), including only those pQTLs meeting the following criteria: (1) SNPs showed high association (*P* < 5 × 10^−8^). (2) SNPs located in the human major histocompatibility complex (MHC) region (chr6: 25.5 to 34.0 Mb) were excluded. (3) SNPs demonstrated independence (linkage disequilibrium coefficient r^2^ = 0.001, linkage disequilibrium region width = 5000 kb). (4) For duplicate proteins, we selected those with the highest R^2^ total (F > 10).

**Table 1:** Summary information of pQTLs and GWAS databases used in the MR study

Data source	Phenotype	Sample size	Population	Adjustment
Exposure: circulating plasma proteins	Pietzner et al.	10,708	European	–
	Ferkingstad et al.	35,559	European	–
	Sun_1 et al.	3301	European	–
	Sun_2 et al.	35,571	European	–
	Suhre et al.	1000	European	–
	Folkersen et al.	30,000	European	–
	Yao et al.	6861	European	–
Outcome: diabetic polyneuropathy	IEU Open GWAS project	217,735	European	Males and females

This study selection criteria for serum pQTLs instrumental variables were as follows: (1) The instrumental variable had a strong association with the exposure, with *P* < 5 × 10^−8^ as the strong correlation standard (relevance assumption); (2) SNPs located in the MHC region (chr6: 25.5 to 34.0 Mb) were excluded; (3) Setting the linkage disequilibrium coefficient r^2^ = 0.001 and linkage disequilibrium region width = 5000 kb to ensure independence of each SNP, removing the influence of linkage disequilibrium on results. (4) Using R^2^ (R^2^ = 2 × EAF × (1 − EAF) × β^2^) and F-statistic (F = R^2^ × (N − 2)/(1 − R^2^)) to estimate the strength of genetic instrumental variables. For duplicate proteins, we selected those with the highest R^2^ total, using F-statistic > 10 as the threshold for excluding weak instruments.

Relevant SNPs were extracted from DPN GWAS data, excluding palindromic SNPs and those with MAF > 0.42, and outlier SNPs were excluded using MR-PRESSO. Instrumental variables were classified into cis-pQTLs and trans-pQTLs. Cis-pQTLs were defined as pQTLs within a 500 kb window of the corresponding protein-coding sequence, while trans-pQTLs were defined as pQTLs outside the 500 kb window of the protein-coding gene.

### Mendelian randomization analysis

We used five regression models—MR-Egger regression, inverse-variance weighted (IVW) random effects, weighted median estimator (WME), weighted mode, and simple mode—to evaluate the potential causal relationship between exposure factors (circulating plasma proteins) and outcome (DPN) risks using pQTLs as instrumental variables. IVW was the primary method for causal estimation, with MR-Egger regression as a supplementary method. When SNPs ≤ 3, the Wald ratio method was used; otherwise, the IVW random effects method was employed. MR-Egger uses weaker assumptions (InSIDE) on top of IVW to estimate causal effects, introducing a regression intercept to detect and correct bias caused by instrument pleiotropy.

SNP heterogeneity was assessed using Cochran’s Q test and I^2^ statistics. If Cochran’s Q test *P* < 0.05 or I^2^ > 50%, results were considered heterogeneous. Pleiotropy was analyzed using the MR-Egger intercept and MR-PRESSO. An MR-Egger intercept ≠ 0 (*P* > 0.05) and MR-PRESSO *P* > 0.05 indicated no pleiotropy. Sensitivity analysis was performed using leave-one-out analysis to observe the influence of each SNP on the results. All analyses were conducted using the TwoSampleMR package in R 4.1.0, with a significance level of α = 0.05.

### Steiger filtering analysis

The Steiger filtering test is a statistical method to verify the consistency of the causal direction between genotype, intermediate variable, and final outcome. We calculated the explained variance of SNPs for circulating plasma proteins and DPN, then tested whether the outcome variance was less than the target gene variance. In MR Steiger results, if the outcome variance was less than the circulating plasma protein variance, it was judged as "TRUE," indicating the causal relationship direction was consistent with expectations, while "FALSE" indicated the opposite.

## Results

### Identification of pQTLs

From seven proteomic GWAS datasets, we screened pQTLs as genetic instrumental variables, retaining 8259 pQTLs for 3726 unique proteins for MR analysis. We further classified these instrumental variables into cis-pQTLs and trans-pQTLs, with 1258 cis-pQTLs for 1247 proteins and 7016 trans-pQTLs for 3265 proteins. Among the effective instrumental variables, 37 proteins were influenced by both cis-pQTLs and cis-pQTLs + trans-pQTLs, 25 by cis-pQTLs only, and 79 by cis-pQTLs + trans-pQTLs only. Detailed SNP information associated with DPN can be found in Supplementary Tables 1 and 2.

### Circulating plasma proteins are associated with the risk of DPN

**Fig. 2:**
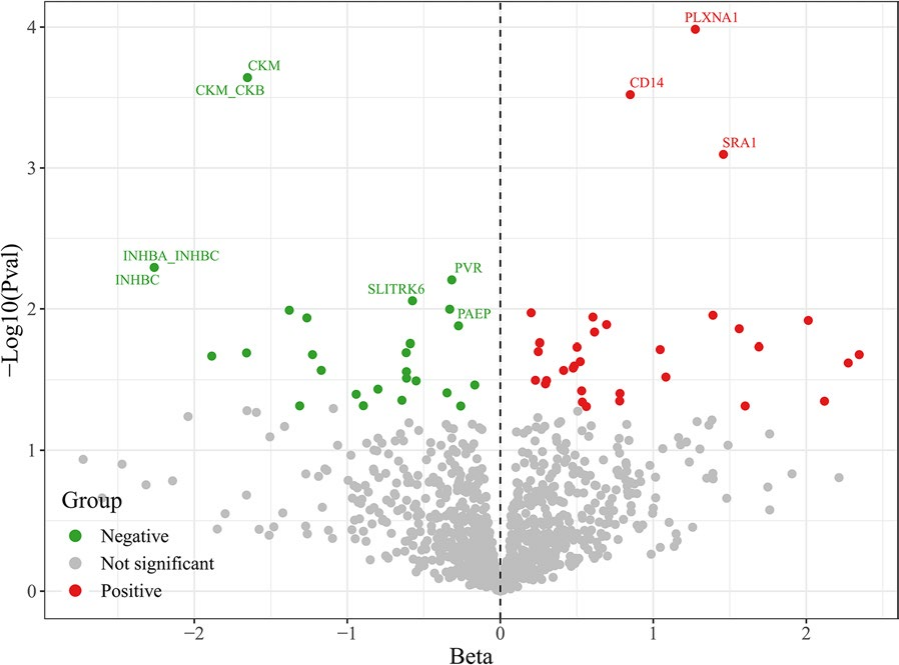
Volcano plot of the causal effects of circulating plasma proteome on diabetic polyneuropathy using *cis*-pQTLs analysis

First, using cis-pQTLs as genetic instruments for MR analysis, we systematically evaluated the causal effects of circulating plasma proteins on DPN (Fig. 2). MR analysis identified 62 proteins associated with DPN, with 33 increasing the risk and 29 decreasing the risk of DPN. For example, IVW results showed that circulating plasma protein CD14 was associated with an increased risk of DPN (OR = 2.338, 95% CI 1.475–3.707, *P* < 0.001), with no heterogeneity between eQTLs associated with CD14 and DPN (I^2^ = 0%, Cochran’s Q = 0.205, *P* = 0.650) (Supplementary Table 3). Conversely, circulating plasma protein CKM was a protective factor for DPN (OR = 0.192, 95% CI 0.080–0.461, *P* < 0.001). Additionally, circulating plasma protein INHBC significantly reduced the risk of DPN (OR = 0.104, 95% CI 0.021–0.507, *P* = 0.005). Due to the small number of SNPs, MR-Egger and MR-PRESSO results for the 62 proteins associated with DPN were reported as NA.

**Fig. 3:**
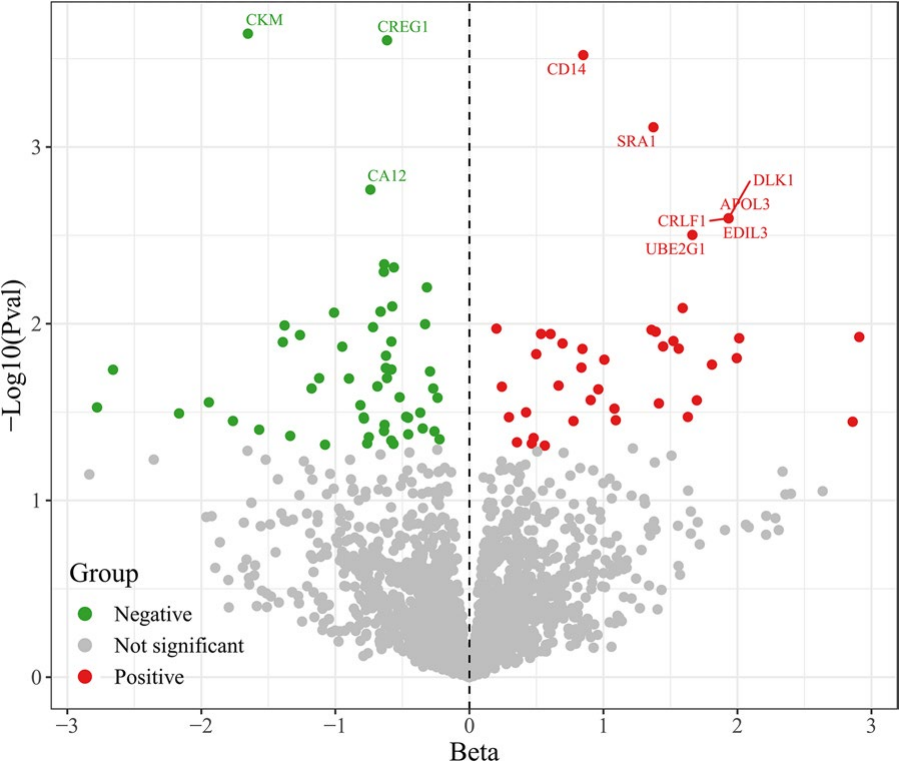
Volcano plot of the causal effects of circulating plasma proteome on diabetic polyneuropathy using *cis*-pQTLs + trans-pQTLs analysis

In MR analysis, adding trans-pQTLs may increase the reliability of protein-phenotype associations. Therefore, we used all (*cis* + *trans*) pQTLs as instrumental variables to extend our MR analysis. In the cis + trans pQTLs MR analysis, IVW showed statistically significant causal relationships between 116 plasma proteins and DPN (Fig. 3). Among them, 72 decreased the risk and 44 increased the risk of DPN. Plasma protein CREG1 was a protective factor for DPN (OR = 0.541, 95% CI 0.389–0.751, *P* < 0.001), with no heterogeneity between SNPs associated with CREG1 and DPN (I^2^ = 0%, Cochran’s Q = 0.603, *P* = 0.978), and MR-Egger showed no significant difference in the intercept (*P* = 0.963). MR-PRESSO did not detect significant horizontal pleiotropy (*P* = 0.960), indicating robust MR results. Plasma protein CD14 was a risk factor for DPN (OR = 2.338, 95% CI 1.475–3.707, *P* < 0.001), with no heterogeneity between SNPs associated with CD14 and DPN (I^2^ = 0%, Cochran’s Q = 0.205, *P* = 0.650), and MR-Egger showed no significant difference in the intercept (*P* = 0.506). MR-PRESSO did not detect significant horizontal pleiotropy (*P* = 0.107), indicating robust MR results (Supplementary Table 4). For plasma proteins with SNPs > 3, MR-Egger showed no significant difference in intercepts (*P* > 0.05), and MR-PRESSO did not detect significant horizontal pleiotropy (*P* > 0.05), suggesting robust MR results (Supplementary Table 4). The forest plots, leave-one-out plots, scatter plots, and funnel plots for representative circulating plasma proteins ITM2B, CREG1, CD14, and PLXNA4 are shown in Figures S1-4. Funnel plots showed symmetrical distribution of points representing causal association effects when a single SNP was used as IV, suggesting minimal potential bias influence (Figures S1–4). Scatter plots showed significant associations between SNP effects and diabetic polyneuropathy across different MR analysis methods (e.g., inverse-variance weighted, MR-Egger, simple mode, weighted median, and weighted mode) (Figures S1–4). Leave-one-out results showed minimal changes in overall error lines after sequential exclusion of each SNP, indicating reliable MR results (Figures S1–4).

### Filter analysis

Using cis-pQTLs and *cis* + *trans* pQTLs as genetic instruments for MR analysis, Steiger directionality test results are shown in Supplementary Tables 5 and 6. In the DPN dataset, the direction of circulating plasma proteins was “TRUE,” indicating that the causal relationship between circulating plasma proteins and outcomes was consistent with expectations. For example, the explained variance of the instrumental variable SNPs for circulating plasma protein CD14 was greater than its variance for DPN (Steiger_pval = 6.3233E-113), and the MR Steiger result was judged as “TRUE,” indicating that the causal relationship between circulating plasma protein CD14 and DPN was consistent with the expected direction.

## Discussion

Despite significant progress in exploring the etiology of DPN in recent years, the specific mechanisms and molecular targets remain unclear. In this study, we comprehensively investigated the causal relationship between 8259 pQTLs and DPN using MR. Using cis-pQTLs as genetic instruments, we identified 62 proteins with genetically determined levels associated with DPN: 33 proteins increase the risk, while 29 proteins decrease the risk of DPN. Using both cis-pQTLs and trans-pQTLs as genetic instruments, we identified 116 proteins with genetically determined levels associated with DPN: 72 increasing the risk and 44 decreasing the risk of DPN. Among the 96 circulating plasma proteins associated with the risk of DPN, we consider ITM2B, CREG1, CD14, and PLXNA4 to be valuable potential targets for drug development. This is because the IVW method results for these four proteins have smaller p-values among all positive results and are mechanistically closely related to DPN.

DPN is a common complication of diabetes, with a complex pathology involving factors such as hyperglycemia, oxidative stress, inflammation, and microvascular disease [25, 26]. ITM2B (Integral Membrane Protein 2B) is a widely expressed transmembrane protein, playing significant roles in the nervous system, particularly in neurodegenerative diseases. In Alzheimer's disease, ITM2B, through its BRICHOS domain, participates in protein folding and prevents amyloid fibril formation [27]. In diabetic mouse models, ITM2B expression is also upregulated, suggesting its role in diabetes and neuropathy [28]. We found that circulating ITM2B protein is a protective factor for DPN, indicating its potential as a valuable therapeutic target for DPN.

CREG1 protein (cellular repressor of E1A-stimulated genes 1) is an endolysosomal protein that promotes endocytic transport and lysosome biogenesis. Overexpression of CREG1 increases phosphorylation of InsR and its downstream effectors Akt and GSK-3beta, improving insulin resistance. Knockdown of CREG1 reduces InsR expression, indicating its crucial role in insulin signaling by enhancing insulin receptor recycling [29]. Additionally, CREG1 is an important cardioprotective factor, inhibiting FBXO27 expression to prevent LAMP2 protein degradation, promoting autophagy in cardiomyocytes, and alleviating diabetic cardiomyopathy [30]. Our findings show that circulating CREG1 protein is also a protective factor for DPN, underscoring its potential as a therapeutic target.

Immune system dysregulation and chronic low-grade inflammation are common features of diabetes and peripheral neuropathy [31, 32]. CD14 is a component of the lipopolysaccharide (LPS) receptor, recognizing and binding LPS to initiate inflammatory responses. Elevated CD14 expression levels have been observed in diabetic patients, potentially promoting inflammation and leading to nerve damage [33, 34]. Elzinga et al. found that TLR (Toll-like receptor) signaling, particularly TLR 2 and 4, is involved in the onset and progression of diabetic peripheral neuropathy [35]. CD14 is a co-receptor for TLR4, both participating in inflammatory signal transduction and playing key roles in innate immune responses [36]. Activation of the TLR4/CD14 signaling pathway in DPN may be a significant cause of neuronal injury and death. In our study, elevated CD14 protein levels were a risk factor for DPN, suggesting that CD14 inhibitors could be potential treatments for DPN.

PLXNA4 (Plexin A4) is a transmembrane receptor protein belonging to the Plexin family and is a critical receptor for class 3, 6A, and 6B secreted semaphorins, playing significant roles in the nervous system. In PLXNA4 knockout mice, defects are observed in peripheral sensory and sympathetic nerve fibers [37]. Moreover, PLXNA4 can influence cell morphology and movement by regulating Rac1 GTPase activation, affecting cytoskeletal organization, thus influencing peripheral nerve axon growth [38]. Our study suggests that PLXNA4 is a risk factor for DPN, and it has the potential to be a therapeutic target for DPN. In this study, there are several other targets that merit attention. For example, proteins that may influence DPN by modulating inflammatory responses include INHBC, TIGIT, LEFTY2, CA12, SRA1, ENPP7, HADH, and ULBP2. Additionally, proteins that may affect DPN by protecting neural cells and promoting nerve regeneration include INHBA_INHBC, PVR, SLITRK6, NECTIN4, and PPP1R12A.

Recent studies have also explored new drug targets for DPN, identifying factors such as Methylglyoxal, Glyoxalase I, and Nrf2 as promising candidates for therapeutic intervention. These factors are associated with the production of advanced glycation end products, inflammation, and apoptosis. However, these studies primarily employ methods such as PCR, Western Blot detection, or transcriptome sequencing on tissue samples from T2DM patients, which present challenges such as small sample sizes, inconsistent results, and difficulties in distinguishing correlation from causation [39, 40]. In contrast, our study utilizes Mendelian randomization analysis, which offers advantages such as large sample sizes, reduced confounding factors, and the ability to establish clear causal relationships. This approach allows us to identify potential drug targets that have a causal relationship with DPN.

Song et al. [16] through a two-sample Mendelian randomization analysis, identified plasma protein UBC12 as a potential protective factor for DN. Similar to their study, our research also employed Mendelian randomization, focused on neuropathy-related proteins, and involved exploration of drug targets. However, we included more comprehensive circulating plasma protein data from seven GWAS studies and adopted stricter instrumental variable selection criteria (linkage disequilibrium region width of 5000 Kb) to optimize the selection of instrumental variables, thus enhancing the reliability and statistical power of the analysis while comprehensively considering the genetic basis of complex traits. Furthermore, we investigated the causal associations of cis-pQTLs and trans-pQTLs of circulating plasma proteins with DPN, providing a more comprehensive perspective to the field. Steiger filtering analysis further enhanced the credibility of causal inference in our study. Notably, primarily due to differences in instrumental variable selection criteria and datasets, we did not replicate a positive finding for UBC12 in this study. In future research, we plan to validate our findings, as well as those of Song et al. [16] through basic experiments and clinical trials.

The strengths of our study are as follows: First, there is no overlap between exposure factors—SNPs and outcome factors—SNPs, ensuring that the instrumental variables for exposure and outcome factors are not in linkage disequilibrium. Second, by using GWAS results for causal inference, we reduce the possibility of reverse causality and confounding bias. Third, we employ MR-Egger and MR-PRESSO to make different assumptions about the presence of direct pleiotropy and conduct sensitivity analyses. These complementary methods help evaluate the reliability of causal estimates. Our study also has some limitations. First, we examine the effects of proteins from different studies, and the heterogeneity between these studies may introduce bias. Second, this study cannot directly assess whether population stratification and other potential confounding factors might affect the results, leaving the possibility of unmeasured confounding factors. Third, we only analyzed European populations, so caution is needed when extending the findings to other populations.

## Conclusions

This study identified 96 circulating plasma proteins that significantly influence the risk of DPN. Among these, ITM2B, CREG1, CD14, and PLXNA4 stand out as promising potential drug targets, offering new possibilities for therapeutic intervention in DPN. These findings provide valuable insights that could guide future research and treatment strategies for this challenging complication of diabetes.

## Supplementary Information

Supplementary Material 1: Figure S1. ITM2B: (A) Forest plot, (B) Leave-one-out sensitivity analysis plot, (C) Scatter plot, and (D) Funnel plot.

Supplementary Material 2: Figure S2. CREG1: (A) Forest plot, (B) Leave-one-out sensitivity analysis plot, (C) Scatter plot, and (D) Funnel plot.

Supplementary Material 3: Figure S3. CD14: (A) Forest plot, (B) Scatter plot, and (C) Funnel plot.

Supplementary Material 4: Figure S4. PLXNA4: (A) Forest plot, (B) Leave-one-out sensitivity analysis plot, (C) Scatter plot, and (D) Funnel plot.

Supplementary Material 5.

Supplementary Material 6.

Supplementary Material 7.

## Data Availability

The datasets used and/or analysed during the current study are available from the corresponding author on reasonable request.

## Abbreviations

DPN: Diabetic polyneuropathy
MR: Mendelian Randomization
pQTLs: Protein quantitative trait loci
GWASs: Genome-wide association studies
DN: Diabetic neuropathy
IVW: Inverse-variance weighted
ITM2B: Integral Membrane Protein 2B
CREG1: Cellular repressor of E1A-stimulated genes 1
LPS: Lipopolysaccharide
TLR: Toll-like receptor
PLXNA4: Plexin A4
