# Supplementary material for: Identification of potential drug targets for diabetic polyneuropathy through Mendelian randomization analysis
Source: Cell Biosci. 2024 Dec 5;14:147. doi: 10.1186/s13578-024-01323-4 (PMC11619124; doi:10.1186/s13578-024-01323-4)
Supplement: Supplementary file 1 — Supplementary Material 1: Figure S1. ITM2B: (A) Forest plot, (B) Leave-one-out sensitivity analysis plot, (C) Scatter plot, and (D) Funnel plot. [file 13578_2024_1323_MOESM1_ESM.docx]

**Supplementary materials**


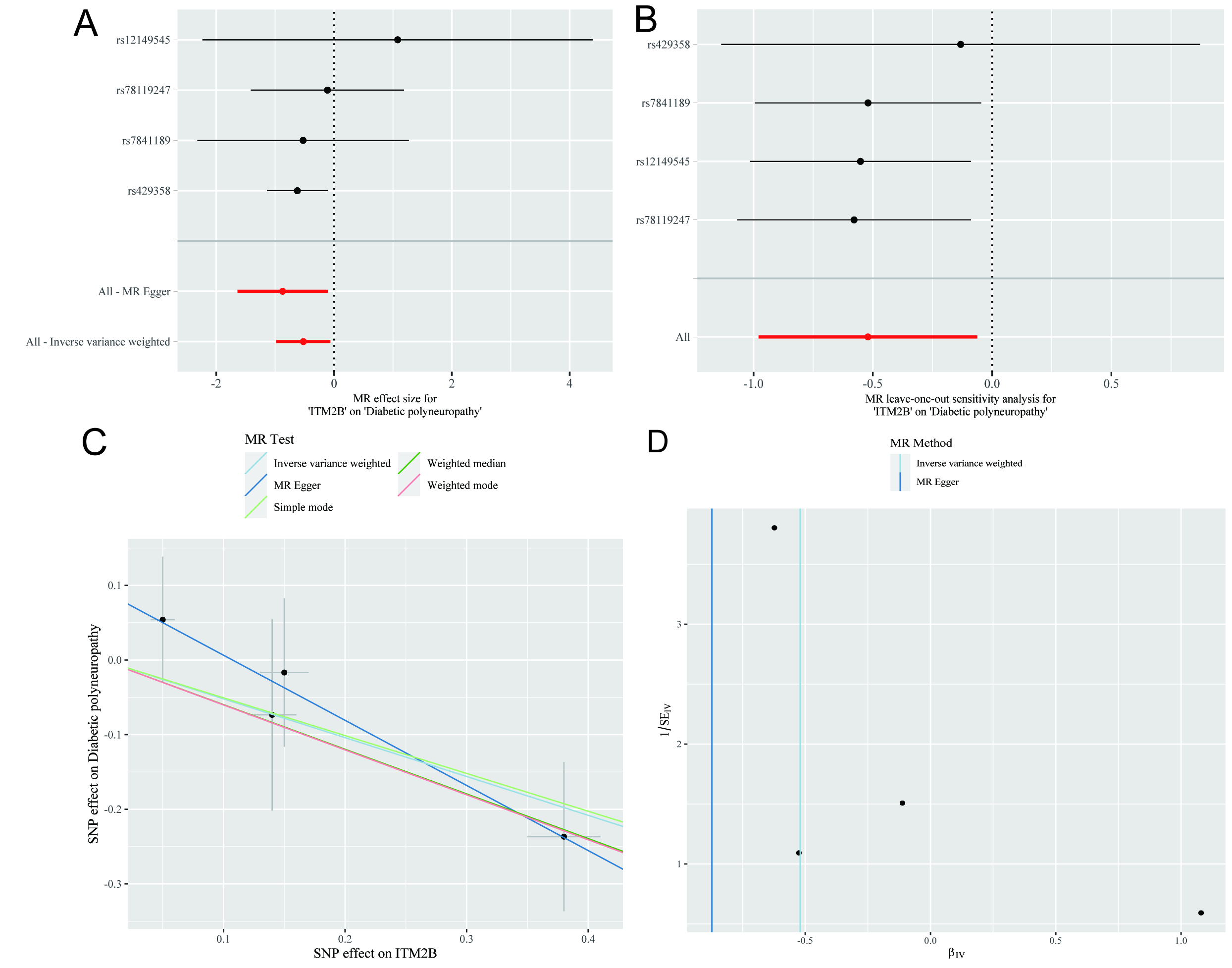


Figure S1. ITM2B: (A) Forest plot, (B) Leave-one-out sensitivity analysis plot, (C) Scatter plot, and (D) Funnel plot


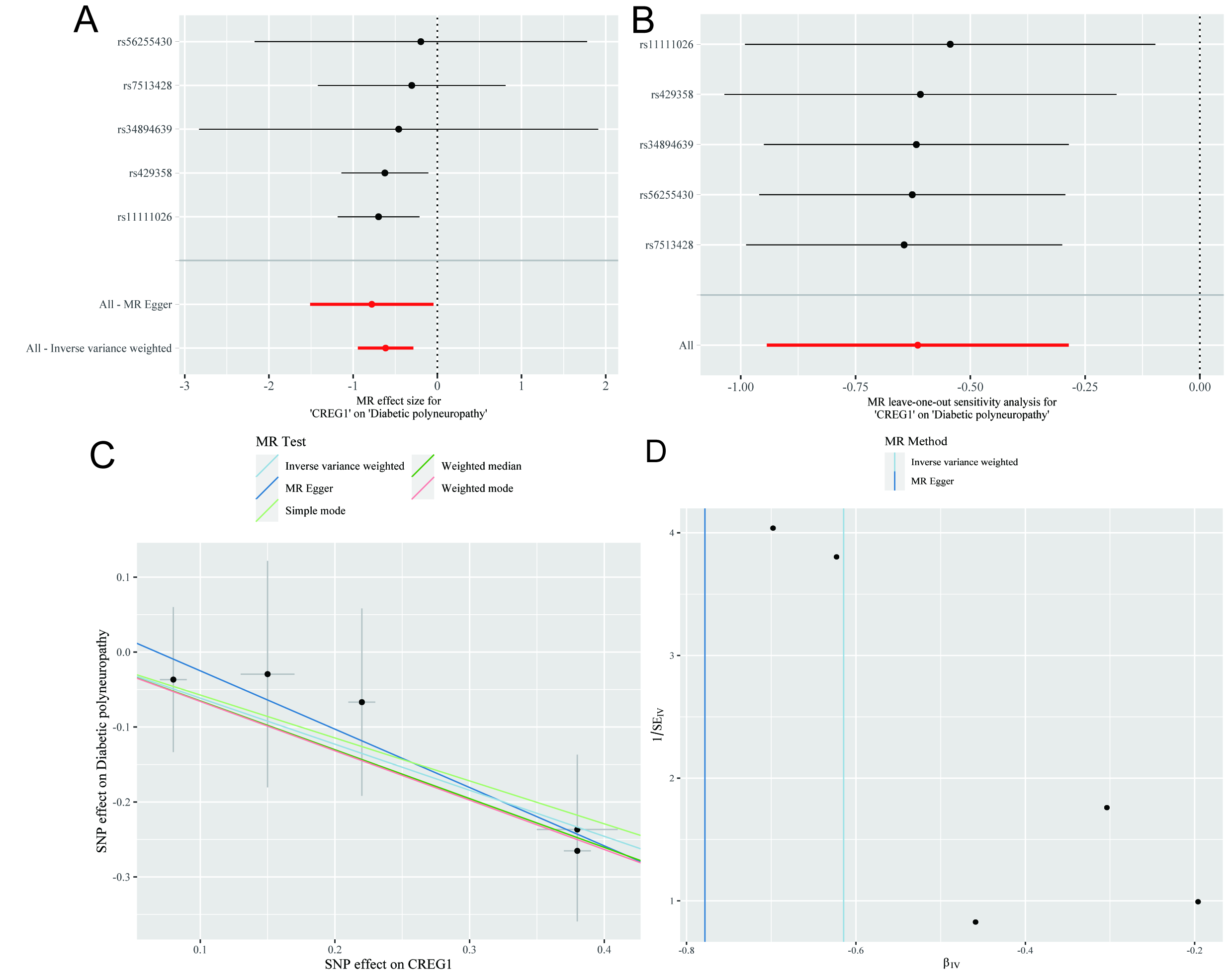


Figure S2. CREG1: (A) Forest plot, (B) Leave-one-out sensitivity analysis plot, (C) Scatter plot, and (D) Funnel plot.


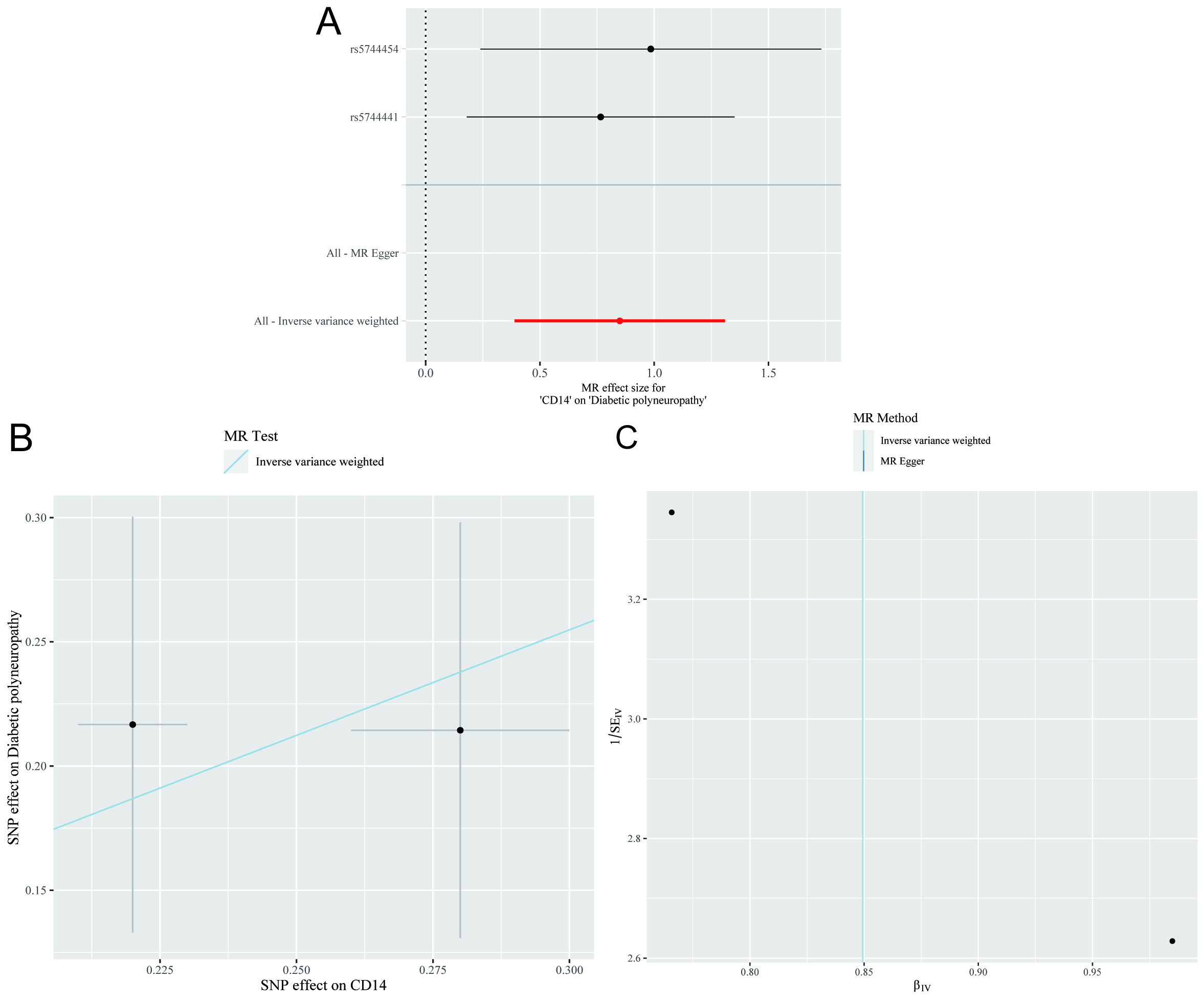


Figure S3. CD14: (A) Forest plot, (B) Scatter plot, and (C) Funnel plot.


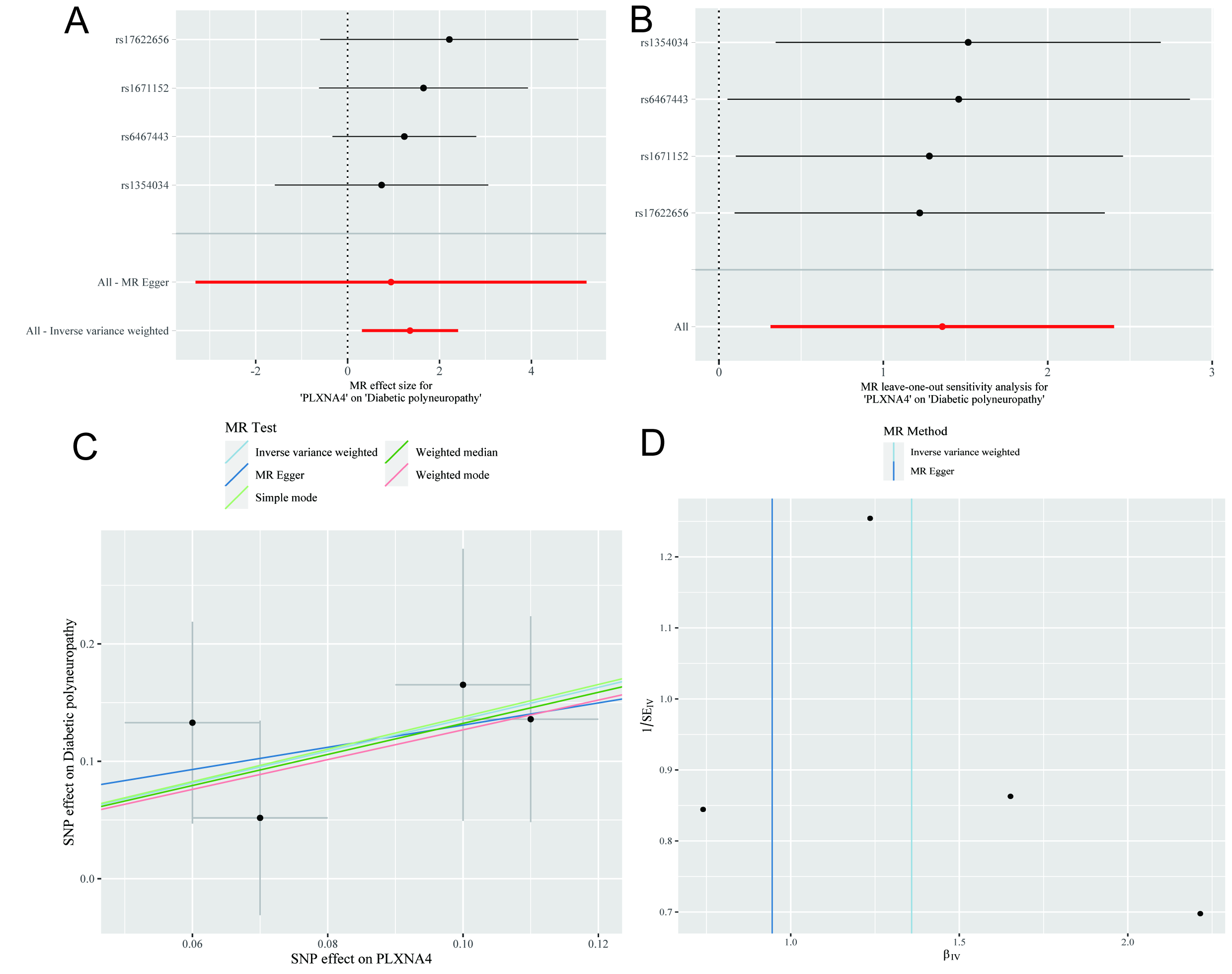


Figure S4. PLXNA4: (A) Forest plot, (B) Leave-one-out sensitivity analysis plot, (C) Scatter plot, and (D) Funnel plot.
