# Supplementary material for: Identification of potential drug targets for diabetic polyneuropathy through Mendelian randomization analysis
Source: Cell Biosci. 2024 Dec 5;14:147. doi: 10.1186/s13578-024-01323-4 (PMC11619124; doi:10.1186/s13578-024-01323-4)
Supplement: Supplementary file 2 — Supplementary Material 2: Figure S2. CREG1: (A) Forest plot, (B) Leave-one-out sensitivity analysis plot, (C) Scatter plot, and (D) Funnel plot. [file 13578_2024_1323_MOESM2_ESM.docx]

**Plasma Protein cis-pQTLs as Genetic Instruments for Mendelian Randomization Analysis**

| **SNP** | **Outcome** | **CHR** | **POS** | **EA/OA** | **EAF** | **β*** | **SE** | **P** | **MAF** | **Cis_trans** |
| --- | --- | --- | --- | --- | --- | --- | --- | --- | --- | --- |
| TNFSF8 |  |  |  |  |  |  |  |  |  |  |
| rs1006026 | Diabetic polyneuropathy | 9 | 117691270 | A/G | 0.583 | 0.017 | 0.077 | 0.826 | 0.417 | cis |
| ADAMTSL2 |  |  |  |  |  |  |  |  |  |  |
| rs1008250 | Diabetic polyneuropathy | 9 | 136397834 | A/G | 0.657 | -0.150 | 0.082 | 0.066 | 0.343 | cis |
| MGMT |  |  |  |  |  |  |  |  |  |  |
| rs1008982 | Diabetic polyneuropathy | 10 | 131445731 | C/T | 0.412 | -0.170 | 0.077 | 0.027 | 0.412 | cis |
| EFS |  |  |  |  |  |  |  |  |  |  |
| rs10137082 | Diabetic polyneuropathy | 14 | 23840033 | T/C | 0.156 | -0.153 | 0.105 | 0.146 | 0.156 | cis |
| PGLYRP2 |  |  |  |  |  |  |  |  |  |  |
| rs10164310 | Diabetic polyneuropathy | 19 | 15585945 | A/G | 0.186 | 0.012 | 0.097 | 0.905 | 0.186 | cis |
| MERTK |  |  |  |  |  |  |  |  |  |  |
| rs10188642 | Diabetic polyneuropathy | 2 | 112741099 | A/G | 0.668 | 0.058 | 0.080 | 0.468 | 0.333 | cis |
| SEMA3C |  |  |  |  |  |  |  |  |  |  |
| rs1019016 | Diabetic polyneuropathy | 7 | 80570562 | T/G | 0.583 | 0.098 | 0.077 | 0.201 | 0.417 | cis |
| RAB6B |  |  |  |  |  |  |  |  |  |  |
| rs10212397 | Diabetic polyneuropathy | 3 | 133607140 | A/G | 0.208 | -0.079 | 0.094 | 0.400 | 0.208 | cis |
| CXCL12 |  |  |  |  |  |  |  |  |  |  |
| rs1023264 | Diabetic polyneuropathy | 10 | 44893756 | T/C | 0.721 | 0.014 | 0.085 | 0.865 | 0.280 | cis |
| NUDCD3 |  |  |  |  |  |  |  |  |  |  |
| rs10233273 | Diabetic polyneuropathy | 7 | 44502186 | T/C | 0.203 | 0.187 | 0.093 | 0.045 | 0.203 | cis |
| CBR3 |  |  |  |  |  |  |  |  |  |  |
| rs1028997 | Diabetic polyneuropathy | 21 | 37532222 | A/G | 0.304 | -0.024 | 0.082 | 0.769 | 0.304 | cis |
| PZP |  |  |  |  |  |  |  |  |  |  |
| rs1035849 | Diabetic polyneuropathy | 12 | 9362168 | T/C | 0.326 | 0.117 | 0.081 | 0.146 | 0.326 | cis |
| SEMA6B |  |  |  |  |  |  |  |  |  |  |
| rs10404223 | Diabetic polyneuropathy | 19 | 4549646 | A/G | 0.372 | 0.026 | 0.080 | 0.742 | 0.372 | cis |
| CD22 |  |  |  |  |  |  |  |  |  |  |
| rs10411704 | Diabetic polyneuropathy | 19 | 35800662 | T/G | 0.749 | -0.025 | 0.087 | 0.776 | 0.251 | cis |
| IRF3 |  |  |  |  |  |  |  |  |  |  |
| rs10423580 | Diabetic polyneuropathy | 19 | 50195771 | T/G | 0.615 | -0.030 | 0.078 | 0.703 | 0.385 | cis |
| ANPEP |  |  |  |  |  |  |  |  |  |  |
| rs1042499 | Diabetic polyneuropathy | 15 | 90328305 | C/T | 0.030 | -0.137 | 0.219 | 0.531 | 0.030 | cis |
| THBD |  |  |  |  |  |  |  |  |  |  |
| rs1042579 | Diabetic polyneuropathy | 20 | 23028724 | A/G | 0.278 | 0.158 | 0.085 | 0.063 | 0.278 | cis |
| ARHGEF25 |  |  |  |  |  |  |  |  |  |  |
| rs10437954 | Diabetic polyneuropathy | 12 | 58003922 | A/G | 0.894 | 0.027 | 0.125 | 0.827 | 0.106 | cis |
| IDI2 |  |  |  |  |  |  |  |  |  |  |
| rs1044261 | Diabetic polyneuropathy | 10 | 1065710 | T/C | 0.050 | -0.338 | 0.171 | 0.049 | 0.050 | cis |
| AOC1 |  |  |  |  |  |  |  |  |  |  |
| rs10452848 | Diabetic polyneuropathy | 7 | 150523544 | A/G | 0.638 | -0.004 | 0.079 | 0.961 | 0.362 | cis |
| ECHS1 |  |  |  |  |  |  |  |  |  |  |
| rs10466126 | Diabetic polyneuropathy | 10 | 135186806 | A/G | 0.177 | -0.108 | 0.100 | 0.282 | 0.177 | cis |
| KL |  |  |  |  |  |  |  |  |  |  |
| rs10467359 | Diabetic polyneuropathy | 13 | 33529264 | G/A | 0.073 | 0.301 | 0.147 | 0.040 | 0.073 | cis |
| GUCA2B |  |  |  |  |  |  |  |  |  |  |
| rs1047047 | Diabetic polyneuropathy | 1 | 42619139 | A/G | 0.832 | 0.005 | 0.101 | 0.964 | 0.168 | cis |
| ENPP5 |  |  |  |  |  |  |  |  |  |  |
| rs1047153 | Diabetic polyneuropathy | 6 | 46128745 | T/C | 0.717 | -0.064 | 0.084 | 0.444 | 0.283 | cis |
| KLK11 |  |  |  |  |  |  |  |  |  |  |
| rs1048328 | Diabetic polyneuropathy | 19 | 51527364 | A/G | 0.100 | 0.133 | 0.128 | 0.299 | 0.100 | cis |
| CRNN |  |  |  |  |  |  |  |  |  |  |
| rs10494275 | Diabetic polyneuropathy | 1 | 152391567 | A/G | 0.064 | 0.305 | 0.155 | 0.048 | 0.064 | cis |
| BDNF |  |  |  |  |  |  |  |  |  |  |
| rs10501089 | Diabetic polyneuropathy | 11 | 27788859 | T/C | 0.095 | 0.256 | 0.130 | 0.049 | 0.095 | cis |
| SMPD1 |  |  |  |  |  |  |  |  |  |  |
| rs1050239 | Diabetic polyneuropathy | 11 | 6415463 | A/G | 0.216 | -0.076 | 0.093 | 0.409 | 0.216 | cis |
| BLMH |  |  |  |  |  |  |  |  |  |  |
| rs1050565 | Diabetic polyneuropathy | 17 | 28576076 | C/T | 0.343 | 0.015 | 0.079 | 0.848 | 0.343 | cis |
| LIPN |  |  |  |  |  |  |  |  |  |  |
| rs10509554 | Diabetic polyneuropathy | 10 | 90525792 | T/C | 0.273 | 0.043 | 0.085 | 0.613 | 0.273 | cis |
| CLEC3B |  |  |  |  |  |  |  |  |  |  |
| rs10514712 | Diabetic polyneuropathy | 3 | 45083928 | T/C | 0.802 | -0.014 | 0.094 | 0.886 | 0.198 | cis |
| NCS1 |  |  |  |  |  |  |  |  |  |  |
| rs1054879 | Diabetic polyneuropathy | 9 | 132995768 | A/G | 0.581 | 0.029 | 0.080 | 0.714 | 0.419 | cis |
| CCDC134 |  |  |  |  |  |  |  |  |  |  |
| rs10608168 | Diabetic polyneuropathy | 22 | 42230349 | T/TCA | 0.591 | 0.067 | 0.077 | 0.381 | 0.409 | cis |
| CFH |  |  |  |  |  |  |  |  |  |  |
| rs10737680 | Diabetic polyneuropathy | 1 | 196679455 | C/A | 0.395 | 0.042 | 0.077 | 0.585 | 0.395 | cis |
| ASAH2 |  |  |  |  |  |  |  |  |  |  |
| rs10740617 | Diabetic polyneuropathy | 10 | 52027609 | C/A | 0.796 | -0.134 | 0.094 | 0.156 | 0.204 | cis |
| XCL2 |  |  |  |  |  |  |  |  |  |  |
| rs10753774 | Diabetic polyneuropathy | 1 | 168514008 | T/C | 0.385 | -0.093 | 0.078 | 0.234 | 0.385 | cis |
| VSIR |  |  |  |  |  |  |  |  |  |  |
| rs10762477 | Diabetic polyneuropathy | 10 | 73531069 | A/G | 0.865 | 0.026 | 0.111 | 0.813 | 0.136 | cis |
| rs10762476 | Diabetic polyneuropathy | 10 | 73530977 | A/C | 0.135 | -0.026 | 0.111 | 0.813 | 0.135 | cis |
| CALCA |  |  |  |  |  |  |  |  |  |  |
| rs10766197 | Diabetic polyneuropathy | 11 | 14921880 | A/G | 0.405 | 0.141 | 0.077 | 0.068 | 0.405 | cis |
| CALCB |  |  |  |  |  |  |  |  |  |  |
| rs10766205 | Diabetic polyneuropathy | 11 | 15092320 | G/A | 0.189 | -0.058 | 0.096 | 0.541 | 0.189 | cis |
| ART4 |  |  |  |  |  |  |  |  |  |  |
| rs10772808 | Diabetic polyneuropathy | 12 | 14990587 | A/G | 0.303 | -0.117 | 0.082 | 0.153 | 0.303 | cis |
| GOLM1 |  |  |  |  |  |  |  |  |  |  |
| rs10780749 | Diabetic polyneuropathy | 9 | 88720605 | A/G | 0.062 | 0.086 | 0.156 | 0.583 | 0.062 | cis |
| LIMA1 |  |  |  |  |  |  |  |  |  |  |
| rs10783342 | Diabetic polyneuropathy | 12 | 50628466 | T/C | 0.411 | -0.038 | 0.077 | 0.625 | 0.411 | cis |
| PLEKHA1 |  |  |  |  |  |  |  |  |  |  |
| rs10788274 | Diabetic polyneuropathy | 10 | 124060802 | A/G | 0.611 | -0.095 | 0.077 | 0.218 | 0.390 | cis |
| ENAH |  |  |  |  |  |  |  |  |  |  |
| rs10799316 | Diabetic polyneuropathy | 1 | 225668031 | A/G | 0.788 | 0.039 | 0.092 | 0.675 | 0.212 | cis |
| CD58 |  |  |  |  |  |  |  |  |  |  |
| rs10801908 | Diabetic polyneuropathy | 1 | 117090493 | T/C | 0.176 | 0.208 | 0.100 | 0.037 | 0.176 | cis |
| ROR2 |  |  |  |  |  |  |  |  |  |  |
| rs10820900 | Diabetic polyneuropathy | 9 | 94495608 | T/C | 0.378 | -0.086 | 0.078 | 0.271 | 0.378 | cis |
| PLA2G12B |  |  |  |  |  |  |  |  |  |  |
| rs10823949 | Diabetic polyneuropathy | 10 | 74632924 | T/C | 0.040 | -0.329 | 0.192 | 0.087 | 0.040 | cis |
| MRVI1 |  |  |  |  |  |  |  |  |  |  |
| rs10840457 | Diabetic polyneuropathy | 11 | 10675738 | A/G | 0.340 | 0.066 | 0.080 | 0.405 | 0.340 | cis |
| PTHLH |  |  |  |  |  |  |  |  |  |  |
| rs10843115 | Diabetic polyneuropathy | 12 | 28307717 | T/C | 0.238 | -0.040 | 0.088 | 0.649 | 0.238 | cis |
| LTBR |  |  |  |  |  |  |  |  |  |  |
| rs10849449 | Diabetic polyneuropathy | 12 | 6498662 | G/A | 0.700 | -0.166 | 0.083 | 0.046 | 0.300 | cis |
| PRTG |  |  |  |  |  |  |  |  |  |  |
| rs10851591 | Diabetic polyneuropathy | 15 | 56003705 | G/A | 0.652 | -0.005 | 0.080 | 0.953 | 0.348 | cis |
| ACOX1 |  |  |  |  |  |  |  |  |  |  |
| rs10852766 | Diabetic polyneuropathy | 17 | 73951864 | C/T | 0.755 | -0.079 | 0.088 | 0.370 | 0.245 | cis |
| IGFBP6 |  |  |  |  |  |  |  |  |  |  |
| rs10876406 | Diabetic polyneuropathy | 12 | 53501437 | C/T | 0.154 | -0.035 | 0.105 | 0.740 | 0.154 | cis |
| GRK5 |  |  |  |  |  |  |  |  |  |  |
| rs10886430 | Diabetic polyneuropathy | 10 | 121010256 | G/A | 0.095 | -0.084 | 0.130 | 0.517 | 0.095 | cis |
| FAS |  |  |  |  |  |  |  |  |  |  |
| rs7911226 | Diabetic polyneuropathy | 10 | 90768965 | A/G | 0.636 | 0.021 | 0.079 | 0.790 | 0.364 | cis |
| rs10887883 | Diabetic polyneuropathy | 10 | 90782973 | A/G | 0.418 | 0.015 | 0.077 | 0.846 | 0.418 | cis |
| CAPN1_CAPNS1 |  |  |  |  |  |  |  |  |  |  |
| rs10895987 | Diabetic polyneuropathy | 11 | 64904908 | T/C | 0.225 | -0.115 | 0.090 | 0.205 | 0.225 | cis |
| B4GAT1 |  |  |  |  |  |  |  |  |  |  |
| rs10896113 | Diabetic polyneuropathy | 11 | 66117111 | T/C | 0.235 | 0.044 | 0.089 | 0.619 | 0.235 | cis |
| FCGR3B |  |  |  |  |  |  |  |  |  |  |
| rs10919544 | Diabetic polyneuropathy | 1 | 161508763 | C/T | 0.267 | 0.127 | 0.086 | 0.141 | 0.267 | cis |
| FAM171B |  |  |  |  |  |  |  |  |  |  |
| rs10931256 | Diabetic polyneuropathy | 2 | 187685195 | C/T | 0.245 | -0.117 | 0.087 | 0.180 | 0.245 | cis |
| CERT |  |  |  |  |  |  |  |  |  |  |
| rs10942737 | Diabetic polyneuropathy | 5 | 74682813 | C/T | 0.069 | -0.081 | 0.147 | 0.583 | 0.069 | cis |
| MYORG |  |  |  |  |  |  |  |  |  |  |
| rs10972076 | Diabetic polyneuropathy | 9 | 34356359 | T/C | 0.583 | 0.043 | 0.077 | 0.578 | 0.417 | cis |
| CNTFR |  |  |  |  |  |  |  |  |  |  |
| rs10972159 | Diabetic polyneuropathy | 9 | 34593086 | A/G | 0.052 | -0.044 | 0.175 | 0.801 | 0.052 | cis |
| GPX7 |  |  |  |  |  |  |  |  |  |  |
| rs1097234 | Diabetic polyneuropathy | 1 | 53063559 | A/C | 0.151 | -0.128 | 0.105 | 0.225 | 0.151 | cis |
| GSN |  |  |  |  |  |  |  |  |  |  |
| rs10985196 | Diabetic polyneuropathy | 9 | 124033044 | A/C | 0.217 | -0.148 | 0.091 | 0.105 | 0.217 | cis |
| DKK3 |  |  |  |  |  |  |  |  |  |  |
| rs11022114 | Diabetic polyneuropathy | 11 | 12038874 | A/G | 0.346 | 0.176 | 0.079 | 0.026 | 0.346 | cis |
| SAA2 |  |  |  |  |  |  |  |  |  |  |
| rs11024589 | Diabetic polyneuropathy | 11 | 18278423 | A/C | 0.904 | 0.131 | 0.129 | 0.308 | 0.096 | cis |
| CGA_FSHB |  |  |  |  |  |  |  |  |  |  |
| rs11031006 | Diabetic polyneuropathy | 11 | 30226528 | A/G | 0.166 | 0.065 | 0.101 | 0.522 | 0.166 | cis |
| MPPED2 |  |  |  |  |  |  |  |  |  |  |
| rs11031140 | Diabetic polyneuropathy | 11 | 30608133 | A/G | 0.138 | 0.043 | 0.109 | 0.695 | 0.138 | cis |
| CLEC6A |  |  |  |  |  |  |  |  |  |  |
| rs11045427 | Diabetic polyneuropathy | 12 | 8606523 | A/C | 0.249 | -0.116 | 0.088 | 0.187 | 0.249 | cis |
| CD163 |  |  |  |  |  |  |  |  |  |  |
| rs11054859 | Diabetic polyneuropathy | 12 | 7769776 | A/G | 0.098 | 0.249 | 0.126 | 0.048 | 0.098 | cis |
| CLEC4C |  |  |  |  |  |  |  |  |  |  |
| rs11055602 | Diabetic polyneuropathy | 12 | 7904111 | G/T | 0.279 | 0.030 | 0.084 | 0.724 | 0.279 | cis |
| TNFRSF10B |  |  |  |  |  |  |  |  |  |  |
| rs1105944 | Diabetic polyneuropathy | 8 | 22885109 | G/A | 0.932 | -0.245 | 0.150 | 0.103 | 0.068 | cis |
| PLA2G1B |  |  |  |  |  |  |  |  |  |  |
| rs11065078 | Diabetic polyneuropathy | 12 | 120748585 | T/C | 0.226 | 0.052 | 0.090 | 0.562 | 0.226 | cis |
| PRPSAP2 |  |  |  |  |  |  |  |  |  |  |
| rs11078421 | Diabetic polyneuropathy | 17 | 18804385 | T/C | 0.605 | 0.028 | 0.077 | 0.717 | 0.395 | cis |
| SERPINF2 |  |  |  |  |  |  |  |  |  |  |
| rs11078597 | Diabetic polyneuropathy | 17 | 1618363 | T/C | 0.811 | -0.013 | 0.096 | 0.891 | 0.189 | cis |
| CNP |  |  |  |  |  |  |  |  |  |  |
| rs11079027 | Diabetic polyneuropathy | 17 | 40123521 | A/G | 0.349 | 0.091 | 0.080 | 0.255 | 0.349 | cis |
| DNAJB1 |  |  |  |  |  |  |  |  |  |  |
| rs11085896 | Diabetic polyneuropathy | 19 | 14651886 | C/A | 0.687 | -0.049 | 0.082 | 0.546 | 0.313 | cis |
| FBLN1 |  |  |  |  |  |  |  |  |  |  |
| rs11090631 | Diabetic polyneuropathy | 22 | 45846371 | T/C | 0.194 | 0.162 | 0.096 | 0.089 | 0.194 | cis |
| LCN1 |  |  |  |  |  |  |  |  |  |  |
| rs11103042 | Diabetic polyneuropathy | 9 | 138404116 | A/G | 0.137 | -0.080 | 0.110 | 0.467 | 0.137 | cis |
| FCN1 |  |  |  |  |  |  |  |  |  |  |
| rs11103602 | Diabetic polyneuropathy | 9 | 137854872 | A/G | 0.353 | 0.034 | 0.079 | 0.670 | 0.353 | cis |
| KITLG |  |  |  |  |  |  |  |  |  |  |
| rs11105121 | Diabetic polyneuropathy | 12 | 89364586 | T/C | 0.660 | 0.028 | 0.080 | 0.731 | 0.340 | cis |
| REG1B |  |  |  |  |  |  |  |  |  |  |
| rs11126696 | Diabetic polyneuropathy | 2 | 79323888 | G/A | 0.602 | 0.003 | 0.077 | 0.971 | 0.398 | cis |
| LDHA |  |  |  |  |  |  |  |  |  |  |
| rs111294128 | Diabetic polyneuropathy | 11 | 18411722 | A/G | 0.989 | 0.303 | 0.352 | 0.390 | 0.011 | cis |
| ENSA |  |  |  |  |  |  |  |  |  |  |
| rs111428433 | Diabetic polyneuropathy | 1 | 150527629 | T/C | 0.059 | -0.221 | 0.164 | 0.178 | 0.059 | cis |
| PCDH17 |  |  |  |  |  |  |  |  |  |  |
| rs11148472 | Diabetic polyneuropathy | 13 | 60020189 | C/A | 0.319 | 0.043 | 0.081 | 0.595 | 0.319 | cis |
| ITGAM |  |  |  |  |  |  |  |  |  |  |
| rs11150613 | Diabetic polyneuropathy | 16 | 31357810 | T/C | 0.596 | -0.008 | 0.077 | 0.922 | 0.404 | cis |
| LGALS3BP |  |  |  |  |  |  |  |  |  |  |
| rs111526614 | Diabetic polyneuropathy | 17 | 76962761 | T/C | 0.009 | -0.031 | 0.426 | 0.943 | 0.009 | cis |
| PDK2 |  |  |  |  |  |  |  |  |  |  |
| rs111564639 | Diabetic polyneuropathy | 17 | 48172095 | A/C | 0.044 | -0.078 | 0.188 | 0.678 | 0.044 | cis |
| NPTXR |  |  |  |  |  |  |  |  |  |  |
| rs111577133 | Diabetic polyneuropathy | 22 | 39241262 | G/A | 0.045 | -0.068 | 0.185 | 0.712 | 0.045 | cis |
| TIRAP |  |  |  |  |  |  |  |  |  |  |
| rs111577916 | Diabetic polyneuropathy | 11 | 126071349 | T/G | 0.037 | -0.072 | 0.203 | 0.725 | 0.037 | cis |
| VASH1 |  |  |  |  |  |  |  |  |  |  |
| rs11159226 | Diabetic polyneuropathy | 14 | 77181432 | G/T | 0.101 | -0.150 | 0.127 | 0.236 | 0.101 | cis |
| ENDOU |  |  |  |  |  |  |  |  |  |  |
| rs11168216 | Diabetic polyneuropathy | 12 | 48132176 | T/C | 0.750 | -0.123 | 0.088 | 0.163 | 0.250 | cis |
| LRIG3 |  |  |  |  |  |  |  |  |  |  |
| rs11172791 | Diabetic polyneuropathy | 12 | 59272973 | T/C | 0.962 | -0.074 | 0.194 | 0.702 | 0.038 | cis |
| KAZALD1 |  |  |  |  |  |  |  |  |  |  |
| rs11190812 | Diabetic polyneuropathy | 10 | 102824292 | A/G | 0.079 | -0.069 | 0.141 | 0.625 | 0.079 | cis |
| GFRA1 |  |  |  |  |  |  |  |  |  |  |
| rs11197603 | Diabetic polyneuropathy | 10 | 118001771 | C/T | 0.185 | 0.070 | 0.098 | 0.476 | 0.185 | cis |
| NAAA |  |  |  |  |  |  |  |  |  |  |
| rs111981122 | Diabetic polyneuropathy | 4 | 76855106 | A/C | 0.734 | -0.142 | 0.086 | 0.097 | 0.266 | cis |
| ANGPTL3 |  |  |  |  |  |  |  |  |  |  |
| rs11207970 | Diabetic polyneuropathy | 1 | 62915473 | T/C | 0.264 | 0.048 | 0.086 | 0.572 | 0.264 | cis |
| ACAT1 |  |  |  |  |  |  |  |  |  |  |
| rs112087419 | Diabetic polyneuropathy | 11 | 107998126 | A/G | 0.169 | 0.039 | 0.101 | 0.702 | 0.169 | cis |
| IL23R |  |  |  |  |  |  |  |  |  |  |
| rs11209026 | Diabetic polyneuropathy | 1 | 67705958 | A/G | 0.046 | 0.049 | 0.184 | 0.792 | 0.046 | cis |
| NCAM1 |  |  |  |  |  |  |  |  |  |  |
| rs11214489 | Diabetic polyneuropathy | 11 | 112975934 | C/T | 0.753 | -0.092 | 0.087 | 0.294 | 0.248 | cis |
| CADM1 |  |  |  |  |  |  |  |  |  |  |
| rs11215406 | Diabetic polyneuropathy | 11 | 115065082 | C/T | 0.267 | -0.045 | 0.085 | 0.600 | 0.267 | cis |
| CD6 |  |  |  |  |  |  |  |  |  |  |
| rs11230563 | Diabetic polyneuropathy | 11 | 60776209 | T/C | 0.248 | -0.209 | 0.088 | 0.017 | 0.248 | cis |
| MEGF10 |  |  |  |  |  |  |  |  |  |  |
| rs112394041 | Diabetic polyneuropathy | 5 | 126628584 | CT/C | 0.374 | -0.044 | 0.078 | 0.570 | 0.374 | cis |
| FSTL3 |  |  |  |  |  |  |  |  |  |  |
| rs112418024 | Diabetic polyneuropathy | 19 | 692790 | A/G | 0.066 | -0.024 | 0.155 | 0.875 | 0.066 | cis |
| IMPAD1 |  |  |  |  |  |  |  |  |  |  |
| rs112433249 | Diabetic polyneuropathy | 8 | 57876576 | C/T | 0.006 | 0.082 | 0.471 | 0.862 | 0.006 | cis |
| BPNT2 |  |  |  |  |  |  |  |  |  |  |
| rs112433249 | Diabetic polyneuropathy | 8 | 57876576 | C/T | 0.006 | 0.082 | 0.471 | 0.862 | 0.006 | cis |
| EPS8L2 |  |  |  |  |  |  |  |  |  |  |
| rs11246276 | Diabetic polyneuropathy | 11 | 706284 | T/G | 0.125 | -0.122 | 0.114 | 0.286 | 0.125 | cis |
| NAGLU |  |  |  |  |  |  |  |  |  |  |
| rs112629880 | Diabetic polyneuropathy | 17 | 40615761 | T/C | 0.091 | -0.055 | 0.133 | 0.678 | 0.091 | cis |
| PGM1 |  |  |  |  |  |  |  |  |  |  |
| rs1126728 | Diabetic polyneuropathy | 1 | 64097432 | T/C | 0.256 | 0.010 | 0.087 | 0.905 | 0.256 | cis |
| CCL16 |  |  |  |  |  |  |  |  |  |  |
| rs112689088 | Diabetic polyneuropathy | 17 | 34307457 | C/T | 0.068 | -0.040 | 0.152 | 0.794 | 0.068 | cis |
| MUC13 |  |  |  |  |  |  |  |  |  |  |
| rs1127233 | Diabetic polyneuropathy | 3 | 124627024 | G/T | 0.196 | 0.092 | 0.095 | 0.333 | 0.196 | cis |
| HEXIM2 |  |  |  |  |  |  |  |  |  |  |
| rs112981250 | Diabetic polyneuropathy | 17 | 43223316 | C/CAT | 0.665 | -0.111 | 0.080 | 0.168 | 0.335 | cis |
| MAPK9 |  |  |  |  |  |  |  |  |  |  |
| rs113096165 | Diabetic polyneuropathy | 5 | 179740366 | T/C | 0.190 | -0.037 | 0.097 | 0.705 | 0.190 | cis |
| PSG7 |  |  |  |  |  |  |  |  |  |  |
| rs113247044 | Diabetic polyneuropathy | 19 | 43439694 | A/G | 0.869 | -0.117 | 0.111 | 0.290 | 0.131 | cis |
| B3GALT6 |  |  |  |  |  |  |  |  |  |  |
| rs113272753 | Diabetic polyneuropathy | 1 | 1164749 | G/GC | 0.845 | 0.131 | 0.105 | 0.209 | 0.155 | cis |
| TNFRSF11A |  |  |  |  |  |  |  |  |  |  |
| rs113339733 | Diabetic polyneuropathy | 18 | 60018329 | A/G | 0.121 | -0.064 | 0.116 | 0.579 | 0.121 | cis |
| CCL8 |  |  |  |  |  |  |  |  |  |  |
| rs1133763 | Diabetic polyneuropathy | 17 | 32647831 | A/C | 0.848 | -0.069 | 0.105 | 0.509 | 0.152 | cis |
| EGFLAM |  |  |  |  |  |  |  |  |  |  |
| rs113400125 | Diabetic polyneuropathy | 5 | 38406251 | T/C | 0.021 | -0.293 | 0.273 | 0.284 | 0.021 | cis |
| SEZ6L2 |  |  |  |  |  |  |  |  |  |  |
| rs113443718 | Diabetic polyneuropathy | 16 | 29892184 | A/G | 0.314 | 0.074 | 0.082 | 0.368 | 0.314 | cis |
| TP53I3 |  |  |  |  |  |  |  |  |  |  |
| rs1134516 | Diabetic polyneuropathy | 2 | 24342532 | A/G | 0.207 | 0.018 | 0.094 | 0.849 | 0.207 | cis |
| P4HB |  |  |  |  |  |  |  |  |  |  |
| rs113708033 | Diabetic polyneuropathy | 17 | 79835930 | T/G | 0.152 | -0.133 | 0.105 | 0.208 | 0.152 | cis |
| MGAT4B |  |  |  |  |  |  |  |  |  |  |
| rs113756550 | Diabetic polyneuropathy | 5 | 179228377 | A/G | 0.004 | 0.140 | 0.623 | 0.822 | 0.004 | cis |
| NINJ1 |  |  |  |  |  |  |  |  |  |  |
| rs11379524 | Diabetic polyneuropathy | 9 | 95882745 | AC/A | 0.653 | 0.115 | 0.080 | 0.148 | 0.347 | cis |
| TNC |  |  |  |  |  |  |  |  |  |  |
| rs1138545 | Diabetic polyneuropathy | 9 | 117835899 | T/C | 0.159 | -0.053 | 0.104 | 0.607 | 0.159 | cis |
| NUB1 |  |  |  |  |  |  |  |  |  |  |
| rs113920358 | Diabetic polyneuropathy | 7 | 151038887 | T/C | 0.130 | 0.086 | 0.114 | 0.447 | 0.130 | cis |
| CKMT2 |  |  |  |  |  |  |  |  |  |  |
| rs114173713 | Diabetic polyneuropathy | 5 | 80562348 | T/C | 0.968 | 0.104 | 0.213 | 0.626 | 0.032 | cis |
| SCAMP3 |  |  |  |  |  |  |  |  |  |  |
| rs1142287 | Diabetic polyneuropathy | 1 | 155230131 | T/C | 0.276 | -0.051 | 0.085 | 0.548 | 0.276 | cis |
| FAHD1 |  |  |  |  |  |  |  |  |  |  |
| rs1143032 | Diabetic polyneuropathy | 16 | 1878031 | A/G | 0.249 | -0.078 | 0.088 | 0.375 | 0.249 | cis |
| ABLIM3 |  |  |  |  |  |  |  |  |  |  |
| rs114464628 | Diabetic polyneuropathy | 5 | 148533724 | A/G | 0.021 | 0.049 | 0.262 | 0.852 | 0.021 | cis |
| NLGN2 |  |  |  |  |  |  |  |  |  |  |
| rs114576150 | Diabetic polyneuropathy | 17 | 7307950 | T/G | 0.834 | -0.012 | 0.103 | 0.911 | 0.166 | cis |
| FSTL1 |  |  |  |  |  |  |  |  |  |  |
| rs1147707 | Diabetic polyneuropathy | 3 | 120169248 | T/C | 0.417 | -0.114 | 0.077 | 0.138 | 0.417 | cis |
| WWOX |  |  |  |  |  |  |  |  |  |  |
| rs11545029 | Diabetic polyneuropathy | 16 | 78420775 | A/G | 0.588 | -0.092 | 0.077 | 0.231 | 0.412 | cis |
| VWA1 |  |  |  |  |  |  |  |  |  |  |
| rs115503338 | Diabetic polyneuropathy | 1 | 1369623 | A/G | 0.008 | -0.159 | 0.423 | 0.708 | 0.008 | cis |
| ACP1 |  |  |  |  |  |  |  |  |  |  |
| rs11553746 | Diabetic polyneuropathy | 2 | 272203 | T/C | 0.361 | 0.026 | 0.079 | 0.738 | 0.361 | cis |
| FAH |  |  |  |  |  |  |  |  |  |  |
| rs11555096 | Diabetic polyneuropathy | 15 | 80472526 | T/C | 0.026 | 0.103 | 0.240 | 0.669 | 0.026 | cis |
| ADA |  |  |  |  |  |  |  |  |  |  |
| rs11555566 | Diabetic polyneuropathy | 20 | 43255220 | C/T | 0.045 | -0.130 | 0.180 | 0.470 | 0.045 | cis |
| NMRAL1 |  |  |  |  |  |  |  |  |  |  |
| rs11557236 | Diabetic polyneuropathy | 16 | 4519439 | A/G | 0.086 | 0.090 | 0.134 | 0.501 | 0.086 | cis |
| HNMT |  |  |  |  |  |  |  |  |  |  |
| rs11558538 | Diabetic polyneuropathy | 2 | 138759649 | T/C | 0.155 | 0.036 | 0.105 | 0.728 | 0.155 | cis |
| CKM_CKB |  |  |  |  |  |  |  |  |  |  |
| rs11559024 | Diabetic polyneuropathy | 19 | 45821183 | C/T | 0.019 | 1.124 | 0.305 | 0.000 | 0.019 | cis |
| CKM |  |  |  |  |  |  |  |  |  |  |
| rs11559024 | Diabetic polyneuropathy | 19 | 45821183 | C/T | 0.019 | 1.124 | 0.305 | 0.000 | 0.019 | cis |
| DPY30 |  |  |  |  |  |  |  |  |  |  |
| rs115631838 | Diabetic polyneuropathy | 2 | 32439307 | A/G | 0.027 | -0.061 | 0.228 | 0.790 | 0.027 | cis |
| PLXNC1 |  |  |  |  |  |  |  |  |  |  |
| rs115651556 | Diabetic polyneuropathy | 12 | 94613898 | A/G | 0.032 | 0.109 | 0.216 | 0.613 | 0.032 | cis |
| MMP7 |  |  |  |  |  |  |  |  |  |  |
| rs11568819 | Diabetic polyneuropathy | 11 | 102401633 | A/G | 0.063 | -0.121 | 0.157 | 0.441 | 0.063 | cis |
| C3 |  |  |  |  |  |  |  |  |  |  |
| rs11569415 | Diabetic polyneuropathy | 19 | 6716279 | A/G | 0.185 | -0.101 | 0.098 | 0.304 | 0.185 | cis |
| PF4 |  |  |  |  |  |  |  |  |  |  |
| rs11574452 | Diabetic polyneuropathy | 4 | 74846661 | A/C | 0.035 | -0.119 | 0.207 | 0.565 | 0.035 | cis |
| IL11RA |  |  |  |  |  |  |  |  |  |  |
| rs11575578 | Diabetic polyneuropathy | 9 | 34656479 | A/G | 0.072 | -0.150 | 0.145 | 0.300 | 0.072 | cis |
| SLAMF7 |  |  |  |  |  |  |  |  |  |  |
| rs11581248 | Diabetic polyneuropathy | 1 | 160720074 | T/C | 0.108 | -0.019 | 0.121 | 0.877 | 0.108 | cis |
| ADAM15 |  |  |  |  |  |  |  |  |  |  |
| rs11589479 | Diabetic polyneuropathy | 1 | 155033308 | A/G | 0.139 | -0.156 | 0.109 | 0.152 | 0.139 | cis |
| PCSK9 |  |  |  |  |  |  |  |  |  |  |
| rs11591147 | Diabetic polyneuropathy | 1 | 55505647 | T/G | 0.036 | 0.361 | 0.208 | 0.082 | 0.036 | cis |
| SPINK6 |  |  |  |  |  |  |  |  |  |  |
| rs1159203 | Diabetic polyneuropathy | 5 | 147619072 | A/C | 0.104 | -0.127 | 0.123 | 0.301 | 0.104 | cis |
| ENTPD1 |  |  |  |  |  |  |  |  |  |  |
| rs11598475 | Diabetic polyneuropathy | 10 | 97621266 | A/G | 0.308 | 0.025 | 0.082 | 0.758 | 0.308 | cis |
| CTSC |  |  |  |  |  |  |  |  |  |  |
| rs11600158 | Diabetic polyneuropathy | 11 | 88070914 | G/A | 0.070 | 0.266 | 0.147 | 0.071 | 0.070 | cis |
| SERPING1 |  |  |  |  |  |  |  |  |  |  |
| rs11603020 | Diabetic polyneuropathy | 11 | 57374332 | C/T | 0.305 | -0.058 | 0.082 | 0.484 | 0.305 | cis |
| SIRT3 |  |  |  |  |  |  |  |  |  |  |
| rs11604127 | Diabetic polyneuropathy | 11 | 196944 | T/C | 0.296 | -0.004 | 0.084 | 0.963 | 0.296 | cis |
| BET1L |  |  |  |  |  |  |  |  |  |  |
| rs11604127 | Diabetic polyneuropathy | 11 | 196944 | T/C | 0.296 | -0.004 | 0.084 | 0.963 | 0.296 | cis |
| CHRDL2 |  |  |  |  |  |  |  |  |  |  |
| rs11607100 | Diabetic polyneuropathy | 11 | 74414919 | T/C | 0.043 | 0.250 | 0.186 | 0.178 | 0.043 | cis |
| GGPS1 |  |  |  |  |  |  |  |  |  |  |
| rs116088703 | Diabetic polyneuropathy | 1 | 235498377 | A/G | 0.105 | -0.210 | 0.123 | 0.089 | 0.105 | cis |
| HYAL1 |  |  |  |  |  |  |  |  |  |  |
| rs116482870 | Diabetic polyneuropathy | 3 | 50339622 | T/C | 0.062 | -0.114 | 0.157 | 0.466 | 0.062 | cis |
| HSP90B1 |  |  |  |  |  |  |  |  |  |  |
| rs1165693 | Diabetic polyneuropathy | 12 | 104340204 | A/G | 0.279 | 0.098 | 0.084 | 0.243 | 0.279 | cis |
| TXNL4B |  |  |  |  |  |  |  |  |  |  |
| rs116891509 | Diabetic polyneuropathy | 16 | 72105560 | T/C | 0.071 | 0.028 | 0.148 | 0.851 | 0.071 | cis |
| CRYGD |  |  |  |  |  |  |  |  |  |  |
| rs11690925 | Diabetic polyneuropathy | 2 | 208977161 | T/C | 0.323 | -0.071 | 0.081 | 0.383 | 0.323 | cis |
| CLEC11A |  |  |  |  |  |  |  |  |  |  |
| rs116924815 | Diabetic polyneuropathy | 19 | 51230733 | T/C | 0.028 | -0.319 | 0.238 | 0.180 | 0.028 | cis |
| MATN3 |  |  |  |  |  |  |  |  |  |  |
| rs11694716 | Diabetic polyneuropathy | 2 | 20207619 | A/G | 0.691 | -0.073 | 0.082 | 0.372 | 0.309 | cis |
| PGP |  |  |  |  |  |  |  |  |  |  |
| rs116977380 | Diabetic polyneuropathy | 16 | 2263836 | T/C | 0.076 | 0.043 | 0.146 | 0.769 | 0.076 | cis |
| VOPP1 |  |  |  |  |  |  |  |  |  |  |
| rs117042408 | Diabetic polyneuropathy | 7 | 55623391 | A/G | 0.015 | 0.007 | 0.297 | 0.982 | 0.015 | cis |
| AP2A2 |  |  |  |  |  |  |  |  |  |  |
| rs117126836 | Diabetic polyneuropathy | 11 | 948299 | A/G | 0.114 | 0.045 | 0.118 | 0.705 | 0.114 | cis |
| PCOLCE2 |  |  |  |  |  |  |  |  |  |  |
| rs11716897 | Diabetic polyneuropathy | 3 | 142605556 | A/G | 0.379 | -0.056 | 0.078 | 0.469 | 0.379 | cis |
| KIAA1467 |  |  |  |  |  |  |  |  |  |  |
| rs117172138 | Diabetic polyneuropathy | 12 | 13193814 | T/C | 0.053 | 0.131 | 0.170 | 0.440 | 0.053 | cis |
| MANF |  |  |  |  |  |  |  |  |  |  |
| rs11721253 | Diabetic polyneuropathy | 3 | 51547219 | G/A | 0.870 | -0.009 | 0.112 | 0.935 | 0.130 | cis |
| CLEC4A |  |  |  |  |  |  |  |  |  |  |
| rs117213717 | Diabetic polyneuropathy | 12 | 8278195 | A/G | 0.006 | -0.614 | 0.484 | 0.205 | 0.006 | cis |
| LYAR |  |  |  |  |  |  |  |  |  |  |
| rs11723339 | Diabetic polyneuropathy | 4 | 4271886 | T/C | 0.643 | 0.074 | 0.079 | 0.350 | 0.357 | cis |
| HHIP |  |  |  |  |  |  |  |  |  |  |
| rs11727676 | Diabetic polyneuropathy | 4 | 145659064 | T/C | 0.914 | -0.148 | 0.134 | 0.268 | 0.086 | cis |
| ANGPTL2 |  |  |  |  |  |  |  |  |  |  |
| rs117360431 | Diabetic polyneuropathy | 9 | 129815495 | A/G | 0.011 | -0.333 | 0.366 | 0.362 | 0.011 | cis |
| CMBL |  |  |  |  |  |  |  |  |  |  |
| rs11738492 | Diabetic polyneuropathy | 5 | 10307022 | T/C | 0.594 | 0.033 | 0.077 | 0.668 | 0.406 | cis |
| IL6ST |  |  |  |  |  |  |  |  |  |  |
| rs11739016 | Diabetic polyneuropathy | 5 | 55263373 | T/C | 0.121 | -0.088 | 0.117 | 0.452 | 0.121 | cis |
| CA10 |  |  |  |  |  |  |  |  |  |  |
| rs117399000 | Diabetic polyneuropathy | 17 | 50213731 | A/G | 0.066 | -0.013 | 0.155 | 0.933 | 0.066 | cis |
| CLIP2 |  |  |  |  |  |  |  |  |  |  |
| rs117471007 | Diabetic polyneuropathy | 7 | 73696488 | T/C | 0.082 | -0.206 | 0.140 | 0.141 | 0.082 | cis |
| ACOT13 |  |  |  |  |  |  |  |  |  |  |
| rs11753290 | Diabetic polyneuropathy | 6 | 24714433 | A/G | 0.257 | 0.034 | 0.086 | 0.691 | 0.257 | cis |
| IFNGR1 |  |  |  |  |  |  |  |  |  |  |
| rs11754268 | Diabetic polyneuropathy | 6 | 137540335 | T/C | 0.213 | 0.078 | 0.093 | 0.404 | 0.213 | cis |
| NPTX2 |  |  |  |  |  |  |  |  |  |  |
| rs11773439 | Diabetic polyneuropathy | 7 | 98286099 | A/C | 0.689 | -0.057 | 0.081 | 0.485 | 0.311 | cis |
| GLT8D2 |  |  |  |  |  |  |  |  |  |  |
| rs117801489 | Diabetic polyneuropathy | 12 | 104408832 | C/T | 0.040 | -0.197 | 0.195 | 0.312 | 0.040 | cis |
| FABP5 |  |  |  |  |  |  |  |  |  |  |
| rs118019635 | Diabetic polyneuropathy | 8 | 82193282 | A/C | 0.142 | -0.053 | 0.110 | 0.628 | 0.142 | cis |
| ATRN |  |  |  |  |  |  |  |  |  |  |
| rs118065662 | Diabetic polyneuropathy | 20 | 3562866 | A/G | 0.973 | -0.194 | 0.235 | 0.408 | 0.027 | cis |
| INSL5 |  |  |  |  |  |  |  |  |  |  |
| rs11809759 | Diabetic polyneuropathy | 1 | 67269905 | A/G | 0.624 | -0.032 | 0.078 | 0.682 | 0.376 | cis |
| RNPEP |  |  |  |  |  |  |  |  |  |  |
| rs11810017 | Diabetic polyneuropathy | 1 | 201950949 | T/C | 0.724 | 0.008 | 0.084 | 0.927 | 0.276 | cis |
| CYB5D2 |  |  |  |  |  |  |  |  |  |  |
| rs118123280 | Diabetic polyneuropathy | 17 | 4060233 | A/G | 0.020 | 0.318 | 0.281 | 0.258 | 0.020 | cis |
| SERPINA9 |  |  |  |  |  |  |  |  |  |  |
| rs11850199 | Diabetic polyneuropathy | 14 | 94942663 | A/C | 0.191 | -0.094 | 0.095 | 0.322 | 0.191 | cis |
| ENPP7 |  |  |  |  |  |  |  |  |  |  |
| rs11871061 | Diabetic polyneuropathy | 17 | 77706544 | C/T | 0.374 | 0.200 | 0.078 | 0.011 | 0.374 | cis |
| CDH2 |  |  |  |  |  |  |  |  |  |  |
| rs11872284 | Diabetic polyneuropathy | 18 | 25680423 | T/C | 0.246 | 0.127 | 0.088 | 0.147 | 0.246 | cis |
| ALPPL2 |  |  |  |  |  |  |  |  |  |  |
| rs11886043 | Diabetic polyneuropathy | 2 | 233301535 | T/C | 0.717 | -0.072 | 0.084 | 0.392 | 0.283 | cis |
| LAMP3 |  |  |  |  |  |  |  |  |  |  |
| rs11921884 | Diabetic polyneuropathy | 3 | 182828245 | T/G | 0.668 | 0.080 | 0.080 | 0.319 | 0.332 | cis |
| SPINK2 |  |  |  |  |  |  |  |  |  |  |
| rs11941335 | Diabetic polyneuropathy | 4 | 57689460 | T/C | 0.014 | -0.263 | 0.312 | 0.399 | 0.014 | cis |
| LYPD8 |  |  |  |  |  |  |  |  |  |  |
| rs12044252 | Diabetic polyneuropathy | 1 | 249065691 | C/T | 0.064 | -0.089 | 0.151 | 0.556 | 0.064 | cis |
| PDK1 |  |  |  |  |  |  |  |  |  |  |
| rs12052479 | Diabetic polyneuropathy | 2 | 173404804 | A/C | 0.332 | 0.033 | 0.081 | 0.687 | 0.332 | cis |
| C1QA |  |  |  |  |  |  |  |  |  |  |
| rs12058824 | Diabetic polyneuropathy | 1 | 22963050 | A/G | 0.095 | 0.155 | 0.130 | 0.233 | 0.095 | cis |
| C8A_C8B_C8G |  |  |  |  |  |  |  |  |  |  |
| rs12067507 | Diabetic polyneuropathy | 1 | 57422511 | T/C | 0.037 | 0.014 | 0.199 | 0.944 | 0.037 | cis |
| FH |  |  |  |  |  |  |  |  |  |  |
| rs12071124 | Diabetic polyneuropathy | 1 | 241683705 | A/C | 0.763 | 0.065 | 0.088 | 0.462 | 0.237 | cis |
| EPHA10 |  |  |  |  |  |  |  |  |  |  |
| rs12074120 | Diabetic polyneuropathy | 1 | 38197401 | T/G | 0.743 | 0.016 | 0.087 | 0.856 | 0.257 | cis |
| AMY2B |  |  |  |  |  |  |  |  |  |  |
| rs12080068 | Diabetic polyneuropathy | 1 | 104329823 | A/C | 0.082 | -0.112 | 0.140 | 0.423 | 0.082 | cis |
| GBP1 |  |  |  |  |  |  |  |  |  |  |
| rs12089335 | Diabetic polyneuropathy | 1 | 89530425 | T/C | 0.191 | -0.159 | 0.096 | 0.097 | 0.191 | cis |
| AKR7A3 |  |  |  |  |  |  |  |  |  |  |
| rs12095284 | Diabetic polyneuropathy | 1 | 19571849 | T/C | 0.722 | 0.012 | 0.084 | 0.887 | 0.278 | cis |
| GALNT16 |  |  |  |  |  |  |  |  |  |  |
| rs12100668 | Diabetic polyneuropathy | 14 | 69793475 | G/A | 0.409 | 0.124 | 0.077 | 0.106 | 0.409 | cis |
| DDC |  |  |  |  |  |  |  |  |  |  |
| rs12112308 | Diabetic polyneuropathy | 7 | 50603487 | C/T | 0.010 | -0.760 | 0.366 | 0.038 | 0.010 | cis |
| AKR7A2 |  |  |  |  |  |  |  |  |  |  |
| rs12122880 | Diabetic polyneuropathy | 1 | 19661156 | T/C | 0.708 | 0.063 | 0.083 | 0.448 | 0.292 | cis |
| CD55 |  |  |  |  |  |  |  |  |  |  |
| rs12134133 | Diabetic polyneuropathy | 1 | 207457845 | A/G | 0.295 | -0.001 | 0.083 | 0.987 | 0.295 | cis |
| C1R |  |  |  |  |  |  |  |  |  |  |
| rs12146727 | Diabetic polyneuropathy | 12 | 7170336 | A/G | 0.173 | -0.046 | 0.100 | 0.651 | 0.173 | cis |
| C1S |  |  |  |  |  |  |  |  |  |  |
| rs12146727 | Diabetic polyneuropathy | 12 | 7170336 | A/G | 0.173 | -0.046 | 0.100 | 0.651 | 0.173 | cis |
| PLD3 |  |  |  |  |  |  |  |  |  |  |
| rs12151243 | Diabetic polyneuropathy | 19 | 40862872 | T/G | 0.201 | -0.028 | 0.095 | 0.765 | 0.201 | cis |
| ANKRD27 |  |  |  |  |  |  |  |  |  |  |
| rs12151311 | Diabetic polyneuropathy | 19 | 33112707 | T/C | 0.381 | 0.016 | 0.078 | 0.836 | 0.381 | cis |
| GNLY |  |  |  |  |  |  |  |  |  |  |
| rs12151621 | Diabetic polyneuropathy | 2 | 85934499 | A/C | 0.271 | -0.046 | 0.085 | 0.593 | 0.271 | cis |
| CCN4 |  |  |  |  |  |  |  |  |  |  |
| rs12156037 | Diabetic polyneuropathy | 8 | 134204458 | G/A | 0.418 | 0.011 | 0.077 | 0.885 | 0.418 | cis |
| PREP |  |  |  |  |  |  |  |  |  |  |
| rs12192369 | Diabetic polyneuropathy | 6 | 105777378 | G/A | 0.170 | 0.066 | 0.100 | 0.509 | 0.170 | cis |
| MTRF1L |  |  |  |  |  |  |  |  |  |  |
| rs12206911 | Diabetic polyneuropathy | 6 | 153323142 | T/C | 0.193 | 0.000 | 0.095 | 0.998 | 0.193 | cis |
| ITGB7 |  |  |  |  |  |  |  |  |  |  |
| rs12232003 | Diabetic polyneuropathy | 12 | 53593632 | C/T | 0.134 | 0.149 | 0.111 | 0.180 | 0.134 | cis |
| NQO1 |  |  |  |  |  |  |  |  |  |  |
| rs12232410 | Diabetic polyneuropathy | 16 | 69736851 | A/G | 0.185 | 0.013 | 0.097 | 0.898 | 0.185 | cis |
| GLRX3 |  |  |  |  |  |  |  |  |  |  |
| rs12248841 | Diabetic polyneuropathy | 10 | 131945252 | T/C | 0.634 | 0.037 | 0.078 | 0.638 | 0.366 | cis |
| CDHR5 |  |  |  |  |  |  |  |  |  |  |
| rs12360820 | Diabetic polyneuropathy | 11 | 620927 | C/A | 0.832 | -0.031 | 0.101 | 0.760 | 0.168 | cis |
| QSOX1 |  |  |  |  |  |  |  |  |  |  |
| rs12371 | Diabetic polyneuropathy | 1 | 180163390 | G/A | 0.076 | 0.023 | 0.141 | 0.870 | 0.076 | cis |
| IGSF8 |  |  |  |  |  |  |  |  |  |  |
| rs12408242 | Diabetic polyneuropathy | 1 | 160061934 | C/T | 0.260 | 0.124 | 0.086 | 0.150 | 0.260 | cis |
| NBL1 |  |  |  |  |  |  |  |  |  |  |
| rs12408663 | Diabetic polyneuropathy | 1 | 19968058 | C/T | 0.206 | -0.103 | 0.094 | 0.274 | 0.206 | cis |
| SERPINA10 |  |  |  |  |  |  |  |  |  |  |
| rs12434093 | Diabetic polyneuropathy | 14 | 94755184 | T/C | 0.811 | 0.164 | 0.097 | 0.090 | 0.189 | cis |
| PLCG2 |  |  |  |  |  |  |  |  |  |  |
| rs12445050 | Diabetic polyneuropathy | 16 | 81870969 | T/C | 0.130 | -0.033 | 0.113 | 0.769 | 0.130 | cis |
| CEACAM8 |  |  |  |  |  |  |  |  |  |  |
| rs12459454 | Diabetic polyneuropathy | 19 | 42263143 | G/A | 0.299 | 0.004 | 0.083 | 0.966 | 0.299 | cis |
| IGFLR1 |  |  |  |  |  |  |  |  |  |  |
| rs12459634 | Diabetic polyneuropathy | 19 | 36230174 | C/T | 0.084 | 0.186 | 0.133 | 0.162 | 0.084 | cis |
| ALPP |  |  |  |  |  |  |  |  |  |  |
| rs12478529 | Diabetic polyneuropathy | 2 | 233286654 | T/C | 0.283 | 0.073 | 0.084 | 0.388 | 0.283 | cis |
| RAB22A |  |  |  |  |  |  |  |  |  |  |
| rs12480383 | Diabetic polyneuropathy | 20 | 56887156 | T/C | 0.080 | 0.123 | 0.140 | 0.381 | 0.080 | cis |
| DCBLD2 |  |  |  |  |  |  |  |  |  |  |
| rs12487717 | Diabetic polyneuropathy | 3 | 98605007 | A/G | 0.001 | -0.957 | 1.005 | 0.341 | 0.001 | cis |
| FN1 |  |  |  |  |  |  |  |  |  |  |
| rs1250258 | Diabetic polyneuropathy | 2 | 216300185 | C/T | 0.212 | 0.023 | 0.092 | 0.800 | 0.212 | cis |
| AFP |  |  |  |  |  |  |  |  |  |  |
| rs12506899 | Diabetic polyneuropathy | 4 | 74319283 | G/T | 0.420 | -0.050 | 0.077 | 0.520 | 0.420 | cis |
| FGF5 |  |  |  |  |  |  |  |  |  |  |
| rs12509595 | Diabetic polyneuropathy | 4 | 81182554 | C/T | 0.312 | -0.035 | 0.081 | 0.668 | 0.312 | cis |
| LEAP2 |  |  |  |  |  |  |  |  |  |  |
| rs12515756 | Diabetic polyneuropathy | 5 | 132238880 | C/T | 0.137 | -0.076 | 0.109 | 0.486 | 0.137 | cis |
| CD83 |  |  |  |  |  |  |  |  |  |  |
| rs12526086 | Diabetic polyneuropathy | 6 | 14105897 | A/G | 0.212 | -0.025 | 0.093 | 0.789 | 0.212 | cis |
| ZYX |  |  |  |  |  |  |  |  |  |  |
| rs12539742 | Diabetic polyneuropathy | 7 | 143187097 | A/G | 0.262 | 0.037 | 0.086 | 0.667 | 0.262 | cis |
| SSC4D |  |  |  |  |  |  |  |  |  |  |
| rs12540573 | Diabetic polyneuropathy | 7 | 76039013 | C/A | 0.042 | 0.043 | 0.187 | 0.819 | 0.042 | cis |
| MSRA |  |  |  |  |  |  |  |  |  |  |
| rs12546887 | Diabetic polyneuropathy | 8 | 10292876 | T/C | 0.661 | 0.164 | 0.104 | 0.116 | 0.339 | cis |
| HOMER2 |  |  |  |  |  |  |  |  |  |  |
| rs1256430 | Diabetic polyneuropathy | 15 | 83519472 | A/G | 0.142 | -0.133 | 0.108 | 0.219 | 0.142 | cis |
| FCRL2 |  |  |  |  |  |  |  |  |  |  |
| rs12568320 | Diabetic polyneuropathy | 1 | 157744004 | G/A | 0.146 | 0.046 | 0.107 | 0.667 | 0.146 | cis |
| CST6 |  |  |  |  |  |  |  |  |  |  |
| rs12576095 | Diabetic polyneuropathy | 11 | 65776305 | A/G | 0.023 | -0.345 | 0.251 | 0.169 | 0.023 | cis |
| RNASE4 |  |  |  |  |  |  |  |  |  |  |
| rs12588573 | Diabetic polyneuropathy | 14 | 21146584 | T/C | 0.240 | 0.134 | 0.088 | 0.128 | 0.240 | cis |
| PPCDC |  |  |  |  |  |  |  |  |  |  |
| rs12591994 | Diabetic polyneuropathy | 15 | 75320306 | G/A | 0.102 | 0.151 | 0.126 | 0.233 | 0.102 | cis |
| CBLN1 |  |  |  |  |  |  |  |  |  |  |
| rs12596832 | Diabetic polyneuropathy | 16 | 49004958 | A/G | 0.133 | 0.074 | 0.111 | 0.505 | 0.133 | cis |
| NAGPA |  |  |  |  |  |  |  |  |  |  |
| rs12599777 | Diabetic polyneuropathy | 16 | 5079466 | G/A | 0.145 | -0.043 | 0.107 | 0.690 | 0.145 | cis |
| GCKR |  |  |  |  |  |  |  |  |  |  |
| rs1260326 | Diabetic polyneuropathy | 2 | 27730940 | T/C | 0.351 | -0.100 | 0.079 | 0.208 | 0.351 | cis |
| RAB31 |  |  |  |  |  |  |  |  |  |  |
| rs12607866 | Diabetic polyneuropathy | 18 | 9696474 | T/C | 0.657 | -0.183 | 0.081 | 0.023 | 0.344 | cis |
| METTL24 |  |  |  |  |  |  |  |  |  |  |
| rs12664330 | Diabetic polyneuropathy | 6 | 110681879 | A/G | 0.632 | 0.078 | 0.079 | 0.323 | 0.368 | cis |
| DNAJB6 |  |  |  |  |  |  |  |  |  |  |
| rs12668458 | Diabetic polyneuropathy | 7 | 157145474 | T/C | 0.580 | -0.065 | 0.077 | 0.402 | 0.420 | cis |
| EGFR |  |  |  |  |  |  |  |  |  |  |
| rs12669749 | Diabetic polyneuropathy | 7 | 55235804 | A/C | 0.117 | 0.005 | 0.118 | 0.966 | 0.117 | cis |
| GGH |  |  |  |  |  |  |  |  |  |  |
| rs12676348 | Diabetic polyneuropathy | 8 | 63943175 | T/C | 0.905 | -0.275 | 0.128 | 0.032 | 0.095 | cis |
| CTSV |  |  |  |  |  |  |  |  |  |  |
| rs12686511 | Diabetic polyneuropathy | 9 | 99909044 | T/C | 0.238 | 0.133 | 0.090 | 0.139 | 0.238 | cis |
| IL2RA |  |  |  |  |  |  |  |  |  |  |
| rs12722497 | Diabetic polyneuropathy | 10 | 6095928 | A/C | 0.068 | -0.066 | 0.150 | 0.662 | 0.068 | cis |
| HSPG2 |  |  |  |  |  |  |  |  |  |  |
| rs12742444 | Diabetic polyneuropathy | 1 | 22180723 | T/C | 0.047 | 0.005 | 0.178 | 0.979 | 0.047 | cis |
| ESAM |  |  |  |  |  |  |  |  |  |  |
| rs12792040 | Diabetic polyneuropathy | 11 | 124624150 | A/G | 0.104 | -0.033 | 0.125 | 0.790 | 0.104 | cis |
| CD69 |  |  |  |  |  |  |  |  |  |  |
| rs12819552 | Diabetic polyneuropathy | 12 | 9919832 | G/A | 0.388 | 0.107 | 0.077 | 0.165 | 0.388 | cis |
| SLITRK6 |  |  |  |  |  |  |  |  |  |  |
| rs12863734 | Diabetic polyneuropathy | 13 | 86370571 | A/G | 0.016 | 0.804 | 0.307 | 0.009 | 0.016 | cis |
| CA12 |  |  |  |  |  |  |  |  |  |  |
| rs12916532 | Diabetic polyneuropathy | 15 | 63674171 | C/T | 0.324 | 0.178 | 0.081 | 0.028 | 0.324 | cis |
| NPW |  |  |  |  |  |  |  |  |  |  |
| rs12921264 | Diabetic polyneuropathy | 16 | 2070433 | A/G | 0.212 | -0.108 | 0.093 | 0.247 | 0.212 | cis |
| CD79B |  |  |  |  |  |  |  |  |  |  |
| rs12946669 | Diabetic polyneuropathy | 17 | 62006007 | T/C | 0.369 | -0.033 | 0.078 | 0.676 | 0.369 | cis |
| COMP |  |  |  |  |  |  |  |  |  |  |
| rs12974746 | Diabetic polyneuropathy | 19 | 18906548 | G/A | 0.025 | 0.386 | 0.248 | 0.119 | 0.025 | cis |
| LILRB5 |  |  |  |  |  |  |  |  |  |  |
| rs12975366 | Diabetic polyneuropathy | 19 | 54759361 | C/T | 0.372 | 0.142 | 0.079 | 0.071 | 0.372 | cis |
| PSG1 |  |  |  |  |  |  |  |  |  |  |
| rs12986075 | Diabetic polyneuropathy | 19 | 43373034 | A/G | 0.189 | 0.065 | 0.098 | 0.508 | 0.189 | cis |
| MITD1 |  |  |  |  |  |  |  |  |  |  |
| rs13009147 | Diabetic polyneuropathy | 2 | 99803998 | G/T | 0.590 | 0.000 | 0.077 | 0.996 | 0.410 | cis |
| AHCY |  |  |  |  |  |  |  |  |  |  |
| rs13043752 | Diabetic polyneuropathy | 20 | 32883308 | A/G | 0.009 | 0.485 | 0.393 | 0.217 | 0.009 | cis |
| NIT2 |  |  |  |  |  |  |  |  |  |  |
| rs13073252 | Diabetic polyneuropathy | 3 | 100094208 | T/C | 0.320 | -0.082 | 0.081 | 0.311 | 0.320 | cis |
| KLB |  |  |  |  |  |  |  |  |  |  |
| rs13103023 | Diabetic polyneuropathy | 4 | 39457617 | A/G | 0.373 | -0.092 | 0.078 | 0.239 | 0.373 | cis |
| CXCL9 |  |  |  |  |  |  |  |  |  |  |
| rs13118503 | Diabetic polyneuropathy | 4 | 76860470 | T/C | 0.281 | 0.119 | 0.084 | 0.156 | 0.281 | cis |
| HEXB |  |  |  |  |  |  |  |  |  |  |
| rs13164140 | Diabetic polyneuropathy | 5 | 74030338 | A/G | 0.291 | -0.054 | 0.084 | 0.520 | 0.291 | cis |
| TYMP |  |  |  |  |  |  |  |  |  |  |
| rs131805 | Diabetic polyneuropathy | 22 | 50964153 | C/T | 0.818 | 0.022 | 0.098 | 0.824 | 0.182 | cis |
| CPA1 |  |  |  |  |  |  |  |  |  |  |
| rs13226219 | Diabetic polyneuropathy | 7 | 130019491 | C/T | 0.105 | 0.010 | 0.122 | 0.936 | 0.105 | cis |
| ENPP2 |  |  |  |  |  |  |  |  |  |  |
| rs13267597 | Diabetic polyneuropathy | 8 | 120654225 | G/T | 0.336 | -0.015 | 0.080 | 0.850 | 0.336 | cis |
| PTGES2 |  |  |  |  |  |  |  |  |  |  |
| rs13283456 | Diabetic polyneuropathy | 9 | 130884753 | T/C | 0.105 | -0.182 | 0.127 | 0.150 | 0.105 | cis |
| PDLIM1 |  |  |  |  |  |  |  |  |  |  |
| rs1328599 | Diabetic polyneuropathy | 10 | 97053775 | T/G | 0.929 | -0.061 | 0.147 | 0.677 | 0.071 | cis |
| TPPP3 |  |  |  |  |  |  |  |  |  |  |
| rs13334364 | Diabetic polyneuropathy | 16 | 67332365 | C/T | 0.130 | -0.162 | 0.115 | 0.158 | 0.130 | cis |
| EGLN1 |  |  |  |  |  |  |  |  |  |  |
| rs1339891 | Diabetic polyneuropathy | 1 | 231556395 | T/C | 0.040 | 0.103 | 0.189 | 0.584 | 0.040 | cis |
| SERPINE2 |  |  |  |  |  |  |  |  |  |  |
| rs13412535 | Diabetic polyneuropathy | 2 | 224874874 | A/G | 0.205 | -0.204 | 0.095 | 0.032 | 0.205 | cis |
| FUCA1 |  |  |  |  |  |  |  |  |  |  |
| rs13551 | Diabetic polyneuropathy | 1 | 24180962 | C/T | 0.396 | -0.063 | 0.077 | 0.413 | 0.396 | cis |
| SEZ6L |  |  |  |  |  |  |  |  |  |  |
| rs137203 | Diabetic polyneuropathy | 22 | 26688831 | T/G | 0.036 | 0.039 | 0.206 | 0.848 | 0.036 | cis |
| SLC27A4 |  |  |  |  |  |  |  |  |  |  |
| rs137948800 | Diabetic polyneuropathy | 9 | 131069717 | TA/T | 0.999 | 1.180 | 1.296 | 0.363 | 0.001 | cis |
| ATXN10 |  |  |  |  |  |  |  |  |  |  |
| rs138166 | Diabetic polyneuropathy | 22 | 46131238 | A/G | 0.268 | 0.032 | 0.085 | 0.705 | 0.268 | cis |
| DTD2 |  |  |  |  |  |  |  |  |  |  |
| rs138832021 | Diabetic polyneuropathy | 14 | 31906728 | A/C | 0.103 | -0.189 | 0.124 | 0.127 | 0.103 | cis |
| FLII |  |  |  |  |  |  |  |  |  |  |
| rs138865936 | Diabetic polyneuropathy | 17 | 18165019 | T/C | 0.023 | 0.083 | 0.257 | 0.745 | 0.023 | cis |
| ADGRE5 |  |  |  |  |  |  |  |  |  |  |
| rs139113505 | Diabetic polyneuropathy | 19 | 14499616 | T/C | 0.026 | 0.017 | 0.242 | 0.944 | 0.026 | cis |
| CCL20 |  |  |  |  |  |  |  |  |  |  |
| rs1395338 | Diabetic polyneuropathy | 2 | 228648323 | A/G | 0.733 | -0.086 | 0.085 | 0.314 | 0.267 | cis |
| VCAM1 |  |  |  |  |  |  |  |  |  |  |
| rs139561173 | Diabetic polyneuropathy | 1 | 101145190 | C/A | 0.222 | 0.001 | 0.091 | 0.990 | 0.222 | cis |
| CGA_LHB |  |  |  |  |  |  |  |  |  |  |
| rs139643250 | Diabetic polyneuropathy | 19 | 49517146 | T/C | 0.141 | -0.095 | 0.110 | 0.385 | 0.141 | cis |
| GXYLT1 |  |  |  |  |  |  |  |  |  |  |
| rs139695917 | Diabetic polyneuropathy | 12 | 42527041 | T/C | 0.981 | 0.138 | 0.275 | 0.616 | 0.019 | cis |
| DNMBP |  |  |  |  |  |  |  |  |  |  |
| rs139736391 | Diabetic polyneuropathy | 10 | 101452711 | T/C | 0.032 | -0.167 | 0.216 | 0.441 | 0.032 | cis |
| DDT |  |  |  |  |  |  |  |  |  |  |
| rs140117085 | Diabetic polyneuropathy | 22 | 24298603 | T/C | 0.013 | -0.293 | 0.332 | 0.378 | 0.013 | cis |
| PTK7 |  |  |  |  |  |  |  |  |  |  |
| rs140147428 | Diabetic polyneuropathy | 6 | 43067044 | T/C | 0.024 | 0.134 | 0.240 | 0.578 | 0.024 | cis |
| EDAR |  |  |  |  |  |  |  |  |  |  |
| rs140661471 | Diabetic polyneuropathy | 2 | 109603739 | A/G | 0.008 | 1.010 | 0.414 | 0.015 | 0.008 | cis |
| ALDOB |  |  |  |  |  |  |  |  |  |  |
| rs142540788 | Diabetic polyneuropathy | 9 | 104209068 | A/G | 0.971 | 0.045 | 0.231 | 0.847 | 0.029 | cis |
| NEK7 |  |  |  |  |  |  |  |  |  |  |
| rs142662761 | Diabetic polyneuropathy | 1 | 198010781 | A/G | 0.988 | -0.429 | 0.337 | 0.203 | 0.012 | cis |
| ADIPOQ |  |  |  |  |  |  |  |  |  |  |
| rs143257534 | Diabetic polyneuropathy | 3 | 186551888 | T/C | 0.025 | 0.133 | 0.243 | 0.585 | 0.025 | cis |
| PRTFDC1 |  |  |  |  |  |  |  |  |  |  |
| rs143356584 | Diabetic polyneuropathy | 10 | 25241502 | T/C | 0.030 | -0.292 | 0.219 | 0.182 | 0.030 | cis |
| PARP1 |  |  |  |  |  |  |  |  |  |  |
| rs1433574 | Diabetic polyneuropathy | 1 | 226609339 | C/A | 0.273 | 0.187 | 0.085 | 0.027 | 0.273 | cis |
| COL18A1 |  |  |  |  |  |  |  |  |  |  |
| rs144147445 | Diabetic polyneuropathy | 21 | 46930092 | A/G | 0.008 | 0.458 | 0.435 | 0.293 | 0.008 | cis |
| ITGB5 |  |  |  |  |  |  |  |  |  |  |
| rs144195265 | Diabetic polyneuropathy | 3 | 124509367 | A/G | 0.023 | -0.069 | 0.251 | 0.783 | 0.023 | cis |
| RCL |  |  |  |  |  |  |  |  |  |  |
| rs144767533 | Diabetic polyneuropathy | 6 | 43186138 | C/T | 0.092 | 0.047 | 0.129 | 0.714 | 0.092 | cis |
| ACADM |  |  |  |  |  |  |  |  |  |  |
| rs145024038 | Diabetic polyneuropathy | 1 | 76195431 | T/TG | 0.739 | 0.178 | 0.086 | 0.039 | 0.261 | cis |
| BOLA1 |  |  |  |  |  |  |  |  |  |  |
| rs1451641 | Diabetic polyneuropathy | 1 | 149865880 | A/G | 0.944 | -0.300 | 0.165 | 0.068 | 0.056 | cis |
| OVCA2 |  |  |  |  |  |  |  |  |  |  |
| rs145234879 | Diabetic polyneuropathy | 17 | 1945354 | A/C | 0.022 | 0.196 | 0.266 | 0.461 | 0.022 | cis |
| NPPA |  |  |  |  |  |  |  |  |  |  |
| rs145488887 | Diabetic polyneuropathy | 1 | 11827796 | C/T | 0.094 | -0.116 | 0.131 | 0.376 | 0.094 | cis |
| AKR1C1 |  |  |  |  |  |  |  |  |  |  |
| rs145648894 | Diabetic polyneuropathy | 10 | 5009739 | G/T | 0.132 | -0.060 | 0.112 | 0.590 | 0.132 | cis |
| SYT11 |  |  |  |  |  |  |  |  |  |  |
| rs145976521 | Diabetic polyneuropathy | 1 | 155816085 | A/G | 0.137 | -0.194 | 0.112 | 0.082 | 0.137 | cis |
| RIDA |  |  |  |  |  |  |  |  |  |  |
| rs1462977 | Diabetic polyneuropathy | 8 | 99115359 | G/A | 0.321 | 0.032 | 0.081 | 0.690 | 0.321 | cis |
| ADH7 |  |  |  |  |  |  |  |  |  |  |
| rs146315698 | Diabetic polyneuropathy | 4 | 100377127 | C/CT | 0.136 | -0.159 | 0.110 | 0.147 | 0.136 | cis |
| IL17RD |  |  |  |  |  |  |  |  |  |  |
| rs1463657 | Diabetic polyneuropathy | 3 | 57137461 | C/A | 0.262 | -0.064 | 0.086 | 0.454 | 0.262 | cis |
| MRC2 |  |  |  |  |  |  |  |  |  |  |
| rs146385050 | Diabetic polyneuropathy | 17 | 60637258 | A/C | 0.161 | 0.127 | 0.103 | 0.220 | 0.161 | cis |
| GP5 |  |  |  |  |  |  |  |  |  |  |
| rs1466733 | Diabetic polyneuropathy | 3 | 194120998 | A/G | 0.756 | 0.011 | 0.088 | 0.905 | 0.244 | cis |
| VIT |  |  |  |  |  |  |  |  |  |  |
| rs1468810 | Diabetic polyneuropathy | 2 | 36993940 | C/A | 0.416 | -0.023 | 0.077 | 0.764 | 0.416 | cis |
| FOLR2 |  |  |  |  |  |  |  |  |  |  |
| rs146893981 | Diabetic polyneuropathy | 11 | 71917353 | T/C | 0.036 | -0.025 | 0.200 | 0.900 | 0.036 | cis |
| RNASE3 |  |  |  |  |  |  |  |  |  |  |
| rs147307766 | Diabetic polyneuropathy | 14 | 21385991 | T/C | 0.030 | 0.165 | 0.216 | 0.445 | 0.030 | cis |
| LGALS8 |  |  |  |  |  |  |  |  |  |  |
| rs1475133 | Diabetic polyneuropathy | 1 | 236716686 | A/G | 0.775 | -0.071 | 0.090 | 0.429 | 0.226 | cis |
| IGLON5 |  |  |  |  |  |  |  |  |  |  |
| rs148108087 | Diabetic polyneuropathy | 19 | 51781455 | A/G | 0.078 | 0.137 | 0.142 | 0.334 | 0.078 | cis |
| GLRX2 |  |  |  |  |  |  |  |  |  |  |
| rs148212596 | Diabetic polyneuropathy | 1 | 193074511 | G/A | 0.007 | 0.302 | 0.434 | 0.486 | 0.007 | cis |
| DTYMK |  |  |  |  |  |  |  |  |  |  |
| rs148221699 | Diabetic polyneuropathy | 2 | 242630880 | A/C | 0.029 | -0.066 | 0.227 | 0.772 | 0.029 | cis |
| LGMN |  |  |  |  |  |  |  |  |  |  |
| rs148659834 | Diabetic polyneuropathy | 14 | 93176042 | A/G | 0.015 | -0.250 | 0.319 | 0.433 | 0.015 | cis |
| SIGLEC10 |  |  |  |  |  |  |  |  |  |  |
| rs148783636 | Diabetic polyneuropathy | 19 | 51910979 | C/T | 0.018 | -0.438 | 0.285 | 0.124 | 0.018 | cis |
| PON3 |  |  |  |  |  |  |  |  |  |  |
| rs149867961 | Diabetic polyneuropathy | 7 | 95025744 | C/T | 0.015 | 0.202 | 0.309 | 0.514 | 0.015 | cis |
| BIN2 |  |  |  |  |  |  |  |  |  |  |
| rs149960700 | Diabetic polyneuropathy | 12 | 51705168 | CTTATTTA/C | 0.071 | -0.064 | 0.146 | 0.661 | 0.071 | cis |
| CAPN2 |  |  |  |  |  |  |  |  |  |  |
| rs150394890 | Diabetic polyneuropathy | 1 | 223864380 | T/G | 0.016 | -0.012 | 0.299 | 0.968 | 0.016 | cis |
| ACY1 |  |  |  |  |  |  |  |  |  |  |
| rs150416778 | Diabetic polyneuropathy | 3 | 52016836 | T/C | 0.007 | -0.475 | 0.467 | 0.310 | 0.007 | cis |
| LPA |  |  |  |  |  |  |  |  |  |  |
| rs151292106 | Diabetic polyneuropathy | 6 | 160943149 | C/T | 0.988 | 0.327 | 0.347 | 0.346 | 0.012 | cis |
| rs41259144 | Diabetic polyneuropathy | 6 | 161022107 | T/C | 0.006 | 0.161 | 0.495 | 0.745 | 0.006 | cis |
| rs55730499 | Diabetic polyneuropathy | 6 | 161005610 | C/T | 0.954 | -0.033 | 0.178 | 0.852 | 0.046 | cis |
| rs4646272 | Diabetic polyneuropathy | 6 | 160551093 | T/G | 0.938 | -0.070 | 0.156 | 0.652 | 0.062 | cis |
| GPC6 |  |  |  |  |  |  |  |  |  |  |
| rs1535692 | Diabetic polyneuropathy | 13 | 95034749 | A/G | 0.273 | 0.115 | 0.085 | 0.178 | 0.273 | cis |
| LSAMP |  |  |  |  |  |  |  |  |  |  |
| rs1541871 | Diabetic polyneuropathy | 3 | 116102816 | T/C | 0.219 | -0.083 | 0.092 | 0.367 | 0.219 | cis |
| CPE |  |  |  |  |  |  |  |  |  |  |
| rs1550270 | Diabetic polyneuropathy | 4 | 166261800 | C/T | 0.276 | 0.154 | 0.084 | 0.067 | 0.276 | cis |
| A2ML1 |  |  |  |  |  |  |  |  |  |  |
| rs1558526 | Diabetic polyneuropathy | 12 | 9009820 | A/G | 0.171 | -0.034 | 0.100 | 0.733 | 0.171 | cis |
| ATP1B2 |  |  |  |  |  |  |  |  |  |  |
| rs1642762 | Diabetic polyneuropathy | 17 | 7554772 | T/C | 0.595 | -0.095 | 0.077 | 0.217 | 0.405 | cis |
| GP6 |  |  |  |  |  |  |  |  |  |  |
| rs1654439 | Diabetic polyneuropathy | 19 | 55553647 | T/G | 0.132 | -0.182 | 0.111 | 0.102 | 0.132 | cis |
| SPOCK2 |  |  |  |  |  |  |  |  |  |  |
| rs1668153 | Diabetic polyneuropathy | 10 | 73844563 | A/G | 0.599 | 0.010 | 0.077 | 0.902 | 0.401 | cis |
| CPOX |  |  |  |  |  |  |  |  |  |  |
| rs1675513 | Diabetic polyneuropathy | 3 | 98308698 | G/A | 0.232 | -0.010 | 0.090 | 0.916 | 0.232 | cis |
| GALNT13 |  |  |  |  |  |  |  |  |  |  |
| rs16834610 | Diabetic polyneuropathy | 2 | 154730495 | T/C | 0.032 | -0.321 | 0.210 | 0.126 | 0.032 | cis |
| CXCL6 |  |  |  |  |  |  |  |  |  |  |
| rs16850073 | Diabetic polyneuropathy | 4 | 74703999 | T/C | 0.385 | 0.037 | 0.078 | 0.638 | 0.385 | cis |
| FMOD |  |  |  |  |  |  |  |  |  |  |
| rs16851364 | Diabetic polyneuropathy | 1 | 203318144 | A/G | 0.100 | -0.080 | 0.126 | 0.529 | 0.100 | cis |
| CD200R1 |  |  |  |  |  |  |  |  |  |  |
| rs16860233 | Diabetic polyneuropathy | 3 | 112642122 | T/C | 0.022 | -0.295 | 0.261 | 0.259 | 0.022 | cis |
| BMPER |  |  |  |  |  |  |  |  |  |  |
| rs16879245 | Diabetic polyneuropathy | 7 | 33804485 | A/G | 0.908 | -0.147 | 0.133 | 0.268 | 0.092 | cis |
| CLEC7A |  |  |  |  |  |  |  |  |  |  |
| rs16910526 | Diabetic polyneuropathy | 12 | 10271087 | C/A | 0.058 | 0.221 | 0.160 | 0.168 | 0.058 | cis |
| AREG |  |  |  |  |  |  |  |  |  |  |
| rs1691273 | Diabetic polyneuropathy | 4 | 75323645 | T/C | 0.799 | -0.124 | 0.094 | 0.187 | 0.201 | cis |
| GSTP1 |  |  |  |  |  |  |  |  |  |  |
| rs1695 | Diabetic polyneuropathy | 11 | 67352689 | G/A | 0.283 | -0.074 | 0.084 | 0.378 | 0.283 | cis |
| CCL17 |  |  |  |  |  |  |  |  |  |  |
| rs16956811 | Diabetic polyneuropathy | 16 | 57444002 | T/G | 0.950 | -0.177 | 0.172 | 0.305 | 0.050 | cis |
| CCL3L1 |  |  |  |  |  |  |  |  |  |  |
| rs16971895 | Diabetic polyneuropathy | 17 | 34386399 | A/G | 0.046 | -0.016 | 0.181 | 0.931 | 0.046 | cis |
| GZMM |  |  |  |  |  |  |  |  |  |  |
| rs16989724 | Diabetic polyneuropathy | 19 | 531115 | T/C | 0.948 | 0.021 | 0.172 | 0.902 | 0.052 | cis |
| CBR1 |  |  |  |  |  |  |  |  |  |  |
| rs16993864 | Diabetic polyneuropathy | 21 | 37446599 | A/C | 0.009 | -0.329 | 0.419 | 0.432 | 0.009 | cis |
| CRHBP |  |  |  |  |  |  |  |  |  |  |
| rs1700658 | Diabetic polyneuropathy | 5 | 76210859 | A/G | 0.642 | -0.011 | 0.080 | 0.887 | 0.358 | cis |
| FABP2 |  |  |  |  |  |  |  |  |  |  |
| rs17009129 | Diabetic polyneuropathy | 4 | 120280617 | T/C | 0.367 | -0.031 | 0.078 | 0.698 | 0.367 | cis |
| MEPE |  |  |  |  |  |  |  |  |  |  |
| rs17013212 | Diabetic polyneuropathy | 4 | 88754060 | T/C | 0.202 | -0.008 | 0.096 | 0.937 | 0.202 | cis |
| ISCU |  |  |  |  |  |  |  |  |  |  |
| rs17040770 | Diabetic polyneuropathy | 12 | 108955106 | C/CT | 0.146 | -0.013 | 0.108 | 0.903 | 0.146 | cis |
| ARSK |  |  |  |  |  |  |  |  |  |  |
| rs17084933 | Diabetic polyneuropathy | 5 | 94942780 | T/C | 0.951 | 0.271 | 0.176 | 0.123 | 0.049 | cis |
| SERPINA12 |  |  |  |  |  |  |  |  |  |  |
| rs17091005 | Diabetic polyneuropathy | 14 | 94988407 | C/T | 0.209 | 0.020 | 0.093 | 0.831 | 0.209 | cis |
| ENTPD5 |  |  |  |  |  |  |  |  |  |  |
| rs17094448 | Diabetic polyneuropathy | 14 | 74440119 | G/A | 0.072 | -0.085 | 0.148 | 0.567 | 0.072 | cis |
| CALCOCO1 |  |  |  |  |  |  |  |  |  |  |
| rs17102261 | Diabetic polyneuropathy | 12 | 54126732 | G/T | 0.302 | 0.010 | 0.083 | 0.907 | 0.302 | cis |
| REXO2 |  |  |  |  |  |  |  |  |  |  |
| rs17116987 | Diabetic polyneuropathy | 11 | 114301927 | T/C | 0.892 | -0.067 | 0.121 | 0.583 | 0.108 | cis |
| SAMD9L |  |  |  |  |  |  |  |  |  |  |
| rs17165120 | Diabetic polyneuropathy | 7 | 92777091 | C/A | 0.040 | 0.004 | 0.191 | 0.985 | 0.040 | cis |
| CD300E |  |  |  |  |  |  |  |  |  |  |
| rs1719465 | Diabetic polyneuropathy | 17 | 72576391 | C/T | 0.703 | -0.043 | 0.083 | 0.605 | 0.297 | cis |
| C5 |  |  |  |  |  |  |  |  |  |  |
| rs17220750 | Diabetic polyneuropathy | 9 | 123787999 | A/G | 0.084 | 0.161 | 0.136 | 0.237 | 0.084 | cis |
| RNASE1 |  |  |  |  |  |  |  |  |  |  |
| rs17254387 | Diabetic polyneuropathy | 14 | 21280678 | A/G | 0.736 | -0.019 | 0.086 | 0.826 | 0.264 | cis |
| NTN4 |  |  |  |  |  |  |  |  |  |  |
| rs17288108 | Diabetic polyneuropathy | 12 | 96131895 | A/G | 0.829 | 0.048 | 0.099 | 0.627 | 0.171 | cis |
| SCRN1 |  |  |  |  |  |  |  |  |  |  |
| rs17324153 | Diabetic polyneuropathy | 7 | 29966141 | C/T | 0.027 | 0.105 | 0.230 | 0.648 | 0.027 | cis |
| CTSB |  |  |  |  |  |  |  |  |  |  |
| rs1736084 | Diabetic polyneuropathy | 8 | 11703657 | T/C | 0.195 | -0.190 | 0.105 | 0.070 | 0.195 | cis |
| PAPPA |  |  |  |  |  |  |  |  |  |  |
| rs17372936 | Diabetic polyneuropathy | 9 | 119066203 | T/C | 0.783 | 0.019 | 0.091 | 0.833 | 0.217 | cis |
| PPIC |  |  |  |  |  |  |  |  |  |  |
| rs17388251 | Diabetic polyneuropathy | 5 | 122360403 | T/C | 0.650 | 0.040 | 0.079 | 0.615 | 0.351 | cis |
| FCGR2A_FCGR2B |  |  |  |  |  |  |  |  |  |  |
| rs17413015 | Diabetic polyneuropathy | 1 | 161644811 | T/C | 0.164 | -0.186 | 0.102 | 0.069 | 0.164 | cis |
| LEPR |  |  |  |  |  |  |  |  |  |  |
| rs17415296 | Diabetic polyneuropathy | 1 | 66099013 | A/C | 0.120 | 0.048 | 0.116 | 0.682 | 0.120 | cis |
| FAM3D |  |  |  |  |  |  |  |  |  |  |
| rs17551388 | Diabetic polyneuropathy | 3 | 58652970 | A/C | 0.123 | -0.063 | 0.115 | 0.581 | 0.123 | cis |
| PEBP1 |  |  |  |  |  |  |  |  |  |  |
| rs17619811 | Diabetic polyneuropathy | 12 | 118733582 | T/G | 0.007 | -0.613 | 0.464 | 0.187 | 0.007 | cis |
| TGFA |  |  |  |  |  |  |  |  |  |  |
| rs17639251 | Diabetic polyneuropathy | 2 | 70776281 | C/T | 0.105 | 0.287 | 0.122 | 0.018 | 0.105 | cis |
| FLRT2 |  |  |  |  |  |  |  |  |  |  |
| rs17646457 | Diabetic polyneuropathy | 14 | 86089315 | A/G | 0.179 | 0.122 | 0.099 | 0.220 | 0.179 | cis |
| SPINT1 |  |  |  |  |  |  |  |  |  |  |
| rs17658212 | Diabetic polyneuropathy | 15 | 41145919 | T/C | 0.026 | -0.270 | 0.244 | 0.269 | 0.026 | cis |
| HS3ST3B1 |  |  |  |  |  |  |  |  |  |  |
| rs17669311 | Diabetic polyneuropathy | 17 | 13837051 | A/G | 0.321 | -0.014 | 0.081 | 0.865 | 0.321 | cis |
| BCHE |  |  |  |  |  |  |  |  |  |  |
| rs17713088 | Diabetic polyneuropathy | 3 | 165488604 | T/G | 0.174 | -0.015 | 0.099 | 0.878 | 0.174 | cis |
| CLN5 |  |  |  |  |  |  |  |  |  |  |
| rs1773045 | Diabetic polyneuropathy | 13 | 77584886 | A/G | 0.098 | 0.119 | 0.126 | 0.343 | 0.098 | cis |
| CANT1 |  |  |  |  |  |  |  |  |  |  |
| rs17739056 | Diabetic polyneuropathy | 17 | 76995205 | C/T | 0.095 | 0.156 | 0.129 | 0.226 | 0.095 | cis |
| CA13 |  |  |  |  |  |  |  |  |  |  |
| rs17741049 | Diabetic polyneuropathy | 8 | 86198253 | T/C | 0.049 | -0.276 | 0.175 | 0.115 | 0.049 | cis |
| MAP4K5 |  |  |  |  |  |  |  |  |  |  |
| rs17780143 | Diabetic polyneuropathy | 14 | 50901768 | A/G | 0.028 | -0.070 | 0.231 | 0.761 | 0.028 | cis |
| BPI |  |  |  |  |  |  |  |  |  |  |
| rs1780617 | Diabetic polyneuropathy | 20 | 36974157 | G/A | 0.120 | 0.003 | 0.117 | 0.981 | 0.120 | cis |
| CNDP1 |  |  |  |  |  |  |  |  |  |  |
| rs17817077 | Diabetic polyneuropathy | 18 | 72209543 | A/G | 0.347 | -0.022 | 0.080 | 0.778 | 0.347 | cis |
| QPCTL |  |  |  |  |  |  |  |  |  |  |
| rs17850756 | Diabetic polyneuropathy | 19 | 46206262 | A/G | 0.278 | 0.118 | 0.085 | 0.164 | 0.278 | cis |
| MMP10 |  |  |  |  |  |  |  |  |  |  |
| rs17860955 | Diabetic polyneuropathy | 11 | 102649482 | T/C | 0.996 | 0.419 | 0.552 | 0.448 | 0.004 | cis |
| IL16 |  |  |  |  |  |  |  |  |  |  |
| rs17875523 | Diabetic polyneuropathy | 15 | 81596552 | T/C | 0.044 | -0.091 | 0.178 | 0.609 | 0.044 | cis |
| LRPAP1 |  |  |  |  |  |  |  |  |  |  |
| rs1800493 | Diabetic polyneuropathy | 4 | 3516559 | T/C | 0.013 | -0.173 | 0.330 | 0.600 | 0.013 | cis |
| LPL |  |  |  |  |  |  |  |  |  |  |
| rs1801177 | Diabetic polyneuropathy | 8 | 19805708 | A/G | 0.003 | -0.453 | 0.715 | 0.526 | 0.003 | cis |
| APOH |  |  |  |  |  |  |  |  |  |  |
| rs1801689 | Diabetic polyneuropathy | 17 | 64210580 | A/C | 0.990 | 0.158 | 0.383 | 0.681 | 0.010 | cis |
| SNX1 |  |  |  |  |  |  |  |  |  |  |
| rs1802376 | Diabetic polyneuropathy | 15 | 64428559 | A/G | 0.026 | -0.023 | 0.239 | 0.923 | 0.026 | cis |
| LUM |  |  |  |  |  |  |  |  |  |  |
| rs1803343 | Diabetic polyneuropathy | 12 | 91539784 | T/C | 0.991 | 0.029 | 0.409 | 0.943 | 0.009 | cis |
| MRC1 |  |  |  |  |  |  |  |  |  |  |
| rs181242111 | Diabetic polyneuropathy | 10 | 17865664 | A/G | 0.088 | 0.206 | 0.134 | 0.126 | 0.088 | cis |
| DEFB104A_DEFB104B |  |  |  |  |  |  |  |  |  |  |
| rs183772362 | Diabetic polyneuropathy | 8 | 7243016 | T/C | 0.010 | -0.372 | 0.377 | 0.324 | 0.010 | cis |
| RILP |  |  |  |  |  |  |  |  |  |  |
| rs183827902 | Diabetic polyneuropathy | 17 | 1552519 | T/C | 0.009 | 0.458 | 0.411 | 0.265 | 0.009 | cis |
| CHAD |  |  |  |  |  |  |  |  |  |  |
| rs184613584 | Diabetic polyneuropathy | 17 | 48508221 | A/C | 0.990 | 0.504 | 0.365 | 0.167 | 0.010 | cis |
| EPHB1 |  |  |  |  |  |  |  |  |  |  |
| rs185257 | Diabetic polyneuropathy | 3 | 134672285 | A/C | 0.404 | -0.052 | 0.077 | 0.498 | 0.404 | cis |
| PROK1 |  |  |  |  |  |  |  |  |  |  |
| rs1857512 | Diabetic polyneuropathy | 1 | 110993349 | A/G | 0.145 | 0.004 | 0.108 | 0.972 | 0.145 | cis |
| PILRB |  |  |  |  |  |  |  |  |  |  |
| rs1859788 | Diabetic polyneuropathy | 7 | 99971834 | G/A | 0.691 | -0.006 | 0.082 | 0.944 | 0.309 | cis |
| PGLYRP3 |  |  |  |  |  |  |  |  |  |  |
| rs1865234 | Diabetic polyneuropathy | 1 | 153315784 | T/C | 0.088 | -0.016 | 0.133 | 0.904 | 0.088 | cis |
| GPNMB |  |  |  |  |  |  |  |  |  |  |
| rs2268748 | Diabetic polyneuropathy | 7 | 23313171 | T/C | 0.917 | 0.033 | 0.138 | 0.810 | 0.083 | cis |
| rs1881203 | Diabetic polyneuropathy | 7 | 23292808 | T/C | 0.711 | -0.073 | 0.083 | 0.378 | 0.289 | cis |
| CXCL2_CXCL3 |  |  |  |  |  |  |  |  |  |  |
| rs1893319 | Diabetic polyneuropathy | 4 | 74973129 | T/C | 0.339 | 0.090 | 0.080 | 0.258 | 0.339 | cis |
| FES |  |  |  |  |  |  |  |  |  |  |
| rs1894401 | Diabetic polyneuropathy | 15 | 91429042 | A/G | 0.596 | -0.032 | 0.077 | 0.681 | 0.405 | cis |
| MCL1 |  |  |  |  |  |  |  |  |  |  |
| rs190596489 | Diabetic polyneuropathy | 1 | 150562043 | A/C | 0.993 | -0.477 | 0.432 | 0.270 | 0.007 | cis |
| SMAP1 |  |  |  |  |  |  |  |  |  |  |
| rs1917342 | Diabetic polyneuropathy | 6 | 71349754 | A/G | 0.669 | 0.031 | 0.081 | 0.704 | 0.331 | cis |
| UROS |  |  |  |  |  |  |  |  |  |  |
| rs1935451 | Diabetic polyneuropathy | 10 | 127505426 | G/A | 0.091 | 0.072 | 0.133 | 0.588 | 0.091 | cis |
| OLFM3 |  |  |  |  |  |  |  |  |  |  |
| rs1938364 | Diabetic polyneuropathy | 1 | 102667388 | A/G | 0.138 | 0.038 | 0.108 | 0.724 | 0.138 | cis |
| SMOC1 |  |  |  |  |  |  |  |  |  |  |
| rs1958078 | Diabetic polyneuropathy | 14 | 70354858 | C/A | 0.919 | 0.033 | 0.139 | 0.813 | 0.081 | cis |
| SFTPA1 |  |  |  |  |  |  |  |  |  |  |
| rs1965708 | Diabetic polyneuropathy | 10 | 81317045 | T/G | 0.215 | 0.076 | 0.092 | 0.409 | 0.215 | cis |
| IMPA1 |  |  |  |  |  |  |  |  |  |  |
| rs1967328 | Diabetic polyneuropathy | 8 | 82583771 | T/G | 0.402 | 0.137 | 0.077 | 0.077 | 0.402 | cis |
| ANGPT2 |  |  |  |  |  |  |  |  |  |  |
| rs1968586 | Diabetic polyneuropathy | 8 | 6279511 | T/C | 0.380 | 0.035 | 0.078 | 0.655 | 0.380 | cis |
| SPON1 |  |  |  |  |  |  |  |  |  |  |
| rs1969539 | Diabetic polyneuropathy | 11 | 14038621 | A/G | 0.412 | -0.014 | 0.076 | 0.856 | 0.412 | cis |
| CDH5 |  |  |  |  |  |  |  |  |  |  |
| rs1972838 | Diabetic polyneuropathy | 16 | 66428226 | A/C | 0.322 | 0.025 | 0.081 | 0.755 | 0.322 | cis |
| NPPB |  |  |  |  |  |  |  |  |  |  |
| rs198389 | Diabetic polyneuropathy | 1 | 11919271 | G/A | 0.364 | -0.101 | 0.079 | 0.200 | 0.364 | cis |
| NTproBNP |  |  |  |  |  |  |  |  |  |  |
| rs198389 | Diabetic polyneuropathy | 1 | 11919271 | G/A | 0.364 | -0.101 | 0.079 | 0.200 | 0.364 | cis |
| B3GALTL |  |  |  |  |  |  |  |  |  |  |
| rs1995970 | Diabetic polyneuropathy | 13 | 31874303 | T/C | 0.716 | -0.028 | 0.084 | 0.741 | 0.285 | cis |
| GGT1 |  |  |  |  |  |  |  |  |  |  |
| rs2006227 | Diabetic polyneuropathy | 22 | 24995756 | A/C | 0.331 | -0.042 | 0.080 | 0.603 | 0.331 | cis |
| B4GALT6 |  |  |  |  |  |  |  |  |  |  |
| rs201022770 | Diabetic polyneuropathy | 18 | 29213359 | CT/C | 0.021 | 0.229 | 0.259 | 0.376 | 0.021 | cis |
| WISP1 |  |  |  |  |  |  |  |  |  |  |
| rs2013158 | Diabetic polyneuropathy | 8 | 134202942 | A/C | 0.143 | 0.030 | 0.108 | 0.783 | 0.143 | cis |
| CDON |  |  |  |  |  |  |  |  |  |  |
| rs201453301 | Diabetic polyneuropathy | 11 | 125889893 | G/GC | 0.123 | 0.194 | 0.116 | 0.095 | 0.123 | cis |
| CCL18 |  |  |  |  |  |  |  |  |  |  |
| rs2015086 | Diabetic polyneuropathy | 17 | 34391617 | A/G | 0.846 | 0.100 | 0.105 | 0.342 | 0.154 | cis |
| CCL3 |  |  |  |  |  |  |  |  |  |  |
| rs2015086 | Diabetic polyneuropathy | 17 | 34391617 | A/G | 0.846 | 0.100 | 0.105 | 0.342 | 0.154 | cis |
| SEMA3G |  |  |  |  |  |  |  |  |  |  |
| rs2016575 | Diabetic polyneuropathy | 3 | 52477080 | T/C | 0.098 | -0.046 | 0.125 | 0.712 | 0.098 | cis |
| APOF |  |  |  |  |  |  |  |  |  |  |
| rs2020854 | Diabetic polyneuropathy | 12 | 56743367 | C/T | 0.055 | 0.381 | 0.165 | 0.021 | 0.055 | cis |
| PLAT |  |  |  |  |  |  |  |  |  |  |
| rs2020921 | Diabetic polyneuropathy | 8 | 42044965 | A/G | 0.008 | -0.527 | 0.414 | 0.203 | 0.008 | cis |
| CCL24 |  |  |  |  |  |  |  |  |  |  |
| rs2024050 | Diabetic polyneuropathy | 7 | 75460393 | G/A | 0.928 | -0.340 | 0.146 | 0.020 | 0.073 | cis |
| CCL25 |  |  |  |  |  |  |  |  |  |  |
| rs2032887 | Diabetic polyneuropathy | 19 | 8121360 | G/A | 0.275 | 0.137 | 0.085 | 0.105 | 0.275 | cis |
| GZMA |  |  |  |  |  |  |  |  |  |  |
| rs2047745 | Diabetic polyneuropathy | 5 | 54489541 | C/T | 0.202 | 0.088 | 0.095 | 0.352 | 0.202 | cis |
| RTN4IP1 |  |  |  |  |  |  |  |  |  |  |
| rs2054365 | Diabetic polyneuropathy | 6 | 107077502 | A/G | 0.381 | -0.007 | 0.078 | 0.928 | 0.381 | cis |
| IL18R1 |  |  |  |  |  |  |  |  |  |  |
| rs2058622 | Diabetic polyneuropathy | 2 | 102985424 | A/G | 0.191 | -0.023 | 0.096 | 0.813 | 0.191 | cis |
| TNFSF11 |  |  |  |  |  |  |  |  |  |  |
| rs2062305 | Diabetic polyneuropathy | 13 | 43052880 | A/G | 0.614 | -0.062 | 0.077 | 0.422 | 0.386 | cis |
| PMEL |  |  |  |  |  |  |  |  |  |  |
| rs2069398 | Diabetic polyneuropathy | 12 | 56360876 | A/G | 0.075 | -0.284 | 0.145 | 0.051 | 0.075 | cis |
| CCL27 |  |  |  |  |  |  |  |  |  |  |
| rs2070074 | Diabetic polyneuropathy | 9 | 34649442 | G/A | 0.111 | -0.174 | 0.120 | 0.146 | 0.111 | cis |
| RBP1 |  |  |  |  |  |  |  |  |  |  |
| rs2071387 | Diabetic polyneuropathy | 3 | 139257603 | G/A | 0.190 | -0.051 | 0.096 | 0.592 | 0.190 | cis |
| PGLYRP1 |  |  |  |  |  |  |  |  |  |  |
| rs2072563 | Diabetic polyneuropathy | 19 | 46526648 | A/G | 0.228 | -0.025 | 0.090 | 0.784 | 0.228 | cis |
| FGL1 |  |  |  |  |  |  |  |  |  |  |
| rs2073562 | Diabetic polyneuropathy | 8 | 17742929 | A/G | 0.312 | -0.096 | 0.082 | 0.240 | 0.312 | cis |
| UCMA |  |  |  |  |  |  |  |  |  |  |
| rs2093847 | Diabetic polyneuropathy | 10 | 13276534 | T/C | 0.036 | 0.210 | 0.199 | 0.292 | 0.036 | cis |
| CCL5 |  |  |  |  |  |  |  |  |  |  |
| rs2107538 | Diabetic polyneuropathy | 17 | 34207780 | T/C | 0.173 | 0.197 | 0.100 | 0.048 | 0.173 | cis |
| GALNT3 |  |  |  |  |  |  |  |  |  |  |
| rs2116546 | Diabetic polyneuropathy | 2 | 166723533 | C/T | 0.276 | 0.073 | 0.084 | 0.389 | 0.276 | cis |
| SIGLEC6 |  |  |  |  |  |  |  |  |  |  |
| rs2124910 | Diabetic polyneuropathy | 19 | 52025247 | T/C | 0.403 | 0.021 | 0.077 | 0.782 | 0.403 | cis |
| DLK2 |  |  |  |  |  |  |  |  |  |  |
| rs2125739 | Diabetic polyneuropathy | 6 | 43412865 | C/T | 0.177 | -0.001 | 0.098 | 0.996 | 0.177 | cis |
| ADAMTS8 |  |  |  |  |  |  |  |  |  |  |
| rs2131535 | Diabetic polyneuropathy | 11 | 130281488 | A/G | 0.742 | 0.083 | 0.087 | 0.337 | 0.258 | cis |
| GPC5 |  |  |  |  |  |  |  |  |  |  |
| rs342706 | Diabetic polyneuropathy | 13 | 92417058 | C/T | 0.312 | 0.072 | 0.082 | 0.381 | 0.312 | cis |
| rs2147190 | Diabetic polyneuropathy | 13 | 92058888 | T/C | 0.403 | 0.044 | 0.077 | 0.570 | 0.403 | cis |
| ACP6 |  |  |  |  |  |  |  |  |  |  |
| rs2153463 | Diabetic polyneuropathy | 1 | 147124310 | G/T | 0.732 | -0.029 | 0.085 | 0.731 | 0.268 | cis |
| CPXM1 |  |  |  |  |  |  |  |  |  |  |
| rs215545 | Diabetic polyneuropathy | 20 | 2782015 | C/T | 0.750 | -0.018 | 0.087 | 0.835 | 0.250 | cis |
| HP |  |  |  |  |  |  |  |  |  |  |
| rs217181 | Diabetic polyneuropathy | 16 | 72114002 | T/C | 0.214 | 0.114 | 0.092 | 0.216 | 0.214 | cis |
| KIT |  |  |  |  |  |  |  |  |  |  |
| rs218263 | Diabetic polyneuropathy | 4 | 55408104 | T/C | 0.149 | -0.092 | 0.107 | 0.392 | 0.149 | cis |
| STX16 |  |  |  |  |  |  |  |  |  |  |
| rs218476 | Diabetic polyneuropathy | 20 | 57237670 | A/G | 0.392 | -0.032 | 0.078 | 0.682 | 0.392 | cis |
| GLYAT |  |  |  |  |  |  |  |  |  |  |
| rs2186414 | Diabetic polyneuropathy | 11 | 58530764 | A/C | 0.300 | 0.087 | 0.084 | 0.298 | 0.300 | cis |
| SELE |  |  |  |  |  |  |  |  |  |  |
| rs2205850 | Diabetic polyneuropathy | 1 | 169691439 | A/G | 0.674 | -0.055 | 0.081 | 0.497 | 0.326 | cis |
| FGA_FGB_FGG |  |  |  |  |  |  |  |  |  |  |
| rs2227426 | Diabetic polyneuropathy | 4 | 155493171 | A/G | 0.173 | 0.077 | 0.099 | 0.437 | 0.173 | cis |
| SERPINE1 |  |  |  |  |  |  |  |  |  |  |
| rs2227674 | Diabetic polyneuropathy | 7 | 100776208 | G/A | 0.179 | 0.226 | 0.098 | 0.022 | 0.179 | cis |
| ANXA4 |  |  |  |  |  |  |  |  |  |  |
| rs2228203 | Diabetic polyneuropathy | 2 | 70033584 | T/C | 0.261 | 0.024 | 0.086 | 0.777 | 0.261 | cis |
| NCAN |  |  |  |  |  |  |  |  |  |  |
| rs2228603 | Diabetic polyneuropathy | 19 | 19329924 | T/C | 0.070 | -0.054 | 0.149 | 0.718 | 0.070 | cis |
| ECH1 |  |  |  |  |  |  |  |  |  |  |
| rs2229259 | Diabetic polyneuropathy | 19 | 39307103 | T/C | 0.096 | -0.125 | 0.127 | 0.323 | 0.096 | cis |
| PRCP |  |  |  |  |  |  |  |  |  |  |
| rs2229437 | Diabetic polyneuropathy | 11 | 82564294 | T/G | 0.848 | 0.030 | 0.105 | 0.777 | 0.152 | cis |
| NT5E |  |  |  |  |  |  |  |  |  |  |
| rs2229523 | Diabetic polyneuropathy | 6 | 86199233 | G/A | 0.632 | -0.134 | 0.078 | 0.086 | 0.368 | cis |
| ACAA1 |  |  |  |  |  |  |  |  |  |  |
| rs2229528 | Diabetic polyneuropathy | 3 | 38167095 | G/A | 0.028 | 0.323 | 0.225 | 0.151 | 0.028 | cis |
| AKR1B1 |  |  |  |  |  |  |  |  |  |  |
| rs2229542 | Diabetic polyneuropathy | 7 | 134135621 | C/T | 0.013 | -0.306 | 0.351 | 0.383 | 0.013 | cis |
| CECR1 |  |  |  |  |  |  |  |  |  |  |
| rs2231495 | Diabetic polyneuropathy | 22 | 17669306 | C/T | 0.313 | 0.005 | 0.082 | 0.948 | 0.313 | cis |
| ADA2 |  |  |  |  |  |  |  |  |  |  |
| rs2231495 | Diabetic polyneuropathy | 22 | 17669306 | C/T | 0.313 | 0.005 | 0.082 | 0.948 | 0.313 | cis |
| TBCB |  |  |  |  |  |  |  |  |  |  |
| rs2231569 | Diabetic polyneuropathy | 19 | 36605875 | T/G | 0.038 | 0.244 | 0.198 | 0.217 | 0.038 | cis |
| LBP |  |  |  |  |  |  |  |  |  |  |
| rs2232613 | Diabetic polyneuropathy | 20 | 36997655 | T/C | 0.137 | 0.105 | 0.110 | 0.339 | 0.137 | cis |
| TBCC |  |  |  |  |  |  |  |  |  |  |
| rs2234026 | Diabetic polyneuropathy | 6 | 42713618 | G/A | 0.977 | 0.114 | 0.256 | 0.655 | 0.023 | cis |
| IL22RA2 |  |  |  |  |  |  |  |  |  |  |
| rs2234711 | Diabetic polyneuropathy | 6 | 137540520 | A/G | 0.640 | -0.071 | 0.079 | 0.369 | 0.360 | cis |
| CABLES2 |  |  |  |  |  |  |  |  |  |  |
| rs2236201 | Diabetic polyneuropathy | 20 | 60985627 | T/C | 0.675 | 0.083 | 0.081 | 0.306 | 0.325 | cis |
| PSME2 |  |  |  |  |  |  |  |  |  |  |
| rs2236352 | Diabetic polyneuropathy | 14 | 24610733 | C/T | 0.309 | -0.071 | 0.083 | 0.389 | 0.309 | cis |
| PON1 |  |  |  |  |  |  |  |  |  |  |
| rs2237582 | Diabetic polyneuropathy | 7 | 94934200 | G/A | 0.264 | -0.096 | 0.086 | 0.265 | 0.264 | cis |
| CSF2RB |  |  |  |  |  |  |  |  |  |  |
| rs2239749 | Diabetic polyneuropathy | 22 | 37331305 | G/A | 0.584 | -0.031 | 0.077 | 0.685 | 0.416 | cis |
| PADI4 |  |  |  |  |  |  |  |  |  |  |
| rs2240336 | Diabetic polyneuropathy | 1 | 17674402 | T/C | 0.398 | 0.054 | 0.077 | 0.486 | 0.398 | cis |
| LILRA4 |  |  |  |  |  |  |  |  |  |  |
| rs2241384 | Diabetic polyneuropathy | 19 | 54849942 | A/G | 0.165 | 0.141 | 0.103 | 0.169 | 0.165 | cis |
| SNCA |  |  |  |  |  |  |  |  |  |  |
| rs2245801 | Diabetic polyneuropathy | 4 | 90757840 | C/T | 0.833 | 0.001 | 0.101 | 0.994 | 0.167 | cis |
| C1QTNF5 |  |  |  |  |  |  |  |  |  |  |
| rs2248863 | Diabetic polyneuropathy | 11 | 119207341 | A/G | 0.179 | -0.023 | 0.099 | 0.816 | 0.179 | cis |
| TFF2 |  |  |  |  |  |  |  |  |  |  |
| rs225344 | Diabetic polyneuropathy | 21 | 43775884 | A/G | 0.665 | 0.140 | 0.080 | 0.082 | 0.335 | cis |
| KLRK1 |  |  |  |  |  |  |  |  |  |  |
| rs2255336 | Diabetic polyneuropathy | 12 | 10532326 | T/C | 0.190 | -0.054 | 0.097 | 0.577 | 0.190 | cis |
| PLXND1 |  |  |  |  |  |  |  |  |  |  |
| rs2255703 | Diabetic polyneuropathy | 3 | 129293256 | T/C | 0.670 | 0.096 | 0.081 | 0.232 | 0.330 | cis |
| A2M |  |  |  |  |  |  |  |  |  |  |
| rs226384 | Diabetic polyneuropathy | 12 | 9263647 | T/C | 0.409 | 0.075 | 0.077 | 0.331 | 0.409 | cis |
| THG1L |  |  |  |  |  |  |  |  |  |  |
| rs2270812 | Diabetic polyneuropathy | 5 | 157164962 | T/C | 0.336 | 0.021 | 0.080 | 0.795 | 0.336 | cis |
| CD300A |  |  |  |  |  |  |  |  |  |  |
| rs2272111 | Diabetic polyneuropathy | 17 | 72469966 | A/G | 0.202 | 0.093 | 0.094 | 0.324 | 0.202 | cis |
| CD59 |  |  |  |  |  |  |  |  |  |  |
| rs2273121 | Diabetic polyneuropathy | 11 | 33757770 | A/G | 0.261 | 0.003 | 0.086 | 0.972 | 0.261 | cis |
| VSTM2L |  |  |  |  |  |  |  |  |  |  |
| rs2273349 | Diabetic polyneuropathy | 20 | 36624756 | A/C | 0.158 | -0.193 | 0.104 | 0.063 | 0.158 | cis |
| MANBA |  |  |  |  |  |  |  |  |  |  |
| rs227370 | Diabetic polyneuropathy | 4 | 103612043 | C/T | 0.660 | -0.018 | 0.080 | 0.827 | 0.341 | cis |
| CST7 |  |  |  |  |  |  |  |  |  |  |
| rs227651 | Diabetic polyneuropathy | 20 | 24929834 | G/A | 0.103 | -0.185 | 0.122 | 0.127 | 0.103 | cis |
| LAMC2 |  |  |  |  |  |  |  |  |  |  |
| rs2276543 | Diabetic polyneuropathy | 1 | 183155305 | A/G | 0.358 | -0.030 | 0.079 | 0.710 | 0.358 | cis |
| DTX3 |  |  |  |  |  |  |  |  |  |  |
| rs2277323 | Diabetic polyneuropathy | 12 | 58009372 | A/G | 0.230 | 0.068 | 0.089 | 0.444 | 0.230 | cis |
| TNFAIP6 |  |  |  |  |  |  |  |  |  |  |
| rs2278089 | Diabetic polyneuropathy | 2 | 152146672 | G/T | 0.373 | -0.036 | 0.078 | 0.646 | 0.373 | cis |
| CCN3 |  |  |  |  |  |  |  |  |  |  |
| rs2279112 | Diabetic polyneuropathy | 8 | 120429024 | A/G | 0.203 | -0.121 | 0.094 | 0.196 | 0.203 | cis |
| GCNT1 |  |  |  |  |  |  |  |  |  |  |
| rs2282683 | Diabetic polyneuropathy | 9 | 79117751 | G/A | 0.159 | -0.023 | 0.103 | 0.820 | 0.159 | cis |
| CAT |  |  |  |  |  |  |  |  |  |  |
| rs2284367 | Diabetic polyneuropathy | 11 | 34484542 | C/T | 0.204 | 0.019 | 0.094 | 0.839 | 0.204 | cis |
| IGSF3 |  |  |  |  |  |  |  |  |  |  |
| rs2284860 | Diabetic polyneuropathy | 1 | 117122887 | C/T | 0.173 | 0.071 | 0.100 | 0.482 | 0.173 | cis |
| ITIH1 |  |  |  |  |  |  |  |  |  |  |
| rs2286798 | Diabetic polyneuropathy | 3 | 52821177 | A/C | 0.625 | 0.035 | 0.078 | 0.651 | 0.375 | cis |
| NRP2 |  |  |  |  |  |  |  |  |  |  |
| rs2289023 | Diabetic polyneuropathy | 2 | 205986321 | G/A | 0.285 | -0.087 | 0.083 | 0.294 | 0.285 | cis |
| ULK3 |  |  |  |  |  |  |  |  |  |  |
| rs2290574 | Diabetic polyneuropathy | 15 | 75135447 | T/C | 0.310 | 0.007 | 0.082 | 0.936 | 0.310 | cis |
| CD276 |  |  |  |  |  |  |  |  |  |  |
| rs2291014 | Diabetic polyneuropathy | 15 | 73996359 | A/G | 0.124 | 0.046 | 0.116 | 0.690 | 0.124 | cis |
| CPB1 |  |  |  |  |  |  |  |  |  |  |
| rs2291671 | Diabetic polyneuropathy | 3 | 148558447 | A/G | 0.226 | -0.042 | 0.091 | 0.645 | 0.226 | cis |
| TNFRSF1B |  |  |  |  |  |  |  |  |  |  |
| rs2301258 | Diabetic polyneuropathy | 1 | 12247940 | T/C | 0.015 | -0.388 | 0.304 | 0.202 | 0.015 | cis |
| PVR |  |  |  |  |  |  |  |  |  |  |
| rs2301274 | Diabetic polyneuropathy | 19 | 45146402 | C/T | 0.222 | 0.247 | 0.090 | 0.006 | 0.222 | cis |
| PLAUR |  |  |  |  |  |  |  |  |  |  |
| rs2302524 | Diabetic polyneuropathy | 19 | 44156472 | C/T | 0.109 | 0.108 | 0.121 | 0.375 | 0.109 | cis |
| DKKL1 |  |  |  |  |  |  |  |  |  |  |
| rs2303759 | Diabetic polyneuropathy | 19 | 49869051 | G/T | 0.171 | -0.137 | 0.100 | 0.170 | 0.171 | cis |
| DARS |  |  |  |  |  |  |  |  |  |  |
| rs2304371 | Diabetic polyneuropathy | 2 | 136561557 | A/G | 0.832 | 0.170 | 0.101 | 0.092 | 0.169 | cis |
| ACP5 |  |  |  |  |  |  |  |  |  |  |
| rs2305799 | Diabetic polyneuropathy | 19 | 11687351 | T/C | 0.145 | 0.141 | 0.108 | 0.193 | 0.145 | cis |
| ITGA11 |  |  |  |  |  |  |  |  |  |  |
| rs2306022 | Diabetic polyneuropathy | 15 | 68628163 | T/C | 0.088 | -0.105 | 0.132 | 0.426 | 0.088 | cis |
| LRP4 |  |  |  |  |  |  |  |  |  |  |
| rs2306029 | Diabetic polyneuropathy | 11 | 46893108 | T/C | 0.410 | -0.041 | 0.079 | 0.604 | 0.410 | cis |
| LRIG1 |  |  |  |  |  |  |  |  |  |  |
| rs2306272 | Diabetic polyneuropathy | 3 | 66434643 | C/T | 0.252 | -0.037 | 0.087 | 0.671 | 0.252 | cis |
| PDGFRA |  |  |  |  |  |  |  |  |  |  |
| rs2307050 | Diabetic polyneuropathy | 4 | 55141293 | A/G | 0.110 | -0.042 | 0.122 | 0.733 | 0.110 | cis |
| H6PD |  |  |  |  |  |  |  |  |  |  |
| rs2310925 | Diabetic polyneuropathy | 1 | 9306849 | T/C | 0.662 | 0.146 | 0.080 | 0.067 | 0.338 | cis |
| AIF1L |  |  |  |  |  |  |  |  |  |  |
| rs2315073 | Diabetic polyneuropathy | 9 | 133936775 | T/C | 0.735 | 0.053 | 0.086 | 0.534 | 0.266 | cis |
| CTSO |  |  |  |  |  |  |  |  |  |  |
| rs2334114 | Diabetic polyneuropathy | 4 | 156856193 | A/C | 0.173 | 0.063 | 0.099 | 0.524 | 0.173 | cis |
| ASPH |  |  |  |  |  |  |  |  |  |  |
| rs2350578 | Diabetic polyneuropathy | 8 | 62660578 | A/G | 0.621 | -0.045 | 0.078 | 0.568 | 0.379 | cis |
| PRDX1 |  |  |  |  |  |  |  |  |  |  |
| rs2356552 | Diabetic polyneuropathy | 1 | 46009316 | T/C | 0.063 | 0.109 | 0.154 | 0.478 | 0.063 | cis |
| CHGB |  |  |  |  |  |  |  |  |  |  |
| rs236153 | Diabetic polyneuropathy | 20 | 5903894 | G/A | 0.418 | 0.038 | 0.076 | 0.616 | 0.418 | cis |
| BCAN |  |  |  |  |  |  |  |  |  |  |
| rs2365715 | Diabetic polyneuropathy | 1 | 156615114 | G/A | 0.321 | -0.056 | 0.082 | 0.495 | 0.321 | cis |
| PF4V1 |  |  |  |  |  |  |  |  |  |  |
| rs2367288 | Diabetic polyneuropathy | 4 | 74714098 | A/G | 0.708 | -0.026 | 0.083 | 0.757 | 0.293 | cis |
| CRTAM |  |  |  |  |  |  |  |  |  |  |
| rs2370794 | Diabetic polyneuropathy | 11 | 122714782 | G/A | 0.407 | 0.017 | 0.077 | 0.829 | 0.407 | cis |
| ENO3 |  |  |  |  |  |  |  |  |  |  |
| rs238238 | Diabetic polyneuropathy | 17 | 4856376 | A/G | 0.368 | 0.200 | 0.079 | 0.011 | 0.368 | cis |
| CGREF1 |  |  |  |  |  |  |  |  |  |  |
| rs2384572 | Diabetic polyneuropathy | 2 | 27324751 | T/G | 0.648 | 0.044 | 0.079 | 0.579 | 0.352 | cis |
| FBLN5 |  |  |  |  |  |  |  |  |  |  |
| rs2402088 | Diabetic polyneuropathy | 14 | 92388714 | A/G | 0.399 | -0.091 | 0.077 | 0.239 | 0.399 | cis |
| PILRA |  |  |  |  |  |  |  |  |  |  |
| rs2405442 | Diabetic polyneuropathy | 7 | 99971313 | T/C | 0.306 | 0.011 | 0.082 | 0.898 | 0.306 | cis |
| IGFBP7 |  |  |  |  |  |  |  |  |  |  |
| rs2412776 | Diabetic polyneuropathy | 4 | 57951468 | G/A | 0.241 | -0.006 | 0.089 | 0.947 | 0.241 | cis |
| PIK3IP1 |  |  |  |  |  |  |  |  |  |  |
| rs2413028 | Diabetic polyneuropathy | 22 | 31541907 | C/T | 0.174 | 0.021 | 0.100 | 0.834 | 0.174 | cis |
| MGP |  |  |  |  |  |  |  |  |  |  |
| rs2430687 | Diabetic polyneuropathy | 12 | 15047396 | T/C | 0.690 | 0.106 | 0.082 | 0.193 | 0.310 | cis |
| ALKBH3 |  |  |  |  |  |  |  |  |  |  |
| rs2434484 | Diabetic polyneuropathy | 11 | 43949008 | T/G | 0.692 | 0.116 | 0.082 | 0.157 | 0.308 | cis |
| THBS4 |  |  |  |  |  |  |  |  |  |  |
| rs2438632 | Diabetic polyneuropathy | 5 | 79392193 | T/G | 0.322 | 0.035 | 0.081 | 0.662 | 0.322 | cis |
| CD33 |  |  |  |  |  |  |  |  |  |  |
| rs2455069 | Diabetic polyneuropathy | 19 | 51728641 | G/A | 0.388 | 0.081 | 0.077 | 0.293 | 0.388 | cis |
| HBZ |  |  |  |  |  |  |  |  |  |  |
| rs2461286 | Diabetic polyneuropathy | 16 | 203254 | G/A | 0.663 | 0.027 | 0.080 | 0.735 | 0.337 | cis |
| ELOA |  |  |  |  |  |  |  |  |  |  |
| rs2473378 | Diabetic polyneuropathy | 1 | 24050735 | A/G | 0.596 | 0.040 | 0.077 | 0.601 | 0.404 | cis |
| AGT |  |  |  |  |  |  |  |  |  |  |
| rs2493151 | Diabetic polyneuropathy | 1 | 230878561 | A/G | 0.210 | 0.034 | 0.093 | 0.719 | 0.210 | cis |
| HGFAC |  |  |  |  |  |  |  |  |  |  |
| rs2498323 | Diabetic polyneuropathy | 4 | 3451109 | A/G | 0.067 | -0.152 | 0.149 | 0.309 | 0.067 | cis |
| IPCEF1 |  |  |  |  |  |  |  |  |  |  |
| rs2499649 | Diabetic polyneuropathy | 6 | 154675915 | C/T | 0.227 | -0.205 | 0.091 | 0.024 | 0.227 | cis |
| NRP1 |  |  |  |  |  |  |  |  |  |  |
| rs2506150 | Diabetic polyneuropathy | 10 | 33483308 | A/G | 0.363 | 0.045 | 0.078 | 0.563 | 0.363 | cis |
| HYOU1 |  |  |  |  |  |  |  |  |  |  |
| rs2509121 | Diabetic polyneuropathy | 11 | 118928253 | T/C | 0.293 | -0.061 | 0.083 | 0.458 | 0.293 | cis |
| NTM |  |  |  |  |  |  |  |  |  |  |
| rs2511504 | Diabetic polyneuropathy | 11 | 131196396 | C/T | 0.372 | -0.041 | 0.078 | 0.600 | 0.372 | cis |
| NSF |  |  |  |  |  |  |  |  |  |  |
| rs2532240 | Diabetic polyneuropathy | 17 | 44265839 | T/C | 0.375 | -0.031 | 0.078 | 0.694 | 0.375 | cis |
| ITIH3 |  |  |  |  |  |  |  |  |  |  |
| rs2535629 | Diabetic polyneuropathy | 3 | 52833219 | A/G | 0.360 | -0.041 | 0.079 | 0.598 | 0.360 | cis |
| CD302 |  |  |  |  |  |  |  |  |  |  |
| rs2556106 | Diabetic polyneuropathy | 2 | 160654524 | T/G | 0.908 | -0.094 | 0.131 | 0.475 | 0.092 | cis |
| ACAT2 |  |  |  |  |  |  |  |  |  |  |
| rs25683 | Diabetic polyneuropathy | 6 | 160196343 | A/G | 0.415 | -0.080 | 0.077 | 0.300 | 0.415 | cis |
| KLK14 |  |  |  |  |  |  |  |  |  |  |
| rs2569491 | Diabetic polyneuropathy | 19 | 51584916 | A/G | 0.233 | 0.046 | 0.089 | 0.607 | 0.233 | cis |
| LY6D |  |  |  |  |  |  |  |  |  |  |
| rs2572925 | Diabetic polyneuropathy | 8 | 143867905 | T/C | 0.369 | -0.020 | 0.079 | 0.799 | 0.369 | cis |
| ITGAV |  |  |  |  |  |  |  |  |  |  |
| rs2595391 | Diabetic polyneuropathy | 2 | 187532373 | G/T | 0.070 | -0.048 | 0.146 | 0.743 | 0.070 | cis |
| SFRP4 |  |  |  |  |  |  |  |  |  |  |
| rs2598105 | Diabetic polyneuropathy | 7 | 37977116 | T/C | 0.157 | 0.150 | 0.104 | 0.150 | 0.157 | cis |
| MIA |  |  |  |  |  |  |  |  |  |  |
| rs2604877 | Diabetic polyneuropathy | 19 | 41275048 | C/T | 0.125 | 0.128 | 0.115 | 0.265 | 0.125 | cis |
| COL6A3 |  |  |  |  |  |  |  |  |  |  |
| rs2646260 | Diabetic polyneuropathy | 2 | 238277795 | A/G | 0.781 | 0.022 | 0.092 | 0.814 | 0.219 | cis |
| KLK4 |  |  |  |  |  |  |  |  |  |  |
| rs2664153 | Diabetic polyneuropathy | 19 | 51415252 | A/G | 0.657 | -0.043 | 0.080 | 0.589 | 0.343 | cis |
| DSG2 |  |  |  |  |  |  |  |  |  |  |
| rs2704053 | Diabetic polyneuropathy | 18 | 29095089 | G/A | 0.416 | 0.066 | 0.077 | 0.391 | 0.416 | cis |
| CKAP4 |  |  |  |  |  |  |  |  |  |  |
| rs2704895 | Diabetic polyneuropathy | 12 | 106649988 | A/G | 0.263 | -0.056 | 0.086 | 0.512 | 0.263 | cis |
| PCYOX1 |  |  |  |  |  |  |  |  |  |  |
| rs2706762 | Diabetic polyneuropathy | 2 | 70488470 | T/C | 0.073 | -0.113 | 0.143 | 0.430 | 0.073 | cis |
| RPN1 |  |  |  |  |  |  |  |  |  |  |
| rs2712417 | Diabetic polyneuropathy | 3 | 128345179 | G/A | 0.615 | -0.059 | 0.077 | 0.442 | 0.385 | cis |
| SCARA5 |  |  |  |  |  |  |  |  |  |  |
| rs2726951 | Diabetic polyneuropathy | 8 | 27805783 | T/C | 0.287 | -0.009 | 0.083 | 0.911 | 0.287 | cis |
| CXADR |  |  |  |  |  |  |  |  |  |  |
| rs2739393 | Diabetic polyneuropathy | 21 | 18934539 | A/G | 0.675 | 0.014 | 0.081 | 0.868 | 0.326 | cis |
| DEFB1 |  |  |  |  |  |  |  |  |  |  |
| rs2741117 | Diabetic polyneuropathy | 8 | 6725969 | T/G | 0.411 | -0.100 | 0.077 | 0.194 | 0.411 | cis |
| SCUBE1 |  |  |  |  |  |  |  |  |  |  |
| rs2744874 | Diabetic polyneuropathy | 22 | 43715862 | T/C | 0.835 | -0.177 | 0.103 | 0.085 | 0.165 | cis |
| ARG1 |  |  |  |  |  |  |  |  |  |  |
| rs2781668 | Diabetic polyneuropathy | 6 | 131897278 | T/C | 0.231 | 0.079 | 0.090 | 0.382 | 0.231 | cis |
| RET |  |  |  |  |  |  |  |  |  |  |
| rs2795507 | Diabetic polyneuropathy | 10 | 43352894 | C/T | 0.802 | -0.213 | 0.095 | 0.025 | 0.198 | cis |
| ICAM5 |  |  |  |  |  |  |  |  |  |  |
| rs281440 | Diabetic polyneuropathy | 19 | 10400304 | A/G | 0.767 | -0.041 | 0.089 | 0.649 | 0.233 | cis |
| FKBP5 |  |  |  |  |  |  |  |  |  |  |
| rs2817032 | Diabetic polyneuropathy | 6 | 35688619 | C/T | 0.267 | 0.052 | 0.086 | 0.543 | 0.267 | cis |
| NCAM2 |  |  |  |  |  |  |  |  |  |  |
| rs2826851 | Diabetic polyneuropathy | 21 | 22835946 | G/A | 0.263 | -0.090 | 0.087 | 0.296 | 0.263 | cis |
| ADAMTS5 |  |  |  |  |  |  |  |  |  |  |
| rs2830585 | Diabetic polyneuropathy | 21 | 28305212 | T/C | 0.141 | -0.169 | 0.110 | 0.123 | 0.141 | cis |
| ADH1A |  |  |  |  |  |  |  |  |  |  |
| rs28364331 | Diabetic polyneuropathy | 4 | 100201295 | A/G | 0.997 | 0.453 | 0.684 | 0.508 | 0.003 | cis |
| ICAM3 |  |  |  |  |  |  |  |  |  |  |
| rs28378712 | Diabetic polyneuropathy | 19 | 10457472 | G/T | 0.321 | 0.024 | 0.081 | 0.772 | 0.321 | cis |
| SMPDL3A |  |  |  |  |  |  |  |  |  |  |
| rs28385609 | Diabetic polyneuropathy | 6 | 123122464 | T/C | 0.180 | -0.009 | 0.099 | 0.925 | 0.180 | cis |
| BCAM |  |  |  |  |  |  |  |  |  |  |
| rs28399654 | Diabetic polyneuropathy | 19 | 45316588 | A/G | 0.015 | 0.359 | 0.306 | 0.242 | 0.015 | cis |
| COCH |  |  |  |  |  |  |  |  |  |  |
| rs28400019 | Diabetic polyneuropathy | 14 | 31343494 | A/G | 0.107 | 0.023 | 0.124 | 0.855 | 0.107 | cis |
| PTPRF |  |  |  |  |  |  |  |  |  |  |
| rs2842194 | Diabetic polyneuropathy | 1 | 44028962 | A/G | 0.186 | 0.061 | 0.097 | 0.529 | 0.186 | cis |
| FABP9 |  |  |  |  |  |  |  |  |  |  |
| rs28497692 | Diabetic polyneuropathy | 8 | 82412691 | T/G | 0.032 | 0.113 | 0.220 | 0.606 | 0.032 | cis |
| BTC |  |  |  |  |  |  |  |  |  |  |
| rs28549760 | Diabetic polyneuropathy | 4 | 75719517 | C/A | 0.138 | 0.200 | 0.110 | 0.070 | 0.138 | cis |
| SCARB2 |  |  |  |  |  |  |  |  |  |  |
| rs28563976 | Diabetic polyneuropathy | 4 | 77097373 | C/A | 0.136 | 0.070 | 0.110 | 0.524 | 0.136 | cis |
| HPGDS |  |  |  |  |  |  |  |  |  |  |
| rs2865353 | Diabetic polyneuropathy | 4 | 95270878 | C/T | 0.639 | -0.009 | 0.079 | 0.909 | 0.361 | cis |
| IFNL1 |  |  |  |  |  |  |  |  |  |  |
| rs28668750 | Diabetic polyneuropathy | 19 | 39819802 | T/G | 0.110 | 0.115 | 0.121 | 0.345 | 0.110 | cis |
| NUDT9 |  |  |  |  |  |  |  |  |  |  |
| rs28696943 | Diabetic polyneuropathy | 4 | 88310135 | G/A | 0.133 | 0.089 | 0.111 | 0.422 | 0.133 | cis |
| CD38 |  |  |  |  |  |  |  |  |  |  |
| rs28703311 | Diabetic polyneuropathy | 4 | 15775851 | A/G | 0.209 | -0.050 | 0.093 | 0.592 | 0.209 | cis |
| QDPR |  |  |  |  |  |  |  |  |  |  |
| rs28719835 | Diabetic polyneuropathy | 4 | 17520066 | T/C | 0.214 | -0.088 | 0.092 | 0.340 | 0.214 | cis |
| FRZB |  |  |  |  |  |  |  |  |  |  |
| rs288326 | Diabetic polyneuropathy | 2 | 183703336 | A/G | 0.061 | 0.200 | 0.157 | 0.204 | 0.061 | cis |
| SERPINA1 |  |  |  |  |  |  |  |  |  |  |
| rs28929474 | Diabetic polyneuropathy | 14 | 94844947 | T/C | 0.020 | -0.002 | 0.272 | 0.993 | 0.020 | cis |
| PIGR |  |  |  |  |  |  |  |  |  |  |
| rs291102 | Diabetic polyneuropathy | 1 | 207106478 | A/G | 0.006 | 0.002 | 0.463 | 0.997 | 0.006 | cis |
| HIBCH |  |  |  |  |  |  |  |  |  |  |
| rs291466 | Diabetic polyneuropathy | 2 | 191184475 | A/G | 0.383 | 0.079 | 0.078 | 0.309 | 0.383 | cis |
| ATOX1 |  |  |  |  |  |  |  |  |  |  |
| rs2915894 | Diabetic polyneuropathy | 5 | 151195933 | T/C | 0.415 | 0.115 | 0.077 | 0.135 | 0.415 | cis |
| LRP12 |  |  |  |  |  |  |  |  |  |  |
| rs2942570 | Diabetic polyneuropathy | 8 | 105481705 | T/C | 0.369 | -0.007 | 0.078 | 0.925 | 0.369 | cis |
| RBP2 |  |  |  |  |  |  |  |  |  |  |
| rs295469 | Diabetic polyneuropathy | 3 | 139212900 | A/G | 0.269 | -0.049 | 0.085 | 0.563 | 0.269 | cis |
| FGFR2 |  |  |  |  |  |  |  |  |  |  |
| rs2981430 | Diabetic polyneuropathy | 10 | 123311698 | A/G | 0.585 | -0.130 | 0.077 | 0.091 | 0.415 | cis |
| ADAM8 |  |  |  |  |  |  |  |  |  |  |
| rs2995310 | Diabetic polyneuropathy | 10 | 135082810 | T/C | 0.908 | 0.128 | 0.129 | 0.322 | 0.092 | cis |
| CD8A |  |  |  |  |  |  |  |  |  |  |
| rs3020726 | Diabetic polyneuropathy | 2 | 87016506 | G/A | 0.096 | -0.084 | 0.125 | 0.501 | 0.096 | cis |
| IL10 |  |  |  |  |  |  |  |  |  |  |
| rs3024495 | Diabetic polyneuropathy | 1 | 206942413 | T/C | 0.157 | -0.082 | 0.104 | 0.428 | 0.157 | cis |
| RAB1A |  |  |  |  |  |  |  |  |  |  |
| rs3052195 | Diabetic polyneuropathy | 2 | 65313215 | C/CAG | 0.252 | 0.000 | 0.087 | 0.998 | 0.252 | cis |
| SCGB3A1 |  |  |  |  |  |  |  |  |  |  |
| rs307802 | Diabetic polyneuropathy | 5 | 180019237 | T/C | 0.659 | 0.090 | 0.080 | 0.260 | 0.341 | cis |
| LYPLAL1 |  |  |  |  |  |  |  |  |  |  |
| rs3084102 | Diabetic polyneuropathy | 1 | 219340555 | A/AGTACTC | 0.105 | -0.018 | 0.122 | 0.885 | 0.105 | cis |
| F7 |  |  |  |  |  |  |  |  |  |  |
| rs3093253 | Diabetic polyneuropathy | 13 | 113774092 | A/G | 0.077 | -0.092 | 0.141 | 0.516 | 0.077 | cis |
| BANK1 |  |  |  |  |  |  |  |  |  |  |
| rs3113676 | Diabetic polyneuropathy | 4 | 102965043 | C/T | 0.985 | 0.131 | 0.322 | 0.684 | 0.015 | cis |
| CXCL1 |  |  |  |  |  |  |  |  |  |  |
| rs3117600 | Diabetic polyneuropathy | 4 | 74731812 | G/A | 0.319 | -0.007 | 0.081 | 0.936 | 0.319 | cis |
| TIE1 |  |  |  |  |  |  |  |  |  |  |
| rs3120276 | Diabetic polyneuropathy | 1 | 43779564 | T/C | 0.334 | 0.114 | 0.080 | 0.156 | 0.334 | cis |
| FST |  |  |  |  |  |  |  |  |  |  |
| rs31226 | Diabetic polyneuropathy | 5 | 53327571 | T/C | 0.396 | -0.009 | 0.077 | 0.909 | 0.396 | cis |
| AGRN |  |  |  |  |  |  |  |  |  |  |
| rs3128125 | Diabetic polyneuropathy | 1 | 961464 | T/G | 0.380 | -0.027 | 0.079 | 0.732 | 0.380 | cis |
| CCL13 |  |  |  |  |  |  |  |  |  |  |
| rs3136674 | Diabetic polyneuropathy | 17 | 32682616 | C/T | 0.054 | -0.145 | 0.168 | 0.388 | 0.054 | cis |
| CCL7 |  |  |  |  |  |  |  |  |  |  |
| rs3138036 | Diabetic polyneuropathy | 17 | 32647544 | G/A | 0.152 | 0.072 | 0.105 | 0.494 | 0.152 | cis |
| TPST1 |  |  |  |  |  |  |  |  |  |  |
| rs313829 | Diabetic polyneuropathy | 7 | 65552497 | G/A | 0.614 | -0.015 | 0.078 | 0.851 | 0.386 | cis |
| CROT |  |  |  |  |  |  |  |  |  |  |
| rs31653 | Diabetic polyneuropathy | 7 | 87032613 | A/G | 0.131 | -0.067 | 0.112 | 0.551 | 0.131 | cis |
| ITPKA |  |  |  |  |  |  |  |  |  |  |
| rs316617 | Diabetic polyneuropathy | 15 | 41795900 | T/C | 0.612 | -0.006 | 0.078 | 0.938 | 0.388 | cis |
| CD7 |  |  |  |  |  |  |  |  |  |  |
| rs3176831 | Diabetic polyneuropathy | 17 | 80272491 | T/C | 0.039 | -0.170 | 0.195 | 0.383 | 0.039 | cis |
| LARGE |  |  |  |  |  |  |  |  |  |  |
| rs3210587 | Diabetic polyneuropathy | 22 | 34316077 | A/G | 0.341 | 0.078 | 0.080 | 0.326 | 0.341 | cis |
| PODXL |  |  |  |  |  |  |  |  |  |  |
| rs3212298 | Diabetic polyneuropathy | 7 | 131193739 | T/C | 0.046 | 0.360 | 0.179 | 0.044 | 0.046 | cis |
| POLI |  |  |  |  |  |  |  |  |  |  |
| rs3218784 | Diabetic polyneuropathy | 18 | 51807260 | A/G | 0.979 | -0.084 | 0.267 | 0.753 | 0.021 | cis |
| AARSD1 |  |  |  |  |  |  |  |  |  |  |
| rs323500 | Diabetic polyneuropathy | 17 | 41140545 | T/C | 0.349 | 0.072 | 0.080 | 0.370 | 0.349 | cis |
| PRDX6 |  |  |  |  |  |  |  |  |  |  |
| rs33951697 | Diabetic polyneuropathy | 1 | 173458326 | T/C | 0.142 | 0.027 | 0.108 | 0.801 | 0.142 | cis |
| ENPEP |  |  |  |  |  |  |  |  |  |  |
| rs33966350 | Diabetic polyneuropathy | 4 | 111431444 | A/G | 0.010 | -0.104 | 0.359 | 0.773 | 0.010 | cis |
| AOC3 |  |  |  |  |  |  |  |  |  |  |
| rs33986943 | Diabetic polyneuropathy | 17 | 41004637 | A/G | 0.071 | 0.157 | 0.145 | 0.278 | 0.071 | cis |
| CHRD |  |  |  |  |  |  |  |  |  |  |
| rs34095724 | Diabetic polyneuropathy | 3 | 184099050 | T/C | 0.013 | 0.076 | 0.324 | 0.814 | 0.013 | cis |
| C4BPA |  |  |  |  |  |  |  |  |  |  |
| rs34101855 | Diabetic polyneuropathy | 1 | 207281706 | A/AG | 0.900 | -0.072 | 0.126 | 0.569 | 0.100 | cis |
| NOV |  |  |  |  |  |  |  |  |  |  |
| rs34112166 | Diabetic polyneuropathy | 8 | 120400532 | A/G | 0.171 | -0.139 | 0.100 | 0.164 | 0.171 | cis |
| CPVL |  |  |  |  |  |  |  |  |  |  |
| rs34219043 | Diabetic polyneuropathy | 7 | 29160604 | T/C | 0.128 | -0.015 | 0.112 | 0.893 | 0.128 | cis |
| SYK |  |  |  |  |  |  |  |  |  |  |
| rs34221447 | Diabetic polyneuropathy | 9 | 93563884 | T/C | 0.185 | 0.055 | 0.097 | 0.572 | 0.185 | cis |
| KDR |  |  |  |  |  |  |  |  |  |  |
| rs34231037 | Diabetic polyneuropathy | 4 | 55972946 | G/A | 0.036 | 0.311 | 0.211 | 0.139 | 0.036 | cis |
| CELA3A |  |  |  |  |  |  |  |  |  |  |
| rs34262568 | Diabetic polyneuropathy | 1 | 22313371 | A/G | 0.231 | -0.047 | 0.090 | 0.598 | 0.231 | cis |
| ANXA2 |  |  |  |  |  |  |  |  |  |  |
| rs34293839 | Diabetic polyneuropathy | 15 | 60715248 | A/G | 0.895 | -0.008 | 0.122 | 0.945 | 0.105 | cis |
| GMPR2 |  |  |  |  |  |  |  |  |  |  |
| rs34354104 | Diabetic polyneuropathy | 14 | 24707479 | A/G | 0.047 | 0.176 | 0.177 | 0.320 | 0.047 | cis |
| TNFSF14 |  |  |  |  |  |  |  |  |  |  |
| rs344560 | Diabetic polyneuropathy | 19 | 6665020 | T/C | 0.057 | 0.347 | 0.162 | 0.032 | 0.057 | cis |
| GLTPD2 |  |  |  |  |  |  |  |  |  |  |
| rs34460487 | Diabetic polyneuropathy | 17 | 4685228 | A/G | 0.286 | -0.075 | 0.084 | 0.373 | 0.286 | cis |
| SLITRK5 |  |  |  |  |  |  |  |  |  |  |
| rs34509844 | Diabetic polyneuropathy | 13 | 88703595 | C/CT | 0.260 | 0.104 | 0.086 | 0.227 | 0.260 | cis |
| TCN1 |  |  |  |  |  |  |  |  |  |  |
| rs34528912 | Diabetic polyneuropathy | 11 | 59631535 | T/C | 0.054 | -0.260 | 0.172 | 0.131 | 0.054 | cis |
| TNFRSF13B |  |  |  |  |  |  |  |  |  |  |
| rs34562254 | Diabetic polyneuropathy | 17 | 16842991 | A/G | 0.100 | -0.145 | 0.126 | 0.247 | 0.100 | cis |
| MCAM |  |  |  |  |  |  |  |  |  |  |
| rs34587557 | Diabetic polyneuropathy | 11 | 119185677 | C/T | 0.046 | -0.107 | 0.182 | 0.556 | 0.046 | cis |
| CPA4 |  |  |  |  |  |  |  |  |  |  |
| rs34587586 | Diabetic polyneuropathy | 7 | 129938598 | T/G | 0.371 | 0.019 | 0.078 | 0.806 | 0.371 | cis |
| SLAMF8 |  |  |  |  |  |  |  |  |  |  |
| rs34687326 | Diabetic polyneuropathy | 1 | 159799910 | A/G | 0.118 | 0.098 | 0.117 | 0.403 | 0.118 | cis |
| NPNT |  |  |  |  |  |  |  |  |  |  |
| rs34712979 | Diabetic polyneuropathy | 4 | 106819053 | A/G | 0.258 | -0.035 | 0.088 | 0.688 | 0.258 | cis |
| ALDH1A1 |  |  |  |  |  |  |  |  |  |  |
| rs348452 | Diabetic polyneuropathy | 9 | 75553385 | T/C | 0.233 | 0.125 | 0.089 | 0.160 | 0.233 | cis |
| TCEA2 |  |  |  |  |  |  |  |  |  |  |
| rs34848242 | Diabetic polyneuropathy | 20 | 62698188 | A/G | 0.311 | -0.003 | 0.082 | 0.971 | 0.311 | cis |
| PSRC1 |  |  |  |  |  |  |  |  |  |  |
| rs34863121 | Diabetic polyneuropathy | 1 | 109823458 | T/C | 0.013 | -0.270 | 0.330 | 0.413 | 0.013 | cis |
| ISLR2 |  |  |  |  |  |  |  |  |  |  |
| rs34868798 | Diabetic polyneuropathy | 15 | 74469716 | C/T | 0.280 | -0.116 | 0.084 | 0.168 | 0.280 | cis |
| ALCAM |  |  |  |  |  |  |  |  |  |  |
| rs34926152 | Diabetic polyneuropathy | 3 | 105264176 | T/G | 0.036 | 0.171 | 0.204 | 0.402 | 0.036 | cis |
| PVRL4 |  |  |  |  |  |  |  |  |  |  |
| rs34990628 | Diabetic polyneuropathy | 1 | 161067085 | T/C | 0.136 | 0.251 | 0.112 | 0.024 | 0.136 | cis |
| TEK |  |  |  |  |  |  |  |  |  |  |
| rs35030851 | Diabetic polyneuropathy | 9 | 27197486 | T/G | 0.018 | 0.199 | 0.278 | 0.476 | 0.018 | cis |
| VWA2 |  |  |  |  |  |  |  |  |  |  |
| rs35060624 | Diabetic polyneuropathy | 10 | 115899540 | T/C | 0.059 | -0.172 | 0.161 | 0.285 | 0.059 | cis |
| AHSG |  |  |  |  |  |  |  |  |  |  |
| rs35094235 | Diabetic polyneuropathy | 3 | 186328951 | G/T | 0.661 | 0.040 | 0.080 | 0.616 | 0.339 | cis |
| BIN1 |  |  |  |  |  |  |  |  |  |  |
| rs35103166 | Diabetic polyneuropathy | 2 | 127882182 | T/C | 0.631 | 0.067 | 0.079 | 0.395 | 0.369 | cis |
| PMM1 |  |  |  |  |  |  |  |  |  |  |
| rs35117663 | Diabetic polyneuropathy | 22 | 42091027 | A/AAG | 0.237 | 0.109 | 0.089 | 0.222 | 0.237 | cis |
| CCDC126 |  |  |  |  |  |  |  |  |  |  |
| rs35121828 | Diabetic polyneuropathy | 7 | 23634985 | A/G | 0.193 | -0.144 | 0.096 | 0.131 | 0.193 | cis |
| SAA1 |  |  |  |  |  |  |  |  |  |  |
| rs35179000 | Diabetic polyneuropathy | 11 | 18290903 | C/T | 0.776 | -0.021 | 0.091 | 0.818 | 0.224 | cis |
| PRKCA |  |  |  |  |  |  |  |  |  |  |
| rs35180366 | Diabetic polyneuropathy | 17 | 64295082 | C/CAT | 0.358 | -0.097 | 0.079 | 0.219 | 0.358 | cis |
| PCSK7 |  |  |  |  |  |  |  |  |  |  |
| rs35186251 | Diabetic polyneuropathy | 11 | 117076708 | T/C | 0.708 | 0.029 | 0.083 | 0.732 | 0.292 | cis |
| CXCL5 |  |  |  |  |  |  |  |  |  |  |
| rs352045 | Diabetic polyneuropathy | 4 | 74864687 | T/G | 0.112 | 0.039 | 0.120 | 0.747 | 0.112 | cis |
| MMP8 |  |  |  |  |  |  |  |  |  |  |
| rs35231465 | Diabetic polyneuropathy | 11 | 102584135 | A/G | 0.018 | 0.062 | 0.280 | 0.826 | 0.018 | cis |
| NT5C2 |  |  |  |  |  |  |  |  |  |  |
| rs35243673 | Diabetic polyneuropathy | 10 | 104934396 | C/CAA | 0.282 | -0.115 | 0.084 | 0.171 | 0.282 | cis |
| TNFRSF8 |  |  |  |  |  |  |  |  |  |  |
| rs35249183 | Diabetic polyneuropathy | 1 | 12099345 | G/A | 0.050 | -0.121 | 0.173 | 0.486 | 0.050 | cis |
| CSTB |  |  |  |  |  |  |  |  |  |  |
| rs35285321 | Diabetic polyneuropathy | 21 | 45201832 | A/G | 0.384 | 0.052 | 0.078 | 0.500 | 0.384 | cis |
| MARCO |  |  |  |  |  |  |  |  |  |  |
| rs35288880 | Diabetic polyneuropathy | 2 | 119728472 | T/C | 0.167 | 0.059 | 0.101 | 0.557 | 0.167 | cis |
| HSD17B14 |  |  |  |  |  |  |  |  |  |  |
| rs35299026 | Diabetic polyneuropathy | 19 | 49318380 | A/G | 0.042 | 0.107 | 0.190 | 0.572 | 0.042 | cis |
| SUMF2 |  |  |  |  |  |  |  |  |  |  |
| rs35384521 | Diabetic polyneuropathy | 7 | 56145825 | A/G | 0.010 | -0.224 | 0.364 | 0.537 | 0.010 | cis |
| MPHOSPH8 |  |  |  |  |  |  |  |  |  |  |
| rs35424555 | Diabetic polyneuropathy | 13 | 20221516 | G/A | 0.042 | -0.234 | 0.190 | 0.218 | 0.042 | cis |
| NECTIN4 |  |  |  |  |  |  |  |  |  |  |
| rs35434391 | Diabetic polyneuropathy | 1 | 161049509 | T/G | 0.137 | 0.265 | 0.112 | 0.018 | 0.137 | cis |
| BAG3 |  |  |  |  |  |  |  |  |  |  |
| rs35434411 | Diabetic polyneuropathy | 10 | 121429394 | A/G | 0.036 | -0.161 | 0.202 | 0.425 | 0.036 | cis |
| LYVE1 |  |  |  |  |  |  |  |  |  |  |
| rs35468145 | Diabetic polyneuropathy | 11 | 10647995 | A/G | 0.071 | -0.032 | 0.147 | 0.826 | 0.071 | cis |
| CD300LF |  |  |  |  |  |  |  |  |  |  |
| rs35489971 | Diabetic polyneuropathy | 17 | 72700943 | G/A | 0.770 | 0.101 | 0.090 | 0.259 | 0.230 | cis |
| COL6A2 |  |  |  |  |  |  |  |  |  |  |
| rs35548026 | Diabetic polyneuropathy | 21 | 47552209 | A/G | 0.085 | 0.021 | 0.135 | 0.879 | 0.085 | cis |
| CASP8 |  |  |  |  |  |  |  |  |  |  |
| rs35550815 | Diabetic polyneuropathy | 2 | 202150914 | A/G | 0.895 | -0.186 | 0.122 | 0.128 | 0.105 | cis |
| CA8 |  |  |  |  |  |  |  |  |  |  |
| rs35552143 | Diabetic polyneuropathy | 8 | 61188688 | A/G | 0.418 | -0.060 | 0.077 | 0.436 | 0.418 | cis |
| PAM |  |  |  |  |  |  |  |  |  |  |
| rs35658696 | Diabetic polyneuropathy | 5 | 102338811 | A/G | 0.943 | -0.204 | 0.163 | 0.209 | 0.057 | cis |
| CFHR5 |  |  |  |  |  |  |  |  |  |  |
| rs72732234 | Diabetic polyneuropathy | 1 | 196303889 | C/T | 0.054 | 0.289 | 0.168 | 0.085 | 0.054 | cis |
| rs35662416 | Diabetic polyneuropathy | 1 | 196967354 | A/G | 0.018 | -0.046 | 0.278 | 0.870 | 0.018 | cis |
| VASN |  |  |  |  |  |  |  |  |  |  |
| rs35713275 | Diabetic polyneuropathy | 16 | 4431360 | A/G | 0.051 | -0.017 | 0.169 | 0.922 | 0.051 | cis |
| S100A16 |  |  |  |  |  |  |  |  |  |  |
| rs35760493 | Diabetic polyneuropathy | 1 | 153596603 | A/G | 0.411 | -0.014 | 0.078 | 0.859 | 0.411 | cis |
| CLIC5 |  |  |  |  |  |  |  |  |  |  |
| rs35822882 | Diabetic polyneuropathy | 6 | 45916999 | T/G | 0.009 | -0.528 | 0.413 | 0.201 | 0.009 | cis |
| RBP4 |  |  |  |  |  |  |  |  |  |  |
| rs36014035 | Diabetic polyneuropathy | 10 | 95360027 | A/C | 0.660 | -0.038 | 0.081 | 0.640 | 0.340 | cis |
| RSPO1 |  |  |  |  |  |  |  |  |  |  |
| rs36043533 | Diabetic polyneuropathy | 1 | 38079517 | G/T | 0.027 | 0.076 | 0.238 | 0.748 | 0.027 | cis |
| NRTN |  |  |  |  |  |  |  |  |  |  |
| rs36055559 | Diabetic polyneuropathy | 19 | 5799433 | A/G | 0.166 | 0.026 | 0.102 | 0.797 | 0.166 | cis |
| VAV1 |  |  |  |  |  |  |  |  |  |  |
| rs36097961 | Diabetic polyneuropathy | 19 | 6850767 | T/C | 0.129 | -0.233 | 0.116 | 0.045 | 0.129 | cis |
| LMNB1 |  |  |  |  |  |  |  |  |  |  |
| rs36105360 | Diabetic polyneuropathy | 5 | 126161690 | T/C | 0.015 | 0.061 | 0.303 | 0.841 | 0.015 | cis |
| LILRA3 |  |  |  |  |  |  |  |  |  |  |
| rs367070 | Diabetic polyneuropathy | 19 | 54800500 | A/G | 0.701 | 0.125 | 0.083 | 0.132 | 0.299 | cis |
| REG3G |  |  |  |  |  |  |  |  |  |  |
| rs369967 | Diabetic polyneuropathy | 2 | 79253150 | G/A | 0.285 | -0.035 | 0.084 | 0.673 | 0.285 | cis |
| LIFR |  |  |  |  |  |  |  |  |  |  |
| rs3729734 | Diabetic polyneuropathy | 5 | 38527308 | A/G | 0.084 | -0.282 | 0.137 | 0.040 | 0.084 | cis |
| HCLS1 |  |  |  |  |  |  |  |  |  |  |
| rs3732410 | Diabetic polyneuropathy | 3 | 121415720 | C/T | 0.210 | 0.044 | 0.093 | 0.637 | 0.210 | cis |
| CPN2 |  |  |  |  |  |  |  |  |  |  |
| rs3732477 | Diabetic polyneuropathy | 3 | 194062519 | T/C | 0.350 | -0.053 | 0.079 | 0.507 | 0.350 | cis |
| TXNDC15 |  |  |  |  |  |  |  |  |  |  |
| rs3733897 | Diabetic polyneuropathy | 5 | 134223593 | G/A | 0.201 | 0.073 | 0.094 | 0.439 | 0.201 | cis |
| DPP6 |  |  |  |  |  |  |  |  |  |  |
| rs3734960 | Diabetic polyneuropathy | 7 | 154684153 | C/T | 0.267 | -0.068 | 0.085 | 0.424 | 0.267 | cis |
| SCGB1A1 |  |  |  |  |  |  |  |  |  |  |
| rs3741240 | Diabetic polyneuropathy | 11 | 62186542 | A/G | 0.345 | -0.011 | 0.080 | 0.891 | 0.345 | cis |
| INHBC |  |  |  |  |  |  |  |  |  |  |
| rs3741414 | Diabetic polyneuropathy | 12 | 57844049 | T/C | 0.233 | 0.249 | 0.089 | 0.005 | 0.233 | cis |
| INHBA_INHBC |  |  |  |  |  |  |  |  |  |  |
| rs3741414 | Diabetic polyneuropathy | 12 | 57844049 | T/C | 0.233 | 0.249 | 0.089 | 0.005 | 0.233 | cis |
| CPB2 |  |  |  |  |  |  |  |  |  |  |
| rs3742264 | Diabetic polyneuropathy | 13 | 46648094 | T/C | 0.380 | -0.017 | 0.078 | 0.828 | 0.380 | cis |
| IGF1R |  |  |  |  |  |  |  |  |  |  |
| rs3743249 | Diabetic polyneuropathy | 15 | 99505423 | T/G | 0.233 | -0.024 | 0.089 | 0.783 | 0.233 | cis |
| KIR2DL4 |  |  |  |  |  |  |  |  |  |  |
| rs3745900 | Diabetic polyneuropathy | 19 | 55377583 | T/C | 0.187 | 0.022 | 0.096 | 0.820 | 0.187 | cis |
| RNASET2 |  |  |  |  |  |  |  |  |  |  |
| rs3756838 | Diabetic polyneuropathy | 6 | 167371251 | A/G | 0.258 | -0.051 | 0.087 | 0.562 | 0.258 | cis |
| CINP |  |  |  |  |  |  |  |  |  |  |
| rs3759562 | Diabetic polyneuropathy | 14 | 102831817 | G/A | 0.183 | 0.051 | 0.097 | 0.600 | 0.183 | cis |
| HBQ1 |  |  |  |  |  |  |  |  |  |  |
| rs3760047 | Diabetic polyneuropathy | 16 | 281299 | A/G | 0.840 | -0.006 | 0.103 | 0.955 | 0.160 | cis |
| IL12RB1 |  |  |  |  |  |  |  |  |  |  |
| rs376008 | Diabetic polyneuropathy | 19 | 18189568 | T/C | 0.321 | 0.100 | 0.081 | 0.219 | 0.321 | cis |
| KLK13 |  |  |  |  |  |  |  |  |  |  |
| rs3760739 | Diabetic polyneuropathy | 19 | 51538561 | T/G | 0.354 | -0.035 | 0.079 | 0.661 | 0.354 | cis |
| FUT3_FUT5 |  |  |  |  |  |  |  |  |  |  |
| rs3760775 | Diabetic polyneuropathy | 19 | 5841356 | T/G | 0.108 | -0.031 | 0.123 | 0.803 | 0.108 | cis |
| GNRH2 |  |  |  |  |  |  |  |  |  |  |
| rs3761244 | Diabetic polyneuropathy | 20 | 3023181 | A/G | 0.805 | 0.147 | 0.095 | 0.122 | 0.195 | cis |
| TFF1 |  |  |  |  |  |  |  |  |  |  |
| rs3761376 | Diabetic polyneuropathy | 21 | 43787038 | A/G | 0.185 | -0.005 | 0.097 | 0.955 | 0.185 | cis |
| B4GALT2 |  |  |  |  |  |  |  |  |  |  |
| rs3762423 | Diabetic polyneuropathy | 1 | 44445667 | T/C | 0.972 | 0.227 | 0.227 | 0.318 | 0.028 | cis |
| ERBB2 |  |  |  |  |  |  |  |  |  |  |
| rs3764354 | Diabetic polyneuropathy | 17 | 37916823 | T/C | 0.185 | -0.113 | 0.099 | 0.253 | 0.185 | cis |
| CA6 |  |  |  |  |  |  |  |  |  |  |
| rs3765963 | Diabetic polyneuropathy | 1 | 9034598 | G/A | 0.278 | -0.113 | 0.084 | 0.182 | 0.278 | cis |
| ROBO1 |  |  |  |  |  |  |  |  |  |  |
| rs3773244 | Diabetic polyneuropathy | 3 | 78784770 | A/G | 0.244 | -0.002 | 0.088 | 0.985 | 0.244 | cis |
| TLR3 |  |  |  |  |  |  |  |  |  |  |
| rs3775291 | Diabetic polyneuropathy | 4 | 187004074 | T/C | 0.315 | -0.077 | 0.081 | 0.342 | 0.315 | cis |
| MAPRE2 |  |  |  |  |  |  |  |  |  |  |
| rs3786314 | Diabetic polyneuropathy | 18 | 32720189 | G/A | 0.397 | 0.010 | 0.077 | 0.898 | 0.397 | cis |
| GSS |  |  |  |  |  |  |  |  |  |  |
| rs3787215 | Diabetic polyneuropathy | 20 | 33493877 | A/G | 0.398 | -0.093 | 0.077 | 0.228 | 0.398 | cis |
| EFEMP1 |  |  |  |  |  |  |  |  |  |  |
| rs3791679 | Diabetic polyneuropathy | 2 | 56096892 | A/G | 0.774 | -0.015 | 0.091 | 0.869 | 0.226 | cis |
| ADGRB3 |  |  |  |  |  |  |  |  |  |  |
| rs3798969 | Diabetic polyneuropathy | 6 | 69671585 | G/A | 0.625 | 0.037 | 0.078 | 0.632 | 0.375 | cis |
| BPHL |  |  |  |  |  |  |  |  |  |  |
| rs3799219 | Diabetic polyneuropathy | 6 | 3151103 | A/G | 0.926 | 0.122 | 0.145 | 0.401 | 0.074 | cis |
| PTPRN2 |  |  |  |  |  |  |  |  |  |  |
| rs3800860 | Diabetic polyneuropathy | 7 | 157903504 | T/C | 0.332 | 0.132 | 0.080 | 0.100 | 0.332 | cis |
| TNFSF13 |  |  |  |  |  |  |  |  |  |  |
| rs3803800 | Diabetic polyneuropathy | 17 | 7462969 | G/A | 0.755 | 0.012 | 0.088 | 0.894 | 0.245 | cis |
| PDIA5 |  |  |  |  |  |  |  |  |  |  |
| rs3804749 | Diabetic polyneuropathy | 3 | 122833003 | T/C | 0.620 | 0.007 | 0.078 | 0.930 | 0.380 | cis |
| COL9A1 |  |  |  |  |  |  |  |  |  |  |
| rs3806095 | Diabetic polyneuropathy | 6 | 70987976 | A/G | 0.263 | 0.075 | 0.086 | 0.385 | 0.263 | cis |
| CRABP2 |  |  |  |  |  |  |  |  |  |  |
| rs3806412 | Diabetic polyneuropathy | 1 | 156676553 | T/G | 0.652 | 0.035 | 0.081 | 0.667 | 0.348 | cis |
| HDGF |  |  |  |  |  |  |  |  |  |  |
| rs3806417 | Diabetic polyneuropathy | 1 | 156711623 | A/C | 0.345 | -0.060 | 0.079 | 0.451 | 0.345 | cis |
| PRSS22 |  |  |  |  |  |  |  |  |  |  |
| rs3810801 | Diabetic polyneuropathy | 16 | 2892370 | A/C | 0.290 | 0.043 | 0.083 | 0.605 | 0.290 | cis |
| LAMB1_LAMC1_LAMA2 |  |  |  |  |  |  |  |  |  |  |
| rs3816665 | Diabetic polyneuropathy | 6 | 129571330 | A/G | 0.179 | -0.028 | 0.100 | 0.781 | 0.179 | cis |
| IDUA |  |  |  |  |  |  |  |  |  |  |
| rs3822020 | Diabetic polyneuropathy | 4 | 985727 | G/A | 0.730 | 0.180 | 0.085 | 0.034 | 0.270 | cis |
| TMEM132B |  |  |  |  |  |  |  |  |  |  |
| rs3825381 | Diabetic polyneuropathy | 12 | 126136863 | T/C | 0.279 | 0.034 | 0.084 | 0.686 | 0.279 | cis |
| PMM2 |  |  |  |  |  |  |  |  |  |  |
| rs3826198 | Diabetic polyneuropathy | 16 | 8906539 | A/C | 0.815 | -0.052 | 0.098 | 0.594 | 0.185 | cis |
| SIGLEC12 |  |  |  |  |  |  |  |  |  |  |
| rs3826667 | Diabetic polyneuropathy | 19 | 52004074 | T/C | 0.812 | -0.116 | 0.097 | 0.234 | 0.188 | cis |
| EGF |  |  |  |  |  |  |  |  |  |  |
| rs3831508 | Diabetic polyneuropathy | 4 | 110837903 | GT/G | 0.368 | -0.055 | 0.078 | 0.483 | 0.368 | cis |
| PTPRU |  |  |  |  |  |  |  |  |  |  |
| rs3835409 | Diabetic polyneuropathy | 1 | 29633491 | T/TCAGCC | 0.644 | 0.030 | 0.079 | 0.708 | 0.357 | cis |
| LILRB2 |  |  |  |  |  |  |  |  |  |  |
| rs383925 | Diabetic polyneuropathy | 19 | 54783521 | T/C | 0.275 | -0.086 | 0.085 | 0.310 | 0.275 | cis |
| SPHK2 |  |  |  |  |  |  |  |  |  |  |
| rs386243 | Diabetic polyneuropathy | 19 | 49127490 | T/C | 0.360 | 0.012 | 0.079 | 0.884 | 0.360 | cis |
| PRPSAP1 |  |  |  |  |  |  |  |  |  |  |
| rs388283 | Diabetic polyneuropathy | 17 | 74335069 | A/G | 0.212 | -0.082 | 0.094 | 0.382 | 0.212 | cis |
| IL1R1 |  |  |  |  |  |  |  |  |  |  |
| rs3917238 | Diabetic polyneuropathy | 2 | 102773083 | T/C | 0.279 | 0.018 | 0.085 | 0.832 | 0.279 | cis |
| WFDC1 |  |  |  |  |  |  |  |  |  |  |
| rs400345 | Diabetic polyneuropathy | 16 | 84328494 | T/C | 0.286 | -0.030 | 0.084 | 0.719 | 0.286 | cis |
| KIRREL2 |  |  |  |  |  |  |  |  |  |  |
| rs404299 | Diabetic polyneuropathy | 19 | 36349752 | A/G | 0.040 | -0.173 | 0.196 | 0.379 | 0.040 | cis |
| ADAMTS16 |  |  |  |  |  |  |  |  |  |  |
| rs40468 | Diabetic polyneuropathy | 5 | 4982814 | G/A | 0.330 | -0.025 | 0.081 | 0.758 | 0.330 | cis |
| CARD9 |  |  |  |  |  |  |  |  |  |  |
| rs4077515 | Diabetic polyneuropathy | 9 | 139266496 | T/C | 0.417 | 0.141 | 0.076 | 0.065 | 0.417 | cis |
| CFHR2 |  |  |  |  |  |  |  |  |  |  |
| rs4085749 | Diabetic polyneuropathy | 1 | 196920148 | T/C | 0.292 | 0.125 | 0.084 | 0.135 | 0.292 | cis |
| LTBP3 |  |  |  |  |  |  |  |  |  |  |
| rs4099470 | Diabetic polyneuropathy | 11 | 65319986 | T/C | 0.052 | -0.231 | 0.173 | 0.183 | 0.052 | cis |
| CTSS |  |  |  |  |  |  |  |  |  |  |
| rs41271951 | Diabetic polyneuropathy | 1 | 150737220 | G/A | 0.065 | 0.031 | 0.156 | 0.841 | 0.065 | cis |
| IL6R |  |  |  |  |  |  |  |  |  |  |
| rs4129267 | Diabetic polyneuropathy | 1 | 154426264 | T/C | 0.297 | -0.019 | 0.083 | 0.814 | 0.297 | cis |
| THSD1 |  |  |  |  |  |  |  |  |  |  |
| rs41292808 | Diabetic polyneuropathy | 13 | 52971517 | T/C | 0.014 | -0.188 | 0.313 | 0.549 | 0.014 | cis |
| COL15A1 |  |  |  |  |  |  |  |  |  |  |
| rs41305481 | Diabetic polyneuropathy | 9 | 101767385 | G/A | 0.340 | 0.107 | 0.080 | 0.180 | 0.340 | cis |
| PIK3AP1 |  |  |  |  |  |  |  |  |  |  |
| rs41317268 | Diabetic polyneuropathy | 10 | 98469114 | G/A | 0.208 | -0.135 | 0.094 | 0.151 | 0.208 | cis |
| CCL23 |  |  |  |  |  |  |  |  |  |  |
| rs41341749 | Diabetic polyneuropathy | 17 | 34310702 | G/A | 0.076 | 0.054 | 0.145 | 0.710 | 0.076 | cis |
| GPR37 |  |  |  |  |  |  |  |  |  |  |
| rs4141005 | Diabetic polyneuropathy | 7 | 124128619 | C/A | 0.189 | -0.009 | 0.096 | 0.926 | 0.189 | cis |
| ASL |  |  |  |  |  |  |  |  |  |  |
| rs4145009 | Diabetic polyneuropathy | 7 | 65726615 | T/C | 0.370 | 0.030 | 0.078 | 0.701 | 0.370 | cis |
| TNFRSF1A |  |  |  |  |  |  |  |  |  |  |
| rs4149584 | Diabetic polyneuropathy | 12 | 6442643 | T/C | 0.011 | -0.640 | 0.380 | 0.092 | 0.011 | cis |
| FGG |  |  |  |  |  |  |  |  |  |  |
| rs4220 | Diabetic polyneuropathy | 4 | 155491759 | A/G | 0.173 | 0.076 | 0.099 | 0.444 | 0.173 | cis |
| PTGFRN |  |  |  |  |  |  |  |  |  |  |
| rs4233450 | Diabetic polyneuropathy | 1 | 117490261 | T/G | 0.744 | 0.154 | 0.087 | 0.076 | 0.256 | cis |
| SORCS2 |  |  |  |  |  |  |  |  |  |  |
| rs4234798 | Diabetic polyneuropathy | 4 | 7219933 | G/T | 0.619 | 0.047 | 0.078 | 0.543 | 0.382 | cis |
| ESM1 |  |  |  |  |  |  |  |  |  |  |
| rs4242051 | Diabetic polyneuropathy | 5 | 54198775 | C/T | 0.763 | -0.017 | 0.089 | 0.851 | 0.237 | cis |
| CLEC4D |  |  |  |  |  |  |  |  |  |  |
| rs4242896 | Diabetic polyneuropathy | 12 | 8688999 | C/A | 0.620 | 0.033 | 0.078 | 0.673 | 0.381 | cis |
| IL12B_IL23A |  |  |  |  |  |  |  |  |  |  |
| rs4244437 | Diabetic polyneuropathy | 5 | 158773117 | A/G | 0.750 | -0.117 | 0.086 | 0.177 | 0.250 | cis |
| IL12A_IL12B |  |  |  |  |  |  |  |  |  |  |
| rs4244437 | Diabetic polyneuropathy | 5 | 158773117 | A/G | 0.750 | -0.117 | 0.086 | 0.177 | 0.250 | cis |
| IL12B |  |  |  |  |  |  |  |  |  |  |
| rs4244437 | Diabetic polyneuropathy | 5 | 158773117 | A/G | 0.750 | -0.117 | 0.086 | 0.177 | 0.250 | cis |
| PSMB4 |  |  |  |  |  |  |  |  |  |  |
| rs4246527 | Diabetic polyneuropathy | 1 | 151403948 | C/T | 0.149 | 0.036 | 0.106 | 0.736 | 0.149 | cis |
| PLEKHA7 |  |  |  |  |  |  |  |  |  |  |
| rs425325 | Diabetic polyneuropathy | 11 | 16885175 | A/G | 0.075 | -0.001 | 0.141 | 0.994 | 0.075 | cis |
| CST2 |  |  |  |  |  |  |  |  |  |  |
| rs4260306 | Diabetic polyneuropathy | 20 | 23730336 | T/C | 0.192 | 0.103 | 0.096 | 0.282 | 0.192 | cis |
| LZIC |  |  |  |  |  |  |  |  |  |  |
| rs4265433 | Diabetic polyneuropathy | 1 | 10125555 | A/G | 0.029 | -0.297 | 0.223 | 0.183 | 0.029 | cis |
| RPIA |  |  |  |  |  |  |  |  |  |  |
| rs4281908 | Diabetic polyneuropathy | 2 | 88970680 | A/G | 0.032 | -0.049 | 0.212 | 0.817 | 0.032 | cis |
| OLFML3 |  |  |  |  |  |  |  |  |  |  |
| rs4381184 | Diabetic polyneuropathy | 1 | 114489769 | A/C | 0.280 | -0.041 | 0.084 | 0.630 | 0.280 | cis |
| STARD5 |  |  |  |  |  |  |  |  |  |  |
| rs4392019 | Diabetic polyneuropathy | 15 | 81616559 | T/C | 0.014 | 0.555 | 0.327 | 0.089 | 0.014 | cis |
| SHMT1 |  |  |  |  |  |  |  |  |  |  |
| rs4398149 | Diabetic polyneuropathy | 17 | 18268999 | A/G | 0.345 | 0.006 | 0.080 | 0.945 | 0.345 | cis |
| PAG1 |  |  |  |  |  |  |  |  |  |  |
| rs4436084 | Diabetic polyneuropathy | 8 | 82038006 | C/T | 0.281 | 0.043 | 0.084 | 0.610 | 0.281 | cis |
| GFRA2 |  |  |  |  |  |  |  |  |  |  |
| rs4443656 | Diabetic polyneuropathy | 8 | 21541084 | G/A | 0.350 | 0.039 | 0.079 | 0.625 | 0.350 | cis |
| OBP2B |  |  |  |  |  |  |  |  |  |  |
| rs4454354 | Diabetic polyneuropathy | 9 | 136089529 | C/T | 0.691 | -0.030 | 0.082 | 0.716 | 0.309 | cis |
| ITIH2 |  |  |  |  |  |  |  |  |  |  |
| rs4463754 | Diabetic polyneuropathy | 10 | 7753366 | A/G | 0.901 | -0.067 | 0.128 | 0.603 | 0.099 | cis |
| PSAPL1 |  |  |  |  |  |  |  |  |  |  |
| rs4484302 | Diabetic polyneuropathy | 4 | 7436997 | T/C | 0.820 | 0.091 | 0.098 | 0.352 | 0.180 | cis |
| PDCD5 |  |  |  |  |  |  |  |  |  |  |
| rs4499344 | Diabetic polyneuropathy | 19 | 33073431 | A/G | 0.300 | 0.107 | 0.082 | 0.192 | 0.300 | cis |
| AKT3 |  |  |  |  |  |  |  |  |  |  |
| rs4515770 | Diabetic polyneuropathy | 1 | 243686913 | C/T | 0.229 | 0.004 | 0.089 | 0.964 | 0.229 | cis |
| MAVS |  |  |  |  |  |  |  |  |  |  |
| rs45437096 | Diabetic polyneuropathy | 20 | 3844929 | T/C | 0.115 | 0.019 | 0.120 | 0.875 | 0.115 | cis |
| TIMP4 |  |  |  |  |  |  |  |  |  |  |
| rs454615 | Diabetic polyneuropathy | 3 | 12077010 | C/T | 0.842 | 0.016 | 0.103 | 0.873 | 0.159 | cis |
| SEMA4D |  |  |  |  |  |  |  |  |  |  |
| rs45464494 | Diabetic polyneuropathy | 9 | 91994433 | T/C | 0.114 | 0.016 | 0.118 | 0.892 | 0.114 | cis |
| CRP |  |  |  |  |  |  |  |  |  |  |
| rs4546916 | Diabetic polyneuropathy | 1 | 159699249 | T/G | 0.362 | 0.036 | 0.079 | 0.651 | 0.362 | cis |
| DEF6 |  |  |  |  |  |  |  |  |  |  |
| rs45482297 | Diabetic polyneuropathy | 6 | 35265535 | A/G | 0.019 | 0.052 | 0.281 | 0.853 | 0.019 | cis |
| FAM20B |  |  |  |  |  |  |  |  |  |  |
| rs45499094 | Diabetic polyneuropathy | 1 | 179057597 | A/C | 0.132 | -0.076 | 0.112 | 0.496 | 0.132 | cis |
| IL32 |  |  |  |  |  |  |  |  |  |  |
| rs45499297 | Diabetic polyneuropathy | 16 | 3115272 | C/T | 0.050 | 0.026 | 0.176 | 0.882 | 0.050 | cis |
| GSTO1 |  |  |  |  |  |  |  |  |  |  |
| rs45596840 | Diabetic polyneuropathy | 10 | 106028154 | A/G | 0.277 | 0.055 | 0.085 | 0.517 | 0.277 | cis |
| CHMP1A |  |  |  |  |  |  |  |  |  |  |
| rs460879 | Diabetic polyneuropathy | 16 | 89712889 | T/C | 0.419 | 0.085 | 0.076 | 0.265 | 0.419 | cis |
| WIF1 |  |  |  |  |  |  |  |  |  |  |
| rs462010 | Diabetic polyneuropathy | 12 | 65338145 | A/G | 0.407 | -0.029 | 0.077 | 0.706 | 0.407 | cis |
| PIK3R1_PIK3CA |  |  |  |  |  |  |  |  |  |  |
| rs4637331 | Diabetic polyneuropathy | 3 | 178767098 | T/C | 0.205 | -0.098 | 0.094 | 0.298 | 0.205 | cis |
| SH3BGRL3 |  |  |  |  |  |  |  |  |  |  |
| rs4659424 | Diabetic polyneuropathy | 1 | 26622993 | T/C | 0.086 | 0.025 | 0.135 | 0.853 | 0.086 | cis |
| PEAR1 |  |  |  |  |  |  |  |  |  |  |
| rs4661012 | Diabetic polyneuropathy | 1 | 156885491 | T/G | 0.628 | -0.012 | 0.078 | 0.876 | 0.372 | cis |
| QPCT |  |  |  |  |  |  |  |  |  |  |
| rs4670696 | Diabetic polyneuropathy | 2 | 37599963 | C/A | 0.054 | -0.050 | 0.167 | 0.765 | 0.054 | cis |
| IGFBP2 |  |  |  |  |  |  |  |  |  |  |
| rs4674100 | Diabetic polyneuropathy | 2 | 217480424 | A/G | 0.312 | -0.001 | 0.083 | 0.986 | 0.312 | cis |
| ERAP1 |  |  |  |  |  |  |  |  |  |  |
| rs467735 | Diabetic polyneuropathy | 5 | 96123160 | T/C | 0.231 | -0.112 | 0.090 | 0.212 | 0.231 | cis |
| CNTN3 |  |  |  |  |  |  |  |  |  |  |
| rs4677414 | Diabetic polyneuropathy | 3 | 74607020 | G/A | 0.955 | -0.091 | 0.180 | 0.614 | 0.045 | cis |
| PLXNA1 |  |  |  |  |  |  |  |  |  |  |
| rs4679138 | Diabetic polyneuropathy | 3 | 126711070 | G/A | 0.307 | -0.319 | 0.082 | 0.000 | 0.307 | cis |
| BOC |  |  |  |  |  |  |  |  |  |  |
| rs4682481 | Diabetic polyneuropathy | 3 | 112983855 | T/C | 0.295 | 0.013 | 0.084 | 0.874 | 0.295 | cis |
| POMGNT2 |  |  |  |  |  |  |  |  |  |  |
| rs4683355 | Diabetic polyneuropathy | 3 | 43145671 | T/C | 0.390 | 0.111 | 0.077 | 0.150 | 0.390 | cis |
| ST6GAL1 |  |  |  |  |  |  |  |  |  |  |
| rs4686837 | Diabetic polyneuropathy | 3 | 186739677 | A/G | 0.300 | -0.138 | 0.083 | 0.095 | 0.300 | cis |
| RAP1GDS1 |  |  |  |  |  |  |  |  |  |  |
| rs4699632 | Diabetic polyneuropathy | 4 | 99349327 | A/G | 0.902 | 0.003 | 0.127 | 0.979 | 0.098 | cis |
| TIMD4 |  |  |  |  |  |  |  |  |  |  |
| rs4704826 | Diabetic polyneuropathy | 5 | 156392082 | A/C | 0.667 | -0.104 | 0.081 | 0.197 | 0.333 | cis |
| SMOC2 |  |  |  |  |  |  |  |  |  |  |
| rs4708467 | Diabetic polyneuropathy | 6 | 168825259 | T/C | 0.308 | 0.127 | 0.082 | 0.119 | 0.308 | cis |
| MMP1 |  |  |  |  |  |  |  |  |  |  |
| rs471994 | Diabetic polyneuropathy | 11 | 102697731 | A/G | 0.343 | 0.078 | 0.080 | 0.326 | 0.343 | cis |
| EPHA1 |  |  |  |  |  |  |  |  |  |  |
| rs4725617 | Diabetic polyneuropathy | 7 | 143097100 | A/G | 0.147 | 0.105 | 0.107 | 0.326 | 0.147 | cis |
| GSR |  |  |  |  |  |  |  |  |  |  |
| rs4733505 | Diabetic polyneuropathy | 8 | 30553430 | A/G | 0.214 | -0.022 | 0.092 | 0.813 | 0.214 | cis |
| GLO1 |  |  |  |  |  |  |  |  |  |  |
| rs4746 | Diabetic polyneuropathy | 6 | 38650628 | G/T | 0.367 | -0.074 | 0.079 | 0.345 | 0.367 | cis |
| SAR1A |  |  |  |  |  |  |  |  |  |  |
| rs4746023 | Diabetic polyneuropathy | 10 | 71912664 | A/G | 0.389 | 0.121 | 0.077 | 0.117 | 0.389 | cis |
| PNLIPRP2 |  |  |  |  |  |  |  |  |  |  |
| rs4751995 | Diabetic polyneuropathy | 10 | 118397884 | G/A | 0.624 | -0.035 | 0.078 | 0.658 | 0.376 | cis |
| PFKM |  |  |  |  |  |  |  |  |  |  |
| rs4760682 | Diabetic polyneuropathy | 12 | 48512285 | C/A | 0.160 | 0.003 | 0.104 | 0.974 | 0.160 | cis |
| GPR116 |  |  |  |  |  |  |  |  |  |  |
| rs476374 | Diabetic polyneuropathy | 6 | 46831060 | T/C | 0.944 | -0.002 | 0.165 | 0.993 | 0.057 | cis |
| OAS1 |  |  |  |  |  |  |  |  |  |  |
| rs4767027 | Diabetic polyneuropathy | 12 | 113359157 | C/T | 0.729 | -0.128 | 0.084 | 0.130 | 0.271 | cis |
| PXN |  |  |  |  |  |  |  |  |  |  |
| rs4767884 | Diabetic polyneuropathy | 12 | 120661977 | C/T | 0.768 | -0.120 | 0.089 | 0.178 | 0.232 | cis |
| IL17D |  |  |  |  |  |  |  |  |  |  |
| rs4770059 | Diabetic polyneuropathy | 13 | 21151047 | C/T | 0.740 | 0.005 | 0.086 | 0.955 | 0.260 | cis |
| MMP13 |  |  |  |  |  |  |  |  |  |  |
| rs478927 | Diabetic polyneuropathy | 11 | 102824826 | T/C | 0.369 | -0.012 | 0.079 | 0.876 | 0.369 | cis |
| KRT20 |  |  |  |  |  |  |  |  |  |  |
| rs4796667 | Diabetic polyneuropathy | 17 | 39678716 | T/C | 0.354 | -0.026 | 0.079 | 0.745 | 0.354 | cis |
| TWSG1 |  |  |  |  |  |  |  |  |  |  |
| rs4798783 | Diabetic polyneuropathy | 18 | 9174885 | A/G | 0.718 | -0.073 | 0.084 | 0.382 | 0.282 | cis |
| DSC2 |  |  |  |  |  |  |  |  |  |  |
| rs4799305 | Diabetic polyneuropathy | 18 | 28689144 | A/G | 0.895 | 0.032 | 0.122 | 0.795 | 0.105 | cis |
| PPP1R14A |  |  |  |  |  |  |  |  |  |  |
| rs4803999 | Diabetic polyneuropathy | 19 | 38787965 | A/G | 0.582 | -0.141 | 0.077 | 0.066 | 0.418 | cis |
| CD40 |  |  |  |  |  |  |  |  |  |  |
| rs4810485 | Diabetic polyneuropathy | 20 | 44747947 | T/G | 0.271 | 0.077 | 0.085 | 0.366 | 0.271 | cis |
| LGALS1 |  |  |  |  |  |  |  |  |  |  |
| rs4820294 | Diabetic polyneuropathy | 22 | 38071043 | A/G | 0.311 | -0.013 | 0.081 | 0.869 | 0.311 | cis |
| PVALB |  |  |  |  |  |  |  |  |  |  |
| rs4821544 | Diabetic polyneuropathy | 22 | 37258503 | C/T | 0.219 | -0.064 | 0.093 | 0.492 | 0.219 | cis |
| CRYBB1 |  |  |  |  |  |  |  |  |  |  |
| rs4822754 | Diabetic polyneuropathy | 22 | 27012894 | A/G | 0.413 | -0.054 | 0.077 | 0.480 | 0.413 | cis |
| APOE |  |  |  |  |  |  |  |  |  |  |
| rs483082 | Diabetic polyneuropathy | 19 | 45416178 | T/G | 0.236 | -0.147 | 0.090 | 0.101 | 0.236 | cis |
| SPOCK1 |  |  |  |  |  |  |  |  |  |  |
| rs4835737 | Diabetic polyneuropathy | 5 | 136895064 | G/A | 0.357 | -0.024 | 0.079 | 0.759 | 0.357 | cis |
| ART3 |  |  |  |  |  |  |  |  |  |  |
| rs4859610 | Diabetic polyneuropathy | 4 | 77000441 | G/A | 0.771 | 0.209 | 0.090 | 0.020 | 0.229 | cis |
| PDLIM4 |  |  |  |  |  |  |  |  |  |  |
| rs4877 | Diabetic polyneuropathy | 5 | 131607588 | T/G | 0.083 | -0.084 | 0.137 | 0.540 | 0.083 | cis |
| GRHPR |  |  |  |  |  |  |  |  |  |  |
| rs4878690 | Diabetic polyneuropathy | 9 | 37422656 | T/C | 0.380 | 0.098 | 0.078 | 0.212 | 0.380 | cis |
| SPINK4 |  |  |  |  |  |  |  |  |  |  |
| rs4879679 | Diabetic polyneuropathy | 9 | 33234019 | T/C | 0.857 | 0.169 | 0.107 | 0.115 | 0.143 | cis |
| NUDT2 |  |  |  |  |  |  |  |  |  |  |
| rs4879779 | Diabetic polyneuropathy | 9 | 34366134 | A/C | 0.242 | -0.018 | 0.088 | 0.843 | 0.242 | cis |
| NAPG |  |  |  |  |  |  |  |  |  |  |
| rs489837 | Diabetic polyneuropathy | 18 | 10485357 | T/C | 0.610 | 0.055 | 0.077 | 0.476 | 0.391 | cis |
| IL27 |  |  |  |  |  |  |  |  |  |  |
| rs4905 | Diabetic polyneuropathy | 19 | 4237067 | A/G | 0.693 | 0.082 | 0.082 | 0.318 | 0.307 | cis |
| WARS |  |  |  |  |  |  |  |  |  |  |
| rs4905957 | Diabetic polyneuropathy | 14 | 100837230 | T/C | 0.800 | 0.182 | 0.094 | 0.053 | 0.200 | cis |
| CTSF |  |  |  |  |  |  |  |  |  |  |
| rs4930383 | Diabetic polyneuropathy | 11 | 66340039 | T/C | 0.789 | -0.143 | 0.092 | 0.120 | 0.211 | cis |
| CCN1 |  |  |  |  |  |  |  |  |  |  |
| rs4949896 | Diabetic polyneuropathy | 1 | 86062336 | T/G | 0.208 | 0.021 | 0.093 | 0.823 | 0.208 | cis |
| MMP16 |  |  |  |  |  |  |  |  |  |  |
| rs4961089 | Diabetic polyneuropathy | 8 | 89348016 | T/C | 0.705 | 0.044 | 0.083 | 0.597 | 0.296 | cis |
| CTRC |  |  |  |  |  |  |  |  |  |  |
| rs497078 | Diabetic polyneuropathy | 1 | 15767036 | T/C | 0.115 | 0.191 | 0.119 | 0.108 | 0.115 | cis |
| GNPTG |  |  |  |  |  |  |  |  |  |  |
| rs4984644 | Diabetic polyneuropathy | 16 | 1407792 | T/C | 0.068 | -0.058 | 0.149 | 0.699 | 0.068 | cis |
| IL34 |  |  |  |  |  |  |  |  |  |  |
| rs4985556 | Diabetic polyneuropathy | 16 | 70694000 | A/C | 0.107 | -0.064 | 0.122 | 0.599 | 0.107 | cis |
| TLR4_LY96 |  |  |  |  |  |  |  |  |  |  |
| rs4986791 | Diabetic polyneuropathy | 9 | 120475602 | T/C | 0.101 | 0.101 | 0.125 | 0.422 | 0.101 | cis |
| LCT |  |  |  |  |  |  |  |  |  |  |
| rs4988235 | Diabetic polyneuropathy | 2 | 136608646 | A/G | 0.595 | 0.066 | 0.078 | 0.395 | 0.405 | cis |
| FBP1 |  |  |  |  |  |  |  |  |  |  |
| rs499206 | Diabetic polyneuropathy | 9 | 97359479 | G/A | 0.946 | 0.176 | 0.168 | 0.295 | 0.054 | cis |
| FBP2 |  |  |  |  |  |  |  |  |  |  |
| rs499480 | Diabetic polyneuropathy | 9 | 97352468 | T/G | 0.899 | 0.169 | 0.126 | 0.178 | 0.101 | cis |
| TMEM106B |  |  |  |  |  |  |  |  |  |  |
| rs5011434 | Diabetic polyneuropathy | 7 | 12268717 | T/C | 0.344 | 0.005 | 0.079 | 0.946 | 0.344 | cis |
| KNG1 |  |  |  |  |  |  |  |  |  |  |
| rs5030062 | Diabetic polyneuropathy | 3 | 186454180 | A/C | 0.652 | 0.045 | 0.079 | 0.573 | 0.348 | cis |
| rs710446 | Diabetic polyneuropathy | 3 | 186459927 | T/C | 0.622 | 0.028 | 0.078 | 0.719 | 0.378 | cis |
| rs76438938 | Diabetic polyneuropathy | 3 | 186461524 | T/C | 0.014 | -0.281 | 0.313 | 0.369 | 0.014 | cis |
| ICAM4 |  |  |  |  |  |  |  |  |  |  |
| rs5030377 | Diabetic polyneuropathy | 19 | 10393234 | A/G | 0.606 | 0.105 | 0.077 | 0.174 | 0.394 | cis |
| SPRY2 |  |  |  |  |  |  |  |  |  |  |
| rs504122 | Diabetic polyneuropathy | 13 | 80911525 | A/G | 0.357 | -0.073 | 0.079 | 0.360 | 0.357 | cis |
| ACADVL |  |  |  |  |  |  |  |  |  |  |
| rs507506 | Diabetic polyneuropathy | 17 | 7118322 | A/G | 0.372 | 0.111 | 0.078 | 0.156 | 0.372 | cis |
| ATP5IF1 |  |  |  |  |  |  |  |  |  |  |
| rs510379 | Diabetic polyneuropathy | 1 | 28535233 | C/T | 0.328 | -0.087 | 0.080 | 0.278 | 0.328 | cis |
| CLEC1B |  |  |  |  |  |  |  |  |  |  |
| rs521040 | Diabetic polyneuropathy | 12 | 10147850 | C/T | 0.246 | -0.070 | 0.087 | 0.421 | 0.246 | cis |
| LAYN |  |  |  |  |  |  |  |  |  |  |
| rs542275 | Diabetic polyneuropathy | 11 | 111431614 | T/C | 0.682 | -0.025 | 0.081 | 0.756 | 0.318 | cis |
| CALCOCO2 |  |  |  |  |  |  |  |  |  |  |
| rs550510 | Diabetic polyneuropathy | 17 | 46926615 | A/G | 0.166 | -0.115 | 0.102 | 0.261 | 0.166 | cis |
| TMEM132A |  |  |  |  |  |  |  |  |  |  |
| rs555835 | Diabetic polyneuropathy | 11 | 60689445 | T/C | 0.311 | -0.031 | 0.081 | 0.702 | 0.311 | cis |
| AGXT |  |  |  |  |  |  |  |  |  |  |
| rs55649245 | Diabetic polyneuropathy | 2 | 241793545 | A/G | 0.314 | 0.079 | 0.082 | 0.335 | 0.314 | cis |
| FCRL6 |  |  |  |  |  |  |  |  |  |  |
| rs55650803 | Diabetic polyneuropathy | 1 | 159783148 | T/C | 0.148 | 0.012 | 0.106 | 0.909 | 0.148 | cis |
| PPFIA1 |  |  |  |  |  |  |  |  |  |  |
| rs55678639 | Diabetic polyneuropathy | 11 | 70316391 | A/G | 0.651 | -0.057 | 0.080 | 0.475 | 0.349 | cis |
| ASGR1 |  |  |  |  |  |  |  |  |  |  |
| rs55714927 | Diabetic polyneuropathy | 17 | 7080316 | T/C | 0.264 | -0.118 | 0.087 | 0.177 | 0.264 | cis |
| NLGN1 |  |  |  |  |  |  |  |  |  |  |
| rs557382 | Diabetic polyneuropathy | 3 | 173113559 | G/T | 0.313 | 0.118 | 0.081 | 0.147 | 0.313 | cis |
| PTPRS |  |  |  |  |  |  |  |  |  |  |
| rs55763631 | Diabetic polyneuropathy | 19 | 5293583 | A/C | 0.086 | 0.059 | 0.135 | 0.662 | 0.086 | cis |
| HAPLN4 |  |  |  |  |  |  |  |  |  |  |
| rs55765017 | Diabetic polyneuropathy | 19 | 19368264 | A/G | 0.100 | -0.063 | 0.124 | 0.613 | 0.100 | cis |
| CAMP |  |  |  |  |  |  |  |  |  |  |
| rs55771110 | Diabetic polyneuropathy | 3 | 47346717 | A/G | 0.218 | 0.021 | 0.093 | 0.821 | 0.218 | cis |
| WFIKKN1 |  |  |  |  |  |  |  |  |  |  |
| rs55798945 | Diabetic polyneuropathy | 16 | 669708 | G/T | 0.287 | 0.062 | 0.085 | 0.463 | 0.287 | cis |
| SSC5D |  |  |  |  |  |  |  |  |  |  |
| rs55799523 | Diabetic polyneuropathy | 19 | 55999545 | A/C | 0.281 | -0.001 | 0.085 | 0.991 | 0.281 | cis |
| TMEM132D |  |  |  |  |  |  |  |  |  |  |
| rs55851595 | Diabetic polyneuropathy | 12 | 130080289 | T/C | 0.105 | -0.035 | 0.124 | 0.777 | 0.105 | cis |
| CTSD |  |  |  |  |  |  |  |  |  |  |
| rs55861089 | Diabetic polyneuropathy | 11 | 1783757 | G/A | 0.055 | -0.224 | 0.166 | 0.178 | 0.055 | cis |
| CA5A |  |  |  |  |  |  |  |  |  |  |
| rs55870502 | Diabetic polyneuropathy | 16 | 87927222 | C/T | 0.148 | -0.138 | 0.106 | 0.192 | 0.148 | cis |
| THY1 |  |  |  |  |  |  |  |  |  |  |
| rs55933700 | Diabetic polyneuropathy | 11 | 119293913 | T/G | 0.023 | 0.016 | 0.244 | 0.949 | 0.023 | cis |
| CRTAC1 |  |  |  |  |  |  |  |  |  |  |
| rs56007204 | Diabetic polyneuropathy | 10 | 99625319 | T/C | 0.163 | -0.033 | 0.103 | 0.748 | 0.163 | cis |
| BSG |  |  |  |  |  |  |  |  |  |  |
| rs56101188 | Diabetic polyneuropathy | 19 | 586745 | C/T | 0.090 | 0.198 | 0.135 | 0.141 | 0.090 | cis |
| ENGASE |  |  |  |  |  |  |  |  |  |  |
| rs56107536 | Diabetic polyneuropathy | 17 | 77071040 | A/C | 0.117 | 0.103 | 0.117 | 0.379 | 0.117 | cis |
| ALDOC |  |  |  |  |  |  |  |  |  |  |
| rs56147019 | Diabetic polyneuropathy | 17 | 26991821 | T/C | 0.012 | -0.244 | 0.338 | 0.470 | 0.012 | cis |
| DNAJB11 |  |  |  |  |  |  |  |  |  |  |
| rs56324474 | Diabetic polyneuropathy | 3 | 186287166 | A/G | 0.051 | -0.134 | 0.172 | 0.434 | 0.051 | cis |
| APOB |  |  |  |  |  |  |  |  |  |  |
| rs563290 | Diabetic polyneuropathy | 2 | 21288226 | G/A | 0.187 | 0.087 | 0.097 | 0.367 | 0.187 | cis |
| RELT |  |  |  |  |  |  |  |  |  |  |
| rs56801796 | Diabetic polyneuropathy | 11 | 73090265 | G/A | 0.285 | 0.069 | 0.084 | 0.414 | 0.285 | cis |
| MANSC1 |  |  |  |  |  |  |  |  |  |  |
| rs56829405 | Diabetic polyneuropathy | 12 | 12496935 | A/G | 0.106 | 0.099 | 0.123 | 0.420 | 0.106 | cis |
| CRADD |  |  |  |  |  |  |  |  |  |  |
| rs56944668 | Diabetic polyneuropathy | 12 | 94243853 | T/C | 0.142 | 0.204 | 0.108 | 0.058 | 0.142 | cis |
| FKBP4 |  |  |  |  |  |  |  |  |  |  |
| rs57120900 | Diabetic polyneuropathy | 12 | 2905888 | A/G | 0.964 | 0.167 | 0.202 | 0.409 | 0.036 | cis |
| FCN2 |  |  |  |  |  |  |  |  |  |  |
| rs7851696 | Diabetic polyneuropathy | 9 | 137779091 | T/G | 0.108 | -0.093 | 0.121 | 0.440 | 0.108 | cis |
| CD14 |  |  |  |  |  |  |  |  |  |  |
| rs5744454 | Diabetic polyneuropathy | 5 | 140013567 | T/G | 0.712 | 0.217 | 0.084 | 0.010 | 0.288 | cis |
| rs5744441 | Diabetic polyneuropathy | 5 | 140016847 | A/G | 0.287 | -0.214 | 0.084 | 0.010 | 0.287 | cis |
| HGF |  |  |  |  |  |  |  |  |  |  |
| rs5745687 | Diabetic polyneuropathy | 7 | 81359051 | T/C | 0.041 | -0.127 | 0.187 | 0.497 | 0.041 | cis |
| LRRC15 |  |  |  |  |  |  |  |  |  |  |
| rs57514363 | Diabetic polyneuropathy | 3 | 194087927 | G/T | 0.160 | 0.017 | 0.104 | 0.872 | 0.160 | cis |
| GSTT2B |  |  |  |  |  |  |  |  |  |  |
| rs5751777 | Diabetic polyneuropathy | 22 | 24267047 | T/C | 0.639 | 0.057 | 0.079 | 0.469 | 0.361 | cis |
| FAM3B |  |  |  |  |  |  |  |  |  |  |
| rs57529409 | Diabetic polyneuropathy | 21 | 42718262 | T/C | 0.042 | -0.020 | 0.190 | 0.917 | 0.042 | cis |
| TIMP3 |  |  |  |  |  |  |  |  |  |  |
| rs5754256 | Diabetic polyneuropathy | 22 | 33159962 | T/C | 0.734 | -0.116 | 0.085 | 0.173 | 0.266 | cis |
| LGALS2 |  |  |  |  |  |  |  |  |  |  |
| rs5756729 | Diabetic polyneuropathy | 22 | 37961353 | T/C | 0.642 | -0.017 | 0.079 | 0.834 | 0.358 | cis |
| S100A4 |  |  |  |  |  |  |  |  |  |  |
| rs58056804 | Diabetic polyneuropathy | 1 | 153524706 | A/G | 0.041 | -0.178 | 0.191 | 0.351 | 0.041 | cis |
| CHST4 |  |  |  |  |  |  |  |  |  |  |
| rs58134724 | Diabetic polyneuropathy | 16 | 71526091 | T/C | 0.303 | -0.120 | 0.082 | 0.146 | 0.303 | cis |
| ERLEC1 |  |  |  |  |  |  |  |  |  |  |
| rs58359565 | Diabetic polyneuropathy | 2 | 53958919 | A/C | 0.236 | 0.028 | 0.089 | 0.752 | 0.236 | cis |
| CD300C |  |  |  |  |  |  |  |  |  |  |
| rs58440339 | Diabetic polyneuropathy | 17 | 72539474 | T/C | 0.141 | -0.040 | 0.109 | 0.713 | 0.141 | cis |
| GRN |  |  |  |  |  |  |  |  |  |  |
| rs5848 | Diabetic polyneuropathy | 17 | 42430244 | T/C | 0.345 | -0.042 | 0.080 | 0.598 | 0.345 | cis |
| HRG |  |  |  |  |  |  |  |  |  |  |
| rs59123177 | Diabetic polyneuropathy | 3 | 186391274 | A/G | 0.286 | -0.048 | 0.084 | 0.564 | 0.286 | cis |
| PTGR1 |  |  |  |  |  |  |  |  |  |  |
| rs59165439 | Diabetic polyneuropathy | 9 | 114357952 | T/C | 0.270 | 0.111 | 0.086 | 0.194 | 0.270 | cis |
| CEL |  |  |  |  |  |  |  |  |  |  |
| rs592267 | Diabetic polyneuropathy | 9 | 135940973 | T/C | 0.002 | -0.498 | 0.758 | 0.511 | 0.002 | cis |
| TNFAIP3 |  |  |  |  |  |  |  |  |  |  |
| rs59693083 | Diabetic polyneuropathy | 6 | 138186532 | A/G | 0.970 | 0.544 | 0.215 | 0.012 | 0.030 | cis |
| TESC |  |  |  |  |  |  |  |  |  |  |
| rs59756806 | Diabetic polyneuropathy | 12 | 117479019 | T/C | 0.714 | -0.107 | 0.083 | 0.200 | 0.286 | cis |
| PDE5A |  |  |  |  |  |  |  |  |  |  |
| rs59867181 | Diabetic polyneuropathy | 4 | 120556467 | T/C | 0.645 | 0.051 | 0.079 | 0.522 | 0.355 | cis |
| POR |  |  |  |  |  |  |  |  |  |  |
| rs59882870 | Diabetic polyneuropathy | 7 | 75638421 | A/G | 0.317 | -0.029 | 0.082 | 0.722 | 0.317 | cis |
| KLK1 |  |  |  |  |  |  |  |  |  |  |
| rs601338 | Diabetic polyneuropathy | 19 | 49206674 | A/G | 0.375 | -0.044 | 0.078 | 0.571 | 0.375 | cis |
| CBLN4 |  |  |  |  |  |  |  |  |  |  |
| rs6024420 | Diabetic polyneuropathy | 20 | 54371100 | T/C | 0.287 | -0.017 | 0.084 | 0.839 | 0.287 | cis |
| F11R |  |  |  |  |  |  |  |  |  |  |
| rs60315407 | Diabetic polyneuropathy | 1 | 161032805 | C/T | 0.359 | -0.035 | 0.079 | 0.658 | 0.359 | cis |
| YWHAB |  |  |  |  |  |  |  |  |  |  |
| rs6031847 | Diabetic polyneuropathy | 20 | 43514203 | T/C | 0.232 | -0.067 | 0.089 | 0.456 | 0.232 | cis |
| F5 |  |  |  |  |  |  |  |  |  |  |
| rs6033 | Diabetic polyneuropathy | 1 | 169521853 | G/A | 0.115 | 0.160 | 0.121 | 0.186 | 0.115 | cis |
| NSFL1C |  |  |  |  |  |  |  |  |  |  |
| rs6033860 | Diabetic polyneuropathy | 20 | 1448575 | G/A | 0.247 | -0.084 | 0.088 | 0.339 | 0.247 | cis |
| SIRPG |  |  |  |  |  |  |  |  |  |  |
| rs6043409 | Diabetic polyneuropathy | 20 | 1616206 | A/G | 0.305 | 0.061 | 0.082 | 0.455 | 0.305 | cis |
| ENTPD6 |  |  |  |  |  |  |  |  |  |  |
| rs6050446 | Diabetic polyneuropathy | 20 | 25195509 | G/A | 0.977 | -0.042 | 0.252 | 0.869 | 0.023 | cis |
| CPNE1 |  |  |  |  |  |  |  |  |  |  |
| rs6060524 | Diabetic polyneuropathy | 20 | 34221155 | T/G | 0.077 | 0.018 | 0.141 | 0.901 | 0.077 | cis |
| TRIM3 |  |  |  |  |  |  |  |  |  |  |
| rs60616451 | Diabetic polyneuropathy | 11 | 6493153 | T/C | 0.881 | -0.064 | 0.117 | 0.583 | 0.119 | cis |
| TNFRSF6B |  |  |  |  |  |  |  |  |  |  |
| rs6062497 | Diabetic polyneuropathy | 20 | 62336258 | T/C | 0.734 | 0.094 | 0.085 | 0.271 | 0.266 | cis |
| PLTP |  |  |  |  |  |  |  |  |  |  |
| rs6073958 | Diabetic polyneuropathy | 20 | 44551855 | T/C | 0.824 | -0.002 | 0.099 | 0.985 | 0.176 | cis |
| GFRAL |  |  |  |  |  |  |  |  |  |  |
| rs60761034 | Diabetic polyneuropathy | 6 | 55258516 | T/C | 0.163 | 0.050 | 0.103 | 0.628 | 0.163 | cis |
| IRAK4 |  |  |  |  |  |  |  |  |  |  |
| rs60813848 | Diabetic polyneuropathy | 12 | 44120092 | T/C | 0.053 | -0.127 | 0.164 | 0.439 | 0.053 | cis |
| SDCBP2 |  |  |  |  |  |  |  |  |  |  |
| rs6109526 | Diabetic polyneuropathy | 20 | 1323192 | T/C | 0.745 | 0.084 | 0.087 | 0.333 | 0.255 | cis |
| CST1 |  |  |  |  |  |  |  |  |  |  |
| rs6114248 | Diabetic polyneuropathy | 20 | 23711882 | A/G | 0.388 | 0.046 | 0.077 | 0.550 | 0.388 | cis |
| SERPINA5 |  |  |  |  |  |  |  |  |  |  |
| rs6119 | Diabetic polyneuropathy | 14 | 95054012 | A/G | 0.891 | -0.207 | 0.121 | 0.087 | 0.109 | cis |
| FLRT3 |  |  |  |  |  |  |  |  |  |  |
| rs6135225 | Diabetic polyneuropathy | 20 | 14678135 | T/G | 0.282 | 0.004 | 0.084 | 0.960 | 0.282 | cis |
| SELP |  |  |  |  |  |  |  |  |  |  |
| rs6136 | Diabetic polyneuropathy | 1 | 169563951 | G/T | 0.122 | 0.198 | 0.117 | 0.090 | 0.122 | cis |
| SORT1 |  |  |  |  |  |  |  |  |  |  |
| rs61394658 | Diabetic polyneuropathy | 1 | 109873290 | A/G | 0.217 | -0.103 | 0.092 | 0.260 | 0.217 | cis |
| THPO |  |  |  |  |  |  |  |  |  |  |
| rs6141 | Diabetic polyneuropathy | 3 | 184090266 | T/C | 0.388 | -0.085 | 0.079 | 0.278 | 0.388 | cis |
| GFER |  |  |  |  |  |  |  |  |  |  |
| rs61516948 | Diabetic polyneuropathy | 16 | 2023322 | T/C | 0.163 | 0.035 | 0.103 | 0.732 | 0.163 | cis |
| RARRES1 |  |  |  |  |  |  |  |  |  |  |
| rs61696028 | Diabetic polyneuropathy | 3 | 158455703 | A/C | 0.910 | -0.032 | 0.132 | 0.807 | 0.090 | cis |
| FTCD |  |  |  |  |  |  |  |  |  |  |
| rs61735836 | Diabetic polyneuropathy | 21 | 47572887 | T/C | 0.061 | -0.175 | 0.166 | 0.290 | 0.061 | cis |
| PAMR1 |  |  |  |  |  |  |  |  |  |  |
| rs61736408 | Diabetic polyneuropathy | 11 | 35456061 | C/T | 0.118 | 0.068 | 0.116 | 0.558 | 0.118 | cis |
| CC2D1A |  |  |  |  |  |  |  |  |  |  |
| rs61740117 | Diabetic polyneuropathy | 19 | 14024269 | T/C | 0.005 | 0.504 | 0.556 | 0.364 | 0.005 | cis |
| HPSE |  |  |  |  |  |  |  |  |  |  |
| rs61751211 | Diabetic polyneuropathy | 4 | 84223426 | T/C | 0.969 | -0.027 | 0.236 | 0.907 | 0.031 | cis |
| RAB21 |  |  |  |  |  |  |  |  |  |  |
| rs61754230 | Diabetic polyneuropathy | 12 | 72179446 | T/C | 0.006 | -0.345 | 0.499 | 0.489 | 0.006 | cis |
| DDX19A |  |  |  |  |  |  |  |  |  |  |
| rs61757207 | Diabetic polyneuropathy | 16 | 70358495 | A/G | 0.994 | 0.538 | 0.498 | 0.280 | 0.006 | cis |
| DDX19B |  |  |  |  |  |  |  |  |  |  |
| rs61757207 | Diabetic polyneuropathy | 16 | 70358495 | A/G | 0.994 | 0.538 | 0.498 | 0.280 | 0.006 | cis |
| INPP5B |  |  |  |  |  |  |  |  |  |  |
| rs61776676 | Diabetic polyneuropathy | 1 | 38364229 | T/C | 0.073 | -0.169 | 0.142 | 0.235 | 0.073 | cis |
| CR2 |  |  |  |  |  |  |  |  |  |  |
| rs61821111 | Diabetic polyneuropathy | 1 | 207595899 | C/T | 0.070 | -0.075 | 0.146 | 0.608 | 0.070 | cis |
| C1orf198 |  |  |  |  |  |  |  |  |  |  |
| rs61826310 | Diabetic polyneuropathy | 1 | 231002496 | T/C | 0.146 | -0.005 | 0.106 | 0.966 | 0.146 | cis |
| FOLH1 |  |  |  |  |  |  |  |  |  |  |
| rs61886537 | Diabetic polyneuropathy | 11 | 49248755 | T/C | 0.963 | 0.072 | 0.208 | 0.731 | 0.037 | cis |
| TNFRSF19 |  |  |  |  |  |  |  |  |  |  |
| rs61947047 | Diabetic polyneuropathy | 13 | 24153909 | T/C | 0.328 | -0.149 | 0.081 | 0.064 | 0.328 | cis |
| ATXN3 |  |  |  |  |  |  |  |  |  |  |
| rs61988390 | Diabetic polyneuropathy | 14 | 92476005 | A/G | 0.674 | 0.079 | 0.080 | 0.323 | 0.326 | cis |
| ARG2 |  |  |  |  |  |  |  |  |  |  |
| rs61990120 | Diabetic polyneuropathy | 14 | 68089420 | A/G | 0.157 | 0.005 | 0.106 | 0.962 | 0.157 | cis |
| NPTX1 |  |  |  |  |  |  |  |  |  |  |
| rs62068268 | Diabetic polyneuropathy | 17 | 78539090 | T/C | 0.068 | 0.053 | 0.150 | 0.726 | 0.068 | cis |
| PLXDC1 |  |  |  |  |  |  |  |  |  |  |
| rs62076601 | Diabetic polyneuropathy | 17 | 37223409 | T/C | 0.026 | -0.180 | 0.234 | 0.443 | 0.026 | cis |
| SULT2A1 |  |  |  |  |  |  |  |  |  |  |
| rs62129966 | Diabetic polyneuropathy | 19 | 48374950 | A/C | 0.152 | -0.060 | 0.106 | 0.572 | 0.152 | cis |
| GKN2 |  |  |  |  |  |  |  |  |  |  |
| rs62133344 | Diabetic polyneuropathy | 2 | 69177269 | A/C | 0.047 | -0.061 | 0.179 | 0.735 | 0.047 | cis |
| HADH |  |  |  |  |  |  |  |  |  |  |
| rs62311424 | Diabetic polyneuropathy | 4 | 108925253 | T/C | 0.933 | 0.383 | 0.152 | 0.012 | 0.067 | cis |
| ADH5 |  |  |  |  |  |  |  |  |  |  |
| rs62325239 | Diabetic polyneuropathy | 4 | 99987492 | A/G | 0.964 | -0.133 | 0.203 | 0.512 | 0.036 | cis |
| ADH6 |  |  |  |  |  |  |  |  |  |  |
| rs62325239 | Diabetic polyneuropathy | 4 | 99987492 | A/G | 0.964 | -0.133 | 0.203 | 0.512 | 0.036 | cis |
| NFKB1 |  |  |  |  |  |  |  |  |  |  |
| rs62328536 | Diabetic polyneuropathy | 4 | 103399049 | A/C | 0.343 | 0.067 | 0.080 | 0.403 | 0.343 | cis |
| SPOCK3 |  |  |  |  |  |  |  |  |  |  |
| rs62353586 | Diabetic polyneuropathy | 4 | 168139859 | A/G | 0.017 | 0.148 | 0.285 | 0.603 | 0.017 | cis |
| GDNF |  |  |  |  |  |  |  |  |  |  |
| rs62360373 | Diabetic polyneuropathy | 5 | 37820741 | A/G | 0.078 | -0.118 | 0.141 | 0.401 | 0.078 | cis |
| CCNH |  |  |  |  |  |  |  |  |  |  |
| rs62368956 | Diabetic polyneuropathy | 5 | 86735368 | A/G | 0.253 | 0.110 | 0.087 | 0.209 | 0.253 | cis |
| TREML2 |  |  |  |  |  |  |  |  |  |  |
| rs62396356 | Diabetic polyneuropathy | 6 | 41167763 | A/G | 0.908 | -0.079 | 0.131 | 0.547 | 0.092 | cis |
| TFPI2 |  |  |  |  |  |  |  |  |  |  |
| rs62466701 | Diabetic polyneuropathy | 7 | 93585663 | C/T | 0.084 | -0.054 | 0.139 | 0.695 | 0.084 | cis |
| LECT2 |  |  |  |  |  |  |  |  |  |  |
| rs62623707 | Diabetic polyneuropathy | 5 | 135288632 | G/A | 0.042 | 0.030 | 0.191 | 0.874 | 0.042 | cis |
| IGF2R |  |  |  |  |  |  |  |  |  |  |
| rs629849 | Diabetic polyneuropathy | 6 | 160494409 | G/A | 0.905 | 0.073 | 0.127 | 0.566 | 0.095 | cis |
| MGAT1 |  |  |  |  |  |  |  |  |  |  |
| rs634501 | Diabetic polyneuropathy | 5 | 180218668 | A/G | 0.215 | -0.068 | 0.097 | 0.482 | 0.215 | cis |
| C2CD2L |  |  |  |  |  |  |  |  |  |  |
| rs640603 | Diabetic polyneuropathy | 11 | 118964330 | A/G | 0.355 | -0.114 | 0.079 | 0.147 | 0.355 | cis |
| FAIM |  |  |  |  |  |  |  |  |  |  |
| rs641320 | Diabetic polyneuropathy | 3 | 138347957 | A/G | 0.031 | -0.013 | 0.221 | 0.953 | 0.031 | cis |
| KRT5 |  |  |  |  |  |  |  |  |  |  |
| rs641615 | Diabetic polyneuropathy | 12 | 52912909 | T/G | 0.242 | -0.052 | 0.088 | 0.554 | 0.242 | cis |
| APOA1BP |  |  |  |  |  |  |  |  |  |  |
| rs6427322 | Diabetic polyneuropathy | 1 | 156563726 | A/C | 0.021 | -0.143 | 0.263 | 0.587 | 0.021 | cis |
| ITGB6 |  |  |  |  |  |  |  |  |  |  |
| rs6432601 | Diabetic polyneuropathy | 2 | 160977943 | G/A | 0.688 | -0.017 | 0.081 | 0.838 | 0.312 | cis |
| IL1RAP |  |  |  |  |  |  |  |  |  |  |
| rs6444442 | Diabetic polyneuropathy | 3 | 190346060 | G/A | 0.857 | 0.045 | 0.109 | 0.684 | 0.143 | cis |
| IL17RB |  |  |  |  |  |  |  |  |  |  |
| rs6445607 | Diabetic polyneuropathy | 3 | 53877149 | T/G | 0.645 | -0.091 | 0.079 | 0.248 | 0.355 | cis |
| GSTA1 |  |  |  |  |  |  |  |  |  |  |
| rs6458869 | Diabetic polyneuropathy | 6 | 52630269 | A/C | 0.624 | 0.024 | 0.078 | 0.763 | 0.376 | cis |
| UPP1 |  |  |  |  |  |  |  |  |  |  |
| rs6463455 | Diabetic polyneuropathy | 7 | 47987296 | A/G | 0.225 | 0.195 | 0.090 | 0.030 | 0.225 | cis |
| PLXNA4 |  |  |  |  |  |  |  |  |  |  |
| rs6467443 | Diabetic polyneuropathy | 7 | 132238011 | T/G | 0.250 | 0.136 | 0.088 | 0.121 | 0.250 | cis |
| LY96 |  |  |  |  |  |  |  |  |  |  |
| rs6472812 | Diabetic polyneuropathy | 8 | 74917084 | G/A | 0.976 | -0.054 | 0.247 | 0.827 | 0.024 | cis |
| CRAT |  |  |  |  |  |  |  |  |  |  |
| rs6478861 | Diabetic polyneuropathy | 9 | 131854303 | C/T | 0.240 | -0.069 | 0.088 | 0.429 | 0.240 | cis |
| NT5C3L |  |  |  |  |  |  |  |  |  |  |
| rs6503667 | Diabetic polyneuropathy | 17 | 39982264 | G/A | 0.340 | 0.108 | 0.080 | 0.180 | 0.340 | cis |
| EVA1C |  |  |  |  |  |  |  |  |  |  |
| rs6517101 | Diabetic polyneuropathy | 21 | 33868483 | G/T | 0.304 | 0.062 | 0.082 | 0.454 | 0.304 | cis |
| COL4A1 |  |  |  |  |  |  |  |  |  |  |
| rs652572 | Diabetic polyneuropathy | 13 | 110819457 | G/A | 0.726 | -0.047 | 0.084 | 0.579 | 0.274 | cis |
| COLEC11 |  |  |  |  |  |  |  |  |  |  |
| rs6542680 | Diabetic polyneuropathy | 2 | 3640142 | T/C | 0.649 | 0.070 | 0.081 | 0.384 | 0.351 | cis |
| IL18RAP |  |  |  |  |  |  |  |  |  |  |
| rs6543140 | Diabetic polyneuropathy | 2 | 103074274 | T/G | 0.340 | 0.008 | 0.080 | 0.925 | 0.340 | cis |
| GLRX |  |  |  |  |  |  |  |  |  |  |
| rs6556886 | Diabetic polyneuropathy | 5 | 95163449 | A/G | 0.070 | 0.060 | 0.147 | 0.683 | 0.070 | cis |
| ROR1 |  |  |  |  |  |  |  |  |  |  |
| rs6588083 | Diabetic polyneuropathy | 1 | 64614011 | C/T | 0.298 | -0.196 | 0.083 | 0.019 | 0.298 | cis |
| IL9 |  |  |  |  |  |  |  |  |  |  |
| rs6596287 | Diabetic polyneuropathy | 5 | 135479348 | A/C | 0.718 | -0.044 | 0.084 | 0.604 | 0.282 | cis |
| NME4 |  |  |  |  |  |  |  |  |  |  |
| rs6600214 | Diabetic polyneuropathy | 16 | 444814 | T/C | 0.310 | -0.095 | 0.082 | 0.248 | 0.310 | cis |
| GAS6 |  |  |  |  |  |  |  |  |  |  |
| rs6602909 | Diabetic polyneuropathy | 13 | 114551993 | C/T | 0.345 | -0.038 | 0.080 | 0.638 | 0.345 | cis |
| RTN4R |  |  |  |  |  |  |  |  |  |  |
| rs663353 | Diabetic polyneuropathy | 22 | 20174712 | A/G | 0.766 | -0.160 | 0.089 | 0.073 | 0.234 | cis |
| FCGR2B |  |  |  |  |  |  |  |  |  |  |
| rs6665610 | Diabetic polyneuropathy | 1 | 161641384 | A/G | 0.173 | -0.124 | 0.100 | 0.215 | 0.173 | cis |
| NFASC |  |  |  |  |  |  |  |  |  |  |
| rs6667532 | Diabetic polyneuropathy | 1 | 204948659 | G/A | 0.080 | -0.041 | 0.138 | 0.767 | 0.080 | cis |
| DRAXIN |  |  |  |  |  |  |  |  |  |  |
| rs6679089 | Diabetic polyneuropathy | 1 | 11752140 | A/G | 0.044 | -0.032 | 0.188 | 0.863 | 0.044 | cis |
| EPHB2 |  |  |  |  |  |  |  |  |  |  |
| rs6687487 | Diabetic polyneuropathy | 1 | 23061551 | A/G | 0.139 | 0.030 | 0.110 | 0.782 | 0.139 | cis |
| DNAJB4 |  |  |  |  |  |  |  |  |  |  |
| rs6699769 | Diabetic polyneuropathy | 1 | 78504264 | A/G | 0.831 | 0.015 | 0.100 | 0.878 | 0.169 | cis |
| CASS4 |  |  |  |  |  |  |  |  |  |  |
| rs67041321 | Diabetic polyneuropathy | 20 | 54988172 | T/G | 0.906 | -0.100 | 0.129 | 0.438 | 0.094 | cis |
| NHEJ1 |  |  |  |  |  |  |  |  |  |  |
| rs6713887 | Diabetic polyneuropathy | 2 | 220026369 | T/G | 0.674 | -0.188 | 0.080 | 0.019 | 0.326 | cis |
| ITGA6 |  |  |  |  |  |  |  |  |  |  |
| rs6714597 | Diabetic polyneuropathy | 2 | 173338486 | C/T | 0.269 | 0.072 | 0.085 | 0.395 | 0.269 | cis |
| RALB |  |  |  |  |  |  |  |  |  |  |
| rs6722943 | Diabetic polyneuropathy | 2 | 121025601 | T/C | 0.595 | -0.008 | 0.077 | 0.921 | 0.405 | cis |
| PTPRJ |  |  |  |  |  |  |  |  |  |  |
| rs67277976 | Diabetic polyneuropathy | 11 | 48275826 | A/C | 0.982 | 0.236 | 0.284 | 0.407 | 0.018 | cis |
| EFNA3 |  |  |  |  |  |  |  |  |  |  |
| rs67333363 | Diabetic polyneuropathy | 1 | 155058046 | G/A | 0.020 | -0.562 | 0.259 | 0.030 | 0.020 | cis |
| IL1RN |  |  |  |  |  |  |  |  |  |  |
| rs6734238 | Diabetic polyneuropathy | 2 | 113841030 | A/G | 0.696 | 0.060 | 0.082 | 0.468 | 0.304 | cis |
| MCEE |  |  |  |  |  |  |  |  |  |  |
| rs6740268 | Diabetic polyneuropathy | 2 | 71345964 | T/G | 0.897 | -0.090 | 0.123 | 0.468 | 0.103 | cis |
| UGT1A6 |  |  |  |  |  |  |  |  |  |  |
| rs6742078 | Diabetic polyneuropathy | 2 | 234672639 | T/G | 0.393 | -0.077 | 0.077 | 0.322 | 0.393 | cis |
| HS6ST1 |  |  |  |  |  |  |  |  |  |  |
| rs6761320 | Diabetic polyneuropathy | 2 | 129050354 | T/C | 0.404 | -0.023 | 0.078 | 0.769 | 0.404 | cis |
| TDGF1 |  |  |  |  |  |  |  |  |  |  |
| rs6769392 | Diabetic polyneuropathy | 3 | 46617719 | A/G | 0.312 | -0.015 | 0.082 | 0.859 | 0.312 | cis |
| CCDC80 |  |  |  |  |  |  |  |  |  |  |
| rs6772337 | Diabetic polyneuropathy | 3 | 112362188 | A/G | 0.397 | 0.100 | 0.077 | 0.196 | 0.397 | cis |
| CD200 |  |  |  |  |  |  |  |  |  |  |
| rs6774405 | Diabetic polyneuropathy | 3 | 112065028 | T/C | 0.139 | 0.114 | 0.109 | 0.295 | 0.139 | cis |
| CR1 |  |  |  |  |  |  |  |  |  |  |
| rs679515 | Diabetic polyneuropathy | 1 | 207750568 | T/C | 0.193 | 0.028 | 0.095 | 0.770 | 0.193 | cis |
| LRRN1 |  |  |  |  |  |  |  |  |  |  |
| rs6801789 | Diabetic polyneuropathy | 3 | 3807592 | C/T | 0.409 | -0.121 | 0.077 | 0.117 | 0.409 | cis |
| SPON2 |  |  |  |  |  |  |  |  |  |  |
| rs6826705 | Diabetic polyneuropathy | 4 | 1122634 | A/G | 0.322 | 0.045 | 0.081 | 0.581 | 0.322 | cis |
| TEC |  |  |  |  |  |  |  |  |  |  |
| rs6845258 | Diabetic polyneuropathy | 4 | 48181697 | A/G | 0.398 | 0.024 | 0.077 | 0.752 | 0.398 | cis |
| IL17B |  |  |  |  |  |  |  |  |  |  |
| rs6862301 | Diabetic polyneuropathy | 5 | 148783690 | A/C | 0.254 | 0.096 | 0.087 | 0.268 | 0.254 | cis |
| FER |  |  |  |  |  |  |  |  |  |  |
| rs6880917 | Diabetic polyneuropathy | 5 | 108080817 | T/C | 0.275 | -0.085 | 0.085 | 0.316 | 0.275 | cis |
| SPINK5 |  |  |  |  |  |  |  |  |  |  |
| rs6883868 | Diabetic polyneuropathy | 5 | 147500883 | T/C | 0.636 | 0.032 | 0.079 | 0.683 | 0.364 | cis |
| CAPG |  |  |  |  |  |  |  |  |  |  |
| rs6886 | Diabetic polyneuropathy | 2 | 85622059 | C/T | 0.612 | -0.098 | 0.078 | 0.205 | 0.388 | cis |
| IL7R |  |  |  |  |  |  |  |  |  |  |
| rs6897932 | Diabetic polyneuropathy | 5 | 35874575 | T/C | 0.332 | 0.054 | 0.080 | 0.498 | 0.332 | cis |
| CD164 |  |  |  |  |  |  |  |  |  |  |
| rs6929023 | Diabetic polyneuropathy | 6 | 109720350 | A/G | 0.375 | -0.081 | 0.078 | 0.304 | 0.375 | cis |
| C9 |  |  |  |  |  |  |  |  |  |  |
| rs696766 | Diabetic polyneuropathy | 5 | 39356126 | A/G | 0.049 | 0.150 | 0.175 | 0.390 | 0.049 | cis |
| MASP1 |  |  |  |  |  |  |  |  |  |  |
| rs698090 | Diabetic polyneuropathy | 3 | 186964300 | T/C | 0.674 | -0.084 | 0.081 | 0.299 | 0.326 | cis |
| ABHD10 |  |  |  |  |  |  |  |  |  |  |
| rs698360 | Diabetic polyneuropathy | 3 | 111681335 | T/C | 0.407 | -0.149 | 0.077 | 0.053 | 0.407 | cis |
| B4GALT1 |  |  |  |  |  |  |  |  |  |  |
| rs7019909 | Diabetic polyneuropathy | 9 | 33113322 | T/C | 0.121 | 0.010 | 0.116 | 0.935 | 0.121 | cis |
| ADAMTSL1 |  |  |  |  |  |  |  |  |  |  |
| rs702208 | Diabetic polyneuropathy | 9 | 18639743 | G/A | 0.052 | -0.005 | 0.173 | 0.979 | 0.052 | cis |
| DDX58 |  |  |  |  |  |  |  |  |  |  |
| rs7025666 | Diabetic polyneuropathy | 9 | 32533829 | T/C | 0.760 | 0.019 | 0.088 | 0.827 | 0.240 | cis |
| OGN |  |  |  |  |  |  |  |  |  |  |
| rs7026361 | Diabetic polyneuropathy | 9 | 95138564 | T/G | 0.298 | -0.103 | 0.082 | 0.213 | 0.298 | cis |
| ITGA2 |  |  |  |  |  |  |  |  |  |  |
| rs7035 | Diabetic polyneuropathy | 5 | 52252191 | T/C | 0.310 | 0.106 | 0.081 | 0.191 | 0.310 | cis |
| VTN |  |  |  |  |  |  |  |  |  |  |
| rs704 | Diabetic polyneuropathy | 17 | 26694861 | A/G | 0.420 | 0.049 | 0.076 | 0.519 | 0.420 | cis |
| VTN_SEBOX |  |  |  |  |  |  |  |  |  |  |
| rs704 | Diabetic polyneuropathy | 17 | 26694861 | A/G | 0.420 | 0.049 | 0.076 | 0.519 | 0.420 | cis |
| SDF2 |  |  |  |  |  |  |  |  |  |  |
| rs704 | Diabetic polyneuropathy | 17 | 26694861 | A/G | 0.420 | 0.049 | 0.076 | 0.519 | 0.420 | cis |
| ORM1 |  |  |  |  |  |  |  |  |  |  |
| rs7040440 | Diabetic polyneuropathy | 9 | 117091074 | C/T | 0.970 | -0.003 | 0.225 | 0.990 | 0.030 | cis |
| FUT3 |  |  |  |  |  |  |  |  |  |  |
| rs708686 | Diabetic polyneuropathy | 19 | 5840619 | T/C | 0.334 | -0.105 | 0.081 | 0.194 | 0.334 | cis |
| RBM17 |  |  |  |  |  |  |  |  |  |  |
| rs7096838 | Diabetic polyneuropathy | 10 | 6137083 | T/C | 0.937 | 0.128 | 0.158 | 0.417 | 0.063 | cis |
| TMPRSS5 |  |  |  |  |  |  |  |  |  |  |
| rs7114195 | Diabetic polyneuropathy | 11 | 113561421 | C/A | 0.627 | -0.059 | 0.078 | 0.446 | 0.373 | cis |
| PDGFD |  |  |  |  |  |  |  |  |  |  |
| rs7115797 | Diabetic polyneuropathy | 11 | 104028877 | A/G | 0.348 | -0.002 | 0.079 | 0.980 | 0.348 | cis |
| UBASH3B |  |  |  |  |  |  |  |  |  |  |
| rs7116968 | Diabetic polyneuropathy | 11 | 122526069 | T/C | 0.215 | 0.062 | 0.092 | 0.497 | 0.215 | cis |
| SAA4 |  |  |  |  |  |  |  |  |  |  |
| rs7117484 | Diabetic polyneuropathy | 11 | 18248214 | A/G | 0.666 | -0.093 | 0.080 | 0.243 | 0.334 | cis |
| CFD |  |  |  |  |  |  |  |  |  |  |
| rs71335276 | Diabetic polyneuropathy | 19 | 859368 | T/G | 0.160 | 0.166 | 0.103 | 0.108 | 0.160 | cis |
| SPINT2 |  |  |  |  |  |  |  |  |  |  |
| rs71354995 | Diabetic polyneuropathy | 19 | 38791841 | G/A | 0.381 | 0.165 | 0.078 | 0.034 | 0.381 | cis |
| PRSS27 |  |  |  |  |  |  |  |  |  |  |
| rs71386687 | Diabetic polyneuropathy | 16 | 2767894 | T/G | 0.117 | 0.007 | 0.121 | 0.957 | 0.117 | cis |
| DGCR14 |  |  |  |  |  |  |  |  |  |  |
| rs715544 | Diabetic polyneuropathy | 22 | 19133605 | A/G | 0.111 | 0.219 | 0.122 | 0.072 | 0.111 | cis |
| AP1G2 |  |  |  |  |  |  |  |  |  |  |
| rs7159262 | Diabetic polyneuropathy | 14 | 24020127 | A/C | 0.907 | -0.078 | 0.131 | 0.550 | 0.093 | cis |
| FGF7 |  |  |  |  |  |  |  |  |  |  |
| rs7162172 | Diabetic polyneuropathy | 15 | 50364841 | C/T | 0.329 | 0.120 | 0.080 | 0.135 | 0.329 | cis |
| MYZAP |  |  |  |  |  |  |  |  |  |  |
| rs7162622 | Diabetic polyneuropathy | 15 | 57857245 | T/C | 0.718 | 0.116 | 0.084 | 0.167 | 0.282 | cis |
| APCS |  |  |  |  |  |  |  |  |  |  |
| rs71632673 | Diabetic polyneuropathy | 1 | 159536213 | A/C | 0.034 | 0.002 | 0.210 | 0.993 | 0.034 | cis |
| CES3 |  |  |  |  |  |  |  |  |  |  |
| rs71647892 | Diabetic polyneuropathy | 16 | 66997756 | C/T | 0.004 | 0.414 | 0.617 | 0.503 | 0.004 | cis |
| IVD |  |  |  |  |  |  |  |  |  |  |
| rs7165012 | Diabetic polyneuropathy | 15 | 40654038 | A/G | 0.338 | -0.045 | 0.080 | 0.569 | 0.338 | cis |
| UNC45A |  |  |  |  |  |  |  |  |  |  |
| rs7173521 | Diabetic polyneuropathy | 15 | 91493207 | T/C | 0.022 | 0.162 | 0.253 | 0.521 | 0.022 | cis |
| MTHFS |  |  |  |  |  |  |  |  |  |  |
| rs7173566 | Diabetic polyneuropathy | 15 | 80211691 | C/T | 0.273 | -0.117 | 0.084 | 0.165 | 0.273 | cis |
| AGRP |  |  |  |  |  |  |  |  |  |  |
| rs7184253 | Diabetic polyneuropathy | 16 | 67380550 | C/T | 0.039 | -0.109 | 0.195 | 0.578 | 0.039 | cis |
| HAGH |  |  |  |  |  |  |  |  |  |  |
| rs7185299 | Diabetic polyneuropathy | 16 | 1879423 | T/C | 0.150 | -0.002 | 0.105 | 0.987 | 0.150 | cis |
| NDE1 |  |  |  |  |  |  |  |  |  |  |
| rs7206272 | Diabetic polyneuropathy | 16 | 15726293 | A/C | 0.751 | 0.064 | 0.088 | 0.464 | 0.249 | cis |
| NME2_NME1-NME2 |  |  |  |  |  |  |  |  |  |  |
| rs7207370 | Diabetic polyneuropathy | 17 | 49231180 | A/G | 0.620 | -0.099 | 0.078 | 0.203 | 0.380 | cis |
| VAT1 |  |  |  |  |  |  |  |  |  |  |
| rs7210098 | Diabetic polyneuropathy | 17 | 41293613 | T/C | 0.385 | 0.116 | 0.078 | 0.136 | 0.385 | cis |
| SFTPD |  |  |  |  |  |  |  |  |  |  |
| rs721917 | Diabetic polyneuropathy | 10 | 81706324 | G/A | 0.404 | -0.030 | 0.077 | 0.700 | 0.404 | cis |
| MXRA7 |  |  |  |  |  |  |  |  |  |  |
| rs7219390 | Diabetic polyneuropathy | 17 | 74684858 | C/T | 0.401 | -0.097 | 0.077 | 0.210 | 0.401 | cis |
| LPO |  |  |  |  |  |  |  |  |  |  |
| rs7219860 | Diabetic polyneuropathy | 17 | 56321271 | A/G | 0.310 | 0.003 | 0.082 | 0.972 | 0.310 | cis |
| CCL14 |  |  |  |  |  |  |  |  |  |  |
| rs7222922 | Diabetic polyneuropathy | 17 | 34335694 | T/C | 0.095 | -0.013 | 0.130 | 0.922 | 0.095 | cis |
| DYNLL2 |  |  |  |  |  |  |  |  |  |  |
| rs7225351 | Diabetic polyneuropathy | 17 | 56155011 | G/A | 0.317 | -0.021 | 0.081 | 0.798 | 0.317 | cis |
| DSG3 |  |  |  |  |  |  |  |  |  |  |
| rs7234091 | Diabetic polyneuropathy | 18 | 29019829 | T/C | 0.210 | 0.005 | 0.093 | 0.955 | 0.210 | cis |
| COLEC12 |  |  |  |  |  |  |  |  |  |  |
| rs7243583 | Diabetic polyneuropathy | 18 | 400766 | T/C | 0.338 | -0.100 | 0.080 | 0.209 | 0.338 | cis |
| ADGRE2 |  |  |  |  |  |  |  |  |  |  |
| rs7251607 | Diabetic polyneuropathy | 19 | 14506991 | C/A | 0.773 | -0.125 | 0.091 | 0.169 | 0.227 | cis |
| HMHA1 |  |  |  |  |  |  |  |  |  |  |
| rs7251797 | Diabetic polyneuropathy | 19 | 1077985 | A/G | 0.657 | -0.004 | 0.081 | 0.963 | 0.343 | cis |
| RTBDN |  |  |  |  |  |  |  |  |  |  |
| rs7255045 | Diabetic polyneuropathy | 19 | 12932269 | A/G | 0.336 | 0.094 | 0.080 | 0.241 | 0.336 | cis |
| LYPD3 |  |  |  |  |  |  |  |  |  |  |
| rs7257767 | Diabetic polyneuropathy | 19 | 44001345 | T/C | 0.074 | -0.225 | 0.143 | 0.116 | 0.074 | cis |
| EMR2 |  |  |  |  |  |  |  |  |  |  |
| rs7260110 | Diabetic polyneuropathy | 19 | 14501544 | G/A | 0.357 | -0.104 | 0.079 | 0.189 | 0.357 | cis |
| CST4 |  |  |  |  |  |  |  |  |  |  |
| rs7263473 | Diabetic polyneuropathy | 20 | 23690467 | A/G | 0.056 | -0.106 | 0.162 | 0.514 | 0.056 | cis |
| KPNA6 |  |  |  |  |  |  |  |  |  |  |
| rs72666722 | Diabetic polyneuropathy | 1 | 32462654 | T/G | 0.019 | 0.250 | 0.284 | 0.379 | 0.019 | cis |
| RNASE6 |  |  |  |  |  |  |  |  |  |  |
| rs72669422 | Diabetic polyneuropathy | 14 | 21251586 | A/C | 0.756 | 0.123 | 0.088 | 0.160 | 0.244 | cis |
| AKR1A1 |  |  |  |  |  |  |  |  |  |  |
| rs72688441 | Diabetic polyneuropathy | 1 | 46051053 | A/G | 0.113 | -0.056 | 0.121 | 0.644 | 0.113 | cis |
| GPA33 |  |  |  |  |  |  |  |  |  |  |
| rs72689400 | Diabetic polyneuropathy | 1 | 167038219 | A/C | 0.020 | 0.460 | 0.268 | 0.086 | 0.020 | cis |
| SF3B4 |  |  |  |  |  |  |  |  |  |  |
| rs72692816 | Diabetic polyneuropathy | 1 | 149902588 | A/G | 0.944 | -0.319 | 0.165 | 0.054 | 0.056 | cis |
| IFI16 |  |  |  |  |  |  |  |  |  |  |
| rs72709516 | Diabetic polyneuropathy | 1 | 159004851 | T/C | 0.044 | 0.017 | 0.181 | 0.927 | 0.044 | cis |
| SORD |  |  |  |  |  |  |  |  |  |  |
| rs72722075 | Diabetic polyneuropathy | 15 | 45323474 | T/C | 0.127 | -0.001 | 0.114 | 0.991 | 0.127 | cis |
| FAIM3 |  |  |  |  |  |  |  |  |  |  |
| rs72758947 | Diabetic polyneuropathy | 1 | 207087053 | G/A | 0.128 | 0.025 | 0.114 | 0.823 | 0.128 | cis |
| FKBP1B |  |  |  |  |  |  |  |  |  |  |
| rs72781699 | Diabetic polyneuropathy | 2 | 24277709 | A/G | 0.146 | 0.107 | 0.106 | 0.310 | 0.146 | cis |
| WWP2 |  |  |  |  |  |  |  |  |  |  |
| rs72783174 | Diabetic polyneuropathy | 16 | 69809678 | T/C | 0.075 | 0.117 | 0.144 | 0.414 | 0.075 | cis |
| TK2 |  |  |  |  |  |  |  |  |  |  |
| rs72790432 | Diabetic polyneuropathy | 16 | 66635531 | A/G | 0.927 | -0.081 | 0.146 | 0.582 | 0.073 | cis |
| OMG |  |  |  |  |  |  |  |  |  |  |
| rs72813607 | Diabetic polyneuropathy | 17 | 29461525 | A/G | 0.105 | -0.067 | 0.122 | 0.586 | 0.105 | cis |
| SOD1 |  |  |  |  |  |  |  |  |  |  |
| rs7282332 | Diabetic polyneuropathy | 21 | 33126239 | A/G | 0.175 | 0.099 | 0.101 | 0.325 | 0.175 | cis |
| CD300LG |  |  |  |  |  |  |  |  |  |  |
| rs72836561 | Diabetic polyneuropathy | 17 | 41926126 | T/C | 0.032 | 0.119 | 0.216 | 0.581 | 0.032 | cis |
| AFM |  |  |  |  |  |  |  |  |  |  |
| rs72856641 | Diabetic polyneuropathy | 4 | 74359582 | T/C | 0.987 | 0.258 | 0.333 | 0.438 | 0.013 | cis |
| DNAJC10 |  |  |  |  |  |  |  |  |  |  |
| rs72898732 | Diabetic polyneuropathy | 2 | 183627183 | T/C | 0.011 | 0.055 | 0.371 | 0.882 | 0.011 | cis |
| PTPRM |  |  |  |  |  |  |  |  |  |  |
| rs72911294 | Diabetic polyneuropathy | 18 | 8253602 | T/C | 0.340 | -0.188 | 0.081 | 0.021 | 0.340 | cis |
| ST3GAL6 |  |  |  |  |  |  |  |  |  |  |
| rs72934623 | Diabetic polyneuropathy | 3 | 98509705 | A/G | 0.064 | 0.094 | 0.156 | 0.547 | 0.064 | cis |
| SELPLG |  |  |  |  |  |  |  |  |  |  |
| rs7294337 | Diabetic polyneuropathy | 12 | 109012694 | T/C | 0.039 | -0.357 | 0.198 | 0.072 | 0.039 | cis |
| LY75 |  |  |  |  |  |  |  |  |  |  |
| rs72957586 | Diabetic polyneuropathy | 2 | 160762687 | A/G | 0.621 | 0.033 | 0.078 | 0.675 | 0.379 | cis |
| MMP19 |  |  |  |  |  |  |  |  |  |  |
| rs7296597 | Diabetic polyneuropathy | 12 | 56241955 | A/G | 0.067 | -0.101 | 0.152 | 0.508 | 0.067 | cis |
| MMP12 |  |  |  |  |  |  |  |  |  |  |
| rs72981675 | Diabetic polyneuropathy | 11 | 102721251 | T/C | 0.182 | -0.189 | 0.100 | 0.059 | 0.182 | cis |
| CHGA |  |  |  |  |  |  |  |  |  |  |
| rs729940 | Diabetic polyneuropathy | 14 | 93399101 | T/C | 0.142 | 0.027 | 0.108 | 0.803 | 0.142 | cis |
| HMBS |  |  |  |  |  |  |  |  |  |  |
| rs72997349 | Diabetic polyneuropathy | 11 | 118945502 | C/T | 0.015 | -0.451 | 0.316 | 0.153 | 0.015 | cis |
| ZBTB16 |  |  |  |  |  |  |  |  |  |  |
| rs73000929 | Diabetic polyneuropathy | 11 | 113953622 | A/G | 0.031 | 0.048 | 0.224 | 0.830 | 0.031 | cis |
| SARS2 |  |  |  |  |  |  |  |  |  |  |
| rs730078 | Diabetic polyneuropathy | 19 | 39421388 | A/G | 0.320 | 0.035 | 0.081 | 0.661 | 0.320 | cis |
| MAN1A2 |  |  |  |  |  |  |  |  |  |  |
| rs73013841 | Diabetic polyneuropathy | 1 | 117854911 | T/C | 0.111 | -0.091 | 0.119 | 0.446 | 0.111 | cis |
| NTF3 |  |  |  |  |  |  |  |  |  |  |
| rs73039984 | Diabetic polyneuropathy | 12 | 5549456 | T/C | 0.076 | -0.002 | 0.143 | 0.991 | 0.076 | cis |
| THBS2 |  |  |  |  |  |  |  |  |  |  |
| rs73043857 | Diabetic polyneuropathy | 6 | 169624900 | G/A | 0.136 | -0.144 | 0.111 | 0.195 | 0.136 | cis |
| ADAMTS15 |  |  |  |  |  |  |  |  |  |  |
| rs73044885 | Diabetic polyneuropathy | 11 | 130344063 | A/G | 0.051 | 0.049 | 0.176 | 0.782 | 0.051 | cis |
| TGFB1 |  |  |  |  |  |  |  |  |  |  |
| rs73045269 | Diabetic polyneuropathy | 19 | 41825191 | T/C | 0.215 | 0.060 | 0.093 | 0.517 | 0.215 | cis |
| CD4 |  |  |  |  |  |  |  |  |  |  |
| rs73053728 | Diabetic polyneuropathy | 12 | 6896546 | G/A | 0.287 | 0.012 | 0.084 | 0.885 | 0.287 | cis |
| USP15 |  |  |  |  |  |  |  |  |  |  |
| rs73139022 | Diabetic polyneuropathy | 12 | 62818841 | T/C | 0.938 | -0.229 | 0.155 | 0.140 | 0.062 | cis |
| MAGI2 |  |  |  |  |  |  |  |  |  |  |
| rs73147474 | Diabetic polyneuropathy | 7 | 79065927 | A/C | 0.867 | 0.044 | 0.111 | 0.692 | 0.133 | cis |
| SNAP29 |  |  |  |  |  |  |  |  |  |  |
| rs73159197 | Diabetic polyneuropathy | 22 | 21040925 | A/G | 0.938 | -0.029 | 0.156 | 0.852 | 0.062 | cis |
| PXDN |  |  |  |  |  |  |  |  |  |  |
| rs73182757 | Diabetic polyneuropathy | 2 | 1747897 | G/A | 0.320 | 0.023 | 0.081 | 0.778 | 0.320 | cis |
| BST1 |  |  |  |  |  |  |  |  |  |  |
| rs73224660 | Diabetic polyneuropathy | 4 | 15714762 | A/G | 0.140 | 0.002 | 0.109 | 0.985 | 0.140 | cis |
| TIGIT |  |  |  |  |  |  |  |  |  |  |
| rs73238029 | Diabetic polyneuropathy | 3 | 114014080 | C/A | 0.216 | -0.247 | 0.092 | 0.007 | 0.216 | cis |
| TACSTD2 |  |  |  |  |  |  |  |  |  |  |
| rs7333 | Diabetic polyneuropathy | 1 | 59041220 | T/C | 0.132 | 0.164 | 0.111 | 0.141 | 0.132 | cis |
| CST3 |  |  |  |  |  |  |  |  |  |  |
| rs734801 | Diabetic polyneuropathy | 20 | 23612791 | A/G | 0.776 | 0.108 | 0.091 | 0.233 | 0.224 | cis |
| CNN1 |  |  |  |  |  |  |  |  |  |  |
| rs73512794 | Diabetic polyneuropathy | 19 | 11620979 | T/C | 0.950 | 0.171 | 0.173 | 0.321 | 0.050 | cis |
| CD28 |  |  |  |  |  |  |  |  |  |  |
| rs7369876 | Diabetic polyneuropathy | 2 | 204636472 | T/C | 0.876 | -0.011 | 0.115 | 0.923 | 0.124 | cis |
| DNAJC30 |  |  |  |  |  |  |  |  |  |  |
| rs73702564 | Diabetic polyneuropathy | 7 | 73084816 | T/C | 0.034 | -0.112 | 0.206 | 0.588 | 0.034 | cis |
| NOTUM |  |  |  |  |  |  |  |  |  |  |
| rs74002709 | Diabetic polyneuropathy | 17 | 79924552 | A/C | 0.056 | -0.055 | 0.164 | 0.736 | 0.056 | cis |
| CEP20 |  |  |  |  |  |  |  |  |  |  |
| rs74009487 | Diabetic polyneuropathy | 16 | 15965897 | T/C | 0.030 | -0.387 | 0.219 | 0.077 | 0.030 | cis |
| CXCL8 |  |  |  |  |  |  |  |  |  |  |
| rs74468557 | Diabetic polyneuropathy | 4 | 74591403 | A/G | 0.041 | -0.105 | 0.192 | 0.585 | 0.041 | cis |
| SCO2 |  |  |  |  |  |  |  |  |  |  |
| rs74479613 | Diabetic polyneuropathy | 22 | 50963905 | T/C | 0.097 | 0.014 | 0.128 | 0.916 | 0.097 | cis |
| C7 |  |  |  |  |  |  |  |  |  |  |
| rs74480769 | Diabetic polyneuropathy | 5 | 40972211 | A/G | 0.916 | 0.018 | 0.139 | 0.899 | 0.084 | cis |
| EGFL7 |  |  |  |  |  |  |  |  |  |  |
| rs74557797 | Diabetic polyneuropathy | 9 | 139550503 | T/G | 0.176 | 0.028 | 0.102 | 0.785 | 0.176 | cis |
| CNPY4 |  |  |  |  |  |  |  |  |  |  |
| rs7457787 | Diabetic polyneuropathy | 7 | 99777869 | C/A | 0.094 | -0.014 | 0.129 | 0.914 | 0.094 | cis |
| RRM2B |  |  |  |  |  |  |  |  |  |  |
| rs74589258 | Diabetic polyneuropathy | 8 | 103215228 | G/A | 0.054 | -0.091 | 0.168 | 0.588 | 0.054 | cis |
| RECQL |  |  |  |  |  |  |  |  |  |  |
| rs74626198 | Diabetic polyneuropathy | 12 | 21696251 | T/C | 0.955 | 0.264 | 0.186 | 0.155 | 0.045 | cis |
| NFU1 |  |  |  |  |  |  |  |  |  |  |
| rs74637005 | Diabetic polyneuropathy | 2 | 69650730 | A/G | 0.019 | 0.193 | 0.271 | 0.476 | 0.019 | cis |
| CD72 |  |  |  |  |  |  |  |  |  |  |
| rs7465764 | Diabetic polyneuropathy | 9 | 35614924 | A/G | 0.972 | -0.562 | 0.228 | 0.014 | 0.028 | cis |
| TBC1D17 |  |  |  |  |  |  |  |  |  |  |
| rs746738 | Diabetic polyneuropathy | 19 | 50386340 | C/T | 0.384 | 0.029 | 0.078 | 0.706 | 0.384 | cis |
| KLK8 |  |  |  |  |  |  |  |  |  |  |
| rs74705037 | Diabetic polyneuropathy | 19 | 51504808 | A/G | 0.015 | -0.278 | 0.304 | 0.361 | 0.015 | cis |
| NPL |  |  |  |  |  |  |  |  |  |  |
| rs74844585 | Diabetic polyneuropathy | 1 | 182781838 | T/C | 0.048 | 0.307 | 0.175 | 0.080 | 0.048 | cis |
| POGLUT3 |  |  |  |  |  |  |  |  |  |  |
| rs74911261 | Diabetic polyneuropathy | 11 | 108357137 | A/G | 0.009 | -0.088 | 0.397 | 0.824 | 0.009 | cis |
| KDELC2 |  |  |  |  |  |  |  |  |  |  |
| rs74911261 | Diabetic polyneuropathy | 11 | 108357137 | A/G | 0.009 | -0.088 | 0.397 | 0.824 | 0.009 | cis |
| MYDGF |  |  |  |  |  |  |  |  |  |  |
| rs75029446 | Diabetic polyneuropathy | 19 | 4701727 | T/C | 0.012 | -0.089 | 0.344 | 0.797 | 0.012 | cis |
| CREG1 |  |  |  |  |  |  |  |  |  |  |
| rs7513428 | Diabetic polyneuropathy | 1 | 167515272 | T/C | 0.099 | -0.067 | 0.125 | 0.593 | 0.099 | cis |
| HDHD2 |  |  |  |  |  |  |  |  |  |  |
| rs75228657 | Diabetic polyneuropathy | 18 | 44741063 | G/A | 0.104 | -0.084 | 0.124 | 0.497 | 0.104 | cis |
| ITLN1 |  |  |  |  |  |  |  |  |  |  |
| rs7532133 | Diabetic polyneuropathy | 1 | 160851534 | A/G | 0.322 | 0.058 | 0.081 | 0.470 | 0.322 | cis |
| PPT1 |  |  |  |  |  |  |  |  |  |  |
| rs7533094 | Diabetic polyneuropathy | 1 | 40559686 | A/G | 0.122 | -0.185 | 0.118 | 0.116 | 0.122 | cis |
| CAMKK1 |  |  |  |  |  |  |  |  |  |  |
| rs753513 | Diabetic polyneuropathy | 17 | 3793472 | T/C | 0.319 | 0.010 | 0.082 | 0.907 | 0.319 | cis |
| SLAMF1 |  |  |  |  |  |  |  |  |  |  |
| rs7535367 | Diabetic polyneuropathy | 1 | 160638250 | T/G | 0.183 | 0.131 | 0.098 | 0.183 | 0.183 | cis |
| AMY1B_AMY1C_AMY1A |  |  |  |  |  |  |  |  |  |  |
| rs7538379 | Diabetic polyneuropathy | 1 | 104067356 | T/C | 0.063 | -0.113 | 0.156 | 0.469 | 0.063 | cis |
| EVL |  |  |  |  |  |  |  |  |  |  |
| rs75416067 | Diabetic polyneuropathy | 14 | 100421429 | C/CT | 0.832 | -0.059 | 0.103 | 0.572 | 0.168 | cis |
| LDLRAP1 |  |  |  |  |  |  |  |  |  |  |
| rs75446219 | Diabetic polyneuropathy | 1 | 25877492 | T/C | 0.031 | -0.056 | 0.220 | 0.798 | 0.031 | cis |
| EPB41 |  |  |  |  |  |  |  |  |  |  |
| rs7556059 | Diabetic polyneuropathy | 1 | 29202162 | T/C | 0.082 | 0.204 | 0.138 | 0.139 | 0.082 | cis |
| IHH |  |  |  |  |  |  |  |  |  |  |
| rs7561119 | Diabetic polyneuropathy | 2 | 219986253 | T/C | 0.896 | -0.013 | 0.123 | 0.918 | 0.104 | cis |
| FXYD5 |  |  |  |  |  |  |  |  |  |  |
| rs756845 | Diabetic polyneuropathy | 19 | 35663250 | G/A | 0.729 | 0.016 | 0.086 | 0.856 | 0.271 | cis |
| TFPI |  |  |  |  |  |  |  |  |  |  |
| rs7576066 | Diabetic polyneuropathy | 2 | 188343781 | A/G | 0.310 | 0.105 | 0.082 | 0.198 | 0.310 | cis |
| CNRIP1 |  |  |  |  |  |  |  |  |  |  |
| rs7578047 | Diabetic polyneuropathy | 2 | 68579931 | G/A | 0.159 | -0.129 | 0.104 | 0.214 | 0.159 | cis |
| ARPC1B |  |  |  |  |  |  |  |  |  |  |
| rs75944999 | Diabetic polyneuropathy | 7 | 98995724 | A/G | 0.066 | -0.067 | 0.152 | 0.660 | 0.066 | cis |
| LILRA5 |  |  |  |  |  |  |  |  |  |  |
| rs759819 | Diabetic polyneuropathy | 19 | 54815577 | C/T | 0.416 | -0.107 | 0.077 | 0.164 | 0.416 | cis |
| PECR |  |  |  |  |  |  |  |  |  |  |
| rs7600044 | Diabetic polyneuropathy | 2 | 216905927 | A/G | 0.070 | -0.167 | 0.148 | 0.260 | 0.070 | cis |
| ITGB2 |  |  |  |  |  |  |  |  |  |  |
| rs760462 | Diabetic polyneuropathy | 21 | 46328099 | C/T | 0.797 | -0.060 | 0.095 | 0.529 | 0.203 | cis |
| NAGK |  |  |  |  |  |  |  |  |  |  |
| rs7606102 | Diabetic polyneuropathy | 2 | 71276399 | G/A | 0.114 | 0.057 | 0.117 | 0.629 | 0.114 | cis |
| MMAB |  |  |  |  |  |  |  |  |  |  |
| rs76118041 | Diabetic polyneuropathy | 12 | 109988723 | A/G | 0.111 | 0.094 | 0.119 | 0.429 | 0.111 | cis |
| POFUT1 |  |  |  |  |  |  |  |  |  |  |
| rs76143353 | Diabetic polyneuropathy | 20 | 30815755 | T/C | 0.048 | 0.293 | 0.177 | 0.099 | 0.048 | cis |
| ODAM |  |  |  |  |  |  |  |  |  |  |
| rs76153614 | Diabetic polyneuropathy | 4 | 71061318 | C/T | 0.052 | -0.163 | 0.171 | 0.340 | 0.052 | cis |
| NOG |  |  |  |  |  |  |  |  |  |  |
| rs76164057 | Diabetic polyneuropathy | 17 | 54840864 | A/G | 0.988 | -0.277 | 0.350 | 0.429 | 0.012 | cis |
| PHPT1 |  |  |  |  |  |  |  |  |  |  |
| rs76229681 | Diabetic polyneuropathy | 9 | 139760830 | C/CG | 0.729 | 0.091 | 0.085 | 0.283 | 0.271 | cis |
| MAX |  |  |  |  |  |  |  |  |  |  |
| rs762810 | Diabetic polyneuropathy | 14 | 65544367 | A/C | 0.395 | -0.060 | 0.077 | 0.434 | 0.395 | cis |
| GLB1 |  |  |  |  |  |  |  |  |  |  |
| rs7637133 | Diabetic polyneuropathy | 3 | 33138763 | A/C | 0.602 | 0.034 | 0.078 | 0.657 | 0.398 | cis |
| LGALSL |  |  |  |  |  |  |  |  |  |  |
| rs76392037 | Diabetic polyneuropathy | 2 | 64568900 | A/C | 0.971 | 0.256 | 0.224 | 0.254 | 0.029 | cis |
| LGALS3 |  |  |  |  |  |  |  |  |  |  |
| rs76424323 | Diabetic polyneuropathy | 14 | 55612573 | A/G | 0.924 | 0.072 | 0.142 | 0.613 | 0.076 | cis |
| PROK2 |  |  |  |  |  |  |  |  |  |  |
| rs7644362 | Diabetic polyneuropathy | 3 | 71829242 | A/G | 0.778 | 0.105 | 0.092 | 0.253 | 0.222 | cis |
| CLEC4G |  |  |  |  |  |  |  |  |  |  |
| rs76560987 | Diabetic polyneuropathy | 19 | 7799452 | T/C | 0.076 | 0.153 | 0.143 | 0.287 | 0.076 | cis |
| HTRA1 |  |  |  |  |  |  |  |  |  |  |
| rs76579910 | Diabetic polyneuropathy | 10 | 124202034 | T/G | 0.047 | -0.207 | 0.176 | 0.240 | 0.047 | cis |
| UGDH |  |  |  |  |  |  |  |  |  |  |
| rs7669821 | Diabetic polyneuropathy | 4 | 39522223 | T/C | 0.620 | 0.021 | 0.078 | 0.789 | 0.380 | cis |
| ANTXR2 |  |  |  |  |  |  |  |  |  |  |
| rs7674623 | Diabetic polyneuropathy | 4 | 80794681 | T/C | 0.166 | -0.071 | 0.103 | 0.486 | 0.166 | cis |
| METAP1 |  |  |  |  |  |  |  |  |  |  |
| rs7676259 | Diabetic polyneuropathy | 4 | 99942649 | A/G | 0.412 | 0.007 | 0.077 | 0.929 | 0.412 | cis |
| REG1A |  |  |  |  |  |  |  |  |  |  |
| rs76841471 | Diabetic polyneuropathy | 2 | 79330169 | T/G | 0.976 | 0.182 | 0.244 | 0.458 | 0.024 | cis |
| CPLX1 |  |  |  |  |  |  |  |  |  |  |
| rs7687101 | Diabetic polyneuropathy | 4 | 812679 | T/G | 0.302 | -0.066 | 0.082 | 0.422 | 0.302 | cis |
| RBP5 |  |  |  |  |  |  |  |  |  |  |
| rs76904513 | Diabetic polyneuropathy | 12 | 7278118 | G/A | 0.053 | 0.199 | 0.169 | 0.239 | 0.053 | cis |
| SEMA4A |  |  |  |  |  |  |  |  |  |  |
| rs7695 | Diabetic polyneuropathy | 1 | 156147326 | C/T | 0.408 | 0.024 | 0.077 | 0.753 | 0.408 | cis |
| GC |  |  |  |  |  |  |  |  |  |  |
| rs7697091 | Diabetic polyneuropathy | 4 | 72625097 | A/C | 0.691 | -0.061 | 0.082 | 0.457 | 0.309 | cis |
| SRI |  |  |  |  |  |  |  |  |  |  |
| rs77010538 | Diabetic polyneuropathy | 7 | 87864495 | A/G | 0.142 | -0.276 | 0.107 | 0.010 | 0.142 | cis |
| GPC1 |  |  |  |  |  |  |  |  |  |  |
| rs77078946 | Diabetic polyneuropathy | 2 | 241403417 | G/T | 0.122 | -0.194 | 0.115 | 0.092 | 0.122 | cis |
| SERPINB13 |  |  |  |  |  |  |  |  |  |  |
| rs77157727 | Diabetic polyneuropathy | 18 | 61253961 | A/G | 0.014 | -0.158 | 0.318 | 0.620 | 0.014 | cis |
| SPINK1 |  |  |  |  |  |  |  |  |  |  |
| rs7725017 | Diabetic polyneuropathy | 5 | 147217823 | A/C | 0.700 | -0.152 | 0.082 | 0.064 | 0.300 | cis |
| DNPH1 |  |  |  |  |  |  |  |  |  |  |
| rs77321231 | Diabetic polyneuropathy | 6 | 43200203 | T/C | 0.085 | 0.076 | 0.133 | 0.567 | 0.085 | cis |
| ITIH4 |  |  |  |  |  |  |  |  |  |  |
| rs77347777 | Diabetic polyneuropathy | 3 | 52848207 | T/C | 0.098 | 0.182 | 0.127 | 0.152 | 0.098 | cis |
| PKLR |  |  |  |  |  |  |  |  |  |  |
| rs77350683 | Diabetic polyneuropathy | 1 | 155484430 | C/T | 0.085 | -0.081 | 0.138 | 0.558 | 0.085 | cis |
| IL5RA |  |  |  |  |  |  |  |  |  |  |
| rs77400868 | Diabetic polyneuropathy | 3 | 3150964 | G/A | 0.075 | -0.313 | 0.145 | 0.031 | 0.075 | cis |
| SERPINB9 |  |  |  |  |  |  |  |  |  |  |
| rs7751676 | Diabetic polyneuropathy | 6 | 2931879 | T/C | 0.057 | 0.304 | 0.162 | 0.061 | 0.057 | cis |
| ACVRL1 |  |  |  |  |  |  |  |  |  |  |
| rs77709482 | Diabetic polyneuropathy | 12 | 52308021 | C/T | 0.108 | 0.080 | 0.122 | 0.509 | 0.108 | cis |
| FABP4 |  |  |  |  |  |  |  |  |  |  |
| rs77878271 | Diabetic polyneuropathy | 8 | 82395535 | A/G | 0.939 | -0.090 | 0.157 | 0.570 | 0.061 | cis |
| FUT5 |  |  |  |  |  |  |  |  |  |  |
| rs778809 | Diabetic polyneuropathy | 19 | 5830302 | A/G | 0.384 | -0.065 | 0.078 | 0.403 | 0.384 | cis |
| RFESD |  |  |  |  |  |  |  |  |  |  |
| rs77881626 | Diabetic polyneuropathy | 5 | 95017852 | G/T | 0.025 | 0.122 | 0.242 | 0.614 | 0.025 | cis |
| EPHB6 |  |  |  |  |  |  |  |  |  |  |
| rs7789303 | Diabetic polyneuropathy | 7 | 142552547 | A/G | 0.738 | 0.129 | 0.086 | 0.133 | 0.262 | cis |
| CCM2 |  |  |  |  |  |  |  |  |  |  |
| rs7805382 | Diabetic polyneuropathy | 7 | 45031315 | T/C | 0.202 | -0.025 | 0.094 | 0.793 | 0.202 | cis |
| CNTNAP2 |  |  |  |  |  |  |  |  |  |  |
| rs7810370 | Diabetic polyneuropathy | 7 | 145366002 | G/A | 0.726 | 0.115 | 0.085 | 0.176 | 0.274 | cis |
| DSCAM |  |  |  |  |  |  |  |  |  |  |
| rs78111814 | Diabetic polyneuropathy | 21 | 42060498 | T/C | 0.938 | -0.044 | 0.158 | 0.779 | 0.062 | cis |
| TAC1 |  |  |  |  |  |  |  |  |  |  |
| rs78145257 | Diabetic polyneuropathy | 7 | 97366332 | A/G | 0.241 | 0.043 | 0.088 | 0.623 | 0.241 | cis |
| AMY2A |  |  |  |  |  |  |  |  |  |  |
| rs78245241 | Diabetic polyneuropathy | 1 | 104335726 | T/G | 0.963 | -0.327 | 0.201 | 0.104 | 0.037 | cis |
| ENPP6 |  |  |  |  |  |  |  |  |  |  |
| rs78293932 | Diabetic polyneuropathy | 4 | 185139101 | T/C | 0.026 | 0.191 | 0.244 | 0.435 | 0.026 | cis |
| PLG |  |  |  |  |  |  |  |  |  |  |
| rs783150 | Diabetic polyneuropathy | 6 | 161226939 | T/C | 0.167 | -0.030 | 0.102 | 0.767 | 0.167 | cis |
| CDH17 |  |  |  |  |  |  |  |  |  |  |
| rs7833351 | Diabetic polyneuropathy | 8 | 95182178 | G/A | 0.364 | 0.011 | 0.079 | 0.890 | 0.364 | cis |
| BDH2 |  |  |  |  |  |  |  |  |  |  |
| rs78336913 | Diabetic polyneuropathy | 4 | 104111414 | T/C | 0.132 | 0.094 | 0.110 | 0.392 | 0.132 | cis |
| DEFA5 |  |  |  |  |  |  |  |  |  |  |
| rs7839771 | Diabetic polyneuropathy | 8 | 6913026 | T/C | 0.013 | 0.602 | 0.344 | 0.080 | 0.013 | cis |
| HSPC159 |  |  |  |  |  |  |  |  |  |  |
| rs78459351 | Diabetic polyneuropathy | 2 | 64593459 | A/G | 0.025 | -0.202 | 0.236 | 0.392 | 0.025 | cis |
| MEGF9 |  |  |  |  |  |  |  |  |  |  |
| rs7849566 | Diabetic polyneuropathy | 9 | 123460769 | C/A | 0.740 | -0.054 | 0.086 | 0.526 | 0.260 | cis |
| IGFBPL1 |  |  |  |  |  |  |  |  |  |  |
| rs7857243 | Diabetic polyneuropathy | 9 | 38409081 | A/G | 0.750 | 0.114 | 0.088 | 0.196 | 0.250 | cis |
| NT5C |  |  |  |  |  |  |  |  |  |  |
| rs78625720 | Diabetic polyneuropathy | 17 | 73140941 | A/G | 0.021 | -0.034 | 0.267 | 0.899 | 0.021 | cis |
| SIAE |  |  |  |  |  |  |  |  |  |  |
| rs78778622 | Diabetic polyneuropathy | 11 | 124530664 | T/C | 0.936 | -0.258 | 0.157 | 0.101 | 0.064 | cis |
| EREG |  |  |  |  |  |  |  |  |  |  |
| rs78803121 | Diabetic polyneuropathy | 4 | 75248505 | T/G | 0.034 | 0.167 | 0.217 | 0.443 | 0.034 | cis |
| C1QC |  |  |  |  |  |  |  |  |  |  |
| rs78865058 | Diabetic polyneuropathy | 1 | 22944209 | A/G | 0.053 | 0.180 | 0.167 | 0.281 | 0.053 | cis |
| CELA2A |  |  |  |  |  |  |  |  |  |  |
| rs78880003 | Diabetic polyneuropathy | 1 | 15740930 | T/C | 0.031 | 0.027 | 0.218 | 0.903 | 0.031 | cis |
| NUDT5 |  |  |  |  |  |  |  |  |  |  |
| rs7895525 | Diabetic polyneuropathy | 10 | 12250620 | T/C | 0.700 | 0.084 | 0.083 | 0.309 | 0.300 | cis |
| TCL1A |  |  |  |  |  |  |  |  |  |  |
| rs78986913 | Diabetic polyneuropathy | 14 | 96162418 | A/G | 0.047 | 0.091 | 0.179 | 0.612 | 0.047 | cis |
| SEMA7A |  |  |  |  |  |  |  |  |  |  |
| rs78994380 | Diabetic polyneuropathy | 15 | 74692412 | A/C | 0.076 | 0.058 | 0.145 | 0.687 | 0.076 | cis |
| MBL2 |  |  |  |  |  |  |  |  |  |  |
| rs7899547 | Diabetic polyneuropathy | 10 | 54536839 | G/T | 0.666 | -0.047 | 0.080 | 0.558 | 0.334 | cis |
| FIS1 |  |  |  |  |  |  |  |  |  |  |
| rs79067051 | Diabetic polyneuropathy | 7 | 100882477 | A/G | 0.059 | -0.011 | 0.162 | 0.945 | 0.059 | cis |
| TAFA5 |  |  |  |  |  |  |  |  |  |  |
| rs79072 | Diabetic polyneuropathy | 22 | 48767186 | T/G | 0.678 | -0.006 | 0.081 | 0.943 | 0.322 | cis |
| LGR4 |  |  |  |  |  |  |  |  |  |  |
| rs79076592 | Diabetic polyneuropathy | 11 | 27391525 | T/C | 0.994 | 0.607 | 0.458 | 0.185 | 0.006 | cis |
| CD74 |  |  |  |  |  |  |  |  |  |  |
| rs79078220 | Diabetic polyneuropathy | 5 | 149793650 | A/G | 0.065 | 0.208 | 0.154 | 0.176 | 0.065 | cis |
| TNFSF10 |  |  |  |  |  |  |  |  |  |  |
| rs79287178 | Diabetic polyneuropathy | 3 | 172294500 | A/G | 0.042 | -0.058 | 0.192 | 0.763 | 0.042 | cis |
| BRSK2 |  |  |  |  |  |  |  |  |  |  |
| rs7932863 | Diabetic polyneuropathy | 11 | 1412848 | A/G | 0.372 | 0.130 | 0.078 | 0.097 | 0.372 | cis |
| SBDS |  |  |  |  |  |  |  |  |  |  |
| rs79344818 | Diabetic polyneuropathy | 7 | 66453476 | A/G | 0.990 | -0.129 | 0.359 | 0.720 | 0.010 | cis |
| GIF |  |  |  |  |  |  |  |  |  |  |
| rs7938323 | Diabetic polyneuropathy | 11 | 60969799 | G/A | 0.332 | 0.114 | 0.081 | 0.158 | 0.332 | cis |
| ETFA |  |  |  |  |  |  |  |  |  |  |
| rs79495512 | Diabetic polyneuropathy | 15 | 76492337 | T/C | 0.877 | 0.043 | 0.116 | 0.712 | 0.123 | cis |
| CLEC1A |  |  |  |  |  |  |  |  |  |  |
| rs7961483 | Diabetic polyneuropathy | 12 | 10230460 | T/C | 0.635 | -0.061 | 0.078 | 0.434 | 0.366 | cis |
| VIM |  |  |  |  |  |  |  |  |  |  |
| rs796667 | Diabetic polyneuropathy | 10 | 16946819 | T/G | 0.667 | -0.027 | 0.080 | 0.739 | 0.333 | cis |
| GSTZ1 |  |  |  |  |  |  |  |  |  |  |
| rs7975 | Diabetic polyneuropathy | 14 | 77793207 | A/G | 0.390 | 0.018 | 0.077 | 0.813 | 0.390 | cis |
| CRACR2A |  |  |  |  |  |  |  |  |  |  |
| rs7977865 | Diabetic polyneuropathy | 12 | 3900555 | A/G | 0.330 | 0.085 | 0.081 | 0.292 | 0.330 | cis |
| CPM |  |  |  |  |  |  |  |  |  |  |
| rs7978197 | Diabetic polyneuropathy | 12 | 69326547 | T/C | 0.003 | -0.543 | 0.702 | 0.439 | 0.003 | cis |
| REG4 |  |  |  |  |  |  |  |  |  |  |
| rs79795228 | Diabetic polyneuropathy | 1 | 120359286 | A/C | 0.023 | 0.376 | 0.260 | 0.148 | 0.023 | cis |
| DCLK1 |  |  |  |  |  |  |  |  |  |  |
| rs7982300 | Diabetic polyneuropathy | 13 | 36641930 | T/C | 0.780 | -0.032 | 0.090 | 0.724 | 0.221 | cis |
| MME |  |  |  |  |  |  |  |  |  |  |
| rs79837905 | Diabetic polyneuropathy | 3 | 154785591 | G/A | 0.065 | 0.021 | 0.154 | 0.893 | 0.065 | cis |
| FAM177A1 |  |  |  |  |  |  |  |  |  |  |
| rs799473 | Diabetic polyneuropathy | 14 | 35516213 | T/G | 0.858 | 0.112 | 0.108 | 0.302 | 0.142 | cis |
| TNFSF12 |  |  |  |  |  |  |  |  |  |  |
| rs80067372 | Diabetic polyneuropathy | 17 | 7452752 | A/G | 0.249 | -0.026 | 0.087 | 0.762 | 0.249 | cis |
| MYBPC1 |  |  |  |  |  |  |  |  |  |  |
| rs80087033 | Diabetic polyneuropathy | 12 | 102048447 | A/G | 0.162 | -0.170 | 0.103 | 0.098 | 0.162 | cis |
| SRA1 |  |  |  |  |  |  |  |  |  |  |
| rs801458 | Diabetic polyneuropathy | 5 | 139953189 | T/C | 0.370 | 0.263 | 0.078 | 0.001 | 0.370 | cis |
| SERPINA3 |  |  |  |  |  |  |  |  |  |  |
| rs8023057 | Diabetic polyneuropathy | 14 | 95097556 | A/G | 0.852 | 0.007 | 0.106 | 0.946 | 0.148 | cis |
| TYRO3 |  |  |  |  |  |  |  |  |  |  |
| rs8024626 | Diabetic polyneuropathy | 15 | 41863181 | A/G | 0.314 | -0.051 | 0.082 | 0.531 | 0.314 | cis |
| CTRB1 |  |  |  |  |  |  |  |  |  |  |
| rs8051363 | Diabetic polyneuropathy | 16 | 75255217 | G/A | 0.802 | 0.018 | 0.095 | 0.853 | 0.198 | cis |
| IL4R |  |  |  |  |  |  |  |  |  |  |
| rs8060025 | Diabetic polyneuropathy | 16 | 27327214 | G/T | 0.653 | 0.130 | 0.079 | 0.099 | 0.347 | cis |
| KCNAB2 |  |  |  |  |  |  |  |  |  |  |
| rs806109 | Diabetic polyneuropathy | 1 | 6052581 | A/G | 0.789 | 0.071 | 0.093 | 0.443 | 0.212 | cis |
| CCL4L2_CCL4L1 |  |  |  |  |  |  |  |  |  |  |
| rs8064426 | Diabetic polyneuropathy | 17 | 34819750 | A/G | 0.218 | -0.161 | 0.093 | 0.081 | 0.218 | cis |
| CCL4 |  |  |  |  |  |  |  |  |  |  |
| rs8064426 | Diabetic polyneuropathy | 17 | 34819750 | A/G | 0.218 | -0.161 | 0.093 | 0.081 | 0.218 | cis |
| SCARF1 |  |  |  |  |  |  |  |  |  |  |
| rs8071756 | Diabetic polyneuropathy | 17 | 1574342 | G/A | 0.194 | -0.046 | 0.095 | 0.631 | 0.194 | cis |
| ERN1 |  |  |  |  |  |  |  |  |  |  |
| rs8076809 | Diabetic polyneuropathy | 17 | 62181308 | A/C | 0.753 | 0.092 | 0.088 | 0.293 | 0.247 | cis |
| BST2 |  |  |  |  |  |  |  |  |  |  |
| rs8101243 | Diabetic polyneuropathy | 19 | 17488793 | C/T | 0.901 | 0.222 | 0.129 | 0.086 | 0.099 | cis |
| TPM4 |  |  |  |  |  |  |  |  |  |  |
| rs8104011 | Diabetic polyneuropathy | 19 | 16196764 | A/G | 0.007 | 0.363 | 0.432 | 0.401 | 0.007 | cis |
| APMAP |  |  |  |  |  |  |  |  |  |  |
| rs8125909 | Diabetic polyneuropathy | 20 | 24975835 | C/A | 0.054 | -0.009 | 0.164 | 0.958 | 0.054 | cis |
| APP |  |  |  |  |  |  |  |  |  |  |
| rs8131895 | Diabetic polyneuropathy | 21 | 27503527 | A/C | 0.673 | 0.009 | 0.080 | 0.910 | 0.327 | cis |
| KEL |  |  |  |  |  |  |  |  |  |  |
| rs8176059 | Diabetic polyneuropathy | 7 | 142651354 | A/G | 0.006 | 0.530 | 0.506 | 0.295 | 0.006 | cis |
| NELL1 |  |  |  |  |  |  |  |  |  |  |
| rs8176786 | Diabetic polyneuropathy | 11 | 20959394 | T/C | 0.050 | -0.106 | 0.174 | 0.541 | 0.050 | cis |
| TF |  |  |  |  |  |  |  |  |  |  |
| rs8177245 | Diabetic polyneuropathy | 3 | 133479230 | G/A | 0.304 | 0.046 | 0.082 | 0.571 | 0.304 | cis |
| MFNG |  |  |  |  |  |  |  |  |  |  |
| rs8192548 | Diabetic polyneuropathy | 22 | 37866063 | A/G | 0.026 | 0.326 | 0.241 | 0.175 | 0.026 | cis |
| GZMB |  |  |  |  |  |  |  |  |  |  |
| rs8192917 | Diabetic polyneuropathy | 14 | 25102160 | T/C | 0.798 | 0.151 | 0.094 | 0.106 | 0.202 | cis |
| CD274 |  |  |  |  |  |  |  |  |  |  |
| rs822340 | Diabetic polyneuropathy | 9 | 5453260 | G/A | 0.727 | 0.068 | 0.085 | 0.421 | 0.273 | cis |
| PPID |  |  |  |  |  |  |  |  |  |  |
| rs8396 | Diabetic polyneuropathy | 4 | 159630817 | C/T | 0.259 | -0.111 | 0.086 | 0.199 | 0.259 | cis |
| C1QTNF3 |  |  |  |  |  |  |  |  |  |  |
| rs840390 | Diabetic polyneuropathy | 5 | 34018623 | A/G | 0.161 | -0.005 | 0.104 | 0.962 | 0.161 | cis |
| ECI2 |  |  |  |  |  |  |  |  |  |  |
| rs853417 | Diabetic polyneuropathy | 6 | 4113123 | A/G | 0.250 | -0.081 | 0.087 | 0.357 | 0.250 | cis |
| CCL15 |  |  |  |  |  |  |  |  |  |  |
| rs854624 | Diabetic polyneuropathy | 17 | 34327923 | T/G | 0.894 | 0.074 | 0.124 | 0.552 | 0.106 | cis |
| SHBG |  |  |  |  |  |  |  |  |  |  |
| rs858519 | Diabetic polyneuropathy | 17 | 7531965 | T/C | 0.378 | 0.136 | 0.078 | 0.082 | 0.378 | cis |
| UROD |  |  |  |  |  |  |  |  |  |  |
| rs8681 | Diabetic polyneuropathy | 1 | 45468606 | A/G | 0.249 | 0.084 | 0.087 | 0.334 | 0.249 | cis |
| CASP3 |  |  |  |  |  |  |  |  |  |  |
| rs870825 | Diabetic polyneuropathy | 4 | 185588045 | G/A | 0.134 | -0.278 | 0.112 | 0.013 | 0.134 | cis |
| CHIT1 |  |  |  |  |  |  |  |  |  |  |
| rs872583 | Diabetic polyneuropathy | 1 | 203184766 | C/T | 0.183 | -0.098 | 0.098 | 0.320 | 0.183 | cis |
| SNCG |  |  |  |  |  |  |  |  |  |  |
| rs873110 | Diabetic polyneuropathy | 10 | 88724619 | T/C | 0.203 | -0.154 | 0.094 | 0.103 | 0.203 | cis |
| ARSA |  |  |  |  |  |  |  |  |  |  |
| rs873697 | Diabetic polyneuropathy | 22 | 51064169 | A/G | 0.015 | 0.413 | 0.305 | 0.175 | 0.015 | cis |
| OXT |  |  |  |  |  |  |  |  |  |  |
| rs877172 | Diabetic polyneuropathy | 20 | 3049890 | G/T | 0.312 | 0.061 | 0.084 | 0.466 | 0.312 | cis |
| SERPINA4 |  |  |  |  |  |  |  |  |  |  |
| rs882732 | Diabetic polyneuropathy | 14 | 95027722 | A/G | 0.300 | -0.057 | 0.083 | 0.489 | 0.300 | cis |
| DLD |  |  |  |  |  |  |  |  |  |  |
| rs886774 | Diabetic polyneuropathy | 7 | 107495434 | A/G | 0.611 | -0.093 | 0.078 | 0.229 | 0.389 | cis |
| ALDH3A1 |  |  |  |  |  |  |  |  |  |  |
| rs887241 | Diabetic polyneuropathy | 17 | 19645938 | C/A | 0.663 | -0.048 | 0.080 | 0.545 | 0.337 | cis |
| LTBP2 |  |  |  |  |  |  |  |  |  |  |
| rs888414 | Diabetic polyneuropathy | 14 | 75104905 | A/G | 0.288 | -0.063 | 0.083 | 0.449 | 0.288 | cis |
| PRSS8 |  |  |  |  |  |  |  |  |  |  |
| rs889555 | Diabetic polyneuropathy | 16 | 31122571 | T/C | 0.330 | -0.104 | 0.080 | 0.198 | 0.330 | cis |
| GALNT10 |  |  |  |  |  |  |  |  |  |  |
| rs890793 | Diabetic polyneuropathy | 5 | 153573938 | T/C | 0.340 | -0.070 | 0.080 | 0.380 | 0.340 | cis |
| A1BG |  |  |  |  |  |  |  |  |  |  |
| rs893184 | Diabetic polyneuropathy | 19 | 58864479 | T/C | 0.042 | -0.067 | 0.186 | 0.720 | 0.042 | cis |
| CNTN5 |  |  |  |  |  |  |  |  |  |  |
| rs898776 | Diabetic polyneuropathy | 11 | 99089041 | A/G | 0.897 | -0.039 | 0.124 | 0.752 | 0.103 | cis |
| KYNU |  |  |  |  |  |  |  |  |  |  |
| rs9013 | Diabetic polyneuropathy | 2 | 143798189 | G/A | 0.153 | 0.192 | 0.104 | 0.065 | 0.153 | cis |
| LSP1 |  |  |  |  |  |  |  |  |  |  |
| rs907612 | Diabetic polyneuropathy | 11 | 1874221 | T/C | 0.385 | 0.029 | 0.078 | 0.715 | 0.385 | cis |
| CLEC10A |  |  |  |  |  |  |  |  |  |  |
| rs90951 | Diabetic polyneuropathy | 17 | 6981397 | G/A | 0.382 | 0.039 | 0.078 | 0.622 | 0.382 | cis |
| SLPI |  |  |  |  |  |  |  |  |  |  |
| rs916311 | Diabetic polyneuropathy | 20 | 43736533 | T/G | 0.350 | -0.006 | 0.079 | 0.944 | 0.350 | cis |
| DUT |  |  |  |  |  |  |  |  |  |  |
| rs919129 | Diabetic polyneuropathy | 15 | 48675227 | G/A | 0.149 | 0.027 | 0.106 | 0.802 | 0.149 | cis |
| EPHA2 |  |  |  |  |  |  |  |  |  |  |
| rs924204 | Diabetic polyneuropathy | 1 | 16513926 | G/A | 0.690 | -0.047 | 0.082 | 0.568 | 0.310 | cis |
| KIAA2013 |  |  |  |  |  |  |  |  |  |  |
| rs926270 | Diabetic polyneuropathy | 1 | 11969786 | T/G | 0.387 | -0.095 | 0.077 | 0.220 | 0.387 | cis |
| OSMR |  |  |  |  |  |  |  |  |  |  |
| rs9292723 | Diabetic polyneuropathy | 5 | 38852572 | T/C | 0.199 | -0.043 | 0.095 | 0.655 | 0.199 | cis |
| FAM20A |  |  |  |  |  |  |  |  |  |  |
| rs929477 | Diabetic polyneuropathy | 17 | 66655816 | A/G | 0.141 | 0.112 | 0.110 | 0.309 | 0.141 | cis |
| GNPDA1 |  |  |  |  |  |  |  |  |  |  |
| rs9324861 | Diabetic polyneuropathy | 5 | 141396566 | T/C | 0.187 | 0.156 | 0.097 | 0.108 | 0.187 | cis |
| MDGA1 |  |  |  |  |  |  |  |  |  |  |
| rs9349050 | Diabetic polyneuropathy | 6 | 37663624 | C/T | 0.588 | -0.031 | 0.077 | 0.683 | 0.412 | cis |
| PAEP |  |  |  |  |  |  |  |  |  |  |
| rs9409964 | Diabetic polyneuropathy | 9 | 138471928 | A/G | 0.129 | -0.288 | 0.112 | 0.010 | 0.129 | cis |
| CHST15 |  |  |  |  |  |  |  |  |  |  |
| rs9422302 | Diabetic polyneuropathy | 10 | 125865777 | T/G | 0.276 | -0.029 | 0.086 | 0.733 | 0.276 | cis |
| ESD |  |  |  |  |  |  |  |  |  |  |
| rs947409 | Diabetic polyneuropathy | 13 | 47377928 | G/A | 0.063 | -0.144 | 0.155 | 0.355 | 0.063 | cis |
| VNN2 |  |  |  |  |  |  |  |  |  |  |
| rs9493423 | Diabetic polyneuropathy | 6 | 133074133 | A/G | 0.351 | 0.197 | 0.079 | 0.013 | 0.351 | cis |
| PDE3A |  |  |  |  |  |  |  |  |  |  |
| rs954865 | Diabetic polyneuropathy | 12 | 20834162 | A/G | 0.301 | 0.209 | 0.082 | 0.011 | 0.301 | cis |
| SCARF2 |  |  |  |  |  |  |  |  |  |  |
| rs9610447 | Diabetic polyneuropathy | 22 | 20768891 | T/C | 0.774 | -0.155 | 0.091 | 0.088 | 0.226 | cis |
| SHANK3 |  |  |  |  |  |  |  |  |  |  |
| rs9616906 | Diabetic polyneuropathy | 22 | 51104680 | A/G | 0.420 | -0.011 | 0.077 | 0.890 | 0.420 | cis |
| MAPK12 |  |  |  |  |  |  |  |  |  |  |
| rs9617128 | Diabetic polyneuropathy | 22 | 50704267 | T/C | 0.802 | -0.080 | 0.094 | 0.393 | 0.198 | cis |
| CEACAM5 |  |  |  |  |  |  |  |  |  |  |
| rs9621 | Diabetic polyneuropathy | 19 | 42231159 | A/G | 0.021 | 0.075 | 0.258 | 0.772 | 0.021 | cis |
| IGLL1 |  |  |  |  |  |  |  |  |  |  |
| rs9624216 | Diabetic polyneuropathy | 22 | 23922552 | A/G | 0.056 | 0.001 | 0.171 | 0.994 | 0.056 | cis |
| RARRES2 |  |  |  |  |  |  |  |  |  |  |
| rs9640161 | Diabetic polyneuropathy | 7 | 150045910 | A/C | 0.414 | 0.017 | 0.077 | 0.821 | 0.414 | cis |
| ST3GAL1 |  |  |  |  |  |  |  |  |  |  |
| rs9643300 | Diabetic polyneuropathy | 8 | 134503148 | T/C | 0.609 | 0.016 | 0.077 | 0.836 | 0.391 | cis |
| PDCD6 |  |  |  |  |  |  |  |  |  |  |
| rs9654451 | Diabetic polyneuropathy | 5 | 299543 | T/C | 0.091 | -0.169 | 0.132 | 0.200 | 0.091 | cis |
| MANSC4 |  |  |  |  |  |  |  |  |  |  |
| rs9668702 | Diabetic polyneuropathy | 12 | 27919491 | T/G | 0.790 | 0.095 | 0.093 | 0.309 | 0.210 | cis |
| HAPLN1 |  |  |  |  |  |  |  |  |  |  |
| rs975595 | Diabetic polyneuropathy | 5 | 83010095 | A/G | 0.681 | -0.077 | 0.081 | 0.345 | 0.319 | cis |
| MATN4 |  |  |  |  |  |  |  |  |  |  |
| rs9784191 | Diabetic polyneuropathy | 20 | 43939090 | G/A | 0.300 | -0.079 | 0.082 | 0.338 | 0.300 | cis |
| PPIE |  |  |  |  |  |  |  |  |  |  |
| rs9787249 | Diabetic polyneuropathy | 1 | 40212878 | A/G | 0.368 | -0.003 | 0.078 | 0.971 | 0.368 | cis |
| COL4A3BP |  |  |  |  |  |  |  |  |  |  |
| rs9791146 | Diabetic polyneuropathy | 5 | 74740950 | T/C | 0.072 | -0.127 | 0.144 | 0.379 | 0.072 | cis |
| IFNGR2 |  |  |  |  |  |  |  |  |  |  |
| rs9808753 | Diabetic polyneuropathy | 21 | 34787312 | G/A | 0.185 | 0.029 | 0.098 | 0.766 | 0.185 | cis |
| MST1 |  |  |  |  |  |  |  |  |  |  |
| rs9858542 | Diabetic polyneuropathy | 3 | 49701983 | A/G | 0.414 | -0.138 | 0.077 | 0.074 | 0.414 | cis |
| PTX3 |  |  |  |  |  |  |  |  |  |  |
| rs9859018 | Diabetic polyneuropathy | 3 | 157204598 | A/G | 0.265 | -0.045 | 0.085 | 0.602 | 0.265 | cis |
| PDCD6IP |  |  |  |  |  |  |  |  |  |  |
| rs9876986 | Diabetic polyneuropathy | 3 | 33841065 | A/G | 0.763 | 0.026 | 0.089 | 0.771 | 0.237 | cis |
| NTN1 |  |  |  |  |  |  |  |  |  |  |
| rs9897200 | Diabetic polyneuropathy | 17 | 8985510 | T/G | 0.168 | 0.002 | 0.101 | 0.988 | 0.168 | cis |
| CD68 |  |  |  |  |  |  |  |  |  |  |
| rs9901675 | Diabetic polyneuropathy | 17 | 7484812 | A/G | 0.030 | -0.127 | 0.218 | 0.560 | 0.030 | cis |
| PKP2 |  |  |  |  |  |  |  |  |  |  |
| rs9919823 | Diabetic polyneuropathy | 12 | 32974611 | T/C | 0.942 | -0.246 | 0.166 | 0.139 | 0.058 | cis |
| HDGFRP3 |  |  |  |  |  |  |  |  |  |  |
| rs9919979 | Diabetic polyneuropathy | 15 | 83748799 | A/C | 0.078 | 0.135 | 0.141 | 0.336 | 0.078 | cis |
| ZG16B |  |  |  |  |  |  |  |  |  |  |
| rs9925556 | Diabetic polyneuropathy | 16 | 2880105 | C/T | 0.354 | 0.109 | 0.079 | 0.170 | 0.354 | cis |
| MLYCD |  |  |  |  |  |  |  |  |  |  |
| rs9931691 | Diabetic polyneuropathy | 16 | 83936496 | T/C | 0.857 | 0.028 | 0.108 | 0.794 | 0.143 | cis |
| FGFBP1 |  |  |  |  |  |  |  |  |  |  |
| rs9968276 | Diabetic polyneuropathy | 4 | 15949551 | A/G | 0.271 | 0.199 | 0.086 | 0.020 | 0.271 | cis |
| BAIAP2 |  |  |  |  |  |  |  |  |  |  |
| rs9989483 | Diabetic polyneuropathy | 17 | 78971536 | T/C | 0.240 | 0.010 | 0.089 | 0.913 | 0.240 | cis |
| CYTL1 |  |  |  |  |  |  |  |  |  |  |
| rs9998211 | Diabetic polyneuropathy | 4 | 5026641 | C/A | 0.119 | 0.033 | 0.116 | 0.778 | 0.119 | cis |

Note: SNP, Single Nucleotide Polymorphism; CHR, Chromosome; POS, Position; EA, Effect Allele; OA, Other Allele; EAF, Effect Allele Frequency; β, Beta Value; SE, Standard Error of β; P, P-value; MAF, Minor Allele Frequency; *, SNPs are sorted in descending order of β (effect allele value).
