# Supplementary material for: Identification of potential drug targets for diabetic polyneuropathy through Mendelian randomization analysis
Source: Cell Biosci. 2024 Dec 5;14:147. doi: 10.1186/s13578-024-01323-4 (PMC11619124; doi:10.1186/s13578-024-01323-4)
Supplement: Supplementary file 3 — Supplementary Material 3: Figure S3. CD14: (A) Forest plot, (B) Scatter plot, and (C) Funnel plot. [file 13578_2024_1323_MOESM3_ESM.docx]

**Plasma Protein cis- and trans-pQTLs as Genetic Instruments for Mendelian Randomization Analysis**

| **SNP** | **Outcome** | **CHR** | **POS** | **EA/OA** | **EAF** | **β*** | **SE** | **P** | **MAF** | **Cis_trans** |
| --- | --- | --- | --- | --- | --- | --- | --- | --- | --- | --- |
| SPOCK1 |  |  |  |  |  |  |  |  |  |  |
| rs149440923 | Diabetic polyneuropathy | 11 | 71708649 | G/A | 0.008 | 0.486 | 0.429 | 0.258 | 0.008 | trans |
| rs4655581 | Diabetic polyneuropathy | 1 | 68720435 | T/C | 0.129 | 0.044 | 0.112 | 0.692 | 0.129 | trans |
| rs77736461 | Diabetic polyneuropathy | 2 | 18680765 | T/C | 0.252 | 0.029 | 0.087 | 0.737 | 0.252 | trans |
| rs62155324 | Diabetic polyneuropathy | 2 | 67768340 | A/C | 0.055 | 0.016 | 0.168 | 0.926 | 0.055 | trans |
| rs2505507 | Diabetic polyneuropathy | 10 | 43644824 | T/C | 0.301 | -0.005 | 0.083 | 0.951 | 0.301 | trans |
| rs4835737 | Diabetic polyneuropathy | 5 | 136895064 | G/A | 0.357 | -0.024 | 0.079 | 0.759 | 0.357 | cis |
| rs7756288 | Diabetic polyneuropathy | 6 | 15110712 | C/T | 0.254 | -0.044 | 0.087 | 0.615 | 0.254 | trans |
| rs36100715 | Diabetic polyneuropathy | 12 | 129346208 | G/T | 0.119 | -0.129 | 0.119 | 0.278 | 0.119 | trans |
| FCRL1 |  |  |  |  |  |  |  |  |  |  |
| rs180798958 | Diabetic polyneuropathy | 5 | 143188976 | A/G | 0.025 | 0.419 | 0.247 | 0.089 | 0.025 | trans |
| rs77542162 | Diabetic polyneuropathy | 17 | 67081278 | A/G | 0.993 | 0.407 | 0.465 | 0.382 | 0.007 | trans |
| rs76428106 | Diabetic polyneuropathy | 13 | 28604007 | C/T | 0.012 | 0.322 | 0.347 | 0.353 | 0.012 | trans |
| rs73926277 | Diabetic polyneuropathy | 19 | 33742626 | T/C | 0.342 | 0.157 | 0.080 | 0.049 | 0.342 | trans |
| rs34762068 | Diabetic polyneuropathy | 3 | 128325763 | A/G | 0.302 | 0.105 | 0.082 | 0.203 | 0.302 | trans |
| rs2009581 | Diabetic polyneuropathy | 2 | 111807677 | A/G | 0.264 | 0.088 | 0.086 | 0.302 | 0.264 | trans |
| rs73348121 | Diabetic polyneuropathy | 7 | 50411987 | C/T | 0.030 | 0.061 | 0.223 | 0.785 | 0.030 | trans |
| rs1035606 | Diabetic polyneuropathy | 12 | 29509450 | T/C | 0.295 | 0.036 | 0.083 | 0.662 | 0.295 | trans |
| rs1433577 | Diabetic polyneuropathy | 8 | 130595881 | A/G | 0.266 | 0.020 | 0.085 | 0.819 | 0.266 | trans |
| rs4245597 | Diabetic polyneuropathy | 10 | 63725942 | A/G | 0.752 | 0.018 | 0.087 | 0.835 | 0.248 | trans |
| rs17361097 | Diabetic polyneuropathy | 4 | 153001219 | A/G | 0.398 | -0.045 | 0.077 | 0.557 | 0.398 | trans |
| rs10002742 | Diabetic polyneuropathy | 4 | 141020165 | A/G | 0.419 | -0.073 | 0.078 | 0.349 | 0.419 | trans |
| rs5001409 | Diabetic polyneuropathy | 3 | 186735690 | C/A | 0.416 | -0.098 | 0.077 | 0.201 | 0.416 | trans |
| rs12784975 | Diabetic polyneuropathy | 10 | 98380137 | C/T | 0.116 | -0.128 | 0.118 | 0.278 | 0.116 | trans |
| rs112824187 | Diabetic polyneuropathy | 5 | 158265308 | A/G | 0.056 | -0.219 | 0.167 | 0.188 | 0.056 | trans |
| DRAXIN |  |  |  |  |  |  |  |  |  |  |
| rs180798958 | Diabetic polyneuropathy | 5 | 143188976 | A/G | 0.025 | 0.419 | 0.247 | 0.089 | 0.025 | trans |
| rs7508294 | Diabetic polyneuropathy | 19 | 33743697 | A/G | 0.342 | 0.158 | 0.080 | 0.048 | 0.342 | trans |
| rs10107630 | Diabetic polyneuropathy | 8 | 130603635 | T/C | 0.581 | 0.031 | 0.076 | 0.684 | 0.419 | trans |
| rs9483788 | Diabetic polyneuropathy | 6 | 135435501 | C/T | 0.314 | -0.004 | 0.082 | 0.958 | 0.314 | trans |
| rs6679089 | Diabetic polyneuropathy | 1 | 11752140 | A/G | 0.044 | -0.032 | 0.188 | 0.863 | 0.044 | cis |
| rs681343 | Diabetic polyneuropathy | 19 | 49206462 | T/C | 0.375 | -0.045 | 0.078 | 0.568 | 0.375 | trans |
| rs17361097 | Diabetic polyneuropathy | 4 | 153001219 | A/G | 0.398 | -0.045 | 0.077 | 0.557 | 0.398 | trans |
| rs10002742 | Diabetic polyneuropathy | 4 | 141020165 | A/G | 0.419 | -0.073 | 0.078 | 0.349 | 0.419 | trans |
| rs11410806 | Diabetic polyneuropathy | 15 | 81844771 | TA/T | 0.164 | -0.138 | 0.102 | 0.177 | 0.164 | trans |
| rs4704963 | Diabetic polyneuropathy | 5 | 158247378 | C/T | 0.056 | -0.220 | 0.166 | 0.187 | 0.056 | trans |
| rs76529516 | Diabetic polyneuropathy | 2 | 60583370 | G/A | 0.012 | -0.226 | 0.343 | 0.511 | 0.012 | trans |
| rs62621812 | Diabetic polyneuropathy | 7 | 127015083 | A/G | 0.043 | -0.307 | 0.191 | 0.108 | 0.043 | trans |
| PRSS27 |  |  |  |  |  |  |  |  |  |  |
| rs79755767 | Diabetic polyneuropathy | 12 | 54698408 | A/G | 0.095 | 0.038 | 0.128 | 0.767 | 0.095 | trans |
| rs112972631 | Diabetic polyneuropathy | 10 | 104358178 | T/G | 0.196 | 0.034 | 0.095 | 0.723 | 0.196 | trans |
| rs635634 | Diabetic polyneuropathy | 9 | 136155000 | T/C | 0.200 | 0.020 | 0.095 | 0.833 | 0.200 | trans |
| rs71386687 | Diabetic polyneuropathy | 16 | 2767894 | T/G | 0.117 | 0.007 | 0.121 | 0.957 | 0.117 | cis |
| rs3824458 | Diabetic polyneuropathy | 9 | 33144809 | T/C | 0.259 | -0.003 | 0.086 | 0.974 | 0.259 | trans |
| rs141129381 | Diabetic polyneuropathy | 9 | 100691900 | TG/T | 0.398 | -0.025 | 0.077 | 0.751 | 0.398 | trans |
| rs681343 | Diabetic polyneuropathy | 19 | 49206462 | T/C | 0.375 | -0.045 | 0.078 | 0.568 | 0.375 | trans |
| rs1036332 | Diabetic polyneuropathy | 1 | 199012478 | A/C | 0.278 | -0.051 | 0.084 | 0.542 | 0.278 | trans |
| rs708686 | Diabetic polyneuropathy | 19 | 5840619 | T/C | 0.334 | -0.105 | 0.081 | 0.194 | 0.334 | trans |
| rs587935 | Diabetic polyneuropathy | 21 | 40910740 | T/C | 0.793 | -0.105 | 0.093 | 0.259 | 0.207 | trans |
| rs11220477 | Diabetic polyneuropathy | 11 | 126275402 | T/C | 0.046 | -0.114 | 0.183 | 0.534 | 0.046 | trans |
| rs1654439 | Diabetic polyneuropathy | 19 | 55553647 | T/G | 0.132 | -0.182 | 0.111 | 0.102 | 0.132 | cis |
| CR2 |  |  |  |  |  |  |  |  |  |  |
| rs12554848 | Diabetic polyneuropathy | 9 | 36981822 | C/T | 0.191 | 0.089 | 0.096 | 0.355 | 0.191 | trans |
| rs1528149 | Diabetic polyneuropathy | 7 | 16107358 | T/C | 0.307 | 0.060 | 0.081 | 0.460 | 0.307 | trans |
| rs114697502 | Diabetic polyneuropathy | 12 | 94677559 | T/C | 0.057 | 0.034 | 0.161 | 0.832 | 0.057 | trans |
| rs11238349 | Diabetic polyneuropathy | 7 | 55156071 | A/G | 0.363 | -0.002 | 0.079 | 0.982 | 0.363 | trans |
| rs4656100 | Diabetic polyneuropathy | 1 | 86917944 | C/T | 0.647 | -0.004 | 0.079 | 0.965 | 0.353 | trans |
| rs10774624 | Diabetic polyneuropathy | 12 | 111833788 | A/G | 0.597 | -0.015 | 0.077 | 0.847 | 0.403 | trans |
| rs35374520 | Diabetic polyneuropathy | 22 | 19851149 | G/A | 0.098 | -0.026 | 0.126 | 0.835 | 0.098 | trans |
| rs4055121 | Diabetic polyneuropathy | 11 | 126232337 | T/C | 0.153 | -0.048 | 0.106 | 0.651 | 0.153 | trans |
| rs10012161 | Diabetic polyneuropathy | 4 | 177966780 | G/A | 0.881 | -0.060 | 0.116 | 0.604 | 0.119 | trans |
| rs7228151 | Diabetic polyneuropathy | 18 | 57181694 | C/T | 0.264 | -0.074 | 0.086 | 0.390 | 0.264 | trans |
| rs61821111 | Diabetic polyneuropathy | 1 | 207595899 | C/T | 0.070 | -0.075 | 0.146 | 0.608 | 0.070 | cis |
| rs5001409 | Diabetic polyneuropathy | 3 | 186735690 | C/A | 0.416 | -0.098 | 0.077 | 0.201 | 0.416 | trans |
| REG3G |  |  |  |  |  |  |  |  |  |  |
| rs369967 | Diabetic polyneuropathy | 2 | 79253150 | G/A | 0.285 | -0.035 | 0.084 | 0.673 | 0.285 | cis |
| rs10013413 | Diabetic polyneuropathy | 4 | 148982278 | C/T | 0.244 | -0.174 | 0.089 | 0.050 | 0.244 | trans |
| CPA1 |  |  |  |  |  |  |  |  |  |  |
| rs10901250 | Diabetic polyneuropathy | 9 | 136121626 | A/G | 0.223 | 0.217 | 0.091 | 0.018 | 0.223 | trans |
| rs2641348 | Diabetic polyneuropathy | 1 | 120437884 | G/A | 0.149 | 0.192 | 0.106 | 0.070 | 0.149 | trans |
| rs1673931 | Diabetic polyneuropathy | 16 | 88976477 | C/T | 0.273 | 0.097 | 0.085 | 0.250 | 0.273 | trans |
| rs686056 | Diabetic polyneuropathy | 11 | 100646689 | A/G | 0.292 | 0.039 | 0.083 | 0.640 | 0.292 | trans |
| rs13226219 | Diabetic polyneuropathy | 7 | 130019491 | C/T | 0.105 | 0.010 | 0.122 | 0.936 | 0.105 | cis |
| rs8131986 | Diabetic polyneuropathy | 21 | 39459318 | C/A | 0.590 | 0.007 | 0.077 | 0.926 | 0.410 | trans |
| rs56278466 | Diabetic polyneuropathy | 10 | 17875857 | G/T | 0.581 | -0.003 | 0.077 | 0.970 | 0.419 | trans |
| rs72802342 | Diabetic polyneuropathy | 16 | 75234872 | A/C | 0.086 | -0.032 | 0.135 | 0.814 | 0.086 | trans |
| rs10014383 | Diabetic polyneuropathy | 4 | 110848319 | C/T | 0.301 | -0.047 | 0.082 | 0.567 | 0.301 | trans |
| rs17138478 | Diabetic polyneuropathy | 17 | 36073320 | A/C | 0.158 | -0.052 | 0.105 | 0.622 | 0.158 | trans |
| rs11724862 | Diabetic polyneuropathy | 4 | 186572503 | C/T | 0.251 | -0.067 | 0.089 | 0.450 | 0.251 | trans |
| rs76734539 | Diabetic polyneuropathy | 12 | 133108555 | A/G | 0.127 | -0.094 | 0.114 | 0.408 | 0.127 | trans |
| rs28456 | Diabetic polyneuropathy | 11 | 61589481 | G/A | 0.390 | -0.104 | 0.077 | 0.178 | 0.390 | trans |
| HBEGF |  |  |  |  |  |  |  |  |  |  |
| rs80137017 | Diabetic polyneuropathy | 10 | 71237382 | T/C | 0.107 | 0.201 | 0.121 | 0.097 | 0.107 | trans |
| rs11553699 | Diabetic polyneuropathy | 12 | 122216910 | G/A | 0.107 | 0.174 | 0.123 | 0.158 | 0.107 | trans |
| rs1654425 | Diabetic polyneuropathy | 19 | 55538980 | C/T | 0.879 | 0.126 | 0.115 | 0.272 | 0.121 | trans |
| rs489298 | Diabetic polyneuropathy | 12 | 3239165 | T/C | 0.373 | 0.082 | 0.078 | 0.292 | 0.373 | trans |
| rs62396356 | Diabetic polyneuropathy | 6 | 41167763 | G/A | 0.092 | 0.079 | 0.131 | 0.547 | 0.092 | trans |
| rs1354034 | Diabetic polyneuropathy | 3 | 56849749 | C/T | 0.707 | 0.052 | 0.083 | 0.532 | 0.293 | trans |
| rs10761737 | Diabetic polyneuropathy | 10 | 65052205 | C/T | 0.385 | -0.004 | 0.078 | 0.958 | 0.385 | trans |
| rs12445050 | Diabetic polyneuropathy | 16 | 81870969 | T/C | 0.130 | -0.033 | 0.113 | 0.769 | 0.130 | trans |
| rs3811444 | Diabetic polyneuropathy | 1 | 248039451 | T/C | 0.346 | -0.037 | 0.080 | 0.645 | 0.346 | trans |
| rs3790176 | Diabetic polyneuropathy | 20 | 19261922 | A/G | 0.290 | -0.058 | 0.083 | 0.487 | 0.290 | trans |
| rs78909033 | Diabetic polyneuropathy | 2 | 241510903 | A/G | 0.131 | -0.058 | 0.112 | 0.603 | 0.131 | trans |
| rs6961069 | Diabetic polyneuropathy | 7 | 80218961 | T/C | 0.417 | -0.076 | 0.077 | 0.324 | 0.417 | trans |
| rs467369 | Diabetic polyneuropathy | 9 | 136905765 | T/C | 0.703 | -0.091 | 0.083 | 0.272 | 0.297 | trans |
| rs116905185 | Diabetic polyneuropathy | 20 | 49221303 | C/T | 0.007 | -0.811 | 0.473 | 0.086 | 0.007 | trans |
| EGF |  |  |  |  |  |  |  |  |  |  |
| rs12445050 | Diabetic polyneuropathy | 16 | 81870969 | T/C | 0.130 | -0.033 | 0.113 | 0.769 | 0.130 | trans |
| rs3831508 | Diabetic polyneuropathy | 4 | 110837903 | GT/G | 0.368 | -0.055 | 0.078 | 0.483 | 0.368 | cis |
| rs6081565 | Diabetic polyneuropathy | 20 | 19287904 | A/G | 0.281 | -0.059 | 0.084 | 0.479 | 0.281 | trans |
| rs6961069 | Diabetic polyneuropathy | 7 | 80218961 | T/C | 0.417 | -0.076 | 0.077 | 0.324 | 0.417 | trans |
| rs892090 | Diabetic polyneuropathy | 19 | 55539072 | T/G | 0.121 | -0.126 | 0.115 | 0.272 | 0.121 | trans |
| TSHB |  |  |  |  |  |  |  |  |  |  |
| rs334713 | Diabetic polyneuropathy | 1 | 61622287 | C/A | 0.980 | 0.281 | 0.276 | 0.309 | 0.020 | trans |
| rs200066768 | Diabetic polyneuropathy | 15 | 49728877 | AT/A | 0.248 | 0.124 | 0.087 | 0.158 | 0.248 | trans |
| rs1203944 | Diabetic polyneuropathy | 20 | 22596879 | C/T | 0.792 | 0.023 | 0.093 | 0.808 | 0.208 | trans |
| rs9472136 | Diabetic polyneuropathy | 6 | 43810021 | T/C | 0.398 | 0.008 | 0.077 | 0.918 | 0.398 | trans |
| rs12068854 | Diabetic polyneuropathy | 1 | 108409665 | C/A | 0.120 | 0.006 | 0.117 | 0.958 | 0.120 | trans |
| rs2046045 | Diabetic polyneuropathy | 5 | 76535811 | G/T | 0.413 | -0.028 | 0.077 | 0.714 | 0.413 | trans |
| rs2712172 | Diabetic polyneuropathy | 2 | 217620733 | A/G | 0.372 | -0.082 | 0.079 | 0.294 | 0.372 | trans |
| rs116909374 | Diabetic polyneuropathy | 14 | 36738361 | T/C | 0.031 | -0.134 | 0.219 | 0.541 | 0.031 | trans |
| rs61938844 | Diabetic polyneuropathy | 12 | 96583858 | A/G | 0.009 | -0.269 | 0.406 | 0.508 | 0.009 | trans |
| NPS |  |  |  |  |  |  |  |  |  |  |
| rs10038285 | Diabetic polyneuropathy | 5 | 42689540 | A/G | 0.708 | 0.082 | 0.083 | 0.324 | 0.292 | trans |
| SFTPA2 |  |  |  |  |  |  |  |  |  |  |
| rs117981338 | Diabetic polyneuropathy | 17 | 71088843 | T/C | 0.019 | 0.185 | 0.289 | 0.523 | 0.019 | trans |
| rs7962469 | Diabetic polyneuropathy | 12 | 52348259 | A/G | 0.318 | 0.022 | 0.081 | 0.789 | 0.318 | trans |
| PTH |  |  |  |  |  |  |  |  |  |  |
| rs219771 | Diabetic polyneuropathy | 21 | 37835501 | T/C | 0.215 | 0.099 | 0.092 | 0.279 | 0.215 | trans |
| rs10051765 | Diabetic polyneuropathy | 5 | 176799992 | C/T | 0.401 | -0.015 | 0.077 | 0.846 | 0.401 | trans |
| PDGFA |  |  |  |  |  |  |  |  |  |  |
| rs1654425 | Diabetic polyneuropathy | 19 | 55538980 | C/T | 0.879 | 0.126 | 0.115 | 0.272 | 0.121 | trans |
| rs1354034 | Diabetic polyneuropathy | 3 | 56849749 | C/T | 0.707 | 0.052 | 0.083 | 0.532 | 0.293 | trans |
| rs2278668 | Diabetic polyneuropathy | 3 | 122835232 | C/T | 0.619 | -0.009 | 0.078 | 0.907 | 0.381 | trans |
| rs12445050 | Diabetic polyneuropathy | 16 | 81870969 | T/C | 0.130 | -0.033 | 0.113 | 0.769 | 0.130 | trans |
| rs34377578 | Diabetic polyneuropathy | 10 | 104336426 | A/C | 0.804 | -0.034 | 0.095 | 0.723 | 0.196 | trans |
| rs3811444 | Diabetic polyneuropathy | 1 | 248039451 | T/C | 0.346 | -0.037 | 0.080 | 0.645 | 0.346 | trans |
| rs10058074 | Diabetic polyneuropathy | 5 | 131686146 | A/G | 0.341 | -0.056 | 0.080 | 0.483 | 0.341 | trans |
| rs3827978 | Diabetic polyneuropathy | 20 | 19281291 | T/C | 0.281 | -0.059 | 0.084 | 0.479 | 0.281 | trans |
| rs6961069 | Diabetic polyneuropathy | 7 | 80218961 | T/C | 0.417 | -0.076 | 0.077 | 0.324 | 0.417 | trans |
| rs2759391 | Diabetic polyneuropathy | 6 | 163864226 | C/A | 0.275 | -0.093 | 0.084 | 0.270 | 0.275 | trans |
| rs6671171 | Diabetic polyneuropathy | 1 | 156871318 | A/G | 0.129 | -0.105 | 0.113 | 0.355 | 0.129 | trans |
| rs7618405 | Diabetic polyneuropathy | 3 | 18250509 | A/C | 0.211 | -0.178 | 0.093 | 0.055 | 0.211 | trans |
| ANGPT1 |  |  |  |  |  |  |  |  |  |  |
| rs1654425 | Diabetic polyneuropathy | 19 | 55538980 | C/T | 0.879 | 0.126 | 0.115 | 0.272 | 0.121 | trans |
| rs9410207 | Diabetic polyneuropathy | 9 | 91404799 | C/T | 0.032 | 0.091 | 0.211 | 0.666 | 0.032 | trans |
| rs1354034 | Diabetic polyneuropathy | 3 | 56849749 | C/T | 0.707 | 0.052 | 0.083 | 0.532 | 0.293 | trans |
| rs3792366 | Diabetic polyneuropathy | 3 | 122839876 | A/G | 0.620 | -0.008 | 0.078 | 0.923 | 0.380 | trans |
| rs12445050 | Diabetic polyneuropathy | 16 | 81870969 | T/C | 0.130 | -0.033 | 0.113 | 0.769 | 0.130 | trans |
| rs4759076 | Diabetic polyneuropathy | 12 | 54729872 | C/T | 0.581 | -0.046 | 0.077 | 0.546 | 0.419 | trans |
| rs10058074 | Diabetic polyneuropathy | 5 | 131686146 | A/G | 0.341 | -0.056 | 0.080 | 0.483 | 0.341 | trans |
| rs3827978 | Diabetic polyneuropathy | 20 | 19281291 | T/C | 0.281 | -0.059 | 0.084 | 0.479 | 0.281 | trans |
| rs6961069 | Diabetic polyneuropathy | 7 | 80218961 | T/C | 0.417 | -0.076 | 0.077 | 0.324 | 0.417 | trans |
| rs12041331 | Diabetic polyneuropathy | 1 | 156869714 | A/G | 0.054 | -0.103 | 0.168 | 0.541 | 0.054 | trans |
| rs7618405 | Diabetic polyneuropathy | 3 | 18250509 | A/C | 0.211 | -0.178 | 0.093 | 0.055 | 0.211 | trans |
| rs13412535 | Diabetic polyneuropathy | 2 | 224874874 | A/G | 0.205 | -0.204 | 0.095 | 0.032 | 0.205 | trans |
| rs61978213 | Diabetic polyneuropathy | 14 | 70653758 | A/G | 0.018 | -0.274 | 0.293 | 0.350 | 0.018 | trans |
| IL5RA |  |  |  |  |  |  |  |  |  |  |
| rs6810926 | Diabetic polyneuropathy | 4 | 146968852 | G/A | 0.077 | 0.233 | 0.139 | 0.094 | 0.077 | trans |
| rs2511713 | Diabetic polyneuropathy | 8 | 103577865 | G/A | 0.290 | 0.063 | 0.083 | 0.453 | 0.290 | trans |
| rs66867810 | Diabetic polyneuropathy | 6 | 41940886 | A/G | 0.309 | 0.040 | 0.082 | 0.629 | 0.309 | trans |
| rs11085015 | Diabetic polyneuropathy | 19 | 3369572 | G/T | 0.801 | 0.035 | 0.097 | 0.718 | 0.199 | trans |
| rs968567 | Diabetic polyneuropathy | 11 | 61595564 | T/C | 0.109 | 0.017 | 0.120 | 0.888 | 0.109 | trans |
| rs9869437 | Diabetic polyneuropathy | 3 | 196228360 | A/C | 0.322 | 0.015 | 0.081 | 0.857 | 0.322 | trans |
| rs3803800 | Diabetic polyneuropathy | 17 | 7462969 | G/A | 0.755 | 0.012 | 0.088 | 0.894 | 0.245 | trans |
| rs7705189 | Diabetic polyneuropathy | 5 | 131623358 | G/A | 0.312 | 0.002 | 0.082 | 0.978 | 0.312 | trans |
| rs12880641 | Diabetic polyneuropathy | 14 | 103251452 | G/T | 0.626 | 0.000 | 0.078 | 0.998 | 0.374 | trans |
| rs78740585 | Diabetic polyneuropathy | 7 | 150944302 | A/G | 0.116 | -0.010 | 0.117 | 0.932 | 0.116 | trans |
| rs12342831 | Diabetic polyneuropathy | 9 | 33124872 | C/T | 0.216 | -0.016 | 0.091 | 0.863 | 0.216 | trans |
| rs10896045 | Diabetic polyneuropathy | 11 | 65555524 | G/A | 0.728 | -0.045 | 0.085 | 0.593 | 0.272 | trans |
| rs4794063 | Diabetic polyneuropathy | 17 | 45804494 | T/C | 0.292 | -0.049 | 0.083 | 0.554 | 0.292 | trans |
| rs9819371 | Diabetic polyneuropathy | 3 | 141206800 | T/C | 0.052 | -0.103 | 0.173 | 0.552 | 0.052 | trans |
| rs34562254 | Diabetic polyneuropathy | 17 | 16842991 | A/G | 0.100 | -0.145 | 0.126 | 0.247 | 0.100 | trans |
| rs188468174 | Diabetic polyneuropathy | 1 | 25291697 | T/C | 0.004 | -0.166 | 0.540 | 0.758 | 0.004 | trans |
| rs7797255 | Diabetic polyneuropathy | 7 | 50351604 | G/A | 0.356 | -0.184 | 0.079 | 0.021 | 0.356 | trans |
| rs77400868 | Diabetic polyneuropathy | 3 | 3150964 | G/A | 0.075 | -0.313 | 0.145 | 0.031 | 0.075 | cis |
| TNFSF8 |  |  |  |  |  |  |  |  |  |  |
| rs550057 | Diabetic polyneuropathy | 9 | 136146597 | T/C | 0.302 | 0.128 | 0.082 | 0.121 | 0.302 | trans |
| rs37453 | Diabetic polyneuropathy | 1 | 44295047 | A/G | 0.639 | 0.089 | 0.079 | 0.258 | 0.361 | trans |
| rs1006026 | Diabetic polyneuropathy | 9 | 117691270 | A/G | 0.583 | 0.017 | 0.077 | 0.826 | 0.417 | cis |
| rs55714927 | Diabetic polyneuropathy | 17 | 7080316 | T/C | 0.264 | -0.118 | 0.087 | 0.177 | 0.264 | cis |
| LEAP2 |  |  |  |  |  |  |  |  |  |  |
| rs113414093 | Diabetic polyneuropathy | 2 | 219859171 | A/G | 0.085 | 0.149 | 0.137 | 0.274 | 0.085 | trans |
| rs2954038 | Diabetic polyneuropathy | 8 | 126507389 | C/A | 0.259 | 0.070 | 0.086 | 0.413 | 0.259 | trans |
| rs12515756 | Diabetic polyneuropathy | 5 | 132238880 | C/T | 0.137 | -0.076 | 0.109 | 0.486 | 0.137 | cis |
| rs1260326 | Diabetic polyneuropathy | 2 | 27730940 | T/C | 0.351 | -0.100 | 0.079 | 0.208 | 0.351 | trans |
| OLFML3 |  |  |  |  |  |  |  |  |  |  |
| rs56254331 | Diabetic polyneuropathy | 19 | 41826020 | C/A | 0.222 | 0.047 | 0.092 | 0.605 | 0.222 | trans |
| rs760715 | Diabetic polyneuropathy | 22 | 39862343 | C/T | 0.327 | -0.010 | 0.081 | 0.906 | 0.327 | trans |
| rs4381184 | Diabetic polyneuropathy | 1 | 114489769 | A/C | 0.280 | -0.041 | 0.084 | 0.630 | 0.280 | cis |
| rs7951028 | Diabetic polyneuropathy | 11 | 126238394 | A/G | 0.184 | -0.044 | 0.099 | 0.652 | 0.184 | trans |
| rs61830291 | Diabetic polyneuropathy | 1 | 221001142 | A/C | 0.885 | -0.118 | 0.119 | 0.320 | 0.115 | trans |
| rs77262773 | Diabetic polyneuropathy | 17 | 67249711 | T/C | 0.007 | -0.544 | 0.464 | 0.241 | 0.007 | trans |
| CD302 |  |  |  |  |  |  |  |  |  |  |
| rs10073754 | Diabetic polyneuropathy | 5 | 179306517 | G/A | 0.125 | -0.024 | 0.114 | 0.831 | 0.125 | trans |
| rs9302635 | Diabetic polyneuropathy | 16 | 72144174 | C/T | 0.174 | -0.069 | 0.099 | 0.482 | 0.174 | trans |
| rs7310409 | Diabetic polyneuropathy | 12 | 121424861 | G/A | 0.584 | -0.090 | 0.077 | 0.244 | 0.416 | trans |
| rs2556106 | Diabetic polyneuropathy | 2 | 160654524 | T/G | 0.908 | -0.094 | 0.131 | 0.475 | 0.092 | cis |
| rs8178824 | Diabetic polyneuropathy | 17 | 64224775 | T/C | 0.009 | -0.140 | 0.389 | 0.718 | 0.009 | trans |
| NT5E |  |  |  |  |  |  |  |  |  |  |
| rs13107325 | Diabetic polyneuropathy | 4 | 103188709 | T/C | 0.014 | 0.106 | 0.309 | 0.731 | 0.014 | trans |
| rs601338 | Diabetic polyneuropathy | 19 | 49206674 | G/A | 0.626 | 0.044 | 0.078 | 0.571 | 0.375 | trans |
| rs10075805 | Diabetic polyneuropathy | 5 | 31021358 | G/A | 0.322 | 0.034 | 0.081 | 0.678 | 0.322 | trans |
| rs12373325 | Diabetic polyneuropathy | 18 | 56087648 | C/T | 0.767 | 0.015 | 0.090 | 0.866 | 0.233 | trans |
| rs4835265 | Diabetic polyneuropathy | 4 | 146821410 | A/C | 0.172 | -0.047 | 0.101 | 0.641 | 0.172 | trans |
| rs1497406 | Diabetic polyneuropathy | 1 | 16505320 | G/A | 0.682 | -0.071 | 0.081 | 0.379 | 0.318 | trans |
| rs2229523 | Diabetic polyneuropathy | 6 | 86199233 | G/A | 0.632 | -0.134 | 0.078 | 0.086 | 0.368 | cis |
| rs7429191 | Diabetic polyneuropathy | 3 | 149113080 | A/G | 0.752 | -0.144 | 0.087 | 0.099 | 0.248 | trans |
| MME |  |  |  |  |  |  |  |  |  |  |
| rs17674290 | Diabetic polyneuropathy | 12 | 20998109 | G/T | 0.109 | 0.102 | 0.120 | 0.394 | 0.109 | trans |
| rs58659609 | Diabetic polyneuropathy | 19 | 3482459 | A/C | 0.188 | 0.051 | 0.096 | 0.597 | 0.188 | trans |
| rs10075805 | Diabetic polyneuropathy | 5 | 31021358 | G/A | 0.322 | 0.034 | 0.081 | 0.678 | 0.322 | trans |
| rs79837905 | Diabetic polyneuropathy | 3 | 154785591 | G/A | 0.065 | 0.021 | 0.154 | 0.893 | 0.065 | cis |
| rs12373325 | Diabetic polyneuropathy | 18 | 56087648 | C/T | 0.767 | 0.015 | 0.090 | 0.866 | 0.233 | trans |
| rs4687657 | Diabetic polyneuropathy | 3 | 52852538 | T/G | 0.287 | -0.028 | 0.083 | 0.741 | 0.287 | trans |
| rs4835265 | Diabetic polyneuropathy | 4 | 146821410 | A/C | 0.172 | -0.047 | 0.101 | 0.641 | 0.172 | trans |
| rs112875651 | Diabetic polyneuropathy | 8 | 126506694 | A/G | 0.387 | -0.058 | 0.078 | 0.456 | 0.387 | trans |
| rs1497406 | Diabetic polyneuropathy | 1 | 16505320 | G/A | 0.682 | -0.071 | 0.081 | 0.379 | 0.318 | trans |
| rs59643720 | Diabetic polyneuropathy | 14 | 103564807 | C/A | 0.232 | -0.092 | 0.090 | 0.304 | 0.232 | trans |
| rs1650146 | Diabetic polyneuropathy | 10 | 79688208 | G/A | 0.587 | -0.111 | 0.077 | 0.146 | 0.413 | trans |
| rs12169946 | Diabetic polyneuropathy | 22 | 18468105 | G/A | 0.227 | -0.156 | 0.090 | 0.083 | 0.227 | trans |
| ADAMTSL2 |  |  |  |  |  |  |  |  |  |  |
| rs73045269 | Diabetic polyneuropathy | 19 | 41825191 | T/C | 0.215 | 0.060 | 0.093 | 0.517 | 0.215 | trans |
| rs61830291 | Diabetic polyneuropathy | 1 | 221001142 | A/C | 0.885 | -0.118 | 0.119 | 0.320 | 0.115 | trans |
| rs1008250 | Diabetic polyneuropathy | 9 | 136397834 | A/G | 0.657 | -0.150 | 0.082 | 0.066 | 0.343 | cis |
| GPR37 |  |  |  |  |  |  |  |  |  |  |
| rs10083137 | Diabetic polyneuropathy | 12 | 24198178 | G/A | 0.020 | 0.401 | 0.270 | 0.137 | 0.020 | trans |
| rs780094 | Diabetic polyneuropathy | 2 | 27741237 | C/T | 0.643 | 0.105 | 0.079 | 0.183 | 0.357 | trans |
| rs390082 | Diabetic polyneuropathy | 19 | 45416831 | G/T | 0.075 | 0.079 | 0.142 | 0.579 | 0.075 | trans |
| rs511154 | Diabetic polyneuropathy | 3 | 135950921 | G/A | 0.849 | 0.006 | 0.105 | 0.954 | 0.151 | trans |
| rs13235543 | Diabetic polyneuropathy | 7 | 73013901 | T/C | 0.129 | 0.001 | 0.112 | 0.992 | 0.129 | trans |
| rs4141005 | Diabetic polyneuropathy | 7 | 124128619 | C/A | 0.189 | -0.009 | 0.096 | 0.926 | 0.189 | cis |
| rs112875651 | Diabetic polyneuropathy | 8 | 126506694 | A/G | 0.387 | -0.058 | 0.078 | 0.456 | 0.387 | trans |
| LRIG1 |  |  |  |  |  |  |  |  |  |  |
| rs10083143 | Diabetic polyneuropathy | 12 | 24198568 | C/A | 0.020 | 0.379 | 0.267 | 0.155 | 0.020 | trans |
| rs9372538 | Diabetic polyneuropathy | 6 | 119791212 | T/C | 0.334 | 0.139 | 0.080 | 0.084 | 0.334 | trans |
| rs11045856 | Diabetic polyneuropathy | 12 | 21350689 | G/T | 0.172 | 0.098 | 0.100 | 0.326 | 0.172 | trans |
| rs28929474 | Diabetic polyneuropathy | 14 | 94844947 | T/C | 0.020 | -0.002 | 0.272 | 0.993 | 0.020 | trans |
| rs56278466 | Diabetic polyneuropathy | 10 | 17875857 | G/T | 0.581 | -0.003 | 0.077 | 0.970 | 0.419 | trans |
| rs17616063 | Diabetic polyneuropathy | 16 | 51436882 | G/A | 0.141 | -0.008 | 0.110 | 0.946 | 0.141 | trans |
| rs2306272 | Diabetic polyneuropathy | 3 | 66434643 | C/T | 0.252 | -0.037 | 0.087 | 0.671 | 0.252 | cis |
| rs7979473 | Diabetic polyneuropathy | 12 | 121420260 | G/A | 0.581 | -0.108 | 0.077 | 0.161 | 0.419 | trans |
| PILRB |  |  |  |  |  |  |  |  |  |  |
| rs1859788 | Diabetic polyneuropathy | 7 | 99971834 | G/A | 0.691 | -0.006 | 0.082 | 0.944 | 0.309 | cis |
| TFPI2 |  |  |  |  |  |  |  |  |  |  |
| rs10087526 | Diabetic polyneuropathy | 8 | 55433151 | G/T | 0.226 | 0.177 | 0.090 | 0.050 | 0.226 | trans |
| rs1056523 | Diabetic polyneuropathy | 3 | 126261207 | T/C | 0.287 | 0.064 | 0.083 | 0.441 | 0.287 | trans |
| rs35719208 | Diabetic polyneuropathy | 8 | 105966314 | T/C | 0.225 | 0.035 | 0.091 | 0.701 | 0.225 | trans |
| rs62466701 | Diabetic polyneuropathy | 7 | 93585663 | C/T | 0.084 | -0.054 | 0.139 | 0.695 | 0.084 | cis |
| rs61804208 | Diabetic polyneuropathy | 1 | 161661411 | T/G | 0.118 | -0.094 | 0.119 | 0.433 | 0.118 | trans |
| rs68066031 | Diabetic polyneuropathy | 2 | 224880498 | C/T | 0.204 | -0.200 | 0.095 | 0.036 | 0.204 | trans |
| ARG2 |  |  |  |  |  |  |  |  |  |  |
| rs1008910 | Diabetic polyneuropathy | 22 | 23055440 | A/C | 0.581 | 0.148 | 0.080 | 0.065 | 0.420 | trans |
| rs1354034 | Diabetic polyneuropathy | 3 | 56849749 | C/T | 0.707 | 0.052 | 0.083 | 0.532 | 0.293 | trans |
| rs61990120 | Diabetic polyneuropathy | 14 | 68089420 | A/G | 0.157 | 0.005 | 0.106 | 0.962 | 0.157 | cis |
| rs3748136 | Diabetic polyneuropathy | 8 | 9030160 | A/G | 0.304 | -0.115 | 0.101 | 0.254 | 0.304 | trans |
| CLSPN |  |  |  |  |  |  |  |  |  |  |
| rs1008982 | Diabetic polyneuropathy | 10 | 131445731 | C/T | 0.412 | -0.170 | 0.077 | 0.027 | 0.412 | trans |
| MGMT |  |  |  |  |  |  |  |  |  |  |
| rs1354034 | Diabetic polyneuropathy | 3 | 56849749 | C/T | 0.707 | 0.052 | 0.083 | 0.532 | 0.293 | trans |
| rs1008982 | Diabetic polyneuropathy | 10 | 131445731 | C/T | 0.412 | -0.170 | 0.077 | 0.027 | 0.412 | trans |
| SELPLG |  |  |  |  |  |  |  |  |  |  |
| rs10214273 | Diabetic polyneuropathy | 5 | 35883986 | G/T | 0.335 | 0.056 | 0.080 | 0.482 | 0.335 | trans |
| rs343808 | Diabetic polyneuropathy | 1 | 111330007 | T/C | 0.260 | 0.002 | 0.087 | 0.982 | 0.260 | trans |
| rs10819317 | Diabetic polyneuropathy | 9 | 130668957 | G/A | 0.190 | -0.003 | 0.097 | 0.975 | 0.190 | trans |
| rs10093797 | Diabetic polyneuropathy | 8 | 79572713 | A/C | 0.732 | -0.063 | 0.085 | 0.462 | 0.268 | trans |
| rs2142306 | Diabetic polyneuropathy | 8 | 134470631 | C/T | 0.375 | -0.079 | 0.078 | 0.311 | 0.375 | trans |
| rs7294337 | Diabetic polyneuropathy | 12 | 109012694 | T/C | 0.039 | -0.357 | 0.198 | 0.072 | 0.039 | cis |
| HS3ST3A1 |  |  |  |  |  |  |  |  |  |  |
| rs1354034 | Diabetic polyneuropathy | 3 | 56849749 | C/T | 0.707 | 0.052 | 0.083 | 0.532 | 0.293 | trans |
| rs1009532 | Diabetic polyneuropathy | 10 | 111840725 | A/G | 0.855 | -0.061 | 0.108 | 0.573 | 0.145 | trans |
| CA5B |  |  |  |  |  |  |  |  |  |  |
| rs112657244 | Diabetic polyneuropathy | 17 | 48619290 | A/G | 0.095 | 0.092 | 0.128 | 0.473 | 0.095 | trans |
| rs1009610 | Diabetic polyneuropathy | 22 | 50638953 | A/G | 0.370 | -0.052 | 0.078 | 0.505 | 0.370 | trans |
| C5orf38 |  |  |  |  |  |  |  |  |  |  |
| rs7412 | Diabetic polyneuropathy | 19 | 45412079 | T/C | 0.053 | 0.166 | 0.164 | 0.311 | 0.053 | trans |
| rs10096633 | Diabetic polyneuropathy | 8 | 19830921 | T/C | 0.097 | 0.072 | 0.127 | 0.571 | 0.097 | trans |
| rs1707652 | Diabetic polyneuropathy | 3 | 165478799 | T/C | 0.248 | -0.037 | 0.087 | 0.672 | 0.248 | trans |
| rs2678379 | Diabetic polyneuropathy | 2 | 21226560 | A/G | 0.266 | -0.078 | 0.086 | 0.366 | 0.266 | trans |
| WNT5A |  |  |  |  |  |  |  |  |  |  |
| rs10096633 | Diabetic polyneuropathy | 8 | 19830921 | T/C | 0.097 | 0.072 | 0.127 | 0.571 | 0.097 | trans |
| rs261290 | Diabetic polyneuropathy | 15 | 58678720 | C/T | 0.618 | 0.050 | 0.078 | 0.524 | 0.382 | trans |
| rs673548 | Diabetic polyneuropathy | 2 | 21237544 | A/G | 0.266 | -0.078 | 0.086 | 0.365 | 0.266 | trans |
| rs116843064 | Diabetic polyneuropathy | 19 | 8429323 | A/G | 0.027 | -0.093 | 0.230 | 0.685 | 0.027 | trans |
| rs780093 | Diabetic polyneuropathy | 2 | 27742603 | T/C | 0.353 | -0.111 | 0.079 | 0.163 | 0.353 | trans |
| rs150844304 | Diabetic polyneuropathy | 15 | 43726625 | C/A | 0.005 | -0.223 | 0.528 | 0.673 | 0.005 | trans |
| DEFA1_DEFA1B |  |  |  |  |  |  |  |  |  |  |
| rs10103048 | Diabetic polyneuropathy | 8 | 130602281 | C/A | 0.586 | 0.048 | 0.077 | 0.528 | 0.414 | trans |
| rs4857909 | Diabetic polyneuropathy | 3 | 128300468 | G/A | 0.868 | 0.046 | 0.111 | 0.677 | 0.132 | trans |
| rs210962 | Diabetic polyneuropathy | 6 | 135503785 | T/C | 0.273 | 0.046 | 0.085 | 0.585 | 0.273 | trans |
| rs77547572 | Diabetic polyneuropathy | 5 | 98451375 | A/G | 0.100 | -0.023 | 0.127 | 0.858 | 0.100 | trans |
| rs915125 | Diabetic polyneuropathy | 6 | 82463376 | T/C | 0.306 | -0.045 | 0.082 | 0.589 | 0.306 | trans |
| rs61740288 | Diabetic polyneuropathy | 2 | 71762413 | A/G | 0.022 | -0.114 | 0.268 | 0.669 | 0.022 | trans |
| RBL2 |  |  |  |  |  |  |  |  |  |  |
| rs1056524 | Diabetic polyneuropathy | 3 | 126261202 | A/G | 0.321 | 0.071 | 0.081 | 0.377 | 0.321 | trans |
| rs736408 | Diabetic polyneuropathy | 3 | 52835354 | T/C | 0.382 | -0.012 | 0.078 | 0.883 | 0.382 | trans |
| rs10103148 | Diabetic polyneuropathy | 8 | 18267429 | A/C | 0.817 | -0.130 | 0.098 | 0.181 | 0.183 | trans |
| TNFRSF10C |  |  |  |  |  |  |  |  |  |  |
| rs3014874 | Diabetic polyneuropathy | 1 | 153337943 | A/G | 0.252 | 0.133 | 0.087 | 0.128 | 0.252 | trans |
| rs4760 | Diabetic polyneuropathy | 19 | 44153100 | G/A | 0.168 | 0.087 | 0.103 | 0.396 | 0.168 | trans |
| rs3124753 | Diabetic polyneuropathy | 9 | 136249929 | A/G | 0.390 | 0.033 | 0.078 | 0.675 | 0.390 | trans |
| rs12497115 | Diabetic polyneuropathy | 3 | 47220341 | T/C | 0.233 | 0.026 | 0.090 | 0.771 | 0.233 | trans |
| rs1010366 | Diabetic polyneuropathy | 7 | 7229826 | C/T | 0.313 | -0.034 | 0.081 | 0.679 | 0.313 | trans |
| rs3774315 | Diabetic polyneuropathy | 3 | 172231986 | G/A | 0.278 | -0.054 | 0.085 | 0.527 | 0.278 | trans |
| rs149110519 | Diabetic polyneuropathy | 6 | 144385777 | T/C | 0.027 | -0.066 | 0.234 | 0.780 | 0.027 | trans |
| rs58609917 | Diabetic polyneuropathy | 7 | 35666267 | A/C | 0.333 | -0.139 | 0.080 | 0.084 | 0.333 | trans |
| rs6723921 | Diabetic polyneuropathy | 2 | 62531732 | G/A | 0.622 | -0.158 | 0.078 | 0.043 | 0.378 | trans |
| rs445 | Diabetic polyneuropathy | 7 | 92408370 | T/C | 0.059 | -0.162 | 0.161 | 0.314 | 0.059 | trans |
| MASP1 |  |  |  |  |  |  |  |  |  |  |
| rs6118 | Diabetic polyneuropathy | 14 | 95053863 | T/C | 0.112 | 0.204 | 0.120 | 0.088 | 0.112 | trans |
| rs10104003 | Diabetic polyneuropathy | 8 | 55422440 | T/C | 0.233 | 0.193 | 0.089 | 0.031 | 0.233 | trans |
| rs9987289 | Diabetic polyneuropathy | 8 | 9183358 | G/A | 0.857 | 0.118 | 0.116 | 0.310 | 0.143 | trans |
| rs704 | Diabetic polyneuropathy | 17 | 26694861 | A/G | 0.420 | 0.049 | 0.076 | 0.519 | 0.420 | trans |
| rs215223 | Diabetic polyneuropathy | 12 | 590259 | A/G | 0.366 | 0.036 | 0.078 | 0.645 | 0.366 | trans |
| rs9944724 | Diabetic polyneuropathy | 18 | 60184611 | G/T | 0.337 | -0.005 | 0.080 | 0.953 | 0.337 | trans |
| rs7899547 | Diabetic polyneuropathy | 10 | 54536839 | G/T | 0.666 | -0.047 | 0.080 | 0.558 | 0.334 | trans |
| rs4926 | Diabetic polyneuropathy | 11 | 57381989 | A/G | 0.306 | -0.061 | 0.082 | 0.458 | 0.306 | trans |
| rs3820897 | Diabetic polyneuropathy | 2 | 3642361 | T/C | 0.353 | -0.069 | 0.080 | 0.386 | 0.353 | trans |
| rs698090 | Diabetic polyneuropathy | 3 | 186964300 | T/C | 0.674 | -0.084 | 0.081 | 0.299 | 0.326 | cis |
| rs1486236 | Diabetic polyneuropathy | 2 | 180739450 | A/C | 0.366 | -0.105 | 0.079 | 0.182 | 0.366 | trans |
| ULBP2 |  |  |  |  |  |  |  |  |  |  |
| rs10104997 | Diabetic polyneuropathy | 8 | 55440068 | T/C | 0.233 | 0.191 | 0.089 | 0.032 | 0.233 | trans |
| rs4703854 | Diabetic polyneuropathy | 5 | 71693463 | T/C | 0.786 | 0.029 | 0.092 | 0.757 | 0.214 | trans |
| rs4804181 | Diabetic polyneuropathy | 19 | 12509536 | A/C | 0.776 | -0.096 | 0.093 | 0.304 | 0.224 | trans |
| PRCP |  |  |  |  |  |  |  |  |  |  |
| rs10104997 | Diabetic polyneuropathy | 8 | 55440068 | T/C | 0.233 | 0.191 | 0.089 | 0.032 | 0.233 | trans |
| rs4764822 | Diabetic polyneuropathy | 12 | 102216673 | T/C | 0.282 | 0.176 | 0.084 | 0.036 | 0.282 | trans |
| rs2229437 | Diabetic polyneuropathy | 11 | 82564294 | T/G | 0.848 | 0.030 | 0.105 | 0.777 | 0.152 | cis |
| FCN3 |  |  |  |  |  |  |  |  |  |  |
| rs10104997 | Diabetic polyneuropathy | 8 | 55440068 | T/C | 0.233 | 0.191 | 0.089 | 0.032 | 0.233 | trans |
| rs215224 | Diabetic polyneuropathy | 12 | 590449 | T/C | 0.634 | -0.034 | 0.078 | 0.665 | 0.366 | trans |
| LRP1 |  |  |  |  |  |  |  |  |  |  |
| rs217181 | Diabetic polyneuropathy | 16 | 72114002 | T/C | 0.214 | 0.114 | 0.092 | 0.216 | 0.214 | trans |
| rs2277998 | Diabetic polyneuropathy | 19 | 7831628 | A/G | 0.301 | 0.008 | 0.083 | 0.926 | 0.301 | trans |
| rs2229498 | Diabetic polyneuropathy | 10 | 70856852 | A/G | 0.866 | -0.046 | 0.111 | 0.682 | 0.134 | trans |
| CLEC4C |  |  |  |  |  |  |  |  |  |  |
| rs76428106 | Diabetic polyneuropathy | 13 | 28604007 | C/T | 0.012 | 0.322 | 0.347 | 0.353 | 0.012 | trans |
| rs7658518 | Diabetic polyneuropathy | 4 | 84160279 | A/G | 0.220 | 0.163 | 0.091 | 0.074 | 0.220 | trans |
| rs11789604 | Diabetic polyneuropathy | 9 | 139599129 | G/T | 0.334 | 0.096 | 0.081 | 0.236 | 0.334 | trans |
| rs62045817 | Diabetic polyneuropathy | 16 | 89045233 | T/C | 0.130 | 0.055 | 0.114 | 0.627 | 0.130 | trans |
| rs7573683 | Diabetic polyneuropathy | 2 | 60581094 | C/T | 0.626 | 0.049 | 0.078 | 0.527 | 0.374 | trans |
| rs10107630 | Diabetic polyneuropathy | 8 | 130603635 | T/C | 0.581 | 0.031 | 0.076 | 0.684 | 0.419 | trans |
| rs11055602 | Diabetic polyneuropathy | 12 | 7904111 | G/T | 0.279 | 0.030 | 0.084 | 0.724 | 0.279 | cis |
| rs876038 | Diabetic polyneuropathy | 7 | 50308527 | T/C | 0.300 | 0.028 | 0.082 | 0.735 | 0.300 | trans |
| rs62057151 | Diabetic polyneuropathy | 17 | 43903842 | T/C | 0.085 | 0.025 | 0.134 | 0.852 | 0.085 | trans |
| rs10838702 | Diabetic polyneuropathy | 11 | 47410888 | T/G | 0.299 | 0.020 | 0.083 | 0.810 | 0.299 | trans |
| rs10971417 | Diabetic polyneuropathy | 9 | 33121480 | T/C | 0.216 | -0.014 | 0.092 | 0.876 | 0.216 | trans |
| rs1490589 | Diabetic polyneuropathy | 4 | 105945776 | G/A | 0.691 | -0.050 | 0.082 | 0.538 | 0.309 | trans |
| rs4968607 | Diabetic polyneuropathy | 17 | 62132330 | A/G | 0.145 | -0.065 | 0.107 | 0.543 | 0.145 | trans |
| rs10441775 | Diabetic polyneuropathy | 9 | 94481908 | G/A | 0.768 | -0.080 | 0.089 | 0.372 | 0.232 | trans |
| rs7873862 | Diabetic polyneuropathy | 9 | 116113396 | T/C | 0.119 | -0.093 | 0.116 | 0.424 | 0.119 | trans |
| rs7129402 | Diabetic polyneuropathy | 11 | 121211756 | A/C | 0.643 | -0.136 | 0.080 | 0.089 | 0.357 | trans |
| rs9624326 | Diabetic polyneuropathy | 22 | 24135043 | T/C | 0.157 | -0.240 | 0.104 | 0.021 | 0.157 | trans |
| RAB26 |  |  |  |  |  |  |  |  |  |  |
| rs76428106 | Diabetic polyneuropathy | 13 | 28604007 | C/T | 0.012 | 0.322 | 0.347 | 0.353 | 0.012 | trans |
| rs6775011 | Diabetic polyneuropathy | 3 | 128396483 | C/T | 0.287 | 0.086 | 0.084 | 0.308 | 0.287 | trans |
| rs10799536 | Diabetic polyneuropathy | 1 | 229660791 | G/A | 0.134 | 0.079 | 0.110 | 0.473 | 0.134 | trans |
| rs72786786 | Diabetic polyneuropathy | 16 | 56985514 | A/G | 0.303 | 0.048 | 0.083 | 0.565 | 0.303 | trans |
| rs10107630 | Diabetic polyneuropathy | 8 | 130603635 | T/C | 0.581 | 0.031 | 0.076 | 0.684 | 0.419 | trans |
| rs2305637 | Diabetic polyneuropathy | 3 | 47045846 | T/C | 0.234 | 0.017 | 0.089 | 0.849 | 0.234 | trans |
| rs62132278 | Diabetic polyneuropathy | 19 | 836654 | T/C | 0.261 | 0.011 | 0.088 | 0.896 | 0.261 | trans |
| rs1707652 | Diabetic polyneuropathy | 3 | 165478799 | T/C | 0.248 | -0.037 | 0.087 | 0.672 | 0.248 | trans |
| rs444053 | Diabetic polyneuropathy | 19 | 16252072 | T/C | 0.338 | -0.099 | 0.081 | 0.223 | 0.338 | trans |
| SEMA4A |  |  |  |  |  |  |  |  |  |  |
| rs76428106 | Diabetic polyneuropathy | 13 | 28604007 | C/T | 0.012 | 0.322 | 0.347 | 0.353 | 0.012 | trans |
| rs10107630 | Diabetic polyneuropathy | 8 | 130603635 | T/C | 0.581 | 0.031 | 0.076 | 0.684 | 0.419 | trans |
| rs7695 | Diabetic polyneuropathy | 1 | 156147326 | C/T | 0.408 | 0.024 | 0.077 | 0.753 | 0.408 | cis |
| rs3821801 | Diabetic polyneuropathy | 3 | 186937221 | T/C | 0.346 | -0.006 | 0.080 | 0.936 | 0.346 | trans |
| rs62019249 | Diabetic polyneuropathy | 15 | 90749206 | C/T | 0.401 | -0.022 | 0.077 | 0.775 | 0.401 | trans |
| rs58560372 | Diabetic polyneuropathy | 19 | 38758752 | T/C | 0.151 | -0.034 | 0.106 | 0.750 | 0.151 | trans |
| rs7899547 | Diabetic polyneuropathy | 10 | 54536839 | G/T | 0.666 | -0.047 | 0.080 | 0.558 | 0.334 | trans |
| rs3184504 | Diabetic polyneuropathy | 12 | 111884608 | C/T | 0.591 | -0.051 | 0.077 | 0.504 | 0.409 | trans |
| KPNA2 |  |  |  |  |  |  |  |  |  |  |
| rs76428106 | Diabetic polyneuropathy | 13 | 28604007 | C/T | 0.012 | 0.322 | 0.347 | 0.353 | 0.012 | trans |
| rs34198449 | Diabetic polyneuropathy | 3 | 128381270 | A/G | 0.288 | 0.083 | 0.084 | 0.324 | 0.288 | trans |
| rs2071408 | Diabetic polyneuropathy | 14 | 103987078 | A/G | 0.375 | 0.047 | 0.078 | 0.550 | 0.375 | trans |
| rs61242663 | Diabetic polyneuropathy | 19 | 836059 | T/C | 0.272 | 0.035 | 0.087 | 0.686 | 0.272 | trans |
| rs10107630 | Diabetic polyneuropathy | 8 | 130603635 | T/C | 0.581 | 0.031 | 0.076 | 0.684 | 0.419 | trans |
| rs2305637 | Diabetic polyneuropathy | 3 | 47045846 | T/C | 0.234 | 0.017 | 0.089 | 0.849 | 0.234 | trans |
| rs6796 | Diabetic polyneuropathy | 7 | 6502367 | C/T | 0.321 | -0.010 | 0.082 | 0.900 | 0.321 | trans |
| rs11720167 | Diabetic polyneuropathy | 3 | 165486145 | G/T | 0.174 | -0.015 | 0.099 | 0.877 | 0.174 | trans |
| rs1864163 | Diabetic polyneuropathy | 16 | 56997233 | A/G | 0.208 | -0.058 | 0.093 | 0.531 | 0.208 | trans |
| rs8102236 | Diabetic polyneuropathy | 19 | 16250595 | G/A | 0.339 | -0.103 | 0.080 | 0.201 | 0.339 | trans |
| rs6573880 | Diabetic polyneuropathy | 14 | 25048853 | C/T | 0.123 | -0.131 | 0.115 | 0.254 | 0.123 | trans |
| KIT |  |  |  |  |  |  |  |  |  |  |
| rs74227709 | Diabetic polyneuropathy | 1 | 247722588 | A/G | 0.054 | 0.141 | 0.167 | 0.398 | 0.054 | trans |
| rs3008336 | Diabetic polyneuropathy | 10 | 135099533 | C/T | 0.865 | 0.103 | 0.110 | 0.349 | 0.135 | trans |
| rs35056404 | Diabetic polyneuropathy | 11 | 44600733 | A/G | 0.043 | 0.084 | 0.187 | 0.655 | 0.043 | trans |
| rs72704195 | Diabetic polyneuropathy | 4 | 175490632 | G/A | 0.363 | 0.078 | 0.080 | 0.329 | 0.363 | trans |
| rs115340020 | Diabetic polyneuropathy | 1 | 92766438 | A/G | 0.009 | 0.021 | 0.390 | 0.957 | 0.009 | trans |
| rs17758695 | Diabetic polyneuropathy | 18 | 60920854 | T/C | 0.045 | 0.018 | 0.187 | 0.925 | 0.045 | trans |
| rs7098111 | Diabetic polyneuropathy | 10 | 119573178 | T/C | 0.185 | -0.021 | 0.098 | 0.829 | 0.185 | trans |
| rs9839302 | Diabetic polyneuropathy | 3 | 197795424 | C/T | 0.311 | -0.035 | 0.082 | 0.673 | 0.311 | trans |
| rs78744187 | Diabetic polyneuropathy | 19 | 33754548 | T/C | 0.148 | -0.062 | 0.108 | 0.568 | 0.148 | trans |
| rs1805007 | Diabetic polyneuropathy | 16 | 89986117 | T/C | 0.067 | -0.088 | 0.152 | 0.563 | 0.067 | trans |
| rs218263 | Diabetic polyneuropathy | 4 | 55408104 | T/C | 0.149 | -0.092 | 0.107 | 0.392 | 0.149 | cis |
| rs12609373 | Diabetic polyneuropathy | 19 | 52298281 | C/T | 0.251 | -0.147 | 0.087 | 0.092 | 0.251 | trans |
| rs72853300 | Diabetic polyneuropathy | 2 | 145638766 | T/C | 0.128 | -0.177 | 0.113 | 0.118 | 0.128 | trans |
| rs12203592 | Diabetic polyneuropathy | 6 | 396321 | T/C | 0.031 | -0.186 | 0.222 | 0.402 | 0.031 | trans |
| rs10118718 | Diabetic polyneuropathy | 9 | 109438975 | A/G | 0.079 | -0.195 | 0.138 | 0.159 | 0.079 | trans |
| DCXR |  |  |  |  |  |  |  |  |  |  |
| rs3747207 | Diabetic polyneuropathy | 22 | 44324855 | A/G | 0.226 | -0.010 | 0.090 | 0.915 | 0.226 | trans |
| rs10883451 | Diabetic polyneuropathy | 10 | 101924418 | C/T | 0.389 | -0.058 | 0.077 | 0.456 | 0.389 | trans |
| CDH17 |  |  |  |  |  |  |  |  |  |  |
| rs1012143 | Diabetic polyneuropathy | 2 | 107662341 | G/A | 0.393 | 0.130 | 0.078 | 0.096 | 0.393 | trans |
| rs601338 | Diabetic polyneuropathy | 19 | 49206674 | G/A | 0.626 | 0.044 | 0.078 | 0.571 | 0.375 | trans |
| rs7833351 | Diabetic polyneuropathy | 8 | 95182178 | G/A | 0.364 | 0.011 | 0.079 | 0.890 | 0.364 | cis |
| rs4556017 | Diabetic polyneuropathy | 7 | 100632790 | C/T | 0.179 | 0.007 | 0.100 | 0.942 | 0.179 | trans |
| rs9815073 | Diabetic polyneuropathy | 3 | 188115682 | A/C | 0.328 | -0.036 | 0.081 | 0.654 | 0.328 | trans |
| rs708686 | Diabetic polyneuropathy | 19 | 5840619 | T/C | 0.334 | -0.105 | 0.081 | 0.194 | 0.334 | trans |
| CD5 |  |  |  |  |  |  |  |  |  |  |
| rs2075803 | Diabetic polyneuropathy | 19 | 51628529 | A/G | 0.404 | 0.064 | 0.077 | 0.407 | 0.404 | trans |
| rs10121987 | Diabetic polyneuropathy | 9 | 33119818 | G/A | 0.261 | -0.016 | 0.086 | 0.854 | 0.261 | trans |
| PSAP |  |  |  |  |  |  |  |  |  |  |
| rs10128858 | Diabetic polyneuropathy | 12 | 102221366 | A/G | 0.717 | -0.173 | 0.084 | 0.040 | 0.283 | trans |
| IDS |  |  |  |  |  |  |  |  |  |  |
| rs704 | Diabetic polyneuropathy | 17 | 26694861 | A/G | 0.420 | 0.049 | 0.076 | 0.519 | 0.420 | trans |
| rs10128858 | Diabetic polyneuropathy | 12 | 102221366 | A/G | 0.717 | -0.173 | 0.084 | 0.040 | 0.283 | trans |
| FJX1 |  |  |  |  |  |  |  |  |  |  |
| rs12740374 | Diabetic polyneuropathy | 1 | 109817590 | T/G | 0.215 | 0.109 | 0.092 | 0.235 | 0.215 | trans |
| rs10128858 | Diabetic polyneuropathy | 12 | 102221366 | A/G | 0.717 | -0.173 | 0.084 | 0.040 | 0.283 | trans |
| CD84 |  |  |  |  |  |  |  |  |  |  |
| rs1354034 | Diabetic polyneuropathy | 3 | 56849749 | C/T | 0.707 | 0.052 | 0.083 | 0.532 | 0.293 | trans |
| rs114694170 | Diabetic polyneuropathy | 5 | 88180196 | C/T | 0.056 | 0.015 | 0.163 | 0.926 | 0.056 | trans |
| rs7080386 | Diabetic polyneuropathy | 10 | 65048306 | A/C | 0.384 | -0.001 | 0.078 | 0.993 | 0.384 | trans |
| rs597808 | Diabetic polyneuropathy | 12 | 111973358 | G/A | 0.586 | -0.054 | 0.077 | 0.480 | 0.415 | trans |
| rs467369 | Diabetic polyneuropathy | 9 | 136905765 | T/C | 0.703 | -0.091 | 0.083 | 0.272 | 0.297 | trans |
| TNFRSF11B |  |  |  |  |  |  |  |  |  |  |
| rs77542162 | Diabetic polyneuropathy | 17 | 67081278 | A/G | 0.993 | 0.407 | 0.465 | 0.382 | 0.007 | trans |
| rs704 | Diabetic polyneuropathy | 17 | 26694861 | A/G | 0.420 | 0.049 | 0.076 | 0.519 | 0.420 | trans |
| rs3747207 | Diabetic polyneuropathy | 22 | 44324855 | A/G | 0.226 | -0.010 | 0.090 | 0.915 | 0.226 | trans |
| rs7927191 | Diabetic polyneuropathy | 11 | 16252982 | C/T | 0.223 | -0.037 | 0.090 | 0.680 | 0.223 | trans |
| rs79287178 | Diabetic polyneuropathy | 3 | 172294500 | A/G | 0.042 | -0.058 | 0.192 | 0.763 | 0.042 | trans |
| CEACAM1 |  |  |  |  |  |  |  |  |  |  |
| rs10136766 | Diabetic polyneuropathy | 14 | 106232585 | G/A | 0.594 | -0.040 | 0.080 | 0.615 | 0.406 | trans |
| EFS |  |  |  |  |  |  |  |  |  |  |
| rs10137082 | Diabetic polyneuropathy | 14 | 23840033 | T/C | 0.156 | -0.153 | 0.105 | 0.146 | 0.156 | cis |
| ACP5 |  |  |  |  |  |  |  |  |  |  |
| rs2305799 | Diabetic polyneuropathy | 19 | 11687351 | T/C | 0.145 | 0.141 | 0.108 | 0.193 | 0.145 | cis |
| rs2073333 | Diabetic polyneuropathy | 14 | 94844562 | T/C | 0.243 | 0.053 | 0.088 | 0.547 | 0.243 | trans |
| rs35382467 | Diabetic polyneuropathy | 11 | 128199283 | A/G | 0.104 | 0.023 | 0.124 | 0.856 | 0.104 | trans |
| rs10813949 | Diabetic polyneuropathy | 9 | 33123464 | A/G | 0.261 | -0.013 | 0.086 | 0.879 | 0.261 | trans |
| rs2229498 | Diabetic polyneuropathy | 10 | 70856852 | A/G | 0.866 | -0.046 | 0.111 | 0.682 | 0.134 | trans |
| rs78755089 | Diabetic polyneuropathy | 16 | 79576621 | T/G | 0.055 | -0.200 | 0.169 | 0.235 | 0.055 | trans |
| AGER |  |  |  |  |  |  |  |  |  |  |
| rs117048263 | Diabetic polyneuropathy | 3 | 169089582 | A/C | 0.011 | 0.612 | 0.371 | 0.099 | 0.011 | trans |
| rs35705950 | Diabetic polyneuropathy | 11 | 1241221 | T/G | 0.103 | 0.156 | 0.128 | 0.224 | 0.103 | trans |
| rs73093253 | Diabetic polyneuropathy | 4 | 24589064 | A/G | 0.243 | 0.089 | 0.088 | 0.311 | 0.243 | trans |
| rs79730878 | Diabetic polyneuropathy | 17 | 43849415 | C/T | 0.082 | 0.047 | 0.136 | 0.731 | 0.082 | trans |
| rs1569419 | Diabetic polyneuropathy | 1 | 2996602 | T/C | 0.304 | 0.042 | 0.083 | 0.615 | 0.304 | trans |
| rs17762402 | Diabetic polyneuropathy | 11 | 61553201 | A/G | 0.096 | 0.039 | 0.131 | 0.767 | 0.096 | trans |
| rs7212311 | Diabetic polyneuropathy | 17 | 73094407 | T/C | 0.378 | 0.037 | 0.078 | 0.636 | 0.378 | trans |
| rs7962469 | Diabetic polyneuropathy | 12 | 52348259 | A/G | 0.318 | 0.022 | 0.081 | 0.789 | 0.318 | trans |
| rs2852746 | Diabetic polyneuropathy | 18 | 42229324 | A/G | 0.291 | 0.016 | 0.083 | 0.846 | 0.291 | trans |
| rs7616330 | Diabetic polyneuropathy | 3 | 71115751 | A/C | 0.242 | 0.016 | 0.089 | 0.859 | 0.242 | trans |
| rs10824665 | Diabetic polyneuropathy | 10 | 80676990 | A/G | 0.261 | -0.039 | 0.087 | 0.652 | 0.261 | trans |
| rs6961094 | Diabetic polyneuropathy | 7 | 101546316 | T/C | 0.099 | -0.053 | 0.126 | 0.674 | 0.099 | trans |
| rs11243421 | Diabetic polyneuropathy | 9 | 134426038 | T/C | 0.388 | -0.079 | 0.078 | 0.313 | 0.388 | trans |
| rs56332871 | Diabetic polyneuropathy | 15 | 96714816 | A/C | 0.291 | -0.084 | 0.084 | 0.313 | 0.291 | trans |
| rs12461753 | Diabetic polyneuropathy | 19 | 38202515 | A/G | 0.197 | -0.086 | 0.096 | 0.372 | 0.197 | trans |
| rs7744366 | Diabetic polyneuropathy | 6 | 6854489 | A/G | 0.111 | -0.107 | 0.119 | 0.371 | 0.111 | trans |
| rs12201133 | Diabetic polyneuropathy | 6 | 130290769 | A/C | 0.212 | -0.112 | 0.092 | 0.222 | 0.212 | trans |
| rs149438404 | Diabetic polyneuropathy | 7 | 74024050 | A/G | 0.021 | -0.148 | 0.261 | 0.571 | 0.021 | trans |
| rs4685105 | Diabetic polyneuropathy | 3 | 14320176 | G/A | 0.412 | -0.151 | 0.077 | 0.049 | 0.412 | trans |
| ACP2 |  |  |  |  |  |  |  |  |  |  |
| rs28929474 | Diabetic polyneuropathy | 14 | 94844947 | T/C | 0.020 | -0.002 | 0.272 | 0.993 | 0.020 | trans |
| rs10139058 | Diabetic polyneuropathy | 14 | 107141916 | T/C | 0.398 | -0.041 | 0.078 | 0.599 | 0.398 | trans |
| NME3 |  |  |  |  |  |  |  |  |  |  |
| rs55707100 | Diabetic polyneuropathy | 15 | 43820717 | T/C | 0.006 | 0.181 | 0.496 | 0.715 | 0.006 | trans |
| rs12975366 | Diabetic polyneuropathy | 19 | 54759361 | C/T | 0.372 | 0.142 | 0.079 | 0.071 | 0.372 | trans |
| rs12424638 | Diabetic polyneuropathy | 12 | 49318800 | A/G | 0.075 | 0.081 | 0.144 | 0.575 | 0.075 | trans |
| rs2278093 | Diabetic polyneuropathy | 12 | 29534209 | A/C | 0.295 | 0.033 | 0.083 | 0.691 | 0.295 | trans |
| rs2519093 | Diabetic polyneuropathy | 9 | 136141870 | T/C | 0.201 | 0.028 | 0.094 | 0.762 | 0.201 | trans |
| rs7599 | Diabetic polyneuropathy | 19 | 36038390 | G/A | 0.677 | -0.061 | 0.081 | 0.451 | 0.323 | trans |
| rs1801689 | Diabetic polyneuropathy | 17 | 64210580 | C/A | 0.010 | -0.158 | 0.383 | 0.681 | 0.010 | trans |
| rs188468174 | Diabetic polyneuropathy | 1 | 25291697 | T/C | 0.004 | -0.166 | 0.540 | 0.758 | 0.004 | trans |
| CD244 |  |  |  |  |  |  |  |  |  |  |
| rs2009581 | Diabetic polyneuropathy | 2 | 111807677 | A/G | 0.264 | 0.088 | 0.086 | 0.302 | 0.264 | trans |
| rs79977579 | Diabetic polyneuropathy | 12 | 54694560 | A/C | 0.094 | 0.049 | 0.129 | 0.707 | 0.094 | trans |
| rs10815098 | Diabetic polyneuropathy | 9 | 4865338 | T/C | 0.219 | 0.048 | 0.092 | 0.605 | 0.219 | trans |
| rs12574844 | Diabetic polyneuropathy | 11 | 126273386 | A/G | 0.099 | -0.002 | 0.127 | 0.988 | 0.099 | trans |
| rs34458031 | Diabetic polyneuropathy | 3 | 98752035 | A/G | 0.070 | -0.004 | 0.148 | 0.979 | 0.070 | trans |
| rs113315674 | Diabetic polyneuropathy | 9 | 100720053 | A/G | 0.401 | -0.012 | 0.078 | 0.879 | 0.401 | trans |
| rs3184504 | Diabetic polyneuropathy | 12 | 111884608 | C/T | 0.591 | -0.051 | 0.077 | 0.504 | 0.409 | trans |
| rs37456 | Diabetic polyneuropathy | 1 | 44292989 | T/C | 0.361 | -0.089 | 0.079 | 0.258 | 0.361 | trans |
| rs12616639 | Diabetic polyneuropathy | 2 | 62529925 | G/A | 0.621 | -0.163 | 0.078 | 0.036 | 0.380 | trans |
| CCDC50 |  |  |  |  |  |  |  |  |  |  |
| rs1354034 | Diabetic polyneuropathy | 3 | 56849749 | C/T | 0.707 | 0.052 | 0.083 | 0.532 | 0.293 | trans |
| rs11827437 | Diabetic polyneuropathy | 11 | 6647622 | T/C | 0.606 | 0.035 | 0.077 | 0.650 | 0.394 | trans |
| rs1015193 | Diabetic polyneuropathy | 10 | 73584426 | T/C | 0.594 | -0.006 | 0.077 | 0.941 | 0.406 | trans |
| rs4602967 | Diabetic polyneuropathy | 9 | 115913145 | T/C | 0.244 | -0.006 | 0.089 | 0.943 | 0.244 | trans |
| PGLYRP2 |  |  |  |  |  |  |  |  |  |  |
| rs10164310 | Diabetic polyneuropathy | 19 | 15585945 | A/G | 0.186 | 0.012 | 0.097 | 0.905 | 0.186 | cis |
| ITGAM |  |  |  |  |  |  |  |  |  |  |
| rs11150613 | Diabetic polyneuropathy | 16 | 31357810 | T/C | 0.596 | -0.008 | 0.077 | 0.922 | 0.404 | cis |
| rs3184504 | Diabetic polyneuropathy | 12 | 111884608 | C/T | 0.591 | -0.051 | 0.077 | 0.504 | 0.409 | trans |
| rs4601794 | Diabetic polyneuropathy | 11 | 126245145 | G/A | 0.071 | -0.086 | 0.148 | 0.564 | 0.071 | trans |
| rs118162691 | Diabetic polyneuropathy | 8 | 21767809 | A/C | 0.047 | -0.303 | 0.177 | 0.088 | 0.047 | trans |
| B4GAT1 |  |  |  |  |  |  |  |  |  |  |
| rs74611001 | Diabetic polyneuropathy | 11 | 94220519 | A/C | 0.024 | 0.200 | 0.240 | 0.404 | 0.024 | trans |
| rs12975366 | Diabetic polyneuropathy | 19 | 54759361 | C/T | 0.372 | 0.142 | 0.079 | 0.071 | 0.372 | trans |
| rs10217770 | Diabetic polyneuropathy | 9 | 33146393 | A/C | 0.380 | 0.075 | 0.078 | 0.333 | 0.380 | trans |
| rs11130630 | Diabetic polyneuropathy | 3 | 58360800 | G/A | 0.247 | 0.047 | 0.088 | 0.595 | 0.247 | trans |
| rs10896113 | Diabetic polyneuropathy | 11 | 66117111 | T/C | 0.235 | 0.044 | 0.089 | 0.619 | 0.235 | cis |
| rs56278466 | Diabetic polyneuropathy | 10 | 17875857 | G/T | 0.581 | -0.003 | 0.077 | 0.970 | 0.419 | trans |
| rs10168551 | Diabetic polyneuropathy | 2 | 168597986 | C/T | 0.196 | -0.051 | 0.095 | 0.592 | 0.196 | trans |
| GAGE2A |  |  |  |  |  |  |  |  |  |  |
| rs1017301 | Diabetic polyneuropathy | 12 | 9362931 | A/C | 0.674 | -0.116 | 0.081 | 0.151 | 0.326 | trans |
| EBI3_IL27 |  |  |  |  |  |  |  |  |  |  |
| rs4986790 | Diabetic polyneuropathy | 9 | 120475302 | G/A | 0.100 | 0.110 | 0.126 | 0.385 | 0.100 | trans |
| rs62165726 | Diabetic polyneuropathy | 2 | 134966562 | A/C | 0.026 | 0.074 | 0.243 | 0.760 | 0.026 | trans |
| rs704 | Diabetic polyneuropathy | 17 | 26694861 | A/G | 0.420 | 0.049 | 0.076 | 0.519 | 0.420 | trans |
| rs5743618 | Diabetic polyneuropathy | 4 | 38798648 | A/C | 0.153 | 0.024 | 0.104 | 0.820 | 0.153 | trans |
| rs10513801 | Diabetic polyneuropathy | 3 | 185822353 | G/T | 0.101 | -0.020 | 0.125 | 0.876 | 0.101 | trans |
| rs10183338 | Diabetic polyneuropathy | 2 | 111610816 | T/G | 0.290 | -0.026 | 0.083 | 0.755 | 0.290 | trans |
| rs4486555 | Diabetic polyneuropathy | 10 | 101799302 | T/G | 0.287 | -0.043 | 0.083 | 0.606 | 0.287 | trans |
| rs11713634 | Diabetic polyneuropathy | 3 | 194061578 | A/G | 0.350 | -0.050 | 0.079 | 0.533 | 0.350 | trans |
| rs2529440 | Diabetic polyneuropathy | 7 | 30511794 | T/C | 0.404 | -0.050 | 0.077 | 0.518 | 0.404 | trans |
| rs3184504 | Diabetic polyneuropathy | 12 | 111884608 | C/T | 0.591 | -0.051 | 0.077 | 0.504 | 0.409 | trans |
| rs4804669 | Diabetic polyneuropathy | 19 | 12502457 | G/A | 0.791 | -0.134 | 0.095 | 0.161 | 0.209 | trans |
| rs58298943 | Diabetic polyneuropathy | 12 | 57391292 | T/C | 0.101 | -0.143 | 0.126 | 0.255 | 0.101 | trans |
| rs72749499 | Diabetic polyneuropathy | 15 | 57295579 | G/A | 0.034 | -0.261 | 0.210 | 0.213 | 0.034 | trans |
| CRH |  |  |  |  |  |  |  |  |  |  |
| rs7387462 | Diabetic polyneuropathy | 8 | 100603910 | T/C | 0.041 | 0.424 | 0.193 | 0.028 | 0.041 | trans |
| rs7412 | Diabetic polyneuropathy | 19 | 45412079 | T/C | 0.053 | 0.166 | 0.164 | 0.311 | 0.053 | trans |
| rs12740374 | Diabetic polyneuropathy | 1 | 109817590 | T/G | 0.215 | 0.109 | 0.092 | 0.235 | 0.215 | trans |
| rs7192602 | Diabetic polyneuropathy | 16 | 78346429 | A/G | 0.289 | 0.052 | 0.083 | 0.532 | 0.289 | trans |
| rs13017862 | Diabetic polyneuropathy | 2 | 200449588 | C/T | 0.124 | 0.044 | 0.115 | 0.704 | 0.124 | trans |
| rs111632177 | Diabetic polyneuropathy | 7 | 150931805 | A/G | 0.118 | -0.004 | 0.116 | 0.973 | 0.118 | trans |
| rs10774624 | Diabetic polyneuropathy | 12 | 111833788 | A/G | 0.597 | -0.015 | 0.077 | 0.847 | 0.403 | trans |
| rs10183338 | Diabetic polyneuropathy | 2 | 111610816 | T/G | 0.290 | -0.026 | 0.083 | 0.755 | 0.290 | trans |
| rs1229027 | Diabetic polyneuropathy | 7 | 39023276 | A/G | 0.214 | -0.032 | 0.092 | 0.732 | 0.214 | trans |
| rs16840585 | Diabetic polyneuropathy | 2 | 157466322 | C/T | 0.155 | -0.033 | 0.104 | 0.751 | 0.155 | trans |
| rs7687767 | Diabetic polyneuropathy | 4 | 57824932 | G/A | 0.234 | -0.051 | 0.089 | 0.566 | 0.234 | trans |
| TMEM154 |  |  |  |  |  |  |  |  |  |  |
| rs1018635 | Diabetic polyneuropathy | 10 | 20226672 | T/C | 0.227 | 0.013 | 0.090 | 0.882 | 0.227 | trans |
| rs6027 | Diabetic polyneuropathy | 1 | 169483561 | T/C | 0.915 | -0.074 | 0.135 | 0.584 | 0.085 | trans |
| MPO |  |  |  |  |  |  |  |  |  |  |
| rs10916493 | Diabetic polyneuropathy | 1 | 229634397 | T/G | 0.133 | 0.061 | 0.110 | 0.578 | 0.133 | trans |
| CLEC6A |  |  |  |  |  |  |  |  |  |  |
| rs76428106 | Diabetic polyneuropathy | 13 | 28604007 | C/T | 0.012 | 0.322 | 0.347 | 0.353 | 0.012 | trans |
| rs4795412 | Diabetic polyneuropathy | 17 | 38150604 | T/C | 0.398 | 0.096 | 0.077 | 0.215 | 0.398 | trans |
| rs872629 | Diabetic polyneuropathy | 19 | 52130638 | A/C | 0.163 | -0.023 | 0.103 | 0.826 | 0.163 | trans |
| rs3184504 | Diabetic polyneuropathy | 12 | 111884608 | C/T | 0.591 | -0.051 | 0.077 | 0.504 | 0.409 | trans |
| rs2070901 | Diabetic polyneuropathy | 1 | 161185058 | T/G | 0.283 | -0.089 | 0.084 | 0.291 | 0.283 | trans |
| rs11045427 | Diabetic polyneuropathy | 12 | 8606523 | A/C | 0.249 | -0.116 | 0.088 | 0.187 | 0.249 | cis |
| rs188468174 | Diabetic polyneuropathy | 1 | 25291697 | T/C | 0.004 | -0.166 | 0.540 | 0.758 | 0.004 | trans |
| MERTK |  |  |  |  |  |  |  |  |  |  |
| rs10188642 | Diabetic polyneuropathy | 2 | 112741099 | A/G | 0.668 | 0.058 | 0.080 | 0.468 | 0.333 | cis |
| rs111981233 | Diabetic polyneuropathy | 19 | 50016479 | G/T | 0.045 | 0.045 | 0.180 | 0.803 | 0.045 | trans |
| rs11596680 | Diabetic polyneuropathy | 10 | 20208483 | G/A | 0.274 | 0.014 | 0.084 | 0.872 | 0.274 | trans |
| rs7088799 | Diabetic polyneuropathy | 10 | 65016174 | G/T | 0.384 | 0.002 | 0.078 | 0.981 | 0.384 | trans |
| rs28929474 | Diabetic polyneuropathy | 14 | 94844947 | T/C | 0.020 | -0.002 | 0.272 | 0.993 | 0.020 | trans |
| rs58895965 | Diabetic polyneuropathy | 19 | 35551428 | A/C | 0.152 | -0.023 | 0.106 | 0.830 | 0.152 | trans |
| rs9738226 | Diabetic polyneuropathy | 12 | 121423659 | G/A | 0.584 | -0.089 | 0.077 | 0.248 | 0.416 | trans |
| CD1C |  |  |  |  |  |  |  |  |  |  |
| rs76428106 | Diabetic polyneuropathy | 13 | 28604007 | C/T | 0.012 | 0.322 | 0.347 | 0.353 | 0.012 | trans |
| rs11963621 | Diabetic polyneuropathy | 6 | 44591006 | C/T | 0.107 | 0.177 | 0.122 | 0.147 | 0.107 | trans |
| rs1847472 | Diabetic polyneuropathy | 6 | 90973159 | A/C | 0.252 | 0.134 | 0.087 | 0.122 | 0.252 | trans |
| rs10189685 | Diabetic polyneuropathy | 2 | 203488449 | A/G | 0.265 | 0.095 | 0.085 | 0.266 | 0.265 | trans |
| rs61788913 | Diabetic polyneuropathy | 1 | 60091168 | T/G | 0.071 | 0.040 | 0.147 | 0.789 | 0.071 | trans |
| rs6063502 | Diabetic polyneuropathy | 20 | 48955595 | G/A | 0.385 | 0.020 | 0.078 | 0.795 | 0.385 | trans |
| rs3808460 | Diabetic polyneuropathy | 8 | 116597635 | C/T | 0.691 | -0.053 | 0.082 | 0.518 | 0.309 | trans |
| rs653178 | Diabetic polyneuropathy | 12 | 112007756 | T/C | 0.584 | -0.054 | 0.077 | 0.477 | 0.416 | trans |
| rs17272847 | Diabetic polyneuropathy | 19 | 49979398 | A/G | 0.123 | -0.056 | 0.116 | 0.633 | 0.123 | trans |
| rs12599288 | Diabetic polyneuropathy | 16 | 11822066 | C/T | 0.264 | -0.122 | 0.086 | 0.154 | 0.264 | trans |
| rs4698932 | Diabetic polyneuropathy | 4 | 106049147 | A/G | 0.584 | -0.169 | 0.077 | 0.028 | 0.416 | trans |
| TIMD4 |  |  |  |  |  |  |  |  |  |  |
| rs77542162 | Diabetic polyneuropathy | 17 | 67081278 | A/G | 0.993 | 0.407 | 0.465 | 0.382 | 0.007 | trans |
| rs72823014 | Diabetic polyneuropathy | 10 | 115786236 | A/G | 0.093 | 0.240 | 0.129 | 0.063 | 0.093 | trans |
| rs66817580 | Diabetic polyneuropathy | 21 | 42689637 | T/G | 0.096 | 0.176 | 0.127 | 0.167 | 0.096 | trans |
| rs2737245 | Diabetic polyneuropathy | 8 | 116658583 | T/G | 0.309 | 0.099 | 0.082 | 0.229 | 0.309 | trans |
| rs10189685 | Diabetic polyneuropathy | 2 | 203488449 | A/G | 0.265 | 0.095 | 0.085 | 0.266 | 0.265 | trans |
| rs601338 | Diabetic polyneuropathy | 19 | 49206674 | G/A | 0.626 | 0.044 | 0.078 | 0.571 | 0.375 | trans |
| rs34623398 | Diabetic polyneuropathy | 12 | 29489158 | CAACT/C | 0.291 | 0.030 | 0.083 | 0.715 | 0.291 | trans |
| rs10769256 | Diabetic polyneuropathy | 11 | 47378396 | T/C | 0.301 | 0.028 | 0.083 | 0.734 | 0.301 | trans |
| rs67833823 | Diabetic polyneuropathy | 2 | 232225946 | A/G | 0.168 | 0.023 | 0.102 | 0.821 | 0.168 | trans |
| rs11395592 | Diabetic polyneuropathy | 3 | 69842341 | AT/A | 0.417 | 0.022 | 0.077 | 0.779 | 0.417 | trans |
| rs10778118 | Diabetic polyneuropathy | 12 | 101824506 | T/C | 0.753 | -0.014 | 0.088 | 0.871 | 0.247 | trans |
| rs1060622 | Diabetic polyneuropathy | 1 | 93620393 | A/G | 0.632 | -0.029 | 0.078 | 0.714 | 0.368 | trans |
| rs2143876 | Diabetic polyneuropathy | 20 | 39119074 | C/T | 0.110 | -0.038 | 0.121 | 0.754 | 0.110 | trans |
| rs34417180 | Diabetic polyneuropathy | 16 | 72219756 | A/C | 0.168 | -0.060 | 0.101 | 0.548 | 0.168 | trans |
| rs2142306 | Diabetic polyneuropathy | 8 | 134470631 | C/T | 0.375 | -0.079 | 0.078 | 0.311 | 0.375 | trans |
| rs4704826 | Diabetic polyneuropathy | 5 | 156392082 | A/C | 0.667 | -0.104 | 0.081 | 0.197 | 0.333 | cis |
| rs12145753 | Diabetic polyneuropathy | 1 | 235095939 | T/C | 0.312 | -0.125 | 0.082 | 0.126 | 0.312 | trans |
| rs10456852 | Diabetic polyneuropathy | 6 | 106356046 | T/C | 0.096 | -0.132 | 0.129 | 0.306 | 0.096 | trans |
| SEMA3C |  |  |  |  |  |  |  |  |  |  |
| rs1019016 | Diabetic polyneuropathy | 7 | 80570562 | T/G | 0.583 | 0.098 | 0.077 | 0.201 | 0.417 | cis |
| CSAG1 |  |  |  |  |  |  |  |  |  |  |
| rs10203039 | Diabetic polyneuropathy | 2 | 219187367 | T/C | 0.292 | 0.037 | 0.083 | 0.659 | 0.292 | trans |
| TESC |  |  |  |  |  |  |  |  |  |  |
| rs1354034 | Diabetic polyneuropathy | 3 | 56849749 | C/T | 0.707 | 0.052 | 0.083 | 0.532 | 0.293 | trans |
| rs10203039 | Diabetic polyneuropathy | 2 | 219187367 | T/C | 0.292 | 0.037 | 0.083 | 0.659 | 0.292 | trans |
| rs59756806 | Diabetic polyneuropathy | 12 | 117479019 | T/C | 0.714 | -0.107 | 0.083 | 0.200 | 0.286 | cis |
| CKMT1A_CKMT1B |  |  |  |  |  |  |  |  |  |  |
| rs700882 | Diabetic polyneuropathy | 2 | 101731397 | T/C | 0.202 | 0.289 | 0.094 | 0.002 | 0.202 | trans |
| rs601338 | Diabetic polyneuropathy | 19 | 49206674 | G/A | 0.626 | 0.044 | 0.078 | 0.571 | 0.375 | trans |
| rs10205067 | Diabetic polyneuropathy | 2 | 112789381 | G/A | 0.081 | 0.009 | 0.140 | 0.948 | 0.081 | trans |
| rs73082019 | Diabetic polyneuropathy | 7 | 24756643 | G/A | 0.087 | -0.160 | 0.135 | 0.235 | 0.087 | trans |
| RAB6B |  |  |  |  |  |  |  |  |  |  |
| rs1354034 | Diabetic polyneuropathy | 3 | 56849749 | C/T | 0.707 | 0.052 | 0.083 | 0.532 | 0.293 | trans |
| rs10212397 | Diabetic polyneuropathy | 3 | 133607140 | A/G | 0.208 | -0.079 | 0.094 | 0.400 | 0.208 | cis |
| CD6 |  |  |  |  |  |  |  |  |  |  |
| rs7161799 | Diabetic polyneuropathy | 15 | 58770523 | T/C | 0.043 | 0.120 | 0.187 | 0.522 | 0.043 | trans |
| rs10957898 | Diabetic polyneuropathy | 8 | 79617313 | A/G | 0.227 | 0.097 | 0.090 | 0.280 | 0.227 | trans |
| rs10214273 | Diabetic polyneuropathy | 5 | 35883986 | G/T | 0.335 | 0.056 | 0.080 | 0.482 | 0.335 | trans |
| rs62454712 | Diabetic polyneuropathy | 7 | 6498057 | C/T | 0.264 | 0.011 | 0.087 | 0.902 | 0.264 | trans |
| rs3184504 | Diabetic polyneuropathy | 12 | 111884608 | C/T | 0.591 | -0.051 | 0.077 | 0.504 | 0.409 | trans |
| rs28413174 | Diabetic polyneuropathy | 19 | 17516620 | T/C | 0.182 | -0.151 | 0.098 | 0.124 | 0.182 | trans |
| rs11230563 | Diabetic polyneuropathy | 11 | 60776209 | T/C | 0.248 | -0.209 | 0.088 | 0.017 | 0.248 | cis |
| AMY1A |  |  |  |  |  |  |  |  |  |  |
| rs601338 | Diabetic polyneuropathy | 19 | 49206674 | G/A | 0.626 | 0.044 | 0.078 | 0.571 | 0.375 | trans |
| rs2227296 | Diabetic polyneuropathy | 12 | 11000934 | G/A | 0.269 | -0.057 | 0.085 | 0.506 | 0.269 | trans |
| rs10218826 | Diabetic polyneuropathy | 1 | 207124281 | T/C | 0.402 | -0.123 | 0.077 | 0.110 | 0.402 | trans |
| SHH |  |  |  |  |  |  |  |  |  |  |
| rs1864163 | Diabetic polyneuropathy | 16 | 56997233 | A/G | 0.208 | -0.058 | 0.093 | 0.531 | 0.208 | trans |
| KIAA0101 |  |  |  |  |  |  |  |  |  |  |
| rs102275 | Diabetic polyneuropathy | 11 | 61557803 | T/C | 0.584 | 0.123 | 0.077 | 0.107 | 0.416 | trans |
| rs62295996 | Diabetic polyneuropathy | 3 | 165482064 | A/G | 0.174 | -0.015 | 0.099 | 0.878 | 0.174 | trans |
| EHMT2 |  |  |  |  |  |  |  |  |  |  |
| rs112001035 | Diabetic polyneuropathy | 17 | 66823805 | A/G | 0.081 | 0.352 | 0.140 | 0.012 | 0.081 | trans |
| rs102275 | Diabetic polyneuropathy | 11 | 61557803 | T/C | 0.584 | 0.123 | 0.077 | 0.107 | 0.416 | trans |
| rs704 | Diabetic polyneuropathy | 17 | 26694861 | A/G | 0.420 | 0.049 | 0.076 | 0.519 | 0.420 | trans |
| rs3764261 | Diabetic polyneuropathy | 16 | 56993324 | A/C | 0.279 | 0.042 | 0.084 | 0.615 | 0.279 | trans |
| rs11720167 | Diabetic polyneuropathy | 3 | 165486145 | G/T | 0.174 | -0.015 | 0.099 | 0.877 | 0.174 | trans |
| rs1601935 | Diabetic polyneuropathy | 15 | 58671765 | T/G | 0.616 | -0.034 | 0.078 | 0.662 | 0.384 | trans |
| rs780093 | Diabetic polyneuropathy | 2 | 27742603 | T/C | 0.353 | -0.111 | 0.079 | 0.163 | 0.353 | trans |
| CPB1 |  |  |  |  |  |  |  |  |  |  |
| rs102275 | Diabetic polyneuropathy | 11 | 61557803 | T/C | 0.584 | 0.123 | 0.077 | 0.107 | 0.416 | trans |
| rs6914968 | Diabetic polyneuropathy | 6 | 127528104 | T/C | 0.594 | 0.109 | 0.077 | 0.155 | 0.406 | trans |
| rs507666 | Diabetic polyneuropathy | 9 | 136149399 | A/G | 0.201 | 0.019 | 0.094 | 0.840 | 0.201 | trans |
| rs72802342 | Diabetic polyneuropathy | 16 | 75234872 | A/C | 0.086 | -0.032 | 0.135 | 0.814 | 0.086 | trans |
| rs2291671 | Diabetic polyneuropathy | 3 | 148558447 | A/G | 0.226 | -0.042 | 0.091 | 0.645 | 0.226 | cis |
| SSC5D |  |  |  |  |  |  |  |  |  |  |
| rs13107325 | Diabetic polyneuropathy | 4 | 103188709 | T/C | 0.014 | 0.106 | 0.309 | 0.731 | 0.014 | trans |
| rs55799523 | Diabetic polyneuropathy | 19 | 55999545 | A/C | 0.281 | -0.001 | 0.085 | 0.991 | 0.281 | trans |
| rs10778118 | Diabetic polyneuropathy | 12 | 101824506 | T/C | 0.753 | -0.014 | 0.088 | 0.871 | 0.247 | trans |
| rs2142306 | Diabetic polyneuropathy | 8 | 134470631 | C/T | 0.375 | -0.079 | 0.078 | 0.311 | 0.375 | trans |
| CXCL12 |  |  |  |  |  |  |  |  |  |  |
| rs77542162 | Diabetic polyneuropathy | 17 | 67081278 | A/G | 0.993 | 0.407 | 0.465 | 0.382 | 0.007 | trans |
| rs1023264 | Diabetic polyneuropathy | 10 | 44893756 | T/C | 0.721 | 0.014 | 0.085 | 0.865 | 0.280 | cis |
| NUDCD3 |  |  |  |  |  |  |  |  |  |  |
| rs10233273 | Diabetic polyneuropathy | 7 | 44502186 | T/C | 0.203 | 0.187 | 0.093 | 0.045 | 0.203 | cis |
| rs1354034 | Diabetic polyneuropathy | 3 | 56849749 | C/T | 0.707 | 0.052 | 0.083 | 0.532 | 0.293 | trans |
| FOLR3 |  |  |  |  |  |  |  |  |  |  |
| rs12975366 | Diabetic polyneuropathy | 19 | 54759361 | C/T | 0.372 | 0.142 | 0.079 | 0.071 | 0.372 | trans |
| rs61729512 | Diabetic polyneuropathy | 12 | 7637769 | A/G | 0.152 | 0.101 | 0.107 | 0.348 | 0.152 | trans |
| rs4760 | Diabetic polyneuropathy | 19 | 44153100 | G/A | 0.168 | 0.087 | 0.103 | 0.396 | 0.168 | trans |
| rs333947 | Diabetic polyneuropathy | 1 | 110470764 | A/G | 0.167 | 0.083 | 0.102 | 0.420 | 0.167 | trans |
| rs10242866 | Diabetic polyneuropathy | 7 | 17920613 | T/C | 0.298 | 0.063 | 0.082 | 0.443 | 0.298 | trans |
| rs950802 | Diabetic polyneuropathy | 11 | 60152584 | A/G | 0.252 | 0.040 | 0.087 | 0.643 | 0.252 | trans |
| rs2031902 | Diabetic polyneuropathy | 9 | 33117524 | T/C | 0.624 | -0.117 | 0.078 | 0.136 | 0.376 | trans |
| rs603424 | Diabetic polyneuropathy | 10 | 102075479 | A/G | 0.117 | -0.211 | 0.118 | 0.075 | 0.117 | trans |
| NT5C3A |  |  |  |  |  |  |  |  |  |  |
| rs1354034 | Diabetic polyneuropathy | 3 | 56849749 | C/T | 0.707 | 0.052 | 0.083 | 0.532 | 0.293 | trans |
| rs17622656 | Diabetic polyneuropathy | 5 | 131820997 | A/G | 0.260 | -0.133 | 0.086 | 0.122 | 0.260 | trans |
| EGFL7 |  |  |  |  |  |  |  |  |  |  |
| rs55646096 | Diabetic polyneuropathy | 11 | 64918514 | T/C | 0.132 | 0.090 | 0.112 | 0.423 | 0.132 | trans |
| rs1434282 | Diabetic polyneuropathy | 1 | 199010721 | T/C | 0.711 | 0.030 | 0.083 | 0.720 | 0.290 | trans |
| rs823066 | Diabetic polyneuropathy | 1 | 205771173 | G/A | 0.948 | 0.029 | 0.169 | 0.862 | 0.053 | trans |
| rs74557797 | Diabetic polyneuropathy | 9 | 139550503 | T/G | 0.176 | 0.028 | 0.102 | 0.785 | 0.176 | cis |
| rs114694170 | Diabetic polyneuropathy | 5 | 88180196 | C/T | 0.056 | 0.015 | 0.163 | 0.926 | 0.056 | trans |
| rs78945826 | Diabetic polyneuropathy | 1 | 26057416 | C/A | 0.021 | -0.001 | 0.272 | 0.998 | 0.021 | trans |
| rs6796 | Diabetic polyneuropathy | 7 | 6502367 | C/T | 0.321 | -0.010 | 0.082 | 0.900 | 0.321 | trans |
| rs9635249 | Diabetic polyneuropathy | 14 | 65797784 | G/A | 0.800 | -0.020 | 0.094 | 0.833 | 0.201 | trans |
| rs8193003 | Diabetic polyneuropathy | 9 | 135865496 | C/T | 0.581 | -0.030 | 0.077 | 0.699 | 0.419 | trans |
| rs78909033 | Diabetic polyneuropathy | 2 | 241510903 | A/G | 0.131 | -0.058 | 0.112 | 0.603 | 0.131 | trans |
| rs892090 | Diabetic polyneuropathy | 19 | 55539072 | T/G | 0.121 | -0.126 | 0.115 | 0.272 | 0.121 | trans |
| rs10266182 | Diabetic polyneuropathy | 7 | 35747325 | A/G | 0.333 | -0.143 | 0.080 | 0.076 | 0.333 | trans |
| CD163 |  |  |  |  |  |  |  |  |  |  |
| rs11054859 | Diabetic polyneuropathy | 12 | 7769776 | A/G | 0.098 | 0.249 | 0.126 | 0.048 | 0.098 | cis |
| rs2099684 | Diabetic polyneuropathy | 1 | 161500130 | G/A | 0.285 | 0.137 | 0.084 | 0.104 | 0.285 | trans |
| rs217184 | Diabetic polyneuropathy | 16 | 72105965 | C/T | 0.214 | 0.113 | 0.092 | 0.218 | 0.214 | trans |
| rs62165726 | Diabetic polyneuropathy | 2 | 134966562 | A/C | 0.026 | 0.074 | 0.243 | 0.760 | 0.026 | trans |
| rs601338 | Diabetic polyneuropathy | 19 | 49206674 | G/A | 0.626 | 0.044 | 0.078 | 0.571 | 0.375 | trans |
| rs635634 | Diabetic polyneuropathy | 9 | 136155000 | T/C | 0.200 | 0.020 | 0.095 | 0.833 | 0.200 | trans |
| rs11923060 | Diabetic polyneuropathy | 3 | 186589390 | T/C | 0.171 | -0.018 | 0.101 | 0.857 | 0.171 | trans |
| rs11220505 | Diabetic polyneuropathy | 11 | 126334805 | T/C | 0.276 | -0.073 | 0.085 | 0.391 | 0.276 | trans |
| rs4876611 | Diabetic polyneuropathy | 8 | 116671848 | G/A | 0.691 | -0.104 | 0.082 | 0.206 | 0.309 | trans |
| rs2031902 | Diabetic polyneuropathy | 9 | 33117524 | T/C | 0.624 | -0.117 | 0.078 | 0.136 | 0.376 | trans |
| rs74622686 | Diabetic polyneuropathy | 11 | 134264227 | G/A | 0.124 | -0.134 | 0.115 | 0.245 | 0.124 | trans |
| TNFSF10 |  |  |  |  |  |  |  |  |  |  |
| rs4760 | Diabetic polyneuropathy | 19 | 44153100 | G/A | 0.168 | 0.087 | 0.103 | 0.396 | 0.168 | trans |
| rs1027315 | Diabetic polyneuropathy | 12 | 58034192 | C/T | 0.369 | 0.051 | 0.078 | 0.514 | 0.369 | trans |
| rs28929474 | Diabetic polyneuropathy | 14 | 94844947 | T/C | 0.020 | -0.002 | 0.272 | 0.993 | 0.020 | trans |
| rs79287178 | Diabetic polyneuropathy | 3 | 172294500 | A/G | 0.042 | -0.058 | 0.192 | 0.763 | 0.042 | trans |
| rs7519758 | Diabetic polyneuropathy | 1 | 196825287 | T/C | 0.131 | -0.094 | 0.109 | 0.387 | 0.131 | trans |
| rs174551 | Diabetic polyneuropathy | 11 | 61573684 | C/T | 0.411 | -0.117 | 0.077 | 0.127 | 0.411 | trans |
| rs4788460 | Diabetic polyneuropathy | 16 | 72154509 | T/C | 0.315 | -0.118 | 0.082 | 0.152 | 0.315 | trans |
| rs8178824 | Diabetic polyneuropathy | 17 | 64224775 | T/C | 0.009 | -0.140 | 0.389 | 0.718 | 0.009 | trans |
| RRM1 |  |  |  |  |  |  |  |  |  |  |
| rs73660574 | Diabetic polyneuropathy | 9 | 135860412 | A/G | 0.057 | 0.314 | 0.166 | 0.058 | 0.057 | trans |
| rs6017001 | Diabetic polyneuropathy | 20 | 35643946 | G/A | 0.235 | 0.107 | 0.090 | 0.232 | 0.235 | trans |
| rs10281146 | Diabetic polyneuropathy | 7 | 33035342 | G/A | 0.336 | 0.082 | 0.081 | 0.311 | 0.336 | trans |
| rs1427407 | Diabetic polyneuropathy | 2 | 60718043 | G/T | 0.848 | 0.076 | 0.105 | 0.469 | 0.152 | trans |
| rs230508 | Diabetic polyneuropathy | 4 | 103479279 | A/G | 0.350 | 0.048 | 0.079 | 0.547 | 0.350 | trans |
| rs68149176 | Diabetic polyneuropathy | 16 | 87886490 | T/C | 0.295 | -0.022 | 0.084 | 0.795 | 0.295 | trans |
| rs3811444 | Diabetic polyneuropathy | 1 | 248039451 | T/C | 0.346 | -0.037 | 0.080 | 0.645 | 0.346 | trans |
| rs6875334 | Diabetic polyneuropathy | 5 | 133547684 | A/G | 0.258 | -0.055 | 0.087 | 0.529 | 0.258 | trans |
| rs9609570 | Diabetic polyneuropathy | 22 | 32881600 | T/C | 0.211 | -0.093 | 0.093 | 0.319 | 0.211 | trans |
| rs58141407 | Diabetic polyneuropathy | 8 | 21791772 | T/C | 0.140 | -0.116 | 0.109 | 0.288 | 0.140 | trans |
| NOTCH3 |  |  |  |  |  |  |  |  |  |  |
| rs139974673 | Diabetic polyneuropathy | 15 | 44027885 | T/C | 0.995 | 0.444 | 0.516 | 0.389 | 0.005 | trans |
| rs77542162 | Diabetic polyneuropathy | 17 | 67081278 | A/G | 0.993 | 0.407 | 0.465 | 0.382 | 0.007 | trans |
| rs10282122 | Diabetic polyneuropathy | 7 | 2529623 | T/C | 0.740 | 0.031 | 0.086 | 0.721 | 0.260 | trans |
| rs507666 | Diabetic polyneuropathy | 9 | 136149399 | A/G | 0.201 | 0.019 | 0.094 | 0.840 | 0.201 | trans |
| rs28616221 | Diabetic polyneuropathy | 19 | 35554479 | A/G | 0.171 | -0.021 | 0.101 | 0.838 | 0.171 | trans |
| rs1260326 | Diabetic polyneuropathy | 2 | 27730940 | T/C | 0.351 | -0.100 | 0.079 | 0.208 | 0.351 | trans |
| MB |  |  |  |  |  |  |  |  |  |  |
| rs41155 | Diabetic polyneuropathy | 22 | 30404547 | C/A | 0.696 | 0.141 | 0.082 | 0.085 | 0.304 | trans |
| rs1799938 | Diabetic polyneuropathy | 17 | 65052304 | A/G | 0.187 | -0.023 | 0.096 | 0.811 | 0.187 | trans |
| rs12463674 | Diabetic polyneuropathy | 2 | 179432185 | G/A | 0.373 | -0.118 | 0.079 | 0.133 | 0.373 | trans |
| CBR3 |  |  |  |  |  |  |  |  |  |  |
| rs1028997 | Diabetic polyneuropathy | 21 | 37532222 | A/G | 0.304 | -0.024 | 0.082 | 0.769 | 0.304 | cis |
| LINGO1 |  |  |  |  |  |  |  |  |  |  |
| rs1467206 | Diabetic polyneuropathy | 4 | 82141894 | T/C | 0.233 | 0.042 | 0.089 | 0.641 | 0.233 | trans |
| rs215226 | Diabetic polyneuropathy | 12 | 591300 | G/A | 0.366 | 0.034 | 0.078 | 0.665 | 0.366 | trans |
| rs1033415 | Diabetic polyneuropathy | 22 | 39888774 | G/A | 0.710 | -0.036 | 0.083 | 0.669 | 0.290 | trans |
| rs10762694 | Diabetic polyneuropathy | 10 | 77886953 | A/C | 0.283 | -0.070 | 0.084 | 0.402 | 0.283 | trans |
| CNTNAP2 |  |  |  |  |  |  |  |  |  |  |
| rs7810370 | Diabetic polyneuropathy | 7 | 145366002 | G/A | 0.726 | 0.115 | 0.085 | 0.176 | 0.274 | cis |
| rs4665972 | Diabetic polyneuropathy | 2 | 27598097 | C/T | 0.623 | 0.080 | 0.078 | 0.303 | 0.377 | trans |
| rs56278466 | Diabetic polyneuropathy | 10 | 17875857 | G/T | 0.581 | -0.003 | 0.077 | 0.970 | 0.419 | trans |
| rs1033415 | Diabetic polyneuropathy | 22 | 39888774 | G/A | 0.710 | -0.036 | 0.083 | 0.669 | 0.290 | trans |
| PZP |  |  |  |  |  |  |  |  |  |  |
| rs1035849 | Diabetic polyneuropathy | 12 | 9362168 | T/C | 0.326 | 0.117 | 0.081 | 0.146 | 0.326 | cis |
| COCH |  |  |  |  |  |  |  |  |  |  |
| rs28400019 | Diabetic polyneuropathy | 14 | 31343494 | A/G | 0.107 | 0.023 | 0.124 | 0.855 | 0.107 | cis |
| rs114694170 | Diabetic polyneuropathy | 5 | 88180196 | C/T | 0.056 | 0.015 | 0.163 | 0.926 | 0.056 | trans |
| rs9792084 | Diabetic polyneuropathy | 7 | 129244665 | T/C | 0.102 | -0.042 | 0.125 | 0.738 | 0.102 | trans |
| rs1036332 | Diabetic polyneuropathy | 1 | 199012478 | A/C | 0.278 | -0.051 | 0.084 | 0.542 | 0.278 | trans |
| rs6580981 | Diabetic polyneuropathy | 12 | 54723028 | A/G | 0.582 | -0.054 | 0.077 | 0.484 | 0.419 | trans |
| LTBP2 |  |  |  |  |  |  |  |  |  |  |
| rs333947 | Diabetic polyneuropathy | 1 | 110470764 | A/G | 0.167 | 0.083 | 0.102 | 0.420 | 0.167 | trans |
| rs1036477 | Diabetic polyneuropathy | 15 | 48914926 | G/A | 0.079 | 0.072 | 0.139 | 0.604 | 0.079 | trans |
| rs6973480 | Diabetic polyneuropathy | 7 | 17978129 | T/C | 0.600 | -0.036 | 0.077 | 0.645 | 0.400 | trans |
| rs888414 | Diabetic polyneuropathy | 14 | 75104905 | A/G | 0.288 | -0.063 | 0.083 | 0.449 | 0.288 | cis |
| LGMN |  |  |  |  |  |  |  |  |  |  |
| rs6848819 | Diabetic polyneuropathy | 4 | 154451678 | C/A | 0.014 | 0.372 | 0.307 | 0.226 | 0.014 | trans |
| rs1654425 | Diabetic polyneuropathy | 19 | 55538980 | C/T | 0.879 | 0.126 | 0.115 | 0.272 | 0.121 | trans |
| rs12767683 | Diabetic polyneuropathy | 10 | 104316581 | A/C | 0.296 | 0.103 | 0.083 | 0.215 | 0.296 | trans |
| rs55976836 | Diabetic polyneuropathy | 19 | 48774683 | A/G | 0.246 | 0.089 | 0.088 | 0.313 | 0.246 | trans |
| rs3026096 | Diabetic polyneuropathy | 17 | 5275918 | A/G | 0.642 | 0.037 | 0.079 | 0.636 | 0.358 | trans |
| rs823078 | Diabetic polyneuropathy | 1 | 205776458 | C/T | 0.948 | 0.029 | 0.169 | 0.863 | 0.053 | trans |
| rs4734879 | Diabetic polyneuropathy | 8 | 106583124 | A/G | 0.780 | 0.005 | 0.091 | 0.959 | 0.220 | trans |
| rs2250645 | Diabetic polyneuropathy | 10 | 91008873 | T/C | 0.350 | -0.013 | 0.079 | 0.866 | 0.350 | trans |
| rs7870723 | Diabetic polyneuropathy | 9 | 100699242 | C/T | 0.398 | -0.030 | 0.077 | 0.703 | 0.398 | trans |
| rs600038 | Diabetic polyneuropathy | 9 | 136151806 | C/T | 0.222 | -0.038 | 0.091 | 0.674 | 0.222 | trans |
| rs35677470 | Diabetic polyneuropathy | 3 | 58183636 | A/G | 0.061 | -0.075 | 0.157 | 0.635 | 0.061 | trans |
| rs6986384 | Diabetic polyneuropathy | 8 | 124832181 | A/G | 0.073 | -0.116 | 0.146 | 0.428 | 0.073 | trans |
| rs35340377 | Diabetic polyneuropathy | 1 | 248038210 | A/G | 0.291 | -0.124 | 0.083 | 0.137 | 0.291 | trans |
| rs17154130 | Diabetic polyneuropathy | 7 | 80229484 | C/T | 0.069 | -0.223 | 0.150 | 0.136 | 0.069 | trans |
| rs148659834 | Diabetic polyneuropathy | 14 | 93176042 | A/G | 0.015 | -0.250 | 0.319 | 0.433 | 0.015 | cis |
| SEMA3F |  |  |  |  |  |  |  |  |  |  |
| rs4679317 | Diabetic polyneuropathy | 3 | 126688271 | A/G | 0.691 | 0.299 | 0.082 | 0.000 | 0.309 | trans |
| rs507666 | Diabetic polyneuropathy | 9 | 136149399 | A/G | 0.201 | 0.019 | 0.094 | 0.840 | 0.201 | trans |
| rs3184504 | Diabetic polyneuropathy | 12 | 111884608 | C/T | 0.591 | -0.051 | 0.077 | 0.504 | 0.409 | trans |
| rs1037117 | Diabetic polyneuropathy | 15 | 102068658 | A/G | 0.285 | -0.102 | 0.084 | 0.224 | 0.285 | trans |
| KAAG1 |  |  |  |  |  |  |  |  |  |  |
| rs72550870 | Diabetic polyneuropathy | 1 | 11106666 | C/T | 0.046 | 0.303 | 0.184 | 0.100 | 0.046 | trans |
| rs1800450 | Diabetic polyneuropathy | 10 | 54531235 | T/C | 0.132 | 0.073 | 0.111 | 0.512 | 0.132 | trans |
| rs698088 | Diabetic polyneuropathy | 3 | 186958968 | G/A | 0.357 | -0.015 | 0.079 | 0.849 | 0.357 | trans |
| rs1260326 | Diabetic polyneuropathy | 2 | 27730940 | T/C | 0.351 | -0.100 | 0.079 | 0.208 | 0.351 | trans |
| MET |  |  |  |  |  |  |  |  |  |  |
| rs77542162 | Diabetic polyneuropathy | 17 | 67081278 | A/G | 0.993 | 0.407 | 0.465 | 0.382 | 0.007 | trans |
| rs11045819 | Diabetic polyneuropathy | 12 | 21329813 | A/C | 0.112 | 0.186 | 0.119 | 0.118 | 0.112 | trans |
| rs112167630 | Diabetic polyneuropathy | 19 | 42874901 | A/G | 0.035 | 0.178 | 0.207 | 0.390 | 0.035 | trans |
| rs4850 | Diabetic polyneuropathy | 16 | 21976762 | A/G | 0.059 | 0.102 | 0.161 | 0.524 | 0.059 | trans |
| rs1041316 | Diabetic polyneuropathy | 14 | 57099859 | A/G | 0.722 | 0.060 | 0.085 | 0.479 | 0.278 | trans |
| rs79094524 | Diabetic polyneuropathy | 1 | 40041371 | GA/G | 0.217 | 0.039 | 0.092 | 0.672 | 0.217 | trans |
| rs4855845 | Diabetic polyneuropathy | 3 | 49687043 | C/T | 0.891 | 0.029 | 0.120 | 0.812 | 0.109 | trans |
| rs5398 | Diabetic polyneuropathy | 3 | 170715830 | A/G | 0.263 | 0.021 | 0.085 | 0.806 | 0.263 | trans |
| rs507666 | Diabetic polyneuropathy | 9 | 136149399 | A/G | 0.201 | 0.019 | 0.094 | 0.840 | 0.201 | trans |
| rs10748526 | Diabetic polyneuropathy | 10 | 82273079 | C/T | 0.720 | 0.004 | 0.084 | 0.967 | 0.280 | trans |
| rs28929474 | Diabetic polyneuropathy | 14 | 94844947 | T/C | 0.020 | -0.002 | 0.272 | 0.993 | 0.020 | trans |
| rs10401969 | Diabetic polyneuropathy | 19 | 19407718 | C/T | 0.064 | -0.006 | 0.154 | 0.970 | 0.064 | trans |
| rs1982784 | Diabetic polyneuropathy | 16 | 4666537 | G/A | 0.831 | -0.043 | 0.100 | 0.669 | 0.170 | trans |
| rs174547 | Diabetic polyneuropathy | 11 | 61570783 | C/T | 0.414 | -0.120 | 0.077 | 0.119 | 0.414 | trans |
| CRIP1 |  |  |  |  |  |  |  |  |  |  |
| rs58542926 | Diabetic polyneuropathy | 19 | 19379549 | T/C | 0.064 | 0.005 | 0.153 | 0.977 | 0.064 | trans |
| GZMA |  |  |  |  |  |  |  |  |  |  |
| rs10402422 | Diabetic polyneuropathy | 19 | 16475388 | A/G | 0.641 | 0.138 | 0.079 | 0.081 | 0.359 | trans |
| rs2047745 | Diabetic polyneuropathy | 5 | 54489541 | C/T | 0.202 | 0.088 | 0.095 | 0.352 | 0.202 | cis |
| rs3846730 | Diabetic polyneuropathy | 5 | 131806780 | T/C | 0.306 | 0.078 | 0.082 | 0.346 | 0.306 | trans |
| rs75587749 | Diabetic polyneuropathy | 12 | 7614940 | T/C | 0.078 | 0.071 | 0.139 | 0.612 | 0.078 | trans |
| rs8177652 | Diabetic polyneuropathy | 10 | 6015384 | A/G | 0.314 | 0.066 | 0.082 | 0.421 | 0.314 | trans |
| rs6476398 | Diabetic polyneuropathy | 9 | 33119241 | T/C | 0.261 | -0.013 | 0.086 | 0.876 | 0.261 | trans |
| rs230507 | Diabetic polyneuropathy | 4 | 103480246 | A/C | 0.636 | -0.037 | 0.079 | 0.641 | 0.364 | trans |
| rs3184504 | Diabetic polyneuropathy | 12 | 111884608 | C/T | 0.591 | -0.051 | 0.077 | 0.504 | 0.409 | trans |
| rs41280225 | Diabetic polyneuropathy | 9 | 116023979 | C/A | 0.065 | -0.122 | 0.153 | 0.427 | 0.065 | trans |
| SEMA6B |  |  |  |  |  |  |  |  |  |  |
| rs8176743 | Diabetic polyneuropathy | 9 | 136131415 | T/C | 0.134 | 0.137 | 0.111 | 0.218 | 0.134 | trans |
| rs55954186 | Diabetic polyneuropathy | 3 | 58436476 | A/G | 0.247 | 0.046 | 0.088 | 0.596 | 0.247 | trans |
| rs10404223 | Diabetic polyneuropathy | 19 | 4549646 | A/G | 0.372 | 0.026 | 0.080 | 0.742 | 0.372 | cis |
| rs3184504 | Diabetic polyneuropathy | 12 | 111884608 | C/T | 0.591 | -0.051 | 0.077 | 0.504 | 0.409 | trans |
| SERPINB13 |  |  |  |  |  |  |  |  |  |  |
| rs10405357 | Diabetic polyneuropathy | 19 | 54759666 | C/T | 0.397 | 0.113 | 0.078 | 0.146 | 0.397 | trans |
| rs492602 | Diabetic polyneuropathy | 19 | 49206417 | A/G | 0.625 | 0.045 | 0.078 | 0.566 | 0.375 | trans |
| rs1260326 | Diabetic polyneuropathy | 2 | 27730940 | T/C | 0.351 | -0.100 | 0.079 | 0.208 | 0.351 | trans |
| rs77157727 | Diabetic polyneuropathy | 18 | 61253961 | A/G | 0.014 | -0.158 | 0.318 | 0.620 | 0.014 | cis |
| CKM_CKB |  |  |  |  |  |  |  |  |  |  |
| rs11559024 | Diabetic polyneuropathy | 19 | 45821183 | C/T | 0.019 | 1.124 | 0.305 | 0.000 | 0.019 | cis |
| rs6488388 | Diabetic polyneuropathy | 12 | 7663461 | T/C | 0.073 | 0.221 | 0.145 | 0.128 | 0.073 | trans |
| rs10405357 | Diabetic polyneuropathy | 19 | 54759666 | C/T | 0.397 | 0.113 | 0.078 | 0.146 | 0.397 | trans |
| PCOLCE2 |  |  |  |  |  |  |  |  |  |  |
| rs10405357 | Diabetic polyneuropathy | 19 | 54759666 | C/T | 0.397 | 0.113 | 0.078 | 0.146 | 0.397 | trans |
| rs653178 | Diabetic polyneuropathy | 12 | 112007756 | T/C | 0.584 | -0.054 | 0.077 | 0.477 | 0.416 | trans |
| rs11716897 | Diabetic polyneuropathy | 3 | 142605556 | A/G | 0.379 | -0.056 | 0.078 | 0.469 | 0.379 | cis |
| rs117755721 | Diabetic polyneuropathy | 17 | 11881356 | A/G | 0.033 | -0.122 | 0.212 | 0.564 | 0.033 | trans |
| AGXT |  |  |  |  |  |  |  |  |  |  |
| rs10405357 | Diabetic polyneuropathy | 19 | 54759666 | C/T | 0.397 | 0.113 | 0.078 | 0.146 | 0.397 | trans |
| rs55649245 | Diabetic polyneuropathy | 2 | 241793545 | A/G | 0.314 | 0.079 | 0.082 | 0.335 | 0.314 | cis |
| rs6888304 | Diabetic polyneuropathy | 5 | 31020521 | G/A | 0.322 | 0.034 | 0.081 | 0.674 | 0.322 | trans |
| rs9959832 | Diabetic polyneuropathy | 18 | 56086820 | T/C | 0.767 | 0.015 | 0.090 | 0.867 | 0.233 | trans |
| rs28929474 | Diabetic polyneuropathy | 14 | 94844947 | T/C | 0.020 | -0.002 | 0.272 | 0.993 | 0.020 | trans |
| rs3747207 | Diabetic polyneuropathy | 22 | 44324855 | A/G | 0.226 | -0.010 | 0.090 | 0.915 | 0.226 | trans |
| rs4835265 | Diabetic polyneuropathy | 4 | 146821410 | A/C | 0.172 | -0.047 | 0.101 | 0.641 | 0.172 | trans |
| rs10883451 | Diabetic polyneuropathy | 10 | 101924418 | C/T | 0.389 | -0.058 | 0.077 | 0.456 | 0.389 | trans |
| rs112875651 | Diabetic polyneuropathy | 8 | 126506694 | A/G | 0.387 | -0.058 | 0.078 | 0.456 | 0.387 | trans |
| rs1497406 | Diabetic polyneuropathy | 1 | 16505320 | G/A | 0.682 | -0.071 | 0.081 | 0.379 | 0.318 | trans |
| FBP1 |  |  |  |  |  |  |  |  |  |  |
| rs499206 | Diabetic polyneuropathy | 9 | 97359479 | G/A | 0.946 | 0.176 | 0.168 | 0.295 | 0.054 | cis |
| rs10405357 | Diabetic polyneuropathy | 19 | 54759666 | C/T | 0.397 | 0.113 | 0.078 | 0.146 | 0.397 | trans |
| rs145920606 | Diabetic polyneuropathy | 12 | 7627311 | G/A | 0.078 | 0.071 | 0.139 | 0.610 | 0.078 | trans |
| rs10883451 | Diabetic polyneuropathy | 10 | 101924418 | C/T | 0.389 | -0.058 | 0.077 | 0.456 | 0.389 | trans |
| TMEM70 |  |  |  |  |  |  |  |  |  |  |
| rs10406069 | Diabetic polyneuropathy | 19 | 35836530 | A/G | 0.188 | 0.068 | 0.096 | 0.483 | 0.188 | trans |
| rs2317231 | Diabetic polyneuropathy | 1 | 157686337 | T/G | 0.415 | 0.060 | 0.077 | 0.437 | 0.415 | trans |
| rs16864367 | Diabetic polyneuropathy | 3 | 152234166 | A/G | 0.302 | -0.010 | 0.082 | 0.906 | 0.302 | trans |
| ELANE |  |  |  |  |  |  |  |  |  |  |
| rs116855556 | Diabetic polyneuropathy | 9 | 82223657 | T/C | 0.053 | 0.236 | 0.171 | 0.167 | 0.053 | trans |
| rs12903325 | Diabetic polyneuropathy | 15 | 50353277 | G/T | 0.224 | 0.151 | 0.091 | 0.095 | 0.224 | trans |
| rs6605578 | Diabetic polyneuropathy | 8 | 6880963 | T/C | 0.408 | 0.019 | 0.077 | 0.809 | 0.408 | trans |
| CD22 |  |  |  |  |  |  |  |  |  |  |
| rs76428106 | Diabetic polyneuropathy | 13 | 28604007 | C/T | 0.012 | 0.322 | 0.347 | 0.353 | 0.012 | trans |
| rs12554596 | Diabetic polyneuropathy | 9 | 37002142 | G/A | 0.127 | 0.060 | 0.113 | 0.595 | 0.127 | trans |
| rs4848370 | Diabetic polyneuropathy | 2 | 111811665 | T/C | 0.271 | 0.057 | 0.085 | 0.505 | 0.271 | trans |
| rs9939427 | Diabetic polyneuropathy | 16 | 86016091 | A/G | 0.195 | 0.047 | 0.096 | 0.624 | 0.195 | trans |
| rs492602 | Diabetic polyneuropathy | 19 | 49206417 | A/G | 0.625 | 0.045 | 0.078 | 0.566 | 0.375 | trans |
| rs10411704 | Diabetic polyneuropathy | 19 | 35800662 | T/G | 0.749 | -0.025 | 0.087 | 0.776 | 0.251 | cis |
| rs116968179 | Diabetic polyneuropathy | 7 | 50189183 | A/G | 0.024 | -0.049 | 0.245 | 0.842 | 0.024 | trans |
| rs967367 | Diabetic polyneuropathy | 3 | 186734466 | A/G | 0.395 | -0.067 | 0.077 | 0.389 | 0.395 | trans |
| rs6065926 | Diabetic polyneuropathy | 20 | 44735854 | G/A | 0.727 | -0.070 | 0.085 | 0.411 | 0.273 | trans |
| rs304154 | Diabetic polyneuropathy | 5 | 88120416 | C/T | 0.324 | -0.091 | 0.081 | 0.259 | 0.324 | trans |
| rs12784975 | Diabetic polyneuropathy | 10 | 98380137 | C/T | 0.116 | -0.128 | 0.118 | 0.278 | 0.116 | trans |
| rs11223794 | Diabetic polyneuropathy | 11 | 134287396 | T/C | 0.150 | -0.202 | 0.106 | 0.057 | 0.150 | trans |
| rs112824187 | Diabetic polyneuropathy | 5 | 158265308 | A/G | 0.056 | -0.219 | 0.167 | 0.188 | 0.056 | trans |
| FSTL1 |  |  |  |  |  |  |  |  |  |  |
| rs10418205 | Diabetic polyneuropathy | 19 | 6100433 | A/G | 0.708 | 0.089 | 0.083 | 0.284 | 0.292 | trans |
| rs9302635 | Diabetic polyneuropathy | 16 | 72144174 | C/T | 0.174 | -0.069 | 0.099 | 0.482 | 0.174 | trans |
| rs1147707 | Diabetic polyneuropathy | 3 | 120169248 | T/C | 0.417 | -0.114 | 0.077 | 0.138 | 0.417 | cis |
| HYOU1 |  |  |  |  |  |  |  |  |  |  |
| rs4965806 | Diabetic polyneuropathy | 15 | 101752435 | A/G | 0.403 | 0.106 | 0.077 | 0.167 | 0.403 | trans |
| rs10419198 | Diabetic polyneuropathy | 19 | 50038017 | T/C | 0.262 | 0.064 | 0.086 | 0.456 | 0.262 | trans |
| rs33950747 | Diabetic polyneuropathy | 19 | 36339247 | T/C | 0.086 | 0.042 | 0.137 | 0.757 | 0.086 | trans |
| rs28929474 | Diabetic polyneuropathy | 14 | 94844947 | T/C | 0.020 | -0.002 | 0.272 | 0.993 | 0.020 | trans |
| rs74676624 | Diabetic polyneuropathy | 7 | 127100039 | C/T | 0.054 | -0.036 | 0.164 | 0.828 | 0.054 | trans |
| rs2509121 | Diabetic polyneuropathy | 11 | 118928253 | T/C | 0.293 | -0.061 | 0.083 | 0.458 | 0.293 | cis |
| rs1801689 | Diabetic polyneuropathy | 17 | 64210580 | C/A | 0.010 | -0.158 | 0.383 | 0.681 | 0.010 | trans |
| rs61747728 | Diabetic polyneuropathy | 1 | 179526214 | T/C | 0.064 | -0.201 | 0.158 | 0.204 | 0.064 | trans |
| rs75166367 | Diabetic polyneuropathy | 2 | 162964301 | A/G | 0.071 | -0.204 | 0.149 | 0.170 | 0.071 | trans |
| rs149547076 | Diabetic polyneuropathy | 3 | 57557444 | C/T | 0.012 | -0.568 | 0.339 | 0.094 | 0.012 | trans |
| CYP3A4 |  |  |  |  |  |  |  |  |  |  |
| rs1042031 | Diabetic polyneuropathy | 2 | 21225753 | T/C | 0.198 | -0.106 | 0.095 | 0.268 | 0.198 | trans |
| C1QBP |  |  |  |  |  |  |  |  |  |  |
| rs1042303 | Diabetic polyneuropathy | 6 | 24437458 | T/C | 0.610 | 0.026 | 0.078 | 0.735 | 0.390 | trans |
| rs78119247 | Diabetic polyneuropathy | 3 | 165499135 | G/GAT | 0.173 | -0.017 | 0.100 | 0.866 | 0.173 | trans |
| rs1126605 | Diabetic polyneuropathy | 12 | 7242204 | T/C | 0.079 | -0.176 | 0.138 | 0.204 | 0.079 | trans |
| IRF3 |  |  |  |  |  |  |  |  |  |  |
| rs1354034 | Diabetic polyneuropathy | 3 | 56849749 | C/T | 0.707 | 0.052 | 0.083 | 0.532 | 0.293 | trans |
| rs10423580 | Diabetic polyneuropathy | 19 | 50195771 | T/G | 0.615 | -0.030 | 0.078 | 0.703 | 0.385 | cis |
| PCDH10 |  |  |  |  |  |  |  |  |  |  |
| rs704 | Diabetic polyneuropathy | 17 | 26694861 | A/G | 0.420 | 0.049 | 0.076 | 0.519 | 0.420 | trans |
| rs1042445 | Diabetic polyneuropathy | 3 | 186395436 | T/C | 0.279 | 0.029 | 0.084 | 0.728 | 0.279 | trans |
| LMAN2 |  |  |  |  |  |  |  |  |  |  |
| rs4665972 | Diabetic polyneuropathy | 2 | 27598097 | C/T | 0.623 | 0.080 | 0.078 | 0.303 | 0.377 | trans |
| rs1042445 | Diabetic polyneuropathy | 3 | 186395436 | T/C | 0.279 | 0.029 | 0.084 | 0.728 | 0.279 | trans |
| CRTAC1 |  |  |  |  |  |  |  |  |  |  |
| rs1042445 | Diabetic polyneuropathy | 3 | 186395436 | T/C | 0.279 | 0.029 | 0.084 | 0.728 | 0.279 | trans |
| rs56007204 | Diabetic polyneuropathy | 10 | 99625319 | T/C | 0.163 | -0.033 | 0.103 | 0.748 | 0.163 | cis |
| ELAPOR2 |  |  |  |  |  |  |  |  |  |  |
| rs1042445 | Diabetic polyneuropathy | 3 | 186395436 | T/C | 0.279 | 0.029 | 0.084 | 0.728 | 0.279 | trans |
| BNIP3 |  |  |  |  |  |  |  |  |  |  |
| rs1354034 | Diabetic polyneuropathy | 3 | 56849749 | C/T | 0.707 | 0.052 | 0.083 | 0.532 | 0.293 | trans |
| rs1042445 | Diabetic polyneuropathy | 3 | 186395436 | T/C | 0.279 | 0.029 | 0.084 | 0.728 | 0.279 | trans |
| KIAA1324L |  |  |  |  |  |  |  |  |  |  |
| rs1042445 | Diabetic polyneuropathy | 3 | 186395436 | T/C | 0.279 | 0.029 | 0.084 | 0.728 | 0.279 | trans |
| MYL7 |  |  |  |  |  |  |  |  |  |  |
| rs1042445 | Diabetic polyneuropathy | 3 | 186395436 | T/C | 0.279 | 0.029 | 0.084 | 0.728 | 0.279 | trans |
| HMGN1 |  |  |  |  |  |  |  |  |  |  |
| rs1042445 | Diabetic polyneuropathy | 3 | 186395436 | T/C | 0.279 | 0.029 | 0.084 | 0.728 | 0.279 | trans |
| MPZ |  |  |  |  |  |  |  |  |  |  |
| rs117801489 | Diabetic polyneuropathy | 12 | 104408832 | T/C | 0.960 | 0.197 | 0.195 | 0.312 | 0.040 | trans |
| rs13107325 | Diabetic polyneuropathy | 4 | 103188709 | T/C | 0.014 | 0.106 | 0.309 | 0.731 | 0.014 | trans |
| rs1042445 | Diabetic polyneuropathy | 3 | 186395436 | T/C | 0.279 | 0.029 | 0.084 | 0.728 | 0.279 | trans |
| rs3217964 | Diabetic polyneuropathy | 7 | 44157825 | C/T | 0.211 | -0.157 | 0.093 | 0.092 | 0.211 | trans |
| RIPK2 |  |  |  |  |  |  |  |  |  |  |
| rs8100411 | Diabetic polyneuropathy | 19 | 35743263 | T/C | 0.901 | 0.145 | 0.126 | 0.250 | 0.099 | trans |
| rs1354034 | Diabetic polyneuropathy | 3 | 56849749 | C/T | 0.707 | 0.052 | 0.083 | 0.532 | 0.293 | trans |
| rs1042445 | Diabetic polyneuropathy | 3 | 186395436 | T/C | 0.279 | 0.029 | 0.084 | 0.728 | 0.279 | trans |
| rs17713196 | Diabetic polyneuropathy | 3 | 165489724 | T/C | 0.174 | -0.015 | 0.099 | 0.878 | 0.174 | trans |
| ESR1 |  |  |  |  |  |  |  |  |  |  |
| rs1042445 | Diabetic polyneuropathy | 3 | 186395436 | T/C | 0.279 | 0.029 | 0.084 | 0.728 | 0.279 | trans |
| CCN4 |  |  |  |  |  |  |  |  |  |  |
| rs1654425 | Diabetic polyneuropathy | 19 | 55538980 | C/T | 0.879 | 0.126 | 0.115 | 0.272 | 0.121 | trans |
| rs11591571 | Diabetic polyneuropathy | 10 | 104342804 | A/G | 0.300 | 0.084 | 0.082 | 0.309 | 0.300 | trans |
| rs1354034 | Diabetic polyneuropathy | 3 | 56849749 | C/T | 0.707 | 0.052 | 0.083 | 0.532 | 0.293 | trans |
| rs2274319 | Diabetic polyneuropathy | 1 | 156450873 | C/T | 0.581 | 0.047 | 0.076 | 0.542 | 0.419 | trans |
| rs1868715 | Diabetic polyneuropathy | 4 | 121747970 | T/C | 0.317 | 0.034 | 0.081 | 0.675 | 0.317 | trans |
| rs114694170 | Diabetic polyneuropathy | 5 | 88180196 | C/T | 0.056 | 0.015 | 0.163 | 0.926 | 0.056 | trans |
| rs12156037 | Diabetic polyneuropathy | 8 | 134204458 | G/A | 0.418 | 0.011 | 0.077 | 0.885 | 0.418 | cis |
| rs4541868 | Diabetic polyneuropathy | 8 | 106590705 | A/C | 0.226 | -0.001 | 0.090 | 0.990 | 0.226 | trans |
| rs10424665 | Diabetic polyneuropathy | 19 | 32895631 | C/A | 0.216 | -0.015 | 0.092 | 0.868 | 0.216 | trans |
| rs68066031 | Diabetic polyneuropathy | 2 | 224880498 | C/T | 0.204 | -0.200 | 0.095 | 0.036 | 0.204 | trans |
| IFNL1 |  |  |  |  |  |  |  |  |  |  |
| rs28668750 | Diabetic polyneuropathy | 19 | 39819802 | T/G | 0.110 | 0.115 | 0.121 | 0.345 | 0.110 | cis |
| rs10424978 | Diabetic polyneuropathy | 19 | 4837557 | A/C | 0.599 | 0.028 | 0.077 | 0.719 | 0.401 | trans |
| rs3775291 | Diabetic polyneuropathy | 4 | 187004074 | T/C | 0.315 | -0.077 | 0.081 | 0.342 | 0.315 | trans |
| rs35667974 | Diabetic polyneuropathy | 2 | 163124637 | C/T | 0.019 | -0.352 | 0.279 | 0.207 | 0.019 | trans |
| ANPEP |  |  |  |  |  |  |  |  |  |  |
| rs13135092 | Diabetic polyneuropathy | 4 | 103198082 | G/A | 0.018 | 0.142 | 0.281 | 0.612 | 0.018 | trans |
| rs12373325 | Diabetic polyneuropathy | 18 | 56087648 | C/T | 0.767 | 0.015 | 0.090 | 0.866 | 0.233 | trans |
| rs4835265 | Diabetic polyneuropathy | 4 | 146821410 | A/C | 0.172 | -0.047 | 0.101 | 0.641 | 0.172 | trans |
| rs4055121 | Diabetic polyneuropathy | 11 | 126232337 | T/C | 0.153 | -0.048 | 0.106 | 0.651 | 0.153 | trans |
| rs1042499 | Diabetic polyneuropathy | 15 | 90328305 | C/T | 0.030 | -0.137 | 0.219 | 0.531 | 0.030 | cis |
| THBD |  |  |  |  |  |  |  |  |  |  |
| rs1042579 | Diabetic polyneuropathy | 20 | 23028724 | A/G | 0.278 | 0.158 | 0.085 | 0.063 | 0.278 | cis |
| rs71354106 | Diabetic polyneuropathy | 19 | 36329387 | A/G | 0.086 | 0.045 | 0.137 | 0.741 | 0.086 | trans |
| rs61747728 | Diabetic polyneuropathy | 1 | 179526214 | T/C | 0.064 | -0.201 | 0.158 | 0.204 | 0.064 | trans |
| PLXNA1 |  |  |  |  |  |  |  |  |  |  |
| rs1042704 | Diabetic polyneuropathy | 14 | 23312594 | A/G | 0.209 | -0.006 | 0.094 | 0.947 | 0.209 | trans |
| rs1260326 | Diabetic polyneuropathy | 2 | 27730940 | T/C | 0.351 | -0.100 | 0.079 | 0.208 | 0.351 | trans |
| rs4679138 | Diabetic polyneuropathy | 3 | 126711070 | G/A | 0.307 | -0.319 | 0.082 | 0.000 | 0.307 | cis |
| CD276 |  |  |  |  |  |  |  |  |  |  |
| rs4665972 | Diabetic polyneuropathy | 2 | 27598097 | C/T | 0.623 | 0.080 | 0.078 | 0.303 | 0.377 | trans |
| rs2291014 | Diabetic polyneuropathy | 15 | 73996359 | A/G | 0.124 | 0.046 | 0.116 | 0.690 | 0.124 | cis |
| rs2239651 | Diabetic polyneuropathy | 14 | 94848547 | C/T | 0.247 | 0.043 | 0.088 | 0.627 | 0.247 | trans |
| rs1042704 | Diabetic polyneuropathy | 14 | 23312594 | A/G | 0.209 | -0.006 | 0.094 | 0.947 | 0.209 | trans |
| AOC3 |  |  |  |  |  |  |  |  |  |  |
| rs33986943 | Diabetic polyneuropathy | 17 | 41004637 | A/G | 0.071 | 0.157 | 0.145 | 0.278 | 0.071 | cis |
| rs13135092 | Diabetic polyneuropathy | 4 | 103198082 | G/A | 0.018 | 0.142 | 0.281 | 0.612 | 0.018 | trans |
| rs56278466 | Diabetic polyneuropathy | 10 | 17875857 | G/T | 0.581 | -0.003 | 0.077 | 0.970 | 0.419 | trans |
| rs1042704 | Diabetic polyneuropathy | 14 | 23312594 | A/G | 0.209 | -0.006 | 0.094 | 0.947 | 0.209 | trans |
| rs767298 | Diabetic polyneuropathy | 7 | 7274584 | T/G | 0.638 | -0.011 | 0.079 | 0.889 | 0.362 | trans |
| rs12913657 | Diabetic polyneuropathy | 15 | 51000968 | C/T | 0.136 | -0.025 | 0.110 | 0.819 | 0.136 | trans |
| rs3967200 | Diabetic polyneuropathy | 11 | 126232385 | T/C | 0.153 | -0.048 | 0.106 | 0.651 | 0.153 | trans |
| JAKMIP3 |  |  |  |  |  |  |  |  |  |  |
| rs1042779 | Diabetic polyneuropathy | 3 | 52821011 | G/A | 0.377 | -0.032 | 0.078 | 0.685 | 0.377 | trans |
| ZNF334 |  |  |  |  |  |  |  |  |  |  |
| rs1042779 | Diabetic polyneuropathy | 3 | 52821011 | G/A | 0.377 | -0.032 | 0.078 | 0.685 | 0.377 | trans |
| SIGIRR |  |  |  |  |  |  |  |  |  |  |
| rs1056522 | Diabetic polyneuropathy | 3 | 126261345 | A/G | 0.320 | 0.073 | 0.081 | 0.369 | 0.320 | trans |
| rs1042779 | Diabetic polyneuropathy | 3 | 52821011 | G/A | 0.377 | -0.032 | 0.078 | 0.685 | 0.377 | trans |
| SCN2B |  |  |  |  |  |  |  |  |  |  |
| rs1042779 | Diabetic polyneuropathy | 3 | 52821011 | G/A | 0.377 | -0.032 | 0.078 | 0.685 | 0.377 | trans |
| RNF146 |  |  |  |  |  |  |  |  |  |  |
| rs1042779 | Diabetic polyneuropathy | 3 | 52821011 | G/A | 0.377 | -0.032 | 0.078 | 0.685 | 0.377 | trans |
| rs6602268 | Diabetic polyneuropathy | 10 | 7764233 | A/G | 0.937 | -0.092 | 0.155 | 0.554 | 0.063 | trans |
| CORO1A |  |  |  |  |  |  |  |  |  |  |
| rs12292693 | Diabetic polyneuropathy | 11 | 64936719 | A/C | 0.758 | 0.144 | 0.089 | 0.104 | 0.242 | trans |
| rs10432210 | Diabetic polyneuropathy | 18 | 22861794 | T/C | 0.181 | 0.048 | 0.098 | 0.624 | 0.181 | trans |
| SEPTIN9 |  |  |  |  |  |  |  |  |  |  |
| rs10432210 | Diabetic polyneuropathy | 18 | 22861794 | T/C | 0.181 | 0.048 | 0.098 | 0.624 | 0.181 | trans |
| ARHGEF25 |  |  |  |  |  |  |  |  |  |  |
| rs10437954 | Diabetic polyneuropathy | 12 | 58003922 | A/G | 0.894 | 0.027 | 0.125 | 0.827 | 0.106 | cis |
| SMIM9 |  |  |  |  |  |  |  |  |  |  |
| rs1044032 | Diabetic polyneuropathy | 15 | 45968435 | T/C | 0.740 | 0.150 | 0.086 | 0.081 | 0.260 | trans |
| rs4584622 | Diabetic polyneuropathy | 12 | 72672823 | T/C | 0.672 | 0.021 | 0.081 | 0.790 | 0.328 | trans |
| IDI2 |  |  |  |  |  |  |  |  |  |  |
| rs1044261 | Diabetic polyneuropathy | 10 | 1065710 | T/C | 0.050 | -0.338 | 0.171 | 0.049 | 0.050 | cis |
| KLRC3 |  |  |  |  |  |  |  |  |  |  |
| rs704 | Diabetic polyneuropathy | 17 | 26694861 | A/G | 0.420 | 0.049 | 0.076 | 0.519 | 0.420 | trans |
| rs10445391 | Diabetic polyneuropathy | 17 | 34306106 | A/G | 0.933 | 0.030 | 0.152 | 0.843 | 0.067 | trans |
| rs12092641 | Diabetic polyneuropathy | 1 | 57426950 | T/C | 0.963 | -0.015 | 0.200 | 0.939 | 0.037 | trans |
| TNFRSF21 |  |  |  |  |  |  |  |  |  |  |
| rs13107325 | Diabetic polyneuropathy | 4 | 103188709 | T/C | 0.014 | 0.106 | 0.309 | 0.731 | 0.014 | trans |
| rs77924615 | Diabetic polyneuropathy | 16 | 20392332 | A/G | 0.222 | 0.042 | 0.091 | 0.643 | 0.222 | trans |
| rs2008174 | Diabetic polyneuropathy | 22 | 39860130 | C/T | 0.295 | 0.001 | 0.084 | 0.991 | 0.295 | trans |
| rs2298475 | Diabetic polyneuropathy | 11 | 126278203 | C/T | 0.107 | -0.040 | 0.123 | 0.743 | 0.107 | trans |
| rs583104 | Diabetic polyneuropathy | 1 | 109821307 | T/G | 0.781 | -0.096 | 0.091 | 0.294 | 0.219 | trans |
| rs1260326 | Diabetic polyneuropathy | 2 | 27730940 | T/C | 0.351 | -0.100 | 0.079 | 0.208 | 0.351 | trans |
| rs150816167 | Diabetic polyneuropathy | 1 | 179571862 | C/T | 0.064 | -0.189 | 0.158 | 0.233 | 0.064 | trans |
| rs75166367 | Diabetic polyneuropathy | 2 | 162964301 | A/G | 0.071 | -0.204 | 0.149 | 0.170 | 0.071 | trans |
| IL18BP |  |  |  |  |  |  |  |  |  |  |
| rs61747728 | Diabetic polyneuropathy | 1 | 179526214 | T/C | 0.064 | -0.201 | 0.158 | 0.204 | 0.064 | trans |
| AOC1 |  |  |  |  |  |  |  |  |  |  |
| rs10452848 | Diabetic polyneuropathy | 7 | 150523544 | A/G | 0.638 | -0.004 | 0.079 | 0.961 | 0.362 | cis |
| CD109 |  |  |  |  |  |  |  |  |  |  |
| rs8176749 | Diabetic polyneuropathy | 9 | 136131188 | T/C | 0.134 | 0.149 | 0.111 | 0.181 | 0.134 | trans |
| TPP1 |  |  |  |  |  |  |  |  |  |  |
| rs6848819 | Diabetic polyneuropathy | 4 | 154451678 | C/A | 0.014 | 0.372 | 0.307 | 0.226 | 0.014 | trans |
| rs10778152 | Diabetic polyneuropathy | 12 | 102225751 | G/A | 0.283 | 0.173 | 0.084 | 0.039 | 0.283 | trans |
| rs1654425 | Diabetic polyneuropathy | 19 | 55538980 | C/T | 0.879 | 0.126 | 0.115 | 0.272 | 0.121 | trans |
| rs10455861 | Diabetic polyneuropathy | 6 | 160401662 | A/G | 0.124 | -0.047 | 0.116 | 0.688 | 0.124 | trans |
| rs3184504 | Diabetic polyneuropathy | 12 | 111884608 | C/T | 0.591 | -0.051 | 0.077 | 0.504 | 0.409 | trans |
| AMH |  |  |  |  |  |  |  |  |  |  |
| rs35862450 | Diabetic polyneuropathy | 11 | 6462087 | A/G | 0.260 | -0.120 | 0.086 | 0.163 | 0.260 | trans |
| ECHS1 |  |  |  |  |  |  |  |  |  |  |
| rs10466126 | Diabetic polyneuropathy | 10 | 135186806 | A/G | 0.177 | -0.108 | 0.100 | 0.282 | 0.177 | cis |
| KL |  |  |  |  |  |  |  |  |  |  |
| rs10467359 | Diabetic polyneuropathy | 13 | 33529264 | G/A | 0.073 | 0.301 | 0.147 | 0.040 | 0.073 | cis |
| rs8176746 | Diabetic polyneuropathy | 9 | 136131322 | T/G | 0.133 | 0.161 | 0.111 | 0.146 | 0.133 | trans |
| rs1169311 | Diabetic polyneuropathy | 12 | 121440731 | T/C | 0.355 | 0.063 | 0.079 | 0.429 | 0.355 | trans |
| rs9961915 | Diabetic polyneuropathy | 18 | 24687324 | T/C | 0.247 | 0.041 | 0.087 | 0.639 | 0.247 | trans |
| rs7298766 | Diabetic polyneuropathy | 12 | 661656 | G/A | 0.263 | 0.026 | 0.085 | 0.760 | 0.263 | trans |
| rs4846806 | Diabetic polyneuropathy | 1 | 217207089 | T/C | 0.307 | -0.018 | 0.082 | 0.827 | 0.307 | trans |
| rs17855739 | Diabetic polyneuropathy | 19 | 5831840 | T/C | 0.030 | -0.022 | 0.221 | 0.922 | 0.030 | trans |
| rs11605349 | Diabetic polyneuropathy | 11 | 126305755 | C/T | 0.118 | -0.049 | 0.120 | 0.683 | 0.118 | trans |
| PAF |  |  |  |  |  |  |  |  |  |  |
| rs7412 | Diabetic polyneuropathy | 19 | 45412079 | T/C | 0.053 | 0.166 | 0.164 | 0.311 | 0.053 | trans |
| rs10468017 | Diabetic polyneuropathy | 15 | 58678512 | T/C | 0.332 | -0.029 | 0.080 | 0.717 | 0.332 | trans |
| rs1943977 | Diabetic polyneuropathy | 18 | 47173181 | A/G | 0.170 | -0.102 | 0.100 | 0.309 | 0.170 | trans |
| rs174574 | Diabetic polyneuropathy | 11 | 61600342 | A/C | 0.414 | -0.122 | 0.077 | 0.112 | 0.414 | trans |
| SIT1 |  |  |  |  |  |  |  |  |  |  |
| rs12446515 | Diabetic polyneuropathy | 16 | 56987015 | T/C | 0.280 | 0.041 | 0.085 | 0.631 | 0.280 | trans |
| rs4613118 | Diabetic polyneuropathy | 17 | 7297452 | G/A | 0.840 | 0.032 | 0.103 | 0.758 | 0.160 | trans |
| rs10468017 | Diabetic polyneuropathy | 15 | 58678512 | T/C | 0.332 | -0.029 | 0.080 | 0.717 | 0.332 | trans |
| rs3184504 | Diabetic polyneuropathy | 12 | 111884608 | C/T | 0.591 | -0.051 | 0.077 | 0.504 | 0.409 | trans |
| rs10469472 | Diabetic polyneuropathy | 19 | 17486926 | G/T | 0.180 | -0.114 | 0.100 | 0.251 | 0.180 | trans |
| CTSL |  |  |  |  |  |  |  |  |  |  |
| rs9497576 | Diabetic polyneuropathy | 6 | 146925676 | T/G | 0.065 | 0.267 | 0.155 | 0.084 | 0.065 | trans |
| rs76904798 | Diabetic polyneuropathy | 12 | 40614434 | T/C | 0.091 | 0.150 | 0.129 | 0.245 | 0.091 | trans |
| rs10469365 | Diabetic polyneuropathy | 19 | 12731874 | G/A | 0.081 | 0.115 | 0.138 | 0.407 | 0.081 | trans |
| rs17151689 | Diabetic polyneuropathy | 7 | 76950686 | A/C | 0.021 | 0.115 | 0.263 | 0.663 | 0.021 | trans |
| rs4808758 | Diabetic polyneuropathy | 19 | 18291289 | G/A | 0.231 | 0.012 | 0.089 | 0.893 | 0.231 | trans |
| rs72695000 | Diabetic polyneuropathy | 14 | 95104745 | T/C | 0.132 | -0.008 | 0.111 | 0.944 | 0.132 | trans |
| rs2282240 | Diabetic polyneuropathy | 9 | 27572634 | T/C | 0.271 | -0.052 | 0.085 | 0.539 | 0.271 | trans |
| rs150370599 | Diabetic polyneuropathy | 11 | 60718792 | T/C | 0.073 | -0.082 | 0.147 | 0.578 | 0.073 | trans |
| rs5030044 | Diabetic polyneuropathy | 3 | 186449122 | G/A | 0.134 | -0.100 | 0.111 | 0.368 | 0.134 | trans |
| rs9901675 | Diabetic polyneuropathy | 17 | 7484812 | A/G | 0.030 | -0.127 | 0.218 | 0.560 | 0.030 | cis |
| rs9604045 | Diabetic polyneuropathy | 13 | 113927208 | T/G | 0.171 | -0.150 | 0.103 | 0.143 | 0.171 | trans |
| GUCA2B |  |  |  |  |  |  |  |  |  |  |
| rs1047047 | Diabetic polyneuropathy | 1 | 42619139 | A/G | 0.832 | 0.005 | 0.101 | 0.964 | 0.168 | cis |
| ENPP5 |  |  |  |  |  |  |  |  |  |  |
| rs1047153 | Diabetic polyneuropathy | 6 | 46128745 | T/C | 0.717 | -0.064 | 0.084 | 0.444 | 0.283 | cis |
| GZMK |  |  |  |  |  |  |  |  |  |  |
| rs6693121 | Diabetic polyneuropathy | 1 | 101744633 | A/C | 0.346 | -0.073 | 0.079 | 0.359 | 0.346 | trans |
| NCF4 |  |  |  |  |  |  |  |  |  |  |
| rs1047286 | Diabetic polyneuropathy | 19 | 6713262 | A/G | 0.174 | -0.094 | 0.100 | 0.346 | 0.174 | trans |
| B3GNT4 |  |  |  |  |  |  |  |  |  |  |
| rs704 | Diabetic polyneuropathy | 17 | 26694861 | A/G | 0.420 | 0.049 | 0.076 | 0.519 | 0.420 | trans |
| rs1706435 | Diabetic polyneuropathy | 3 | 165461476 | G/A | 0.310 | -0.079 | 0.082 | 0.331 | 0.310 | trans |
| rs1047286 | Diabetic polyneuropathy | 19 | 6713262 | A/G | 0.174 | -0.094 | 0.100 | 0.346 | 0.174 | trans |
| DUS2 |  |  |  |  |  |  |  |  |  |  |
| rs1047286 | Diabetic polyneuropathy | 19 | 6713262 | A/G | 0.174 | -0.094 | 0.100 | 0.346 | 0.174 | trans |
| MAGEB10 |  |  |  |  |  |  |  |  |  |  |
| rs2686395 | Diabetic polyneuropathy | 3 | 165485019 | T/C | 0.246 | -0.049 | 0.087 | 0.576 | 0.246 | trans |
| rs1047286 | Diabetic polyneuropathy | 19 | 6713262 | A/G | 0.174 | -0.094 | 0.100 | 0.346 | 0.174 | trans |
| HLA-DRB3 |  |  |  |  |  |  |  |  |  |  |
| rs1047286 | Diabetic polyneuropathy | 19 | 6713262 | A/G | 0.174 | -0.094 | 0.100 | 0.346 | 0.174 | trans |
| rs626457 | Diabetic polyneuropathy | 1 | 57407484 | T/C | 0.402 | -0.114 | 0.077 | 0.140 | 0.402 | trans |
| FOXRED1 |  |  |  |  |  |  |  |  |  |  |
| rs1047286 | Diabetic polyneuropathy | 19 | 6713262 | A/G | 0.174 | -0.094 | 0.100 | 0.346 | 0.174 | trans |
| SCN4B |  |  |  |  |  |  |  |  |  |  |
| rs11720167 | Diabetic polyneuropathy | 3 | 165486145 | G/T | 0.174 | -0.015 | 0.099 | 0.877 | 0.174 | trans |
| rs1047286 | Diabetic polyneuropathy | 19 | 6713262 | A/G | 0.174 | -0.094 | 0.100 | 0.346 | 0.174 | trans |
| MST1R |  |  |  |  |  |  |  |  |  |  |
| rs1707652 | Diabetic polyneuropathy | 3 | 165478799 | T/C | 0.248 | -0.037 | 0.087 | 0.672 | 0.248 | trans |
| rs1047286 | Diabetic polyneuropathy | 19 | 6713262 | A/G | 0.174 | -0.094 | 0.100 | 0.346 | 0.174 | trans |
| MAGEA5 |  |  |  |  |  |  |  |  |  |  |
| rs1707652 | Diabetic polyneuropathy | 3 | 165478799 | T/C | 0.248 | -0.037 | 0.087 | 0.672 | 0.248 | trans |
| rs1047286 | Diabetic polyneuropathy | 19 | 6713262 | A/G | 0.174 | -0.094 | 0.100 | 0.346 | 0.174 | trans |
| LRFN5 |  |  |  |  |  |  |  |  |  |  |
| rs1707652 | Diabetic polyneuropathy | 3 | 165478799 | T/C | 0.248 | -0.037 | 0.087 | 0.672 | 0.248 | trans |
| rs1047286 | Diabetic polyneuropathy | 19 | 6713262 | A/G | 0.174 | -0.094 | 0.100 | 0.346 | 0.174 | trans |
| CSN2 |  |  |  |  |  |  |  |  |  |  |
| rs1047286 | Diabetic polyneuropathy | 19 | 6713262 | A/G | 0.174 | -0.094 | 0.100 | 0.346 | 0.174 | trans |
| DES |  |  |  |  |  |  |  |  |  |  |
| rs1047286 | Diabetic polyneuropathy | 19 | 6713262 | A/G | 0.174 | -0.094 | 0.100 | 0.346 | 0.174 | trans |
| LSM4 |  |  |  |  |  |  |  |  |  |  |
| rs704 | Diabetic polyneuropathy | 17 | 26694861 | A/G | 0.420 | 0.049 | 0.076 | 0.519 | 0.420 | trans |
| rs1707652 | Diabetic polyneuropathy | 3 | 165478799 | T/C | 0.248 | -0.037 | 0.087 | 0.672 | 0.248 | trans |
| rs1047286 | Diabetic polyneuropathy | 19 | 6713262 | A/G | 0.174 | -0.094 | 0.100 | 0.346 | 0.174 | trans |
| BTN3A1 |  |  |  |  |  |  |  |  |  |  |
| rs2686395 | Diabetic polyneuropathy | 3 | 165485019 | T/C | 0.246 | -0.049 | 0.087 | 0.576 | 0.246 | trans |
| rs1047286 | Diabetic polyneuropathy | 19 | 6713262 | A/G | 0.174 | -0.094 | 0.100 | 0.346 | 0.174 | trans |
| MOCS3 |  |  |  |  |  |  |  |  |  |  |
| rs2686395 | Diabetic polyneuropathy | 3 | 165485019 | T/C | 0.246 | -0.049 | 0.087 | 0.576 | 0.246 | trans |
| rs1047286 | Diabetic polyneuropathy | 19 | 6713262 | A/G | 0.174 | -0.094 | 0.100 | 0.346 | 0.174 | trans |
| VSTM4 |  |  |  |  |  |  |  |  |  |  |
| rs1047286 | Diabetic polyneuropathy | 19 | 6713262 | A/G | 0.174 | -0.094 | 0.100 | 0.346 | 0.174 | trans |
| C1orf162 |  |  |  |  |  |  |  |  |  |  |
| rs8192297 | Diabetic polyneuropathy | 15 | 90344352 | C/T | 0.152 | 0.105 | 0.108 | 0.331 | 0.152 | trans |
| rs704 | Diabetic polyneuropathy | 17 | 26694861 | A/G | 0.420 | 0.049 | 0.076 | 0.519 | 0.420 | trans |
| rs1047286 | Diabetic polyneuropathy | 19 | 6713262 | A/G | 0.174 | -0.094 | 0.100 | 0.346 | 0.174 | trans |
| rs1260326 | Diabetic polyneuropathy | 2 | 27730940 | T/C | 0.351 | -0.100 | 0.079 | 0.208 | 0.351 | trans |
| rs72840032 | Diabetic polyneuropathy | 10 | 101889964 | T/C | 0.038 | -0.213 | 0.196 | 0.278 | 0.038 | trans |
| RBM24 |  |  |  |  |  |  |  |  |  |  |
| rs2686395 | Diabetic polyneuropathy | 3 | 165485019 | T/C | 0.246 | -0.049 | 0.087 | 0.576 | 0.246 | trans |
| rs1047286 | Diabetic polyneuropathy | 19 | 6713262 | A/G | 0.174 | -0.094 | 0.100 | 0.346 | 0.174 | trans |
| TXNIP |  |  |  |  |  |  |  |  |  |  |
| rs1047286 | Diabetic polyneuropathy | 19 | 6713262 | A/G | 0.174 | -0.094 | 0.100 | 0.346 | 0.174 | trans |
| TPM2 |  |  |  |  |  |  |  |  |  |  |
| rs1707652 | Diabetic polyneuropathy | 3 | 165478799 | T/C | 0.248 | -0.037 | 0.087 | 0.672 | 0.248 | trans |
| rs1047286 | Diabetic polyneuropathy | 19 | 6713262 | A/G | 0.174 | -0.094 | 0.100 | 0.346 | 0.174 | trans |
| TMX2 |  |  |  |  |  |  |  |  |  |  |
| rs2686395 | Diabetic polyneuropathy | 3 | 165485019 | T/C | 0.246 | -0.049 | 0.087 | 0.576 | 0.246 | trans |
| rs1047286 | Diabetic polyneuropathy | 19 | 6713262 | A/G | 0.174 | -0.094 | 0.100 | 0.346 | 0.174 | trans |
| STAB2 |  |  |  |  |  |  |  |  |  |  |
| rs2686395 | Diabetic polyneuropathy | 3 | 165485019 | T/C | 0.246 | -0.049 | 0.087 | 0.576 | 0.246 | trans |
| rs1047286 | Diabetic polyneuropathy | 19 | 6713262 | A/G | 0.174 | -0.094 | 0.100 | 0.346 | 0.174 | trans |
| CD164L2 |  |  |  |  |  |  |  |  |  |  |
| rs1707652 | Diabetic polyneuropathy | 3 | 165478799 | T/C | 0.248 | -0.037 | 0.087 | 0.672 | 0.248 | trans |
| rs1047286 | Diabetic polyneuropathy | 19 | 6713262 | A/G | 0.174 | -0.094 | 0.100 | 0.346 | 0.174 | trans |
| RNF149 |  |  |  |  |  |  |  |  |  |  |
| rs1707652 | Diabetic polyneuropathy | 3 | 165478799 | T/C | 0.248 | -0.037 | 0.087 | 0.672 | 0.248 | trans |
| rs1047286 | Diabetic polyneuropathy | 19 | 6713262 | A/G | 0.174 | -0.094 | 0.100 | 0.346 | 0.174 | trans |
| TNNT3 |  |  |  |  |  |  |  |  |  |  |
| rs1707652 | Diabetic polyneuropathy | 3 | 165478799 | T/C | 0.248 | -0.037 | 0.087 | 0.672 | 0.248 | trans |
| rs1047286 | Diabetic polyneuropathy | 19 | 6713262 | A/G | 0.174 | -0.094 | 0.100 | 0.346 | 0.174 | trans |
| AKR1B10 |  |  |  |  |  |  |  |  |  |  |
| rs704 | Diabetic polyneuropathy | 17 | 26694861 | A/G | 0.420 | 0.049 | 0.076 | 0.519 | 0.420 | trans |
| rs1047286 | Diabetic polyneuropathy | 19 | 6713262 | A/G | 0.174 | -0.094 | 0.100 | 0.346 | 0.174 | trans |
| RAPGEF5 |  |  |  |  |  |  |  |  |  |  |
| rs1047286 | Diabetic polyneuropathy | 19 | 6713262 | A/G | 0.174 | -0.094 | 0.100 | 0.346 | 0.174 | trans |
| RAB3A |  |  |  |  |  |  |  |  |  |  |
| rs1707652 | Diabetic polyneuropathy | 3 | 165478799 | T/C | 0.248 | -0.037 | 0.087 | 0.672 | 0.248 | trans |
| rs1047286 | Diabetic polyneuropathy | 19 | 6713262 | A/G | 0.174 | -0.094 | 0.100 | 0.346 | 0.174 | trans |
| VLDLR |  |  |  |  |  |  |  |  |  |  |
| rs1707652 | Diabetic polyneuropathy | 3 | 165478799 | T/C | 0.248 | -0.037 | 0.087 | 0.672 | 0.248 | trans |
| rs1047286 | Diabetic polyneuropathy | 19 | 6713262 | A/G | 0.174 | -0.094 | 0.100 | 0.346 | 0.174 | trans |
| PTGES3 |  |  |  |  |  |  |  |  |  |  |
| rs704 | Diabetic polyneuropathy | 17 | 26694861 | A/G | 0.420 | 0.049 | 0.076 | 0.519 | 0.420 | trans |
| rs1707652 | Diabetic polyneuropathy | 3 | 165478799 | T/C | 0.248 | -0.037 | 0.087 | 0.672 | 0.248 | trans |
| rs1047286 | Diabetic polyneuropathy | 19 | 6713262 | A/G | 0.174 | -0.094 | 0.100 | 0.346 | 0.174 | trans |
| SNX7 |  |  |  |  |  |  |  |  |  |  |
| rs1707652 | Diabetic polyneuropathy | 3 | 165478799 | T/C | 0.248 | -0.037 | 0.087 | 0.672 | 0.248 | trans |
| rs1047286 | Diabetic polyneuropathy | 19 | 6713262 | A/G | 0.174 | -0.094 | 0.100 | 0.346 | 0.174 | trans |
| SCG5 |  |  |  |  |  |  |  |  |  |  |
| rs1707652 | Diabetic polyneuropathy | 3 | 165478799 | T/C | 0.248 | -0.037 | 0.087 | 0.672 | 0.248 | trans |
| rs1047286 | Diabetic polyneuropathy | 19 | 6713262 | A/G | 0.174 | -0.094 | 0.100 | 0.346 | 0.174 | trans |
| TMEM185A |  |  |  |  |  |  |  |  |  |  |
| rs704 | Diabetic polyneuropathy | 17 | 26694861 | A/G | 0.420 | 0.049 | 0.076 | 0.519 | 0.420 | trans |
| rs1707652 | Diabetic polyneuropathy | 3 | 165478799 | T/C | 0.248 | -0.037 | 0.087 | 0.672 | 0.248 | trans |
| rs1047286 | Diabetic polyneuropathy | 19 | 6713262 | A/G | 0.174 | -0.094 | 0.100 | 0.346 | 0.174 | trans |
| OSM |  |  |  |  |  |  |  |  |  |  |
| rs1047286 | Diabetic polyneuropathy | 19 | 6713262 | A/G | 0.174 | -0.094 | 0.100 | 0.346 | 0.174 | trans |
| rs2511241 | Diabetic polyneuropathy | 11 | 72945341 | T/C | 0.922 | -0.311 | 0.141 | 0.028 | 0.078 | trans |
| PKDCC |  |  |  |  |  |  |  |  |  |  |
| rs1047286 | Diabetic polyneuropathy | 19 | 6713262 | A/G | 0.174 | -0.094 | 0.100 | 0.346 | 0.174 | trans |
| PET117 |  |  |  |  |  |  |  |  |  |  |
| rs1707652 | Diabetic polyneuropathy | 3 | 165478799 | T/C | 0.248 | -0.037 | 0.087 | 0.672 | 0.248 | trans |
| rs1047286 | Diabetic polyneuropathy | 19 | 6713262 | A/G | 0.174 | -0.094 | 0.100 | 0.346 | 0.174 | trans |
| OTUD5 |  |  |  |  |  |  |  |  |  |  |
| rs1707652 | Diabetic polyneuropathy | 3 | 165478799 | T/C | 0.248 | -0.037 | 0.087 | 0.672 | 0.248 | trans |
| rs1047286 | Diabetic polyneuropathy | 19 | 6713262 | A/G | 0.174 | -0.094 | 0.100 | 0.346 | 0.174 | trans |
| POLR1C |  |  |  |  |  |  |  |  |  |  |
| rs1707652 | Diabetic polyneuropathy | 3 | 165478799 | T/C | 0.248 | -0.037 | 0.087 | 0.672 | 0.248 | trans |
| rs1047286 | Diabetic polyneuropathy | 19 | 6713262 | A/G | 0.174 | -0.094 | 0.100 | 0.346 | 0.174 | trans |
| ATP1B3 |  |  |  |  |  |  |  |  |  |  |
| rs2686395 | Diabetic polyneuropathy | 3 | 165485019 | T/C | 0.246 | -0.049 | 0.087 | 0.576 | 0.246 | trans |
| rs1047286 | Diabetic polyneuropathy | 19 | 6713262 | A/G | 0.174 | -0.094 | 0.100 | 0.346 | 0.174 | trans |
| SHISA2 |  |  |  |  |  |  |  |  |  |  |
| rs1047286 | Diabetic polyneuropathy | 19 | 6713262 | A/G | 0.174 | -0.094 | 0.100 | 0.346 | 0.174 | trans |
| RGS8 |  |  |  |  |  |  |  |  |  |  |
| rs1707652 | Diabetic polyneuropathy | 3 | 165478799 | T/C | 0.248 | -0.037 | 0.087 | 0.672 | 0.248 | trans |
| rs1047286 | Diabetic polyneuropathy | 19 | 6713262 | A/G | 0.174 | -0.094 | 0.100 | 0.346 | 0.174 | trans |
| ST6GALNAC2 |  |  |  |  |  |  |  |  |  |  |
| rs1707652 | Diabetic polyneuropathy | 3 | 165478799 | T/C | 0.248 | -0.037 | 0.087 | 0.672 | 0.248 | trans |
| rs1047286 | Diabetic polyneuropathy | 19 | 6713262 | A/G | 0.174 | -0.094 | 0.100 | 0.346 | 0.174 | trans |
| PPP3R2 |  |  |  |  |  |  |  |  |  |  |
| rs704 | Diabetic polyneuropathy | 17 | 26694861 | A/G | 0.420 | 0.049 | 0.076 | 0.519 | 0.420 | trans |
| rs1707652 | Diabetic polyneuropathy | 3 | 165478799 | T/C | 0.248 | -0.037 | 0.087 | 0.672 | 0.248 | trans |
| rs1047286 | Diabetic polyneuropathy | 19 | 6713262 | A/G | 0.174 | -0.094 | 0.100 | 0.346 | 0.174 | trans |
| ZAP70 |  |  |  |  |  |  |  |  |  |  |
| rs1707652 | Diabetic polyneuropathy | 3 | 165478799 | T/C | 0.248 | -0.037 | 0.087 | 0.672 | 0.248 | trans |
| rs1047286 | Diabetic polyneuropathy | 19 | 6713262 | A/G | 0.174 | -0.094 | 0.100 | 0.346 | 0.174 | trans |
| PAK3 |  |  |  |  |  |  |  |  |  |  |
| rs1047286 | Diabetic polyneuropathy | 19 | 6713262 | A/G | 0.174 | -0.094 | 0.100 | 0.346 | 0.174 | trans |
| ORAOV1 |  |  |  |  |  |  |  |  |  |  |
| rs1047286 | Diabetic polyneuropathy | 19 | 6713262 | A/G | 0.174 | -0.094 | 0.100 | 0.346 | 0.174 | trans |
| STK16 |  |  |  |  |  |  |  |  |  |  |
| rs704 | Diabetic polyneuropathy | 17 | 26694861 | A/G | 0.420 | 0.049 | 0.076 | 0.519 | 0.420 | trans |
| rs1707652 | Diabetic polyneuropathy | 3 | 165478799 | T/C | 0.248 | -0.037 | 0.087 | 0.672 | 0.248 | trans |
| rs1047286 | Diabetic polyneuropathy | 19 | 6713262 | A/G | 0.174 | -0.094 | 0.100 | 0.346 | 0.174 | trans |
| RABEPK |  |  |  |  |  |  |  |  |  |  |
| rs704 | Diabetic polyneuropathy | 17 | 26694861 | A/G | 0.420 | 0.049 | 0.076 | 0.519 | 0.420 | trans |
| rs1707652 | Diabetic polyneuropathy | 3 | 165478799 | T/C | 0.248 | -0.037 | 0.087 | 0.672 | 0.248 | trans |
| rs1047286 | Diabetic polyneuropathy | 19 | 6713262 | A/G | 0.174 | -0.094 | 0.100 | 0.346 | 0.174 | trans |
| PPIL2 |  |  |  |  |  |  |  |  |  |  |
| rs1047286 | Diabetic polyneuropathy | 19 | 6713262 | A/G | 0.174 | -0.094 | 0.100 | 0.346 | 0.174 | trans |
| LYPD3 |  |  |  |  |  |  |  |  |  |  |
| rs2479016 | Diabetic polyneuropathy | 6 | 2245345 | G/A | 0.378 | 0.154 | 0.078 | 0.049 | 0.378 | trans |
| rs13107325 | Diabetic polyneuropathy | 4 | 103188709 | T/C | 0.014 | 0.106 | 0.309 | 0.731 | 0.014 | trans |
| rs62034713 | Diabetic polyneuropathy | 16 | 3359124 | G/A | 0.305 | 0.073 | 0.082 | 0.373 | 0.305 | trans |
| rs10893502 | Diabetic polyneuropathy | 11 | 126254018 | C/T | 0.619 | 0.055 | 0.078 | 0.479 | 0.382 | trans |
| rs601338 | Diabetic polyneuropathy | 19 | 49206674 | G/A | 0.626 | 0.044 | 0.078 | 0.571 | 0.375 | trans |
| rs2519093 | Diabetic polyneuropathy | 9 | 136141870 | T/C | 0.201 | 0.028 | 0.094 | 0.762 | 0.201 | trans |
| rs56278466 | Diabetic polyneuropathy | 10 | 17875857 | G/T | 0.581 | -0.003 | 0.077 | 0.970 | 0.419 | trans |
| rs1047891 | Diabetic polyneuropathy | 2 | 211540507 | A/C | 0.324 | -0.048 | 0.081 | 0.560 | 0.324 | trans |
| rs7842080 | Diabetic polyneuropathy | 8 | 134599329 | G/A | 0.177 | -0.053 | 0.098 | 0.588 | 0.177 | trans |
| rs383510 | Diabetic polyneuropathy | 21 | 42858367 | C/T | 0.619 | -0.056 | 0.077 | 0.468 | 0.381 | trans |
| rs1260326 | Diabetic polyneuropathy | 2 | 27730940 | T/C | 0.351 | -0.100 | 0.079 | 0.208 | 0.351 | trans |
| rs708686 | Diabetic polyneuropathy | 19 | 5840619 | T/C | 0.334 | -0.105 | 0.081 | 0.194 | 0.334 | trans |
| rs7257767 | Diabetic polyneuropathy | 19 | 44001345 | T/C | 0.074 | -0.225 | 0.143 | 0.116 | 0.074 | cis |
| ADGRG1 |  |  |  |  |  |  |  |  |  |  |
| rs2519093 | Diabetic polyneuropathy | 9 | 136141870 | T/C | 0.201 | 0.028 | 0.094 | 0.762 | 0.201 | trans |
| rs56278466 | Diabetic polyneuropathy | 10 | 17875857 | G/T | 0.581 | -0.003 | 0.077 | 0.970 | 0.419 | trans |
| rs1047891 | Diabetic polyneuropathy | 2 | 211540507 | A/C | 0.324 | -0.048 | 0.081 | 0.560 | 0.324 | trans |
| rs3184504 | Diabetic polyneuropathy | 12 | 111884608 | C/T | 0.591 | -0.051 | 0.077 | 0.504 | 0.409 | trans |
| PODXL2 |  |  |  |  |  |  |  |  |  |  |
| rs9922607 | Diabetic polyneuropathy | 16 | 17570220 | T/C | 0.079 | 0.224 | 0.139 | 0.105 | 0.079 | trans |
| rs45512696 | Diabetic polyneuropathy | 19 | 35550878 | T/C | 0.152 | -0.022 | 0.106 | 0.833 | 0.152 | trans |
| rs781656 | Diabetic polyneuropathy | 4 | 57778645 | A/G | 0.234 | -0.046 | 0.089 | 0.610 | 0.234 | trans |
| rs1047891 | Diabetic polyneuropathy | 2 | 211540507 | A/C | 0.324 | -0.048 | 0.081 | 0.560 | 0.324 | trans |
| rs61747728 | Diabetic polyneuropathy | 1 | 179526214 | T/C | 0.064 | -0.201 | 0.158 | 0.204 | 0.064 | trans |
| rs74841302 | Diabetic polyneuropathy | 15 | 42084329 | A/G | 0.020 | -0.362 | 0.270 | 0.181 | 0.020 | trans |
| RTN4R |  |  |  |  |  |  |  |  |  |  |
| rs1461729 | Diabetic polyneuropathy | 8 | 9187242 | G/A | 0.844 | 0.152 | 0.113 | 0.178 | 0.156 | trans |
| rs7644541 | Diabetic polyneuropathy | 3 | 136145766 | G/A | 0.859 | 0.030 | 0.107 | 0.779 | 0.141 | trans |
| rs28929474 | Diabetic polyneuropathy | 14 | 94844947 | T/C | 0.020 | -0.002 | 0.272 | 0.993 | 0.020 | trans |
| rs1047891 | Diabetic polyneuropathy | 2 | 211540507 | A/C | 0.324 | -0.048 | 0.081 | 0.560 | 0.324 | trans |
| rs56332871 | Diabetic polyneuropathy | 15 | 96714816 | A/C | 0.291 | -0.084 | 0.084 | 0.313 | 0.291 | trans |
| rs1260326 | Diabetic polyneuropathy | 2 | 27730940 | T/C | 0.351 | -0.100 | 0.079 | 0.208 | 0.351 | trans |
| rs663353 | Diabetic polyneuropathy | 22 | 20174712 | A/G | 0.766 | -0.160 | 0.089 | 0.073 | 0.234 | cis |
| GUSB |  |  |  |  |  |  |  |  |  |  |
| rs116587722 | Diabetic polyneuropathy | 4 | 119790517 | C/T | 0.062 | 0.295 | 0.157 | 0.060 | 0.062 | trans |
| rs1800450 | Diabetic polyneuropathy | 10 | 54531235 | T/C | 0.132 | 0.073 | 0.111 | 0.512 | 0.132 | trans |
| rs1788606 | Diabetic polyneuropathy | 18 | 71964632 | T/C | 0.406 | -0.016 | 0.077 | 0.834 | 0.406 | trans |
| rs2900660 | Diabetic polyneuropathy | 19 | 13022859 | C/A | 0.321 | -0.036 | 0.081 | 0.660 | 0.321 | trans |
| rs1047891 | Diabetic polyneuropathy | 2 | 211540507 | A/C | 0.324 | -0.048 | 0.081 | 0.560 | 0.324 | trans |
| rs4574742 | Diabetic polyneuropathy | 7 | 25995063 | G/A | 0.328 | -0.067 | 0.081 | 0.403 | 0.328 | trans |
| rs7970695 | Diabetic polyneuropathy | 12 | 121423376 | A/G | 0.584 | -0.089 | 0.077 | 0.246 | 0.416 | trans |
| rs174574 | Diabetic polyneuropathy | 11 | 61600342 | A/C | 0.414 | -0.122 | 0.077 | 0.112 | 0.414 | trans |
| rs62440901 | Diabetic polyneuropathy | 6 | 160569068 | T/C | 0.125 | -0.154 | 0.114 | 0.175 | 0.125 | trans |
| rs147233090 | Diabetic polyneuropathy | 15 | 44028047 | T/C | 0.005 | -0.444 | 0.516 | 0.389 | 0.005 | trans |
| ACE2 |  |  |  |  |  |  |  |  |  |  |
| rs340005 | Diabetic polyneuropathy | 15 | 60878030 | A/G | 0.721 | 0.126 | 0.084 | 0.134 | 0.279 | trans |
| rs72787359 | Diabetic polyneuropathy | 16 | 51431230 | C/A | 0.141 | 0.022 | 0.110 | 0.843 | 0.141 | trans |
| rs28929474 | Diabetic polyneuropathy | 14 | 94844947 | T/C | 0.020 | -0.002 | 0.272 | 0.993 | 0.020 | trans |
| rs1047891 | Diabetic polyneuropathy | 2 | 211540507 | A/C | 0.324 | -0.048 | 0.081 | 0.560 | 0.324 | trans |
| rs72694393 | Diabetic polyneuropathy | 14 | 24874193 | T/G | 0.380 | -0.048 | 0.078 | 0.539 | 0.380 | trans |
| rs112875651 | Diabetic polyneuropathy | 8 | 126506694 | A/G | 0.387 | -0.058 | 0.078 | 0.456 | 0.387 | trans |
| rs11065385 | Diabetic polyneuropathy | 12 | 121423386 | G/A | 0.647 | -0.102 | 0.080 | 0.201 | 0.353 | trans |
| CRTAM |  |  |  |  |  |  |  |  |  |  |
| rs35829610 | Diabetic polyneuropathy | 5 | 131823862 | CTG/C | 0.330 | 0.095 | 0.081 | 0.239 | 0.330 | trans |
| rs13401811 | Diabetic polyneuropathy | 2 | 111616104 | A/G | 0.214 | 0.080 | 0.092 | 0.388 | 0.214 | trans |
| rs2370794 | Diabetic polyneuropathy | 11 | 122714782 | G/A | 0.407 | 0.017 | 0.077 | 0.829 | 0.407 | cis |
| rs11215416 | Diabetic polyneuropathy | 11 | 115077327 | G/A | 0.266 | -0.045 | 0.085 | 0.599 | 0.266 | trans |
| rs1047891 | Diabetic polyneuropathy | 2 | 211540507 | A/C | 0.324 | -0.048 | 0.081 | 0.560 | 0.324 | trans |
| rs3184504 | Diabetic polyneuropathy | 12 | 111884608 | C/T | 0.591 | -0.051 | 0.077 | 0.504 | 0.409 | trans |
| VAT1 |  |  |  |  |  |  |  |  |  |  |
| rs7210098 | Diabetic polyneuropathy | 17 | 41293613 | T/C | 0.385 | 0.116 | 0.078 | 0.136 | 0.385 | cis |
| rs4149307 | Diabetic polyneuropathy | 9 | 107589744 | T/C | 0.111 | 0.069 | 0.121 | 0.568 | 0.111 | trans |
| rs11781667 | Diabetic polyneuropathy | 8 | 145040856 | T/G | 0.357 | 0.060 | 0.079 | 0.447 | 0.357 | trans |
| rs173539 | Diabetic polyneuropathy | 16 | 56988044 | T/C | 0.284 | 0.040 | 0.084 | 0.635 | 0.284 | trans |
| rs2868346 | Diabetic polyneuropathy | 20 | 44547970 | T/C | 0.749 | -0.005 | 0.087 | 0.955 | 0.251 | trans |
| rs1047891 | Diabetic polyneuropathy | 2 | 211540507 | A/C | 0.324 | -0.048 | 0.081 | 0.560 | 0.324 | trans |
| rs12342201 | Diabetic polyneuropathy | 9 | 95894964 | A/G | 0.418 | -0.103 | 0.076 | 0.177 | 0.418 | trans |
| rs174564 | Diabetic polyneuropathy | 11 | 61588305 | G/A | 0.415 | -0.118 | 0.077 | 0.124 | 0.415 | trans |
| rs190543502 | Diabetic polyneuropathy | 15 | 43757184 | C/T | 0.005 | -0.753 | 0.549 | 0.170 | 0.005 | trans |
| CA3 |  |  |  |  |  |  |  |  |  |  |
| rs11085824 | Diabetic polyneuropathy | 19 | 13001547 | G/A | 0.307 | 0.021 | 0.082 | 0.800 | 0.307 | trans |
| rs3811444 | Diabetic polyneuropathy | 1 | 248039451 | T/C | 0.346 | -0.037 | 0.080 | 0.645 | 0.346 | trans |
| rs1047891 | Diabetic polyneuropathy | 2 | 211540507 | A/C | 0.324 | -0.048 | 0.081 | 0.560 | 0.324 | trans |
| ASRGL1 |  |  |  |  |  |  |  |  |  |  |
| rs1354034 | Diabetic polyneuropathy | 3 | 56849749 | C/T | 0.707 | 0.052 | 0.083 | 0.532 | 0.293 | trans |
| rs1047891 | Diabetic polyneuropathy | 2 | 211540507 | A/C | 0.324 | -0.048 | 0.081 | 0.560 | 0.324 | trans |
| rs2893923 | Diabetic polyneuropathy | 10 | 65261184 | T/C | 0.299 | -0.072 | 0.083 | 0.383 | 0.299 | trans |
| FGF21 |  |  |  |  |  |  |  |  |  |  |
| rs35332062 | Diabetic polyneuropathy | 7 | 73012042 | A/G | 0.129 | 0.003 | 0.112 | 0.980 | 0.129 | trans |
| rs1047891 | Diabetic polyneuropathy | 2 | 211540507 | A/C | 0.324 | -0.048 | 0.081 | 0.560 | 0.324 | trans |
| rs112875651 | Diabetic polyneuropathy | 8 | 126506694 | A/G | 0.387 | -0.058 | 0.078 | 0.456 | 0.387 | trans |
| rs1260326 | Diabetic polyneuropathy | 2 | 27730940 | T/C | 0.351 | -0.100 | 0.079 | 0.208 | 0.351 | trans |
| PIP |  |  |  |  |  |  |  |  |  |  |
| rs681343 | Diabetic polyneuropathy | 19 | 49206462 | T/C | 0.375 | -0.045 | 0.078 | 0.568 | 0.375 | trans |
| LEG1 |  |  |  |  |  |  |  |  |  |  |
| rs681343 | Diabetic polyneuropathy | 19 | 49206462 | T/C | 0.375 | -0.045 | 0.078 | 0.568 | 0.375 | trans |
| KLK11 |  |  |  |  |  |  |  |  |  |  |
| rs1048328 | Diabetic polyneuropathy | 19 | 51527364 | A/G | 0.100 | 0.133 | 0.128 | 0.299 | 0.100 | cis |
| WFDC12 |  |  |  |  |  |  |  |  |  |  |
| rs17350445 | Diabetic polyneuropathy | 3 | 187632339 | T/C | 0.067 | 0.273 | 0.151 | 0.070 | 0.067 | trans |
| rs6508781 | Diabetic polyneuropathy | 19 | 38785318 | C/A | 0.211 | 0.169 | 0.093 | 0.069 | 0.211 | trans |
| rs714781 | Diabetic polyneuropathy | 21 | 40178381 | T/C | 0.417 | 0.095 | 0.077 | 0.216 | 0.417 | trans |
| rs12816349 | Diabetic polyneuropathy | 12 | 6709945 | A/G | 0.184 | 0.094 | 0.098 | 0.337 | 0.184 | trans |
| rs10483947 | Diabetic polyneuropathy | 14 | 80775115 | T/C | 0.135 | 0.038 | 0.112 | 0.733 | 0.135 | trans |
| rs8038032 | Diabetic polyneuropathy | 15 | 35302869 | G/A | 0.280 | -0.088 | 0.084 | 0.293 | 0.280 | trans |
| rs35673728 | Diabetic polyneuropathy | 15 | 41047777 | C/T | 0.037 | -0.162 | 0.203 | 0.425 | 0.037 | trans |
| RND1 |  |  |  |  |  |  |  |  |  |  |
| rs10484041 | Diabetic polyneuropathy | 14 | 94852691 | T/C | 0.110 | 0.026 | 0.121 | 0.833 | 0.110 | trans |
| CRELD2 |  |  |  |  |  |  |  |  |  |  |
| rs79091287 | Diabetic polyneuropathy | 12 | 133415738 | A/G | 0.054 | 0.195 | 0.166 | 0.241 | 0.054 | trans |
| rs9987289 | Diabetic polyneuropathy | 8 | 9183358 | G/A | 0.857 | 0.118 | 0.116 | 0.310 | 0.143 | trans |
| rs1805214 | Diabetic polyneuropathy | 12 | 104241405 | A/G | 0.195 | 0.014 | 0.096 | 0.887 | 0.195 | trans |
| rs3804749 | Diabetic polyneuropathy | 3 | 122833003 | T/C | 0.620 | 0.007 | 0.078 | 0.930 | 0.380 | trans |
| rs6796 | Diabetic polyneuropathy | 7 | 6502367 | C/T | 0.321 | -0.010 | 0.082 | 0.900 | 0.321 | trans |
| rs3752617 | Diabetic polyneuropathy | 7 | 127220917 | T/G | 0.062 | -0.064 | 0.154 | 0.676 | 0.062 | trans |
| rs10488686 | Diabetic polyneuropathy | 11 | 32129891 | A/G | 0.329 | -0.098 | 0.080 | 0.222 | 0.329 | trans |
| rs1801689 | Diabetic polyneuropathy | 17 | 64210580 | C/A | 0.010 | -0.158 | 0.383 | 0.681 | 0.010 | trans |
| MSTN |  |  |  |  |  |  |  |  |  |  |
| rs1815739 | Diabetic polyneuropathy | 11 | 66328095 | C/T | 0.679 | 0.159 | 0.081 | 0.050 | 0.321 | trans |
| rs10489588 | Diabetic polyneuropathy | 1 | 2951834 | A/G | 0.154 | -0.093 | 0.104 | 0.369 | 0.154 | trans |
| rs34950020 | Diabetic polyneuropathy | 6 | 133800545 | A/G | 0.251 | -0.191 | 0.087 | 0.029 | 0.251 | trans |
| ESM1 |  |  |  |  |  |  |  |  |  |  |
| rs4242051 | Diabetic polyneuropathy | 5 | 54198775 | C/T | 0.763 | -0.017 | 0.089 | 0.851 | 0.237 | cis |
| rs1260326 | Diabetic polyneuropathy | 2 | 27730940 | T/C | 0.351 | -0.100 | 0.079 | 0.208 | 0.351 | trans |
| rs10491244 | Diabetic polyneuropathy | 5 | 81716027 | A/G | 0.164 | -0.118 | 0.102 | 0.246 | 0.164 | trans |
| rs113760175 | Diabetic polyneuropathy | 6 | 22343592 | A/G | 0.082 | -0.158 | 0.140 | 0.258 | 0.082 | trans |
| rs16822633 | Diabetic polyneuropathy | 2 | 227646959 | G/A | 0.023 | -0.332 | 0.245 | 0.175 | 0.023 | trans |
| rs76895963 | Diabetic polyneuropathy | 12 | 4384844 | G/T | 0.031 | -0.346 | 0.225 | 0.125 | 0.031 | trans |
| NAALAD2 |  |  |  |  |  |  |  |  |  |  |
| rs855791 | Diabetic polyneuropathy | 22 | 37462936 | A/G | 0.347 | 0.039 | 0.079 | 0.619 | 0.347 | trans |
| rs1049296 | Diabetic polyneuropathy | 3 | 133494354 | T/C | 0.113 | 0.018 | 0.118 | 0.882 | 0.113 | trans |
| CRNN |  |  |  |  |  |  |  |  |  |  |
| rs10494275 | Diabetic polyneuropathy | 1 | 152391567 | A/G | 0.064 | 0.305 | 0.155 | 0.048 | 0.064 | cis |
| rs704 | Diabetic polyneuropathy | 17 | 26694861 | A/G | 0.420 | 0.049 | 0.076 | 0.519 | 0.420 | trans |
| DEFA5 |  |  |  |  |  |  |  |  |  |  |
| rs7839771 | Diabetic polyneuropathy | 8 | 6913026 | T/C | 0.013 | 0.602 | 0.344 | 0.080 | 0.013 | cis |
| rs12019079 | Diabetic polyneuropathy | 17 | 70066875 | T/C | 0.770 | 0.037 | 0.091 | 0.681 | 0.230 | trans |
| rs507666 | Diabetic polyneuropathy | 9 | 136149399 | A/G | 0.201 | 0.019 | 0.094 | 0.840 | 0.201 | trans |
| rs10495440 | Diabetic polyneuropathy | 1 | 239275579 | T/C | 0.184 | 0.005 | 0.097 | 0.956 | 0.184 | trans |
| rs35470271 | Diabetic polyneuropathy | 3 | 40915239 | A/G | 0.791 | -0.036 | 0.093 | 0.701 | 0.209 | trans |
| rs4647930 | Diabetic polyneuropathy | 4 | 1018705 | A/C | 0.238 | -0.098 | 0.089 | 0.269 | 0.238 | trans |
| FNDC5 |  |  |  |  |  |  |  |  |  |  |
| rs10500671 | Diabetic polyneuropathy | 11 | 6460026 | A/C | 0.260 | -0.121 | 0.086 | 0.162 | 0.260 | trans |
| BDNF |  |  |  |  |  |  |  |  |  |  |
| rs10501089 | Diabetic polyneuropathy | 11 | 27788859 | T/C | 0.095 | 0.256 | 0.130 | 0.049 | 0.095 | cis |
| rs1354034 | Diabetic polyneuropathy | 3 | 56849749 | C/T | 0.707 | 0.052 | 0.083 | 0.532 | 0.293 | trans |
| rs10822155 | Diabetic polyneuropathy | 10 | 65071215 | A/C | 0.385 | 0.000 | 0.078 | 0.997 | 0.385 | trans |
| rs61658003 | Diabetic polyneuropathy | 19 | 55544203 | C/A | 0.113 | -0.135 | 0.121 | 0.261 | 0.113 | trans |
| SCGB1A1 |  |  |  |  |  |  |  |  |  |  |
| rs7963771 | Diabetic polyneuropathy | 12 | 115343492 | T/C | 0.226 | 0.076 | 0.090 | 0.399 | 0.226 | trans |
| rs887373 | Diabetic polyneuropathy | 5 | 126453725 | C/T | 0.593 | 0.034 | 0.077 | 0.654 | 0.407 | trans |
| rs7962469 | Diabetic polyneuropathy | 12 | 52348259 | A/G | 0.318 | 0.022 | 0.081 | 0.789 | 0.318 | trans |
| rs1515498 | Diabetic polyneuropathy | 3 | 189508302 | G/A | 0.334 | 0.012 | 0.080 | 0.880 | 0.334 | trans |
| rs1766143 | Diabetic polyneuropathy | 14 | 36696342 | G/A | 0.256 | 0.003 | 0.086 | 0.970 | 0.256 | trans |
| rs3741240 | Diabetic polyneuropathy | 11 | 62186542 | A/G | 0.345 | -0.011 | 0.080 | 0.891 | 0.345 | cis |
| rs7040029 | Diabetic polyneuropathy | 9 | 117619214 | C/T | 0.742 | -0.035 | 0.086 | 0.688 | 0.258 | trans |
| rs1997814 | Diabetic polyneuropathy | 20 | 10646671 | G/T | 0.717 | -0.041 | 0.084 | 0.622 | 0.283 | trans |
| rs3856521 | Diabetic polyneuropathy | 2 | 61405723 | A/G | 0.110 | -0.045 | 0.121 | 0.707 | 0.110 | trans |
| rs7072547 | Diabetic polyneuropathy | 10 | 111982398 | A/G | 0.147 | -0.058 | 0.106 | 0.587 | 0.147 | trans |
| rs3765766 | Diabetic polyneuropathy | 1 | 3634660 | C/T | 0.848 | -0.094 | 0.106 | 0.379 | 0.152 | trans |
| SMPD1 |  |  |  |  |  |  |  |  |  |  |
| rs139974673 | Diabetic polyneuropathy | 15 | 44027885 | T/C | 0.995 | 0.444 | 0.516 | 0.389 | 0.005 | trans |
| rs10778152 | Diabetic polyneuropathy | 12 | 102225751 | G/A | 0.283 | 0.173 | 0.084 | 0.039 | 0.283 | trans |
| rs77945361 | Diabetic polyneuropathy | 4 | 154254534 | A/G | 0.184 | 0.072 | 0.098 | 0.462 | 0.184 | trans |
| rs78444298 | Diabetic polyneuropathy | 1 | 184672098 | A/G | 0.017 | 0.023 | 0.295 | 0.937 | 0.017 | trans |
| rs58542926 | Diabetic polyneuropathy | 19 | 19379549 | T/C | 0.064 | 0.005 | 0.153 | 0.977 | 0.064 | trans |
| rs11791806 | Diabetic polyneuropathy | 9 | 34633529 | T/C | 0.133 | -0.007 | 0.112 | 0.948 | 0.133 | trans |
| rs3747207 | Diabetic polyneuropathy | 22 | 44324855 | A/G | 0.226 | -0.010 | 0.090 | 0.915 | 0.226 | trans |
| rs112635299 | Diabetic polyneuropathy | 14 | 94838142 | T/G | 0.020 | -0.014 | 0.271 | 0.960 | 0.020 | trans |
| rs2862954 | Diabetic polyneuropathy | 10 | 101912064 | C/T | 0.390 | -0.059 | 0.077 | 0.448 | 0.390 | trans |
| rs1050239 | Diabetic polyneuropathy | 11 | 6415463 | A/G | 0.216 | -0.076 | 0.093 | 0.409 | 0.216 | cis |
| rs429358 | Diabetic polyneuropathy | 19 | 45411941 | C/T | 0.183 | -0.237 | 0.100 | 0.018 | 0.183 | trans |
| BLMH |  |  |  |  |  |  |  |  |  |  |
| rs1050565 | Diabetic polyneuropathy | 17 | 28576076 | C/T | 0.343 | 0.015 | 0.079 | 0.848 | 0.343 | cis |
| rs1250258 | Diabetic polyneuropathy | 2 | 216300185 | T/C | 0.789 | -0.023 | 0.092 | 0.800 | 0.212 | trans |
| MAP1LC3A |  |  |  |  |  |  |  |  |  |  |
| rs7475335 | Diabetic polyneuropathy | 10 | 104343428 | A/C | 0.300 | 0.084 | 0.082 | 0.309 | 0.300 | trans |
| rs11792848 | Diabetic polyneuropathy | 9 | 99089943 | A/G | 0.266 | 0.083 | 0.086 | 0.337 | 0.266 | trans |
| rs1354034 | Diabetic polyneuropathy | 3 | 56849749 | C/T | 0.707 | 0.052 | 0.083 | 0.532 | 0.293 | trans |
| rs7503168 | Diabetic polyneuropathy | 17 | 33885904 | A/G | 0.820 | -0.067 | 0.098 | 0.495 | 0.180 | trans |
| LIPN |  |  |  |  |  |  |  |  |  |  |
| rs10509554 | Diabetic polyneuropathy | 10 | 90525792 | T/C | 0.273 | 0.043 | 0.085 | 0.613 | 0.273 | cis |
| CCL2 |  |  |  |  |  |  |  |  |  |  |
| rs76428106 | Diabetic polyneuropathy | 13 | 28604007 | C/T | 0.012 | 0.322 | 0.347 | 0.353 | 0.012 | trans |
| rs1671152 | Diabetic polyneuropathy | 19 | 55526345 | G/T | 0.882 | 0.165 | 0.116 | 0.154 | 0.119 | trans |
| rs7080386 | Diabetic polyneuropathy | 10 | 65048306 | A/C | 0.384 | -0.001 | 0.078 | 0.993 | 0.384 | trans |
| rs4541868 | Diabetic polyneuropathy | 8 | 106590705 | A/C | 0.226 | -0.001 | 0.090 | 0.990 | 0.226 | trans |
| rs10510751 | Diabetic polyneuropathy | 3 | 46346025 | G/T | 0.077 | -0.071 | 0.139 | 0.611 | 0.077 | trans |
| rs2228467 | Diabetic polyneuropathy | 3 | 42906116 | C/T | 0.076 | -0.071 | 0.143 | 0.617 | 0.076 | trans |
| SRP19 |  |  |  |  |  |  |  |  |  |  |
| rs1354034 | Diabetic polyneuropathy | 3 | 56849749 | C/T | 0.707 | 0.052 | 0.083 | 0.532 | 0.293 | trans |
| rs10512472 | Diabetic polyneuropathy | 17 | 33884804 | T/C | 0.820 | -0.067 | 0.098 | 0.494 | 0.180 | trans |
| RPS3 |  |  |  |  |  |  |  |  |  |  |
| rs1354034 | Diabetic polyneuropathy | 3 | 56849749 | C/T | 0.707 | 0.052 | 0.083 | 0.532 | 0.293 | trans |
| rs28929474 | Diabetic polyneuropathy | 14 | 94844947 | T/C | 0.020 | -0.002 | 0.272 | 0.993 | 0.020 | trans |
| rs11604127 | Diabetic polyneuropathy | 11 | 196944 | T/C | 0.296 | -0.004 | 0.084 | 0.963 | 0.296 | cis |
| rs10512472 | Diabetic polyneuropathy | 17 | 33884804 | T/C | 0.820 | -0.067 | 0.098 | 0.494 | 0.180 | trans |
| ZNRF3 |  |  |  |  |  |  |  |  |  |  |
| rs1354034 | Diabetic polyneuropathy | 3 | 56849749 | C/T | 0.707 | 0.052 | 0.083 | 0.532 | 0.293 | trans |
| rs11604127 | Diabetic polyneuropathy | 11 | 196944 | T/C | 0.296 | -0.004 | 0.084 | 0.963 | 0.296 | cis |
| rs10512472 | Diabetic polyneuropathy | 17 | 33884804 | T/C | 0.820 | -0.067 | 0.098 | 0.494 | 0.180 | trans |
| FLT1 |  |  |  |  |  |  |  |  |  |  |
| rs10513546 | Diabetic polyneuropathy | 3 | 159551651 | A/G | 0.231 | 0.095 | 0.089 | 0.286 | 0.231 | trans |
| rs114694170 | Diabetic polyneuropathy | 5 | 88180196 | C/T | 0.056 | 0.015 | 0.163 | 0.926 | 0.056 | trans |
| rs7098181 | Diabetic polyneuropathy | 10 | 65027143 | T/G | 0.385 | 0.000 | 0.078 | 0.999 | 0.385 | trans |
| BCAM |  |  |  |  |  |  |  |  |  |  |
| rs28399654 | Diabetic polyneuropathy | 19 | 45316588 | A/G | 0.015 | 0.359 | 0.306 | 0.242 | 0.015 | cis |
| rs8176672 | Diabetic polyneuropathy | 9 | 136142185 | T/C | 0.134 | 0.135 | 0.111 | 0.223 | 0.134 | trans |
| rs12522289 | Diabetic polyneuropathy | 5 | 177640864 | G/A | 0.121 | 0.106 | 0.117 | 0.366 | 0.121 | trans |
| rs10514710 | Diabetic polyneuropathy | 3 | 44883971 | A/G | 0.195 | 0.043 | 0.095 | 0.649 | 0.195 | trans |
| rs2410405 | Diabetic polyneuropathy | 21 | 42580363 | T/C | 0.328 | 0.023 | 0.080 | 0.777 | 0.328 | trans |
| rs73080552 | Diabetic polyneuropathy | 5 | 39435433 | A/G | 0.115 | 0.012 | 0.119 | 0.922 | 0.115 | trans |
| rs6592965 | Diabetic polyneuropathy | 7 | 50427982 | A/G | 0.321 | 0.011 | 0.081 | 0.894 | 0.321 | trans |
| rs56278466 | Diabetic polyneuropathy | 10 | 17875857 | G/T | 0.581 | -0.003 | 0.077 | 0.970 | 0.419 | trans |
| rs2137537 | Diabetic polyneuropathy | 12 | 71113087 | C/T | 0.640 | -0.044 | 0.079 | 0.581 | 0.360 | trans |
| rs2638282 | Diabetic polyneuropathy | 19 | 49213833 | A/G | 0.411 | -0.044 | 0.077 | 0.565 | 0.411 | trans |
| rs1260326 | Diabetic polyneuropathy | 2 | 27730940 | T/C | 0.351 | -0.100 | 0.079 | 0.208 | 0.351 | trans |
| CLEC3B |  |  |  |  |  |  |  |  |  |  |
| rs10514712 | Diabetic polyneuropathy | 3 | 45083928 | T/C | 0.802 | -0.014 | 0.094 | 0.886 | 0.198 | cis |
| MEPE |  |  |  |  |  |  |  |  |  |  |
| rs757980 | Diabetic polyneuropathy | 7 | 28725536 | A/G | 0.755 | 0.159 | 0.089 | 0.074 | 0.245 | trans |
| rs3008070 | Diabetic polyneuropathy | 13 | 23026451 | G/T | 0.197 | 0.104 | 0.095 | 0.276 | 0.197 | trans |
| rs77623237 | Diabetic polyneuropathy | 2 | 230114499 | C/T | 0.140 | 0.081 | 0.108 | 0.456 | 0.140 | trans |
| rs6547692 | Diabetic polyneuropathy | 2 | 27734972 | A/G | 0.601 | 0.060 | 0.077 | 0.441 | 0.399 | trans |
| rs28929474 | Diabetic polyneuropathy | 14 | 94844947 | T/C | 0.020 | -0.002 | 0.272 | 0.993 | 0.020 | trans |
| rs17013212 | Diabetic polyneuropathy | 4 | 88754060 | T/C | 0.202 | -0.008 | 0.096 | 0.937 | 0.202 | cis |
| rs806292 | Diabetic polyneuropathy | 13 | 50811031 | G/A | 0.581 | -0.025 | 0.076 | 0.747 | 0.419 | trans |
| rs55712380 | Diabetic polyneuropathy | 6 | 139610124 | T/G | 0.295 | -0.108 | 0.083 | 0.196 | 0.295 | trans |
| NCS1 |  |  |  |  |  |  |  |  |  |  |
| rs1054879 | Diabetic polyneuropathy | 9 | 132995768 | A/G | 0.581 | 0.029 | 0.080 | 0.714 | 0.419 | cis |
| rs2868346 | Diabetic polyneuropathy | 20 | 44547970 | T/C | 0.749 | -0.005 | 0.087 | 0.955 | 0.251 | trans |
| rs9807819 | Diabetic polyneuropathy | 19 | 46239598 | T/C | 0.299 | -0.071 | 0.083 | 0.391 | 0.299 | trans |
| rs662 | Diabetic polyneuropathy | 7 | 94937446 | C/T | 0.265 | -0.101 | 0.086 | 0.240 | 0.265 | trans |
| rs12342201 | Diabetic polyneuropathy | 9 | 95894964 | A/G | 0.418 | -0.103 | 0.076 | 0.177 | 0.418 | trans |
| rs11134475 | Diabetic polyneuropathy | 5 | 156399950 | G/A | 0.669 | -0.107 | 0.081 | 0.183 | 0.331 | trans |
| LAG3 |  |  |  |  |  |  |  |  |  |  |
| rs139974673 | Diabetic polyneuropathy | 15 | 44027885 | T/C | 0.995 | 0.444 | 0.516 | 0.389 | 0.005 | trans |
| rs77542162 | Diabetic polyneuropathy | 17 | 67081278 | A/G | 0.993 | 0.407 | 0.465 | 0.382 | 0.007 | trans |
| rs12740374 | Diabetic polyneuropathy | 1 | 109817590 | T/G | 0.215 | 0.109 | 0.092 | 0.235 | 0.215 | trans |
| rs35829610 | Diabetic polyneuropathy | 5 | 131823862 | CTG/C | 0.330 | 0.095 | 0.081 | 0.239 | 0.330 | trans |
| rs950802 | Diabetic polyneuropathy | 11 | 60152584 | A/G | 0.252 | 0.040 | 0.087 | 0.643 | 0.252 | trans |
| rs11773763 | Diabetic polyneuropathy | 7 | 50271499 | T/C | 0.314 | 0.026 | 0.081 | 0.753 | 0.314 | trans |
| rs1056008 | Diabetic polyneuropathy | 12 | 662838 | C/T | 0.242 | -0.004 | 0.088 | 0.960 | 0.242 | trans |
| rs597808 | Diabetic polyneuropathy | 12 | 111973358 | G/A | 0.586 | -0.054 | 0.077 | 0.480 | 0.415 | trans |
| HSPBP1 |  |  |  |  |  |  |  |  |  |  |
| rs7073297 | Diabetic polyneuropathy | 10 | 7754168 | T/C | 0.203 | 0.078 | 0.095 | 0.413 | 0.203 | trans |
| rs1056522 | Diabetic polyneuropathy | 3 | 126261345 | A/G | 0.320 | 0.073 | 0.081 | 0.369 | 0.320 | trans |
| rs72801433 | Diabetic polyneuropathy | 5 | 132360653 | A/G | 0.947 | -0.059 | 0.167 | 0.725 | 0.053 | trans |
| IGFBP1 |  |  |  |  |  |  |  |  |  |  |
| rs1260326 | Diabetic polyneuropathy | 2 | 27730940 | T/C | 0.351 | -0.100 | 0.079 | 0.208 | 0.351 | trans |
| CEACAM8 |  |  |  |  |  |  |  |  |  |  |
| rs12459454 | Diabetic polyneuropathy | 19 | 42263143 | G/A | 0.299 | 0.004 | 0.083 | 0.966 | 0.299 | cis |
| rs3774298 | Diabetic polyneuropathy | 3 | 187460099 | A/G | 0.627 | -0.004 | 0.080 | 0.958 | 0.373 | trans |
| rs12675298 | Diabetic polyneuropathy | 8 | 6828813 | A/G | 0.007 | -0.107 | 0.438 | 0.807 | 0.007 | trans |
| rs6503533 | Diabetic polyneuropathy | 17 | 38184580 | T/C | 0.611 | -0.132 | 0.077 | 0.088 | 0.390 | trans |
| rs112694524 | Diabetic polyneuropathy | 2 | 43453721 | A/G | 0.033 | -0.167 | 0.211 | 0.429 | 0.033 | trans |
| rs114641762 | Diabetic polyneuropathy | 17 | 42417164 | A/C | 0.108 | -0.177 | 0.123 | 0.150 | 0.108 | trans |
| rs2511241 | Diabetic polyneuropathy | 11 | 72945341 | T/C | 0.922 | -0.311 | 0.141 | 0.028 | 0.078 | trans |
| IL7R |  |  |  |  |  |  |  |  |  |  |
| rs1059091 | Diabetic polyneuropathy | 11 | 309127 | G/A | 0.321 | 0.103 | 0.082 | 0.206 | 0.321 | trans |
| rs170622 | Diabetic polyneuropathy | 7 | 35032100 | T/C | 0.229 | 0.059 | 0.090 | 0.510 | 0.229 | trans |
| rs6897932 | Diabetic polyneuropathy | 5 | 35874575 | T/C | 0.332 | 0.054 | 0.080 | 0.498 | 0.332 | cis |
| rs11083619 | Diabetic polyneuropathy | 19 | 41913267 | C/A | 0.644 | 0.052 | 0.079 | 0.507 | 0.356 | trans |
| rs2519093 | Diabetic polyneuropathy | 9 | 136141870 | T/C | 0.201 | 0.028 | 0.094 | 0.762 | 0.201 | trans |
| rs3791101 | Diabetic polyneuropathy | 1 | 44366250 | A/G | 0.304 | -0.021 | 0.083 | 0.796 | 0.304 | trans |
| rs35166255 | Diabetic polyneuropathy | 11 | 126301756 | A/G | 0.035 | -0.308 | 0.210 | 0.142 | 0.035 | trans |
| IGHE |  |  |  |  |  |  |  |  |  |  |
| rs2251746 | Diabetic polyneuropathy | 1 | 159272060 | T/C | 0.703 | -0.070 | 0.083 | 0.396 | 0.297 | trans |
| rs1059513 | Diabetic polyneuropathy | 12 | 57489709 | T/C | 0.953 | -0.205 | 0.177 | 0.247 | 0.047 | trans |
| CCDC134 |  |  |  |  |  |  |  |  |  |  |
| rs10608168 | Diabetic polyneuropathy | 22 | 42230349 | T/TCA | 0.591 | 0.067 | 0.077 | 0.381 | 0.409 | cis |
| rs1354034 | Diabetic polyneuropathy | 3 | 56849749 | C/T | 0.707 | 0.052 | 0.083 | 0.532 | 0.293 | trans |
| rs3804749 | Diabetic polyneuropathy | 3 | 122833003 | T/C | 0.620 | 0.007 | 0.078 | 0.930 | 0.380 | trans |
| APBB2 |  |  |  |  |  |  |  |  |  |  |
| rs10750866 | Diabetic polyneuropathy | 11 | 57404779 | A/G | 0.703 | 0.054 | 0.083 | 0.517 | 0.297 | trans |
| rs12146727 | Diabetic polyneuropathy | 12 | 7170336 | A/G | 0.173 | -0.046 | 0.100 | 0.651 | 0.173 | trans |
| CACNA2D3 |  |  |  |  |  |  |  |  |  |  |
| rs1063412 | Diabetic polyneuropathy | 1 | 172410967 | A/G | 0.588 | -0.008 | 0.077 | 0.922 | 0.412 | trans |
| CNTN3 |  |  |  |  |  |  |  |  |  |  |
| rs139974673 | Diabetic polyneuropathy | 15 | 44027885 | T/C | 0.995 | 0.444 | 0.516 | 0.389 | 0.005 | trans |
| rs77542162 | Diabetic polyneuropathy | 17 | 67081278 | A/G | 0.993 | 0.407 | 0.465 | 0.382 | 0.007 | trans |
| rs36124182 | Diabetic polyneuropathy | 12 | 24214934 | G/A | 0.021 | 0.326 | 0.261 | 0.213 | 0.021 | trans |
| rs33951980 | Diabetic polyneuropathy | 7 | 73029437 | T/C | 0.129 | 0.002 | 0.112 | 0.986 | 0.129 | trans |
| rs1063412 | Diabetic polyneuropathy | 1 | 172410967 | A/G | 0.588 | -0.008 | 0.077 | 0.922 | 0.412 | trans |
| rs34088055 | Diabetic polyneuropathy | 16 | 88535670 | T/C | 0.272 | -0.024 | 0.085 | 0.781 | 0.272 | trans |
| rs4677414 | Diabetic polyneuropathy | 3 | 74607020 | G/A | 0.955 | -0.091 | 0.180 | 0.614 | 0.045 | cis |
| rs1260326 | Diabetic polyneuropathy | 2 | 27730940 | T/C | 0.351 | -0.100 | 0.079 | 0.208 | 0.351 | trans |
| rs4921914 | Diabetic polyneuropathy | 8 | 18272438 | T/C | 0.750 | -0.113 | 0.087 | 0.192 | 0.251 | trans |
| F8 |  |  |  |  |  |  |  |  |  |  |
| rs1063857 | Diabetic polyneuropathy | 12 | 6153514 | A/G | 0.646 | 0.073 | 0.079 | 0.352 | 0.354 | trans |
| LPL |  |  |  |  |  |  |  |  |  |  |
| rs12600110 | Diabetic polyneuropathy | 16 | 962154 | C/T | 0.341 | 0.026 | 0.080 | 0.749 | 0.341 | trans |
| rs72959041 | Diabetic polyneuropathy | 6 | 127454893 | A/G | 0.065 | -0.020 | 0.157 | 0.898 | 0.065 | trans |
| rs2980888 | Diabetic polyneuropathy | 8 | 126507308 | C/T | 0.741 | -0.070 | 0.086 | 0.415 | 0.259 | trans |
| rs116843064 | Diabetic polyneuropathy | 19 | 8429323 | A/G | 0.027 | -0.093 | 0.230 | 0.685 | 0.027 | trans |
| rs1260326 | Diabetic polyneuropathy | 2 | 27730940 | T/C | 0.351 | -0.100 | 0.079 | 0.208 | 0.351 | trans |
| rs10649609 | Diabetic polyneuropathy | 2 | 165552706 | AAAAAT/A | 0.364 | -0.143 | 0.079 | 0.070 | 0.364 | trans |
| rs1801177 | Diabetic polyneuropathy | 8 | 19805708 | A/G | 0.003 | -0.453 | 0.715 | 0.526 | 0.003 | cis |
| ARSB |  |  |  |  |  |  |  |  |  |  |
| rs10778152 | Diabetic polyneuropathy | 12 | 102225751 | G/A | 0.283 | 0.173 | 0.084 | 0.039 | 0.283 | trans |
| rs10968020 | Diabetic polyneuropathy | 9 | 27628440 | C/T | 0.246 | 0.024 | 0.088 | 0.783 | 0.246 | trans |
| rs892090 | Diabetic polyneuropathy | 19 | 55539072 | T/G | 0.121 | -0.126 | 0.115 | 0.272 | 0.121 | trans |
| rs17622656 | Diabetic polyneuropathy | 5 | 131820997 | A/G | 0.260 | -0.133 | 0.086 | 0.122 | 0.260 | trans |
| IL18 |  |  |  |  |  |  |  |  |  |  |
| rs77762937 | Diabetic polyneuropathy | 2 | 203467470 | T/G | 0.235 | 0.081 | 0.089 | 0.361 | 0.235 | trans |
| rs7137828 | Diabetic polyneuropathy | 12 | 111932800 | T/C | 0.585 | -0.054 | 0.077 | 0.483 | 0.415 | trans |
| rs385076 | Diabetic polyneuropathy | 2 | 32489851 | C/T | 0.645 | -0.128 | 0.079 | 0.104 | 0.355 | trans |
| CNDP1 |  |  |  |  |  |  |  |  |  |  |
| rs8102710 | Diabetic polyneuropathy | 19 | 23870110 | T/G | 0.201 | 0.107 | 0.094 | 0.253 | 0.201 | trans |
| rs138016696 | Diabetic polyneuropathy | 19 | 27771210 | A/C | 0.144 | 0.104 | 0.108 | 0.333 | 0.144 | trans |
| rs10684444 | Diabetic polyneuropathy | 3 | 136298247 | C/CTATT | 0.155 | -0.002 | 0.103 | 0.987 | 0.155 | trans |
| rs17817077 | Diabetic polyneuropathy | 18 | 72209543 | A/G | 0.347 | -0.022 | 0.080 | 0.778 | 0.347 | cis |
| rs140646549 | Diabetic polyneuropathy | 15 | 43680031 | A/G | 0.006 | -0.297 | 0.503 | 0.556 | 0.005 | trans |
| CD248 |  |  |  |  |  |  |  |  |  |  |
| rs1070073 | Diabetic polyneuropathy | 12 | 104000319 | T/G | 0.338 | 0.002 | 0.080 | 0.977 | 0.338 | trans |
| rs1260326 | Diabetic polyneuropathy | 2 | 27730940 | T/C | 0.351 | -0.100 | 0.079 | 0.208 | 0.351 | trans |
| NLGN2 |  |  |  |  |  |  |  |  |  |  |
| rs1070073 | Diabetic polyneuropathy | 12 | 104000319 | T/G | 0.338 | 0.002 | 0.080 | 0.977 | 0.338 | trans |
| rs114576150 | Diabetic polyneuropathy | 17 | 7307950 | T/G | 0.834 | -0.012 | 0.103 | 0.911 | 0.166 | cis |
| rs17713196 | Diabetic polyneuropathy | 3 | 165489724 | T/C | 0.174 | -0.015 | 0.099 | 0.878 | 0.174 | trans |
| SEZ6L |  |  |  |  |  |  |  |  |  |  |
| rs137203 | Diabetic polyneuropathy | 22 | 26688831 | T/G | 0.036 | 0.039 | 0.206 | 0.848 | 0.036 | cis |
| rs10838702 | Diabetic polyneuropathy | 11 | 47410888 | T/G | 0.299 | 0.020 | 0.083 | 0.810 | 0.299 | trans |
| rs1070073 | Diabetic polyneuropathy | 12 | 104000319 | T/G | 0.338 | 0.002 | 0.080 | 0.977 | 0.338 | trans |
| rs60194243 | Diabetic polyneuropathy | 11 | 126247299 | T/C | 0.071 | -0.106 | 0.149 | 0.475 | 0.071 | trans |
| NRP2 |  |  |  |  |  |  |  |  |  |  |
| rs1070073 | Diabetic polyneuropathy | 12 | 104000319 | T/G | 0.338 | 0.002 | 0.080 | 0.977 | 0.338 | trans |
| rs55818129 | Diabetic polyneuropathy | 5 | 39425092 | G/T | 0.112 | -0.015 | 0.121 | 0.903 | 0.112 | trans |
| rs12995151 | Diabetic polyneuropathy | 2 | 62602438 | G/A | 0.131 | -0.043 | 0.110 | 0.696 | 0.131 | trans |
| rs2137537 | Diabetic polyneuropathy | 12 | 71113087 | C/T | 0.640 | -0.044 | 0.079 | 0.581 | 0.360 | trans |
| rs2289023 | Diabetic polyneuropathy | 2 | 205986321 | G/A | 0.285 | -0.087 | 0.083 | 0.294 | 0.285 | cis |
| LILRB2 |  |  |  |  |  |  |  |  |  |  |
| rs77542162 | Diabetic polyneuropathy | 17 | 67081278 | A/G | 0.993 | 0.407 | 0.465 | 0.382 | 0.007 | trans |
| rs10801559 | Diabetic polyneuropathy | 1 | 196704204 | A/G | 0.395 | 0.042 | 0.077 | 0.584 | 0.395 | trans |
| rs3747207 | Diabetic polyneuropathy | 22 | 44324855 | A/G | 0.226 | -0.010 | 0.090 | 0.915 | 0.226 | trans |
| rs1072160 | Diabetic polyneuropathy | 10 | 71194006 | A/G | 0.394 | -0.050 | 0.077 | 0.521 | 0.394 | trans |
| rs3184504 | Diabetic polyneuropathy | 12 | 111884608 | C/T | 0.591 | -0.051 | 0.077 | 0.504 | 0.409 | trans |
| rs383925 | Diabetic polyneuropathy | 19 | 54783521 | T/C | 0.275 | -0.086 | 0.085 | 0.310 | 0.275 | cis |
| rs2031902 | Diabetic polyneuropathy | 9 | 33117524 | T/C | 0.624 | -0.117 | 0.078 | 0.136 | 0.376 | trans |
| SCLY |  |  |  |  |  |  |  |  |  |  |
| rs58964858 | Diabetic polyneuropathy | 11 | 93816083 | T/C | 0.102 | 0.142 | 0.124 | 0.255 | 0.102 | trans |
| rs3747207 | Diabetic polyneuropathy | 22 | 44324855 | A/G | 0.226 | -0.010 | 0.090 | 0.915 | 0.226 | trans |
| rs112635299 | Diabetic polyneuropathy | 14 | 94838142 | T/G | 0.020 | -0.014 | 0.271 | 0.960 | 0.020 | trans |
| rs4835265 | Diabetic polyneuropathy | 4 | 146821410 | A/C | 0.172 | -0.047 | 0.101 | 0.641 | 0.172 | trans |
| rs10883451 | Diabetic polyneuropathy | 10 | 101924418 | C/T | 0.389 | -0.058 | 0.077 | 0.456 | 0.389 | trans |
| LBR |  |  |  |  |  |  |  |  |  |  |
| rs7080536 | Diabetic polyneuropathy | 10 | 115348046 | A/G | 0.028 | 0.086 | 0.226 | 0.702 | 0.028 | trans |
| rs1354034 | Diabetic polyneuropathy | 3 | 56849749 | C/T | 0.707 | 0.052 | 0.083 | 0.532 | 0.293 | trans |
| rs11604127 | Diabetic polyneuropathy | 11 | 196944 | T/C | 0.296 | -0.004 | 0.084 | 0.963 | 0.296 | cis |
| rs10876550 | Diabetic polyneuropathy | 12 | 54712308 | A/G | 0.677 | -0.046 | 0.081 | 0.569 | 0.323 | trans |
| rs10733789 | Diabetic polyneuropathy | 10 | 64948684 | C/T | 0.298 | -0.077 | 0.083 | 0.353 | 0.298 | trans |
| SESTD1 |  |  |  |  |  |  |  |  |  |  |
| rs1354034 | Diabetic polyneuropathy | 3 | 56849749 | C/T | 0.707 | 0.052 | 0.083 | 0.532 | 0.293 | trans |
| rs10733789 | Diabetic polyneuropathy | 10 | 64948684 | C/T | 0.298 | -0.077 | 0.083 | 0.353 | 0.298 | trans |
| F11R |  |  |  |  |  |  |  |  |  |  |
| rs1654425 | Diabetic polyneuropathy | 19 | 55538980 | C/T | 0.879 | 0.126 | 0.115 | 0.272 | 0.121 | trans |
| rs1354034 | Diabetic polyneuropathy | 3 | 56849749 | C/T | 0.707 | 0.052 | 0.083 | 0.532 | 0.293 | trans |
| rs3804749 | Diabetic polyneuropathy | 3 | 122833003 | T/C | 0.620 | 0.007 | 0.078 | 0.930 | 0.380 | trans |
| rs60315407 | Diabetic polyneuropathy | 1 | 161032805 | C/T | 0.359 | -0.035 | 0.079 | 0.658 | 0.359 | cis |
| rs11242109 | Diabetic polyneuropathy | 5 | 131677047 | T/G | 0.345 | -0.065 | 0.079 | 0.414 | 0.345 | trans |
| rs10733789 | Diabetic polyneuropathy | 10 | 64948684 | C/T | 0.298 | -0.077 | 0.083 | 0.353 | 0.298 | trans |
| VTA1 |  |  |  |  |  |  |  |  |  |  |
| rs1354034 | Diabetic polyneuropathy | 3 | 56849749 | C/T | 0.707 | 0.052 | 0.083 | 0.532 | 0.293 | trans |
| rs10733789 | Diabetic polyneuropathy | 10 | 64948684 | C/T | 0.298 | -0.077 | 0.083 | 0.353 | 0.298 | trans |
| rs10900809 | Diabetic polyneuropathy | 5 | 131826322 | A/G | 0.260 | -0.139 | 0.086 | 0.107 | 0.260 | trans |
| DNAJB1 |  |  |  |  |  |  |  |  |  |  |
| rs1354034 | Diabetic polyneuropathy | 3 | 56849749 | C/T | 0.707 | 0.052 | 0.083 | 0.532 | 0.293 | trans |
| rs9612021 | Diabetic polyneuropathy | 22 | 43583598 | C/T | 0.626 | 0.017 | 0.079 | 0.832 | 0.374 | trans |
| rs3811444 | Diabetic polyneuropathy | 1 | 248039451 | T/C | 0.346 | -0.037 | 0.080 | 0.645 | 0.346 | trans |
| rs11085896 | Diabetic polyneuropathy | 19 | 14651886 | C/A | 0.687 | -0.049 | 0.082 | 0.546 | 0.313 | cis |
| rs10733789 | Diabetic polyneuropathy | 10 | 64948684 | C/T | 0.298 | -0.077 | 0.083 | 0.353 | 0.298 | trans |
| ATP5IF1 |  |  |  |  |  |  |  |  |  |  |
| rs1354034 | Diabetic polyneuropathy | 3 | 56849749 | C/T | 0.707 | 0.052 | 0.083 | 0.532 | 0.293 | trans |
| rs10733789 | Diabetic polyneuropathy | 10 | 64948684 | C/T | 0.298 | -0.077 | 0.083 | 0.353 | 0.298 | trans |
| rs510379 | Diabetic polyneuropathy | 1 | 28535233 | C/T | 0.328 | -0.087 | 0.080 | 0.278 | 0.328 | cis |
| rs10849412 | Diabetic polyneuropathy | 12 | 6285846 | G/A | 0.386 | -0.090 | 0.078 | 0.250 | 0.386 | trans |
| rs17622656 | Diabetic polyneuropathy | 5 | 131820997 | A/G | 0.260 | -0.133 | 0.086 | 0.122 | 0.260 | trans |
| FXN |  |  |  |  |  |  |  |  |  |  |
| rs1354034 | Diabetic polyneuropathy | 3 | 56849749 | C/T | 0.707 | 0.052 | 0.083 | 0.532 | 0.293 | trans |
| rs10733789 | Diabetic polyneuropathy | 10 | 64948684 | C/T | 0.298 | -0.077 | 0.083 | 0.353 | 0.298 | trans |
| DUSP3 |  |  |  |  |  |  |  |  |  |  |
| rs10733789 | Diabetic polyneuropathy | 10 | 64948684 | C/T | 0.298 | -0.077 | 0.083 | 0.353 | 0.298 | trans |
| CIAPIN1 |  |  |  |  |  |  |  |  |  |  |
| rs1354034 | Diabetic polyneuropathy | 3 | 56849749 | C/T | 0.707 | 0.052 | 0.083 | 0.532 | 0.293 | trans |
| rs10733789 | Diabetic polyneuropathy | 10 | 64948684 | C/T | 0.298 | -0.077 | 0.083 | 0.353 | 0.298 | trans |
| AKT1S1 |  |  |  |  |  |  |  |  |  |  |
| rs1354034 | Diabetic polyneuropathy | 3 | 56849749 | C/T | 0.707 | 0.052 | 0.083 | 0.532 | 0.293 | trans |
| rs10733789 | Diabetic polyneuropathy | 10 | 64948684 | C/T | 0.298 | -0.077 | 0.083 | 0.353 | 0.298 | trans |
| STAMBP |  |  |  |  |  |  |  |  |  |  |
| rs1354034 | Diabetic polyneuropathy | 3 | 56849749 | C/T | 0.707 | 0.052 | 0.083 | 0.532 | 0.293 | trans |
| rs10733789 | Diabetic polyneuropathy | 10 | 64948684 | C/T | 0.298 | -0.077 | 0.083 | 0.353 | 0.298 | trans |
| VPS26A |  |  |  |  |  |  |  |  |  |  |
| rs1354034 | Diabetic polyneuropathy | 3 | 56849749 | C/T | 0.707 | 0.052 | 0.083 | 0.532 | 0.293 | trans |
| rs10736610 | Diabetic polyneuropathy | 11 | 134041884 | T/C | 0.408 | 0.048 | 0.077 | 0.534 | 0.408 | trans |
| CFH |  |  |  |  |  |  |  |  |  |  |
| rs10737680 | Diabetic polyneuropathy | 1 | 196679455 | C/A | 0.395 | 0.042 | 0.077 | 0.585 | 0.395 | cis |
| CPXM1 |  |  |  |  |  |  |  |  |  |  |
| rs1354034 | Diabetic polyneuropathy | 3 | 56849749 | C/T | 0.707 | 0.052 | 0.083 | 0.532 | 0.293 | trans |
| rs1434282 | Diabetic polyneuropathy | 1 | 199010721 | T/C | 0.711 | 0.030 | 0.083 | 0.720 | 0.290 | trans |
| rs215545 | Diabetic polyneuropathy | 20 | 2782015 | C/T | 0.750 | -0.018 | 0.087 | 0.835 | 0.250 | cis |
| rs12445050 | Diabetic polyneuropathy | 16 | 81870969 | T/C | 0.130 | -0.033 | 0.113 | 0.769 | 0.130 | trans |
| rs4759076 | Diabetic polyneuropathy | 12 | 54729872 | C/T | 0.581 | -0.046 | 0.077 | 0.546 | 0.419 | trans |
| rs4814837 | Diabetic polyneuropathy | 20 | 19241680 | T/C | 0.282 | -0.061 | 0.084 | 0.464 | 0.282 | trans |
| rs6961069 | Diabetic polyneuropathy | 7 | 80218961 | T/C | 0.417 | -0.076 | 0.077 | 0.324 | 0.417 | trans |
| rs61469632 | Diabetic polyneuropathy | 9 | 135861990 | C/T | 0.058 | -0.126 | 0.164 | 0.442 | 0.058 | trans |
| rs892090 | Diabetic polyneuropathy | 19 | 55539072 | T/G | 0.121 | -0.126 | 0.115 | 0.272 | 0.121 | trans |
| rs7618405 | Diabetic polyneuropathy | 3 | 18250509 | A/C | 0.211 | -0.178 | 0.093 | 0.055 | 0.211 | trans |
| rs13412535 | Diabetic polyneuropathy | 2 | 224874874 | A/G | 0.205 | -0.204 | 0.095 | 0.032 | 0.205 | trans |
| CD164 |  |  |  |  |  |  |  |  |  |  |
| rs4950771 | Diabetic polyneuropathy | 1 | 202111779 | G/T | 0.961 | 0.484 | 0.197 | 0.014 | 0.039 | trans |
| rs4734879 | Diabetic polyneuropathy | 8 | 106583124 | A/G | 0.780 | 0.005 | 0.091 | 0.959 | 0.220 | trans |
| rs45492299 | Diabetic polyneuropathy | 1 | 226054333 | A/G | 0.125 | -0.015 | 0.113 | 0.893 | 0.125 | trans |
| rs9857570 | Diabetic polyneuropathy | 3 | 58280690 | G/A | 0.404 | -0.016 | 0.077 | 0.839 | 0.404 | trans |
| rs6929023 | Diabetic polyneuropathy | 6 | 109720350 | A/G | 0.375 | -0.081 | 0.078 | 0.304 | 0.375 | cis |
| rs892090 | Diabetic polyneuropathy | 19 | 55539072 | T/G | 0.121 | -0.126 | 0.115 | 0.272 | 0.121 | trans |
| rs1692821 | Diabetic polyneuropathy | 8 | 11699988 | T/C | 0.211 | -0.163 | 0.104 | 0.117 | 0.211 | trans |
| rs72701845 | Diabetic polyneuropathy | 14 | 93217023 | A/G | 0.039 | -0.358 | 0.194 | 0.065 | 0.039 | trans |
| EGFL6 |  |  |  |  |  |  |  |  |  |  |
| rs3850140 | Diabetic polyneuropathy | 19 | 14805604 | T/C | 0.358 | 0.063 | 0.079 | 0.426 | 0.358 | trans |
| rs8084967 | Diabetic polyneuropathy | 18 | 22853862 | T/G | 0.273 | 0.051 | 0.085 | 0.551 | 0.273 | trans |
| THPO |  |  |  |  |  |  |  |  |  |  |
| rs1654425 | Diabetic polyneuropathy | 19 | 55538980 | C/T | 0.879 | 0.126 | 0.115 | 0.272 | 0.121 | trans |
| rs2305637 | Diabetic polyneuropathy | 3 | 47045846 | T/C | 0.234 | 0.017 | 0.089 | 0.849 | 0.234 | trans |
| rs28929474 | Diabetic polyneuropathy | 14 | 94844947 | T/C | 0.020 | -0.002 | 0.272 | 0.993 | 0.020 | trans |
| rs10762489 | Diabetic polyneuropathy | 10 | 73651458 | G/A | 0.263 | -0.005 | 0.086 | 0.956 | 0.263 | trans |
| rs12445050 | Diabetic polyneuropathy | 16 | 81870969 | T/C | 0.130 | -0.033 | 0.113 | 0.769 | 0.130 | trans |
| rs6580981 | Diabetic polyneuropathy | 12 | 54723028 | A/G | 0.582 | -0.054 | 0.077 | 0.484 | 0.419 | trans |
| rs11242109 | Diabetic polyneuropathy | 5 | 131677047 | T/G | 0.345 | -0.065 | 0.079 | 0.414 | 0.345 | trans |
| rs6141 | Diabetic polyneuropathy | 3 | 184090266 | T/C | 0.388 | -0.085 | 0.079 | 0.278 | 0.388 | cis |
| rs56385468 | Diabetic polyneuropathy | 11 | 1794676 | G/T | 0.051 | -0.183 | 0.172 | 0.290 | 0.051 | trans |
| PDGFB |  |  |  |  |  |  |  |  |  |  |
| rs1654425 | Diabetic polyneuropathy | 19 | 55538980 | C/T | 0.879 | 0.126 | 0.115 | 0.272 | 0.121 | trans |
| rs1354034 | Diabetic polyneuropathy | 3 | 56849749 | C/T | 0.707 | 0.052 | 0.083 | 0.532 | 0.293 | trans |
| rs1917342 | Diabetic polyneuropathy | 6 | 71349754 | G/A | 0.331 | -0.031 | 0.081 | 0.704 | 0.331 | trans |
| rs11639051 | Diabetic polyneuropathy | 15 | 101992998 | A/C | 0.257 | -0.032 | 0.087 | 0.713 | 0.257 | trans |
| rs12445050 | Diabetic polyneuropathy | 16 | 81870969 | T/C | 0.130 | -0.033 | 0.113 | 0.769 | 0.130 | trans |
| rs34377578 | Diabetic polyneuropathy | 10 | 104336426 | A/C | 0.804 | -0.034 | 0.095 | 0.723 | 0.196 | trans |
| rs3827978 | Diabetic polyneuropathy | 20 | 19281291 | T/C | 0.281 | -0.059 | 0.084 | 0.479 | 0.281 | trans |
| rs6961069 | Diabetic polyneuropathy | 7 | 80218961 | T/C | 0.417 | -0.076 | 0.077 | 0.324 | 0.417 | trans |
| rs61469632 | Diabetic polyneuropathy | 9 | 135861990 | C/T | 0.058 | -0.126 | 0.164 | 0.442 | 0.058 | trans |
| rs68066031 | Diabetic polyneuropathy | 2 | 224880498 | C/T | 0.204 | -0.200 | 0.095 | 0.036 | 0.204 | trans |
| rs75699653 | Diabetic polyneuropathy | 1 | 156871995 | T/C | 0.015 | -0.228 | 0.301 | 0.450 | 0.015 | trans |
| VEGFA |  |  |  |  |  |  |  |  |  |  |
| rs114694170 | Diabetic polyneuropathy | 5 | 88180196 | C/T | 0.056 | 0.015 | 0.163 | 0.926 | 0.056 | trans |
| ASAH2 |  |  |  |  |  |  |  |  |  |  |
| rs10740617 | Diabetic polyneuropathy | 10 | 52027609 | C/A | 0.796 | -0.134 | 0.094 | 0.156 | 0.204 | cis |
| WIF1 |  |  |  |  |  |  |  |  |  |  |
| rs28457828 | Diabetic polyneuropathy | 8 | 55451142 | T/C | 0.233 | 0.191 | 0.089 | 0.032 | 0.233 | trans |
| rs10741178 | Diabetic polyneuropathy | 10 | 130903004 | C/T | 0.804 | 0.140 | 0.095 | 0.140 | 0.196 | trans |
| rs117068593 | Diabetic polyneuropathy | 14 | 93118229 | T/C | 0.142 | 0.094 | 0.109 | 0.390 | 0.142 | trans |
| rs7949566 | Diabetic polyneuropathy | 11 | 126285301 | A/G | 0.361 | 0.047 | 0.078 | 0.549 | 0.361 | trans |
| rs10963680 | Diabetic polyneuropathy | 9 | 18629283 | A/G | 0.219 | 0.041 | 0.092 | 0.651 | 0.219 | trans |
| rs2145943 | Diabetic polyneuropathy | 20 | 38563245 | G/A | 0.317 | 0.006 | 0.081 | 0.938 | 0.317 | trans |
| rs462010 | Diabetic polyneuropathy | 12 | 65338145 | A/G | 0.407 | -0.029 | 0.077 | 0.706 | 0.407 | cis |
| rs6489548 | Diabetic polyneuropathy | 12 | 588604 | G/A | 0.634 | -0.035 | 0.078 | 0.656 | 0.366 | trans |
| rs1414660 | Diabetic polyneuropathy | 1 | 240586695 | T/C | 0.128 | -0.065 | 0.113 | 0.566 | 0.128 | trans |
| rs12894709 | Diabetic polyneuropathy | 14 | 75441734 | A/G | 0.418 | -0.071 | 0.076 | 0.355 | 0.418 | trans |
| NAAA |  |  |  |  |  |  |  |  |  |  |
| rs10745925 | Diabetic polyneuropathy | 12 | 102218899 | C/T | 0.283 | 0.172 | 0.084 | 0.040 | 0.283 | trans |
| rs111981122 | Diabetic polyneuropathy | 4 | 76855106 | A/C | 0.734 | -0.142 | 0.086 | 0.097 | 0.266 | cis |
| CPQ |  |  |  |  |  |  |  |  |  |  |
| rs10745925 | Diabetic polyneuropathy | 12 | 102218899 | C/T | 0.283 | 0.172 | 0.084 | 0.040 | 0.283 | trans |
| ARSA |  |  |  |  |  |  |  |  |  |  |
| rs873697 | Diabetic polyneuropathy | 22 | 51064169 | A/G | 0.015 | 0.413 | 0.305 | 0.175 | 0.015 | cis |
| rs10745925 | Diabetic polyneuropathy | 12 | 102218899 | C/T | 0.283 | 0.172 | 0.084 | 0.040 | 0.283 | trans |
| rs58542926 | Diabetic polyneuropathy | 19 | 19379549 | T/C | 0.064 | 0.005 | 0.153 | 0.977 | 0.064 | trans |
| rs687339 | Diabetic polyneuropathy | 3 | 135932359 | T/C | 0.849 | -0.007 | 0.105 | 0.950 | 0.151 | trans |
| rs429358 | Diabetic polyneuropathy | 19 | 45411941 | C/T | 0.183 | -0.237 | 0.100 | 0.018 | 0.183 | trans |
| GXYLT1 |  |  |  |  |  |  |  |  |  |  |
| rs10745925 | Diabetic polyneuropathy | 12 | 102218899 | C/T | 0.283 | 0.172 | 0.084 | 0.040 | 0.283 | trans |
| rs139695917 | Diabetic polyneuropathy | 12 | 42527041 | T/C | 0.981 | 0.138 | 0.275 | 0.616 | 0.019 | cis |
| CD46 |  |  |  |  |  |  |  |  |  |  |
| rs77924615 | Diabetic polyneuropathy | 16 | 20392332 | A/G | 0.222 | 0.042 | 0.091 | 0.643 | 0.222 | trans |
| rs972570 | Diabetic polyneuropathy | 10 | 71272018 | T/C | 0.395 | 0.002 | 0.077 | 0.985 | 0.395 | trans |
| rs72704449 | Diabetic polyneuropathy | 1 | 179473273 | C/T | 0.065 | -0.202 | 0.157 | 0.197 | 0.065 | trans |
| CDH1 |  |  |  |  |  |  |  |  |  |  |
| rs2519093 | Diabetic polyneuropathy | 9 | 136141870 | T/C | 0.201 | 0.028 | 0.094 | 0.762 | 0.201 | trans |
| rs3814995 | Diabetic polyneuropathy | 19 | 36342212 | T/C | 0.361 | 0.011 | 0.080 | 0.894 | 0.361 | trans |
| rs10749609 | Diabetic polyneuropathy | 10 | 82249752 | A/G | 0.743 | -0.028 | 0.087 | 0.750 | 0.257 | trans |
| rs681343 | Diabetic polyneuropathy | 19 | 49206462 | T/C | 0.375 | -0.045 | 0.078 | 0.568 | 0.375 | trans |
| rs708686 | Diabetic polyneuropathy | 19 | 5840619 | T/C | 0.334 | -0.105 | 0.081 | 0.194 | 0.334 | trans |
| NXPH2 |  |  |  |  |  |  |  |  |  |  |
| rs6445035 | Diabetic polyneuropathy | 3 | 165480100 | A/G | 0.176 | 0.000 | 0.099 | 0.998 | 0.176 | trans |
| rs10750096 | Diabetic polyneuropathy | 11 | 116656788 | C/A | 0.082 | -0.061 | 0.137 | 0.657 | 0.082 | trans |
| rs887829 | Diabetic polyneuropathy | 2 | 234668570 | T/C | 0.393 | -0.077 | 0.077 | 0.318 | 0.393 | trans |
| rs429358 | Diabetic polyneuropathy | 19 | 45411941 | C/T | 0.183 | -0.237 | 0.100 | 0.018 | 0.183 | trans |
| ZNF275 |  |  |  |  |  |  |  |  |  |  |
| rs10750866 | Diabetic polyneuropathy | 11 | 57404779 | A/G | 0.703 | 0.054 | 0.083 | 0.517 | 0.297 | trans |
| CPM |  |  |  |  |  |  |  |  |  |  |
| rs10751647 | Diabetic polyneuropathy | 11 | 306884 | C/T | 0.333 | 0.020 | 0.081 | 0.803 | 0.333 | trans |
| rs56278466 | Diabetic polyneuropathy | 10 | 17875857 | G/T | 0.581 | -0.003 | 0.077 | 0.970 | 0.419 | trans |
| rs11118611 | Diabetic polyneuropathy | 1 | 220990473 | G/A | 0.322 | -0.109 | 0.081 | 0.178 | 0.322 | trans |
| rs7978197 | Diabetic polyneuropathy | 12 | 69326547 | T/C | 0.003 | -0.543 | 0.702 | 0.439 | 0.003 | cis |
| VOPP1 |  |  |  |  |  |  |  |  |  |  |
| rs117042408 | Diabetic polyneuropathy | 7 | 55623391 | A/G | 0.015 | 0.007 | 0.297 | 0.982 | 0.015 | cis |
| rs4810479 | Diabetic polyneuropathy | 20 | 44545048 | C/T | 0.253 | 0.000 | 0.087 | 0.999 | 0.253 | trans |
| rs1800588 | Diabetic polyneuropathy | 15 | 58723675 | T/C | 0.249 | -0.026 | 0.088 | 0.764 | 0.249 | trans |
| rs2686395 | Diabetic polyneuropathy | 3 | 165485019 | T/C | 0.246 | -0.049 | 0.087 | 0.576 | 0.246 | trans |
| SDCBP |  |  |  |  |  |  |  |  |  |  |
| rs1354034 | Diabetic polyneuropathy | 3 | 56849749 | C/T | 0.707 | 0.052 | 0.083 | 0.532 | 0.293 | trans |
| rs11557154 | Diabetic polyneuropathy | 9 | 34107505 | T/C | 0.132 | -0.026 | 0.112 | 0.819 | 0.132 | trans |
| IFNLR1 |  |  |  |  |  |  |  |  |  |  |
| rs1801689 | Diabetic polyneuropathy | 17 | 64210580 | C/A | 0.010 | -0.158 | 0.383 | 0.681 | 0.010 | trans |
| XCL2 |  |  |  |  |  |  |  |  |  |  |
| rs10753774 | Diabetic polyneuropathy | 1 | 168514008 | T/C | 0.385 | -0.093 | 0.078 | 0.234 | 0.385 | cis |
| rs34538474 | Diabetic polyneuropathy | 10 | 3139540 | A/G | 0.628 | -0.178 | 0.078 | 0.023 | 0.372 | trans |
| LTA_LTB |  |  |  |  |  |  |  |  |  |  |
| rs62295996 | Diabetic polyneuropathy | 3 | 165482064 | A/G | 0.174 | -0.015 | 0.099 | 0.878 | 0.174 | trans |
| rs2364485 | Diabetic polyneuropathy | 12 | 6514963 | A/C | 0.196 | -0.045 | 0.098 | 0.647 | 0.196 | trans |
| HTR7 |  |  |  |  |  |  |  |  |  |  |
| rs28929474 | Diabetic polyneuropathy | 14 | 94844947 | T/C | 0.020 | -0.002 | 0.272 | 0.993 | 0.020 | trans |
| MLEC |  |  |  |  |  |  |  |  |  |  |
| rs1339847 | Diabetic polyneuropathy | 1 | 248039294 | A/G | 0.103 | 0.085 | 0.123 | 0.490 | 0.103 | trans |
| HRK |  |  |  |  |  |  |  |  |  |  |
| rs1354034 | Diabetic polyneuropathy | 3 | 56849749 | C/T | 0.707 | 0.052 | 0.083 | 0.532 | 0.293 | trans |
| MAP2K2 |  |  |  |  |  |  |  |  |  |  |
| rs559054 | Diabetic polyneuropathy | 13 | 113800622 | T/C | 0.326 | 0.000 | 0.081 | 1.000 | 0.326 | trans |
| rs2232710 | Diabetic polyneuropathy | 14 | 94750486 | T/C | 0.981 | -0.316 | 0.283 | 0.264 | 0.019 | trans |
| FKBP4 |  |  |  |  |  |  |  |  |  |  |
| rs57120900 | Diabetic polyneuropathy | 12 | 2905888 | A/G | 0.964 | 0.167 | 0.202 | 0.409 | 0.036 | cis |
| rs1354034 | Diabetic polyneuropathy | 3 | 56849749 | C/T | 0.707 | 0.052 | 0.083 | 0.532 | 0.293 | trans |
| IFNAR1 |  |  |  |  |  |  |  |  |  |  |
| rs12720356 | Diabetic polyneuropathy | 19 | 10469975 | A/C | 0.918 | 0.064 | 0.138 | 0.643 | 0.082 | trans |
| KLK15 |  |  |  |  |  |  |  |  |  |  |
| rs1354034 | Diabetic polyneuropathy | 3 | 56849749 | C/T | 0.707 | 0.052 | 0.083 | 0.532 | 0.293 | trans |
| ULBP3 |  |  |  |  |  |  |  |  |  |  |
| rs1354034 | Diabetic polyneuropathy | 3 | 56849749 | C/T | 0.707 | 0.052 | 0.083 | 0.532 | 0.293 | trans |
| PA2G4 |  |  |  |  |  |  |  |  |  |  |
| rs1354034 | Diabetic polyneuropathy | 3 | 56849749 | C/T | 0.707 | 0.052 | 0.083 | 0.532 | 0.293 | trans |
| CARS |  |  |  |  |  |  |  |  |  |  |
| rs1354034 | Diabetic polyneuropathy | 3 | 56849749 | C/T | 0.707 | 0.052 | 0.083 | 0.532 | 0.293 | trans |
| USP15 |  |  |  |  |  |  |  |  |  |  |
| rs73139022 | Diabetic polyneuropathy | 12 | 62818841 | T/C | 0.938 | -0.229 | 0.155 | 0.140 | 0.062 | cis |
| VIP |  |  |  |  |  |  |  |  |  |  |
| rs1354034 | Diabetic polyneuropathy | 3 | 56849749 | C/T | 0.707 | 0.052 | 0.083 | 0.532 | 0.293 | trans |
| THOC1 |  |  |  |  |  |  |  |  |  |  |
| rs1354034 | Diabetic polyneuropathy | 3 | 56849749 | C/T | 0.707 | 0.052 | 0.083 | 0.532 | 0.293 | trans |
| L3MBTL2 |  |  |  |  |  |  |  |  |  |  |
| rs1354034 | Diabetic polyneuropathy | 3 | 56849749 | C/T | 0.707 | 0.052 | 0.083 | 0.532 | 0.293 | trans |
| SNTA1 |  |  |  |  |  |  |  |  |  |  |
| rs1354034 | Diabetic polyneuropathy | 3 | 56849749 | C/T | 0.707 | 0.052 | 0.083 | 0.532 | 0.293 | trans |
| COPS7B |  |  |  |  |  |  |  |  |  |  |
| rs17713196 | Diabetic polyneuropathy | 3 | 165489724 | T/C | 0.174 | -0.015 | 0.099 | 0.878 | 0.174 | trans |
| rs5167 | Diabetic polyneuropathy | 19 | 45448465 | G/T | 0.415 | -0.084 | 0.077 | 0.276 | 0.415 | trans |
| PRDX1 |  |  |  |  |  |  |  |  |  |  |
| rs2356552 | Diabetic polyneuropathy | 1 | 46009316 | T/C | 0.063 | 0.109 | 0.154 | 0.478 | 0.063 | cis |
| CMBL |  |  |  |  |  |  |  |  |  |  |
| rs11738492 | Diabetic polyneuropathy | 5 | 10307022 | T/C | 0.594 | 0.033 | 0.077 | 0.668 | 0.406 | cis |
| IL15 |  |  |  |  |  |  |  |  |  |  |
| rs1354034 | Diabetic polyneuropathy | 3 | 56849749 | C/T | 0.707 | 0.052 | 0.083 | 0.532 | 0.293 | trans |
| PDLIM4 |  |  |  |  |  |  |  |  |  |  |
| rs4877 | Diabetic polyneuropathy | 5 | 131607588 | T/G | 0.083 | -0.084 | 0.137 | 0.540 | 0.083 | cis |
| MLF1 |  |  |  |  |  |  |  |  |  |  |
| rs3917529 | Diabetic polyneuropathy | 7 | 94940235 | A/G | 0.735 | 0.099 | 0.086 | 0.251 | 0.265 | trans |
| MYZAP |  |  |  |  |  |  |  |  |  |  |
| rs7162622 | Diabetic polyneuropathy | 15 | 57857245 | T/C | 0.718 | 0.116 | 0.084 | 0.167 | 0.282 | cis |
| rs17713196 | Diabetic polyneuropathy | 3 | 165489724 | T/C | 0.174 | -0.015 | 0.099 | 0.878 | 0.174 | trans |
| PCDH8 |  |  |  |  |  |  |  |  |  |  |
| rs1354034 | Diabetic polyneuropathy | 3 | 56849749 | C/T | 0.707 | 0.052 | 0.083 | 0.532 | 0.293 | trans |
| TAC1 |  |  |  |  |  |  |  |  |  |  |
| rs78145257 | Diabetic polyneuropathy | 7 | 97366332 | A/G | 0.241 | 0.043 | 0.088 | 0.623 | 0.241 | cis |
| DHH |  |  |  |  |  |  |  |  |  |  |
| rs1354034 | Diabetic polyneuropathy | 3 | 56849749 | C/T | 0.707 | 0.052 | 0.083 | 0.532 | 0.293 | trans |
| QPRT |  |  |  |  |  |  |  |  |  |  |
| rs893522 | Diabetic polyneuropathy | 3 | 165560618 | A/C | 0.918 | -0.111 | 0.137 | 0.419 | 0.082 | trans |
| BMPER |  |  |  |  |  |  |  |  |  |  |
| rs16879245 | Diabetic polyneuropathy | 7 | 33804485 | A/G | 0.908 | -0.147 | 0.133 | 0.268 | 0.092 | cis |
| LAMA4 |  |  |  |  |  |  |  |  |  |  |
| rs143040759 | Diabetic polyneuropathy | 17 | 34093726 | A/G | 0.031 | 0.151 | 0.217 | 0.486 | 0.031 | trans |
| rs7980201 | Diabetic polyneuropathy | 12 | 7640589 | T/C | 0.132 | 0.136 | 0.114 | 0.233 | 0.132 | trans |
| rs13339274 | Diabetic polyneuropathy | 16 | 81875559 | T/C | 0.137 | 0.035 | 0.110 | 0.753 | 0.137 | trans |
| rs1917342 | Diabetic polyneuropathy | 6 | 71349754 | G/A | 0.331 | -0.031 | 0.081 | 0.704 | 0.331 | trans |
| rs4813351 | Diabetic polyneuropathy | 20 | 19268933 | T/C | 0.315 | -0.058 | 0.081 | 0.471 | 0.315 | trans |
| rs892090 | Diabetic polyneuropathy | 19 | 55539072 | T/G | 0.121 | -0.126 | 0.115 | 0.272 | 0.121 | trans |
| CCL26 |  |  |  |  |  |  |  |  |  |  |
| rs1654425 | Diabetic polyneuropathy | 19 | 55538980 | C/T | 0.879 | 0.126 | 0.115 | 0.272 | 0.121 | trans |
| rs200838 | Diabetic polyneuropathy | 5 | 131710399 | C/A | 0.655 | 0.073 | 0.079 | 0.355 | 0.345 | trans |
| rs13412535 | Diabetic polyneuropathy | 2 | 224874874 | A/G | 0.205 | -0.204 | 0.095 | 0.032 | 0.205 | trans |
| CXCL5 |  |  |  |  |  |  |  |  |  |  |
| rs74035509 | Diabetic polyneuropathy | 16 | 88567333 | T/C | 0.053 | 0.181 | 0.169 | 0.283 | 0.053 | trans |
| rs352045 | Diabetic polyneuropathy | 4 | 74864687 | T/G | 0.112 | 0.039 | 0.120 | 0.747 | 0.112 | cis |
| rs456207 | Diabetic polyneuropathy | 9 | 136911140 | A/G | 0.396 | 0.022 | 0.077 | 0.776 | 0.396 | trans |
| CCL5 |  |  |  |  |  |  |  |  |  |  |
| rs2107538 | Diabetic polyneuropathy | 17 | 34207780 | T/C | 0.173 | 0.197 | 0.100 | 0.048 | 0.173 | cis |
| rs1654425 | Diabetic polyneuropathy | 19 | 55538980 | C/T | 0.879 | 0.126 | 0.115 | 0.272 | 0.121 | trans |
| rs1354034 | Diabetic polyneuropathy | 3 | 56849749 | C/T | 0.707 | 0.052 | 0.083 | 0.532 | 0.293 | trans |
| rs12445050 | Diabetic polyneuropathy | 16 | 81870969 | T/C | 0.130 | -0.033 | 0.113 | 0.769 | 0.130 | trans |
| rs3827978 | Diabetic polyneuropathy | 20 | 19281291 | T/C | 0.281 | -0.059 | 0.084 | 0.479 | 0.281 | trans |
| rs6961069 | Diabetic polyneuropathy | 7 | 80218961 | T/C | 0.417 | -0.076 | 0.077 | 0.324 | 0.417 | trans |
| CCL28 |  |  |  |  |  |  |  |  |  |  |
| rs73000929 | Diabetic polyneuropathy | 11 | 113953622 | A/G | 0.031 | 0.048 | 0.224 | 0.830 | 0.031 | trans |
| rs635634 | Diabetic polyneuropathy | 9 | 136155000 | T/C | 0.200 | 0.020 | 0.095 | 0.833 | 0.200 | trans |
| CCN2 |  |  |  |  |  |  |  |  |  |  |
| rs7176023 | Diabetic polyneuropathy | 15 | 65101007 | C/T | 0.078 | 0.069 | 0.141 | 0.622 | 0.078 | trans |
| rs1354034 | Diabetic polyneuropathy | 3 | 56849749 | C/T | 0.707 | 0.052 | 0.083 | 0.532 | 0.293 | trans |
| rs79977579 | Diabetic polyneuropathy | 12 | 54694560 | A/C | 0.094 | 0.049 | 0.129 | 0.707 | 0.094 | trans |
| rs56045941 | Diabetic polyneuropathy | 21 | 36421331 | C/T | 0.182 | 0.034 | 0.099 | 0.733 | 0.182 | trans |
| rs1434282 | Diabetic polyneuropathy | 1 | 199010721 | T/C | 0.711 | 0.030 | 0.083 | 0.720 | 0.290 | trans |
| rs11075045 | Diabetic polyneuropathy | 16 | 9034716 | G/A | 0.151 | 0.028 | 0.105 | 0.789 | 0.151 | trans |
| rs1917342 | Diabetic polyneuropathy | 6 | 71349754 | G/A | 0.331 | -0.031 | 0.081 | 0.704 | 0.331 | trans |
| rs12445050 | Diabetic polyneuropathy | 16 | 81870969 | T/C | 0.130 | -0.033 | 0.113 | 0.769 | 0.130 | trans |
| rs4631576 | Diabetic polyneuropathy | 9 | 100705210 | T/C | 0.397 | -0.037 | 0.078 | 0.635 | 0.397 | trans |
| rs6081565 | Diabetic polyneuropathy | 20 | 19287904 | A/G | 0.281 | -0.059 | 0.084 | 0.479 | 0.281 | trans |
| rs892090 | Diabetic polyneuropathy | 19 | 55539072 | T/G | 0.121 | -0.126 | 0.115 | 0.272 | 0.121 | trans |
| rs13412535 | Diabetic polyneuropathy | 2 | 224874874 | A/G | 0.205 | -0.204 | 0.095 | 0.032 | 0.205 | trans |
| DLL3 |  |  |  |  |  |  |  |  |  |  |
| rs10761737 | Diabetic polyneuropathy | 10 | 65052205 | C/T | 0.385 | -0.004 | 0.078 | 0.958 | 0.385 | trans |
| BCL2L11 |  |  |  |  |  |  |  |  |  |  |
| rs71354995 | Diabetic polyneuropathy | 19 | 38791841 | G/A | 0.381 | 0.165 | 0.078 | 0.034 | 0.381 | trans |
| rs1354034 | Diabetic polyneuropathy | 3 | 56849749 | C/T | 0.707 | 0.052 | 0.083 | 0.532 | 0.293 | trans |
| rs1801020 | Diabetic polyneuropathy | 5 | 176836532 | A/G | 0.261 | -0.001 | 0.086 | 0.990 | 0.261 | trans |
| rs10761737 | Diabetic polyneuropathy | 10 | 65052205 | C/T | 0.385 | -0.004 | 0.078 | 0.958 | 0.385 | trans |
| rs5030062 | Diabetic polyneuropathy | 3 | 186454180 | C/A | 0.348 | -0.045 | 0.079 | 0.573 | 0.348 | trans |
| rs6961069 | Diabetic polyneuropathy | 7 | 80218961 | T/C | 0.417 | -0.076 | 0.077 | 0.324 | 0.417 | trans |
| rs892090 | Diabetic polyneuropathy | 19 | 55539072 | T/G | 0.121 | -0.126 | 0.115 | 0.272 | 0.121 | trans |
| rs13412535 | Diabetic polyneuropathy | 2 | 224874874 | A/G | 0.205 | -0.204 | 0.095 | 0.032 | 0.205 | trans |
| SYK |  |  |  |  |  |  |  |  |  |  |
| rs34221447 | Diabetic polyneuropathy | 9 | 93563884 | T/C | 0.185 | 0.055 | 0.097 | 0.572 | 0.185 | cis |
| rs1354034 | Diabetic polyneuropathy | 3 | 56849749 | C/T | 0.707 | 0.052 | 0.083 | 0.532 | 0.293 | trans |
| rs10761741 | Diabetic polyneuropathy | 10 | 65066186 | T/G | 0.385 | 0.000 | 0.078 | 0.999 | 0.385 | trans |
| VSIR |  |  |  |  |  |  |  |  |  |  |
| rs10762477 | Diabetic polyneuropathy | 10 | 73531069 | A/G | 0.865 | 0.026 | 0.111 | 0.813 | 0.136 | cis |
| rs10762476 | Diabetic polyneuropathy | 10 | 73530977 | A/C | 0.135 | -0.026 | 0.111 | 0.813 | 0.135 | cis |
| rs11692780 | Diabetic polyneuropathy | 2 | 219149163 | A/G | 0.692 | -0.114 | 0.082 | 0.163 | 0.308 | trans |
| HSD11B1 |  |  |  |  |  |  |  |  |  |  |
| rs1076485 | Diabetic polyneuropathy | 11 | 116772441 | T/C | 0.189 | 0.140 | 0.097 | 0.151 | 0.189 | trans |
| rs4240624 | Diabetic polyneuropathy | 8 | 9184231 | A/G | 0.857 | 0.119 | 0.116 | 0.307 | 0.143 | trans |
| rs141887102 | Diabetic polyneuropathy | 19 | 59077031 | C/A | 0.200 | 0.044 | 0.094 | 0.643 | 0.200 | trans |
| rs56156922 | Diabetic polyneuropathy | 16 | 56987369 | C/T | 0.280 | 0.041 | 0.085 | 0.629 | 0.280 | trans |
| rs695110 | Diabetic polyneuropathy | 11 | 75456581 | C/T | 0.237 | 0.033 | 0.089 | 0.714 | 0.237 | trans |
| rs78444298 | Diabetic polyneuropathy | 1 | 184672098 | A/G | 0.017 | 0.023 | 0.295 | 0.937 | 0.017 | trans |
| rs58542926 | Diabetic polyneuropathy | 19 | 19379549 | T/C | 0.064 | 0.005 | 0.153 | 0.977 | 0.064 | trans |
| rs28929474 | Diabetic polyneuropathy | 14 | 94844947 | T/C | 0.020 | -0.002 | 0.272 | 0.993 | 0.020 | trans |
| rs7542235 | Diabetic polyneuropathy | 1 | 196823613 | G/A | 0.131 | -0.119 | 0.110 | 0.277 | 0.131 | trans |
| rs174547 | Diabetic polyneuropathy | 11 | 61570783 | C/T | 0.414 | -0.120 | 0.077 | 0.119 | 0.414 | trans |
| rs1801689 | Diabetic polyneuropathy | 17 | 64210580 | C/A | 0.010 | -0.158 | 0.383 | 0.681 | 0.010 | trans |
| CALCA |  |  |  |  |  |  |  |  |  |  |
| rs10766197 | Diabetic polyneuropathy | 11 | 14921880 | A/G | 0.405 | 0.141 | 0.077 | 0.068 | 0.405 | cis |
| rs12619508 | Diabetic polyneuropathy | 2 | 113982040 | A/C | 0.403 | 0.059 | 0.077 | 0.447 | 0.403 | trans |
| rs10841569 | Diabetic polyneuropathy | 12 | 20734839 | G/A | 0.127 | 0.001 | 0.113 | 0.991 | 0.127 | trans |
| rs73995235 | Diabetic polyneuropathy | 2 | 233196537 | T/G | 0.059 | -0.037 | 0.159 | 0.817 | 0.059 | trans |
| rs3790163 | Diabetic polyneuropathy | 20 | 10647951 | G/A | 0.828 | -0.163 | 0.101 | 0.105 | 0.172 | trans |
| rs7008914 | Diabetic polyneuropathy | 8 | 25880400 | C/T | 0.297 | -0.190 | 0.083 | 0.022 | 0.297 | trans |
| CALCB |  |  |  |  |  |  |  |  |  |  |
| rs10766205 | Diabetic polyneuropathy | 11 | 15092320 | G/A | 0.189 | -0.058 | 0.096 | 0.541 | 0.189 | cis |
| CCL24 |  |  |  |  |  |  |  |  |  |  |
| rs10769256 | Diabetic polyneuropathy | 11 | 47378396 | T/C | 0.301 | 0.028 | 0.083 | 0.734 | 0.301 | trans |
| rs1121985 | Diabetic polyneuropathy | 16 | 79363079 | C/A | 0.674 | -0.065 | 0.081 | 0.422 | 0.326 | trans |
| rs2228467 | Diabetic polyneuropathy | 3 | 42906116 | C/T | 0.076 | -0.071 | 0.143 | 0.617 | 0.076 | trans |
| rs4876611 | Diabetic polyneuropathy | 8 | 116671848 | G/A | 0.691 | -0.104 | 0.082 | 0.206 | 0.309 | trans |
| rs2201150 | Diabetic polyneuropathy | 3 | 46258902 | C/T | 0.617 | -0.137 | 0.078 | 0.080 | 0.383 | trans |
| rs2024050 | Diabetic polyneuropathy | 7 | 75460393 | G/A | 0.928 | -0.340 | 0.146 | 0.020 | 0.073 | cis |
| VSIG4 |  |  |  |  |  |  |  |  |  |  |
| rs28640218 | Diabetic polyneuropathy | 16 | 20359267 | T/G | 0.233 | 0.062 | 0.089 | 0.484 | 0.233 | trans |
| rs10769256 | Diabetic polyneuropathy | 11 | 47378396 | T/C | 0.301 | 0.028 | 0.083 | 0.734 | 0.301 | trans |
| rs514591 | Diabetic polyneuropathy | 1 | 196640320 | A/G | 0.710 | -0.125 | 0.083 | 0.134 | 0.290 | trans |
| ST6GALNAC6 |  |  |  |  |  |  |  |  |  |  |
| rs10770 | Diabetic polyneuropathy | 3 | 186389559 | C/T | 0.096 | -0.036 | 0.129 | 0.779 | 0.096 | trans |
| ART4 |  |  |  |  |  |  |  |  |  |  |
| rs10772808 | Diabetic polyneuropathy | 12 | 14990587 | A/G | 0.303 | -0.117 | 0.082 | 0.153 | 0.303 | cis |
| CLEC4A |  |  |  |  |  |  |  |  |  |  |
| rs4760 | Diabetic polyneuropathy | 19 | 44153100 | G/A | 0.168 | 0.087 | 0.103 | 0.396 | 0.168 | trans |
| rs247617 | Diabetic polyneuropathy | 16 | 56990716 | A/C | 0.279 | 0.042 | 0.084 | 0.617 | 0.279 | trans |
| rs1253387 | Diabetic polyneuropathy | 10 | 99032968 | A/G | 0.315 | 0.034 | 0.081 | 0.680 | 0.315 | trans |
| rs10774624 | Diabetic polyneuropathy | 12 | 111833788 | A/G | 0.597 | -0.015 | 0.077 | 0.847 | 0.403 | trans |
| rs791357 | Diabetic polyneuropathy | 5 | 173205225 | C/T | 0.726 | -0.021 | 0.085 | 0.809 | 0.274 | trans |
| rs11775560 | Diabetic polyneuropathy | 8 | 61660163 | G/A | 0.194 | -0.032 | 0.095 | 0.736 | 0.194 | trans |
| rs327 | Diabetic polyneuropathy | 8 | 19819536 | G/T | 0.252 | -0.034 | 0.087 | 0.691 | 0.252 | trans |
| rs11603123 | Diabetic polyneuropathy | 11 | 126305495 | A/G | 0.035 | -0.318 | 0.210 | 0.131 | 0.035 | trans |
| rs117213717 | Diabetic polyneuropathy | 12 | 8278195 | A/G | 0.006 | -0.614 | 0.484 | 0.205 | 0.006 | cis |
| TMSB10 |  |  |  |  |  |  |  |  |  |  |
| rs1354034 | Diabetic polyneuropathy | 3 | 56849749 | C/T | 0.707 | 0.052 | 0.083 | 0.532 | 0.293 | trans |
| rs4632248 | Diabetic polyneuropathy | 19 | 54324995 | T/G | 0.199 | 0.043 | 0.095 | 0.652 | 0.199 | trans |
| rs10774624 | Diabetic polyneuropathy | 12 | 111833788 | A/G | 0.597 | -0.015 | 0.077 | 0.847 | 0.403 | trans |
| TYMP |  |  |  |  |  |  |  |  |  |  |
| rs131805 | Diabetic polyneuropathy | 22 | 50964153 | C/T | 0.818 | 0.022 | 0.098 | 0.824 | 0.182 | cis |
| rs10774624 | Diabetic polyneuropathy | 12 | 111833788 | A/G | 0.597 | -0.015 | 0.077 | 0.847 | 0.403 | trans |
| IL27 |  |  |  |  |  |  |  |  |  |  |
| rs4905 | Diabetic polyneuropathy | 19 | 4237067 | A/G | 0.693 | 0.082 | 0.082 | 0.318 | 0.307 | cis |
| rs12625762 | Diabetic polyneuropathy | 20 | 34158587 | A/G | 0.091 | 0.062 | 0.130 | 0.631 | 0.091 | trans |
| rs704 | Diabetic polyneuropathy | 17 | 26694861 | A/G | 0.420 | 0.049 | 0.076 | 0.519 | 0.420 | trans |
| rs10843390 | Diabetic polyneuropathy | 12 | 29496991 | T/C | 0.292 | 0.022 | 0.083 | 0.794 | 0.292 | trans |
| rs10774624 | Diabetic polyneuropathy | 12 | 111833788 | A/G | 0.597 | -0.015 | 0.077 | 0.847 | 0.403 | trans |
| rs11599750 | Diabetic polyneuropathy | 10 | 101805442 | T/C | 0.287 | -0.043 | 0.083 | 0.605 | 0.287 | trans |
| rs11711157 | Diabetic polyneuropathy | 3 | 194061826 | T/C | 0.350 | -0.051 | 0.079 | 0.524 | 0.350 | trans |
| rs9715769 | Diabetic polyneuropathy | 4 | 38774489 | A/C | 0.910 | -0.148 | 0.131 | 0.256 | 0.090 | trans |
| PLXNB2 |  |  |  |  |  |  |  |  |  |  |
| rs1126735 | Diabetic polyneuropathy | 3 | 58413408 | G/A | 0.240 | 0.062 | 0.089 | 0.484 | 0.240 | trans |
| rs2519093 | Diabetic polyneuropathy | 9 | 136141870 | T/C | 0.201 | 0.028 | 0.094 | 0.762 | 0.201 | trans |
| rs28929474 | Diabetic polyneuropathy | 14 | 94844947 | T/C | 0.020 | -0.002 | 0.272 | 0.993 | 0.020 | trans |
| rs56278466 | Diabetic polyneuropathy | 10 | 17875857 | G/T | 0.581 | -0.003 | 0.077 | 0.970 | 0.419 | trans |
| rs10774624 | Diabetic polyneuropathy | 12 | 111833788 | A/G | 0.597 | -0.015 | 0.077 | 0.847 | 0.403 | trans |
| rs7523508 | Diabetic polyneuropathy | 1 | 177937755 | G/A | 0.263 | -0.075 | 0.086 | 0.386 | 0.263 | trans |
| rs1260326 | Diabetic polyneuropathy | 2 | 27730940 | T/C | 0.351 | -0.100 | 0.079 | 0.208 | 0.351 | trans |
| rs1801689 | Diabetic polyneuropathy | 17 | 64210580 | C/A | 0.010 | -0.158 | 0.383 | 0.681 | 0.010 | trans |
| CD200R1 |  |  |  |  |  |  |  |  |  |  |
| rs6545928 | Diabetic polyneuropathy | 2 | 62592120 | C/T | 0.176 | 0.020 | 0.099 | 0.838 | 0.176 | trans |
| rs2233243 | Diabetic polyneuropathy | 11 | 60152482 | G/A | 0.229 | -0.001 | 0.090 | 0.995 | 0.229 | trans |
| rs10823374 | Diabetic polyneuropathy | 10 | 71201716 | G/A | 0.394 | -0.049 | 0.077 | 0.522 | 0.394 | trans |
| rs75071241 | Diabetic polyneuropathy | 11 | 126232186 | A/G | 0.071 | -0.090 | 0.148 | 0.544 | 0.071 | trans |
| rs16860233 | Diabetic polyneuropathy | 3 | 112642122 | T/C | 0.022 | -0.295 | 0.261 | 0.259 | 0.022 | cis |
| ITGB7 |  |  |  |  |  |  |  |  |  |  |
| rs12232003 | Diabetic polyneuropathy | 12 | 53593632 | C/T | 0.134 | 0.149 | 0.111 | 0.180 | 0.134 | cis |
| rs7161799 | Diabetic polyneuropathy | 15 | 58770523 | T/C | 0.043 | 0.120 | 0.187 | 0.522 | 0.043 | trans |
| rs9987289 | Diabetic polyneuropathy | 8 | 9183358 | G/A | 0.857 | 0.118 | 0.116 | 0.310 | 0.143 | trans |
| rs6771051 | Diabetic polyneuropathy | 3 | 169724228 | T/G | 0.413 | 0.068 | 0.077 | 0.375 | 0.413 | trans |
| rs635634 | Diabetic polyneuropathy | 9 | 136155000 | T/C | 0.200 | 0.020 | 0.095 | 0.833 | 0.200 | trans |
| rs343808 | Diabetic polyneuropathy | 1 | 111330007 | T/C | 0.260 | 0.002 | 0.087 | 0.982 | 0.260 | trans |
| rs4686445 | Diabetic polyneuropathy | 3 | 186627608 | C/T | 0.295 | -0.002 | 0.083 | 0.979 | 0.295 | trans |
| rs6796 | Diabetic polyneuropathy | 7 | 6502367 | C/T | 0.321 | -0.010 | 0.082 | 0.900 | 0.321 | trans |
| rs6759003 | Diabetic polyneuropathy | 2 | 62559205 | C/T | 0.661 | -0.123 | 0.080 | 0.124 | 0.339 | trans |
| AMIGO2 |  |  |  |  |  |  |  |  |  |  |
| rs1354034 | Diabetic polyneuropathy | 3 | 56849749 | C/T | 0.707 | 0.052 | 0.083 | 0.532 | 0.293 | trans |
| rs2519093 | Diabetic polyneuropathy | 9 | 136141870 | T/C | 0.201 | 0.028 | 0.094 | 0.762 | 0.201 | trans |
| rs4055121 | Diabetic polyneuropathy | 11 | 126232337 | T/C | 0.153 | -0.048 | 0.106 | 0.651 | 0.153 | trans |
| rs174564 | Diabetic polyneuropathy | 11 | 61588305 | G/A | 0.415 | -0.118 | 0.077 | 0.124 | 0.415 | trans |
| ERBB3 |  |  |  |  |  |  |  |  |  |  |
| rs13234131 | Diabetic polyneuropathy | 7 | 73025975 | G/A | 0.128 | 0.014 | 0.112 | 0.902 | 0.128 | trans |
| rs10822159 | Diabetic polyneuropathy | 10 | 65096250 | T/C | 0.385 | 0.000 | 0.078 | 1.000 | 0.385 | trans |
| rs1260326 | Diabetic polyneuropathy | 2 | 27730940 | T/C | 0.351 | -0.100 | 0.079 | 0.208 | 0.351 | trans |
| rs150844304 | Diabetic polyneuropathy | 15 | 43726625 | C/A | 0.005 | -0.223 | 0.528 | 0.673 | 0.005 | trans |
| UST |  |  |  |  |  |  |  |  |  |  |
| rs6796 | Diabetic polyneuropathy | 7 | 6502367 | C/T | 0.321 | -0.010 | 0.082 | 0.900 | 0.321 | trans |
| rs10776883 | Diabetic polyneuropathy | 9 | 138999156 | T/C | 0.368 | -0.145 | 0.078 | 0.065 | 0.368 | trans |
| NTRK3 |  |  |  |  |  |  |  |  |  |  |
| rs10777159 | Diabetic polyneuropathy | 12 | 89779344 | G/A | 0.255 | 0.103 | 0.087 | 0.235 | 0.255 | trans |
| rs35887873 | Diabetic polyneuropathy | 11 | 126219396 | C/T | 0.193 | 0.023 | 0.096 | 0.808 | 0.193 | trans |
| rs34324830 | Diabetic polyneuropathy | 1 | 93087855 | T/C | 0.116 | -0.050 | 0.118 | 0.671 | 0.116 | trans |
| rs3184504 | Diabetic polyneuropathy | 12 | 111884608 | C/T | 0.591 | -0.051 | 0.077 | 0.504 | 0.409 | trans |
| rs79445108 | Diabetic polyneuropathy | 14 | 104193024 | A/G | 0.030 | -0.168 | 0.220 | 0.446 | 0.030 | trans |
| GOLM2 |  |  |  |  |  |  |  |  |  |  |
| rs77542162 | Diabetic polyneuropathy | 17 | 67081278 | A/G | 0.993 | 0.407 | 0.465 | 0.382 | 0.007 | trans |
| rs550057 | Diabetic polyneuropathy | 9 | 136146597 | T/C | 0.302 | 0.128 | 0.082 | 0.121 | 0.302 | trans |
| rs13107325 | Diabetic polyneuropathy | 4 | 103188709 | T/C | 0.014 | 0.106 | 0.309 | 0.731 | 0.014 | trans |
| rs10777172 | Diabetic polyneuropathy | 12 | 89894267 | C/T | 0.695 | -0.128 | 0.082 | 0.118 | 0.306 | trans |
| BTD |  |  |  |  |  |  |  |  |  |  |
| rs10778142 | Diabetic polyneuropathy | 12 | 102093698 | A/C | 0.699 | -0.105 | 0.083 | 0.206 | 0.301 | trans |
| GLB1 |  |  |  |  |  |  |  |  |  |  |
| rs10778152 | Diabetic polyneuropathy | 12 | 102225751 | G/A | 0.283 | 0.173 | 0.084 | 0.039 | 0.283 | trans |
| rs7637133 | Diabetic polyneuropathy | 3 | 33138763 | A/C | 0.602 | 0.034 | 0.078 | 0.657 | 0.398 | cis |
| rs76778371 | Diabetic polyneuropathy | 6 | 160418050 | A/G | 0.085 | 0.008 | 0.138 | 0.956 | 0.085 | trans |
| rs58542926 | Diabetic polyneuropathy | 19 | 19379549 | T/C | 0.064 | 0.005 | 0.153 | 0.977 | 0.064 | trans |
| rs687339 | Diabetic polyneuropathy | 3 | 135932359 | T/C | 0.849 | -0.007 | 0.105 | 0.950 | 0.151 | trans |
| rs142042446 | Diabetic polyneuropathy | 19 | 45386467 | GTAA/G | 0.172 | -0.119 | 0.101 | 0.238 | 0.172 | trans |
| HEXB |  |  |  |  |  |  |  |  |  |  |
| rs10778152 | Diabetic polyneuropathy | 12 | 102225751 | G/A | 0.283 | 0.173 | 0.084 | 0.039 | 0.283 | trans |
| rs58542926 | Diabetic polyneuropathy | 19 | 19379549 | T/C | 0.064 | 0.005 | 0.153 | 0.977 | 0.064 | trans |
| rs13164140 | Diabetic polyneuropathy | 5 | 74030338 | A/G | 0.291 | -0.054 | 0.084 | 0.520 | 0.291 | cis |
| PLA2G15 |  |  |  |  |  |  |  |  |  |  |
| rs10778152 | Diabetic polyneuropathy | 12 | 102225751 | G/A | 0.283 | 0.173 | 0.084 | 0.039 | 0.283 | trans |
| rs58542926 | Diabetic polyneuropathy | 19 | 19379549 | T/C | 0.064 | 0.005 | 0.153 | 0.977 | 0.064 | trans |
| rs9346804 | Diabetic polyneuropathy | 6 | 160401028 | A/G | 0.124 | -0.047 | 0.116 | 0.687 | 0.124 | trans |
| rs3743588 | Diabetic polyneuropathy | 16 | 11836508 | A/G | 0.261 | -0.144 | 0.086 | 0.094 | 0.261 | trans |
| ARSK |  |  |  |  |  |  |  |  |  |  |
| rs17084933 | Diabetic polyneuropathy | 5 | 94942780 | T/C | 0.951 | 0.271 | 0.176 | 0.123 | 0.049 | cis |
| rs10778152 | Diabetic polyneuropathy | 12 | 102225751 | G/A | 0.283 | 0.173 | 0.084 | 0.039 | 0.283 | trans |
| rs1354034 | Diabetic polyneuropathy | 3 | 56849749 | C/T | 0.707 | 0.052 | 0.083 | 0.532 | 0.293 | trans |
| rs12818933 | Diabetic polyneuropathy | 12 | 8162100 | T/C | 0.668 | 0.034 | 0.080 | 0.672 | 0.332 | trans |
| rs4363479 | Diabetic polyneuropathy | 1 | 202112285 | A/G | 0.039 | -0.487 | 0.196 | 0.013 | 0.039 | trans |
| CTSD |  |  |  |  |  |  |  |  |  |  |
| rs10778152 | Diabetic polyneuropathy | 12 | 102225751 | G/A | 0.283 | 0.173 | 0.084 | 0.039 | 0.283 | trans |
| rs58542926 | Diabetic polyneuropathy | 19 | 19379549 | T/C | 0.064 | 0.005 | 0.153 | 0.977 | 0.064 | trans |
| rs1260326 | Diabetic polyneuropathy | 2 | 27730940 | T/C | 0.351 | -0.100 | 0.079 | 0.208 | 0.351 | trans |
| rs55861089 | Diabetic polyneuropathy | 11 | 1783757 | G/A | 0.055 | -0.224 | 0.166 | 0.178 | 0.055 | cis |
| FGFBP1 |  |  |  |  |  |  |  |  |  |  |
| rs77542162 | Diabetic polyneuropathy | 17 | 67081278 | A/G | 0.993 | 0.407 | 0.465 | 0.382 | 0.007 | trans |
| rs9968276 | Diabetic polyneuropathy | 4 | 15949551 | A/G | 0.271 | 0.199 | 0.086 | 0.020 | 0.271 | cis |
| rs7528419 | Diabetic polyneuropathy | 1 | 109817192 | G/A | 0.215 | 0.117 | 0.092 | 0.203 | 0.215 | trans |
| rs1077834 | Diabetic polyneuropathy | 15 | 58723479 | C/T | 0.258 | 0.001 | 0.087 | 0.988 | 0.258 | trans |
| rs6800909 | Diabetic polyneuropathy | 3 | 187501571 | G/T | 0.330 | -0.019 | 0.081 | 0.813 | 0.330 | trans |
| rs3184504 | Diabetic polyneuropathy | 12 | 111884608 | C/T | 0.591 | -0.051 | 0.077 | 0.504 | 0.409 | trans |
| rs1260326 | Diabetic polyneuropathy | 2 | 27730940 | T/C | 0.351 | -0.100 | 0.079 | 0.208 | 0.351 | trans |
| TACSTD2 |  |  |  |  |  |  |  |  |  |  |
| rs7333 | Diabetic polyneuropathy | 1 | 59041220 | T/C | 0.132 | 0.164 | 0.111 | 0.141 | 0.132 | cis |
| rs8176693 | Diabetic polyneuropathy | 9 | 136137657 | T/C | 0.134 | 0.135 | 0.111 | 0.223 | 0.134 | trans |
| rs12740374 | Diabetic polyneuropathy | 1 | 109817590 | T/G | 0.215 | 0.109 | 0.092 | 0.235 | 0.215 | trans |
| rs56156922 | Diabetic polyneuropathy | 16 | 56987369 | C/T | 0.280 | 0.041 | 0.085 | 0.629 | 0.280 | trans |
| rs1077834 | Diabetic polyneuropathy | 15 | 58723479 | C/T | 0.258 | 0.001 | 0.087 | 0.988 | 0.258 | trans |
| rs2868346 | Diabetic polyneuropathy | 20 | 44547970 | T/C | 0.749 | -0.005 | 0.087 | 0.955 | 0.251 | trans |
| rs72802342 | Diabetic polyneuropathy | 16 | 75234872 | A/C | 0.086 | -0.032 | 0.135 | 0.814 | 0.086 | trans |
| rs681343 | Diabetic polyneuropathy | 19 | 49206462 | T/C | 0.375 | -0.045 | 0.078 | 0.568 | 0.375 | trans |
| rs780093 | Diabetic polyneuropathy | 2 | 27742603 | T/C | 0.353 | -0.111 | 0.079 | 0.163 | 0.353 | trans |
| rs72835428 | Diabetic polyneuropathy | 17 | 47260650 | T/C | 0.176 | -0.175 | 0.100 | 0.078 | 0.176 | trans |
| PON3 |  |  |  |  |  |  |  |  |  |  |
| rs149867961 | Diabetic polyneuropathy | 7 | 95025744 | C/T | 0.015 | 0.202 | 0.309 | 0.514 | 0.015 | cis |
| rs4240624 | Diabetic polyneuropathy | 8 | 9184231 | A/G | 0.857 | 0.119 | 0.116 | 0.307 | 0.143 | trans |
| rs34415062 | Diabetic polyneuropathy | 19 | 59023166 | A/G | 0.178 | 0.077 | 0.098 | 0.432 | 0.178 | trans |
| rs247617 | Diabetic polyneuropathy | 16 | 56990716 | A/C | 0.279 | 0.042 | 0.084 | 0.617 | 0.279 | trans |
| rs34060476 | Diabetic polyneuropathy | 7 | 73037956 | G/A | 0.132 | 0.003 | 0.111 | 0.982 | 0.132 | trans |
| rs1077835 | Diabetic polyneuropathy | 15 | 58723426 | G/A | 0.258 | 0.001 | 0.087 | 0.988 | 0.258 | trans |
| rs6677604 | Diabetic polyneuropathy | 1 | 196686918 | A/G | 0.130 | -0.136 | 0.111 | 0.222 | 0.130 | trans |
| IL1RL2 |  |  |  |  |  |  |  |  |  |  |
| rs11928797 | Diabetic polyneuropathy | 3 | 33457493 | A/C | 0.121 | 0.102 | 0.117 | 0.385 | 0.121 | trans |
| rs10815254 | Diabetic polyneuropathy | 9 | 5630344 | T/C | 0.276 | 0.046 | 0.085 | 0.585 | 0.276 | trans |
| rs13203302 | Diabetic polyneuropathy | 6 | 96007698 | T/C | 0.061 | 0.032 | 0.156 | 0.838 | 0.061 | trans |
| rs28929474 | Diabetic polyneuropathy | 14 | 94844947 | T/C | 0.020 | -0.002 | 0.272 | 0.993 | 0.020 | trans |
| rs56278466 | Diabetic polyneuropathy | 10 | 17875857 | G/T | 0.581 | -0.003 | 0.077 | 0.970 | 0.419 | trans |
| rs7599 | Diabetic polyneuropathy | 19 | 36038390 | G/A | 0.677 | -0.061 | 0.081 | 0.451 | 0.323 | trans |
| rs62011287 | Diabetic polyneuropathy | 15 | 63791228 | G/A | 0.387 | -0.077 | 0.077 | 0.321 | 0.387 | trans |
| rs11065385 | Diabetic polyneuropathy | 12 | 121423386 | G/A | 0.647 | -0.102 | 0.080 | 0.201 | 0.353 | trans |
| GOLM1 |  |  |  |  |  |  |  |  |  |  |
| rs550057 | Diabetic polyneuropathy | 9 | 136146597 | T/C | 0.302 | 0.128 | 0.082 | 0.121 | 0.302 | trans |
| rs10780749 | Diabetic polyneuropathy | 9 | 88720605 | A/G | 0.062 | 0.086 | 0.156 | 0.583 | 0.062 | cis |
| rs492602 | Diabetic polyneuropathy | 19 | 49206417 | A/G | 0.625 | 0.045 | 0.078 | 0.566 | 0.375 | trans |
| LIMA1 |  |  |  |  |  |  |  |  |  |  |
| rs1354034 | Diabetic polyneuropathy | 3 | 56849749 | C/T | 0.707 | 0.052 | 0.083 | 0.532 | 0.293 | trans |
| rs10783342 | Diabetic polyneuropathy | 12 | 50628466 | T/C | 0.411 | -0.038 | 0.077 | 0.625 | 0.411 | cis |
| NPTX1 |  |  |  |  |  |  |  |  |  |  |
| rs10786548 | Diabetic polyneuropathy | 10 | 101256326 | A/G | 0.934 | 0.348 | 0.153 | 0.023 | 0.066 | trans |
| rs7280064 | Diabetic polyneuropathy | 21 | 16377650 | T/C | 0.176 | 0.095 | 0.098 | 0.335 | 0.176 | trans |
| rs62068268 | Diabetic polyneuropathy | 17 | 78539090 | T/C | 0.068 | 0.053 | 0.150 | 0.726 | 0.068 | cis |
| rs2519093 | Diabetic polyneuropathy | 9 | 136141870 | T/C | 0.201 | 0.028 | 0.094 | 0.762 | 0.201 | trans |
| rs78265569 | Diabetic polyneuropathy | 1 | 2146165 | A/C | 0.056 | 0.010 | 0.165 | 0.952 | 0.056 | trans |
| rs12025349 | Diabetic polyneuropathy | 1 | 213940781 | T/C | 0.694 | -0.006 | 0.082 | 0.944 | 0.306 | trans |
| rs4693802 | Diabetic polyneuropathy | 4 | 87979212 | G/A | 0.271 | -0.008 | 0.086 | 0.929 | 0.271 | trans |
| rs4806469 | Diabetic polyneuropathy | 19 | 55698245 | A/G | 0.236 | -0.084 | 0.089 | 0.347 | 0.236 | trans |
| rs10986338 | Diabetic polyneuropathy | 9 | 127191232 | A/G | 0.686 | -0.117 | 0.081 | 0.150 | 0.315 | trans |
| rs66833621 | Diabetic polyneuropathy | 6 | 6923834 | C/T | 0.224 | -0.190 | 0.091 | 0.037 | 0.224 | trans |
| PLEKHA1 |  |  |  |  |  |  |  |  |  |  |
| rs7832219 | Diabetic polyneuropathy | 8 | 106578977 | T/C | 0.780 | 0.013 | 0.091 | 0.889 | 0.220 | trans |
| rs10788274 | Diabetic polyneuropathy | 10 | 124060802 | A/G | 0.611 | -0.095 | 0.077 | 0.218 | 0.390 | cis |
| ITIH5 |  |  |  |  |  |  |  |  |  |  |
| rs28616296 | Diabetic polyneuropathy | 2 | 129119832 | A/C | 0.268 | 0.070 | 0.085 | 0.414 | 0.268 | trans |
| rs760715 | Diabetic polyneuropathy | 22 | 39862343 | C/T | 0.327 | -0.010 | 0.081 | 0.906 | 0.327 | trans |
| rs61830291 | Diabetic polyneuropathy | 1 | 221001142 | A/C | 0.885 | -0.118 | 0.119 | 0.320 | 0.115 | trans |
| rs10789510 | Diabetic polyneuropathy | 1 | 47950132 | A/G | 0.154 | -0.271 | 0.104 | 0.009 | 0.154 | trans |
| FCRL2 |  |  |  |  |  |  |  |  |  |  |
| rs112001035 | Diabetic polyneuropathy | 17 | 66823805 | A/G | 0.081 | 0.352 | 0.140 | 0.012 | 0.081 | trans |
| rs12554596 | Diabetic polyneuropathy | 9 | 37002142 | G/A | 0.127 | 0.060 | 0.113 | 0.595 | 0.127 | trans |
| rs12568320 | Diabetic polyneuropathy | 1 | 157744004 | G/A | 0.146 | 0.046 | 0.107 | 0.667 | 0.146 | cis |
| rs11085015 | Diabetic polyneuropathy | 19 | 3369572 | G/T | 0.801 | 0.035 | 0.097 | 0.718 | 0.199 | trans |
| rs72832854 | Diabetic polyneuropathy | 2 | 111590770 | G/T | 0.018 | 0.018 | 0.286 | 0.949 | 0.018 | trans |
| rs10791824 | Diabetic polyneuropathy | 11 | 65559266 | G/A | 0.638 | -0.079 | 0.079 | 0.315 | 0.362 | trans |
| rs5001409 | Diabetic polyneuropathy | 3 | 186735690 | C/A | 0.416 | -0.098 | 0.077 | 0.201 | 0.416 | trans |
| rs188468174 | Diabetic polyneuropathy | 1 | 25291697 | T/C | 0.004 | -0.166 | 0.540 | 0.758 | 0.004 | trans |
| PEAR1 |  |  |  |  |  |  |  |  |  |  |
| rs1354034 | Diabetic polyneuropathy | 3 | 56849749 | C/T | 0.707 | 0.052 | 0.083 | 0.532 | 0.293 | trans |
| rs4661012 | Diabetic polyneuropathy | 1 | 156885491 | T/G | 0.628 | -0.012 | 0.078 | 0.876 | 0.372 | cis |
| SEMA7A |  |  |  |  |  |  |  |  |  |  |
| rs73119306 | Diabetic polyneuropathy | 12 | 57826982 | G/A | 0.233 | 0.246 | 0.089 | 0.005 | 0.233 | trans |
| rs13135092 | Diabetic polyneuropathy | 4 | 103198082 | G/A | 0.018 | 0.142 | 0.281 | 0.612 | 0.018 | trans |
| rs12554596 | Diabetic polyneuropathy | 9 | 37002142 | G/A | 0.127 | 0.060 | 0.113 | 0.595 | 0.127 | trans |
| rs78994380 | Diabetic polyneuropathy | 15 | 74692412 | A/C | 0.076 | 0.058 | 0.145 | 0.687 | 0.076 | cis |
| rs56278466 | Diabetic polyneuropathy | 10 | 17875857 | G/T | 0.581 | -0.003 | 0.077 | 0.970 | 0.419 | trans |
| rs28716466 | Diabetic polyneuropathy | 4 | 201074 | C/T | 0.282 | -0.015 | 0.084 | 0.857 | 0.282 | trans |
| rs3184504 | Diabetic polyneuropathy | 12 | 111884608 | C/T | 0.591 | -0.051 | 0.077 | 0.504 | 0.409 | trans |
| rs485186 | Diabetic polyneuropathy | 19 | 49207206 | G/A | 0.417 | -0.080 | 0.077 | 0.296 | 0.417 | trans |
| NOTCH1 |  |  |  |  |  |  |  |  |  |  |
| rs4240624 | Diabetic polyneuropathy | 8 | 9184231 | A/G | 0.857 | 0.119 | 0.116 | 0.307 | 0.143 | trans |
| rs56278466 | Diabetic polyneuropathy | 10 | 17875857 | G/T | 0.581 | -0.003 | 0.077 | 0.970 | 0.419 | trans |
| rs1260326 | Diabetic polyneuropathy | 2 | 27730940 | T/C | 0.351 | -0.100 | 0.079 | 0.208 | 0.351 | trans |
| MMRN2 |  |  |  |  |  |  |  |  |  |  |
| rs17713196 | Diabetic polyneuropathy | 3 | 165489724 | T/C | 0.174 | -0.015 | 0.099 | 0.878 | 0.174 | trans |
| rs1079734 | Diabetic polyneuropathy | 22 | 33161552 | A/G | 0.592 | -0.051 | 0.077 | 0.509 | 0.408 | trans |
| GZMH |  |  |  |  |  |  |  |  |  |  |
| rs3732378 | Diabetic polyneuropathy | 3 | 39307162 | A/G | 0.159 | 0.037 | 0.103 | 0.724 | 0.159 | trans |
| rs56133626 | Diabetic polyneuropathy | 19 | 16441973 | A/G | 0.309 | -0.079 | 0.082 | 0.335 | 0.309 | trans |
| rs576785 | Diabetic polyneuropathy | 11 | 128053274 | C/T | 0.339 | -0.121 | 0.080 | 0.131 | 0.339 | trans |
| ENAH |  |  |  |  |  |  |  |  |  |  |
| rs10799316 | Diabetic polyneuropathy | 1 | 225668031 | A/G | 0.788 | 0.039 | 0.092 | 0.675 | 0.212 | cis |
| RGMA |  |  |  |  |  |  |  |  |  |  |
| rs117353460 | Diabetic polyneuropathy | 16 | 89306703 | T/C | 0.028 | 0.436 | 0.224 | 0.052 | 0.028 | trans |
| rs4841132 | Diabetic polyneuropathy | 8 | 9183596 | G/A | 0.857 | 0.118 | 0.116 | 0.309 | 0.143 | trans |
| rs13107325 | Diabetic polyneuropathy | 4 | 103188709 | T/C | 0.014 | 0.106 | 0.309 | 0.731 | 0.014 | trans |
| rs13147041 | Diabetic polyneuropathy | 4 | 83872675 | T/C | 0.335 | 0.097 | 0.081 | 0.232 | 0.335 | trans |
| rs1885372 | Diabetic polyneuropathy | 9 | 35838030 | A/G | 0.597 | 0.079 | 0.077 | 0.305 | 0.404 | trans |
| rs10800097 | Diabetic polyneuropathy | 1 | 165383793 | G/A | 0.303 | 0.068 | 0.082 | 0.407 | 0.303 | trans |
| rs11956061 | Diabetic polyneuropathy | 5 | 126245898 | T/C | 0.594 | 0.051 | 0.077 | 0.511 | 0.406 | trans |
| rs71149134 | Diabetic polyneuropathy | 16 | 20358347 | CT/C | 0.219 | 0.019 | 0.092 | 0.840 | 0.219 | trans |
| rs73597479 | Diabetic polyneuropathy | 19 | 35545385 | T/C | 0.053 | -0.010 | 0.170 | 0.955 | 0.053 | trans |
| rs705702 | Diabetic polyneuropathy | 12 | 56390636 | G/A | 0.302 | -0.020 | 0.082 | 0.808 | 0.302 | trans |
| rs7303452 | Diabetic polyneuropathy | 12 | 51307149 | C/T | 0.671 | -0.024 | 0.080 | 0.768 | 0.329 | trans |
| rs56058910 | Diabetic polyneuropathy | 15 | 73126038 | C/T | 0.258 | -0.057 | 0.087 | 0.510 | 0.258 | trans |
| rs1260326 | Diabetic polyneuropathy | 2 | 27730940 | T/C | 0.351 | -0.100 | 0.079 | 0.208 | 0.351 | trans |
| PILRA |  |  |  |  |  |  |  |  |  |  |
| rs2405442 | Diabetic polyneuropathy | 7 | 99971313 | T/C | 0.306 | 0.011 | 0.082 | 0.898 | 0.306 | cis |
| GPR68 |  |  |  |  |  |  |  |  |  |  |
| rs704 | Diabetic polyneuropathy | 17 | 26694861 | A/G | 0.420 | 0.049 | 0.076 | 0.519 | 0.420 | trans |
| ROR2 |  |  |  |  |  |  |  |  |  |  |
| rs10820900 | Diabetic polyneuropathy | 9 | 94495608 | T/C | 0.378 | -0.086 | 0.078 | 0.271 | 0.378 | cis |
| FGF10 |  |  |  |  |  |  |  |  |  |  |
| rs6542680 | Diabetic polyneuropathy | 2 | 3640142 | C/T | 0.351 | -0.070 | 0.081 | 0.384 | 0.351 | trans |
| SETD2 |  |  |  |  |  |  |  |  |  |  |
| rs1803274 | Diabetic polyneuropathy | 3 | 165491280 | T/C | 0.174 | -0.016 | 0.099 | 0.869 | 0.174 | trans |
| NUDT5 |  |  |  |  |  |  |  |  |  |  |
| rs7895525 | Diabetic polyneuropathy | 10 | 12250620 | T/C | 0.700 | 0.084 | 0.083 | 0.309 | 0.300 | cis |
| SEMA4F |  |  |  |  |  |  |  |  |  |  |
| rs17713196 | Diabetic polyneuropathy | 3 | 165489724 | T/C | 0.174 | -0.015 | 0.099 | 0.878 | 0.174 | trans |
| ANGPTL1 |  |  |  |  |  |  |  |  |  |  |
| rs117516437 | Diabetic polyneuropathy | 19 | 10226579 | T/C | 0.022 | -0.054 | 0.254 | 0.833 | 0.022 | trans |
| rs8178824 | Diabetic polyneuropathy | 17 | 64224775 | T/C | 0.009 | -0.140 | 0.389 | 0.718 | 0.009 | trans |
| AZGP1 |  |  |  |  |  |  |  |  |  |  |
| rs139974673 | Diabetic polyneuropathy | 15 | 44027885 | T/C | 0.995 | 0.444 | 0.516 | 0.389 | 0.005 | trans |
| rs61747728 | Diabetic polyneuropathy | 1 | 179526214 | T/C | 0.064 | -0.201 | 0.158 | 0.204 | 0.064 | trans |
| ZHX3 |  |  |  |  |  |  |  |  |  |  |
| rs141738059 | Diabetic polyneuropathy | 9 | 116832007 | C/T | 0.020 | -0.645 | 0.283 | 0.023 | 0.020 | trans |
| SPINK14 |  |  |  |  |  |  |  |  |  |  |
| rs704 | Diabetic polyneuropathy | 17 | 26694861 | A/G | 0.420 | 0.049 | 0.076 | 0.519 | 0.420 | trans |
| NRAC |  |  |  |  |  |  |  |  |  |  |
| rs704 | Diabetic polyneuropathy | 17 | 26694861 | A/G | 0.420 | 0.049 | 0.076 | 0.519 | 0.420 | trans |
| NDE1 |  |  |  |  |  |  |  |  |  |  |
| rs7206272 | Diabetic polyneuropathy | 16 | 15726293 | A/C | 0.751 | 0.064 | 0.088 | 0.464 | 0.249 | cis |
| HEPACAM2 |  |  |  |  |  |  |  |  |  |  |
| rs9534302 | Diabetic polyneuropathy | 13 | 46640055 | T/C | 0.380 | -0.017 | 0.078 | 0.829 | 0.380 | trans |
| CCL4L2_CCL4L1 |  |  |  |  |  |  |  |  |  |  |
| rs113010081 | Diabetic polyneuropathy | 3 | 46457412 | T/C | 0.871 | 0.044 | 0.113 | 0.697 | 0.129 | trans |
| rs8064426 | Diabetic polyneuropathy | 17 | 34819750 | A/G | 0.218 | -0.161 | 0.093 | 0.081 | 0.218 | cis |
| FBLN5 |  |  |  |  |  |  |  |  |  |  |
| rs2402088 | Diabetic polyneuropathy | 14 | 92388714 | A/G | 0.399 | -0.091 | 0.077 | 0.239 | 0.399 | cis |
| KIF23 |  |  |  |  |  |  |  |  |  |  |
| rs1354034 | Diabetic polyneuropathy | 3 | 56849749 | C/T | 0.707 | 0.052 | 0.083 | 0.532 | 0.293 | trans |
| NEGR1 |  |  |  |  |  |  |  |  |  |  |
| rs7137828 | Diabetic polyneuropathy | 12 | 111932800 | T/C | 0.585 | -0.054 | 0.077 | 0.483 | 0.415 | trans |
| NCALD |  |  |  |  |  |  |  |  |  |  |
| rs704 | Diabetic polyneuropathy | 17 | 26694861 | A/G | 0.420 | 0.049 | 0.076 | 0.519 | 0.420 | trans |
| PRPSAP1 |  |  |  |  |  |  |  |  |  |  |
| rs388283 | Diabetic polyneuropathy | 17 | 74335069 | A/G | 0.212 | -0.082 | 0.094 | 0.382 | 0.212 | cis |
| CLU |  |  |  |  |  |  |  |  |  |  |
| rs704 | Diabetic polyneuropathy | 17 | 26694861 | A/G | 0.420 | 0.049 | 0.076 | 0.519 | 0.420 | trans |
| rs6065904 | Diabetic polyneuropathy | 20 | 44534651 | A/G | 0.224 | 0.026 | 0.091 | 0.778 | 0.224 | trans |
| rs1126605 | Diabetic polyneuropathy | 12 | 7242204 | T/C | 0.079 | -0.176 | 0.138 | 0.204 | 0.079 | trans |
| ITLN1 |  |  |  |  |  |  |  |  |  |  |
| rs7532133 | Diabetic polyneuropathy | 1 | 160851534 | A/G | 0.322 | 0.058 | 0.081 | 0.470 | 0.322 | cis |
| POFUT1 |  |  |  |  |  |  |  |  |  |  |
| rs76143353 | Diabetic polyneuropathy | 20 | 30815755 | T/C | 0.048 | 0.293 | 0.177 | 0.099 | 0.048 | cis |
| rs1354034 | Diabetic polyneuropathy | 3 | 56849749 | C/T | 0.707 | 0.052 | 0.083 | 0.532 | 0.293 | trans |
| KCNE1L |  |  |  |  |  |  |  |  |  |  |
| rs17713088 | Diabetic polyneuropathy | 3 | 165488604 | T/G | 0.174 | -0.015 | 0.099 | 0.878 | 0.174 | trans |
| rs12283371 | Diabetic polyneuropathy | 11 | 11639915 | T/C | 0.396 | -0.053 | 0.077 | 0.493 | 0.396 | trans |
| HTR2A |  |  |  |  |  |  |  |  |  |  |
| rs1354034 | Diabetic polyneuropathy | 3 | 56849749 | C/T | 0.707 | 0.052 | 0.083 | 0.532 | 0.293 | trans |
| MYSM1 |  |  |  |  |  |  |  |  |  |  |
| rs704 | Diabetic polyneuropathy | 17 | 26694861 | A/G | 0.420 | 0.049 | 0.076 | 0.519 | 0.420 | trans |
| MDM1 |  |  |  |  |  |  |  |  |  |  |
| rs1354034 | Diabetic polyneuropathy | 3 | 56849749 | C/T | 0.707 | 0.052 | 0.083 | 0.532 | 0.293 | trans |
| LMOD1 |  |  |  |  |  |  |  |  |  |  |
| rs5759535 | Diabetic polyneuropathy | 22 | 23268390 | A/G | 0.045 | 0.048 | 0.179 | 0.789 | 0.045 | trans |
| NDUFS4 |  |  |  |  |  |  |  |  |  |  |
| rs1250258 | Diabetic polyneuropathy | 2 | 216300185 | T/C | 0.789 | -0.023 | 0.092 | 0.800 | 0.212 | trans |
| rs2635536 | Diabetic polyneuropathy | 14 | 20396665 | A/C | 0.131 | -0.254 | 0.118 | 0.031 | 0.131 | trans |
| ERCC1 |  |  |  |  |  |  |  |  |  |  |
| rs3136065 | Diabetic polyneuropathy | 16 | 14017448 | A/G | 0.295 | 0.018 | 0.083 | 0.827 | 0.295 | trans |
| VARS |  |  |  |  |  |  |  |  |  |  |
| rs17713196 | Diabetic polyneuropathy | 3 | 165489724 | T/C | 0.174 | -0.015 | 0.099 | 0.878 | 0.174 | trans |
| rs5167 | Diabetic polyneuropathy | 19 | 45448465 | G/T | 0.415 | -0.084 | 0.077 | 0.276 | 0.415 | trans |
| BACH1 |  |  |  |  |  |  |  |  |  |  |
| rs704 | Diabetic polyneuropathy | 17 | 26694861 | A/G | 0.420 | 0.049 | 0.076 | 0.519 | 0.420 | trans |
| rs78119247 | Diabetic polyneuropathy | 3 | 165499135 | G/GAT | 0.173 | -0.017 | 0.100 | 0.866 | 0.173 | trans |
| CKAP2 |  |  |  |  |  |  |  |  |  |  |
| rs17713196 | Diabetic polyneuropathy | 3 | 165489724 | T/C | 0.174 | -0.015 | 0.099 | 0.878 | 0.174 | trans |
| CDH3 |  |  |  |  |  |  |  |  |  |  |
| rs58477040 | Diabetic polyneuropathy | 14 | 95049667 | A/G | 0.663 | 0.096 | 0.080 | 0.227 | 0.337 | trans |
| rs4665972 | Diabetic polyneuropathy | 2 | 27598097 | C/T | 0.623 | 0.080 | 0.078 | 0.303 | 0.377 | trans |
| rs704 | Diabetic polyneuropathy | 17 | 26694861 | A/G | 0.420 | 0.049 | 0.076 | 0.519 | 0.420 | trans |
| CCL19 |  |  |  |  |  |  |  |  |  |  |
| rs62292950 | Diabetic polyneuropathy | 3 | 132197995 | T/G | 0.837 | -0.026 | 0.103 | 0.804 | 0.163 | trans |
| NOV |  |  |  |  |  |  |  |  |  |  |
| rs34112166 | Diabetic polyneuropathy | 8 | 120400532 | A/G | 0.171 | -0.139 | 0.100 | 0.164 | 0.171 | cis |
| FLRT3 |  |  |  |  |  |  |  |  |  |  |
| rs6135225 | Diabetic polyneuropathy | 20 | 14678135 | T/G | 0.282 | 0.004 | 0.084 | 0.960 | 0.282 | trans |
| CD58 |  |  |  |  |  |  |  |  |  |  |
| rs10801908 | Diabetic polyneuropathy | 1 | 117090493 | T/C | 0.176 | 0.208 | 0.100 | 0.037 | 0.176 | cis |
| rs9987289 | Diabetic polyneuropathy | 8 | 9183358 | G/A | 0.857 | 0.118 | 0.116 | 0.310 | 0.143 | trans |
| rs4760 | Diabetic polyneuropathy | 19 | 44153100 | G/A | 0.168 | 0.087 | 0.103 | 0.396 | 0.168 | trans |
| rs2519093 | Diabetic polyneuropathy | 9 | 136141870 | T/C | 0.201 | 0.028 | 0.094 | 0.762 | 0.201 | trans |
| rs3213563 | Diabetic polyneuropathy | 1 | 172412995 | C/T | 0.589 | 0.002 | 0.077 | 0.979 | 0.411 | trans |
| rs3967200 | Diabetic polyneuropathy | 11 | 126232385 | T/C | 0.153 | -0.048 | 0.106 | 0.651 | 0.153 | trans |
| rs1260326 | Diabetic polyneuropathy | 2 | 27730940 | T/C | 0.351 | -0.100 | 0.079 | 0.208 | 0.351 | trans |
| FGFR1 |  |  |  |  |  |  |  |  |  |  |
| rs532436 | Diabetic polyneuropathy | 9 | 136149830 | A/G | 0.201 | 0.018 | 0.094 | 0.848 | 0.201 | trans |
| rs3827211 | Diabetic polyneuropathy | 21 | 42619254 | G/A | 0.411 | 0.001 | 0.077 | 0.987 | 0.411 | trans |
| TNFRSF13B |  |  |  |  |  |  |  |  |  |  |
| rs10806425 | Diabetic polyneuropathy | 6 | 90926612 | A/C | 0.270 | 0.121 | 0.085 | 0.155 | 0.270 | trans |
| rs75587749 | Diabetic polyneuropathy | 12 | 7614940 | T/C | 0.078 | 0.071 | 0.139 | 0.612 | 0.078 | trans |
| rs113745074 | Diabetic polyneuropathy | 7 | 150942349 | C/T | 0.125 | -0.011 | 0.114 | 0.926 | 0.125 | trans |
| rs3804329 | Diabetic polyneuropathy | 6 | 106686427 | G/A | 0.232 | -0.118 | 0.090 | 0.190 | 0.232 | trans |
| rs34562254 | Diabetic polyneuropathy | 17 | 16842991 | A/G | 0.100 | -0.145 | 0.126 | 0.247 | 0.100 | trans |
| DPEP1 |  |  |  |  |  |  |  |  |  |  |
| rs8111600 | Diabetic polyneuropathy | 19 | 5821968 | T/C | 0.028 | -0.013 | 0.225 | 0.954 | 0.028 | trans |
| IGKV1-5 |  |  |  |  |  |  |  |  |  |  |
| rs4665972 | Diabetic polyneuropathy | 2 | 27598097 | C/T | 0.623 | 0.080 | 0.078 | 0.303 | 0.377 | trans |
| CANT1 |  |  |  |  |  |  |  |  |  |  |
| rs17739056 | Diabetic polyneuropathy | 17 | 76995205 | C/T | 0.095 | 0.156 | 0.129 | 0.226 | 0.095 | cis |
| rs1654425 | Diabetic polyneuropathy | 19 | 55538980 | C/T | 0.879 | 0.126 | 0.115 | 0.272 | 0.121 | trans |
| rs10813956 | Diabetic polyneuropathy | 9 | 33145543 | C/T | 0.404 | 0.095 | 0.077 | 0.215 | 0.404 | trans |
| rs1863622 | Diabetic polyneuropathy | 3 | 186396616 | C/T | 0.279 | 0.030 | 0.084 | 0.726 | 0.279 | trans |
| rs56278466 | Diabetic polyneuropathy | 10 | 17875857 | G/T | 0.581 | -0.003 | 0.077 | 0.970 | 0.419 | trans |
| rs61747728 | Diabetic polyneuropathy | 1 | 179526214 | T/C | 0.064 | -0.201 | 0.158 | 0.204 | 0.064 | trans |
| MEGF10 |  |  |  |  |  |  |  |  |  |  |
| rs77542162 | Diabetic polyneuropathy | 17 | 67081278 | A/G | 0.993 | 0.407 | 0.465 | 0.382 | 0.007 | trans |
| rs4841132 | Diabetic polyneuropathy | 8 | 9183596 | G/A | 0.857 | 0.118 | 0.116 | 0.309 | 0.143 | trans |
| rs2236653 | Diabetic polyneuropathy | 11 | 126283785 | T/C | 0.364 | 0.069 | 0.078 | 0.375 | 0.364 | trans |
| rs10813959 | Diabetic polyneuropathy | 9 | 33166234 | C/T | 0.375 | 0.055 | 0.078 | 0.479 | 0.375 | trans |
| rs632887 | Diabetic polyneuropathy | 12 | 3392351 | G/A | 0.386 | 0.042 | 0.078 | 0.589 | 0.386 | trans |
| rs6068599 | Diabetic polyneuropathy | 20 | 52259618 | C/T | 0.708 | -0.008 | 0.083 | 0.928 | 0.292 | trans |
| rs112394041 | Diabetic polyneuropathy | 5 | 126628584 | CT/C | 0.374 | -0.044 | 0.078 | 0.570 | 0.374 | cis |
| GPC1 |  |  |  |  |  |  |  |  |  |  |
| rs2625445 | Diabetic polyneuropathy | 8 | 61540328 | A/G | 0.163 | 0.154 | 0.101 | 0.130 | 0.163 | trans |
| rs56278466 | Diabetic polyneuropathy | 10 | 17875857 | G/T | 0.581 | -0.003 | 0.077 | 0.970 | 0.419 | trans |
| rs1856027 | Diabetic polyneuropathy | 1 | 93544131 | C/T | 0.643 | -0.033 | 0.079 | 0.671 | 0.358 | trans |
| rs4633028 | Diabetic polyneuropathy | 8 | 38909861 | G/A | 0.775 | -0.053 | 0.090 | 0.559 | 0.225 | trans |
| rs3796500 | Diabetic polyneuropathy | 4 | 129206983 | C/T | 0.233 | -0.086 | 0.090 | 0.338 | 0.233 | trans |
| rs737939 | Diabetic polyneuropathy | 22 | 30315416 | T/G | 0.349 | -0.150 | 0.079 | 0.057 | 0.349 | trans |
| rs1801689 | Diabetic polyneuropathy | 17 | 64210580 | C/A | 0.010 | -0.158 | 0.383 | 0.681 | 0.010 | trans |
| rs150816167 | Diabetic polyneuropathy | 1 | 179571862 | C/T | 0.064 | -0.189 | 0.158 | 0.233 | 0.064 | trans |
| rs77078946 | Diabetic polyneuropathy | 2 | 241403417 | G/T | 0.122 | -0.194 | 0.115 | 0.092 | 0.122 | cis |
| rs2273967 | Diabetic polyneuropathy | 1 | 230415293 | T/C | 0.186 | -0.225 | 0.097 | 0.020 | 0.186 | trans |
| rs143362987 | Diabetic polyneuropathy | 2 | 170967940 | A/G | 0.010 | -0.475 | 0.386 | 0.219 | 0.010 | trans |
| GDF2 |  |  |  |  |  |  |  |  |  |  |
| rs34008398 | Diabetic polyneuropathy | 2 | 69093413 | A/G | 0.008 | 0.625 | 0.424 | 0.140 | 0.008 | trans |
| rs77303550 | Diabetic polyneuropathy | 16 | 72079657 | T/C | 0.191 | 0.130 | 0.096 | 0.172 | 0.191 | trans |
| rs12284180 | Diabetic polyneuropathy | 11 | 126265501 | C/A | 0.195 | 0.117 | 0.095 | 0.219 | 0.195 | trans |
| rs12441560 | Diabetic polyneuropathy | 15 | 101985923 | A/G | 0.246 | 0.019 | 0.088 | 0.833 | 0.246 | trans |
| rs7152610 | Diabetic polyneuropathy | 14 | 94928472 | C/T | 0.868 | -0.047 | 0.112 | 0.678 | 0.132 | trans |
| rs3184504 | Diabetic polyneuropathy | 12 | 111884608 | C/T | 0.591 | -0.051 | 0.077 | 0.504 | 0.409 | trans |
| rs68066031 | Diabetic polyneuropathy | 2 | 224880498 | C/T | 0.204 | -0.200 | 0.095 | 0.036 | 0.204 | trans |
| PRSS3 |  |  |  |  |  |  |  |  |  |  |
| rs581752 | Diabetic polyneuropathy | 8 | 102505705 | A/G | 0.083 | 0.083 | 0.138 | 0.547 | 0.083 | trans |
| rs10821611 | Diabetic polyneuropathy | 10 | 52470348 | A/G | 0.594 | -0.031 | 0.077 | 0.692 | 0.406 | trans |
| rs6501457 | Diabetic polyneuropathy | 17 | 69209252 | T/C | 0.723 | -0.056 | 0.085 | 0.513 | 0.277 | trans |
| rs4804669 | Diabetic polyneuropathy | 19 | 12502457 | G/A | 0.791 | -0.134 | 0.095 | 0.161 | 0.209 | trans |
| ERBIN |  |  |  |  |  |  |  |  |  |  |
| rs17622656 | Diabetic polyneuropathy | 5 | 131820997 | A/G | 0.260 | -0.133 | 0.086 | 0.122 | 0.260 | trans |
| DCBLD2 |  |  |  |  |  |  |  |  |  |  |
| rs77542162 | Diabetic polyneuropathy | 17 | 67081278 | A/G | 0.993 | 0.407 | 0.465 | 0.382 | 0.007 | trans |
| rs117486964 | Diabetic polyneuropathy | 19 | 49982043 | G/A | 0.029 | 0.229 | 0.223 | 0.305 | 0.029 | trans |
| rs11078597 | Diabetic polyneuropathy | 17 | 1618363 | C/T | 0.189 | 0.013 | 0.096 | 0.891 | 0.189 | trans |
| rs58542926 | Diabetic polyneuropathy | 19 | 19379549 | T/C | 0.064 | 0.005 | 0.153 | 0.977 | 0.064 | trans |
| rs28929474 | Diabetic polyneuropathy | 14 | 94844947 | T/C | 0.020 | -0.002 | 0.272 | 0.993 | 0.020 | trans |
| rs56278466 | Diabetic polyneuropathy | 10 | 17875857 | G/T | 0.581 | -0.003 | 0.077 | 0.970 | 0.419 | trans |
| rs430881 | Diabetic polyneuropathy | 17 | 9598125 | G/T | 0.299 | -0.017 | 0.083 | 0.842 | 0.299 | trans |
| rs1672992 | Diabetic polyneuropathy | 19 | 35556729 | T/C | 0.860 | -0.059 | 0.109 | 0.587 | 0.140 | trans |
| rs602633 | Diabetic polyneuropathy | 1 | 109821511 | G/T | 0.783 | -0.095 | 0.092 | 0.299 | 0.217 | trans |
| rs174581 | Diabetic polyneuropathy | 11 | 61606683 | A/G | 0.415 | -0.126 | 0.077 | 0.101 | 0.415 | trans |
| rs12487717 | Diabetic polyneuropathy | 3 | 98605007 | A/G | 0.001 | -0.957 | 1.005 | 0.341 | 0.001 | cis |
| FKBP5 |  |  |  |  |  |  |  |  |  |  |
| rs1354034 | Diabetic polyneuropathy | 3 | 56849749 | C/T | 0.707 | 0.052 | 0.083 | 0.532 | 0.293 | trans |
| rs2817032 | Diabetic polyneuropathy | 6 | 35688619 | T/C | 0.733 | -0.052 | 0.086 | 0.543 | 0.267 | trans |
| rs17622656 | Diabetic polyneuropathy | 5 | 131820997 | A/G | 0.260 | -0.133 | 0.086 | 0.122 | 0.260 | trans |
| CGB2 |  |  |  |  |  |  |  |  |  |  |
| rs10822155 | Diabetic polyneuropathy | 10 | 65071215 | A/C | 0.385 | 0.000 | 0.078 | 0.997 | 0.385 | trans |
| rs1654447 | Diabetic polyneuropathy | 19 | 55570574 | A/G | 0.100 | -0.131 | 0.125 | 0.294 | 0.100 | trans |
| SCGB2A1 |  |  |  |  |  |  |  |  |  |  |
| rs10822155 | Diabetic polyneuropathy | 10 | 65071215 | A/C | 0.385 | 0.000 | 0.078 | 0.997 | 0.385 | trans |
| rs1706435 | Diabetic polyneuropathy | 3 | 165461476 | G/A | 0.310 | -0.079 | 0.082 | 0.331 | 0.310 | trans |
| MYC |  |  |  |  |  |  |  |  |  |  |
| rs10822155 | Diabetic polyneuropathy | 10 | 65071215 | A/C | 0.385 | 0.000 | 0.078 | 0.997 | 0.385 | trans |
| UGT2A1 |  |  |  |  |  |  |  |  |  |  |
| rs10822155 | Diabetic polyneuropathy | 10 | 65071215 | A/C | 0.385 | 0.000 | 0.078 | 0.997 | 0.385 | trans |
| rs13412535 | Diabetic polyneuropathy | 2 | 224874874 | A/G | 0.205 | -0.204 | 0.095 | 0.032 | 0.205 | trans |
| BMP4 |  |  |  |  |  |  |  |  |  |  |
| rs35887873 | Diabetic polyneuropathy | 11 | 126219396 | C/T | 0.193 | 0.023 | 0.096 | 0.808 | 0.193 | trans |
| rs10822155 | Diabetic polyneuropathy | 10 | 65071215 | A/C | 0.385 | 0.000 | 0.078 | 0.997 | 0.385 | trans |
| rs6123685 | Diabetic polyneuropathy | 20 | 55836040 | A/G | 0.251 | -0.152 | 0.089 | 0.086 | 0.251 | trans |
| PSMD10 |  |  |  |  |  |  |  |  |  |  |
| rs10822155 | Diabetic polyneuropathy | 10 | 65071215 | A/C | 0.385 | 0.000 | 0.078 | 0.997 | 0.385 | trans |
| rs1355538 | Diabetic polyneuropathy | 3 | 165505177 | A/G | 0.326 | -0.043 | 0.080 | 0.595 | 0.326 | trans |
| CD33 |  |  |  |  |  |  |  |  |  |  |
| rs2455069 | Diabetic polyneuropathy | 19 | 51728641 | G/A | 0.388 | 0.081 | 0.077 | 0.293 | 0.388 | cis |
| rs62165726 | Diabetic polyneuropathy | 2 | 134966562 | A/C | 0.026 | 0.074 | 0.243 | 0.760 | 0.026 | trans |
| rs74612335 | Diabetic polyneuropathy | 11 | 126238633 | C/T | 0.184 | -0.044 | 0.099 | 0.654 | 0.184 | trans |
| rs10823374 | Diabetic polyneuropathy | 10 | 71201716 | G/A | 0.394 | -0.049 | 0.077 | 0.522 | 0.394 | trans |
| LILRB1 |  |  |  |  |  |  |  |  |  |  |
| rs62165726 | Diabetic polyneuropathy | 2 | 134966562 | A/C | 0.026 | 0.074 | 0.243 | 0.760 | 0.026 | trans |
| rs10823375 | Diabetic polyneuropathy | 10 | 71201982 | G/A | 0.394 | -0.044 | 0.077 | 0.568 | 0.394 | trans |
| rs73021415 | Diabetic polyneuropathy | 11 | 126248207 | T/C | 0.071 | -0.113 | 0.148 | 0.447 | 0.071 | trans |
| SLITRK2 |  |  |  |  |  |  |  |  |  |  |
| rs78334147 | Diabetic polyneuropathy | 5 | 109017236 | T/C | 0.085 | 0.170 | 0.137 | 0.214 | 0.085 | trans |
| rs13107325 | Diabetic polyneuropathy | 4 | 103188709 | T/C | 0.014 | 0.106 | 0.309 | 0.731 | 0.014 | trans |
| rs2811708 | Diabetic polyneuropathy | 9 | 21973422 | T/G | 0.220 | 0.042 | 0.091 | 0.643 | 0.220 | trans |
| rs56278466 | Diabetic polyneuropathy | 10 | 17875857 | G/T | 0.581 | -0.003 | 0.077 | 0.970 | 0.419 | trans |
| rs1131773 | Diabetic polyneuropathy | 9 | 95840256 | G/A | 0.116 | -0.070 | 0.118 | 0.555 | 0.116 | trans |
| rs61830291 | Diabetic polyneuropathy | 1 | 221001142 | A/C | 0.885 | -0.118 | 0.119 | 0.320 | 0.115 | trans |
| PLA2G12B |  |  |  |  |  |  |  |  |  |  |
| rs12740374 | Diabetic polyneuropathy | 1 | 109817590 | T/G | 0.215 | 0.109 | 0.092 | 0.235 | 0.215 | trans |
| rs2269829 | Diabetic polyneuropathy | 7 | 94936129 | G/A | 0.262 | -0.086 | 0.087 | 0.321 | 0.262 | trans |
| rs1260326 | Diabetic polyneuropathy | 2 | 27730940 | T/C | 0.351 | -0.100 | 0.079 | 0.208 | 0.351 | trans |
| rs10823949 | Diabetic polyneuropathy | 10 | 74632924 | T/C | 0.040 | -0.329 | 0.192 | 0.087 | 0.040 | cis |
| CLEC4G |  |  |  |  |  |  |  |  |  |  |
| rs76560987 | Diabetic polyneuropathy | 19 | 7799452 | T/C | 0.076 | 0.153 | 0.143 | 0.287 | 0.076 | cis |
| rs17701598 | Diabetic polyneuropathy | 17 | 66320445 | A/G | 0.364 | 0.092 | 0.079 | 0.242 | 0.364 | trans |
| rs12709103 | Diabetic polyneuropathy | 16 | 88712782 | G/A | 0.801 | 0.081 | 0.094 | 0.391 | 0.199 | trans |
| rs56278466 | Diabetic polyneuropathy | 10 | 17875857 | G/T | 0.581 | -0.003 | 0.077 | 0.970 | 0.419 | trans |
| STC2 |  |  |  |  |  |  |  |  |  |  |
| rs2894602 | Diabetic polyneuropathy | 2 | 227249802 | G/A | 0.775 | 0.075 | 0.090 | 0.405 | 0.225 | trans |
| rs3184504 | Diabetic polyneuropathy | 12 | 111884608 | C/T | 0.591 | -0.051 | 0.077 | 0.504 | 0.409 | trans |
| rs12380110 | Diabetic polyneuropathy | 9 | 139086818 | C/T | 0.172 | -0.147 | 0.102 | 0.147 | 0.172 | trans |
| IL1R2 |  |  |  |  |  |  |  |  |  |  |
| rs77542162 | Diabetic polyneuropathy | 17 | 67081278 | A/G | 0.993 | 0.407 | 0.465 | 0.382 | 0.007 | trans |
| rs11045856 | Diabetic polyneuropathy | 12 | 21350689 | G/T | 0.172 | 0.098 | 0.100 | 0.326 | 0.172 | trans |
| rs10831245 | Diabetic polyneuropathy | 11 | 94319888 | A/G | 0.394 | 0.044 | 0.077 | 0.566 | 0.394 | trans |
| rs165316 | Diabetic polyneuropathy | 1 | 91533297 | G/A | 0.213 | -0.021 | 0.093 | 0.820 | 0.213 | trans |
| rs1870138 | Diabetic polyneuropathy | 10 | 82269611 | G/A | 0.778 | -0.046 | 0.091 | 0.616 | 0.223 | trans |
| rs11621792 | Diabetic polyneuropathy | 14 | 24871926 | T/C | 0.369 | -0.049 | 0.078 | 0.534 | 0.369 | trans |
| rs6973520 | Diabetic polyneuropathy | 7 | 128740443 | T/C | 0.373 | -0.065 | 0.078 | 0.406 | 0.373 | trans |
| rs629301 | Diabetic polyneuropathy | 1 | 109818306 | T/G | 0.785 | -0.109 | 0.092 | 0.235 | 0.215 | trans |
| MYBPC1 |  |  |  |  |  |  |  |  |  |  |
| rs80087033 | Diabetic polyneuropathy | 12 | 102048447 | A/G | 0.162 | -0.170 | 0.103 | 0.098 | 0.162 | cis |
| EGFLAM |  |  |  |  |  |  |  |  |  |  |
| rs12975366 | Diabetic polyneuropathy | 19 | 54759361 | C/T | 0.372 | 0.142 | 0.079 | 0.071 | 0.372 | trans |
| rs10838529 | Diabetic polyneuropathy | 11 | 45931502 | G/A | 0.300 | 0.042 | 0.083 | 0.616 | 0.300 | trans |
| rs78804971 | Diabetic polyneuropathy | 3 | 49821769 | C/T | 0.067 | -0.094 | 0.152 | 0.536 | 0.067 | trans |
| rs2740594 | Diabetic polyneuropathy | 8 | 11707174 | G/A | 0.206 | -0.133 | 0.104 | 0.204 | 0.206 | trans |
| rs113400125 | Diabetic polyneuropathy | 5 | 38406251 | T/C | 0.021 | -0.293 | 0.273 | 0.284 | 0.021 | cis |
| MRVI1 |  |  |  |  |  |  |  |  |  |  |
| rs10840457 | Diabetic polyneuropathy | 11 | 10675738 | A/G | 0.340 | 0.066 | 0.080 | 0.405 | 0.340 | cis |
| rs1354034 | Diabetic polyneuropathy | 3 | 56849749 | C/T | 0.707 | 0.052 | 0.083 | 0.532 | 0.293 | trans |
| GFRA3 |  |  |  |  |  |  |  |  |  |  |
| rs12027119 | Diabetic polyneuropathy | 1 | 60152685 | G/A | 0.655 | 0.119 | 0.079 | 0.132 | 0.346 | trans |
| rs13107325 | Diabetic polyneuropathy | 4 | 103188709 | T/C | 0.014 | 0.106 | 0.309 | 0.731 | 0.014 | trans |
| rs73158489 | Diabetic polyneuropathy | 3 | 153757862 | T/C | 0.199 | 0.021 | 0.095 | 0.829 | 0.199 | trans |
| rs2306333 | Diabetic polyneuropathy | 15 | 51217674 | G/A | 0.137 | 0.007 | 0.109 | 0.950 | 0.137 | trans |
| rs1161106 | Diabetic polyneuropathy | 12 | 67840467 | A/C | 0.187 | 0.001 | 0.097 | 0.990 | 0.187 | trans |
| rs56278466 | Diabetic polyneuropathy | 10 | 17875857 | G/T | 0.581 | -0.003 | 0.077 | 0.970 | 0.419 | trans |
| rs3744052 | Diabetic polyneuropathy | 17 | 74558057 | T/C | 0.188 | -0.026 | 0.097 | 0.786 | 0.188 | trans |
| rs10956755 | Diabetic polyneuropathy | 8 | 135302956 | G/A | 0.407 | -0.032 | 0.078 | 0.678 | 0.407 | trans |
| rs35383942 | Diabetic polyneuropathy | 1 | 201437832 | T/C | 0.116 | -0.046 | 0.120 | 0.699 | 0.116 | trans |
| rs1618875 | Diabetic polyneuropathy | 12 | 115744381 | T/C | 0.589 | -0.047 | 0.077 | 0.542 | 0.411 | trans |
| rs554743 | Diabetic polyneuropathy | 20 | 3662142 | T/C | 0.644 | -0.048 | 0.080 | 0.550 | 0.356 | trans |
| rs10840586 | Diabetic polyneuropathy | 11 | 11169808 | G/A | 0.420 | -0.048 | 0.077 | 0.529 | 0.420 | trans |
| rs12710696 | Diabetic polyneuropathy | 2 | 19320803 | C/T | 0.665 | -0.107 | 0.080 | 0.182 | 0.336 | trans |
| rs12654231 | Diabetic polyneuropathy | 5 | 109676706 | G/A | 0.065 | -0.209 | 0.155 | 0.177 | 0.065 | trans |
| IL17RB |  |  |  |  |  |  |  |  |  |  |
| rs77542162 | Diabetic polyneuropathy | 17 | 67081278 | A/G | 0.993 | 0.407 | 0.465 | 0.382 | 0.007 | trans |
| rs1169284 | Diabetic polyneuropathy | 12 | 121419926 | C/T | 0.357 | 0.116 | 0.079 | 0.145 | 0.357 | trans |
| rs10841753 | Diabetic polyneuropathy | 12 | 21321370 | C/T | 0.126 | 0.112 | 0.113 | 0.323 | 0.126 | trans |
| rs34346326 | Diabetic polyneuropathy | 7 | 73016181 | C/T | 0.177 | -0.001 | 0.098 | 0.995 | 0.177 | trans |
| rs28929474 | Diabetic polyneuropathy | 14 | 94844947 | T/C | 0.020 | -0.002 | 0.272 | 0.993 | 0.020 | trans |
| rs7643425 | Diabetic polyneuropathy | 3 | 170737395 | G/A | 0.117 | -0.016 | 0.118 | 0.895 | 0.117 | trans |
| rs1870148 | Diabetic polyneuropathy | 10 | 82271341 | A/G | 0.768 | -0.034 | 0.090 | 0.707 | 0.232 | trans |
| rs6445607 | Diabetic polyneuropathy | 3 | 53877149 | T/G | 0.645 | -0.091 | 0.079 | 0.248 | 0.355 | cis |
| rs1260326 | Diabetic polyneuropathy | 2 | 27730940 | T/C | 0.351 | -0.100 | 0.079 | 0.208 | 0.351 | trans |
| PDCD6 |  |  |  |  |  |  |  |  |  |  |
| rs10842898 | Diabetic polyneuropathy | 12 | 9262289 | T/G | 0.409 | 0.075 | 0.077 | 0.329 | 0.409 | trans |
| rs112635299 | Diabetic polyneuropathy | 14 | 94838142 | T/G | 0.020 | -0.014 | 0.271 | 0.960 | 0.020 | trans |
| rs9654451 | Diabetic polyneuropathy | 5 | 299543 | T/C | 0.091 | -0.169 | 0.132 | 0.200 | 0.091 | cis |
| PTHLH |  |  |  |  |  |  |  |  |  |  |
| rs2731674 | Diabetic polyneuropathy | 5 | 176839890 | G/T | 0.740 | -0.002 | 0.086 | 0.979 | 0.260 | trans |
| rs10843115 | Diabetic polyneuropathy | 12 | 28307717 | T/C | 0.238 | -0.040 | 0.088 | 0.649 | 0.238 | cis |
| H6PD |  |  |  |  |  |  |  |  |  |  |
| rs149062332 | Diabetic polyneuropathy | 3 | 186341025 | T/C | 0.971 | 0.268 | 0.233 | 0.250 | 0.029 | trans |
| rs2310925 | Diabetic polyneuropathy | 1 | 9306849 | T/C | 0.662 | 0.146 | 0.080 | 0.067 | 0.338 | cis |
| rs28929474 | Diabetic polyneuropathy | 14 | 94844947 | T/C | 0.020 | -0.002 | 0.272 | 0.993 | 0.020 | trans |
| rs8178824 | Diabetic polyneuropathy | 17 | 64224775 | T/C | 0.009 | -0.140 | 0.389 | 0.718 | 0.009 | trans |
| CES1 |  |  |  |  |  |  |  |  |  |  |
| rs114082534 | Diabetic polyneuropathy | 4 | 119734184 | A/G | 0.062 | 0.304 | 0.156 | 0.051 | 0.062 | trans |
| rs72775768 | Diabetic polyneuropathy | 9 | 139324574 | T/C | 0.254 | 0.150 | 0.087 | 0.082 | 0.254 | trans |
| rs12144137 | Diabetic polyneuropathy | 1 | 224654854 | T/C | 0.027 | 0.065 | 0.229 | 0.776 | 0.027 | trans |
| rs58542926 | Diabetic polyneuropathy | 19 | 19379549 | T/C | 0.064 | 0.005 | 0.153 | 0.977 | 0.064 | trans |
| rs10845139 | Diabetic polyneuropathy | 12 | 7579654 | A/G | 0.109 | -0.050 | 0.121 | 0.679 | 0.109 | trans |
| rs112875651 | Diabetic polyneuropathy | 8 | 126506694 | A/G | 0.387 | -0.058 | 0.078 | 0.456 | 0.387 | trans |
| rs1260326 | Diabetic polyneuropathy | 2 | 27730940 | T/C | 0.351 | -0.100 | 0.079 | 0.208 | 0.351 | trans |
| rs7979473 | Diabetic polyneuropathy | 12 | 121420260 | G/A | 0.581 | -0.108 | 0.077 | 0.161 | 0.419 | trans |
| rs8736 | Diabetic polyneuropathy | 19 | 54677189 | T/C | 0.394 | -0.187 | 0.078 | 0.016 | 0.394 | trans |
| rs7640956 | Diabetic polyneuropathy | 3 | 57634135 | G/A | 0.805 | -0.200 | 0.095 | 0.035 | 0.195 | trans |
| rs7256200 | Diabetic polyneuropathy | 19 | 45415935 | T/G | 0.161 | -0.242 | 0.105 | 0.021 | 0.161 | trans |
| rs147233090 | Diabetic polyneuropathy | 15 | 44028047 | T/C | 0.005 | -0.444 | 0.516 | 0.389 | 0.005 | trans |
| HMOX1 |  |  |  |  |  |  |  |  |  |  |
| rs34632751 | Diabetic polyneuropathy | 19 | 48715196 | C/T | 0.042 | 0.230 | 0.188 | 0.223 | 0.042 | trans |
| rs11379524 | Diabetic polyneuropathy | 9 | 95882745 | AC/A | 0.653 | 0.115 | 0.080 | 0.148 | 0.347 | trans |
| rs7080536 | Diabetic polyneuropathy | 10 | 115348046 | A/G | 0.028 | 0.086 | 0.226 | 0.702 | 0.028 | trans |
| rs6786207 | Diabetic polyneuropathy | 3 | 69838315 | T/G | 0.604 | 0.079 | 0.077 | 0.310 | 0.396 | trans |
| rs113460913 | Diabetic polyneuropathy | 20 | 30121896 | TGA/T | 0.205 | 0.063 | 0.093 | 0.500 | 0.205 | trans |
| rs12149545 | Diabetic polyneuropathy | 16 | 56993161 | A/G | 0.276 | 0.054 | 0.085 | 0.523 | 0.276 | trans |
| rs261290 | Diabetic polyneuropathy | 15 | 58678720 | C/T | 0.618 | 0.050 | 0.078 | 0.524 | 0.382 | trans |
| rs117280289 | Diabetic polyneuropathy | 20 | 26232799 | T/C | 0.095 | -0.069 | 0.128 | 0.589 | 0.095 | trans |
| rs17476364 | Diabetic polyneuropathy | 10 | 71094504 | C/T | 0.059 | -0.128 | 0.159 | 0.419 | 0.059 | trans |
| rs385076 | Diabetic polyneuropathy | 2 | 32489851 | C/T | 0.645 | -0.128 | 0.079 | 0.104 | 0.355 | trans |
| rs17229943 | Diabetic polyneuropathy | 5 | 68682536 | C/A | 0.102 | -0.149 | 0.127 | 0.242 | 0.102 | trans |
| SERPINA9 |  |  |  |  |  |  |  |  |  |  |
| rs10849448 | Diabetic polyneuropathy | 12 | 6493351 | G/A | 0.755 | -0.056 | 0.089 | 0.528 | 0.246 | trans |
| rs11850199 | Diabetic polyneuropathy | 14 | 94942663 | A/C | 0.191 | -0.094 | 0.095 | 0.322 | 0.191 | cis |
| CXCL13 |  |  |  |  |  |  |  |  |  |  |
| rs77542162 | Diabetic polyneuropathy | 17 | 67081278 | A/G | 0.993 | 0.407 | 0.465 | 0.382 | 0.007 | trans |
| rs190712692 | Diabetic polyneuropathy | 19 | 45425178 | A/G | 0.032 | 0.273 | 0.210 | 0.194 | 0.032 | trans |
| rs55730499 | Diabetic polyneuropathy | 6 | 161005610 | T/C | 0.046 | 0.033 | 0.178 | 0.852 | 0.046 | trans |
| rs10849448 | Diabetic polyneuropathy | 12 | 6493351 | G/A | 0.755 | -0.056 | 0.089 | 0.528 | 0.246 | trans |
| LTBR |  |  |  |  |  |  |  |  |  |  |
| rs77924615 | Diabetic polyneuropathy | 16 | 20392332 | A/G | 0.222 | 0.042 | 0.091 | 0.643 | 0.222 | trans |
| rs966541 | Diabetic polyneuropathy | 12 | 29491528 | G/A | 0.291 | 0.030 | 0.083 | 0.715 | 0.291 | trans |
| rs27295 | Diabetic polyneuropathy | 5 | 96358687 | T/C | 0.593 | -0.002 | 0.077 | 0.983 | 0.407 | trans |
| rs28929474 | Diabetic polyneuropathy | 14 | 94844947 | T/C | 0.020 | -0.002 | 0.272 | 0.993 | 0.020 | trans |
| rs1260326 | Diabetic polyneuropathy | 2 | 27730940 | T/C | 0.351 | -0.100 | 0.079 | 0.208 | 0.351 | trans |
| rs1801689 | Diabetic polyneuropathy | 17 | 64210580 | C/A | 0.010 | -0.158 | 0.383 | 0.681 | 0.010 | trans |
| rs10849449 | Diabetic polyneuropathy | 12 | 6498662 | G/A | 0.700 | -0.166 | 0.083 | 0.046 | 0.300 | cis |
| rs61747728 | Diabetic polyneuropathy | 1 | 179526214 | T/C | 0.064 | -0.201 | 0.158 | 0.204 | 0.064 | trans |
| rs75166367 | Diabetic polyneuropathy | 2 | 162964301 | A/G | 0.071 | -0.204 | 0.149 | 0.170 | 0.071 | trans |
| CHFR |  |  |  |  |  |  |  |  |  |  |
| rs10849546 | Diabetic polyneuropathy | 12 | 7176204 | A/G | 0.177 | -0.038 | 0.099 | 0.702 | 0.177 | trans |
| PRTG |  |  |  |  |  |  |  |  |  |  |
| rs77542162 | Diabetic polyneuropathy | 17 | 67081278 | A/G | 0.993 | 0.407 | 0.465 | 0.382 | 0.007 | trans |
| rs8176746 | Diabetic polyneuropathy | 9 | 136131322 | T/G | 0.133 | 0.161 | 0.111 | 0.146 | 0.133 | trans |
| rs13107325 | Diabetic polyneuropathy | 4 | 103188709 | T/C | 0.014 | 0.106 | 0.309 | 0.731 | 0.014 | trans |
| rs704 | Diabetic polyneuropathy | 17 | 26694861 | A/G | 0.420 | 0.049 | 0.076 | 0.519 | 0.420 | trans |
| rs10851591 | Diabetic polyneuropathy | 15 | 56003705 | G/A | 0.652 | -0.005 | 0.080 | 0.953 | 0.348 | cis |
| rs6565921 | Diabetic polyneuropathy | 18 | 74557753 | G/A | 0.658 | -0.062 | 0.079 | 0.433 | 0.342 | trans |
| UMOD |  |  |  |  |  |  |  |  |  |  |
| rs1997596 | Diabetic polyneuropathy | 21 | 16578448 | T/C | 0.383 | 0.174 | 0.078 | 0.026 | 0.383 | trans |
| rs12465018 | Diabetic polyneuropathy | 2 | 9236443 | A/G | 0.154 | 0.115 | 0.105 | 0.276 | 0.154 | trans |
| rs72819488 | Diabetic polyneuropathy | 2 | 95947099 | A/G | 0.160 | 0.088 | 0.103 | 0.391 | 0.160 | trans |
| rs72835417 | Diabetic polyneuropathy | 17 | 47241642 | A/G | 0.133 | 0.088 | 0.113 | 0.433 | 0.133 | trans |
| rs10851885 | Diabetic polyneuropathy | 15 | 76304503 | G/A | 0.148 | 0.079 | 0.106 | 0.456 | 0.148 | trans |
| rs72831838 | Diabetic polyneuropathy | 2 | 114016401 | T/C | 0.175 | -0.032 | 0.100 | 0.749 | 0.175 | trans |
| rs6793835 | Diabetic polyneuropathy | 3 | 135819934 | A/G | 0.207 | -0.034 | 0.093 | 0.717 | 0.207 | trans |
| rs56012466 | Diabetic polyneuropathy | 7 | 151406788 | A/G | 0.197 | -0.062 | 0.094 | 0.509 | 0.197 | trans |
| rs2934951 | Diabetic polyneuropathy | 17 | 37833328 | G/A | 0.688 | -0.143 | 0.081 | 0.080 | 0.312 | trans |
| rs13288823 | Diabetic polyneuropathy | 9 | 6386584 | T/C | 0.023 | -0.158 | 0.252 | 0.532 | 0.023 | trans |
| rs4846828 | Diabetic polyneuropathy | 1 | 217429631 | A/G | 0.641 | -0.181 | 0.079 | 0.021 | 0.359 | trans |
| MTHFD2 |  |  |  |  |  |  |  |  |  |  |
| rs10852622 | Diabetic polyneuropathy | 16 | 88556880 | A/G | 0.734 | 0.105 | 0.086 | 0.224 | 0.266 | trans |
| ACOX1 |  |  |  |  |  |  |  |  |  |  |
| rs10852766 | Diabetic polyneuropathy | 17 | 73951864 | C/T | 0.755 | -0.079 | 0.088 | 0.370 | 0.245 | cis |
| QSOX2 |  |  |  |  |  |  |  |  |  |  |
| rs3184504 | Diabetic polyneuropathy | 12 | 111884608 | C/T | 0.591 | -0.051 | 0.077 | 0.504 | 0.409 | trans |
| OLFM1 |  |  |  |  |  |  |  |  |  |  |
| rs507666 | Diabetic polyneuropathy | 9 | 136149399 | A/G | 0.201 | 0.019 | 0.094 | 0.840 | 0.201 | trans |
| GP2 |  |  |  |  |  |  |  |  |  |  |
| rs10858917 | Diabetic polyneuropathy | 12 | 90088790 | A/G | 0.895 | 0.204 | 0.122 | 0.093 | 0.105 | trans |
| rs6571015 | Diabetic polyneuropathy | 6 | 96534478 | A/G | 0.836 | 0.114 | 0.101 | 0.260 | 0.164 | trans |
| rs6459924 | Diabetic polyneuropathy | 7 | 158908074 | C/T | 0.314 | 0.066 | 0.082 | 0.421 | 0.314 | trans |
| rs492602 | Diabetic polyneuropathy | 19 | 49206417 | A/G | 0.625 | 0.045 | 0.078 | 0.566 | 0.375 | trans |
| rs708686 | Diabetic polyneuropathy | 19 | 5840619 | T/C | 0.334 | -0.105 | 0.081 | 0.194 | 0.334 | trans |
| HTN3 |  |  |  |  |  |  |  |  |  |  |
| rs13357659 | Diabetic polyneuropathy | 5 | 44432885 | G/A | 0.364 | 0.144 | 0.078 | 0.066 | 0.364 | trans |
| rs61762319 | Diabetic polyneuropathy | 3 | 154801978 | G/A | 0.021 | 0.113 | 0.266 | 0.670 | 0.021 | trans |
| rs150022487 | Diabetic polyneuropathy | 4 | 128821305 | A/G | 0.044 | -0.030 | 0.180 | 0.868 | 0.044 | trans |
| rs681343 | Diabetic polyneuropathy | 19 | 49206462 | T/C | 0.375 | -0.045 | 0.078 | 0.568 | 0.375 | trans |
| rs7398041 | Diabetic polyneuropathy | 12 | 11067524 | A/G | 0.276 | -0.045 | 0.084 | 0.591 | 0.276 | trans |
| rs10863962 | Diabetic polyneuropathy | 1 | 207129266 | T/C | 0.191 | -0.118 | 0.096 | 0.222 | 0.191 | trans |
| MAP2K4 |  |  |  |  |  |  |  |  |  |  |
| rs186268843 | Diabetic polyneuropathy | 3 | 186379356 | A/G | 0.176 | -0.026 | 0.100 | 0.796 | 0.176 | trans |
| NTF3 |  |  |  |  |  |  |  |  |  |  |
| rs73039984 | Diabetic polyneuropathy | 12 | 5549456 | T/C | 0.076 | -0.002 | 0.143 | 0.991 | 0.076 | cis |
| rs117126605 | Diabetic polyneuropathy | 15 | 88792227 | C/T | 0.007 | -0.426 | 0.448 | 0.342 | 0.007 | trans |
| IGFBP6 |  |  |  |  |  |  |  |  |  |  |
| rs10876406 | Diabetic polyneuropathy | 12 | 53501437 | C/T | 0.154 | -0.035 | 0.105 | 0.740 | 0.154 | cis |
| MAFG |  |  |  |  |  |  |  |  |  |  |
| rs10876550 | Diabetic polyneuropathy | 12 | 54712308 | A/G | 0.677 | -0.046 | 0.081 | 0.569 | 0.323 | trans |
| rs11594179 | Diabetic polyneuropathy | 10 | 104392580 | T/C | 0.176 | -0.060 | 0.099 | 0.544 | 0.176 | trans |
| SPG20 |  |  |  |  |  |  |  |  |  |  |
| rs10876550 | Diabetic polyneuropathy | 12 | 54712308 | A/G | 0.677 | -0.046 | 0.081 | 0.569 | 0.323 | trans |
| APRT |  |  |  |  |  |  |  |  |  |  |
| rs1354034 | Diabetic polyneuropathy | 3 | 56849749 | C/T | 0.707 | 0.052 | 0.083 | 0.532 | 0.293 | trans |
| rs704 | Diabetic polyneuropathy | 17 | 26694861 | A/G | 0.420 | 0.049 | 0.076 | 0.519 | 0.420 | trans |
| rs10876550 | Diabetic polyneuropathy | 12 | 54712308 | A/G | 0.677 | -0.046 | 0.081 | 0.569 | 0.323 | trans |
| IL12A_IL12B |  |  |  |  |  |  |  |  |  |  |
| rs76428106 | Diabetic polyneuropathy | 13 | 28604007 | C/T | 0.012 | 0.322 | 0.347 | 0.353 | 0.012 | trans |
| rs1987102 | Diabetic polyneuropathy | 15 | 80265888 | C/T | 0.218 | 0.080 | 0.092 | 0.385 | 0.218 | trans |
| rs10876864 | Diabetic polyneuropathy | 12 | 56401085 | A/G | 0.580 | 0.044 | 0.076 | 0.564 | 0.420 | trans |
| rs11130215 | Diabetic polyneuropathy | 3 | 5026008 | G/A | 0.271 | -0.027 | 0.085 | 0.752 | 0.271 | trans |
| rs4641365 | Diabetic polyneuropathy | 10 | 23666262 | T/C | 0.185 | -0.028 | 0.098 | 0.780 | 0.185 | trans |
| rs9815073 | Diabetic polyneuropathy | 3 | 188115682 | A/C | 0.328 | -0.036 | 0.081 | 0.654 | 0.328 | trans |
| rs3184504 | Diabetic polyneuropathy | 12 | 111884608 | C/T | 0.591 | -0.051 | 0.077 | 0.504 | 0.409 | trans |
| rs561877 | Diabetic polyneuropathy | 11 | 96019254 | A/G | 0.261 | -0.052 | 0.086 | 0.549 | 0.261 | trans |
| rs12471768 | Diabetic polyneuropathy | 2 | 64928603 | C/T | 0.770 | -0.065 | 0.089 | 0.466 | 0.230 | trans |
| rs4244437 | Diabetic polyneuropathy | 5 | 158773117 | A/G | 0.750 | -0.117 | 0.086 | 0.177 | 0.250 | cis |
| rs13025330 | Diabetic polyneuropathy | 2 | 111851212 | T/C | 0.208 | -0.120 | 0.094 | 0.203 | 0.208 | trans |
| rs72852162 | Diabetic polyneuropathy | 2 | 145486323 | C/A | 0.129 | -0.222 | 0.113 | 0.050 | 0.129 | trans |
| IL12B |  |  |  |  |  |  |  |  |  |  |
| rs76428106 | Diabetic polyneuropathy | 13 | 28604007 | C/T | 0.012 | 0.322 | 0.347 | 0.353 | 0.012 | trans |
| rs73068668 | Diabetic polyneuropathy | 19 | 55763262 | A/G | 0.083 | 0.203 | 0.138 | 0.142 | 0.083 | trans |
| rs1987102 | Diabetic polyneuropathy | 15 | 80265888 | C/T | 0.218 | 0.080 | 0.092 | 0.385 | 0.218 | trans |
| rs4427476 | Diabetic polyneuropathy | 10 | 23670802 | T/G | 0.153 | 0.059 | 0.106 | 0.580 | 0.153 | trans |
| rs10876864 | Diabetic polyneuropathy | 12 | 56401085 | A/G | 0.580 | 0.044 | 0.076 | 0.564 | 0.420 | trans |
| rs58394161 | Diabetic polyneuropathy | 1 | 92939959 | C/T | 0.135 | 0.032 | 0.111 | 0.770 | 0.135 | trans |
| rs2422287 | Diabetic polyneuropathy | 2 | 64944765 | T/C | 0.745 | 0.015 | 0.086 | 0.866 | 0.255 | trans |
| rs11130215 | Diabetic polyneuropathy | 3 | 5026008 | G/A | 0.271 | -0.027 | 0.085 | 0.752 | 0.271 | trans |
| rs9815073 | Diabetic polyneuropathy | 3 | 188115682 | A/C | 0.328 | -0.036 | 0.081 | 0.654 | 0.328 | trans |
| rs3184504 | Diabetic polyneuropathy | 12 | 111884608 | C/T | 0.591 | -0.051 | 0.077 | 0.504 | 0.409 | trans |
| rs4244437 | Diabetic polyneuropathy | 5 | 158773117 | A/G | 0.750 | -0.117 | 0.086 | 0.177 | 0.250 | cis |
| rs13025330 | Diabetic polyneuropathy | 2 | 111851212 | T/C | 0.208 | -0.120 | 0.094 | 0.203 | 0.208 | trans |
| rs72852162 | Diabetic polyneuropathy | 2 | 145486323 | C/A | 0.129 | -0.222 | 0.113 | 0.050 | 0.129 | trans |
| ADH4 |  |  |  |  |  |  |  |  |  |  |
| rs11604580 | Diabetic polyneuropathy | 11 | 93852880 | A/G | 0.101 | 0.102 | 0.124 | 0.414 | 0.101 | trans |
| rs3747207 | Diabetic polyneuropathy | 22 | 44324855 | A/G | 0.226 | -0.010 | 0.090 | 0.915 | 0.226 | trans |
| rs10883451 | Diabetic polyneuropathy | 10 | 101924418 | C/T | 0.389 | -0.058 | 0.077 | 0.456 | 0.389 | trans |
| rs112875651 | Diabetic polyneuropathy | 8 | 126506694 | A/G | 0.387 | -0.058 | 0.078 | 0.456 | 0.387 | trans |
| GSTA3 |  |  |  |  |  |  |  |  |  |  |
| rs28929474 | Diabetic polyneuropathy | 14 | 94844947 | T/C | 0.020 | -0.002 | 0.272 | 0.993 | 0.020 | trans |
| rs10883451 | Diabetic polyneuropathy | 10 | 101924418 | C/T | 0.389 | -0.058 | 0.077 | 0.456 | 0.389 | trans |
| HAO1 |  |  |  |  |  |  |  |  |  |  |
| rs12373325 | Diabetic polyneuropathy | 18 | 56087648 | C/T | 0.767 | 0.015 | 0.090 | 0.866 | 0.233 | trans |
| rs4835265 | Diabetic polyneuropathy | 4 | 146821410 | A/C | 0.172 | -0.047 | 0.101 | 0.641 | 0.172 | trans |
| rs10883451 | Diabetic polyneuropathy | 10 | 101924418 | C/T | 0.389 | -0.058 | 0.077 | 0.456 | 0.389 | trans |
| rs112875651 | Diabetic polyneuropathy | 8 | 126506694 | A/G | 0.387 | -0.058 | 0.078 | 0.456 | 0.387 | trans |
| rs1497406 | Diabetic polyneuropathy | 1 | 16505320 | G/A | 0.682 | -0.071 | 0.081 | 0.379 | 0.318 | trans |
| KRT18 |  |  |  |  |  |  |  |  |  |  |
| rs28929474 | Diabetic polyneuropathy | 14 | 94844947 | T/C | 0.020 | -0.002 | 0.272 | 0.993 | 0.020 | trans |
| rs3747207 | Diabetic polyneuropathy | 22 | 44324855 | A/G | 0.226 | -0.010 | 0.090 | 0.915 | 0.226 | trans |
| rs7678352 | Diabetic polyneuropathy | 4 | 146814317 | T/C | 0.175 | -0.057 | 0.099 | 0.566 | 0.175 | trans |
| rs10883451 | Diabetic polyneuropathy | 10 | 101924418 | C/T | 0.389 | -0.058 | 0.077 | 0.456 | 0.389 | trans |
| rs35696875 | Diabetic polyneuropathy | 10 | 70982136 | TCA/T | 0.751 | -0.058 | 0.087 | 0.506 | 0.249 | trans |
| rs79287178 | Diabetic polyneuropathy | 3 | 172294500 | A/G | 0.042 | -0.058 | 0.192 | 0.763 | 0.042 | trans |
| rs11601507 | Diabetic polyneuropathy | 11 | 5701074 | A/C | 0.079 | -0.059 | 0.141 | 0.674 | 0.079 | trans |
| CA5A |  |  |  |  |  |  |  |  |  |  |
| rs4835265 | Diabetic polyneuropathy | 4 | 146821410 | A/C | 0.172 | -0.047 | 0.101 | 0.641 | 0.172 | trans |
| rs10883451 | Diabetic polyneuropathy | 10 | 101924418 | C/T | 0.389 | -0.058 | 0.077 | 0.456 | 0.389 | trans |
| rs55870502 | Diabetic polyneuropathy | 16 | 87927222 | C/T | 0.148 | -0.138 | 0.106 | 0.192 | 0.148 | cis |
| AFP |  |  |  |  |  |  |  |  |  |  |
| rs59950280 | Diabetic polyneuropathy | 4 | 3452345 | A/G | 0.245 | 0.059 | 0.088 | 0.500 | 0.245 | trans |
| rs6116934 | Diabetic polyneuropathy | 20 | 5731803 | C/T | 0.125 | 0.042 | 0.115 | 0.712 | 0.125 | trans |
| rs6007594 | Diabetic polyneuropathy | 22 | 45728370 | A/G | 0.239 | 0.013 | 0.088 | 0.884 | 0.239 | trans |
| rs62576492 | Diabetic polyneuropathy | 9 | 135218708 | G/A | 0.127 | -0.015 | 0.115 | 0.898 | 0.127 | trans |
| rs12506899 | Diabetic polyneuropathy | 4 | 74319283 | G/T | 0.420 | -0.050 | 0.077 | 0.520 | 0.420 | cis |
| rs2548458 | Diabetic polyneuropathy | 19 | 49209325 | T/C | 0.410 | -0.065 | 0.077 | 0.395 | 0.410 | trans |
| rs35103294 | Diabetic polyneuropathy | 17 | 73810937 | T/C | 0.692 | -0.077 | 0.082 | 0.347 | 0.309 | trans |
| rs7310409 | Diabetic polyneuropathy | 12 | 121424861 | G/A | 0.584 | -0.090 | 0.077 | 0.244 | 0.416 | trans |
| rs62280667 | Diabetic polyneuropathy | 3 | 101084604 | C/T | 0.382 | -0.137 | 0.078 | 0.078 | 0.382 | trans |
| rs1801689 | Diabetic polyneuropathy | 17 | 64210580 | C/A | 0.010 | -0.158 | 0.383 | 0.681 | 0.010 | trans |
| rs11900031 | Diabetic polyneuropathy | 2 | 17930874 | G/A | 0.086 | -0.179 | 0.133 | 0.179 | 0.086 | trans |
| rs4149909 | Diabetic polyneuropathy | 1 | 242023898 | G/A | 0.032 | -0.612 | 0.223 | 0.006 | 0.032 | trans |
| CCL17 |  |  |  |  |  |  |  |  |  |  |
| rs142289859 | Diabetic polyneuropathy | 3 | 16954941 | T/C | 0.961 | 0.087 | 0.195 | 0.656 | 0.039 | trans |
| rs10886430 | Diabetic polyneuropathy | 10 | 121010256 | A/G | 0.905 | 0.084 | 0.130 | 0.517 | 0.095 | trans |
| rs2228467 | Diabetic polyneuropathy | 3 | 42906116 | C/T | 0.076 | -0.071 | 0.143 | 0.617 | 0.076 | trans |
| rs16956811 | Diabetic polyneuropathy | 16 | 57444002 | T/G | 0.950 | -0.177 | 0.172 | 0.305 | 0.050 | cis |
| GRK5 |  |  |  |  |  |  |  |  |  |  |
| rs10886430 | Diabetic polyneuropathy | 10 | 121010256 | A/G | 0.905 | 0.084 | 0.130 | 0.517 | 0.095 | trans |
| TMPRSS5 |  |  |  |  |  |  |  |  |  |  |
| rs17326497 | Diabetic polyneuropathy | 9 | 33111686 | A/G | 0.025 | 0.121 | 0.238 | 0.612 | 0.025 | trans |
| rs60028714 | Diabetic polyneuropathy | 4 | 86393300 | T/C | 0.686 | 0.085 | 0.082 | 0.299 | 0.315 | trans |
| rs67670470 | Diabetic polyneuropathy | 3 | 152330739 | A/C | 0.396 | 0.084 | 0.077 | 0.278 | 0.396 | trans |
| rs12024555 | Diabetic polyneuropathy | 1 | 222147845 | A/G | 0.250 | 0.058 | 0.088 | 0.505 | 0.250 | trans |
| rs28404455 | Diabetic polyneuropathy | 13 | 50815358 | C/T | 0.051 | 0.035 | 0.169 | 0.836 | 0.051 | trans |
| rs504549 | Diabetic polyneuropathy | 19 | 37437201 | T/C | 0.346 | 0.026 | 0.079 | 0.739 | 0.346 | trans |
| rs1371687 | Diabetic polyneuropathy | 3 | 98604990 | G/A | 0.365 | 0.021 | 0.079 | 0.785 | 0.365 | trans |
| rs12912843 | Diabetic polyneuropathy | 15 | 50999333 | G/T | 0.122 | 0.010 | 0.115 | 0.929 | 0.122 | trans |
| rs56278466 | Diabetic polyneuropathy | 10 | 17875857 | G/T | 0.581 | -0.003 | 0.077 | 0.970 | 0.419 | trans |
| rs2836683 | Diabetic polyneuropathy | 21 | 40197438 | C/T | 0.361 | -0.014 | 0.078 | 0.857 | 0.361 | trans |
| rs35383942 | Diabetic polyneuropathy | 1 | 201437832 | T/C | 0.116 | -0.046 | 0.120 | 0.699 | 0.116 | trans |
| rs7114195 | Diabetic polyneuropathy | 11 | 113561421 | C/A | 0.627 | -0.059 | 0.078 | 0.446 | 0.373 | cis |
| FAS |  |  |  |  |  |  |  |  |  |  |
| rs7911226 | Diabetic polyneuropathy | 10 | 90768965 | A/G | 0.636 | 0.021 | 0.079 | 0.790 | 0.364 | cis |
| rs10887883 | Diabetic polyneuropathy | 10 | 90782973 | A/G | 0.418 | 0.015 | 0.077 | 0.846 | 0.418 | cis |
| TRAPPC4 |  |  |  |  |  |  |  |  |  |  |
| rs1354034 | Diabetic polyneuropathy | 3 | 56849749 | C/T | 0.707 | 0.052 | 0.083 | 0.532 | 0.293 | trans |
| rs12491503 | Diabetic polyneuropathy | 3 | 165706855 | A/G | 0.284 | -0.048 | 0.083 | 0.566 | 0.284 | trans |
| IQCF1 |  |  |  |  |  |  |  |  |  |  |
| rs704 | Diabetic polyneuropathy | 17 | 26694861 | A/G | 0.420 | 0.049 | 0.076 | 0.519 | 0.420 | trans |
| SUN3 |  |  |  |  |  |  |  |  |  |  |
| rs10891700 | Diabetic polyneuropathy | 11 | 114437886 | T/C | 0.099 | 0.072 | 0.125 | 0.565 | 0.099 | trans |
| PLXNC1 |  |  |  |  |  |  |  |  |  |  |
| rs115651556 | Diabetic polyneuropathy | 12 | 94613898 | A/G | 0.032 | 0.109 | 0.216 | 0.613 | 0.032 | cis |
| rs10893501 | Diabetic polyneuropathy | 11 | 126251033 | G/A | 0.187 | -0.030 | 0.098 | 0.761 | 0.187 | trans |
| CAPN1_CAPNS1 |  |  |  |  |  |  |  |  |  |  |
| rs10895987 | Diabetic polyneuropathy | 11 | 64904908 | T/C | 0.225 | -0.115 | 0.090 | 0.205 | 0.225 | cis |
| PNPLA2 |  |  |  |  |  |  |  |  |  |  |
| rs1354034 | Diabetic polyneuropathy | 3 | 56849749 | C/T | 0.707 | 0.052 | 0.083 | 0.532 | 0.293 | trans |
| rs704 | Diabetic polyneuropathy | 17 | 26694861 | A/G | 0.420 | 0.049 | 0.076 | 0.519 | 0.420 | trans |
| SHISA3 |  |  |  |  |  |  |  |  |  |  |
| rs10896045 | Diabetic polyneuropathy | 11 | 65555524 | G/A | 0.728 | -0.045 | 0.085 | 0.593 | 0.272 | trans |
| QPCT |  |  |  |  |  |  |  |  |  |  |
| rs3803800 | Diabetic polyneuropathy | 17 | 7462969 | G/A | 0.755 | 0.012 | 0.088 | 0.894 | 0.245 | trans |
| rs56278466 | Diabetic polyneuropathy | 10 | 17875857 | G/T | 0.581 | -0.003 | 0.077 | 0.970 | 0.419 | trans |
| rs111632177 | Diabetic polyneuropathy | 7 | 150931805 | A/G | 0.118 | -0.004 | 0.116 | 0.973 | 0.118 | trans |
| rs10896045 | Diabetic polyneuropathy | 11 | 65555524 | G/A | 0.728 | -0.045 | 0.085 | 0.593 | 0.272 | trans |
| rs4670696 | Diabetic polyneuropathy | 2 | 37599963 | C/A | 0.054 | -0.050 | 0.167 | 0.765 | 0.054 | cis |
| rs34562254 | Diabetic polyneuropathy | 17 | 16842991 | A/G | 0.100 | -0.145 | 0.126 | 0.247 | 0.100 | trans |
| rs188468174 | Diabetic polyneuropathy | 1 | 25291697 | T/C | 0.004 | -0.166 | 0.540 | 0.758 | 0.004 | trans |
| rs61747728 | Diabetic polyneuropathy | 1 | 179526214 | T/C | 0.064 | -0.201 | 0.158 | 0.204 | 0.064 | trans |
| STK4 |  |  |  |  |  |  |  |  |  |  |
| rs1354034 | Diabetic polyneuropathy | 3 | 56849749 | C/T | 0.707 | 0.052 | 0.083 | 0.532 | 0.293 | trans |
| rs10900809 | Diabetic polyneuropathy | 5 | 131826322 | A/G | 0.260 | -0.139 | 0.086 | 0.107 | 0.260 | trans |
| SKAP2 |  |  |  |  |  |  |  |  |  |  |
| rs1354034 | Diabetic polyneuropathy | 3 | 56849749 | C/T | 0.707 | 0.052 | 0.083 | 0.532 | 0.293 | trans |
| rs10900809 | Diabetic polyneuropathy | 5 | 131826322 | A/G | 0.260 | -0.139 | 0.086 | 0.107 | 0.260 | trans |
| CLEC1A |  |  |  |  |  |  |  |  |  |  |
| rs7669607 | Diabetic polyneuropathy | 4 | 9997801 | C/T | 0.805 | 0.189 | 0.096 | 0.049 | 0.195 | trans |
| rs77924615 | Diabetic polyneuropathy | 16 | 20392332 | A/G | 0.222 | 0.042 | 0.091 | 0.643 | 0.222 | trans |
| rs7961483 | Diabetic polyneuropathy | 12 | 10230460 | T/C | 0.635 | -0.061 | 0.078 | 0.434 | 0.366 | cis |
| rs61747728 | Diabetic polyneuropathy | 1 | 179526214 | T/C | 0.064 | -0.201 | 0.158 | 0.204 | 0.064 | trans |
| ENPP2 |  |  |  |  |  |  |  |  |  |  |
| rs77542162 | Diabetic polyneuropathy | 17 | 67081278 | A/G | 0.993 | 0.407 | 0.465 | 0.382 | 0.007 | trans |
| rs34707604 | Diabetic polyneuropathy | 4 | 69491456 | C/T | 0.263 | 0.028 | 0.088 | 0.746 | 0.263 | trans |
| rs13267597 | Diabetic polyneuropathy | 8 | 120654225 | G/T | 0.336 | -0.015 | 0.080 | 0.850 | 0.336 | cis |
| rs7970695 | Diabetic polyneuropathy | 12 | 121423376 | A/G | 0.584 | -0.089 | 0.077 | 0.246 | 0.416 | trans |
| rs4921913 | Diabetic polyneuropathy | 8 | 18272377 | T/C | 0.750 | -0.113 | 0.087 | 0.193 | 0.251 | trans |
| FLRT2 |  |  |  |  |  |  |  |  |  |  |
| rs77542162 | Diabetic polyneuropathy | 17 | 67081278 | A/G | 0.993 | 0.407 | 0.465 | 0.382 | 0.007 | trans |
| rs17646457 | Diabetic polyneuropathy | 14 | 86089315 | A/G | 0.179 | 0.122 | 0.099 | 0.220 | 0.179 | cis |
| rs4841132 | Diabetic polyneuropathy | 8 | 9183596 | G/A | 0.857 | 0.118 | 0.116 | 0.309 | 0.143 | trans |
| rs6135225 | Diabetic polyneuropathy | 20 | 14678135 | T/G | 0.282 | 0.004 | 0.084 | 0.960 | 0.282 | trans |
| rs3747207 | Diabetic polyneuropathy | 22 | 44324855 | A/G | 0.226 | -0.010 | 0.090 | 0.915 | 0.226 | trans |
| rs3748770 | Diabetic polyneuropathy | 1 | 82265905 | A/G | 0.258 | -0.058 | 0.087 | 0.507 | 0.258 | trans |
| rs1260326 | Diabetic polyneuropathy | 2 | 27730940 | T/C | 0.351 | -0.100 | 0.079 | 0.208 | 0.351 | trans |
| rs6863407 | Diabetic polyneuropathy | 5 | 166831694 | T/C | 0.735 | -0.140 | 0.085 | 0.102 | 0.265 | trans |
| IL27RA |  |  |  |  |  |  |  |  |  |  |
| rs3184504 | Diabetic polyneuropathy | 12 | 111884608 | C/T | 0.591 | -0.051 | 0.077 | 0.504 | 0.409 | trans |
| IFNGR2 |  |  |  |  |  |  |  |  |  |  |
| rs12740374 | Diabetic polyneuropathy | 1 | 109817590 | T/G | 0.215 | 0.109 | 0.092 | 0.235 | 0.215 | trans |
| rs12149545 | Diabetic polyneuropathy | 16 | 56993161 | A/G | 0.276 | 0.054 | 0.085 | 0.523 | 0.276 | trans |
| rs492602 | Diabetic polyneuropathy | 19 | 49206417 | A/G | 0.625 | 0.045 | 0.078 | 0.566 | 0.375 | trans |
| rs9808753 | Diabetic polyneuropathy | 21 | 34787312 | G/A | 0.185 | 0.029 | 0.098 | 0.766 | 0.185 | cis |
| rs56278466 | Diabetic polyneuropathy | 10 | 17875857 | G/T | 0.581 | -0.003 | 0.077 | 0.970 | 0.419 | trans |
| rs72802342 | Diabetic polyneuropathy | 16 | 75234872 | A/C | 0.086 | -0.032 | 0.135 | 0.814 | 0.086 | trans |
| rs174564 | Diabetic polyneuropathy | 11 | 61588305 | G/A | 0.415 | -0.118 | 0.077 | 0.124 | 0.415 | trans |
| CA4 |  |  |  |  |  |  |  |  |  |  |
| rs61729512 | Diabetic polyneuropathy | 12 | 7637769 | A/G | 0.152 | 0.101 | 0.107 | 0.348 | 0.152 | trans |
| rs4760 | Diabetic polyneuropathy | 19 | 44153100 | G/A | 0.168 | 0.087 | 0.103 | 0.396 | 0.168 | trans |
| rs115007843 | Diabetic polyneuropathy | 4 | 83575212 | C/A | 0.232 | 0.085 | 0.090 | 0.346 | 0.232 | trans |
| rs78058190 | Diabetic polyneuropathy | 2 | 219699999 | A/G | 0.082 | 0.068 | 0.140 | 0.628 | 0.082 | trans |
| rs10906857 | Diabetic polyneuropathy | 10 | 15239498 | G/A | 0.420 | -0.053 | 0.077 | 0.490 | 0.420 | trans |
| FST |  |  |  |  |  |  |  |  |  |  |
| rs31226 | Diabetic polyneuropathy | 5 | 53327571 | T/C | 0.396 | -0.009 | 0.077 | 0.909 | 0.396 | cis |
| rs1260326 | Diabetic polyneuropathy | 2 | 27730940 | T/C | 0.351 | -0.100 | 0.079 | 0.208 | 0.351 | trans |
| rs150844304 | Diabetic polyneuropathy | 15 | 43726625 | C/A | 0.005 | -0.223 | 0.528 | 0.673 | 0.005 | trans |
| rs7974833 | Diabetic polyneuropathy | 12 | 57791833 | T/C | 0.769 | -0.245 | 0.089 | 0.006 | 0.231 | trans |
| FCGR3B |  |  |  |  |  |  |  |  |  |  |
| rs10919544 | Diabetic polyneuropathy | 1 | 161508763 | C/T | 0.267 | 0.127 | 0.086 | 0.141 | 0.267 | cis |
| PAWR |  |  |  |  |  |  |  |  |  |  |
| rs73349601 | Diabetic polyneuropathy | 12 | 79368410 | T/G | 0.260 | 0.103 | 0.086 | 0.232 | 0.260 | trans |
| rs10919615 | Diabetic polyneuropathy | 1 | 198974904 | T/C | 0.738 | 0.086 | 0.086 | 0.318 | 0.262 | trans |
| rs114694170 | Diabetic polyneuropathy | 5 | 88180196 | C/T | 0.056 | 0.015 | 0.163 | 0.926 | 0.056 | trans |
| OLFM3 |  |  |  |  |  |  |  |  |  |  |
| rs10919615 | Diabetic polyneuropathy | 1 | 198974904 | T/C | 0.738 | 0.086 | 0.086 | 0.318 | 0.262 | trans |
| rs704 | Diabetic polyneuropathy | 17 | 26694861 | A/G | 0.420 | 0.049 | 0.076 | 0.519 | 0.420 | trans |
| rs1938364 | Diabetic polyneuropathy | 1 | 102667388 | A/G | 0.138 | 0.038 | 0.108 | 0.724 | 0.138 | cis |
| KERA |  |  |  |  |  |  |  |  |  |  |
| rs2686395 | Diabetic polyneuropathy | 3 | 165485019 | T/C | 0.246 | -0.049 | 0.087 | 0.576 | 0.246 | trans |
| rs186033146 | Diabetic polyneuropathy | 12 | 104771636 | T/C | 0.009 | -0.365 | 0.392 | 0.352 | 0.009 | trans |
| ANGPTL4 |  |  |  |  |  |  |  |  |  |  |
| rs704 | Diabetic polyneuropathy | 17 | 26694861 | A/G | 0.420 | 0.049 | 0.076 | 0.519 | 0.420 | trans |
| rs6542680 | Diabetic polyneuropathy | 2 | 3640142 | C/T | 0.351 | -0.070 | 0.081 | 0.384 | 0.351 | trans |
| HLA-G |  |  |  |  |  |  |  |  |  |  |
| rs78357146 | Diabetic polyneuropathy | 17 | 64305051 | A/G | 0.991 | 0.135 | 0.393 | 0.731 | 0.009 | trans |
| rs4838254 | Diabetic polyneuropathy | 9 | 128008537 | A/G | 0.264 | 0.043 | 0.085 | 0.611 | 0.264 | trans |
| rs28929474 | Diabetic polyneuropathy | 14 | 94844947 | T/C | 0.020 | -0.002 | 0.272 | 0.993 | 0.020 | trans |
| KIF16B |  |  |  |  |  |  |  |  |  |  |
| rs1354034 | Diabetic polyneuropathy | 3 | 56849749 | C/T | 0.707 | 0.052 | 0.083 | 0.532 | 0.293 | trans |
| EEA1 |  |  |  |  |  |  |  |  |  |  |
| rs62295996 | Diabetic polyneuropathy | 3 | 165482064 | A/G | 0.174 | -0.015 | 0.099 | 0.878 | 0.174 | trans |
| RGS3 |  |  |  |  |  |  |  |  |  |  |
| rs17713088 | Diabetic polyneuropathy | 3 | 165488604 | T/G | 0.174 | -0.015 | 0.099 | 0.878 | 0.174 | trans |
| RELL2 |  |  |  |  |  |  |  |  |  |  |
| rs2073453 | Diabetic polyneuropathy | 22 | 22516998 | T/C | 0.414 | -0.046 | 0.077 | 0.552 | 0.414 | trans |
| TBX22 |  |  |  |  |  |  |  |  |  |  |
| rs1355538 | Diabetic polyneuropathy | 3 | 165505177 | A/G | 0.326 | -0.043 | 0.080 | 0.595 | 0.326 | trans |
| BACH2 |  |  |  |  |  |  |  |  |  |  |
| rs2686395 | Diabetic polyneuropathy | 3 | 165485019 | T/C | 0.246 | -0.049 | 0.087 | 0.576 | 0.246 | trans |
| rs3917538 | Diabetic polyneuropathy | 7 | 94937893 | A/G | 0.202 | -0.140 | 0.095 | 0.140 | 0.202 | trans |
| TFR2 |  |  |  |  |  |  |  |  |  |  |
| rs1355538 | Diabetic polyneuropathy | 3 | 165505177 | A/G | 0.326 | -0.043 | 0.080 | 0.595 | 0.326 | trans |
| MRAP |  |  |  |  |  |  |  |  |  |  |
| rs6542680 | Diabetic polyneuropathy | 2 | 3640142 | C/T | 0.351 | -0.070 | 0.081 | 0.384 | 0.351 | trans |
| UBE2D2 |  |  |  |  |  |  |  |  |  |  |
| rs3917534 | Diabetic polyneuropathy | 7 | 94939797 | T/C | 0.735 | 0.099 | 0.086 | 0.251 | 0.265 | trans |
| TERF1 |  |  |  |  |  |  |  |  |  |  |
| rs1354034 | Diabetic polyneuropathy | 3 | 56849749 | C/T | 0.707 | 0.052 | 0.083 | 0.532 | 0.293 | trans |
| rs12491503 | Diabetic polyneuropathy | 3 | 165706855 | A/G | 0.284 | -0.048 | 0.083 | 0.566 | 0.284 | trans |
| MPZL1 |  |  |  |  |  |  |  |  |  |  |
| rs77303550 | Diabetic polyneuropathy | 16 | 72079657 | T/C | 0.191 | 0.130 | 0.096 | 0.172 | 0.191 | trans |
| ARL5A |  |  |  |  |  |  |  |  |  |  |
| rs1706435 | Diabetic polyneuropathy | 3 | 165461476 | G/A | 0.310 | -0.079 | 0.082 | 0.331 | 0.310 | trans |
| VDR |  |  |  |  |  |  |  |  |  |  |
| rs2686395 | Diabetic polyneuropathy | 3 | 165485019 | T/C | 0.246 | -0.049 | 0.087 | 0.576 | 0.246 | trans |
| LETMD1 |  |  |  |  |  |  |  |  |  |  |
| rs17713088 | Diabetic polyneuropathy | 3 | 165488604 | T/G | 0.174 | -0.015 | 0.099 | 0.878 | 0.174 | trans |
| CELA2A |  |  |  |  |  |  |  |  |  |  |
| rs533406 | Diabetic polyneuropathy | 16 | 88974860 | G/A | 0.269 | 0.110 | 0.085 | 0.197 | 0.269 | trans |
| rs78880003 | Diabetic polyneuropathy | 1 | 15740930 | T/C | 0.031 | 0.027 | 0.218 | 0.903 | 0.031 | cis |
| rs77643237 | Diabetic polyneuropathy | 9 | 135889210 | A/G | 0.067 | 0.023 | 0.153 | 0.879 | 0.067 | trans |
| rs28365941 | Diabetic polyneuropathy | 16 | 333138 | C/T | 0.128 | -0.003 | 0.114 | 0.977 | 0.128 | trans |
| rs17032925 | Diabetic polyneuropathy | 2 | 67266483 | C/T | 0.079 | -0.074 | 0.141 | 0.598 | 0.079 | trans |
| rs75593565 | Diabetic polyneuropathy | 12 | 32115266 | G/A | 0.032 | -0.139 | 0.213 | 0.515 | 0.032 | trans |
| RGS5 |  |  |  |  |  |  |  |  |  |  |
| rs11720167 | Diabetic polyneuropathy | 3 | 165486145 | G/T | 0.174 | -0.015 | 0.099 | 0.877 | 0.174 | trans |
| rs6542680 | Diabetic polyneuropathy | 2 | 3640142 | C/T | 0.351 | -0.070 | 0.081 | 0.384 | 0.351 | trans |
| AS3MT |  |  |  |  |  |  |  |  |  |  |
| rs2686395 | Diabetic polyneuropathy | 3 | 165485019 | T/C | 0.246 | -0.049 | 0.087 | 0.576 | 0.246 | trans |
| TFIP11 |  |  |  |  |  |  |  |  |  |  |
| rs704 | Diabetic polyneuropathy | 17 | 26694861 | A/G | 0.420 | 0.049 | 0.076 | 0.519 | 0.420 | trans |
| RBBP6 |  |  |  |  |  |  |  |  |  |  |
| rs1355538 | Diabetic polyneuropathy | 3 | 165505177 | A/G | 0.326 | -0.043 | 0.080 | 0.595 | 0.326 | trans |
| AURKA |  |  |  |  |  |  |  |  |  |  |
| rs1707652 | Diabetic polyneuropathy | 3 | 165478799 | T/C | 0.248 | -0.037 | 0.087 | 0.672 | 0.248 | trans |
| FAM19A1 |  |  |  |  |  |  |  |  |  |  |
| rs1355538 | Diabetic polyneuropathy | 3 | 165505177 | A/G | 0.326 | -0.043 | 0.080 | 0.595 | 0.326 | trans |
| SPINT1 |  |  |  |  |  |  |  |  |  |  |
| rs9438975 | Diabetic polyneuropathy | 1 | 39350591 | A/G | 0.420 | 0.052 | 0.077 | 0.500 | 0.420 | trans |
| rs2419900 | Diabetic polyneuropathy | 2 | 60514364 | C/T | 0.392 | -0.018 | 0.078 | 0.821 | 0.392 | trans |
| rs708686 | Diabetic polyneuropathy | 19 | 5840619 | T/C | 0.334 | -0.105 | 0.081 | 0.194 | 0.334 | trans |
| rs17658212 | Diabetic polyneuropathy | 15 | 41145919 | T/C | 0.026 | -0.270 | 0.244 | 0.269 | 0.026 | trans |
| PCDHB1 |  |  |  |  |  |  |  |  |  |  |
| rs1707652 | Diabetic polyneuropathy | 3 | 165478799 | T/C | 0.248 | -0.037 | 0.087 | 0.672 | 0.248 | trans |
| TENC1 |  |  |  |  |  |  |  |  |  |  |
| rs3197999 | Diabetic polyneuropathy | 3 | 49721532 | A/G | 0.393 | -0.121 | 0.078 | 0.120 | 0.393 | trans |
| TCEAL8 |  |  |  |  |  |  |  |  |  |  |
| rs1706435 | Diabetic polyneuropathy | 3 | 165461476 | G/A | 0.310 | -0.079 | 0.082 | 0.331 | 0.310 | trans |
| TYMSOS |  |  |  |  |  |  |  |  |  |  |
| rs704 | Diabetic polyneuropathy | 17 | 26694861 | A/G | 0.420 | 0.049 | 0.076 | 0.519 | 0.420 | trans |
| THTPA |  |  |  |  |  |  |  |  |  |  |
| rs6445035 | Diabetic polyneuropathy | 3 | 165480100 | A/G | 0.176 | 0.000 | 0.099 | 0.998 | 0.176 | trans |
| PNMT |  |  |  |  |  |  |  |  |  |  |
| rs62294359 | Diabetic polyneuropathy | 3 | 165462217 | A/G | 0.243 | -0.040 | 0.088 | 0.650 | 0.243 | trans |
| CSDC2 |  |  |  |  |  |  |  |  |  |  |
| rs7412 | Diabetic polyneuropathy | 19 | 45412079 | T/C | 0.053 | 0.166 | 0.164 | 0.311 | 0.053 | trans |
| rs1707652 | Diabetic polyneuropathy | 3 | 165478799 | T/C | 0.248 | -0.037 | 0.087 | 0.672 | 0.248 | trans |
| LECT2 |  |  |  |  |  |  |  |  |  |  |
| rs62623707 | Diabetic polyneuropathy | 5 | 135288632 | G/A | 0.042 | 0.030 | 0.191 | 0.874 | 0.042 | cis |
| B4GALT3 |  |  |  |  |  |  |  |  |  |  |
| rs6542680 | Diabetic polyneuropathy | 2 | 3640142 | C/T | 0.351 | -0.070 | 0.081 | 0.384 | 0.351 | trans |
| SPIN1 |  |  |  |  |  |  |  |  |  |  |
| rs1707652 | Diabetic polyneuropathy | 3 | 165478799 | T/C | 0.248 | -0.037 | 0.087 | 0.672 | 0.248 | trans |
| RBP1 |  |  |  |  |  |  |  |  |  |  |
| rs2071387 | Diabetic polyneuropathy | 3 | 139257603 | G/A | 0.190 | -0.051 | 0.096 | 0.592 | 0.190 | cis |
| rs1706435 | Diabetic polyneuropathy | 3 | 165461476 | G/A | 0.310 | -0.079 | 0.082 | 0.331 | 0.310 | trans |
| TCL1B |  |  |  |  |  |  |  |  |  |  |
| rs6542680 | Diabetic polyneuropathy | 2 | 3640142 | C/T | 0.351 | -0.070 | 0.081 | 0.384 | 0.351 | trans |
| UBD |  |  |  |  |  |  |  |  |  |  |
| rs17713196 | Diabetic polyneuropathy | 3 | 165489724 | T/C | 0.174 | -0.015 | 0.099 | 0.878 | 0.174 | trans |
| RBBP5 |  |  |  |  |  |  |  |  |  |  |
| rs1355538 | Diabetic polyneuropathy | 3 | 165505177 | A/G | 0.326 | -0.043 | 0.080 | 0.595 | 0.326 | trans |
| RHPN2 |  |  |  |  |  |  |  |  |  |  |
| rs704 | Diabetic polyneuropathy | 17 | 26694861 | A/G | 0.420 | 0.049 | 0.076 | 0.519 | 0.420 | trans |
| rs1707652 | Diabetic polyneuropathy | 3 | 165478799 | T/C | 0.248 | -0.037 | 0.087 | 0.672 | 0.248 | trans |
| MMP17 |  |  |  |  |  |  |  |  |  |  |
| rs1707652 | Diabetic polyneuropathy | 3 | 165478799 | T/C | 0.248 | -0.037 | 0.087 | 0.672 | 0.248 | trans |
| rs6542680 | Diabetic polyneuropathy | 2 | 3640142 | C/T | 0.351 | -0.070 | 0.081 | 0.384 | 0.351 | trans |
| SNAI2 |  |  |  |  |  |  |  |  |  |  |
| rs1706435 | Diabetic polyneuropathy | 3 | 165461476 | G/A | 0.310 | -0.079 | 0.082 | 0.331 | 0.310 | trans |
| RCVRN |  |  |  |  |  |  |  |  |  |  |
| rs1707652 | Diabetic polyneuropathy | 3 | 165478799 | T/C | 0.248 | -0.037 | 0.087 | 0.672 | 0.248 | trans |
| KRT17 |  |  |  |  |  |  |  |  |  |  |
| rs2686395 | Diabetic polyneuropathy | 3 | 165485019 | T/C | 0.246 | -0.049 | 0.087 | 0.576 | 0.246 | trans |
| BRPF1 |  |  |  |  |  |  |  |  |  |  |
| rs704 | Diabetic polyneuropathy | 17 | 26694861 | A/G | 0.420 | 0.049 | 0.076 | 0.519 | 0.420 | trans |
| PPP1R8 |  |  |  |  |  |  |  |  |  |  |
| rs11720167 | Diabetic polyneuropathy | 3 | 165486145 | G/T | 0.174 | -0.015 | 0.099 | 0.877 | 0.174 | trans |
| MMEL1 |  |  |  |  |  |  |  |  |  |  |
| rs1355538 | Diabetic polyneuropathy | 3 | 165505177 | A/G | 0.326 | -0.043 | 0.080 | 0.595 | 0.326 | trans |
| MPP6 |  |  |  |  |  |  |  |  |  |  |
| rs704 | Diabetic polyneuropathy | 17 | 26694861 | A/G | 0.420 | 0.049 | 0.076 | 0.519 | 0.420 | trans |
| rs1706435 | Diabetic polyneuropathy | 3 | 165461476 | G/A | 0.310 | -0.079 | 0.082 | 0.331 | 0.310 | trans |
| MOBKL3 |  |  |  |  |  |  |  |  |  |  |
| rs1706435 | Diabetic polyneuropathy | 3 | 165461476 | G/A | 0.310 | -0.079 | 0.082 | 0.331 | 0.310 | trans |
| SPDEF |  |  |  |  |  |  |  |  |  |  |
| rs1707652 | Diabetic polyneuropathy | 3 | 165478799 | T/C | 0.248 | -0.037 | 0.087 | 0.672 | 0.248 | trans |
| ANTXR1 |  |  |  |  |  |  |  |  |  |  |
| rs13329672 | Diabetic polyneuropathy | 15 | 58699937 | T/C | 0.259 | 0.119 | 0.086 | 0.169 | 0.259 | trans |
| rs17410962 | Diabetic polyneuropathy | 8 | 19848080 | A/G | 0.094 | 0.074 | 0.128 | 0.564 | 0.094 | trans |
| rs2686395 | Diabetic polyneuropathy | 3 | 165485019 | T/C | 0.246 | -0.049 | 0.087 | 0.576 | 0.246 | trans |
| KLK9 |  |  |  |  |  |  |  |  |  |  |
| rs11720167 | Diabetic polyneuropathy | 3 | 165486145 | G/T | 0.174 | -0.015 | 0.099 | 0.877 | 0.174 | trans |
| rs3820897 | Diabetic polyneuropathy | 2 | 3642361 | T/C | 0.353 | -0.069 | 0.080 | 0.386 | 0.353 | trans |
| CSGALNACT2 |  |  |  |  |  |  |  |  |  |  |
| rs1355538 | Diabetic polyneuropathy | 3 | 165505177 | A/G | 0.326 | -0.043 | 0.080 | 0.595 | 0.326 | trans |
| rs6542680 | Diabetic polyneuropathy | 2 | 3640142 | C/T | 0.351 | -0.070 | 0.081 | 0.384 | 0.351 | trans |
| CYR61 |  |  |  |  |  |  |  |  |  |  |
| rs16830188 | Diabetic polyneuropathy | 1 | 25298841 | T/C | 0.010 | 0.387 | 0.378 | 0.306 | 0.010 | trans |
| CHI3L2 |  |  |  |  |  |  |  |  |  |  |
| rs2232613 | Diabetic polyneuropathy | 20 | 36997655 | T/C | 0.137 | 0.105 | 0.110 | 0.339 | 0.137 | trans |
| rs704 | Diabetic polyneuropathy | 17 | 26694861 | A/G | 0.420 | 0.049 | 0.076 | 0.519 | 0.420 | trans |
| KIAA1467 |  |  |  |  |  |  |  |  |  |  |
| rs117172138 | Diabetic polyneuropathy | 12 | 13193814 | T/C | 0.053 | 0.131 | 0.170 | 0.440 | 0.053 | cis |
| rs6445035 | Diabetic polyneuropathy | 3 | 165480100 | A/G | 0.176 | 0.000 | 0.099 | 0.998 | 0.176 | trans |
| rs28708758 | Diabetic polyneuropathy | 14 | 106612322 | G/T | 0.137 | -0.085 | 0.118 | 0.470 | 0.137 | trans |
| CRCP |  |  |  |  |  |  |  |  |  |  |
| rs704 | Diabetic polyneuropathy | 17 | 26694861 | A/G | 0.420 | 0.049 | 0.076 | 0.519 | 0.420 | trans |
| rs78119247 | Diabetic polyneuropathy | 3 | 165499135 | G/GAT | 0.173 | -0.017 | 0.100 | 0.866 | 0.173 | trans |
| KIF3A |  |  |  |  |  |  |  |  |  |  |
| rs2232613 | Diabetic polyneuropathy | 20 | 36997655 | T/C | 0.137 | 0.105 | 0.110 | 0.339 | 0.137 | trans |
| rs78119247 | Diabetic polyneuropathy | 3 | 165499135 | G/GAT | 0.173 | -0.017 | 0.100 | 0.866 | 0.173 | trans |
| FAM171A2 |  |  |  |  |  |  |  |  |  |  |
| rs704 | Diabetic polyneuropathy | 17 | 26694861 | A/G | 0.420 | 0.049 | 0.076 | 0.519 | 0.420 | trans |
| rs1706435 | Diabetic polyneuropathy | 3 | 165461476 | G/A | 0.310 | -0.079 | 0.082 | 0.331 | 0.310 | trans |
| CSF2RB |  |  |  |  |  |  |  |  |  |  |
| rs2239749 | Diabetic polyneuropathy | 22 | 37331305 | G/A | 0.584 | -0.031 | 0.077 | 0.685 | 0.416 | cis |
| rs1707652 | Diabetic polyneuropathy | 3 | 165478799 | T/C | 0.248 | -0.037 | 0.087 | 0.672 | 0.248 | trans |
| THRA |  |  |  |  |  |  |  |  |  |  |
| rs704 | Diabetic polyneuropathy | 17 | 26694861 | A/G | 0.420 | 0.049 | 0.076 | 0.519 | 0.420 | trans |
| rs1706435 | Diabetic polyneuropathy | 3 | 165461476 | G/A | 0.310 | -0.079 | 0.082 | 0.331 | 0.310 | trans |
| FAXDC2 |  |  |  |  |  |  |  |  |  |  |
| rs61808983 | Diabetic polyneuropathy | 1 | 169474397 | T/C | 0.043 | 0.153 | 0.188 | 0.416 | 0.043 | trans |
| rs1706435 | Diabetic polyneuropathy | 3 | 165461476 | G/A | 0.310 | -0.079 | 0.082 | 0.331 | 0.310 | trans |
| CEACAM3 |  |  |  |  |  |  |  |  |  |  |
| rs1707652 | Diabetic polyneuropathy | 3 | 165478799 | T/C | 0.248 | -0.037 | 0.087 | 0.672 | 0.248 | trans |
| CDH8 |  |  |  |  |  |  |  |  |  |  |
| rs2686395 | Diabetic polyneuropathy | 3 | 165485019 | T/C | 0.246 | -0.049 | 0.087 | 0.576 | 0.246 | trans |
| ULBP1 |  |  |  |  |  |  |  |  |  |  |
| rs6542680 | Diabetic polyneuropathy | 2 | 3640142 | C/T | 0.351 | -0.070 | 0.081 | 0.384 | 0.351 | trans |
| CPT1B |  |  |  |  |  |  |  |  |  |  |
| rs17713088 | Diabetic polyneuropathy | 3 | 165488604 | T/G | 0.174 | -0.015 | 0.099 | 0.878 | 0.174 | trans |
| SULT1C2 |  |  |  |  |  |  |  |  |  |  |
| rs1706435 | Diabetic polyneuropathy | 3 | 165461476 | G/A | 0.310 | -0.079 | 0.082 | 0.331 | 0.310 | trans |
| SFXN5 |  |  |  |  |  |  |  |  |  |  |
| rs238238 | Diabetic polyneuropathy | 17 | 4856376 | A/G | 0.368 | 0.200 | 0.079 | 0.011 | 0.368 | cis |
| SORBS3 |  |  |  |  |  |  |  |  |  |  |
| rs1355538 | Diabetic polyneuropathy | 3 | 165505177 | A/G | 0.326 | -0.043 | 0.080 | 0.595 | 0.326 | trans |
| POMK |  |  |  |  |  |  |  |  |  |  |
| rs2686395 | Diabetic polyneuropathy | 3 | 165485019 | T/C | 0.246 | -0.049 | 0.087 | 0.576 | 0.246 | trans |
| SNX17 |  |  |  |  |  |  |  |  |  |  |
| rs1706435 | Diabetic polyneuropathy | 3 | 165461476 | G/A | 0.310 | -0.079 | 0.082 | 0.331 | 0.310 | trans |
| HERC4 |  |  |  |  |  |  |  |  |  |  |
| rs704 | Diabetic polyneuropathy | 17 | 26694861 | A/G | 0.420 | 0.049 | 0.076 | 0.519 | 0.420 | trans |
| LEPRE1 |  |  |  |  |  |  |  |  |  |  |
| rs1707652 | Diabetic polyneuropathy | 3 | 165478799 | T/C | 0.248 | -0.037 | 0.087 | 0.672 | 0.248 | trans |
| SMURF1 |  |  |  |  |  |  |  |  |  |  |
| rs4419136 | Diabetic polyneuropathy | 18 | 55723630 | A/G | 0.221 | 0.021 | 0.091 | 0.821 | 0.221 | trans |
| PAPLN |  |  |  |  |  |  |  |  |  |  |
| rs1355538 | Diabetic polyneuropathy | 3 | 165505177 | A/G | 0.326 | -0.043 | 0.080 | 0.595 | 0.326 | trans |
| IL3 |  |  |  |  |  |  |  |  |  |  |
| rs1355538 | Diabetic polyneuropathy | 3 | 165505177 | A/G | 0.326 | -0.043 | 0.080 | 0.595 | 0.326 | trans |
| KDELC1 |  |  |  |  |  |  |  |  |  |  |
| rs11720167 | Diabetic polyneuropathy | 3 | 165486145 | G/T | 0.174 | -0.015 | 0.099 | 0.877 | 0.174 | trans |
| MAN2B2 |  |  |  |  |  |  |  |  |  |  |
| rs11155297 | Diabetic polyneuropathy | 6 | 143825104 | T/G | 0.206 | 0.079 | 0.093 | 0.396 | 0.206 | trans |
| OLFML1 |  |  |  |  |  |  |  |  |  |  |
| rs1355538 | Diabetic polyneuropathy | 3 | 165505177 | A/G | 0.326 | -0.043 | 0.080 | 0.595 | 0.326 | trans |
| KRT20 |  |  |  |  |  |  |  |  |  |  |
| rs4796667 | Diabetic polyneuropathy | 17 | 39678716 | T/C | 0.354 | -0.026 | 0.079 | 0.745 | 0.354 | cis |
| TARDBP |  |  |  |  |  |  |  |  |  |  |
| rs1707652 | Diabetic polyneuropathy | 3 | 165478799 | T/C | 0.248 | -0.037 | 0.087 | 0.672 | 0.248 | trans |
| EVL |  |  |  |  |  |  |  |  |  |  |
| rs75416067 | Diabetic polyneuropathy | 14 | 100421429 | C/CT | 0.832 | -0.059 | 0.103 | 0.572 | 0.168 | cis |
| RAD51 |  |  |  |  |  |  |  |  |  |  |
| rs1707652 | Diabetic polyneuropathy | 3 | 165478799 | T/C | 0.248 | -0.037 | 0.087 | 0.672 | 0.248 | trans |
| CYB5R1 |  |  |  |  |  |  |  |  |  |  |
| rs6542680 | Diabetic polyneuropathy | 2 | 3640142 | C/T | 0.351 | -0.070 | 0.081 | 0.384 | 0.351 | trans |
| rs1706435 | Diabetic polyneuropathy | 3 | 165461476 | G/A | 0.310 | -0.079 | 0.082 | 0.331 | 0.310 | trans |
| rs34762726 | Diabetic polyneuropathy | 3 | 49689210 | A/G | 0.413 | -0.140 | 0.077 | 0.069 | 0.413 | trans |
| RNASE10 |  |  |  |  |  |  |  |  |  |  |
| rs6542680 | Diabetic polyneuropathy | 2 | 3640142 | C/T | 0.351 | -0.070 | 0.081 | 0.384 | 0.351 | trans |
| RPP30 |  |  |  |  |  |  |  |  |  |  |
| rs1706435 | Diabetic polyneuropathy | 3 | 165461476 | G/A | 0.310 | -0.079 | 0.082 | 0.331 | 0.310 | trans |
| DOS |  |  |  |  |  |  |  |  |  |  |
| rs11651658 | Diabetic polyneuropathy | 17 | 64198640 | C/T | 0.100 | 0.192 | 0.125 | 0.123 | 0.100 | trans |
| SPTLC2 |  |  |  |  |  |  |  |  |  |  |
| rs1707652 | Diabetic polyneuropathy | 3 | 165478799 | T/C | 0.248 | -0.037 | 0.087 | 0.672 | 0.248 | trans |
| BMP15 |  |  |  |  |  |  |  |  |  |  |
| rs78119247 | Diabetic polyneuropathy | 3 | 165499135 | G/GAT | 0.173 | -0.017 | 0.100 | 0.866 | 0.173 | trans |
| KIR3DL3 |  |  |  |  |  |  |  |  |  |  |
| rs2317230 | Diabetic polyneuropathy | 1 | 157674997 | T/G | 0.411 | 0.064 | 0.077 | 0.407 | 0.411 | trans |
| CD81 |  |  |  |  |  |  |  |  |  |  |
| rs1355538 | Diabetic polyneuropathy | 3 | 165505177 | A/G | 0.326 | -0.043 | 0.080 | 0.595 | 0.326 | trans |
| CDC42BPB |  |  |  |  |  |  |  |  |  |  |
| rs1355538 | Diabetic polyneuropathy | 3 | 165505177 | A/G | 0.326 | -0.043 | 0.080 | 0.595 | 0.326 | trans |
| JUN |  |  |  |  |  |  |  |  |  |  |
| rs704 | Diabetic polyneuropathy | 17 | 26694861 | A/G | 0.420 | 0.049 | 0.076 | 0.519 | 0.420 | trans |
| rs78119247 | Diabetic polyneuropathy | 3 | 165499135 | G/GAT | 0.173 | -0.017 | 0.100 | 0.866 | 0.173 | trans |
| CPLX1 |  |  |  |  |  |  |  |  |  |  |
| rs1355538 | Diabetic polyneuropathy | 3 | 165505177 | A/G | 0.326 | -0.043 | 0.080 | 0.595 | 0.326 | trans |
| rs7687101 | Diabetic polyneuropathy | 4 | 812679 | T/G | 0.302 | -0.066 | 0.082 | 0.422 | 0.302 | cis |
| SH2D1B |  |  |  |  |  |  |  |  |  |  |
| rs140431012 | Diabetic polyneuropathy | 3 | 124473930 | G/A | 0.103 | 0.027 | 0.124 | 0.828 | 0.103 | trans |
| ANXA10 |  |  |  |  |  |  |  |  |  |  |
| rs1706435 | Diabetic polyneuropathy | 3 | 165461476 | G/A | 0.310 | -0.079 | 0.082 | 0.331 | 0.310 | trans |
| ETFA |  |  |  |  |  |  |  |  |  |  |
| rs704 | Diabetic polyneuropathy | 17 | 26694861 | A/G | 0.420 | 0.049 | 0.076 | 0.519 | 0.420 | trans |
| rs79495512 | Diabetic polyneuropathy | 15 | 76492337 | T/C | 0.877 | 0.043 | 0.116 | 0.712 | 0.123 | cis |
| rs78119247 | Diabetic polyneuropathy | 3 | 165499135 | G/GAT | 0.173 | -0.017 | 0.100 | 0.866 | 0.173 | trans |
| BMPR2 |  |  |  |  |  |  |  |  |  |  |
| rs1706435 | Diabetic polyneuropathy | 3 | 165461476 | G/A | 0.310 | -0.079 | 0.082 | 0.331 | 0.310 | trans |
| MGAT4A |  |  |  |  |  |  |  |  |  |  |
| rs1707652 | Diabetic polyneuropathy | 3 | 165478799 | T/C | 0.248 | -0.037 | 0.087 | 0.672 | 0.248 | trans |
| CCDC64 |  |  |  |  |  |  |  |  |  |  |
| rs1355538 | Diabetic polyneuropathy | 3 | 165505177 | A/G | 0.326 | -0.043 | 0.080 | 0.595 | 0.326 | trans |
| rs6542680 | Diabetic polyneuropathy | 2 | 3640142 | C/T | 0.351 | -0.070 | 0.081 | 0.384 | 0.351 | trans |
| BTNL3 |  |  |  |  |  |  |  |  |  |  |
| rs6445035 | Diabetic polyneuropathy | 3 | 165480100 | A/G | 0.176 | 0.000 | 0.099 | 0.998 | 0.176 | trans |
| CCNH |  |  |  |  |  |  |  |  |  |  |
| rs62368956 | Diabetic polyneuropathy | 5 | 86735368 | A/G | 0.253 | 0.110 | 0.087 | 0.209 | 0.253 | cis |
| rs41452950 | Diabetic polyneuropathy | 1 | 57420627 | A/G | 0.963 | -0.014 | 0.199 | 0.944 | 0.037 | trans |
| HINT2 |  |  |  |  |  |  |  |  |  |  |
| rs1355538 | Diabetic polyneuropathy | 3 | 165505177 | A/G | 0.326 | -0.043 | 0.080 | 0.595 | 0.326 | trans |
| P2RX6 |  |  |  |  |  |  |  |  |  |  |
| rs1355538 | Diabetic polyneuropathy | 3 | 165505177 | A/G | 0.326 | -0.043 | 0.080 | 0.595 | 0.326 | trans |
| IGJ |  |  |  |  |  |  |  |  |  |  |
| rs1707652 | Diabetic polyneuropathy | 3 | 165478799 | T/C | 0.248 | -0.037 | 0.087 | 0.672 | 0.248 | trans |
| PBK |  |  |  |  |  |  |  |  |  |  |
| rs704 | Diabetic polyneuropathy | 17 | 26694861 | A/G | 0.420 | 0.049 | 0.076 | 0.519 | 0.420 | trans |
| rs1707652 | Diabetic polyneuropathy | 3 | 165478799 | T/C | 0.248 | -0.037 | 0.087 | 0.672 | 0.248 | trans |
| CEBPG |  |  |  |  |  |  |  |  |  |  |
| rs4566588 | Diabetic polyneuropathy | 3 | 165462568 | T/C | 0.243 | -0.040 | 0.088 | 0.650 | 0.243 | trans |
| IGSF9B |  |  |  |  |  |  |  |  |  |  |
| rs6542680 | Diabetic polyneuropathy | 2 | 3640142 | C/T | 0.351 | -0.070 | 0.081 | 0.384 | 0.351 | trans |
| LMAN1 |  |  |  |  |  |  |  |  |  |  |
| rs12126142 | Diabetic polyneuropathy | 1 | 154425456 | A/G | 0.297 | -0.021 | 0.083 | 0.800 | 0.297 | trans |
| rs1706435 | Diabetic polyneuropathy | 3 | 165461476 | G/A | 0.310 | -0.079 | 0.082 | 0.331 | 0.310 | trans |
| TRAF1 |  |  |  |  |  |  |  |  |  |  |
| rs704 | Diabetic polyneuropathy | 17 | 26694861 | A/G | 0.420 | 0.049 | 0.076 | 0.519 | 0.420 | trans |
| rs1707652 | Diabetic polyneuropathy | 3 | 165478799 | T/C | 0.248 | -0.037 | 0.087 | 0.672 | 0.248 | trans |
| rs11569415 | Diabetic polyneuropathy | 19 | 6716279 | A/G | 0.185 | -0.101 | 0.098 | 0.304 | 0.185 | cis |
| RAB27B |  |  |  |  |  |  |  |  |  |  |
| rs6445035 | Diabetic polyneuropathy | 3 | 165480100 | A/G | 0.176 | 0.000 | 0.099 | 0.998 | 0.176 | trans |
| RXFP1 |  |  |  |  |  |  |  |  |  |  |
| rs6542680 | Diabetic polyneuropathy | 2 | 3640142 | C/T | 0.351 | -0.070 | 0.081 | 0.384 | 0.351 | trans |
| rs1706435 | Diabetic polyneuropathy | 3 | 165461476 | G/A | 0.310 | -0.079 | 0.082 | 0.331 | 0.310 | trans |
| DUSP26 |  |  |  |  |  |  |  |  |  |  |
| rs71352239 | Diabetic polyneuropathy | 19 | 45429543 | T/C | 0.304 | 0.094 | 0.084 | 0.263 | 0.304 | trans |
| rs1707652 | Diabetic polyneuropathy | 3 | 165478799 | T/C | 0.248 | -0.037 | 0.087 | 0.672 | 0.248 | trans |
| rs12478841 | Diabetic polyneuropathy | 2 | 27811722 | G/A | 0.280 | -0.054 | 0.084 | 0.524 | 0.280 | trans |
| PDE4D |  |  |  |  |  |  |  |  |  |  |
| rs1619994 | Diabetic polyneuropathy | 3 | 165461835 | G/A | 0.252 | -0.063 | 0.087 | 0.470 | 0.252 | trans |
| ESRRA |  |  |  |  |  |  |  |  |  |  |
| rs1706435 | Diabetic polyneuropathy | 3 | 165461476 | G/A | 0.310 | -0.079 | 0.082 | 0.331 | 0.310 | trans |
| TRIL |  |  |  |  |  |  |  |  |  |  |
| rs704 | Diabetic polyneuropathy | 17 | 26694861 | A/G | 0.420 | 0.049 | 0.076 | 0.519 | 0.420 | trans |
| rs1707652 | Diabetic polyneuropathy | 3 | 165478799 | T/C | 0.248 | -0.037 | 0.087 | 0.672 | 0.248 | trans |
| RAB17 |  |  |  |  |  |  |  |  |  |  |
| rs1707652 | Diabetic polyneuropathy | 3 | 165478799 | T/C | 0.248 | -0.037 | 0.087 | 0.672 | 0.248 | trans |
| CASC4 |  |  |  |  |  |  |  |  |  |  |
| rs2230281 | Diabetic polyneuropathy | 12 | 89917518 | A/G | 0.306 | 0.128 | 0.082 | 0.118 | 0.306 | trans |
| rs550057 | Diabetic polyneuropathy | 9 | 136146597 | T/C | 0.302 | 0.128 | 0.082 | 0.121 | 0.302 | trans |
| KIAA0319L |  |  |  |  |  |  |  |  |  |  |
| rs1707652 | Diabetic polyneuropathy | 3 | 165478799 | T/C | 0.248 | -0.037 | 0.087 | 0.672 | 0.248 | trans |
| SYT8 |  |  |  |  |  |  |  |  |  |  |
| rs1706435 | Diabetic polyneuropathy | 3 | 165461476 | G/A | 0.310 | -0.079 | 0.082 | 0.331 | 0.310 | trans |
| TAF10 |  |  |  |  |  |  |  |  |  |  |
| rs1707652 | Diabetic polyneuropathy | 3 | 165478799 | T/C | 0.248 | -0.037 | 0.087 | 0.672 | 0.248 | trans |
| CRIM1 |  |  |  |  |  |  |  |  |  |  |
| rs17713196 | Diabetic polyneuropathy | 3 | 165489724 | T/C | 0.174 | -0.015 | 0.099 | 0.878 | 0.174 | trans |
| PCDHB2 |  |  |  |  |  |  |  |  |  |  |
| rs11720167 | Diabetic polyneuropathy | 3 | 165486145 | G/T | 0.174 | -0.015 | 0.099 | 0.877 | 0.174 | trans |
| DEFB107A |  |  |  |  |  |  |  |  |  |  |
| rs6542680 | Diabetic polyneuropathy | 2 | 3640142 | C/T | 0.351 | -0.070 | 0.081 | 0.384 | 0.351 | trans |
| BCAP29 |  |  |  |  |  |  |  |  |  |  |
| rs704 | Diabetic polyneuropathy | 17 | 26694861 | A/G | 0.420 | 0.049 | 0.076 | 0.519 | 0.420 | trans |
| rs1707652 | Diabetic polyneuropathy | 3 | 165478799 | T/C | 0.248 | -0.037 | 0.087 | 0.672 | 0.248 | trans |
| SPR |  |  |  |  |  |  |  |  |  |  |
| rs1355538 | Diabetic polyneuropathy | 3 | 165505177 | A/G | 0.326 | -0.043 | 0.080 | 0.595 | 0.326 | trans |
| GPNMB |  |  |  |  |  |  |  |  |  |  |
| rs2268748 | Diabetic polyneuropathy | 7 | 23313171 | T/C | 0.917 | 0.033 | 0.138 | 0.810 | 0.083 | cis |
| rs1881203 | Diabetic polyneuropathy | 7 | 23292808 | T/C | 0.711 | -0.073 | 0.083 | 0.378 | 0.289 | cis |
| rs12203592 | Diabetic polyneuropathy | 6 | 396321 | T/C | 0.031 | -0.186 | 0.222 | 0.402 | 0.031 | trans |
| OSTM1 |  |  |  |  |  |  |  |  |  |  |
| rs1706435 | Diabetic polyneuropathy | 3 | 165461476 | G/A | 0.310 | -0.079 | 0.082 | 0.331 | 0.310 | trans |
| OTUB2 |  |  |  |  |  |  |  |  |  |  |
| rs72673751 | Diabetic polyneuropathy | 8 | 106578940 | C/T | 0.142 | -0.008 | 0.108 | 0.945 | 0.142 | trans |
| PAK7 |  |  |  |  |  |  |  |  |  |  |
| rs1706435 | Diabetic polyneuropathy | 3 | 165461476 | G/A | 0.310 | -0.079 | 0.082 | 0.331 | 0.310 | trans |
| LGALS9 |  |  |  |  |  |  |  |  |  |  |
| rs516246 | Diabetic polyneuropathy | 19 | 49206172 | T/C | 0.375 | -0.046 | 0.078 | 0.556 | 0.375 | trans |
| rs2686395 | Diabetic polyneuropathy | 3 | 165485019 | T/C | 0.246 | -0.049 | 0.087 | 0.576 | 0.246 | trans |
| POLE2 |  |  |  |  |  |  |  |  |  |  |
| rs893522 | Diabetic polyneuropathy | 3 | 165560618 | A/C | 0.918 | -0.111 | 0.137 | 0.419 | 0.082 | trans |
| RNF34 |  |  |  |  |  |  |  |  |  |  |
| rs1706435 | Diabetic polyneuropathy | 3 | 165461476 | G/A | 0.310 | -0.079 | 0.082 | 0.331 | 0.310 | trans |
| RPS10 |  |  |  |  |  |  |  |  |  |  |
| rs1707652 | Diabetic polyneuropathy | 3 | 165478799 | T/C | 0.248 | -0.037 | 0.087 | 0.672 | 0.248 | trans |
| PTH1R |  |  |  |  |  |  |  |  |  |  |
| rs1706435 | Diabetic polyneuropathy | 3 | 165461476 | G/A | 0.310 | -0.079 | 0.082 | 0.331 | 0.310 | trans |
| TNFRSF10D |  |  |  |  |  |  |  |  |  |  |
| rs7638018 | Diabetic polyneuropathy | 3 | 133495461 | G/A | 0.304 | 0.047 | 0.082 | 0.563 | 0.304 | trans |
| PCDHB10 |  |  |  |  |  |  |  |  |  |  |
| rs1706435 | Diabetic polyneuropathy | 3 | 165461476 | G/A | 0.310 | -0.079 | 0.082 | 0.331 | 0.310 | trans |
| GRID2 |  |  |  |  |  |  |  |  |  |  |
| rs2511737 | Diabetic polyneuropathy | 8 | 103580527 | C/A | 0.290 | 0.058 | 0.084 | 0.490 | 0.290 | trans |
| rs142279279 | Diabetic polyneuropathy | 2 | 119716505 | A/G | 0.133 | 0.055 | 0.112 | 0.627 | 0.133 | trans |
| rs12798553 | Diabetic polyneuropathy | 11 | 123353693 | A/G | 0.227 | -0.049 | 0.090 | 0.587 | 0.227 | trans |
| rs2476601 | Diabetic polyneuropathy | 1 | 114377568 | G/A | 0.852 | -0.179 | 0.107 | 0.093 | 0.148 | trans |
| GKN1 |  |  |  |  |  |  |  |  |  |  |
| rs2686395 | Diabetic polyneuropathy | 3 | 165485019 | T/C | 0.246 | -0.049 | 0.087 | 0.576 | 0.246 | trans |
| ADAM17 |  |  |  |  |  |  |  |  |  |  |
| rs1355538 | Diabetic polyneuropathy | 3 | 165505177 | A/G | 0.326 | -0.043 | 0.080 | 0.595 | 0.326 | trans |
| CARD18 |  |  |  |  |  |  |  |  |  |  |
| rs17713196 | Diabetic polyneuropathy | 3 | 165489724 | T/C | 0.174 | -0.015 | 0.099 | 0.878 | 0.174 | trans |
| JTB |  |  |  |  |  |  |  |  |  |  |
| rs2256952 | Diabetic polyneuropathy | 20 | 44019807 | C/T | 0.247 | 0.030 | 0.088 | 0.735 | 0.247 | trans |
| rs1707652 | Diabetic polyneuropathy | 3 | 165478799 | T/C | 0.248 | -0.037 | 0.087 | 0.672 | 0.248 | trans |
| PYY |  |  |  |  |  |  |  |  |  |  |
| rs7116087 | Diabetic polyneuropathy | 11 | 111133005 | T/C | 0.761 | -0.012 | 0.089 | 0.893 | 0.239 | trans |
| CAMK4 |  |  |  |  |  |  |  |  |  |  |
| rs1706435 | Diabetic polyneuropathy | 3 | 165461476 | G/A | 0.310 | -0.079 | 0.082 | 0.331 | 0.310 | trans |
| AKR1C3 |  |  |  |  |  |  |  |  |  |  |
| rs6592965 | Diabetic polyneuropathy | 7 | 50427982 | A/G | 0.321 | 0.011 | 0.081 | 0.894 | 0.321 | trans |
| rs2245801 | Diabetic polyneuropathy | 4 | 90757840 | T/C | 0.167 | -0.001 | 0.101 | 0.994 | 0.167 | trans |
| rs11557154 | Diabetic polyneuropathy | 9 | 34107505 | T/C | 0.132 | -0.026 | 0.112 | 0.819 | 0.132 | trans |
| rs1707652 | Diabetic polyneuropathy | 3 | 165478799 | T/C | 0.248 | -0.037 | 0.087 | 0.672 | 0.248 | trans |
| rs35301881 | Diabetic polyneuropathy | 1 | 1681665 | T/C | 0.388 | -0.066 | 0.078 | 0.396 | 0.388 | trans |
| ETNK1 |  |  |  |  |  |  |  |  |  |  |
| rs1355538 | Diabetic polyneuropathy | 3 | 165505177 | A/G | 0.326 | -0.043 | 0.080 | 0.595 | 0.326 | trans |
| TRIB2 |  |  |  |  |  |  |  |  |  |  |
| rs697357 | Diabetic polyneuropathy | 3 | 165436272 | G/A | 0.338 | -0.054 | 0.080 | 0.496 | 0.338 | trans |
| MGAT1 |  |  |  |  |  |  |  |  |  |  |
| rs634501 | Diabetic polyneuropathy | 5 | 180218668 | A/G | 0.215 | -0.068 | 0.097 | 0.482 | 0.215 | cis |
| RAB39B |  |  |  |  |  |  |  |  |  |  |
| rs1355538 | Diabetic polyneuropathy | 3 | 165505177 | A/G | 0.326 | -0.043 | 0.080 | 0.595 | 0.326 | trans |
| PTGR2 |  |  |  |  |  |  |  |  |  |  |
| rs1707652 | Diabetic polyneuropathy | 3 | 165478799 | T/C | 0.248 | -0.037 | 0.087 | 0.672 | 0.248 | trans |
| BATF |  |  |  |  |  |  |  |  |  |  |
| rs6445035 | Diabetic polyneuropathy | 3 | 165480100 | A/G | 0.176 | 0.000 | 0.099 | 0.998 | 0.176 | trans |
| rs6542680 | Diabetic polyneuropathy | 2 | 3640142 | C/T | 0.351 | -0.070 | 0.081 | 0.384 | 0.351 | trans |
| CCNB1 |  |  |  |  |  |  |  |  |  |  |
| rs1619994 | Diabetic polyneuropathy | 3 | 165461835 | G/A | 0.252 | -0.063 | 0.087 | 0.470 | 0.252 | trans |
| SLC3A1 |  |  |  |  |  |  |  |  |  |  |
| rs704 | Diabetic polyneuropathy | 17 | 26694861 | A/G | 0.420 | 0.049 | 0.076 | 0.519 | 0.420 | trans |
| rs1355538 | Diabetic polyneuropathy | 3 | 165505177 | A/G | 0.326 | -0.043 | 0.080 | 0.595 | 0.326 | trans |
| IFNA1 |  |  |  |  |  |  |  |  |  |  |
| rs1355538 | Diabetic polyneuropathy | 3 | 165505177 | A/G | 0.326 | -0.043 | 0.080 | 0.595 | 0.326 | trans |
| IL13 |  |  |  |  |  |  |  |  |  |  |
| rs6445035 | Diabetic polyneuropathy | 3 | 165480100 | A/G | 0.176 | 0.000 | 0.099 | 0.998 | 0.176 | trans |
| IL1F5 |  |  |  |  |  |  |  |  |  |  |
| rs704 | Diabetic polyneuropathy | 17 | 26694861 | A/G | 0.420 | 0.049 | 0.076 | 0.519 | 0.420 | trans |
| rs1707652 | Diabetic polyneuropathy | 3 | 165478799 | T/C | 0.248 | -0.037 | 0.087 | 0.672 | 0.248 | trans |
| TRADD |  |  |  |  |  |  |  |  |  |  |
| rs71352239 | Diabetic polyneuropathy | 19 | 45429543 | T/C | 0.304 | 0.094 | 0.084 | 0.263 | 0.304 | trans |
| rs704 | Diabetic polyneuropathy | 17 | 26694861 | A/G | 0.420 | 0.049 | 0.076 | 0.519 | 0.420 | trans |
| rs1707652 | Diabetic polyneuropathy | 3 | 165478799 | T/C | 0.248 | -0.037 | 0.087 | 0.672 | 0.248 | trans |
| rs11569415 | Diabetic polyneuropathy | 19 | 6716279 | A/G | 0.185 | -0.101 | 0.098 | 0.304 | 0.185 | cis |
| SEL1L2 |  |  |  |  |  |  |  |  |  |  |
| rs704 | Diabetic polyneuropathy | 17 | 26694861 | A/G | 0.420 | 0.049 | 0.076 | 0.519 | 0.420 | trans |
| SULT2B1 |  |  |  |  |  |  |  |  |  |  |
| rs2686395 | Diabetic polyneuropathy | 3 | 165485019 | T/C | 0.246 | -0.049 | 0.087 | 0.576 | 0.246 | trans |
| LRRC3B |  |  |  |  |  |  |  |  |  |  |
| rs71352239 | Diabetic polyneuropathy | 19 | 45429543 | T/C | 0.304 | 0.094 | 0.084 | 0.263 | 0.304 | trans |
| rs1707652 | Diabetic polyneuropathy | 3 | 165478799 | T/C | 0.248 | -0.037 | 0.087 | 0.672 | 0.248 | trans |
| ELL |  |  |  |  |  |  |  |  |  |  |
| rs704 | Diabetic polyneuropathy | 17 | 26694861 | A/G | 0.420 | 0.049 | 0.076 | 0.519 | 0.420 | trans |
| SERF1A |  |  |  |  |  |  |  |  |  |  |
| rs1355538 | Diabetic polyneuropathy | 3 | 165505177 | A/G | 0.326 | -0.043 | 0.080 | 0.595 | 0.326 | trans |
| NPM1 |  |  |  |  |  |  |  |  |  |  |
| rs1707652 | Diabetic polyneuropathy | 3 | 165478799 | T/C | 0.248 | -0.037 | 0.087 | 0.672 | 0.248 | trans |
| PLBD2 |  |  |  |  |  |  |  |  |  |  |
| rs1706435 | Diabetic polyneuropathy | 3 | 165461476 | G/A | 0.310 | -0.079 | 0.082 | 0.331 | 0.310 | trans |
| CPD |  |  |  |  |  |  |  |  |  |  |
| rs1707652 | Diabetic polyneuropathy | 3 | 165478799 | T/C | 0.248 | -0.037 | 0.087 | 0.672 | 0.248 | trans |
| SYNJ2BP |  |  |  |  |  |  |  |  |  |  |
| rs1706435 | Diabetic polyneuropathy | 3 | 165461476 | G/A | 0.310 | -0.079 | 0.082 | 0.331 | 0.310 | trans |
| GOSR2 |  |  |  |  |  |  |  |  |  |  |
| rs2686395 | Diabetic polyneuropathy | 3 | 165485019 | T/C | 0.246 | -0.049 | 0.087 | 0.576 | 0.246 | trans |
| SLC26A11 |  |  |  |  |  |  |  |  |  |  |
| rs704 | Diabetic polyneuropathy | 17 | 26694861 | A/G | 0.420 | 0.049 | 0.076 | 0.519 | 0.420 | trans |
| rs1707652 | Diabetic polyneuropathy | 3 | 165478799 | T/C | 0.248 | -0.037 | 0.087 | 0.672 | 0.248 | trans |
| PMEPA1 |  |  |  |  |  |  |  |  |  |  |
| rs11720167 | Diabetic polyneuropathy | 3 | 165486145 | G/T | 0.174 | -0.015 | 0.099 | 0.877 | 0.174 | trans |
| LIN7C |  |  |  |  |  |  |  |  |  |  |
| rs2686395 | Diabetic polyneuropathy | 3 | 165485019 | T/C | 0.246 | -0.049 | 0.087 | 0.576 | 0.246 | trans |
| rs11569415 | Diabetic polyneuropathy | 19 | 6716279 | A/G | 0.185 | -0.101 | 0.098 | 0.304 | 0.185 | cis |
| RNF43 |  |  |  |  |  |  |  |  |  |  |
| rs1707652 | Diabetic polyneuropathy | 3 | 165478799 | T/C | 0.248 | -0.037 | 0.087 | 0.672 | 0.248 | trans |
| GRID1 |  |  |  |  |  |  |  |  |  |  |
| rs6445035 | Diabetic polyneuropathy | 3 | 165480100 | A/G | 0.176 | 0.000 | 0.099 | 0.998 | 0.176 | trans |
| CCDC90B |  |  |  |  |  |  |  |  |  |  |
| rs6542680 | Diabetic polyneuropathy | 2 | 3640142 | C/T | 0.351 | -0.070 | 0.081 | 0.384 | 0.351 | trans |
| HDAC8 |  |  |  |  |  |  |  |  |  |  |
| rs71352239 | Diabetic polyneuropathy | 19 | 45429543 | T/C | 0.304 | 0.094 | 0.084 | 0.263 | 0.304 | trans |
| rs1707652 | Diabetic polyneuropathy | 3 | 165478799 | T/C | 0.248 | -0.037 | 0.087 | 0.672 | 0.248 | trans |
| GPR64 |  |  |  |  |  |  |  |  |  |  |
| rs1619994 | Diabetic polyneuropathy | 3 | 165461835 | G/A | 0.252 | -0.063 | 0.087 | 0.470 | 0.252 | trans |
| MRPL52 |  |  |  |  |  |  |  |  |  |  |
| rs1355538 | Diabetic polyneuropathy | 3 | 165505177 | A/G | 0.326 | -0.043 | 0.080 | 0.595 | 0.326 | trans |
| BECN1 |  |  |  |  |  |  |  |  |  |  |
| rs1355538 | Diabetic polyneuropathy | 3 | 165505177 | A/G | 0.326 | -0.043 | 0.080 | 0.595 | 0.326 | trans |
| NR1H4 |  |  |  |  |  |  |  |  |  |  |
| rs1707652 | Diabetic polyneuropathy | 3 | 165478799 | T/C | 0.248 | -0.037 | 0.087 | 0.672 | 0.248 | trans |
| PSMA2 |  |  |  |  |  |  |  |  |  |  |
| rs2686395 | Diabetic polyneuropathy | 3 | 165485019 | T/C | 0.246 | -0.049 | 0.087 | 0.576 | 0.246 | trans |
| rs11569415 | Diabetic polyneuropathy | 19 | 6716279 | A/G | 0.185 | -0.101 | 0.098 | 0.304 | 0.185 | cis |
| CMA1 |  |  |  |  |  |  |  |  |  |  |
| rs1706435 | Diabetic polyneuropathy | 3 | 165461476 | G/A | 0.310 | -0.079 | 0.082 | 0.331 | 0.310 | trans |
| ZNF174 |  |  |  |  |  |  |  |  |  |  |
| rs1355538 | Diabetic polyneuropathy | 3 | 165505177 | A/G | 0.326 | -0.043 | 0.080 | 0.595 | 0.326 | trans |
| TP53 |  |  |  |  |  |  |  |  |  |  |
| rs2232613 | Diabetic polyneuropathy | 20 | 36997655 | T/C | 0.137 | 0.105 | 0.110 | 0.339 | 0.137 | trans |
| rs17713088 | Diabetic polyneuropathy | 3 | 165488604 | T/G | 0.174 | -0.015 | 0.099 | 0.878 | 0.174 | trans |
| rs5167 | Diabetic polyneuropathy | 19 | 45448465 | G/T | 0.415 | -0.084 | 0.077 | 0.276 | 0.415 | trans |
| TPD52L1 |  |  |  |  |  |  |  |  |  |  |
| rs2686395 | Diabetic polyneuropathy | 3 | 165485019 | T/C | 0.246 | -0.049 | 0.087 | 0.576 | 0.246 | trans |
| KCNA10 |  |  |  |  |  |  |  |  |  |  |
| rs2583262 | Diabetic polyneuropathy | 12 | 104339014 | T/C | 0.279 | 0.097 | 0.084 | 0.248 | 0.279 | trans |
| SYT2 |  |  |  |  |  |  |  |  |  |  |
| rs1619994 | Diabetic polyneuropathy | 3 | 165461835 | G/A | 0.252 | -0.063 | 0.087 | 0.470 | 0.252 | trans |
| SYNCRIP |  |  |  |  |  |  |  |  |  |  |
| rs1354034 | Diabetic polyneuropathy | 3 | 56849749 | C/T | 0.707 | 0.052 | 0.083 | 0.532 | 0.293 | trans |
| CREB3L1 |  |  |  |  |  |  |  |  |  |  |
| rs1355538 | Diabetic polyneuropathy | 3 | 165505177 | A/G | 0.326 | -0.043 | 0.080 | 0.595 | 0.326 | trans |
| RRAS |  |  |  |  |  |  |  |  |  |  |
| rs72932837 | Diabetic polyneuropathy | 11 | 64876740 | T/C | 0.217 | -0.100 | 0.092 | 0.277 | 0.217 | trans |
| ZWILCH |  |  |  |  |  |  |  |  |  |  |
| rs2686395 | Diabetic polyneuropathy | 3 | 165485019 | T/C | 0.246 | -0.049 | 0.087 | 0.576 | 0.246 | trans |
| SATB2 |  |  |  |  |  |  |  |  |  |  |
| rs1707652 | Diabetic polyneuropathy | 3 | 165478799 | T/C | 0.248 | -0.037 | 0.087 | 0.672 | 0.248 | trans |
| rs6542680 | Diabetic polyneuropathy | 2 | 3640142 | C/T | 0.351 | -0.070 | 0.081 | 0.384 | 0.351 | trans |
| SLC3A2 |  |  |  |  |  |  |  |  |  |  |
| rs1355538 | Diabetic polyneuropathy | 3 | 165505177 | A/G | 0.326 | -0.043 | 0.080 | 0.595 | 0.326 | trans |
| FREM2 |  |  |  |  |  |  |  |  |  |  |
| rs1706435 | Diabetic polyneuropathy | 3 | 165461476 | G/A | 0.310 | -0.079 | 0.082 | 0.331 | 0.310 | trans |
| RAB35 |  |  |  |  |  |  |  |  |  |  |
| rs704 | Diabetic polyneuropathy | 17 | 26694861 | A/G | 0.420 | 0.049 | 0.076 | 0.519 | 0.420 | trans |
| MAD1L1 |  |  |  |  |  |  |  |  |  |  |
| rs704 | Diabetic polyneuropathy | 17 | 26694861 | A/G | 0.420 | 0.049 | 0.076 | 0.519 | 0.420 | trans |
| MSRB2 |  |  |  |  |  |  |  |  |  |  |
| rs3917538 | Diabetic polyneuropathy | 7 | 94937893 | A/G | 0.202 | -0.140 | 0.095 | 0.140 | 0.202 | trans |
| MYL6B |  |  |  |  |  |  |  |  |  |  |
| rs1355538 | Diabetic polyneuropathy | 3 | 165505177 | A/G | 0.326 | -0.043 | 0.080 | 0.595 | 0.326 | trans |
| rs115975731 | Diabetic polyneuropathy | 2 | 89131386 | A/G | 0.032 | -0.143 | 0.215 | 0.507 | 0.032 | trans |
| FOXG1 |  |  |  |  |  |  |  |  |  |  |
| rs1707652 | Diabetic polyneuropathy | 3 | 165478799 | T/C | 0.248 | -0.037 | 0.087 | 0.672 | 0.248 | trans |
| LYSMD3 |  |  |  |  |  |  |  |  |  |  |
| rs1355538 | Diabetic polyneuropathy | 3 | 165505177 | A/G | 0.326 | -0.043 | 0.080 | 0.595 | 0.326 | trans |
| AGGF1 |  |  |  |  |  |  |  |  |  |  |
| rs41290289 | Diabetic polyneuropathy | 10 | 7769806 | A/C | 0.937 | -0.092 | 0.155 | 0.555 | 0.063 | trans |
| PRC1 |  |  |  |  |  |  |  |  |  |  |
| rs1706435 | Diabetic polyneuropathy | 3 | 165461476 | G/A | 0.310 | -0.079 | 0.082 | 0.331 | 0.310 | trans |
| COLGALT2 |  |  |  |  |  |  |  |  |  |  |
| rs6542680 | Diabetic polyneuropathy | 2 | 3640142 | C/T | 0.351 | -0.070 | 0.081 | 0.384 | 0.351 | trans |
| PSMB6 |  |  |  |  |  |  |  |  |  |  |
| rs5754116 | Diabetic polyneuropathy | 22 | 32893920 | T/C | 0.345 | 0.007 | 0.080 | 0.934 | 0.345 | trans |
| SUFU |  |  |  |  |  |  |  |  |  |  |
| rs2686395 | Diabetic polyneuropathy | 3 | 165485019 | T/C | 0.246 | -0.049 | 0.087 | 0.576 | 0.246 | trans |
| PITPNA |  |  |  |  |  |  |  |  |  |  |
| rs11720167 | Diabetic polyneuropathy | 3 | 165486145 | G/T | 0.174 | -0.015 | 0.099 | 0.877 | 0.174 | trans |
| rs174533 | Diabetic polyneuropathy | 11 | 61549025 | A/G | 0.415 | -0.125 | 0.077 | 0.104 | 0.415 | trans |
| MAGEA8 |  |  |  |  |  |  |  |  |  |  |
| rs11720167 | Diabetic polyneuropathy | 3 | 165486145 | G/T | 0.174 | -0.015 | 0.099 | 0.877 | 0.174 | trans |
| CLTC |  |  |  |  |  |  |  |  |  |  |
| rs1706435 | Diabetic polyneuropathy | 3 | 165461476 | G/A | 0.310 | -0.079 | 0.082 | 0.331 | 0.310 | trans |
| PGK2 |  |  |  |  |  |  |  |  |  |  |
| rs62205397 | Diabetic polyneuropathy | 20 | 57597011 | T/C | 0.245 | -0.070 | 0.089 | 0.429 | 0.245 | trans |
| PYCR2 |  |  |  |  |  |  |  |  |  |  |
| rs7412 | Diabetic polyneuropathy | 19 | 45412079 | T/C | 0.053 | 0.166 | 0.164 | 0.311 | 0.053 | trans |
| ANKRD54 |  |  |  |  |  |  |  |  |  |  |
| rs1355538 | Diabetic polyneuropathy | 3 | 165505177 | A/G | 0.326 | -0.043 | 0.080 | 0.595 | 0.326 | trans |
| PLEKHA4 |  |  |  |  |  |  |  |  |  |  |
| rs2686395 | Diabetic polyneuropathy | 3 | 165485019 | T/C | 0.246 | -0.049 | 0.087 | 0.576 | 0.246 | trans |
| ADAM29 |  |  |  |  |  |  |  |  |  |  |
| rs1706435 | Diabetic polyneuropathy | 3 | 165461476 | G/A | 0.310 | -0.079 | 0.082 | 0.331 | 0.310 | trans |
| CLEC2A |  |  |  |  |  |  |  |  |  |  |
| rs7080536 | Diabetic polyneuropathy | 10 | 115348046 | A/G | 0.028 | 0.086 | 0.226 | 0.702 | 0.028 | trans |
| TNFRSF4 |  |  |  |  |  |  |  |  |  |  |
| rs6445035 | Diabetic polyneuropathy | 3 | 165480100 | A/G | 0.176 | 0.000 | 0.099 | 0.998 | 0.176 | trans |
| PSMD5 |  |  |  |  |  |  |  |  |  |  |
| rs2284350 | Diabetic polyneuropathy | 3 | 52822856 | C/T | 0.401 | -0.021 | 0.077 | 0.787 | 0.401 | trans |
| rs3917550 | Diabetic polyneuropathy | 7 | 94934573 | A/G | 0.150 | -0.144 | 0.108 | 0.180 | 0.150 | trans |
| LYPD1 |  |  |  |  |  |  |  |  |  |  |
| rs6445035 | Diabetic polyneuropathy | 3 | 165480100 | A/G | 0.176 | 0.000 | 0.099 | 0.998 | 0.176 | trans |
| PLIN3 |  |  |  |  |  |  |  |  |  |  |
| rs1355538 | Diabetic polyneuropathy | 3 | 165505177 | A/G | 0.326 | -0.043 | 0.080 | 0.595 | 0.326 | trans |
| IL36B |  |  |  |  |  |  |  |  |  |  |
| rs1355538 | Diabetic polyneuropathy | 3 | 165505177 | A/G | 0.326 | -0.043 | 0.080 | 0.595 | 0.326 | trans |
| AHSP |  |  |  |  |  |  |  |  |  |  |
| rs1707652 | Diabetic polyneuropathy | 3 | 165478799 | T/C | 0.248 | -0.037 | 0.087 | 0.672 | 0.248 | trans |
| ADGRG5 |  |  |  |  |  |  |  |  |  |  |
| rs1707652 | Diabetic polyneuropathy | 3 | 165478799 | T/C | 0.248 | -0.037 | 0.087 | 0.672 | 0.248 | trans |
| NPPA |  |  |  |  |  |  |  |  |  |  |
| rs6542680 | Diabetic polyneuropathy | 2 | 3640142 | C/T | 0.351 | -0.070 | 0.081 | 0.384 | 0.351 | trans |
| rs145488887 | Diabetic polyneuropathy | 1 | 11827796 | C/T | 0.094 | -0.116 | 0.131 | 0.376 | 0.094 | cis |
| MRPL34 |  |  |  |  |  |  |  |  |  |  |
| rs6542680 | Diabetic polyneuropathy | 2 | 3640142 | C/T | 0.351 | -0.070 | 0.081 | 0.384 | 0.351 | trans |
| EFNB2 |  |  |  |  |  |  |  |  |  |  |
| rs77924615 | Diabetic polyneuropathy | 16 | 20392332 | A/G | 0.222 | 0.042 | 0.091 | 0.643 | 0.222 | trans |
| rs72704449 | Diabetic polyneuropathy | 1 | 179473273 | C/T | 0.065 | -0.202 | 0.157 | 0.197 | 0.065 | trans |
| GAL3ST2 |  |  |  |  |  |  |  |  |  |  |
| rs2686395 | Diabetic polyneuropathy | 3 | 165485019 | T/C | 0.246 | -0.049 | 0.087 | 0.576 | 0.246 | trans |
| LRIG3 |  |  |  |  |  |  |  |  |  |  |
| rs11172791 | Diabetic polyneuropathy | 12 | 59272973 | T/C | 0.962 | -0.074 | 0.194 | 0.702 | 0.038 | cis |
| C4orf32 |  |  |  |  |  |  |  |  |  |  |
| rs17713196 | Diabetic polyneuropathy | 3 | 165489724 | T/C | 0.174 | -0.015 | 0.099 | 0.878 | 0.174 | trans |
| ZNF10 |  |  |  |  |  |  |  |  |  |  |
| rs6542680 | Diabetic polyneuropathy | 2 | 3640142 | C/T | 0.351 | -0.070 | 0.081 | 0.384 | 0.351 | trans |
| SERAC1 |  |  |  |  |  |  |  |  |  |  |
| rs704 | Diabetic polyneuropathy | 17 | 26694861 | A/G | 0.420 | 0.049 | 0.076 | 0.519 | 0.420 | trans |
| MTPAP |  |  |  |  |  |  |  |  |  |  |
| rs1803274 | Diabetic polyneuropathy | 3 | 165491280 | T/C | 0.174 | -0.016 | 0.099 | 0.869 | 0.174 | trans |
| ZNF264 |  |  |  |  |  |  |  |  |  |  |
| rs1355538 | Diabetic polyneuropathy | 3 | 165505177 | A/G | 0.326 | -0.043 | 0.080 | 0.595 | 0.326 | trans |
| TK1 |  |  |  |  |  |  |  |  |  |  |
| rs1707652 | Diabetic polyneuropathy | 3 | 165478799 | T/C | 0.248 | -0.037 | 0.087 | 0.672 | 0.248 | trans |
| rs11569415 | Diabetic polyneuropathy | 19 | 6716279 | A/G | 0.185 | -0.101 | 0.098 | 0.304 | 0.185 | cis |
| GMNN |  |  |  |  |  |  |  |  |  |  |
| rs1355538 | Diabetic polyneuropathy | 3 | 165505177 | A/G | 0.326 | -0.043 | 0.080 | 0.595 | 0.326 | trans |
| FSTL5 |  |  |  |  |  |  |  |  |  |  |
| rs1355538 | Diabetic polyneuropathy | 3 | 165505177 | A/G | 0.326 | -0.043 | 0.080 | 0.595 | 0.326 | trans |
| NNAT |  |  |  |  |  |  |  |  |  |  |
| rs1707652 | Diabetic polyneuropathy | 3 | 165478799 | T/C | 0.248 | -0.037 | 0.087 | 0.672 | 0.248 | trans |
| STAT5B |  |  |  |  |  |  |  |  |  |  |
| rs11720167 | Diabetic polyneuropathy | 3 | 165486145 | G/T | 0.174 | -0.015 | 0.099 | 0.877 | 0.174 | trans |
| TJP1 |  |  |  |  |  |  |  |  |  |  |
| rs1707652 | Diabetic polyneuropathy | 3 | 165478799 | T/C | 0.248 | -0.037 | 0.087 | 0.672 | 0.248 | trans |
| NAALADL1 |  |  |  |  |  |  |  |  |  |  |
| rs704 | Diabetic polyneuropathy | 17 | 26694861 | A/G | 0.420 | 0.049 | 0.076 | 0.519 | 0.420 | trans |
| NLRP4 |  |  |  |  |  |  |  |  |  |  |
| rs6542680 | Diabetic polyneuropathy | 2 | 3640142 | C/T | 0.351 | -0.070 | 0.081 | 0.384 | 0.351 | trans |
| LRP2 |  |  |  |  |  |  |  |  |  |  |
| rs1706435 | Diabetic polyneuropathy | 3 | 165461476 | G/A | 0.310 | -0.079 | 0.082 | 0.331 | 0.310 | trans |
| ZPBP |  |  |  |  |  |  |  |  |  |  |
| rs1707652 | Diabetic polyneuropathy | 3 | 165478799 | T/C | 0.248 | -0.037 | 0.087 | 0.672 | 0.248 | trans |
| SUDS3 |  |  |  |  |  |  |  |  |  |  |
| rs11569415 | Diabetic polyneuropathy | 19 | 6716279 | A/G | 0.185 | -0.101 | 0.098 | 0.304 | 0.185 | cis |
| ZNF276 |  |  |  |  |  |  |  |  |  |  |
| rs1707652 | Diabetic polyneuropathy | 3 | 165478799 | T/C | 0.248 | -0.037 | 0.087 | 0.672 | 0.248 | trans |
| KCNIP4 |  |  |  |  |  |  |  |  |  |  |
| rs1355537 | Diabetic polyneuropathy | 3 | 165505274 | A/G | 0.246 | -0.051 | 0.087 | 0.556 | 0.246 | trans |
| INHBA_INHBC |  |  |  |  |  |  |  |  |  |  |
| rs3741414 | Diabetic polyneuropathy | 12 | 57844049 | T/C | 0.233 | 0.249 | 0.089 | 0.005 | 0.233 | trans |
| MTMR1 |  |  |  |  |  |  |  |  |  |  |
| rs1707652 | Diabetic polyneuropathy | 3 | 165478799 | T/C | 0.248 | -0.037 | 0.087 | 0.672 | 0.248 | trans |
| LNX1 |  |  |  |  |  |  |  |  |  |  |
| rs2686395 | Diabetic polyneuropathy | 3 | 165485019 | T/C | 0.246 | -0.049 | 0.087 | 0.576 | 0.246 | trans |
| ISL1 |  |  |  |  |  |  |  |  |  |  |
| rs77938199 | Diabetic polyneuropathy | 10 | 7742467 | G/A | 0.100 | 0.064 | 0.128 | 0.614 | 0.100 | trans |
| rs2300149 | Diabetic polyneuropathy | 3 | 52822921 | T/C | 0.401 | -0.021 | 0.077 | 0.787 | 0.401 | trans |
| GALNT11 |  |  |  |  |  |  |  |  |  |  |
| rs1707652 | Diabetic polyneuropathy | 3 | 165478799 | T/C | 0.248 | -0.037 | 0.087 | 0.672 | 0.248 | trans |
| WRNIP1 |  |  |  |  |  |  |  |  |  |  |
| rs1706435 | Diabetic polyneuropathy | 3 | 165461476 | G/A | 0.310 | -0.079 | 0.082 | 0.331 | 0.310 | trans |
| NAE1 |  |  |  |  |  |  |  |  |  |  |
| rs11720167 | Diabetic polyneuropathy | 3 | 165486145 | G/T | 0.174 | -0.015 | 0.099 | 0.877 | 0.174 | trans |
| TINF2 |  |  |  |  |  |  |  |  |  |  |
| rs4566588 | Diabetic polyneuropathy | 3 | 165462568 | T/C | 0.243 | -0.040 | 0.088 | 0.650 | 0.243 | trans |
| NFATC4 |  |  |  |  |  |  |  |  |  |  |
| rs11720167 | Diabetic polyneuropathy | 3 | 165486145 | G/T | 0.174 | -0.015 | 0.099 | 0.877 | 0.174 | trans |
| YES1 |  |  |  |  |  |  |  |  |  |  |
| rs1354034 | Diabetic polyneuropathy | 3 | 56849749 | C/T | 0.707 | 0.052 | 0.083 | 0.532 | 0.293 | trans |
| CNTNAP5 |  |  |  |  |  |  |  |  |  |  |
| rs1707652 | Diabetic polyneuropathy | 3 | 165478799 | T/C | 0.248 | -0.037 | 0.087 | 0.672 | 0.248 | trans |
| NEDD9 |  |  |  |  |  |  |  |  |  |  |
| rs1355538 | Diabetic polyneuropathy | 3 | 165505177 | A/G | 0.326 | -0.043 | 0.080 | 0.595 | 0.326 | trans |
| CNEP1R1 |  |  |  |  |  |  |  |  |  |  |
| rs1355538 | Diabetic polyneuropathy | 3 | 165505177 | A/G | 0.326 | -0.043 | 0.080 | 0.595 | 0.326 | trans |
| NDNF |  |  |  |  |  |  |  |  |  |  |
| rs1355538 | Diabetic polyneuropathy | 3 | 165505177 | A/G | 0.326 | -0.043 | 0.080 | 0.595 | 0.326 | trans |
| YWHAE |  |  |  |  |  |  |  |  |  |  |
| rs1355538 | Diabetic polyneuropathy | 3 | 165505177 | A/G | 0.326 | -0.043 | 0.080 | 0.595 | 0.326 | trans |
| DEFB118 |  |  |  |  |  |  |  |  |  |  |
| rs6542680 | Diabetic polyneuropathy | 2 | 3640142 | C/T | 0.351 | -0.070 | 0.081 | 0.384 | 0.351 | trans |
| PDZK1 |  |  |  |  |  |  |  |  |  |  |
| rs1707652 | Diabetic polyneuropathy | 3 | 165478799 | T/C | 0.248 | -0.037 | 0.087 | 0.672 | 0.248 | trans |
| TIGIT |  |  |  |  |  |  |  |  |  |  |
| rs7412 | Diabetic polyneuropathy | 19 | 45412079 | T/C | 0.053 | 0.166 | 0.164 | 0.311 | 0.053 | trans |
| rs1707652 | Diabetic polyneuropathy | 3 | 165478799 | T/C | 0.248 | -0.037 | 0.087 | 0.672 | 0.248 | trans |
| rs73238029 | Diabetic polyneuropathy | 3 | 114014080 | C/A | 0.216 | -0.247 | 0.092 | 0.007 | 0.216 | cis |
| FGFR3 |  |  |  |  |  |  |  |  |  |  |
| rs17329885 | Diabetic polyneuropathy | 12 | 21328565 | C/T | 0.112 | 0.187 | 0.119 | 0.117 | 0.112 | trans |
| rs1355538 | Diabetic polyneuropathy | 3 | 165505177 | A/G | 0.326 | -0.043 | 0.080 | 0.595 | 0.326 | trans |
| rs72704449 | Diabetic polyneuropathy | 1 | 179473273 | C/T | 0.065 | -0.202 | 0.157 | 0.197 | 0.065 | trans |
| NEFH |  |  |  |  |  |  |  |  |  |  |
| rs2686395 | Diabetic polyneuropathy | 3 | 165485019 | T/C | 0.246 | -0.049 | 0.087 | 0.576 | 0.246 | trans |
| NCR2 |  |  |  |  |  |  |  |  |  |  |
| rs1355538 | Diabetic polyneuropathy | 3 | 165505177 | A/G | 0.326 | -0.043 | 0.080 | 0.595 | 0.326 | trans |
| DDR2 |  |  |  |  |  |  |  |  |  |  |
| rs2107432 | Diabetic polyneuropathy | 17 | 55043036 | A/G | 0.340 | 0.040 | 0.080 | 0.616 | 0.340 | trans |
| PGLYRP3 |  |  |  |  |  |  |  |  |  |  |
| rs1865234 | Diabetic polyneuropathy | 1 | 153315784 | T/C | 0.088 | -0.016 | 0.133 | 0.904 | 0.088 | cis |
| NAGS |  |  |  |  |  |  |  |  |  |  |
| rs6445035 | Diabetic polyneuropathy | 3 | 165480100 | A/G | 0.176 | 0.000 | 0.099 | 0.998 | 0.176 | trans |
| CNTF |  |  |  |  |  |  |  |  |  |  |
| rs1355538 | Diabetic polyneuropathy | 3 | 165505177 | A/G | 0.326 | -0.043 | 0.080 | 0.595 | 0.326 | trans |
| ZNF180 |  |  |  |  |  |  |  |  |  |  |
| rs893522 | Diabetic polyneuropathy | 3 | 165560618 | A/C | 0.918 | -0.111 | 0.137 | 0.419 | 0.082 | trans |
| KCTD5 |  |  |  |  |  |  |  |  |  |  |
| rs2686395 | Diabetic polyneuropathy | 3 | 165485019 | T/C | 0.246 | -0.049 | 0.087 | 0.576 | 0.246 | trans |
| NETO1 |  |  |  |  |  |  |  |  |  |  |
| rs6445035 | Diabetic polyneuropathy | 3 | 165480100 | A/G | 0.176 | 0.000 | 0.099 | 0.998 | 0.176 | trans |
| JPH4 |  |  |  |  |  |  |  |  |  |  |
| rs28362944 | Diabetic polyneuropathy | 11 | 57365723 | C/T | 0.054 | -0.353 | 0.169 | 0.037 | 0.054 | trans |
| GALNT13 |  |  |  |  |  |  |  |  |  |  |
| rs12975366 | Diabetic polyneuropathy | 19 | 54759361 | C/T | 0.372 | 0.142 | 0.079 | 0.071 | 0.372 | trans |
| rs16834610 | Diabetic polyneuropathy | 2 | 154730495 | T/C | 0.032 | -0.321 | 0.210 | 0.126 | 0.032 | cis |
| INHBB |  |  |  |  |  |  |  |  |  |  |
| rs3741414 | Diabetic polyneuropathy | 12 | 57844049 | T/C | 0.233 | 0.249 | 0.089 | 0.005 | 0.233 | trans |
| rs28929474 | Diabetic polyneuropathy | 14 | 94844947 | T/C | 0.020 | -0.002 | 0.272 | 0.993 | 0.020 | trans |
| rs4738679 | Diabetic polyneuropathy | 8 | 59370320 | G/A | 0.385 | -0.069 | 0.078 | 0.374 | 0.385 | trans |
| rs1706435 | Diabetic polyneuropathy | 3 | 165461476 | G/A | 0.310 | -0.079 | 0.082 | 0.331 | 0.310 | trans |
| PFKFB3 |  |  |  |  |  |  |  |  |  |  |
| rs704 | Diabetic polyneuropathy | 17 | 26694861 | A/G | 0.420 | 0.049 | 0.076 | 0.519 | 0.420 | trans |
| TMEM52 |  |  |  |  |  |  |  |  |  |  |
| rs8178824 | Diabetic polyneuropathy | 17 | 64224775 | T/C | 0.009 | -0.140 | 0.389 | 0.718 | 0.009 | trans |
| PSMA1 |  |  |  |  |  |  |  |  |  |  |
| rs704 | Diabetic polyneuropathy | 17 | 26694861 | A/G | 0.420 | 0.049 | 0.076 | 0.519 | 0.420 | trans |
| AK4 |  |  |  |  |  |  |  |  |  |  |
| rs1950641 | Diabetic polyneuropathy | 14 | 94750292 | A/G | 0.192 | -0.152 | 0.097 | 0.116 | 0.192 | trans |
| PITPNB |  |  |  |  |  |  |  |  |  |  |
| rs17713196 | Diabetic polyneuropathy | 3 | 165489724 | T/C | 0.174 | -0.015 | 0.099 | 0.878 | 0.174 | trans |
| CLIC5 |  |  |  |  |  |  |  |  |  |  |
| rs35822882 | Diabetic polyneuropathy | 6 | 45916999 | T/G | 0.009 | -0.528 | 0.413 | 0.201 | 0.009 | cis |
| HAT1 |  |  |  |  |  |  |  |  |  |  |
| rs78119247 | Diabetic polyneuropathy | 3 | 165499135 | G/GAT | 0.173 | -0.017 | 0.100 | 0.866 | 0.173 | trans |
| SIRPG |  |  |  |  |  |  |  |  |  |  |
| rs6043409 | Diabetic polyneuropathy | 20 | 1616206 | A/G | 0.305 | 0.061 | 0.082 | 0.455 | 0.305 | cis |
| MCL1 |  |  |  |  |  |  |  |  |  |  |
| rs190596489 | Diabetic polyneuropathy | 1 | 150562043 | A/C | 0.993 | -0.477 | 0.432 | 0.270 | 0.007 | cis |
| DNAJB12 |  |  |  |  |  |  |  |  |  |  |
| rs6987853 | Diabetic polyneuropathy | 8 | 42457450 | T/C | 0.350 | -0.035 | 0.080 | 0.660 | 0.350 | trans |
| CSF3R |  |  |  |  |  |  |  |  |  |  |
| rs1707652 | Diabetic polyneuropathy | 3 | 165478799 | T/C | 0.248 | -0.037 | 0.087 | 0.672 | 0.248 | trans |
| rs6542680 | Diabetic polyneuropathy | 2 | 3640142 | C/T | 0.351 | -0.070 | 0.081 | 0.384 | 0.351 | trans |
| HSPA8 |  |  |  |  |  |  |  |  |  |  |
| rs1354034 | Diabetic polyneuropathy | 3 | 56849749 | C/T | 0.707 | 0.052 | 0.083 | 0.532 | 0.293 | trans |
| SF3B4 |  |  |  |  |  |  |  |  |  |  |
| rs1354034 | Diabetic polyneuropathy | 3 | 56849749 | C/T | 0.707 | 0.052 | 0.083 | 0.532 | 0.293 | trans |
| rs72692816 | Diabetic polyneuropathy | 1 | 149902588 | A/G | 0.944 | -0.319 | 0.165 | 0.054 | 0.056 | cis |
| RPS6KB1 |  |  |  |  |  |  |  |  |  |  |
| rs1354034 | Diabetic polyneuropathy | 3 | 56849749 | C/T | 0.707 | 0.052 | 0.083 | 0.532 | 0.293 | trans |
| rs919791 | Diabetic polyneuropathy | 19 | 15576817 | A/G | 0.207 | -0.048 | 0.093 | 0.602 | 0.207 | trans |
| BMPR1B |  |  |  |  |  |  |  |  |  |  |
| rs704 | Diabetic polyneuropathy | 17 | 26694861 | A/G | 0.420 | 0.049 | 0.076 | 0.519 | 0.420 | trans |
| RERG |  |  |  |  |  |  |  |  |  |  |
| rs704 | Diabetic polyneuropathy | 17 | 26694861 | A/G | 0.420 | 0.049 | 0.076 | 0.519 | 0.420 | trans |
| PRSS35 |  |  |  |  |  |  |  |  |  |  |
| rs4545169 | Diabetic polyneuropathy | 9 | 137781941 | T/G | 0.891 | 0.100 | 0.121 | 0.407 | 0.109 | trans |
| HSD17B11 |  |  |  |  |  |  |  |  |  |  |
| rs6542680 | Diabetic polyneuropathy | 2 | 3640142 | C/T | 0.351 | -0.070 | 0.081 | 0.384 | 0.351 | trans |
| PHGDH |  |  |  |  |  |  |  |  |  |  |
| rs6445035 | Diabetic polyneuropathy | 3 | 165480100 | A/G | 0.176 | 0.000 | 0.099 | 0.998 | 0.176 | trans |
| rs1535 | Diabetic polyneuropathy | 11 | 61597972 | G/A | 0.414 | -0.124 | 0.077 | 0.105 | 0.414 | trans |
| rs429358 | Diabetic polyneuropathy | 19 | 45411941 | C/T | 0.183 | -0.237 | 0.100 | 0.018 | 0.183 | trans |
| APOM |  |  |  |  |  |  |  |  |  |  |
| rs1260326 | Diabetic polyneuropathy | 2 | 27730940 | T/C | 0.351 | -0.100 | 0.079 | 0.208 | 0.351 | trans |
| CRABP2 |  |  |  |  |  |  |  |  |  |  |
| rs3806412 | Diabetic polyneuropathy | 1 | 156676553 | T/G | 0.652 | 0.035 | 0.081 | 0.667 | 0.348 | cis |
| BPIFA1 |  |  |  |  |  |  |  |  |  |  |
| rs1619994 | Diabetic polyneuropathy | 3 | 165461835 | G/A | 0.252 | -0.063 | 0.087 | 0.470 | 0.252 | trans |
| SYTL1 |  |  |  |  |  |  |  |  |  |  |
| rs704 | Diabetic polyneuropathy | 17 | 26694861 | A/G | 0.420 | 0.049 | 0.076 | 0.519 | 0.420 | trans |
| TSR3 |  |  |  |  |  |  |  |  |  |  |
| rs829502 | Diabetic polyneuropathy | 3 | 165499257 | A/G | 0.246 | -0.051 | 0.087 | 0.564 | 0.246 | trans |
| PSMB3 |  |  |  |  |  |  |  |  |  |  |
| rs10924845 | Diabetic polyneuropathy | 1 | 246991094 | T/C | 0.726 | -0.099 | 0.085 | 0.248 | 0.274 | trans |
| OXT |  |  |  |  |  |  |  |  |  |  |
| rs1800961 | Diabetic polyneuropathy | 20 | 43042364 | T/C | 0.045 | 0.445 | 0.180 | 0.013 | 0.045 | trans |
| rs10929757 | Diabetic polyneuropathy | 2 | 11702661 | C/A | 0.662 | 0.132 | 0.080 | 0.099 | 0.338 | trans |
| rs11127048 | Diabetic polyneuropathy | 2 | 27752463 | A/G | 0.626 | 0.065 | 0.079 | 0.406 | 0.374 | trans |
| rs877172 | Diabetic polyneuropathy | 20 | 3049890 | G/T | 0.312 | 0.061 | 0.084 | 0.466 | 0.312 | cis |
| rs35332062 | Diabetic polyneuropathy | 7 | 73012042 | A/G | 0.129 | 0.003 | 0.112 | 0.980 | 0.129 | trans |
| rs112875651 | Diabetic polyneuropathy | 8 | 126506694 | A/G | 0.387 | -0.058 | 0.078 | 0.456 | 0.387 | trans |
| rs4895864 | Diabetic polyneuropathy | 6 | 130364482 | T/C | 0.601 | -0.170 | 0.077 | 0.027 | 0.399 | trans |
| rs45446698 | Diabetic polyneuropathy | 7 | 99332948 | G/T | 0.052 | -0.288 | 0.171 | 0.092 | 0.052 | trans |
| FAM171B |  |  |  |  |  |  |  |  |  |  |
| rs10931256 | Diabetic polyneuropathy | 2 | 187685195 | C/T | 0.245 | -0.117 | 0.087 | 0.180 | 0.245 | cis |
| TIE1 |  |  |  |  |  |  |  |  |  |  |
| rs8176672 | Diabetic polyneuropathy | 9 | 136142185 | T/C | 0.134 | 0.135 | 0.111 | 0.223 | 0.134 | trans |
| rs3120276 | Diabetic polyneuropathy | 1 | 43779564 | T/C | 0.334 | 0.114 | 0.080 | 0.156 | 0.334 | cis |
| rs878381 | Diabetic polyneuropathy | 16 | 138032 | T/C | 0.772 | -0.033 | 0.091 | 0.718 | 0.228 | trans |
| ICAM2 |  |  |  |  |  |  |  |  |  |  |
| rs2519093 | Diabetic polyneuropathy | 9 | 136141870 | T/C | 0.201 | 0.028 | 0.094 | 0.762 | 0.201 | trans |
| rs2837989 | Diabetic polyneuropathy | 21 | 42620119 | A/C | 0.158 | -0.007 | 0.103 | 0.948 | 0.158 | trans |
| rs111626763 | Diabetic polyneuropathy | 12 | 26834804 | TACTC/T | 0.640 | -0.036 | 0.079 | 0.645 | 0.360 | trans |
| rs9399137 | Diabetic polyneuropathy | 6 | 135419018 | C/T | 0.340 | -0.092 | 0.080 | 0.248 | 0.340 | trans |
| MICB_MICA |  |  |  |  |  |  |  |  |  |  |
| rs2009581 | Diabetic polyneuropathy | 2 | 111807677 | A/G | 0.264 | 0.088 | 0.086 | 0.302 | 0.264 | trans |
| rs4760 | Diabetic polyneuropathy | 19 | 44153100 | G/A | 0.168 | 0.087 | 0.103 | 0.396 | 0.168 | trans |
| rs3184504 | Diabetic polyneuropathy | 12 | 111884608 | C/T | 0.591 | -0.051 | 0.077 | 0.504 | 0.409 | trans |
| PLTP |  |  |  |  |  |  |  |  |  |  |
| rs6073958 | Diabetic polyneuropathy | 20 | 44551855 | C/T | 0.176 | 0.002 | 0.099 | 0.985 | 0.176 | trans |
| IL4R |  |  |  |  |  |  |  |  |  |  |
| rs8060025 | Diabetic polyneuropathy | 16 | 27327214 | G/T | 0.653 | 0.130 | 0.079 | 0.099 | 0.347 | cis |
| rs2519093 | Diabetic polyneuropathy | 9 | 136141870 | T/C | 0.201 | 0.028 | 0.094 | 0.762 | 0.201 | trans |
| rs28929474 | Diabetic polyneuropathy | 14 | 94844947 | T/C | 0.020 | -0.002 | 0.272 | 0.993 | 0.020 | trans |
| PDCD1LG2 |  |  |  |  |  |  |  |  |  |  |
| rs2070725 | Diabetic polyneuropathy | 5 | 131821788 | T/C | 0.330 | 0.095 | 0.081 | 0.237 | 0.330 | trans |
| rs507666 | Diabetic polyneuropathy | 9 | 136149399 | A/G | 0.201 | 0.019 | 0.094 | 0.840 | 0.201 | trans |
| rs4055121 | Diabetic polyneuropathy | 11 | 126232337 | T/C | 0.153 | -0.048 | 0.106 | 0.651 | 0.153 | trans |
| rs3184504 | Diabetic polyneuropathy | 12 | 111884608 | C/T | 0.591 | -0.051 | 0.077 | 0.504 | 0.409 | trans |
| SCARB2 |  |  |  |  |  |  |  |  |  |  |
| rs28563976 | Diabetic polyneuropathy | 4 | 77097373 | C/A | 0.136 | 0.070 | 0.110 | 0.524 | 0.136 | cis |
| rs13192569 | Diabetic polyneuropathy | 6 | 123127597 | A/G | 0.180 | -0.013 | 0.099 | 0.898 | 0.180 | trans |
| rs62018815 | Diabetic polyneuropathy | 15 | 51058854 | A/G | 0.013 | -0.165 | 0.325 | 0.611 | 0.013 | trans |
| IL1R1 |  |  |  |  |  |  |  |  |  |  |
| rs8176672 | Diabetic polyneuropathy | 9 | 136142185 | T/C | 0.134 | 0.135 | 0.111 | 0.223 | 0.134 | trans |
| rs9987289 | Diabetic polyneuropathy | 8 | 9183358 | G/A | 0.857 | 0.118 | 0.116 | 0.310 | 0.143 | trans |
| rs3917238 | Diabetic polyneuropathy | 2 | 102773083 | T/C | 0.279 | 0.018 | 0.085 | 0.832 | 0.279 | cis |
| rs55709272 | Diabetic polyneuropathy | 2 | 113867288 | C/T | 0.323 | 0.002 | 0.081 | 0.985 | 0.323 | trans |
| rs28929474 | Diabetic polyneuropathy | 14 | 94844947 | T/C | 0.020 | -0.002 | 0.272 | 0.993 | 0.020 | trans |
| rs56278466 | Diabetic polyneuropathy | 10 | 17875857 | G/T | 0.581 | -0.003 | 0.077 | 0.970 | 0.419 | trans |
| rs6545929 | Diabetic polyneuropathy | 2 | 62610179 | A/G | 0.124 | -0.059 | 0.113 | 0.601 | 0.124 | trans |
| rs61830291 | Diabetic polyneuropathy | 1 | 221001142 | A/C | 0.885 | -0.118 | 0.119 | 0.320 | 0.115 | trans |
| IL6ST |  |  |  |  |  |  |  |  |  |  |
| rs77542162 | Diabetic polyneuropathy | 17 | 67081278 | A/G | 0.993 | 0.407 | 0.465 | 0.382 | 0.007 | trans |
| rs507666 | Diabetic polyneuropathy | 9 | 136149399 | A/G | 0.201 | 0.019 | 0.094 | 0.840 | 0.201 | trans |
| rs2008174 | Diabetic polyneuropathy | 22 | 39860130 | C/T | 0.295 | 0.001 | 0.084 | 0.991 | 0.295 | trans |
| rs3791101 | Diabetic polyneuropathy | 1 | 44366250 | A/G | 0.304 | -0.021 | 0.083 | 0.796 | 0.304 | trans |
| rs4721064 | Diabetic polyneuropathy | 7 | 12284378 | A/G | 0.330 | -0.025 | 0.080 | 0.758 | 0.330 | trans |
| rs3967200 | Diabetic polyneuropathy | 11 | 126232385 | T/C | 0.153 | -0.048 | 0.106 | 0.651 | 0.153 | trans |
| rs7145500 | Diabetic polyneuropathy | 14 | 65884212 | A/G | 0.610 | -0.072 | 0.077 | 0.351 | 0.390 | trans |
| rs11739016 | Diabetic polyneuropathy | 5 | 55263373 | T/C | 0.121 | -0.088 | 0.117 | 0.452 | 0.121 | cis |
| TYRO3 |  |  |  |  |  |  |  |  |  |  |
| rs13107325 | Diabetic polyneuropathy | 4 | 103188709 | T/C | 0.014 | 0.106 | 0.309 | 0.731 | 0.014 | trans |
| rs2008174 | Diabetic polyneuropathy | 22 | 39860130 | C/T | 0.295 | 0.001 | 0.084 | 0.991 | 0.295 | trans |
| rs3791101 | Diabetic polyneuropathy | 1 | 44366250 | A/G | 0.304 | -0.021 | 0.083 | 0.796 | 0.304 | trans |
| rs3967200 | Diabetic polyneuropathy | 11 | 126232385 | T/C | 0.153 | -0.048 | 0.106 | 0.651 | 0.153 | trans |
| rs8024626 | Diabetic polyneuropathy | 15 | 41863181 | A/G | 0.314 | -0.051 | 0.082 | 0.531 | 0.314 | cis |
| ROBO1 |  |  |  |  |  |  |  |  |  |  |
| rs77542162 | Diabetic polyneuropathy | 17 | 67081278 | A/G | 0.993 | 0.407 | 0.465 | 0.382 | 0.007 | trans |
| rs9987289 | Diabetic polyneuropathy | 8 | 9183358 | G/A | 0.857 | 0.118 | 0.116 | 0.310 | 0.143 | trans |
| rs3773244 | Diabetic polyneuropathy | 3 | 78784770 | A/G | 0.244 | -0.002 | 0.088 | 0.985 | 0.244 | cis |
| rs56278466 | Diabetic polyneuropathy | 10 | 17875857 | G/T | 0.581 | -0.003 | 0.077 | 0.970 | 0.419 | trans |
| rs3849768 | Diabetic polyneuropathy | 5 | 39418887 | A/C | 0.197 | -0.021 | 0.095 | 0.825 | 0.197 | trans |
| FLT4 |  |  |  |  |  |  |  |  |  |  |
| rs651007 | Diabetic polyneuropathy | 9 | 136153875 | T/C | 0.222 | -0.046 | 0.091 | 0.612 | 0.222 | trans |
| EBI3 |  |  |  |  |  |  |  |  |  |  |
| rs62165726 | Diabetic polyneuropathy | 2 | 134966562 | A/C | 0.026 | 0.074 | 0.243 | 0.760 | 0.026 | trans |
| rs10937241 | Diabetic polyneuropathy | 3 | 185822774 | A/G | 0.172 | 0.053 | 0.100 | 0.600 | 0.172 | trans |
| rs704 | Diabetic polyneuropathy | 17 | 26694861 | A/G | 0.420 | 0.049 | 0.076 | 0.519 | 0.420 | trans |
| rs5743618 | Diabetic polyneuropathy | 4 | 38798648 | A/C | 0.153 | 0.024 | 0.104 | 0.820 | 0.153 | trans |
| rs11711157 | Diabetic polyneuropathy | 3 | 194061826 | T/C | 0.350 | -0.051 | 0.079 | 0.524 | 0.350 | trans |
| rs3184504 | Diabetic polyneuropathy | 12 | 111884608 | C/T | 0.591 | -0.051 | 0.077 | 0.504 | 0.409 | trans |
| rs61751507 | Diabetic polyneuropathy | 10 | 101829514 | T/C | 0.038 | -0.214 | 0.196 | 0.274 | 0.038 | trans |
| CERT |  |  |  |  |  |  |  |  |  |  |
| rs1354034 | Diabetic polyneuropathy | 3 | 56849749 | C/T | 0.707 | 0.052 | 0.083 | 0.532 | 0.293 | trans |
| rs10942737 | Diabetic polyneuropathy | 5 | 74682813 | C/T | 0.069 | -0.081 | 0.147 | 0.583 | 0.069 | cis |
| SIAE |  |  |  |  |  |  |  |  |  |  |
| rs11602163 | Diabetic polyneuropathy | 11 | 87909193 | A/G | 0.773 | 0.086 | 0.090 | 0.341 | 0.227 | trans |
| rs10968020 | Diabetic polyneuropathy | 9 | 27628440 | C/T | 0.246 | 0.024 | 0.088 | 0.783 | 0.246 | trans |
| rs2296436 | Diabetic polyneuropathy | 10 | 100179851 | T/C | 0.870 | 0.006 | 0.112 | 0.960 | 0.130 | trans |
| rs12191772 | Diabetic polyneuropathy | 6 | 137259725 | A/G | 0.220 | -0.124 | 0.092 | 0.177 | 0.220 | trans |
| rs78778622 | Diabetic polyneuropathy | 11 | 124530664 | T/C | 0.936 | -0.258 | 0.157 | 0.101 | 0.064 | cis |
| rs72701845 | Diabetic polyneuropathy | 14 | 93217023 | A/G | 0.039 | -0.358 | 0.194 | 0.065 | 0.039 | trans |
| MYORG |  |  |  |  |  |  |  |  |  |  |
| rs10972076 | Diabetic polyneuropathy | 9 | 34356359 | T/C | 0.583 | 0.043 | 0.077 | 0.578 | 0.417 | cis |
| CNTFR |  |  |  |  |  |  |  |  |  |  |
| rs2008174 | Diabetic polyneuropathy | 22 | 39860130 | C/T | 0.295 | 0.001 | 0.084 | 0.991 | 0.295 | trans |
| rs10972159 | Diabetic polyneuropathy | 9 | 34593086 | A/G | 0.052 | -0.044 | 0.175 | 0.801 | 0.052 | cis |
| rs72704449 | Diabetic polyneuropathy | 1 | 179473273 | C/T | 0.065 | -0.202 | 0.157 | 0.197 | 0.065 | trans |
| GPX7 |  |  |  |  |  |  |  |  |  |  |
| rs1354034 | Diabetic polyneuropathy | 3 | 56849749 | C/T | 0.707 | 0.052 | 0.083 | 0.532 | 0.293 | trans |
| rs1097234 | Diabetic polyneuropathy | 1 | 53063559 | A/C | 0.151 | -0.128 | 0.105 | 0.225 | 0.151 | cis |
| SIRT3 |  |  |  |  |  |  |  |  |  |  |
| rs2035675 | Diabetic polyneuropathy | 11 | 6463088 | A/G | 0.740 | 0.120 | 0.086 | 0.162 | 0.260 | trans |
| rs11604127 | Diabetic polyneuropathy | 11 | 196944 | T/C | 0.296 | -0.004 | 0.084 | 0.963 | 0.296 | cis |
| rs10981377 | Diabetic polyneuropathy | 9 | 115144889 | T/C | 0.285 | -0.204 | 0.084 | 0.015 | 0.285 | trans |
| TGFBI |  |  |  |  |  |  |  |  |  |  |
| rs12975366 | Diabetic polyneuropathy | 19 | 54759361 | C/T | 0.372 | 0.142 | 0.079 | 0.071 | 0.372 | trans |
| rs11127048 | Diabetic polyneuropathy | 2 | 27752463 | A/G | 0.626 | 0.065 | 0.079 | 0.406 | 0.374 | trans |
| rs28929474 | Diabetic polyneuropathy | 14 | 94844947 | T/C | 0.020 | -0.002 | 0.272 | 0.993 | 0.020 | trans |
| rs56278466 | Diabetic polyneuropathy | 10 | 17875857 | G/T | 0.581 | -0.003 | 0.077 | 0.970 | 0.419 | trans |
| GFRA1 |  |  |  |  |  |  |  |  |  |  |
| rs11197603 | Diabetic polyneuropathy | 10 | 118001771 | C/T | 0.185 | 0.070 | 0.098 | 0.476 | 0.185 | cis |
| rs34262842 | Diabetic polyneuropathy | 16 | 20355811 | G/A | 0.219 | 0.022 | 0.091 | 0.814 | 0.219 | trans |
| rs6041 | Diabetic polyneuropathy | 13 | 113772707 | A/G | 0.078 | -0.087 | 0.141 | 0.538 | 0.078 | trans |
| F10 |  |  |  |  |  |  |  |  |  |  |
| rs4665972 | Diabetic polyneuropathy | 2 | 27598097 | C/T | 0.623 | 0.080 | 0.078 | 0.303 | 0.377 | trans |
| rs116994374 | Diabetic polyneuropathy | 9 | 117084672 | A/G | 0.026 | 0.038 | 0.244 | 0.876 | 0.026 | trans |
| MLN |  |  |  |  |  |  |  |  |  |  |
| rs10982164 | Diabetic polyneuropathy | 9 | 117091067 | A/G | 0.034 | 0.102 | 0.213 | 0.632 | 0.034 | trans |
| rs56092644 | Diabetic polyneuropathy | 5 | 95774809 | T/G | 0.144 | 0.092 | 0.108 | 0.395 | 0.144 | trans |
| rs2208150 | Diabetic polyneuropathy | 20 | 16903467 | C/T | 0.641 | 0.083 | 0.078 | 0.287 | 0.359 | trans |
| rs826404 | Diabetic polyneuropathy | 1 | 118164722 | C/T | 0.621 | 0.059 | 0.078 | 0.450 | 0.379 | trans |
| rs5785928 | Diabetic polyneuropathy | 10 | 71320654 | CT/C | 0.647 | 0.032 | 0.079 | 0.685 | 0.353 | trans |
| rs76405485 | Diabetic polyneuropathy | 5 | 7339111 | C/T | 0.386 | 0.011 | 0.078 | 0.886 | 0.386 | trans |
| rs67131976 | Diabetic polyneuropathy | 6 | 20686878 | T/C | 0.173 | -0.013 | 0.100 | 0.897 | 0.173 | trans |
| rs11213809 | Diabetic polyneuropathy | 11 | 111135745 | G/A | 0.768 | -0.036 | 0.090 | 0.688 | 0.232 | trans |
| rs2251034 | Diabetic polyneuropathy | 19 | 49207792 | A/G | 0.410 | -0.061 | 0.077 | 0.429 | 0.410 | trans |
| rs17681738 | Diabetic polyneuropathy | 17 | 9793240 | T/C | 0.318 | -0.069 | 0.081 | 0.398 | 0.318 | trans |
| rs16862260 | Diabetic polyneuropathy | 2 | 222069957 | A/G | 0.291 | -0.089 | 0.084 | 0.292 | 0.291 | trans |
| rs870465 | Diabetic polyneuropathy | 1 | 3240199 | A/G | 0.103 | -0.130 | 0.126 | 0.302 | 0.103 | trans |
| rs147351977 | Diabetic polyneuropathy | 3 | 183939658 | A/G | 0.013 | -0.179 | 0.327 | 0.584 | 0.013 | trans |
| SORT1 |  |  |  |  |  |  |  |  |  |  |
| rs7176023 | Diabetic polyneuropathy | 15 | 65101007 | C/T | 0.078 | 0.069 | 0.141 | 0.622 | 0.078 | trans |
| rs2519093 | Diabetic polyneuropathy | 9 | 136141870 | T/C | 0.201 | 0.028 | 0.094 | 0.762 | 0.201 | trans |
| rs10984405 | Diabetic polyneuropathy | 9 | 100690555 | G/A | 0.398 | -0.028 | 0.077 | 0.714 | 0.398 | trans |
| rs12445050 | Diabetic polyneuropathy | 16 | 81870969 | T/C | 0.130 | -0.033 | 0.113 | 0.769 | 0.130 | trans |
| rs3184504 | Diabetic polyneuropathy | 12 | 111884608 | C/T | 0.591 | -0.051 | 0.077 | 0.504 | 0.409 | trans |
| rs4703589 | Diabetic polyneuropathy | 5 | 72097351 | C/T | 0.592 | -0.100 | 0.077 | 0.193 | 0.408 | trans |
| rs61394658 | Diabetic polyneuropathy | 1 | 109873290 | A/G | 0.217 | -0.103 | 0.092 | 0.260 | 0.217 | cis |
| rs892090 | Diabetic polyneuropathy | 19 | 55539072 | T/G | 0.121 | -0.126 | 0.115 | 0.272 | 0.121 | trans |
| TREML1 |  |  |  |  |  |  |  |  |  |  |
| rs10984599 | Diabetic polyneuropathy | 9 | 100736718 | T/C | 0.229 | -0.041 | 0.091 | 0.649 | 0.229 | trans |
| rs1775831 | Diabetic polyneuropathy | 1 | 118158079 | A/G | 0.380 | -0.065 | 0.078 | 0.403 | 0.380 | trans |
| GSN |  |  |  |  |  |  |  |  |  |  |
| rs10985196 | Diabetic polyneuropathy | 9 | 124033044 | A/C | 0.217 | -0.148 | 0.091 | 0.105 | 0.217 | cis |
| CDKN2D |  |  |  |  |  |  |  |  |  |  |
| rs1354034 | Diabetic polyneuropathy | 3 | 56849749 | C/T | 0.707 | 0.052 | 0.083 | 0.532 | 0.293 | trans |
| rs10990651 | Diabetic polyneuropathy | 9 | 99105420 | A/G | 0.272 | 0.048 | 0.085 | 0.578 | 0.272 | trans |
| MMP1 |  |  |  |  |  |  |  |  |  |  |
| rs471994 | Diabetic polyneuropathy | 11 | 102697731 | A/G | 0.343 | 0.078 | 0.080 | 0.326 | 0.343 | cis |
| rs10990651 | Diabetic polyneuropathy | 9 | 99105420 | A/G | 0.272 | 0.048 | 0.085 | 0.578 | 0.272 | trans |
| rs7832219 | Diabetic polyneuropathy | 8 | 106578977 | T/C | 0.780 | 0.013 | 0.091 | 0.889 | 0.220 | trans |
| DKK3 |  |  |  |  |  |  |  |  |  |  |
| rs11022114 | Diabetic polyneuropathy | 11 | 12038874 | A/G | 0.346 | 0.176 | 0.079 | 0.026 | 0.346 | cis |
| rs196255 | Diabetic polyneuropathy | 10 | 121480161 | T/C | 0.356 | 0.092 | 0.079 | 0.244 | 0.356 | trans |
| rs507666 | Diabetic polyneuropathy | 9 | 136149399 | A/G | 0.201 | 0.019 | 0.094 | 0.840 | 0.201 | trans |
| rs2137537 | Diabetic polyneuropathy | 12 | 71113087 | C/T | 0.640 | -0.044 | 0.079 | 0.581 | 0.360 | trans |
| rs1609860 | Diabetic polyneuropathy | 12 | 104048454 | A/C | 0.166 | -0.187 | 0.103 | 0.069 | 0.166 | trans |
| SAA2 |  |  |  |  |  |  |  |  |  |  |
| rs11024589 | Diabetic polyneuropathy | 11 | 18278423 | A/C | 0.904 | 0.131 | 0.129 | 0.308 | 0.096 | cis |
| CGA_FSHB |  |  |  |  |  |  |  |  |  |  |
| rs11031006 | Diabetic polyneuropathy | 11 | 30226528 | A/G | 0.166 | 0.065 | 0.101 | 0.522 | 0.166 | cis |
| MPPED2 |  |  |  |  |  |  |  |  |  |  |
| rs11031140 | Diabetic polyneuropathy | 11 | 30608133 | A/G | 0.138 | 0.043 | 0.109 | 0.695 | 0.138 | cis |
| NCAN |  |  |  |  |  |  |  |  |  |  |
| rs1126642 | Diabetic polyneuropathy | 17 | 42989063 | T/C | 0.016 | 0.076 | 0.291 | 0.795 | 0.016 | trans |
| rs11039297 | Diabetic polyneuropathy | 11 | 47581443 | A/G | 0.232 | 0.013 | 0.089 | 0.883 | 0.232 | trans |
| rs12148990 | Diabetic polyneuropathy | 16 | 17461995 | A/G | 0.269 | 0.003 | 0.085 | 0.970 | 0.269 | trans |
| rs1877223 | Diabetic polyneuropathy | 3 | 140997625 | G/T | 0.406 | -0.029 | 0.077 | 0.711 | 0.406 | trans |
| rs2228603 | Diabetic polyneuropathy | 19 | 19329924 | T/C | 0.070 | -0.054 | 0.149 | 0.718 | 0.070 | cis |
| PRL |  |  |  |  |  |  |  |  |  |  |
| rs2094622 | Diabetic polyneuropathy | 9 | 5105679 | A/C | 0.602 | -0.040 | 0.077 | 0.603 | 0.398 | trans |
| rs4738 | Diabetic polyneuropathy | 5 | 1461568 | T/C | 0.729 | -0.047 | 0.085 | 0.578 | 0.271 | trans |
| SCGN |  |  |  |  |  |  |  |  |  |  |
| rs601338 | Diabetic polyneuropathy | 19 | 49206674 | G/A | 0.626 | 0.044 | 0.078 | 0.571 | 0.375 | trans |
| rs11043076 | Diabetic polyneuropathy | 11 | 2223474 | T/C | 0.126 | -0.084 | 0.116 | 0.472 | 0.126 | trans |
| MEGF9 |  |  |  |  |  |  |  |  |  |  |
| rs77542162 | Diabetic polyneuropathy | 17 | 67081278 | A/G | 0.993 | 0.407 | 0.465 | 0.382 | 0.007 | trans |
| rs11045819 | Diabetic polyneuropathy | 12 | 21329813 | A/C | 0.112 | 0.186 | 0.119 | 0.118 | 0.112 | trans |
| rs1461729 | Diabetic polyneuropathy | 8 | 9187242 | G/A | 0.844 | 0.152 | 0.113 | 0.178 | 0.156 | trans |
| rs73066226 | Diabetic polyneuropathy | 19 | 59008213 | C/T | 0.184 | 0.125 | 0.097 | 0.199 | 0.184 | trans |
| rs13107325 | Diabetic polyneuropathy | 4 | 103188709 | T/C | 0.014 | 0.106 | 0.309 | 0.731 | 0.014 | trans |
| rs9633740 | Diabetic polyneuropathy | 10 | 82265271 | G/A | 0.778 | -0.045 | 0.091 | 0.618 | 0.222 | trans |
| rs7849566 | Diabetic polyneuropathy | 9 | 123460769 | C/A | 0.740 | -0.054 | 0.086 | 0.526 | 0.260 | cis |
| rs646776 | Diabetic polyneuropathy | 1 | 109818530 | T/C | 0.785 | -0.110 | 0.092 | 0.233 | 0.215 | trans |
| ALCAM |  |  |  |  |  |  |  |  |  |  |
| rs11045826 | Diabetic polyneuropathy | 12 | 21334599 | C/T | 0.112 | 0.186 | 0.119 | 0.119 | 0.112 | trans |
| rs34926152 | Diabetic polyneuropathy | 3 | 105264176 | T/G | 0.036 | 0.171 | 0.204 | 0.402 | 0.036 | cis |
| rs3747207 | Diabetic polyneuropathy | 22 | 44324855 | A/G | 0.226 | -0.010 | 0.090 | 0.915 | 0.226 | trans |
| rs2638282 | Diabetic polyneuropathy | 19 | 49213833 | A/G | 0.411 | -0.044 | 0.077 | 0.565 | 0.411 | trans |
| rs11075299 | Diabetic polyneuropathy | 16 | 16246164 | G/T | 0.341 | -0.063 | 0.080 | 0.432 | 0.341 | trans |
| CDH2 |  |  |  |  |  |  |  |  |  |  |
| rs181242111 | Diabetic polyneuropathy | 10 | 17865664 | A/G | 0.088 | 0.206 | 0.134 | 0.126 | 0.088 | trans |
| rs4074793 | Diabetic polyneuropathy | 5 | 52193125 | G/A | 0.070 | 0.141 | 0.146 | 0.336 | 0.070 | trans |
| rs11872284 | Diabetic polyneuropathy | 18 | 25680423 | T/C | 0.246 | 0.127 | 0.088 | 0.147 | 0.246 | cis |
| rs11869771 | Diabetic polyneuropathy | 17 | 9586722 | C/A | 0.255 | 0.003 | 0.087 | 0.970 | 0.255 | trans |
| rs28929474 | Diabetic polyneuropathy | 14 | 94844947 | T/C | 0.020 | -0.002 | 0.272 | 0.993 | 0.020 | trans |
| rs116698525 | Diabetic polyneuropathy | 16 | 578190 | T/C | 0.130 | -0.008 | 0.113 | 0.942 | 0.130 | trans |
| rs11045834 | Diabetic polyneuropathy | 12 | 21341096 | T/C | 0.239 | -0.023 | 0.088 | 0.791 | 0.239 | trans |
| rs7108216 | Diabetic polyneuropathy | 11 | 702097 | C/T | 0.648 | -0.025 | 0.079 | 0.749 | 0.353 | trans |
| rs17054431 | Diabetic polyneuropathy | 5 | 156722426 | T/C | 0.132 | -0.041 | 0.114 | 0.719 | 0.132 | trans |
| rs9633740 | Diabetic polyneuropathy | 10 | 82265271 | G/A | 0.778 | -0.045 | 0.091 | 0.618 | 0.222 | trans |
| rs112875651 | Diabetic polyneuropathy | 8 | 126506694 | A/G | 0.387 | -0.058 | 0.078 | 0.456 | 0.387 | trans |
| MEP1B |  |  |  |  |  |  |  |  |  |  |
| rs1800961 | Diabetic polyneuropathy | 20 | 43042364 | T/C | 0.045 | 0.445 | 0.180 | 0.013 | 0.045 | trans |
| rs75460349 | Diabetic polyneuropathy | 1 | 27180088 | C/A | 0.035 | 0.292 | 0.210 | 0.164 | 0.035 | trans |
| rs11045856 | Diabetic polyneuropathy | 12 | 21350689 | G/T | 0.172 | 0.098 | 0.100 | 0.326 | 0.172 | trans |
| rs3735165 | Diabetic polyneuropathy | 7 | 150068371 | C/T | 0.414 | 0.074 | 0.077 | 0.336 | 0.414 | trans |
| rs17616063 | Diabetic polyneuropathy | 16 | 51436882 | G/A | 0.141 | -0.008 | 0.110 | 0.946 | 0.141 | trans |
| rs112875651 | Diabetic polyneuropathy | 8 | 126506694 | A/G | 0.387 | -0.058 | 0.078 | 0.456 | 0.387 | trans |
| rs6973520 | Diabetic polyneuropathy | 7 | 128740443 | T/C | 0.373 | -0.065 | 0.078 | 0.406 | 0.373 | trans |
| rs1260326 | Diabetic polyneuropathy | 2 | 27730940 | T/C | 0.351 | -0.100 | 0.079 | 0.208 | 0.351 | trans |
| rs7979473 | Diabetic polyneuropathy | 12 | 121420260 | G/A | 0.581 | -0.108 | 0.077 | 0.161 | 0.419 | trans |
| rs174533 | Diabetic polyneuropathy | 11 | 61549025 | A/G | 0.415 | -0.125 | 0.077 | 0.104 | 0.415 | trans |
| rs1730862 | Diabetic polyneuropathy | 1 | 107614003 | A/G | 0.705 | -0.143 | 0.083 | 0.085 | 0.295 | trans |
| rs4895864 | Diabetic polyneuropathy | 6 | 130364482 | T/C | 0.601 | -0.170 | 0.077 | 0.027 | 0.399 | trans |
| PTPRF |  |  |  |  |  |  |  |  |  |  |
| rs11045856 | Diabetic polyneuropathy | 12 | 21350689 | G/T | 0.172 | 0.098 | 0.100 | 0.326 | 0.172 | trans |
| rs2842194 | Diabetic polyneuropathy | 1 | 44028962 | A/G | 0.186 | 0.061 | 0.097 | 0.529 | 0.186 | cis |
| rs17039766 | Diabetic polyneuropathy | 4 | 110006432 | A/C | 0.105 | 0.033 | 0.126 | 0.796 | 0.105 | trans |
| rs507666 | Diabetic polyneuropathy | 9 | 136149399 | A/G | 0.201 | 0.019 | 0.094 | 0.840 | 0.201 | trans |
| rs9873618 | Diabetic polyneuropathy | 3 | 170733076 | A/G | 0.267 | 0.016 | 0.085 | 0.854 | 0.267 | trans |
| rs7088799 | Diabetic polyneuropathy | 10 | 65016174 | G/T | 0.384 | 0.002 | 0.078 | 0.981 | 0.384 | trans |
| rs28929474 | Diabetic polyneuropathy | 14 | 94844947 | T/C | 0.020 | -0.002 | 0.272 | 0.993 | 0.020 | trans |
| rs9633740 | Diabetic polyneuropathy | 10 | 82265271 | G/A | 0.778 | -0.045 | 0.091 | 0.618 | 0.222 | trans |
| rs1535 | Diabetic polyneuropathy | 11 | 61597972 | G/A | 0.414 | -0.124 | 0.077 | 0.105 | 0.414 | trans |
| rs1801689 | Diabetic polyneuropathy | 17 | 64210580 | C/A | 0.010 | -0.158 | 0.383 | 0.681 | 0.010 | trans |
| ERBB2 |  |  |  |  |  |  |  |  |  |  |
| rs1800961 | Diabetic polyneuropathy | 20 | 43042364 | T/C | 0.045 | 0.445 | 0.180 | 0.013 | 0.045 | trans |
| rs1461729 | Diabetic polyneuropathy | 8 | 9187242 | G/A | 0.844 | 0.152 | 0.113 | 0.178 | 0.156 | trans |
| rs8176749 | Diabetic polyneuropathy | 9 | 136131188 | T/C | 0.134 | 0.149 | 0.111 | 0.181 | 0.134 | trans |
| rs11045856 | Diabetic polyneuropathy | 12 | 21350689 | G/T | 0.172 | 0.098 | 0.100 | 0.326 | 0.172 | trans |
| rs28929474 | Diabetic polyneuropathy | 14 | 94844947 | T/C | 0.020 | -0.002 | 0.272 | 0.993 | 0.020 | trans |
| rs3764354 | Diabetic polyneuropathy | 17 | 37916823 | T/C | 0.185 | -0.113 | 0.099 | 0.253 | 0.185 | cis |
| rs4788460 | Diabetic polyneuropathy | 16 | 72154509 | T/C | 0.315 | -0.118 | 0.082 | 0.152 | 0.315 | trans |
| rs1801689 | Diabetic polyneuropathy | 17 | 64210580 | C/A | 0.010 | -0.158 | 0.383 | 0.681 | 0.010 | trans |
| CDCP1 |  |  |  |  |  |  |  |  |  |  |
| rs7485656 | Diabetic polyneuropathy | 12 | 125315647 | G/A | 0.170 | 0.132 | 0.101 | 0.194 | 0.170 | trans |
| rs11048473 | Diabetic polyneuropathy | 12 | 26493814 | T/C | 0.262 | 0.018 | 0.087 | 0.837 | 0.262 | trans |
| rs3747207 | Diabetic polyneuropathy | 22 | 44324855 | A/G | 0.226 | -0.010 | 0.090 | 0.915 | 0.226 | trans |
| rs683486 | Diabetic polyneuropathy | 11 | 126226663 | A/C | 0.371 | -0.019 | 0.079 | 0.806 | 0.371 | trans |
| rs77029323 | Diabetic polyneuropathy | 10 | 64449879 | T/G | 0.351 | -0.031 | 0.079 | 0.692 | 0.351 | trans |
| COX5A |  |  |  |  |  |  |  |  |  |  |
| rs11049103 | Diabetic polyneuropathy | 12 | 27862081 | A/G | 0.149 | -0.083 | 0.107 | 0.437 | 0.149 | trans |
| FASLG |  |  |  |  |  |  |  |  |  |  |
| rs739141 | Diabetic polyneuropathy | 22 | 39824450 | T/C | 0.661 | 0.101 | 0.080 | 0.208 | 0.339 | trans |
| rs35477491 | Diabetic polyneuropathy | 21 | 15273817 | T/C | 0.281 | 0.094 | 0.088 | 0.282 | 0.281 | trans |
| rs9916257 | Diabetic polyneuropathy | 17 | 33797371 | T/G | 0.383 | 0.075 | 0.078 | 0.331 | 0.383 | trans |
| rs11127048 | Diabetic polyneuropathy | 2 | 27752463 | A/G | 0.626 | 0.065 | 0.079 | 0.406 | 0.374 | trans |
| rs738722 | Diabetic polyneuropathy | 22 | 29130012 | C/T | 0.748 | 0.062 | 0.087 | 0.478 | 0.252 | trans |
| rs8177655 | Diabetic polyneuropathy | 10 | 6015119 | A/G | 0.309 | 0.062 | 0.083 | 0.455 | 0.309 | trans |
| rs978522 | Diabetic polyneuropathy | 10 | 90748338 | C/T | 0.414 | 0.054 | 0.077 | 0.479 | 0.414 | trans |
| rs11053802 | Diabetic polyneuropathy | 12 | 10597207 | T/C | 0.654 | 0.019 | 0.080 | 0.812 | 0.347 | trans |
| rs11895938 | Diabetic polyneuropathy | 2 | 68619082 | G/A | 0.715 | -0.033 | 0.083 | 0.693 | 0.285 | trans |
| rs1205340 | Diabetic polyneuropathy | 20 | 32923871 | A/G | 0.411 | -0.037 | 0.077 | 0.628 | 0.411 | trans |
| rs13111860 | Diabetic polyneuropathy | 4 | 142612879 | G/A | 0.749 | -0.041 | 0.087 | 0.641 | 0.251 | trans |
| rs3184504 | Diabetic polyneuropathy | 12 | 111884608 | C/T | 0.591 | -0.051 | 0.077 | 0.504 | 0.409 | trans |
| rs748988 | Diabetic polyneuropathy | 14 | 99786241 | G/A | 0.626 | -0.091 | 0.079 | 0.247 | 0.374 | trans |
| AMY2B |  |  |  |  |  |  |  |  |  |  |
| rs550057 | Diabetic polyneuropathy | 9 | 136146597 | T/C | 0.302 | 0.128 | 0.082 | 0.121 | 0.302 | trans |
| rs533406 | Diabetic polyneuropathy | 16 | 88974860 | G/A | 0.269 | 0.110 | 0.085 | 0.197 | 0.269 | trans |
| rs55901622 | Diabetic polyneuropathy | 6 | 127157438 | G/A | 0.194 | 0.041 | 0.096 | 0.674 | 0.194 | trans |
| rs56278466 | Diabetic polyneuropathy | 10 | 17875857 | G/T | 0.581 | -0.003 | 0.077 | 0.970 | 0.419 | trans |
| rs11054116 | Diabetic polyneuropathy | 12 | 11123337 | A/G | 0.276 | -0.040 | 0.084 | 0.632 | 0.276 | trans |
| rs2638282 | Diabetic polyneuropathy | 19 | 49213833 | A/G | 0.411 | -0.044 | 0.077 | 0.565 | 0.411 | trans |
| rs28456 | Diabetic polyneuropathy | 11 | 61589481 | G/A | 0.390 | -0.104 | 0.077 | 0.178 | 0.390 | trans |
| rs12080068 | Diabetic polyneuropathy | 1 | 104329823 | A/C | 0.082 | -0.112 | 0.140 | 0.423 | 0.082 | cis |
| rs1561929 | Diabetic polyneuropathy | 8 | 129567373 | T/C | 0.873 | -0.139 | 0.113 | 0.220 | 0.127 | trans |
| GALNT7 |  |  |  |  |  |  |  |  |  |  |
| rs11054859 | Diabetic polyneuropathy | 12 | 7769776 | A/G | 0.098 | 0.249 | 0.126 | 0.048 | 0.098 | cis |
| rs12975366 | Diabetic polyneuropathy | 19 | 54759361 | C/T | 0.372 | 0.142 | 0.079 | 0.071 | 0.372 | trans |
| rs13107325 | Diabetic polyneuropathy | 4 | 103188709 | T/C | 0.014 | 0.106 | 0.309 | 0.731 | 0.014 | trans |
| rs117905808 | Diabetic polyneuropathy | 7 | 100509130 | A/G | 0.005 | 0.043 | 0.520 | 0.934 | 0.005 | trans |
| TNFRSF10B |  |  |  |  |  |  |  |  |  |  |
| rs3184504 | Diabetic polyneuropathy | 12 | 111884608 | C/T | 0.591 | -0.051 | 0.077 | 0.504 | 0.409 | trans |
| rs231996 | Diabetic polyneuropathy | 3 | 172273735 | A/C | 0.818 | -0.071 | 0.097 | 0.462 | 0.182 | trans |
| rs1105944 | Diabetic polyneuropathy | 8 | 22885109 | G/A | 0.932 | -0.245 | 0.150 | 0.103 | 0.068 | cis |
| CCL13 |  |  |  |  |  |  |  |  |  |  |
| rs7433284 | Diabetic polyneuropathy | 3 | 42872590 | A/G | 0.346 | 0.135 | 0.080 | 0.090 | 0.346 | trans |
| rs2737245 | Diabetic polyneuropathy | 8 | 116658583 | T/G | 0.309 | 0.099 | 0.082 | 0.229 | 0.309 | trans |
| rs7080386 | Diabetic polyneuropathy | 10 | 65048306 | A/C | 0.384 | -0.001 | 0.078 | 0.993 | 0.384 | trans |
| rs2842059 | Diabetic polyneuropathy | 6 | 71344854 | A/C | 0.332 | -0.024 | 0.081 | 0.770 | 0.332 | trans |
| rs12445050 | Diabetic polyneuropathy | 16 | 81870969 | T/C | 0.130 | -0.033 | 0.113 | 0.769 | 0.130 | trans |
| rs12495098 | Diabetic polyneuropathy | 3 | 46342502 | G/T | 0.077 | -0.061 | 0.139 | 0.660 | 0.077 | trans |
| rs892090 | Diabetic polyneuropathy | 19 | 55539072 | T/G | 0.121 | -0.126 | 0.115 | 0.272 | 0.121 | trans |
| rs3136674 | Diabetic polyneuropathy | 17 | 32682616 | C/T | 0.054 | -0.145 | 0.168 | 0.388 | 0.054 | cis |
| rs13412535 | Diabetic polyneuropathy | 2 | 224874874 | A/G | 0.205 | -0.204 | 0.095 | 0.032 | 0.205 | trans |
| SPN |  |  |  |  |  |  |  |  |  |  |
| rs11064498 | Diabetic polyneuropathy | 12 | 7171507 | A/G | 0.775 | 0.057 | 0.091 | 0.531 | 0.225 | trans |
| SIGLEC14 |  |  |  |  |  |  |  |  |  |  |
| rs3820897 | Diabetic polyneuropathy | 2 | 3642361 | T/C | 0.353 | -0.069 | 0.080 | 0.386 | 0.353 | trans |
| SIGLEC5 |  |  |  |  |  |  |  |  |  |  |
| rs7137828 | Diabetic polyneuropathy | 12 | 111932800 | T/C | 0.585 | -0.054 | 0.077 | 0.483 | 0.415 | trans |
| RPN1 |  |  |  |  |  |  |  |  |  |  |
| rs2712417 | Diabetic polyneuropathy | 3 | 128345179 | G/A | 0.615 | -0.059 | 0.077 | 0.442 | 0.385 | cis |
| PLA2G1B |  |  |  |  |  |  |  |  |  |  |
| rs2438988 | Diabetic polyneuropathy | 10 | 49370090 | G/A | 0.638 | 0.143 | 0.080 | 0.073 | 0.362 | trans |
| rs1372993 | Diabetic polyneuropathy | 8 | 129565662 | A/G | 0.127 | 0.142 | 0.113 | 0.211 | 0.127 | trans |
| rs61729512 | Diabetic polyneuropathy | 12 | 7637769 | A/G | 0.152 | 0.101 | 0.107 | 0.348 | 0.152 | trans |
| rs1673931 | Diabetic polyneuropathy | 16 | 88976477 | C/T | 0.273 | 0.097 | 0.085 | 0.250 | 0.273 | trans |
| rs11065078 | Diabetic polyneuropathy | 12 | 120748585 | T/C | 0.226 | 0.052 | 0.090 | 0.562 | 0.226 | cis |
| rs71327329 | Diabetic polyneuropathy | 22 | 29327347 | G/A | 0.111 | 0.035 | 0.120 | 0.770 | 0.111 | trans |
| rs6507586 | Diabetic polyneuropathy | 18 | 42415090 | G/A | 0.077 | -0.007 | 0.144 | 0.963 | 0.077 | trans |
| rs1468481 | Diabetic polyneuropathy | 17 | 69139979 | T/C | 0.706 | -0.010 | 0.083 | 0.903 | 0.294 | trans |
| rs72802342 | Diabetic polyneuropathy | 16 | 75234872 | A/C | 0.086 | -0.032 | 0.135 | 0.814 | 0.086 | trans |
| rs4751995 | Diabetic polyneuropathy | 10 | 118397884 | G/A | 0.624 | -0.035 | 0.078 | 0.658 | 0.376 | trans |
| rs681343 | Diabetic polyneuropathy | 19 | 49206462 | T/C | 0.375 | -0.045 | 0.078 | 0.568 | 0.375 | trans |
| rs17032925 | Diabetic polyneuropathy | 2 | 67266483 | C/T | 0.079 | -0.074 | 0.141 | 0.598 | 0.079 | trans |
| rs1260326 | Diabetic polyneuropathy | 2 | 27730940 | T/C | 0.351 | -0.100 | 0.079 | 0.208 | 0.351 | trans |
| rs116842520 | Diabetic polyneuropathy | 9 | 135954065 | A/G | 0.028 | -0.200 | 0.231 | 0.387 | 0.028 | trans |
| ACAN |  |  |  |  |  |  |  |  |  |  |
| rs1337526 | Diabetic polyneuropathy | 1 | 47965130 | G/A | 0.841 | 0.261 | 0.103 | 0.011 | 0.159 | trans |
| rs6461354 | Diabetic polyneuropathy | 7 | 17914600 | T/C | 0.298 | 0.063 | 0.082 | 0.444 | 0.298 | trans |
| rs2519093 | Diabetic polyneuropathy | 9 | 136141870 | T/C | 0.201 | 0.028 | 0.094 | 0.762 | 0.201 | trans |
| rs12684083 | Diabetic polyneuropathy | 9 | 79148591 | A/G | 0.307 | 0.025 | 0.082 | 0.762 | 0.307 | trans |
| rs17549173 | Diabetic polyneuropathy | 19 | 36400231 | A/G | 0.087 | 0.017 | 0.134 | 0.900 | 0.087 | trans |
| rs59379014 | Diabetic polyneuropathy | 11 | 126228000 | T/C | 0.081 | 0.002 | 0.140 | 0.991 | 0.081 | trans |
| rs828602 | Diabetic polyneuropathy | 3 | 98473318 | A/G | 0.393 | 0.000 | 0.077 | 0.999 | 0.393 | trans |
| rs56278466 | Diabetic polyneuropathy | 10 | 17875857 | G/T | 0.581 | -0.003 | 0.077 | 0.970 | 0.419 | trans |
| rs11713838 | Diabetic polyneuropathy | 3 | 118928491 | A/C | 0.365 | -0.015 | 0.079 | 0.846 | 0.365 | trans |
| APOL1 |  |  |  |  |  |  |  |  |  |  |
| rs11075921 | Diabetic polyneuropathy | 16 | 72132129 | T/C | 0.167 | -0.063 | 0.101 | 0.532 | 0.167 | trans |
| rs72840032 | Diabetic polyneuropathy | 10 | 101889964 | T/C | 0.038 | -0.213 | 0.196 | 0.278 | 0.038 | trans |
| APOA1 |  |  |  |  |  |  |  |  |  |  |
| rs11075921 | Diabetic polyneuropathy | 16 | 72132129 | T/C | 0.167 | -0.063 | 0.101 | 0.532 | 0.167 | trans |
| PRPSAP2 |  |  |  |  |  |  |  |  |  |  |
| rs11078421 | Diabetic polyneuropathy | 17 | 18804385 | T/C | 0.605 | 0.028 | 0.077 | 0.717 | 0.395 | cis |
| NPW |  |  |  |  |  |  |  |  |  |  |
| rs11078596 | Diabetic polyneuropathy | 17 | 1618262 | T/C | 0.189 | 0.014 | 0.097 | 0.889 | 0.189 | trans |
| rs28929474 | Diabetic polyneuropathy | 14 | 94844947 | T/C | 0.020 | -0.002 | 0.272 | 0.993 | 0.020 | trans |
| rs1260326 | Diabetic polyneuropathy | 2 | 27730940 | T/C | 0.351 | -0.100 | 0.079 | 0.208 | 0.351 | trans |
| rs12921264 | Diabetic polyneuropathy | 16 | 2070433 | A/G | 0.212 | -0.108 | 0.093 | 0.247 | 0.212 | cis |
| rs77262773 | Diabetic polyneuropathy | 17 | 67249711 | T/C | 0.007 | -0.544 | 0.464 | 0.241 | 0.007 | trans |
| SERPINF2 |  |  |  |  |  |  |  |  |  |  |
| rs11078597 | Diabetic polyneuropathy | 17 | 1618363 | C/T | 0.189 | 0.013 | 0.096 | 0.891 | 0.189 | trans |
| SLC39A5 |  |  |  |  |  |  |  |  |  |  |
| rs2272662 | Diabetic polyneuropathy | 8 | 145639726 | C/T | 0.604 | 0.155 | 0.079 | 0.051 | 0.396 | trans |
| rs12065546 | Diabetic polyneuropathy | 1 | 230295245 | T/C | 0.809 | 0.060 | 0.097 | 0.537 | 0.192 | trans |
| rs7786376 | Diabetic polyneuropathy | 7 | 73042614 | G/A | 0.216 | 0.031 | 0.091 | 0.737 | 0.216 | trans |
| rs11078597 | Diabetic polyneuropathy | 17 | 1618363 | C/T | 0.189 | 0.013 | 0.096 | 0.891 | 0.189 | trans |
| rs28929474 | Diabetic polyneuropathy | 14 | 94844947 | T/C | 0.020 | -0.002 | 0.272 | 0.993 | 0.020 | trans |
| rs55953905 | Diabetic polyneuropathy | 21 | 42882671 | T/C | 0.255 | -0.072 | 0.086 | 0.406 | 0.255 | trans |
| CNP |  |  |  |  |  |  |  |  |  |  |
| rs11079027 | Diabetic polyneuropathy | 17 | 40123521 | A/G | 0.349 | 0.091 | 0.080 | 0.255 | 0.349 | cis |
| MSTN_GDF11 |  |  |  |  |  |  |  |  |  |  |
| rs1815739 | Diabetic polyneuropathy | 11 | 66328095 | C/T | 0.679 | 0.159 | 0.081 | 0.050 | 0.321 | trans |
| rs11079936 | Diabetic polyneuropathy | 17 | 48907834 | C/T | 0.656 | 0.050 | 0.080 | 0.536 | 0.344 | trans |
| CABLES2 |  |  |  |  |  |  |  |  |  |  |
| rs2236201 | Diabetic polyneuropathy | 20 | 60985627 | T/C | 0.675 | 0.083 | 0.081 | 0.306 | 0.325 | cis |
| FIBP |  |  |  |  |  |  |  |  |  |  |
| rs1354034 | Diabetic polyneuropathy | 3 | 56849749 | C/T | 0.707 | 0.052 | 0.083 | 0.532 | 0.293 | trans |
| B4GALT2 |  |  |  |  |  |  |  |  |  |  |
| rs3762423 | Diabetic polyneuropathy | 1 | 44445667 | T/C | 0.972 | 0.227 | 0.227 | 0.318 | 0.028 | cis |
| rs550057 | Diabetic polyneuropathy | 9 | 136146597 | T/C | 0.302 | 0.128 | 0.082 | 0.121 | 0.302 | trans |
| rs17695224 | Diabetic polyneuropathy | 19 | 52324216 | A/G | 0.187 | 0.115 | 0.097 | 0.235 | 0.187 | trans |
| rs7124974 | Diabetic polyneuropathy | 11 | 59906972 | T/G | 0.317 | 0.080 | 0.081 | 0.324 | 0.317 | trans |
| rs11082551 | Diabetic polyneuropathy | 18 | 44291419 | A/G | 0.637 | 0.064 | 0.079 | 0.417 | 0.363 | trans |
| RAD51L3 |  |  |  |  |  |  |  |  |  |  |
| rs1108301 | Diabetic polyneuropathy | 3 | 47029426 | A/G | 0.398 | -0.054 | 0.077 | 0.483 | 0.398 | trans |
| rs45446698 | Diabetic polyneuropathy | 7 | 99332948 | G/T | 0.052 | -0.288 | 0.171 | 0.092 | 0.052 | trans |
| MUL1 |  |  |  |  |  |  |  |  |  |  |
| rs11086556 | Diabetic polyneuropathy | 20 | 36969972 | G/A | 0.060 | -0.070 | 0.160 | 0.659 | 0.060 | trans |
| FBLN1 |  |  |  |  |  |  |  |  |  |  |
| rs11090631 | Diabetic polyneuropathy | 22 | 45846371 | T/C | 0.194 | 0.162 | 0.096 | 0.089 | 0.194 | cis |
| HS3ST3B1 |  |  |  |  |  |  |  |  |  |  |
| rs13107325 | Diabetic polyneuropathy | 4 | 103188709 | T/C | 0.014 | 0.106 | 0.309 | 0.731 | 0.014 | trans |
| rs1801020 | Diabetic polyneuropathy | 5 | 176836532 | A/G | 0.261 | -0.001 | 0.086 | 0.990 | 0.261 | trans |
| rs28929474 | Diabetic polyneuropathy | 14 | 94844947 | T/C | 0.020 | -0.002 | 0.272 | 0.993 | 0.020 | trans |
| rs17669311 | Diabetic polyneuropathy | 17 | 13837051 | A/G | 0.321 | -0.014 | 0.081 | 0.865 | 0.321 | cis |
| rs5030062 | Diabetic polyneuropathy | 3 | 186454180 | C/A | 0.348 | -0.045 | 0.079 | 0.573 | 0.348 | trans |
| rs9302635 | Diabetic polyneuropathy | 16 | 72144174 | C/T | 0.174 | -0.069 | 0.099 | 0.482 | 0.174 | trans |
| MZT1 |  |  |  |  |  |  |  |  |  |  |
| rs1354034 | Diabetic polyneuropathy | 3 | 56849749 | C/T | 0.707 | 0.052 | 0.083 | 0.532 | 0.293 | trans |
| rs11101690 | Diabetic polyneuropathy | 10 | 135121732 | C/T | 0.118 | -0.055 | 0.116 | 0.636 | 0.118 | trans |
| RBP5 |  |  |  |  |  |  |  |  |  |  |
| rs76904513 | Diabetic polyneuropathy | 12 | 7278118 | G/A | 0.053 | 0.199 | 0.169 | 0.239 | 0.053 | cis |
| rs1110236 | Diabetic polyneuropathy | 9 | 95902595 | A/G | 0.108 | 0.066 | 0.122 | 0.587 | 0.108 | trans |
| rs112875651 | Diabetic polyneuropathy | 8 | 126506694 | A/G | 0.387 | -0.058 | 0.078 | 0.456 | 0.387 | trans |
| LCN1 |  |  |  |  |  |  |  |  |  |  |
| rs11103042 | Diabetic polyneuropathy | 9 | 138404116 | A/G | 0.137 | -0.080 | 0.110 | 0.467 | 0.137 | cis |
| FCN1 |  |  |  |  |  |  |  |  |  |  |
| rs11103602 | Diabetic polyneuropathy | 9 | 137854872 | A/G | 0.353 | 0.034 | 0.079 | 0.670 | 0.353 | cis |
| KITLG |  |  |  |  |  |  |  |  |  |  |
| rs34931250 | Diabetic polyneuropathy | 17 | 66879927 | T/C | 0.066 | 0.464 | 0.154 | 0.003 | 0.066 | trans |
| rs983309 | Diabetic polyneuropathy | 8 | 9177732 | G/T | 0.831 | 0.119 | 0.108 | 0.272 | 0.169 | trans |
| rs247617 | Diabetic polyneuropathy | 16 | 56990716 | A/C | 0.279 | 0.042 | 0.084 | 0.617 | 0.279 | trans |
| rs673335 | Diabetic polyneuropathy | 11 | 75450576 | C/T | 0.241 | 0.031 | 0.088 | 0.721 | 0.241 | trans |
| rs11105121 | Diabetic polyneuropathy | 12 | 89364586 | T/C | 0.660 | 0.028 | 0.080 | 0.731 | 0.340 | cis |
| rs635634 | Diabetic polyneuropathy | 9 | 136155000 | T/C | 0.200 | 0.020 | 0.095 | 0.833 | 0.200 | trans |
| rs705379 | Diabetic polyneuropathy | 7 | 94953895 | A/G | 0.366 | 0.018 | 0.079 | 0.817 | 0.366 | trans |
| rs6672758 | Diabetic polyneuropathy | 1 | 230303512 | T/C | 0.742 | -0.004 | 0.087 | 0.963 | 0.258 | trans |
| rs2868346 | Diabetic polyneuropathy | 20 | 44547970 | T/C | 0.749 | -0.005 | 0.087 | 0.955 | 0.251 | trans |
| rs2292318 | Diabetic polyneuropathy | 16 | 67985706 | T/C | 0.156 | -0.016 | 0.105 | 0.876 | 0.156 | trans |
| rs112875651 | Diabetic polyneuropathy | 8 | 126506694 | A/G | 0.387 | -0.058 | 0.078 | 0.456 | 0.387 | trans |
| rs174564 | Diabetic polyneuropathy | 11 | 61588305 | G/A | 0.415 | -0.118 | 0.077 | 0.124 | 0.415 | trans |
| rs367070 | Diabetic polyneuropathy | 19 | 54800500 | G/A | 0.299 | -0.125 | 0.083 | 0.132 | 0.299 | trans |
| rs150844304 | Diabetic polyneuropathy | 15 | 43726625 | C/A | 0.005 | -0.223 | 0.528 | 0.673 | 0.005 | trans |
| CD7 |  |  |  |  |  |  |  |  |  |  |
| rs11105298 | Diabetic polyneuropathy | 12 | 89876143 | T/C | 0.776 | 0.187 | 0.091 | 0.039 | 0.224 | trans |
| rs3176831 | Diabetic polyneuropathy | 17 | 80272491 | T/C | 0.039 | -0.170 | 0.195 | 0.383 | 0.039 | cis |
| CD300A |  |  |  |  |  |  |  |  |  |  |
| rs11105298 | Diabetic polyneuropathy | 12 | 89876143 | T/C | 0.776 | 0.187 | 0.091 | 0.039 | 0.224 | trans |
| rs11888817 | Diabetic polyneuropathy | 2 | 62530980 | T/C | 0.349 | 0.185 | 0.080 | 0.020 | 0.349 | trans |
| rs2272111 | Diabetic polyneuropathy | 17 | 72469966 | A/G | 0.202 | 0.093 | 0.094 | 0.324 | 0.202 | cis |
| PCDHA4 |  |  |  |  |  |  |  |  |  |  |
| rs2511737 | Diabetic polyneuropathy | 8 | 103580527 | C/A | 0.290 | 0.058 | 0.084 | 0.490 | 0.290 | trans |
| rs1110701 | Diabetic polyneuropathy | 7 | 50478627 | G/A | 0.326 | -0.007 | 0.081 | 0.927 | 0.326 | trans |
| rs1953209 | Diabetic polyneuropathy | 14 | 30325693 | G/A | 0.285 | -0.023 | 0.084 | 0.780 | 0.285 | trans |
| rs12989427 | Diabetic polyneuropathy | 2 | 61137506 | A/G | 0.216 | -0.053 | 0.092 | 0.567 | 0.216 | trans |
| rs735665 | Diabetic polyneuropathy | 11 | 123361397 | A/G | 0.220 | -0.056 | 0.091 | 0.540 | 0.220 | trans |
| rs2476601 | Diabetic polyneuropathy | 1 | 114377568 | G/A | 0.852 | -0.179 | 0.107 | 0.093 | 0.148 | trans |
| CREG1 |  |  |  |  |  |  |  |  |  |  |
| rs11111026 | Diabetic polyneuropathy | 12 | 102192803 | T/G | 0.203 | 0.265 | 0.094 | 0.005 | 0.203 | trans |
| rs34894639 | Diabetic polyneuropathy | 3 | 135798658 | T/C | 0.188 | 0.037 | 0.097 | 0.705 | 0.188 | trans |
| rs56255430 | Diabetic polyneuropathy | 19 | 19477877 | C/A | 0.066 | -0.029 | 0.151 | 0.846 | 0.066 | trans |
| rs7513428 | Diabetic polyneuropathy | 1 | 167515272 | T/C | 0.099 | -0.067 | 0.125 | 0.593 | 0.099 | cis |
| rs429358 | Diabetic polyneuropathy | 19 | 45411941 | C/T | 0.183 | -0.237 | 0.100 | 0.018 | 0.183 | trans |
| RNASET2 |  |  |  |  |  |  |  |  |  |  |
| rs11111026 | Diabetic polyneuropathy | 12 | 102192803 | T/G | 0.203 | 0.265 | 0.094 | 0.005 | 0.203 | trans |
| rs13107325 | Diabetic polyneuropathy | 4 | 103188709 | T/C | 0.014 | 0.106 | 0.309 | 0.731 | 0.014 | trans |
| rs2721961 | Diabetic polyneuropathy | 8 | 116657911 | G/T | 0.309 | 0.102 | 0.082 | 0.214 | 0.309 | trans |
| rs3756838 | Diabetic polyneuropathy | 6 | 167371251 | A/G | 0.258 | -0.051 | 0.087 | 0.562 | 0.258 | cis |
| rs61747728 | Diabetic polyneuropathy | 1 | 179526214 | T/C | 0.064 | -0.201 | 0.158 | 0.204 | 0.064 | trans |
| TNR |  |  |  |  |  |  |  |  |  |  |
| rs11594905 | Diabetic polyneuropathy | 10 | 77659733 | A/G | 0.066 | 0.189 | 0.153 | 0.216 | 0.066 | trans |
| rs4981022 | Diabetic polyneuropathy | 12 | 104149874 | A/G | 0.683 | 0.109 | 0.082 | 0.184 | 0.317 | trans |
| rs1727 | Diabetic polyneuropathy | 10 | 91066769 | A/C | 0.798 | 0.095 | 0.094 | 0.310 | 0.203 | trans |
| rs2169305 | Diabetic polyneuropathy | 3 | 159509380 | C/T | 0.303 | 0.060 | 0.082 | 0.463 | 0.303 | trans |
| rs12879626 | Diabetic polyneuropathy | 14 | 34721134 | G/T | 0.640 | 0.043 | 0.079 | 0.591 | 0.360 | trans |
| rs3184504 | Diabetic polyneuropathy | 12 | 111884608 | C/T | 0.591 | -0.051 | 0.077 | 0.504 | 0.409 | trans |
| rs11603123 | Diabetic polyneuropathy | 11 | 126305495 | A/G | 0.035 | -0.318 | 0.210 | 0.131 | 0.035 | trans |
| TFPI |  |  |  |  |  |  |  |  |  |  |
| rs7412 | Diabetic polyneuropathy | 19 | 45412079 | T/C | 0.053 | 0.166 | 0.164 | 0.311 | 0.053 | trans |
| rs7576066 | Diabetic polyneuropathy | 2 | 188343781 | A/G | 0.310 | 0.105 | 0.082 | 0.198 | 0.310 | cis |
| rs12127364 | Diabetic polyneuropathy | 1 | 169469142 | A/C | 0.217 | -0.025 | 0.091 | 0.782 | 0.217 | trans |
| rs1260326 | Diabetic polyneuropathy | 2 | 27730940 | T/C | 0.351 | -0.100 | 0.079 | 0.208 | 0.351 | trans |
| ATP13A1 |  |  |  |  |  |  |  |  |  |  |
| rs704 | Diabetic polyneuropathy | 17 | 26694861 | A/G | 0.420 | 0.049 | 0.076 | 0.519 | 0.420 | trans |
| rs11122449 | Diabetic polyneuropathy | 1 | 230300481 | T/C | 0.678 | 0.047 | 0.081 | 0.561 | 0.322 | trans |
| rs35004449 | Diabetic polyneuropathy | 3 | 52852897 | T/G | 0.287 | -0.028 | 0.083 | 0.741 | 0.287 | trans |
| ROR1 |  |  |  |  |  |  |  |  |  |  |
| rs111239279 | Diabetic polyneuropathy | 7 | 155670175 | C/T | 0.009 | 0.698 | 0.417 | 0.094 | 0.009 | trans |
| rs13107325 | Diabetic polyneuropathy | 4 | 103188709 | T/C | 0.014 | 0.106 | 0.309 | 0.731 | 0.014 | trans |
| rs33950747 | Diabetic polyneuropathy | 19 | 36339247 | T/C | 0.086 | 0.042 | 0.137 | 0.757 | 0.086 | trans |
| rs77924615 | Diabetic polyneuropathy | 16 | 20392332 | A/G | 0.222 | 0.042 | 0.091 | 0.643 | 0.222 | trans |
| rs2298475 | Diabetic polyneuropathy | 11 | 126278203 | C/T | 0.107 | -0.040 | 0.123 | 0.743 | 0.107 | trans |
| rs1260326 | Diabetic polyneuropathy | 2 | 27730940 | T/C | 0.351 | -0.100 | 0.079 | 0.208 | 0.351 | trans |
| rs150816167 | Diabetic polyneuropathy | 1 | 179571862 | C/T | 0.064 | -0.189 | 0.158 | 0.233 | 0.064 | trans |
| rs6588083 | Diabetic polyneuropathy | 1 | 64614011 | C/T | 0.298 | -0.196 | 0.083 | 0.019 | 0.298 | cis |
| rs75166367 | Diabetic polyneuropathy | 2 | 162964301 | A/G | 0.071 | -0.204 | 0.149 | 0.170 | 0.071 | trans |
| REG1B |  |  |  |  |  |  |  |  |  |  |
| rs4421693 | Diabetic polyneuropathy | 10 | 124306428 | T/G | 0.599 | 0.098 | 0.078 | 0.207 | 0.401 | trans |
| rs492602 | Diabetic polyneuropathy | 19 | 49206417 | A/G | 0.625 | 0.045 | 0.078 | 0.566 | 0.375 | trans |
| rs11126696 | Diabetic polyneuropathy | 2 | 79323888 | G/A | 0.602 | 0.003 | 0.077 | 0.971 | 0.398 | cis |
| rs17802036 | Diabetic polyneuropathy | 12 | 68667269 | T/C | 0.293 | -0.011 | 0.083 | 0.899 | 0.293 | trans |
| rs72802342 | Diabetic polyneuropathy | 16 | 75234872 | A/C | 0.086 | -0.032 | 0.135 | 0.814 | 0.086 | trans |
| rs708686 | Diabetic polyneuropathy | 19 | 5840619 | T/C | 0.334 | -0.105 | 0.081 | 0.194 | 0.334 | trans |
| APOA5 |  |  |  |  |  |  |  |  |  |  |
| rs11127048 | Diabetic polyneuropathy | 2 | 27752463 | A/G | 0.626 | 0.065 | 0.079 | 0.406 | 0.374 | trans |
| LDHA |  |  |  |  |  |  |  |  |  |  |
| rs111294128 | Diabetic polyneuropathy | 11 | 18411722 | A/G | 0.989 | 0.303 | 0.352 | 0.390 | 0.011 | cis |
| rs74626198 | Diabetic polyneuropathy | 12 | 21696251 | T/C | 0.955 | 0.264 | 0.186 | 0.155 | 0.045 | trans |
| rs1354034 | Diabetic polyneuropathy | 3 | 56849749 | C/T | 0.707 | 0.052 | 0.083 | 0.532 | 0.293 | trans |
| rs113315674 | Diabetic polyneuropathy | 9 | 100720053 | A/G | 0.401 | -0.012 | 0.078 | 0.879 | 0.401 | trans |
| SPINK4 |  |  |  |  |  |  |  |  |  |  |
| rs4879679 | Diabetic polyneuropathy | 9 | 33234019 | T/C | 0.857 | 0.169 | 0.107 | 0.115 | 0.143 | cis |
| rs2834350 | Diabetic polyneuropathy | 21 | 35397128 | T/C | 0.342 | 0.069 | 0.079 | 0.386 | 0.342 | trans |
| rs28529068 | Diabetic polyneuropathy | 18 | 19673005 | G/A | 0.267 | -0.061 | 0.086 | 0.476 | 0.267 | trans |
| RSPO1 |  |  |  |  |  |  |  |  |  |  |
| rs36043533 | Diabetic polyneuropathy | 1 | 38079517 | G/T | 0.027 | 0.076 | 0.238 | 0.748 | 0.027 | cis |
| rs11130124 | Diabetic polyneuropathy | 3 | 47292183 | C/T | 0.614 | 0.062 | 0.077 | 0.424 | 0.387 | trans |
| rs1354034 | Diabetic polyneuropathy | 3 | 56849749 | C/T | 0.707 | 0.052 | 0.083 | 0.532 | 0.293 | trans |
| rs12445050 | Diabetic polyneuropathy | 16 | 81870969 | T/C | 0.130 | -0.033 | 0.113 | 0.769 | 0.130 | trans |
| rs3184504 | Diabetic polyneuropathy | 12 | 111884608 | C/T | 0.591 | -0.051 | 0.077 | 0.504 | 0.409 | trans |
| rs61469632 | Diabetic polyneuropathy | 9 | 135861990 | C/T | 0.058 | -0.126 | 0.164 | 0.442 | 0.058 | trans |
| rs892090 | Diabetic polyneuropathy | 19 | 55539072 | T/G | 0.121 | -0.126 | 0.115 | 0.272 | 0.121 | trans |
| rs13412535 | Diabetic polyneuropathy | 2 | 224874874 | A/G | 0.205 | -0.204 | 0.095 | 0.032 | 0.205 | trans |
| TMPRSS15 |  |  |  |  |  |  |  |  |  |  |
| rs550057 | Diabetic polyneuropathy | 9 | 136146597 | T/C | 0.302 | 0.128 | 0.082 | 0.121 | 0.302 | trans |
| rs492602 | Diabetic polyneuropathy | 19 | 49206417 | A/G | 0.625 | 0.045 | 0.078 | 0.566 | 0.375 | trans |
| rs59922816 | Diabetic polyneuropathy | 8 | 41378297 | C/T | 0.067 | -0.006 | 0.151 | 0.967 | 0.067 | trans |
| rs708686 | Diabetic polyneuropathy | 19 | 5840619 | T/C | 0.334 | -0.105 | 0.081 | 0.194 | 0.334 | trans |
| rs11134475 | Diabetic polyneuropathy | 5 | 156399950 | G/A | 0.669 | -0.107 | 0.081 | 0.183 | 0.331 | trans |
| rs438811 | Diabetic polyneuropathy | 19 | 45416741 | T/C | 0.236 | -0.146 | 0.090 | 0.103 | 0.236 | trans |
| MFGE8 |  |  |  |  |  |  |  |  |  |  |
| rs17145750 | Diabetic polyneuropathy | 7 | 73026378 | T/C | 0.148 | 0.036 | 0.106 | 0.731 | 0.148 | trans |
| rs6602913 | Diabetic polyneuropathy | 13 | 114544125 | C/A | 0.370 | 0.030 | 0.079 | 0.699 | 0.370 | trans |
| rs2980888 | Diabetic polyneuropathy | 8 | 126507308 | C/T | 0.741 | -0.070 | 0.086 | 0.415 | 0.259 | trans |
| rs1260326 | Diabetic polyneuropathy | 2 | 27730940 | T/C | 0.351 | -0.100 | 0.079 | 0.208 | 0.351 | trans |
| rs11134475 | Diabetic polyneuropathy | 5 | 156399950 | G/A | 0.669 | -0.107 | 0.081 | 0.183 | 0.331 | trans |
| EPCAM |  |  |  |  |  |  |  |  |  |  |
| rs56398830 | Diabetic polyneuropathy | 13 | 103701690 | A/G | 0.005 | 0.428 | 0.540 | 0.428 | 0.005 | trans |
| rs11142461 | Diabetic polyneuropathy | 9 | 71059889 | C/T | 0.025 | 0.098 | 0.246 | 0.690 | 0.025 | trans |
| rs2318997 | Diabetic polyneuropathy | 18 | 55529230 | G/T | 0.390 | 0.077 | 0.077 | 0.319 | 0.390 | trans |
| rs35853577 | Diabetic polyneuropathy | 2 | 233558879 | C/T | 0.333 | -0.012 | 0.080 | 0.878 | 0.333 | trans |
| rs2241764 | Diabetic polyneuropathy | 2 | 133174764 | T/C | 0.305 | -0.027 | 0.082 | 0.739 | 0.305 | trans |
| rs681343 | Diabetic polyneuropathy | 19 | 49206462 | T/C | 0.375 | -0.045 | 0.078 | 0.568 | 0.375 | trans |
| rs1713810 | Diabetic polyneuropathy | 3 | 153876826 | G/A | 0.821 | -0.048 | 0.099 | 0.631 | 0.179 | trans |
| rs9478784 | Diabetic polyneuropathy | 6 | 151020330 | T/C | 0.095 | -0.079 | 0.129 | 0.542 | 0.095 | trans |
| rs3746778 | Diabetic polyneuropathy | 20 | 61341472 | A/G | 0.327 | -0.142 | 0.082 | 0.081 | 0.327 | trans |
| rs1430780 | Diabetic polyneuropathy | 2 | 67878328 | C/T | 0.709 | -0.151 | 0.083 | 0.068 | 0.291 | trans |
| GPA33 |  |  |  |  |  |  |  |  |  |  |
| rs72689400 | Diabetic polyneuropathy | 1 | 167038219 | A/C | 0.020 | 0.460 | 0.268 | 0.086 | 0.020 | cis |
| rs56398830 | Diabetic polyneuropathy | 13 | 103701690 | A/G | 0.005 | 0.428 | 0.540 | 0.428 | 0.005 | trans |
| rs11142461 | Diabetic polyneuropathy | 9 | 71059889 | C/T | 0.025 | 0.098 | 0.246 | 0.690 | 0.025 | trans |
| rs2241764 | Diabetic polyneuropathy | 2 | 133174764 | T/C | 0.305 | -0.027 | 0.082 | 0.739 | 0.305 | trans |
| rs6437071 | Diabetic polyneuropathy | 2 | 233568793 | A/G | 0.336 | -0.037 | 0.080 | 0.648 | 0.336 | trans |
| rs681343 | Diabetic polyneuropathy | 19 | 49206462 | T/C | 0.375 | -0.045 | 0.078 | 0.568 | 0.375 | trans |
| rs9478784 | Diabetic polyneuropathy | 6 | 151020330 | T/C | 0.095 | -0.079 | 0.129 | 0.542 | 0.095 | trans |
| rs3746778 | Diabetic polyneuropathy | 20 | 61341472 | A/G | 0.327 | -0.142 | 0.082 | 0.081 | 0.327 | trans |
| ENSA |  |  |  |  |  |  |  |  |  |  |
| rs1354034 | Diabetic polyneuropathy | 3 | 56849749 | C/T | 0.707 | 0.052 | 0.083 | 0.532 | 0.293 | trans |
| rs111428433 | Diabetic polyneuropathy | 1 | 150527629 | T/C | 0.059 | -0.221 | 0.164 | 0.178 | 0.059 | cis |
| PCDH17 |  |  |  |  |  |  |  |  |  |  |
| rs8176749 | Diabetic polyneuropathy | 9 | 136131188 | T/C | 0.134 | 0.149 | 0.111 | 0.181 | 0.134 | trans |
| rs11148472 | Diabetic polyneuropathy | 13 | 60020189 | C/A | 0.319 | 0.043 | 0.081 | 0.595 | 0.319 | cis |
| rs5757683 | Diabetic polyneuropathy | 22 | 39850174 | A/G | 0.737 | 0.028 | 0.086 | 0.741 | 0.263 | trans |
| rs56278466 | Diabetic polyneuropathy | 10 | 17875857 | G/T | 0.581 | -0.003 | 0.077 | 0.970 | 0.419 | trans |
| rs3184504 | Diabetic polyneuropathy | 12 | 111884608 | C/T | 0.591 | -0.051 | 0.077 | 0.504 | 0.409 | trans |
| rs6789547 | Diabetic polyneuropathy | 3 | 58426047 | T/C | 0.168 | -0.138 | 0.101 | 0.173 | 0.168 | trans |
| rs11603123 | Diabetic polyneuropathy | 11 | 126305495 | A/G | 0.035 | -0.318 | 0.210 | 0.131 | 0.035 | trans |
| DUSP6 |  |  |  |  |  |  |  |  |  |  |
| rs111490052 | Diabetic polyneuropathy | 3 | 52077132 | G/A | 0.108 | 0.043 | 0.122 | 0.724 | 0.108 | trans |
| rs1355538 | Diabetic polyneuropathy | 3 | 165505177 | A/G | 0.326 | -0.043 | 0.080 | 0.595 | 0.326 | trans |
| LGALS3BP |  |  |  |  |  |  |  |  |  |  |
| rs111526614 | Diabetic polyneuropathy | 17 | 76962761 | T/C | 0.009 | -0.031 | 0.426 | 0.943 | 0.009 | cis |
| EDAR |  |  |  |  |  |  |  |  |  |  |
| rs140661471 | Diabetic polyneuropathy | 2 | 109603739 | A/G | 0.008 | 1.010 | 0.414 | 0.015 | 0.008 | cis |
| rs74035509 | Diabetic polyneuropathy | 16 | 88567333 | T/C | 0.053 | 0.181 | 0.169 | 0.283 | 0.053 | trans |
| rs11553699 | Diabetic polyneuropathy | 12 | 122216910 | G/A | 0.107 | 0.174 | 0.123 | 0.158 | 0.107 | trans |
| rs16937003 | Diabetic polyneuropathy | 10 | 80938499 | A/G | 0.019 | 0.173 | 0.275 | 0.529 | 0.019 | trans |
| rs12762934 | Diabetic polyneuropathy | 10 | 104359884 | T/C | 0.296 | 0.099 | 0.083 | 0.232 | 0.296 | trans |
| rs652963 | Diabetic polyneuropathy | 11 | 32911737 | T/C | 0.880 | 0.055 | 0.115 | 0.634 | 0.120 | trans |
| rs28362642 | Diabetic polyneuropathy | 3 | 39149503 | G/A | 0.072 | 0.031 | 0.144 | 0.831 | 0.072 | trans |
| rs114694170 | Diabetic polyneuropathy | 5 | 88180196 | C/T | 0.056 | 0.015 | 0.163 | 0.926 | 0.056 | trans |
| rs7080386 | Diabetic polyneuropathy | 10 | 65048306 | A/C | 0.384 | -0.001 | 0.078 | 0.993 | 0.384 | trans |
| rs111527738 | Diabetic polyneuropathy | 21 | 36259324 | G/A | 0.018 | -0.073 | 0.280 | 0.794 | 0.018 | trans |
| rs141232262 | Diabetic polyneuropathy | 14 | 68558737 | G/A | 0.104 | -0.083 | 0.124 | 0.501 | 0.104 | trans |
| rs12377089 | Diabetic polyneuropathy | 9 | 136941295 | G/A | 0.388 | -0.096 | 0.078 | 0.218 | 0.388 | trans |
| rs12712870 | Diabetic polyneuropathy | 2 | 43161180 | G/A | 0.725 | -0.098 | 0.085 | 0.246 | 0.275 | trans |
| rs72665955 | Diabetic polyneuropathy | 1 | 54841656 | A/G | 0.070 | -0.192 | 0.147 | 0.190 | 0.070 | trans |
| CHGB |  |  |  |  |  |  |  |  |  |  |
| rs2509902 | Diabetic polyneuropathy | 2 | 158514969 | C/T | 0.885 | 0.165 | 0.118 | 0.161 | 0.115 | trans |
| rs28688991 | Diabetic polyneuropathy | 16 | 20367550 | T/C | 0.233 | 0.062 | 0.090 | 0.487 | 0.233 | trans |
| rs236153 | Diabetic polyneuropathy | 20 | 5903894 | G/A | 0.418 | 0.038 | 0.076 | 0.616 | 0.418 | cis |
| rs13159918 | Diabetic polyneuropathy | 5 | 157892732 | A/C | 0.274 | -0.097 | 0.085 | 0.256 | 0.274 | trans |
| FUCA1 |  |  |  |  |  |  |  |  |  |  |
| rs4764823 | Diabetic polyneuropathy | 12 | 102219766 | A/G | 0.283 | 0.172 | 0.084 | 0.040 | 0.283 | trans |
| rs11155297 | Diabetic polyneuropathy | 6 | 143825104 | T/G | 0.206 | 0.079 | 0.093 | 0.396 | 0.206 | trans |
| rs13551 | Diabetic polyneuropathy | 1 | 24180962 | C/T | 0.396 | -0.063 | 0.077 | 0.413 | 0.396 | cis |
| FCAR |  |  |  |  |  |  |  |  |  |  |
| rs11155297 | Diabetic polyneuropathy | 6 | 143825104 | T/G | 0.206 | 0.079 | 0.093 | 0.396 | 0.206 | trans |
| rs6796 | Diabetic polyneuropathy | 7 | 6502367 | C/T | 0.321 | -0.010 | 0.082 | 0.900 | 0.321 | trans |
| rs3184504 | Diabetic polyneuropathy | 12 | 111884608 | C/T | 0.591 | -0.051 | 0.077 | 0.504 | 0.409 | trans |
| rs2070901 | Diabetic polyneuropathy | 1 | 161185058 | T/G | 0.283 | -0.089 | 0.084 | 0.291 | 0.283 | trans |
| rs4012248 | Diabetic polyneuropathy | 3 | 186779607 | A/G | 0.044 | -0.106 | 0.186 | 0.570 | 0.044 | trans |
| rs3826331 | Diabetic polyneuropathy | 17 | 38150492 | C/T | 0.599 | -0.116 | 0.077 | 0.131 | 0.401 | trans |
| rs73017385 | Diabetic polyneuropathy | 11 | 126179575 | A/G | 0.201 | -0.118 | 0.094 | 0.210 | 0.201 | trans |
| rs188468174 | Diabetic polyneuropathy | 1 | 25291697 | T/C | 0.004 | -0.166 | 0.540 | 0.758 | 0.004 | trans |
| RBM19 |  |  |  |  |  |  |  |  |  |  |
| rs11155297 | Diabetic polyneuropathy | 6 | 143825104 | T/G | 0.206 | 0.079 | 0.093 | 0.396 | 0.206 | trans |
| PDK2 |  |  |  |  |  |  |  |  |  |  |
| rs111564639 | Diabetic polyneuropathy | 17 | 48172095 | A/C | 0.044 | -0.078 | 0.188 | 0.678 | 0.044 | cis |
| NPTXR |  |  |  |  |  |  |  |  |  |  |
| rs12216891 | Diabetic polyneuropathy | 9 | 136127366 | T/C | 0.130 | 0.171 | 0.112 | 0.129 | 0.130 | trans |
| rs111577133 | Diabetic polyneuropathy | 22 | 39241262 | G/A | 0.045 | -0.068 | 0.185 | 0.712 | 0.045 | cis |
| TIRAP |  |  |  |  |  |  |  |  |  |  |
| rs62143198 | Diabetic polyneuropathy | 19 | 54320939 | A/G | 0.193 | 0.039 | 0.097 | 0.687 | 0.193 | trans |
| rs111577916 | Diabetic polyneuropathy | 11 | 126071349 | T/G | 0.037 | -0.072 | 0.203 | 0.725 | 0.037 | cis |
| VASH1 |  |  |  |  |  |  |  |  |  |  |
| rs1354034 | Diabetic polyneuropathy | 3 | 56849749 | C/T | 0.707 | 0.052 | 0.083 | 0.532 | 0.293 | trans |
| rs114694170 | Diabetic polyneuropathy | 5 | 88180196 | C/T | 0.056 | 0.015 | 0.163 | 0.926 | 0.056 | trans |
| rs17622656 | Diabetic polyneuropathy | 5 | 131820997 | A/G | 0.260 | -0.133 | 0.086 | 0.122 | 0.260 | trans |
| rs11159226 | Diabetic polyneuropathy | 14 | 77181432 | G/T | 0.101 | -0.150 | 0.127 | 0.236 | 0.101 | cis |
| CHIC2 |  |  |  |  |  |  |  |  |  |  |
| rs704 | Diabetic polyneuropathy | 17 | 26694861 | A/G | 0.420 | 0.049 | 0.076 | 0.519 | 0.420 | trans |
| rs11160165 | Diabetic polyneuropathy | 14 | 94689200 | T/G | 0.192 | -0.153 | 0.096 | 0.112 | 0.192 | trans |
| IGLL1 |  |  |  |  |  |  |  |  |  |  |
| rs75141179 | Diabetic polyneuropathy | 5 | 143274202 | T/C | 0.026 | 0.428 | 0.242 | 0.077 | 0.026 | trans |
| rs73217470 | Diabetic polyneuropathy | 13 | 72513420 | G/A | 0.021 | 0.371 | 0.259 | 0.153 | 0.021 | trans |
| rs76428106 | Diabetic polyneuropathy | 13 | 28604007 | C/T | 0.012 | 0.322 | 0.347 | 0.353 | 0.012 | trans |
| rs55737395 | Diabetic polyneuropathy | 19 | 33751349 | A/G | 0.325 | 0.129 | 0.081 | 0.108 | 0.325 | trans |
| rs1687391 | Diabetic polyneuropathy | 9 | 117090006 | T/C | 0.025 | 0.091 | 0.247 | 0.713 | 0.025 | trans |
| rs9939427 | Diabetic polyneuropathy | 16 | 86016091 | A/G | 0.195 | 0.047 | 0.096 | 0.624 | 0.195 | trans |
| rs79755767 | Diabetic polyneuropathy | 12 | 54698408 | A/G | 0.095 | 0.038 | 0.128 | 0.767 | 0.095 | trans |
| rs1433577 | Diabetic polyneuropathy | 8 | 130595881 | A/G | 0.266 | 0.020 | 0.085 | 0.819 | 0.266 | trans |
| rs9624216 | Diabetic polyneuropathy | 22 | 23922552 | A/G | 0.056 | 0.001 | 0.171 | 0.994 | 0.056 | cis |
| rs1538971 | Diabetic polyneuropathy | 1 | 161676394 | T/C | 0.338 | -0.019 | 0.080 | 0.810 | 0.338 | trans |
| rs116968179 | Diabetic polyneuropathy | 7 | 50189183 | A/G | 0.024 | -0.049 | 0.245 | 0.842 | 0.024 | trans |
| rs700588 | Diabetic polyneuropathy | 5 | 88108574 | A/G | 0.324 | -0.091 | 0.081 | 0.258 | 0.324 | trans |
| rs111672628 | Diabetic polyneuropathy | 5 | 158256903 | G/A | 0.056 | -0.220 | 0.166 | 0.186 | 0.056 | trans |
| rs76529516 | Diabetic polyneuropathy | 2 | 60583370 | G/A | 0.012 | -0.226 | 0.343 | 0.511 | 0.012 | trans |
| ENDOU |  |  |  |  |  |  |  |  |  |  |
| rs11168216 | Diabetic polyneuropathy | 12 | 48132176 | T/C | 0.750 | -0.123 | 0.088 | 0.163 | 0.250 | cis |
| BCHE |  |  |  |  |  |  |  |  |  |  |
| rs111696008 | Diabetic polyneuropathy | 2 | 217487709 | A/G | 0.014 | 0.181 | 0.331 | 0.584 | 0.014 | trans |
| rs56768485 | Diabetic polyneuropathy | 1 | 201952598 | A/G | 0.340 | 0.072 | 0.080 | 0.365 | 0.340 | trans |
| rs17713088 | Diabetic polyneuropathy | 3 | 165488604 | T/G | 0.174 | -0.015 | 0.099 | 0.878 | 0.174 | trans |
| rs887829 | Diabetic polyneuropathy | 2 | 234668570 | T/C | 0.393 | -0.077 | 0.077 | 0.318 | 0.393 | trans |
| CEP43 |  |  |  |  |  |  |  |  |  |  |
| rs3811444 | Diabetic polyneuropathy | 1 | 248039451 | T/C | 0.346 | -0.037 | 0.080 | 0.645 | 0.346 | trans |
| rs56279106 | Diabetic polyneuropathy | 1 | 180103904 | A/G | 0.277 | -0.052 | 0.085 | 0.540 | 0.277 | trans |
| CCN3 |  |  |  |  |  |  |  |  |  |  |
| rs7827593 | Diabetic polyneuropathy | 8 | 134024462 | A/G | 0.090 | -0.005 | 0.131 | 0.967 | 0.090 | trans |
| rs11172113 | Diabetic polyneuropathy | 12 | 57527283 | C/T | 0.400 | -0.093 | 0.077 | 0.225 | 0.400 | trans |
| rs2279112 | Diabetic polyneuropathy | 8 | 120429024 | A/G | 0.203 | -0.121 | 0.094 | 0.196 | 0.203 | cis |
| SSC4D |  |  |  |  |  |  |  |  |  |  |
| rs1800961 | Diabetic polyneuropathy | 20 | 43042364 | T/C | 0.045 | 0.445 | 0.180 | 0.013 | 0.045 | trans |
| rs12540573 | Diabetic polyneuropathy | 7 | 76039013 | C/A | 0.042 | 0.043 | 0.187 | 0.819 | 0.042 | cis |
| rs2739344 | Diabetic polyneuropathy | 22 | 24298293 | C/A | 0.620 | 0.036 | 0.079 | 0.644 | 0.380 | trans |
| rs112635299 | Diabetic polyneuropathy | 14 | 94838142 | T/G | 0.020 | -0.014 | 0.271 | 0.960 | 0.020 | trans |
| rs165316 | Diabetic polyneuropathy | 1 | 91533297 | G/A | 0.213 | -0.021 | 0.093 | 0.820 | 0.213 | trans |
| rs4803852 | Diabetic polyneuropathy | 19 | 46238926 | T/G | 0.777 | -0.057 | 0.091 | 0.527 | 0.223 | trans |
| rs12140070 | Diabetic polyneuropathy | 1 | 28335581 | G/A | 0.323 | -0.084 | 0.081 | 0.299 | 0.323 | trans |
| SIGLEC1 |  |  |  |  |  |  |  |  |  |  |
| rs11668950 | Diabetic polyneuropathy | 19 | 18282940 | A/G | 0.252 | 0.220 | 0.087 | 0.011 | 0.252 | trans |
| rs62165726 | Diabetic polyneuropathy | 2 | 134966562 | A/C | 0.026 | 0.074 | 0.243 | 0.760 | 0.026 | trans |
| rs2111485 | Diabetic polyneuropathy | 2 | 163110536 | G/A | 0.582 | 0.048 | 0.077 | 0.534 | 0.418 | trans |
| rs11185602 | Diabetic polyneuropathy | 7 | 50299077 | G/A | 0.313 | 0.029 | 0.081 | 0.725 | 0.313 | trans |
| rs3184504 | Diabetic polyneuropathy | 12 | 111884608 | C/T | 0.591 | -0.051 | 0.077 | 0.504 | 0.409 | trans |
| rs2031902 | Diabetic polyneuropathy | 9 | 33117524 | T/C | 0.624 | -0.117 | 0.078 | 0.136 | 0.376 | trans |
| rs17849502 | Diabetic polyneuropathy | 1 | 183532580 | T/G | 0.038 | -0.150 | 0.198 | 0.451 | 0.038 | trans |
| LY9 |  |  |  |  |  |  |  |  |  |  |
| rs76428106 | Diabetic polyneuropathy | 13 | 28604007 | C/T | 0.012 | 0.322 | 0.347 | 0.353 | 0.012 | trans |
| rs35829610 | Diabetic polyneuropathy | 5 | 131823862 | CTG/C | 0.330 | 0.095 | 0.081 | 0.239 | 0.330 | trans |
| rs2009581 | Diabetic polyneuropathy | 2 | 111807677 | A/G | 0.264 | 0.088 | 0.086 | 0.302 | 0.264 | trans |
| rs11185602 | Diabetic polyneuropathy | 7 | 50299077 | G/A | 0.313 | 0.029 | 0.081 | 0.725 | 0.313 | trans |
| rs9824474 | Diabetic polyneuropathy | 3 | 169745527 | G/A | 0.394 | -0.004 | 0.077 | 0.963 | 0.394 | trans |
| rs3184504 | Diabetic polyneuropathy | 12 | 111884608 | C/T | 0.591 | -0.051 | 0.077 | 0.504 | 0.409 | trans |
| rs7634389 | Diabetic polyneuropathy | 3 | 186738421 | C/T | 0.398 | -0.067 | 0.077 | 0.387 | 0.398 | trans |
| rs37455 | Diabetic polyneuropathy | 1 | 44294228 | G/A | 0.361 | -0.089 | 0.079 | 0.258 | 0.361 | trans |
| rs75071241 | Diabetic polyneuropathy | 11 | 126232186 | A/G | 0.071 | -0.090 | 0.148 | 0.544 | 0.071 | trans |
| rs188468174 | Diabetic polyneuropathy | 1 | 25291697 | T/C | 0.004 | -0.166 | 0.540 | 0.758 | 0.004 | trans |
| SLC5A5 |  |  |  |  |  |  |  |  |  |  |
| rs111874634 | Diabetic polyneuropathy | 19 | 35835021 | T/C | 0.187 | 0.085 | 0.097 | 0.378 | 0.187 | trans |
| KAZALD1 |  |  |  |  |  |  |  |  |  |  |
| rs11190812 | Diabetic polyneuropathy | 10 | 102824292 | A/G | 0.079 | -0.069 | 0.141 | 0.625 | 0.079 | cis |
| rs2228243 | Diabetic polyneuropathy | 3 | 186395113 | G/A | 0.159 | -0.091 | 0.103 | 0.379 | 0.159 | trans |
| LAIR1 |  |  |  |  |  |  |  |  |  |  |
| rs333947 | Diabetic polyneuropathy | 1 | 110470764 | A/G | 0.167 | 0.083 | 0.102 | 0.420 | 0.167 | trans |
| rs77924615 | Diabetic polyneuropathy | 16 | 20392332 | A/G | 0.222 | 0.042 | 0.091 | 0.643 | 0.222 | trans |
| rs3184504 | Diabetic polyneuropathy | 12 | 111884608 | C/T | 0.591 | -0.051 | 0.077 | 0.504 | 0.409 | trans |
| DKKL1 |  |  |  |  |  |  |  |  |  |  |
| rs112001035 | Diabetic polyneuropathy | 17 | 66823805 | A/G | 0.081 | 0.352 | 0.140 | 0.012 | 0.081 | trans |
| rs12149545 | Diabetic polyneuropathy | 16 | 56993161 | A/G | 0.276 | 0.054 | 0.085 | 0.523 | 0.276 | trans |
| rs2303759 | Diabetic polyneuropathy | 19 | 49869051 | G/T | 0.171 | -0.137 | 0.100 | 0.170 | 0.171 | cis |
| rs149615216 | Diabetic polyneuropathy | 18 | 47106028 | T/C | 0.005 | -0.778 | 0.516 | 0.132 | 0.005 | trans |
| CCL25 |  |  |  |  |  |  |  |  |  |  |
| rs112001035 | Diabetic polyneuropathy | 17 | 66823805 | A/G | 0.081 | 0.352 | 0.140 | 0.012 | 0.081 | trans |
| rs2032887 | Diabetic polyneuropathy | 19 | 8121360 | G/A | 0.275 | 0.137 | 0.085 | 0.105 | 0.275 | cis |
| rs118062058 | Diabetic polyneuropathy | 8 | 42103378 | G/A | 0.118 | 0.045 | 0.118 | 0.700 | 0.118 | trans |
| rs601338 | Diabetic polyneuropathy | 19 | 49206674 | G/A | 0.626 | 0.044 | 0.078 | 0.571 | 0.375 | trans |
| rs7295693 | Diabetic polyneuropathy | 12 | 576796 | C/T | 0.594 | -0.059 | 0.077 | 0.444 | 0.406 | trans |
| PLA2G10 |  |  |  |  |  |  |  |  |  |  |
| rs112001035 | Diabetic polyneuropathy | 17 | 66823805 | A/G | 0.081 | 0.352 | 0.140 | 0.012 | 0.081 | trans |
| rs75278536 | Diabetic polyneuropathy | 8 | 19821425 | G/T | 0.091 | 0.054 | 0.130 | 0.677 | 0.091 | trans |
| rs12136083 | Diabetic polyneuropathy | 1 | 63163073 | C/T | 0.251 | -0.014 | 0.087 | 0.871 | 0.251 | trans |
| rs1260326 | Diabetic polyneuropathy | 2 | 27730940 | T/C | 0.351 | -0.100 | 0.079 | 0.208 | 0.351 | trans |
| rs533617 | Diabetic polyneuropathy | 2 | 21233972 | C/T | 0.067 | -0.207 | 0.152 | 0.173 | 0.067 | trans |
| ANTXR2 |  |  |  |  |  |  |  |  |  |  |
| rs112001035 | Diabetic polyneuropathy | 17 | 66823805 | A/G | 0.081 | 0.352 | 0.140 | 0.012 | 0.081 | trans |
| rs2070895 | Diabetic polyneuropathy | 15 | 58723939 | A/G | 0.258 | 0.006 | 0.087 | 0.949 | 0.258 | trans |
| rs6073958 | Diabetic polyneuropathy | 20 | 44551855 | C/T | 0.176 | 0.002 | 0.099 | 0.985 | 0.176 | trans |
| rs7674623 | Diabetic polyneuropathy | 4 | 80794681 | T/C | 0.166 | -0.071 | 0.103 | 0.486 | 0.166 | cis |
| rs1260326 | Diabetic polyneuropathy | 2 | 27730940 | T/C | 0.351 | -0.100 | 0.079 | 0.208 | 0.351 | trans |
| KLB |  |  |  |  |  |  |  |  |  |  |
| rs112001035 | Diabetic polyneuropathy | 17 | 66823805 | A/G | 0.081 | 0.352 | 0.140 | 0.012 | 0.081 | trans |
| rs35254320 | Diabetic polyneuropathy | 12 | 21038869 | T/C | 0.109 | 0.096 | 0.120 | 0.423 | 0.109 | trans |
| rs60134803 | Diabetic polyneuropathy | 17 | 9600728 | G/A | 0.253 | 0.016 | 0.087 | 0.857 | 0.253 | trans |
| rs28929474 | Diabetic polyneuropathy | 14 | 94844947 | T/C | 0.020 | -0.002 | 0.272 | 0.993 | 0.020 | trans |
| rs897764 | Diabetic polyneuropathy | 19 | 35559624 | C/T | 0.939 | -0.010 | 0.159 | 0.952 | 0.061 | trans |
| rs13108218 | Diabetic polyneuropathy | 4 | 3443931 | G/A | 0.677 | -0.059 | 0.081 | 0.469 | 0.323 | trans |
| rs13103023 | Diabetic polyneuropathy | 4 | 39457617 | A/G | 0.373 | -0.092 | 0.078 | 0.239 | 0.373 | cis |
| rs3135911 | Diabetic polyneuropathy | 5 | 176513896 | A/C | 0.333 | -0.118 | 0.080 | 0.143 | 0.333 | trans |
| PIK3AP1 |  |  |  |  |  |  |  |  |  |  |
| rs1354034 | Diabetic polyneuropathy | 3 | 56849749 | C/T | 0.707 | 0.052 | 0.083 | 0.532 | 0.293 | trans |
| rs41317268 | Diabetic polyneuropathy | 10 | 98469114 | G/A | 0.208 | -0.135 | 0.094 | 0.151 | 0.208 | cis |
| TGFBR3 |  |  |  |  |  |  |  |  |  |  |
| rs181242111 | Diabetic polyneuropathy | 10 | 17865664 | A/G | 0.088 | 0.206 | 0.134 | 0.126 | 0.088 | trans |
| rs150816167 | Diabetic polyneuropathy | 1 | 179571862 | C/T | 0.064 | -0.189 | 0.158 | 0.233 | 0.064 | trans |
| ANGPTL3 |  |  |  |  |  |  |  |  |  |  |
| rs11207970 | Diabetic polyneuropathy | 1 | 62915473 | T/C | 0.264 | 0.048 | 0.086 | 0.572 | 0.264 | cis |
| ACAT1 |  |  |  |  |  |  |  |  |  |  |
| rs112087419 | Diabetic polyneuropathy | 11 | 107998126 | A/G | 0.169 | 0.039 | 0.101 | 0.702 | 0.169 | cis |
| IL23R |  |  |  |  |  |  |  |  |  |  |
| rs11209026 | Diabetic polyneuropathy | 1 | 67705958 | A/G | 0.046 | 0.049 | 0.184 | 0.792 | 0.046 | cis |
| NCAM1 |  |  |  |  |  |  |  |  |  |  |
| rs11214489 | Diabetic polyneuropathy | 11 | 112975934 | C/T | 0.753 | -0.092 | 0.087 | 0.294 | 0.248 | cis |
| CD79B |  |  |  |  |  |  |  |  |  |  |
| rs76428106 | Diabetic polyneuropathy | 13 | 28604007 | C/T | 0.012 | 0.322 | 0.347 | 0.353 | 0.012 | trans |
| rs55654737 | Diabetic polyneuropathy | 5 | 179218842 | A/G | 0.345 | 0.117 | 0.079 | 0.141 | 0.345 | trans |
| rs112151347 | Diabetic polyneuropathy | 2 | 227695446 | A/G | 0.026 | 0.078 | 0.245 | 0.751 | 0.026 | trans |
| rs4674 | Diabetic polyneuropathy | 19 | 41930396 | G/A | 0.645 | 0.056 | 0.079 | 0.479 | 0.355 | trans |
| rs9939427 | Diabetic polyneuropathy | 16 | 86016091 | A/G | 0.195 | 0.047 | 0.096 | 0.624 | 0.195 | trans |
| rs2395904 | Diabetic polyneuropathy | 8 | 130617819 | G/T | 0.267 | 0.046 | 0.085 | 0.593 | 0.267 | trans |
| rs78444298 | Diabetic polyneuropathy | 1 | 184672098 | A/G | 0.017 | 0.023 | 0.295 | 0.937 | 0.017 | trans |
| rs7087507 | Diabetic polyneuropathy | 10 | 63745689 | G/A | 0.271 | 0.020 | 0.085 | 0.811 | 0.271 | trans |
| rs507666 | Diabetic polyneuropathy | 9 | 136149399 | A/G | 0.201 | 0.019 | 0.094 | 0.840 | 0.201 | trans |
| rs12946669 | Diabetic polyneuropathy | 17 | 62006007 | T/C | 0.369 | -0.033 | 0.078 | 0.676 | 0.369 | cis |
| rs77397204 | Diabetic polyneuropathy | 3 | 186738899 | C/A | 0.398 | -0.066 | 0.077 | 0.390 | 0.398 | trans |
| rs72704449 | Diabetic polyneuropathy | 1 | 179473273 | C/T | 0.065 | -0.202 | 0.157 | 0.197 | 0.065 | trans |
| rs112824187 | Diabetic polyneuropathy | 5 | 158265308 | A/G | 0.056 | -0.219 | 0.167 | 0.188 | 0.056 | trans |
| CADM1 |  |  |  |  |  |  |  |  |  |  |
| rs11215406 | Diabetic polyneuropathy | 11 | 115065082 | C/T | 0.267 | -0.045 | 0.085 | 0.600 | 0.267 | cis |
| rs72704449 | Diabetic polyneuropathy | 1 | 179473273 | C/T | 0.065 | -0.202 | 0.157 | 0.197 | 0.065 | trans |
| FUT3_FUT5 |  |  |  |  |  |  |  |  |  |  |
| rs9738365 | Diabetic polyneuropathy | 12 | 31997635 | A/C | 0.280 | 0.050 | 0.084 | 0.552 | 0.280 | trans |
| rs492602 | Diabetic polyneuropathy | 19 | 49206417 | A/G | 0.625 | 0.045 | 0.078 | 0.566 | 0.375 | trans |
| rs62542743 | Diabetic polyneuropathy | 9 | 33385241 | A/C | 0.049 | 0.012 | 0.177 | 0.946 | 0.049 | trans |
| rs58542926 | Diabetic polyneuropathy | 19 | 19379549 | T/C | 0.064 | 0.005 | 0.153 | 0.977 | 0.064 | trans |
| rs56278466 | Diabetic polyneuropathy | 10 | 17875857 | G/T | 0.581 | -0.003 | 0.077 | 0.970 | 0.419 | trans |
| rs178765 | Diabetic polyneuropathy | 14 | 23706738 | A/G | 0.246 | -0.030 | 0.089 | 0.732 | 0.246 | trans |
| rs3760775 | Diabetic polyneuropathy | 19 | 5841356 | T/G | 0.108 | -0.031 | 0.123 | 0.803 | 0.108 | cis |
| rs2106854 | Diabetic polyneuropathy | 5 | 131769174 | T/C | 0.222 | -0.083 | 0.091 | 0.364 | 0.222 | trans |
| rs2393791 | Diabetic polyneuropathy | 12 | 121423956 | T/C | 0.584 | -0.089 | 0.077 | 0.246 | 0.416 | trans |
| rs112158477 | Diabetic polyneuropathy | 12 | 111766446 | T/C | 0.115 | -0.095 | 0.118 | 0.422 | 0.115 | trans |
| TFRC |  |  |  |  |  |  |  |  |  |  |
| rs74035509 | Diabetic polyneuropathy | 16 | 88567333 | T/C | 0.053 | 0.181 | 0.169 | 0.283 | 0.053 | trans |
| rs550057 | Diabetic polyneuropathy | 9 | 136146597 | T/C | 0.302 | 0.128 | 0.082 | 0.121 | 0.302 | trans |
| rs6592965 | Diabetic polyneuropathy | 7 | 50427982 | A/G | 0.321 | 0.011 | 0.081 | 0.894 | 0.321 | trans |
| rs11216316 | Diabetic polyneuropathy | 11 | 117081500 | C/A | 0.134 | -0.051 | 0.111 | 0.645 | 0.134 | trans |
| rs252152 | Diabetic polyneuropathy | 5 | 141445774 | G/A | 0.677 | -0.054 | 0.081 | 0.508 | 0.323 | trans |
| VWA1 |  |  |  |  |  |  |  |  |  |  |
| rs112164771 | Diabetic polyneuropathy | 9 | 117091002 | T/C | 0.025 | 0.096 | 0.247 | 0.698 | 0.025 | trans |
| rs28546127 | Diabetic polyneuropathy | 1 | 22200473 | T/C | 0.034 | -0.110 | 0.207 | 0.595 | 0.034 | trans |
| rs115503338 | Diabetic polyneuropathy | 1 | 1369623 | A/G | 0.008 | -0.159 | 0.423 | 0.708 | 0.008 | cis |
| OMG |  |  |  |  |  |  |  |  |  |  |
| rs17196752 | Diabetic polyneuropathy | 20 | 48887268 | T/C | 0.233 | 0.073 | 0.089 | 0.411 | 0.233 | trans |
| rs56278466 | Diabetic polyneuropathy | 10 | 17875857 | G/T | 0.581 | -0.003 | 0.077 | 0.970 | 0.419 | trans |
| rs72813607 | Diabetic polyneuropathy | 17 | 29461525 | A/G | 0.105 | -0.067 | 0.122 | 0.586 | 0.105 | cis |
| rs112168180 | Diabetic polyneuropathy | 5 | 126394937 | T/C | 0.025 | -0.120 | 0.240 | 0.617 | 0.025 | trans |
| rs3757724 | Diabetic polyneuropathy | 7 | 20395067 | T/C | 0.082 | -0.324 | 0.140 | 0.020 | 0.082 | trans |
| GP1BA |  |  |  |  |  |  |  |  |  |  |
| rs78565404 | Diabetic polyneuropathy | 3 | 184090242 | T/C | 0.026 | 0.143 | 0.237 | 0.548 | 0.026 | trans |
| rs12762934 | Diabetic polyneuropathy | 10 | 104359884 | T/C | 0.296 | 0.099 | 0.083 | 0.232 | 0.296 | trans |
| rs7176023 | Diabetic polyneuropathy | 15 | 65101007 | C/T | 0.078 | 0.069 | 0.141 | 0.622 | 0.078 | trans |
| rs3767809 | Diabetic polyneuropathy | 1 | 118154831 | C/T | 0.218 | 0.058 | 0.091 | 0.525 | 0.218 | trans |
| rs1555405 | Diabetic polyneuropathy | 14 | 101176769 | A/G | 0.353 | 0.045 | 0.079 | 0.571 | 0.353 | trans |
| rs11217176 | Diabetic polyneuropathy | 11 | 119065044 | A/G | 0.298 | 0.038 | 0.082 | 0.642 | 0.298 | trans |
| rs1434282 | Diabetic polyneuropathy | 1 | 199010721 | T/C | 0.711 | 0.030 | 0.083 | 0.720 | 0.290 | trans |
| rs3804749 | Diabetic polyneuropathy | 3 | 122833003 | T/C | 0.620 | 0.007 | 0.078 | 0.930 | 0.380 | trans |
| rs3184504 | Diabetic polyneuropathy | 12 | 111884608 | C/T | 0.591 | -0.051 | 0.077 | 0.504 | 0.409 | trans |
| rs2853677 | Diabetic polyneuropathy | 5 | 1287194 | A/G | 0.626 | -0.060 | 0.078 | 0.446 | 0.375 | trans |
| rs6961069 | Diabetic polyneuropathy | 7 | 80218961 | T/C | 0.417 | -0.076 | 0.077 | 0.324 | 0.417 | trans |
| rs35786788 | Diabetic polyneuropathy | 6 | 135419042 | A/G | 0.340 | -0.093 | 0.080 | 0.246 | 0.340 | trans |
| HSPA1A |  |  |  |  |  |  |  |  |  |  |
| rs11218968 | Diabetic polyneuropathy | 11 | 122958478 | G/A | 0.038 | -0.330 | 0.196 | 0.092 | 0.038 | trans |
| MCAM |  |  |  |  |  |  |  |  |  |  |
| rs8176693 | Diabetic polyneuropathy | 9 | 136137657 | T/C | 0.134 | 0.135 | 0.111 | 0.223 | 0.134 | trans |
| rs13107325 | Diabetic polyneuropathy | 4 | 103188709 | T/C | 0.014 | 0.106 | 0.309 | 0.731 | 0.014 | trans |
| rs41289902 | Diabetic polyneuropathy | 6 | 112460365 | T/C | 0.022 | 0.007 | 0.260 | 0.980 | 0.022 | trans |
| rs11220462 | Diabetic polyneuropathy | 11 | 126243952 | A/G | 0.188 | -0.026 | 0.098 | 0.794 | 0.188 | trans |
| rs1260326 | Diabetic polyneuropathy | 2 | 27730940 | T/C | 0.351 | -0.100 | 0.079 | 0.208 | 0.351 | trans |
| rs34587557 | Diabetic polyneuropathy | 11 | 119185677 | C/T | 0.046 | -0.107 | 0.182 | 0.556 | 0.046 | cis |
| SIGLEC7 |  |  |  |  |  |  |  |  |  |  |
| rs11721064 | Diabetic polyneuropathy | 3 | 98408826 | T/G | 0.673 | -0.002 | 0.080 | 0.985 | 0.328 | trans |
| rs11220462 | Diabetic polyneuropathy | 11 | 126243952 | A/G | 0.188 | -0.026 | 0.098 | 0.794 | 0.188 | trans |
| rs3184504 | Diabetic polyneuropathy | 12 | 111884608 | C/T | 0.591 | -0.051 | 0.077 | 0.504 | 0.409 | trans |
| rs4393849 | Diabetic polyneuropathy | 3 | 194478411 | G/A | 0.340 | -0.147 | 0.080 | 0.067 | 0.340 | trans |
| ERBB4 |  |  |  |  |  |  |  |  |  |  |
| rs72835417 | Diabetic polyneuropathy | 17 | 47241642 | A/G | 0.133 | 0.088 | 0.113 | 0.433 | 0.133 | trans |
| rs4665972 | Diabetic polyneuropathy | 2 | 27598097 | C/T | 0.623 | 0.080 | 0.078 | 0.303 | 0.377 | trans |
| rs2519093 | Diabetic polyneuropathy | 9 | 136141870 | T/C | 0.201 | 0.028 | 0.094 | 0.762 | 0.201 | trans |
| rs56278466 | Diabetic polyneuropathy | 10 | 17875857 | G/T | 0.581 | -0.003 | 0.077 | 0.970 | 0.419 | trans |
| rs11220465 | Diabetic polyneuropathy | 11 | 126257779 | A/G | 0.225 | -0.046 | 0.091 | 0.616 | 0.225 | trans |
| rs35383942 | Diabetic polyneuropathy | 1 | 201437832 | T/C | 0.116 | -0.046 | 0.120 | 0.699 | 0.116 | trans |
| rs9787076 | Diabetic polyneuropathy | 1 | 44141149 | C/A | 0.349 | -0.048 | 0.079 | 0.545 | 0.349 | trans |
| rs485073 | Diabetic polyneuropathy | 19 | 49207255 | G/A | 0.416 | -0.077 | 0.077 | 0.313 | 0.416 | trans |
| rs7324484 | Diabetic polyneuropathy | 13 | 48648079 | A/G | 0.666 | -0.098 | 0.080 | 0.217 | 0.334 | trans |
| rs2541593 | Diabetic polyneuropathy | 16 | 103423 | A/C | 0.788 | -0.148 | 0.093 | 0.111 | 0.212 | trans |
| SPINK5 |  |  |  |  |  |  |  |  |  |  |
| rs2479016 | Diabetic polyneuropathy | 6 | 2245345 | G/A | 0.378 | 0.154 | 0.078 | 0.049 | 0.378 | trans |
| rs11384422 | Diabetic polyneuropathy | 2 | 60499861 | AG/A | 0.622 | 0.041 | 0.078 | 0.599 | 0.378 | trans |
| rs6883868 | Diabetic polyneuropathy | 5 | 147500883 | T/C | 0.636 | 0.032 | 0.079 | 0.683 | 0.364 | cis |
| rs681343 | Diabetic polyneuropathy | 19 | 49206462 | T/C | 0.375 | -0.045 | 0.078 | 0.568 | 0.375 | trans |
| rs1260326 | Diabetic polyneuropathy | 2 | 27730940 | T/C | 0.351 | -0.100 | 0.079 | 0.208 | 0.351 | trans |
| rs11220477 | Diabetic polyneuropathy | 11 | 126275402 | T/C | 0.046 | -0.114 | 0.183 | 0.534 | 0.046 | trans |
| KLK12 |  |  |  |  |  |  |  |  |  |  |
| rs2479016 | Diabetic polyneuropathy | 6 | 2245345 | G/A | 0.378 | 0.154 | 0.078 | 0.049 | 0.378 | trans |
| rs79744308 | Diabetic polyneuropathy | 19 | 5827765 | A/G | 0.030 | -0.015 | 0.220 | 0.945 | 0.030 | trans |
| rs11220477 | Diabetic polyneuropathy | 11 | 126275402 | T/C | 0.046 | -0.114 | 0.183 | 0.534 | 0.046 | trans |
| LYPD8 |  |  |  |  |  |  |  |  |  |  |
| rs4760 | Diabetic polyneuropathy | 19 | 44153100 | G/A | 0.168 | 0.087 | 0.103 | 0.396 | 0.168 | trans |
| rs11220520 | Diabetic polyneuropathy | 11 | 126357908 | T/C | 0.216 | 0.057 | 0.092 | 0.535 | 0.216 | trans |
| rs4556017 | Diabetic polyneuropathy | 7 | 100632790 | C/T | 0.179 | 0.007 | 0.100 | 0.942 | 0.179 | trans |
| rs12044252 | Diabetic polyneuropathy | 1 | 249065691 | C/T | 0.064 | -0.089 | 0.151 | 0.556 | 0.064 | cis |
| rs708686 | Diabetic polyneuropathy | 19 | 5840619 | T/C | 0.334 | -0.105 | 0.081 | 0.194 | 0.334 | trans |
| TBC1D5 |  |  |  |  |  |  |  |  |  |  |
| rs1354034 | Diabetic polyneuropathy | 3 | 56849749 | C/T | 0.707 | 0.052 | 0.083 | 0.532 | 0.293 | trans |
| rs11227136 | Diabetic polyneuropathy | 11 | 64931964 | A/G | 0.224 | -0.107 | 0.091 | 0.238 | 0.224 | trans |
| CA14 |  |  |  |  |  |  |  |  |  |  |
| rs61431557 | Diabetic polyneuropathy | 6 | 34717578 | T/C | 0.201 | 0.062 | 0.096 | 0.519 | 0.201 | trans |
| SCGB3A1 |  |  |  |  |  |  |  |  |  |  |
| rs307802 | Diabetic polyneuropathy | 5 | 180019237 | T/C | 0.659 | 0.090 | 0.080 | 0.260 | 0.341 | cis |
| rs61703905 | Diabetic polyneuropathy | 19 | 46370301 | T/C | 0.149 | -0.055 | 0.106 | 0.606 | 0.149 | trans |
| HOMER2 |  |  |  |  |  |  |  |  |  |  |
| rs1354034 | Diabetic polyneuropathy | 3 | 56849749 | C/T | 0.707 | 0.052 | 0.083 | 0.532 | 0.293 | trans |
| rs13053850 | Diabetic polyneuropathy | 22 | 51108509 | A/G | 0.419 | -0.010 | 0.077 | 0.902 | 0.419 | trans |
| rs116052829 | Diabetic polyneuropathy | 10 | 81164146 | T/C | 0.056 | -0.087 | 0.166 | 0.601 | 0.056 | trans |
| rs1256430 | Diabetic polyneuropathy | 15 | 83519472 | A/G | 0.142 | -0.133 | 0.108 | 0.219 | 0.142 | trans |
| FSTL3 |  |  |  |  |  |  |  |  |  |  |
| rs112418024 | Diabetic polyneuropathy | 19 | 692790 | A/G | 0.066 | -0.024 | 0.155 | 0.875 | 0.066 | cis |
| rs1260326 | Diabetic polyneuropathy | 2 | 27730940 | T/C | 0.351 | -0.100 | 0.079 | 0.208 | 0.351 | trans |
| SPARC |  |  |  |  |  |  |  |  |  |  |
| rs1654425 | Diabetic polyneuropathy | 19 | 55538980 | C/T | 0.879 | 0.126 | 0.115 | 0.272 | 0.121 | trans |
| rs28734133 | Diabetic polyneuropathy | 16 | 81874035 | G/A | 0.166 | 0.103 | 0.102 | 0.309 | 0.166 | trans |
| rs2127869 | Diabetic polyneuropathy | 14 | 65794352 | C/T | 0.708 | 0.061 | 0.083 | 0.461 | 0.292 | trans |
| rs1354034 | Diabetic polyneuropathy | 3 | 56849749 | C/T | 0.707 | 0.052 | 0.083 | 0.532 | 0.293 | trans |
| rs1608696 | Diabetic polyneuropathy | 6 | 71329265 | C/T | 0.333 | -0.002 | 0.080 | 0.982 | 0.333 | trans |
| rs34377578 | Diabetic polyneuropathy | 10 | 104336426 | A/C | 0.804 | -0.034 | 0.095 | 0.723 | 0.196 | trans |
| rs3790176 | Diabetic polyneuropathy | 20 | 19261922 | A/G | 0.290 | -0.058 | 0.083 | 0.487 | 0.290 | trans |
| rs11242109 | Diabetic polyneuropathy | 5 | 131677047 | T/G | 0.345 | -0.065 | 0.079 | 0.414 | 0.345 | trans |
| rs6961069 | Diabetic polyneuropathy | 7 | 80218961 | T/C | 0.417 | -0.076 | 0.077 | 0.324 | 0.417 | trans |
| rs7618405 | Diabetic polyneuropathy | 3 | 18250509 | A/C | 0.211 | -0.178 | 0.093 | 0.055 | 0.211 | trans |
| rs13412535 | Diabetic polyneuropathy | 2 | 224874874 | A/G | 0.205 | -0.204 | 0.095 | 0.032 | 0.205 | trans |
| CD63 |  |  |  |  |  |  |  |  |  |  |
| rs113422568 | Diabetic polyneuropathy | 10 | 104337938 | A/G | 0.297 | 0.106 | 0.083 | 0.201 | 0.297 | trans |
| rs4813637 | Diabetic polyneuropathy | 20 | 3682762 | A/G | 0.661 | 0.030 | 0.080 | 0.710 | 0.339 | trans |
| rs2278668 | Diabetic polyneuropathy | 3 | 122835232 | C/T | 0.619 | -0.009 | 0.078 | 0.907 | 0.381 | trans |
| rs12445050 | Diabetic polyneuropathy | 16 | 81870969 | T/C | 0.130 | -0.033 | 0.113 | 0.769 | 0.130 | trans |
| rs11242109 | Diabetic polyneuropathy | 5 | 131677047 | T/G | 0.345 | -0.065 | 0.079 | 0.414 | 0.345 | trans |
| rs892090 | Diabetic polyneuropathy | 19 | 55539072 | T/G | 0.121 | -0.126 | 0.115 | 0.272 | 0.121 | trans |
| rs35496032 | Diabetic polyneuropathy | 19 | 38817628 | A/G | 0.005 | -0.706 | 0.491 | 0.150 | 0.005 | trans |
| SERPINE1 |  |  |  |  |  |  |  |  |  |  |
| rs2227674 | Diabetic polyneuropathy | 7 | 100776208 | G/A | 0.179 | 0.226 | 0.098 | 0.022 | 0.179 | cis |
| rs1654425 | Diabetic polyneuropathy | 19 | 55538980 | C/T | 0.879 | 0.126 | 0.115 | 0.272 | 0.121 | trans |
| rs1354034 | Diabetic polyneuropathy | 3 | 56849749 | C/T | 0.707 | 0.052 | 0.083 | 0.532 | 0.293 | trans |
| rs2274319 | Diabetic polyneuropathy | 1 | 156450873 | C/T | 0.581 | 0.047 | 0.076 | 0.542 | 0.419 | trans |
| rs114694170 | Diabetic polyneuropathy | 5 | 88180196 | C/T | 0.056 | 0.015 | 0.163 | 0.926 | 0.056 | trans |
| rs12445050 | Diabetic polyneuropathy | 16 | 81870969 | T/C | 0.130 | -0.033 | 0.113 | 0.769 | 0.130 | trans |
| rs34377578 | Diabetic polyneuropathy | 10 | 104336426 | A/C | 0.804 | -0.034 | 0.095 | 0.723 | 0.196 | trans |
| rs6580981 | Diabetic polyneuropathy | 12 | 54723028 | A/G | 0.582 | -0.054 | 0.077 | 0.484 | 0.419 | trans |
| rs78909033 | Diabetic polyneuropathy | 2 | 241510903 | A/G | 0.131 | -0.058 | 0.112 | 0.603 | 0.131 | trans |
| rs6081565 | Diabetic polyneuropathy | 20 | 19287904 | A/G | 0.281 | -0.059 | 0.084 | 0.479 | 0.281 | trans |
| rs11242109 | Diabetic polyneuropathy | 5 | 131677047 | T/G | 0.345 | -0.065 | 0.079 | 0.414 | 0.345 | trans |
| rs6961069 | Diabetic polyneuropathy | 7 | 80218961 | T/C | 0.417 | -0.076 | 0.077 | 0.324 | 0.417 | trans |
| rs61469632 | Diabetic polyneuropathy | 9 | 135861990 | C/T | 0.058 | -0.126 | 0.164 | 0.442 | 0.058 | trans |
| rs7618405 | Diabetic polyneuropathy | 3 | 18250509 | A/C | 0.211 | -0.178 | 0.093 | 0.055 | 0.211 | trans |
| rs13412535 | Diabetic polyneuropathy | 2 | 224874874 | A/G | 0.205 | -0.204 | 0.095 | 0.032 | 0.205 | trans |
| CLC |  |  |  |  |  |  |  |  |  |  |
| rs34210653 | Diabetic polyneuropathy | 17 | 4535314 | A/G | 0.007 | 0.290 | 0.450 | 0.520 | 0.007 | trans |
| rs7819099 | Diabetic polyneuropathy | 8 | 144992862 | G/A | 0.400 | 0.047 | 0.077 | 0.544 | 0.400 | trans |
| rs34436714 | Diabetic polyneuropathy | 19 | 54327313 | A/C | 0.199 | 0.043 | 0.095 | 0.649 | 0.199 | trans |
| rs4328821 | Diabetic polyneuropathy | 3 | 128316435 | G/A | 0.092 | 0.043 | 0.129 | 0.740 | 0.092 | trans |
| rs7809058 | Diabetic polyneuropathy | 7 | 75462520 | T/C | 0.927 | -0.336 | 0.146 | 0.021 | 0.073 | trans |
| BPNT2 |  |  |  |  |  |  |  |  |  |  |
| rs112433249 | Diabetic polyneuropathy | 8 | 57876576 | C/T | 0.006 | 0.082 | 0.471 | 0.862 | 0.006 | cis |
| IMPAD1 |  |  |  |  |  |  |  |  |  |  |
| rs112433249 | Diabetic polyneuropathy | 8 | 57876576 | C/T | 0.006 | 0.082 | 0.471 | 0.862 | 0.006 | cis |
| EPS8L2 |  |  |  |  |  |  |  |  |  |  |
| rs3747207 | Diabetic polyneuropathy | 22 | 44324855 | A/G | 0.226 | -0.010 | 0.090 | 0.915 | 0.226 | trans |
| rs11246276 | Diabetic polyneuropathy | 11 | 706284 | T/G | 0.125 | -0.122 | 0.114 | 0.286 | 0.125 | cis |
| IL22RA2 |  |  |  |  |  |  |  |  |  |  |
| rs9512995 | Diabetic polyneuropathy | 13 | 28614361 | A/G | 0.935 | 0.158 | 0.155 | 0.306 | 0.065 | trans |
| rs7287486 | Diabetic polyneuropathy | 22 | 42322854 | T/G | 0.023 | 0.058 | 0.244 | 0.814 | 0.023 | trans |
| rs2234711 | Diabetic polyneuropathy | 6 | 137540520 | A/G | 0.640 | -0.071 | 0.079 | 0.369 | 0.360 | cis |
| PPP3R1_PPP3CA |  |  |  |  |  |  |  |  |  |  |
| rs1354034 | Diabetic polyneuropathy | 3 | 56849749 | C/T | 0.707 | 0.052 | 0.083 | 0.532 | 0.293 | trans |
| rs1125271 | Diabetic polyneuropathy | 4 | 102745985 | T/C | 0.309 | -0.032 | 0.082 | 0.699 | 0.309 | trans |
| MMP9 |  |  |  |  |  |  |  |  |  |  |
| rs11253518 | Diabetic polyneuropathy | 10 | 971600 | C/A | 0.170 | -0.006 | 0.100 | 0.957 | 0.170 | trans |
| rs4660129 | Diabetic polyneuropathy | 1 | 236094087 | A/G | 0.086 | -0.057 | 0.134 | 0.670 | 0.086 | trans |
| rs2511241 | Diabetic polyneuropathy | 11 | 72945341 | T/C | 0.922 | -0.311 | 0.141 | 0.028 | 0.078 | trans |
| NAGLU |  |  |  |  |  |  |  |  |  |  |
| rs529541 | Diabetic polyneuropathy | 1 | 196719716 | G/A | 0.147 | 0.103 | 0.106 | 0.333 | 0.147 | trans |
| rs112629880 | Diabetic polyneuropathy | 17 | 40615761 | T/C | 0.091 | -0.055 | 0.133 | 0.678 | 0.091 | cis |
| P4HB |  |  |  |  |  |  |  |  |  |  |
| rs1354034 | Diabetic polyneuropathy | 3 | 56849749 | C/T | 0.707 | 0.052 | 0.083 | 0.532 | 0.293 | trans |
| rs112635299 | Diabetic polyneuropathy | 14 | 94838142 | T/G | 0.020 | -0.014 | 0.271 | 0.960 | 0.020 | trans |
| rs113708033 | Diabetic polyneuropathy | 17 | 79835930 | T/G | 0.152 | -0.133 | 0.105 | 0.208 | 0.152 | cis |
| HSPA5 |  |  |  |  |  |  |  |  |  |  |
| rs112635299 | Diabetic polyneuropathy | 14 | 94838142 | T/G | 0.020 | -0.014 | 0.271 | 0.960 | 0.020 | trans |
| rs9302635 | Diabetic polyneuropathy | 16 | 72144174 | C/T | 0.174 | -0.069 | 0.099 | 0.482 | 0.174 | trans |
| CD274 |  |  |  |  |  |  |  |  |  |  |
| rs822340 | Diabetic polyneuropathy | 9 | 5453260 | G/A | 0.727 | 0.068 | 0.085 | 0.421 | 0.273 | cis |
| rs1354034 | Diabetic polyneuropathy | 3 | 56849749 | C/T | 0.707 | 0.052 | 0.083 | 0.532 | 0.293 | trans |
| rs78444298 | Diabetic polyneuropathy | 1 | 184672098 | A/G | 0.017 | 0.023 | 0.295 | 0.937 | 0.017 | trans |
| rs112635299 | Diabetic polyneuropathy | 14 | 94838142 | T/G | 0.020 | -0.014 | 0.271 | 0.960 | 0.020 | trans |
| rs8178824 | Diabetic polyneuropathy | 17 | 64224775 | T/C | 0.009 | -0.140 | 0.389 | 0.718 | 0.009 | trans |
| MFAP5 |  |  |  |  |  |  |  |  |  |  |
| rs7412 | Diabetic polyneuropathy | 19 | 45412079 | T/C | 0.053 | 0.166 | 0.164 | 0.311 | 0.053 | trans |
| rs532436 | Diabetic polyneuropathy | 9 | 136149830 | A/G | 0.201 | 0.018 | 0.094 | 0.848 | 0.201 | trans |
| PNLIPRP1 |  |  |  |  |  |  |  |  |  |  |
| rs7120712 | Diabetic polyneuropathy | 11 | 100638002 | A/G | 0.281 | 0.072 | 0.084 | 0.391 | 0.281 | trans |
| rs72802342 | Diabetic polyneuropathy | 16 | 75234872 | A/C | 0.086 | -0.032 | 0.135 | 0.814 | 0.086 | trans |
| UBE2D3 |  |  |  |  |  |  |  |  |  |  |
| rs1126605 | Diabetic polyneuropathy | 12 | 7242204 | T/C | 0.079 | -0.176 | 0.138 | 0.204 | 0.079 | trans |
| DCTN2 |  |  |  |  |  |  |  |  |  |  |
| rs1803274 | Diabetic polyneuropathy | 3 | 165491280 | T/C | 0.174 | -0.016 | 0.099 | 0.869 | 0.174 | trans |
| rs1126605 | Diabetic polyneuropathy | 12 | 7242204 | T/C | 0.079 | -0.176 | 0.138 | 0.204 | 0.079 | trans |
| FBXL4 |  |  |  |  |  |  |  |  |  |  |
| rs1354034 | Diabetic polyneuropathy | 3 | 56849749 | C/T | 0.707 | 0.052 | 0.083 | 0.532 | 0.293 | trans |
| rs6073958 | Diabetic polyneuropathy | 20 | 44551855 | C/T | 0.176 | 0.002 | 0.099 | 0.985 | 0.176 | trans |
| rs78119247 | Diabetic polyneuropathy | 3 | 165499135 | G/GAT | 0.173 | -0.017 | 0.100 | 0.866 | 0.173 | trans |
| rs1126605 | Diabetic polyneuropathy | 12 | 7242204 | T/C | 0.079 | -0.176 | 0.138 | 0.204 | 0.079 | trans |
| NFKBID |  |  |  |  |  |  |  |  |  |  |
| rs1126605 | Diabetic polyneuropathy | 12 | 7242204 | T/C | 0.079 | -0.176 | 0.138 | 0.204 | 0.079 | trans |
| ZWINT |  |  |  |  |  |  |  |  |  |  |
| rs1126605 | Diabetic polyneuropathy | 12 | 7242204 | T/C | 0.079 | -0.176 | 0.138 | 0.204 | 0.079 | trans |
| ARFGAP1 |  |  |  |  |  |  |  |  |  |  |
| rs78119247 | Diabetic polyneuropathy | 3 | 165499135 | G/GAT | 0.173 | -0.017 | 0.100 | 0.866 | 0.173 | trans |
| rs1260326 | Diabetic polyneuropathy | 2 | 27730940 | T/C | 0.351 | -0.100 | 0.079 | 0.208 | 0.351 | trans |
| rs1126605 | Diabetic polyneuropathy | 12 | 7242204 | T/C | 0.079 | -0.176 | 0.138 | 0.204 | 0.079 | trans |
| PGM1 |  |  |  |  |  |  |  |  |  |  |
| rs1126728 | Diabetic polyneuropathy | 1 | 64097432 | T/C | 0.256 | 0.010 | 0.087 | 0.905 | 0.256 | cis |
| CST8 |  |  |  |  |  |  |  |  |  |  |
| rs112689088 | Diabetic polyneuropathy | 17 | 34307457 | C/T | 0.068 | -0.040 | 0.152 | 0.794 | 0.068 | cis |
| CCL16 |  |  |  |  |  |  |  |  |  |  |
| rs112689088 | Diabetic polyneuropathy | 17 | 34307457 | C/T | 0.068 | -0.040 | 0.152 | 0.794 | 0.068 | cis |
| MUC13 |  |  |  |  |  |  |  |  |  |  |
| rs1127233 | Diabetic polyneuropathy | 3 | 124627024 | G/T | 0.196 | 0.092 | 0.095 | 0.333 | 0.196 | cis |
| rs2519093 | Diabetic polyneuropathy | 9 | 136141870 | T/C | 0.201 | 0.028 | 0.094 | 0.762 | 0.201 | trans |
| rs4556017 | Diabetic polyneuropathy | 7 | 100632790 | C/T | 0.179 | 0.007 | 0.100 | 0.942 | 0.179 | trans |
| rs12019136 | Diabetic polyneuropathy | 19 | 5835677 | A/G | 0.028 | -0.139 | 0.228 | 0.544 | 0.028 | trans |
| ANGPTL7 |  |  |  |  |  |  |  |  |  |  |
| rs704 | Diabetic polyneuropathy | 17 | 26694861 | A/G | 0.420 | 0.049 | 0.076 | 0.519 | 0.420 | trans |
| rs7019896 | Diabetic polyneuropathy | 9 | 33113196 | T/G | 0.975 | -0.119 | 0.238 | 0.618 | 0.025 | trans |
| THBS4 |  |  |  |  |  |  |  |  |  |  |
| rs2438632 | Diabetic polyneuropathy | 5 | 79392193 | T/G | 0.322 | 0.035 | 0.081 | 0.662 | 0.322 | cis |
| rs56278466 | Diabetic polyneuropathy | 10 | 17875857 | G/T | 0.581 | -0.003 | 0.077 | 0.970 | 0.419 | trans |
| SCARA5 |  |  |  |  |  |  |  |  |  |  |
| rs2726951 | Diabetic polyneuropathy | 8 | 27805783 | T/C | 0.287 | -0.009 | 0.083 | 0.911 | 0.287 | cis |
| rs73724776 | Diabetic polyneuropathy | 6 | 18010768 | G/A | 0.043 | -0.035 | 0.184 | 0.848 | 0.043 | trans |
| rs597808 | Diabetic polyneuropathy | 12 | 111973358 | G/A | 0.586 | -0.054 | 0.077 | 0.480 | 0.415 | trans |
| rs1260326 | Diabetic polyneuropathy | 2 | 27730940 | T/C | 0.351 | -0.100 | 0.079 | 0.208 | 0.351 | trans |
| ITGAV |  |  |  |  |  |  |  |  |  |  |
| rs2868346 | Diabetic polyneuropathy | 20 | 44547970 | T/C | 0.749 | -0.005 | 0.087 | 0.955 | 0.251 | trans |
| rs2595391 | Diabetic polyneuropathy | 2 | 187532373 | G/T | 0.070 | -0.048 | 0.146 | 0.743 | 0.070 | cis |
| LYVE1 |  |  |  |  |  |  |  |  |  |  |
| rs6112930 | Diabetic polyneuropathy | 20 | 2106355 | A/G | 0.298 | 0.012 | 0.082 | 0.888 | 0.298 | trans |
| rs1955512 | Diabetic polyneuropathy | 14 | 33175822 | A/G | 0.586 | 0.000 | 0.077 | 0.997 | 0.414 | trans |
| rs35468145 | Diabetic polyneuropathy | 11 | 10647995 | A/G | 0.071 | -0.032 | 0.147 | 0.826 | 0.071 | cis |
| ADAMTS13 |  |  |  |  |  |  |  |  |  |  |
| rs9957318 | Diabetic polyneuropathy | 18 | 33039106 | G/A | 0.320 | 0.026 | 0.081 | 0.751 | 0.320 | trans |
| rs1433210 | Diabetic polyneuropathy | 4 | 124766956 | C/A | 0.248 | 0.005 | 0.088 | 0.953 | 0.248 | trans |
| rs3747207 | Diabetic polyneuropathy | 22 | 44324855 | A/G | 0.226 | -0.010 | 0.090 | 0.915 | 0.226 | trans |
| rs112814955 | Diabetic polyneuropathy | 12 | 6225931 | A/G | 0.076 | -0.056 | 0.144 | 0.698 | 0.076 | trans |
| APOC3 |  |  |  |  |  |  |  |  |  |  |
| rs58542926 | Diabetic polyneuropathy | 19 | 19379549 | T/C | 0.064 | 0.005 | 0.153 | 0.977 | 0.064 | trans |
| rs62295996 | Diabetic polyneuropathy | 3 | 165482064 | A/G | 0.174 | -0.015 | 0.099 | 0.878 | 0.174 | trans |
| rs112875651 | Diabetic polyneuropathy | 8 | 126506694 | A/G | 0.387 | -0.058 | 0.078 | 0.456 | 0.387 | trans |
| rs1260326 | Diabetic polyneuropathy | 2 | 27730940 | T/C | 0.351 | -0.100 | 0.079 | 0.208 | 0.351 | trans |
| APOF |  |  |  |  |  |  |  |  |  |  |
| rs2020854 | Diabetic polyneuropathy | 12 | 56743367 | C/T | 0.055 | 0.381 | 0.165 | 0.021 | 0.055 | cis |
| rs77924615 | Diabetic polyneuropathy | 16 | 20392332 | A/G | 0.222 | 0.042 | 0.091 | 0.643 | 0.222 | trans |
| rs2668202 | Diabetic polyneuropathy | 3 | 165490468 | A/G | 0.246 | -0.049 | 0.087 | 0.576 | 0.246 | trans |
| rs112875651 | Diabetic polyneuropathy | 8 | 126506694 | A/G | 0.387 | -0.058 | 0.078 | 0.456 | 0.387 | trans |
| rs150844304 | Diabetic polyneuropathy | 15 | 43726625 | C/A | 0.005 | -0.223 | 0.528 | 0.673 | 0.005 | trans |
| PTS |  |  |  |  |  |  |  |  |  |  |
| rs738408 | Diabetic polyneuropathy | 22 | 44324730 | T/C | 0.227 | -0.004 | 0.090 | 0.964 | 0.227 | trans |
| rs112875651 | Diabetic polyneuropathy | 8 | 126506694 | A/G | 0.387 | -0.058 | 0.078 | 0.456 | 0.387 | trans |
| FGFR2 |  |  |  |  |  |  |  |  |  |  |
| rs7826120 | Diabetic polyneuropathy | 8 | 59371725 | C/T | 0.615 | 0.064 | 0.078 | 0.407 | 0.386 | trans |
| rs6968170 | Diabetic polyneuropathy | 7 | 73020676 | A/G | 0.231 | 0.042 | 0.089 | 0.636 | 0.231 | trans |
| rs112875651 | Diabetic polyneuropathy | 8 | 126506694 | A/G | 0.387 | -0.058 | 0.078 | 0.456 | 0.387 | trans |
| rs36086195 | Diabetic polyneuropathy | 1 | 16510894 | T/C | 0.682 | -0.070 | 0.081 | 0.387 | 0.319 | trans |
| rs2981430 | Diabetic polyneuropathy | 10 | 123311698 | A/G | 0.585 | -0.130 | 0.077 | 0.091 | 0.415 | cis |
| VMO1 |  |  |  |  |  |  |  |  |  |  |
| rs117779442 | Diabetic polyneuropathy | 8 | 19839709 | T/C | 0.054 | 0.156 | 0.168 | 0.352 | 0.054 | trans |
| rs3826688 | Diabetic polyneuropathy | 19 | 45418961 | C/T | 0.712 | 0.083 | 0.084 | 0.323 | 0.289 | trans |
| rs261290 | Diabetic polyneuropathy | 15 | 58678720 | C/T | 0.618 | 0.050 | 0.078 | 0.524 | 0.382 | trans |
| rs112875651 | Diabetic polyneuropathy | 8 | 126506694 | A/G | 0.387 | -0.058 | 0.078 | 0.456 | 0.387 | trans |
| HEXIM2 |  |  |  |  |  |  |  |  |  |  |
| rs3811444 | Diabetic polyneuropathy | 1 | 248039451 | T/C | 0.346 | -0.037 | 0.080 | 0.645 | 0.346 | trans |
| rs112981250 | Diabetic polyneuropathy | 17 | 43223316 | C/CAT | 0.665 | -0.111 | 0.080 | 0.168 | 0.335 | cis |
| DNAJB8 |  |  |  |  |  |  |  |  |  |  |
| rs112988467 | Diabetic polyneuropathy | 14 | 107072766 | T/C | 0.240 | 0.011 | 0.090 | 0.904 | 0.240 | trans |
| rs12668458 | Diabetic polyneuropathy | 7 | 157145474 | T/C | 0.580 | -0.065 | 0.077 | 0.402 | 0.420 | trans |
| B2M |  |  |  |  |  |  |  |  |  |  |
| rs113010081 | Diabetic polyneuropathy | 3 | 46457412 | T/C | 0.871 | 0.044 | 0.113 | 0.697 | 0.129 | trans |
| rs653178 | Diabetic polyneuropathy | 12 | 112007756 | T/C | 0.584 | -0.054 | 0.077 | 0.477 | 0.416 | trans |
| CCL4 |  |  |  |  |  |  |  |  |  |  |
| rs113010081 | Diabetic polyneuropathy | 3 | 46457412 | T/C | 0.871 | 0.044 | 0.113 | 0.697 | 0.129 | trans |
| rs5743604 | Diabetic polyneuropathy | 4 | 38801285 | A/G | 0.874 | 0.012 | 0.113 | 0.914 | 0.126 | trans |
| rs8064426 | Diabetic polyneuropathy | 17 | 34819750 | A/G | 0.218 | -0.161 | 0.093 | 0.081 | 0.218 | cis |
| FABP6 |  |  |  |  |  |  |  |  |  |  |
| rs35866622 | Diabetic polyneuropathy | 19 | 49218060 | T/C | 0.339 | 0.017 | 0.080 | 0.829 | 0.339 | trans |
| PAEP |  |  |  |  |  |  |  |  |  |  |
| rs9409964 | Diabetic polyneuropathy | 9 | 138471928 | A/G | 0.129 | -0.288 | 0.112 | 0.010 | 0.129 | cis |
| MAPK9 |  |  |  |  |  |  |  |  |  |  |
| rs113096165 | Diabetic polyneuropathy | 5 | 179740366 | T/C | 0.190 | -0.037 | 0.097 | 0.705 | 0.190 | cis |
| PSG7 |  |  |  |  |  |  |  |  |  |  |
| rs113247044 | Diabetic polyneuropathy | 19 | 43439694 | A/G | 0.869 | -0.117 | 0.111 | 0.290 | 0.131 | cis |
| B3GALT6 |  |  |  |  |  |  |  |  |  |  |
| rs113272753 | Diabetic polyneuropathy | 1 | 1164749 | G/GC | 0.845 | 0.131 | 0.105 | 0.209 | 0.155 | cis |
| TNFRSF11A |  |  |  |  |  |  |  |  |  |  |
| rs7080386 | Diabetic polyneuropathy | 10 | 65048306 | A/C | 0.384 | -0.001 | 0.078 | 0.993 | 0.384 | trans |
| rs141129381 | Diabetic polyneuropathy | 9 | 100691900 | TG/T | 0.398 | -0.025 | 0.077 | 0.751 | 0.398 | trans |
| rs113339733 | Diabetic polyneuropathy | 18 | 60018329 | A/G | 0.121 | -0.064 | 0.116 | 0.579 | 0.121 | cis |
| CCL8 |  |  |  |  |  |  |  |  |  |  |
| rs1133763 | Diabetic polyneuropathy | 17 | 32647831 | A/C | 0.848 | -0.069 | 0.105 | 0.509 | 0.152 | cis |
| PLAT |  |  |  |  |  |  |  |  |  |  |
[truncated: 659,665 more chars]
