# Supplementary material for: Identification of potential drug targets for diabetic polyneuropathy through Mendelian randomization analysis
Source: Cell Biosci. 2024 Dec 5;14:147. doi: 10.1186/s13578-024-01323-4 (PMC11619124; doi:10.1186/s13578-024-01323-4)
Supplement: Supplementary file 4 — Supplementary Material 4: Figure S4. PLXNA4: (A) Forest plot, (B) Leave-one-out sensitivity analysis plot, (C) Scatter plot, and (D) Funnel plot. [file 13578_2024_1323_MOESM4_ESM.docx]

**Association Between Circulating Plasma Proteome and Diabetic Polyneuropathy Identified Using cis-pQTLs through Mendelian Randomization**

| **Exposure** | **Outcome** | **Method** | **Nsnp** | **MR** |  |  | **Heterogeneity** |  |  | **Horizontal pleiotropy** |  | **MR-PRESSO** |
| --- | --- | --- | --- | --- | --- | --- | --- | --- | --- | --- | --- | --- |
|  |  |  |  | **OR(95%CI)** | **P value** | **I^2^(%)** | **Cochran's Q** | **P-value** | **Egger intercept** | **SE** | **P-value** | **P-value** |
| VNN2 | Diabetic polyneuropathy | Wald ratio | 1 | 0.761(0.613-0.944) | 0.013 | - | - | - | - | - | - | - |
| VAV1 | Diabetic polyneuropathy | Wald ratio | 1 | 8.324(1.050-65.994) | 0.045 | - | - | - | - | - | - | - |
| UPP1 | Diabetic polyneuropathy | Wald ratio | 1 | 2.951(1.109-7.855) | 0.030 | - | - | - | - | - | - | - |
| TNFSF14 | Diabetic polyneuropathy | Wald ratio | 1 | 0.577(0.349-0.954) | 0.032 | - | - | - | - | - | - | - |
| TNFAIP3 | Diabetic polyneuropathy | Wald ratio | 1 | 0.283(0.106-0.754) | 0.012 | - | - | - | - | - | - | - |
| TIGIT | Diabetic polyneuropathy | Wald ratio | 1 | 0.046(0.005-0.435) | 0.007 | - | - | - | - | - | - | - |
| TGFA | Diabetic polyneuropathy | Wald ratio | 1 | 5.423(1.328-22.135) | 0.018 | - | - | - | - | - | - | - |
| SRI | Diabetic polyneuropathy | Wald ratio | 1 | 0.252(0.088-0.721) | 0.010 | - | - | - | - | - | - | - |
| SRA1 | Diabetic polyneuropathy | Wald ratio | 1 | 4.299(1.833-10.084) | 0.001 | - | - | - | - | - | - | - |
| SPINT2 | Diabetic polyneuropathy | Wald ratio | 1 | 0.846(0.725-0.988) | 0.034 | - | - | - | - | - | - | - |
| SLITRK6 | Diabetic polyneuropathy | Wald ratio | 1 | 0.563(0.367-0.865) | 0.009 | - | - | - | - | - | - | - |
| SERPINE2 | Diabetic polyneuropathy | Wald ratio | 1 | 1.258(1.020-1.551) | 0.032 | - | - | - | - | - | - | - |
| SERPINE1 | Diabetic polyneuropathy | Wald ratio | 1 | 0.152(0.030-0.757) | 0.021 | - | - | - | - | - | - | - |
| ROR1 | Diabetic polyneuropathy | Wald ratio | 1 | 1.651(1.088-2.507) | 0.019 | - | - | - | - | - | - | - |
| RET | Diabetic polyneuropathy | Wald ratio | 1 | 1.622(1.062-2.478) | 0.025 | - | - | - | - | - | - | - |
| RAB31 | Diabetic polyneuropathy | Wald ratio | 1 | 1.686(1.073-2.649) | 0.024 | - | - | - | - | - | - | - |
| PVRL4 | Diabetic polyneuropathy | Wald ratio | 1 | 0.043(0.003-0.667) | 0.024 | - | - | - | - | - | - | - |
| PVR | Diabetic polyneuropathy | Wald ratio | 1 | 0.728(0.580-0.914) | 0.006 | - | - | - | - | - | - | - |
| PTPRM | Diabetic polyneuropathy | Wald ratio | 1 | 10.446(1.425-76.562) | 0.021 | - | - | - | - | - | - | - |
| PODXL | Diabetic polyneuropathy | Wald ratio | 1 | 0.526(0.281-0.983) | 0.044 | - | - | - | - | - | - | - |
| PLXNA1 | Diabetic polyneuropathy | Wald ratio | 1 | 3.578(1.880-6.810) | <0.001 | - | - | - | - | - | - | - |
| PDE3A | Diabetic polyneuropathy | Wald ratio | 1 | 4.015(1.373-11.737) | 0.011 | - | - | - | - | - | - | - |
| PARP1 | Diabetic polyneuropathy | Wald ratio | 1 | 0.310(0.110-0.876) | 0.027 | - | - | - | - | - | - | - |
| PAEP | Diabetic polyneuropathy | Wald ratio | 1 | 0.719(0.559-0.924) | 0.010 | - | - | - | - | - | - | - |
| NUDCD3 | Diabetic polyneuropathy | Wald ratio | 1 | 2.182(1.019-4.676) | 0.045 | - | - | - | - | - | - | - |
| NHEJ1 | Diabetic polyneuropathy | Wald ratio | 1 | 2.843(1.185-6.824) | 0.019 | - | - | - | - | - | - | - |
| NECTIN4 | Diabetic polyneuropathy | Wald ratio | 1 | 0.555(0.342-0.902) | 0.018 | - | - | - | - | - | - | - |
| MGMT | Diabetic polyneuropathy | Wald ratio | 1 | 1.513(1.048-2.184) | 0.027 | - | - | - | - | - | - | - |
| LTBR | Diabetic polyneuropathy | Wald ratio | 1 | 1.709(1.011-2.891) | 0.046 | - | - | - | - | - | - | - |
| LIFR | Diabetic polyneuropathy | Wald ratio | 1 | 2.186(1.038-4.604) | 0.040 | - | - | - | - | - | - | - |
| KL | Diabetic polyneuropathy | Wald ratio | 1 | 0.390(0.159-0.958) | 0.040 | - | - | - | - | - | - | - |
| IPCEF1 | Diabetic polyneuropathy | Wald ratio | 1 | 9.723(1.349-70.084) | 0.024 | - | - | - | - | - | - | - |
| INHBC | Diabetic polyneuropathy | Wald ratio | 1 | 0.104(0.021-0.507) | 0.005 | - | - | - | - | - | - | - |
| INHBA_INHBC | Diabetic polyneuropathy | Wald ratio | 1 | 0.104(0.021-0.507) | 0.005 | - | - | - | - | - | - | - |
| IL5RA | Diabetic polyneuropathy | Wald ratio | 1 | 0.542(0.311-0.945) | 0.031 | - | - | - | - | - | - | - |
| IDUA | Diabetic polyneuropathy | Wald ratio | 1 | 1.343(1.023-1.764) | 0.034 | - | - | - | - | - | - | - |
| IDI2 | Diabetic polyneuropathy | Wald ratio | 1 | 1.755(1.003-3.072) | 0.049 | - | - | - | - | - | - | - |
| HADH | Diabetic polyneuropathy | Wald ratio | 1 | 7.487(1.554-36.064) | 0.012 | - | - | - | - | - | - | - |
| GGH | Diabetic polyneuropathy | Wald ratio | 1 | 1.353(1.026-1.783) | 0.032 | - | - | - | - | - | - | - |
| FGFBP1 | Diabetic polyneuropathy | Wald ratio | 1 | 0.190(0.047-0.773) | 0.020 | - | - | - | - | - | - | - |
| ENPP7 | Diabetic polyneuropathy | Wald ratio | 1 | 1.224(1.048-1.428) | 0.011 | - | - | - | - | - | - | - |
| ENO3 | Diabetic polyneuropathy | Wald ratio | 1 | 1.832(1.146-2.929) | 0.011 | - | - | - | - | - | - | - |
| EFNA3 | Diabetic polyneuropathy | Wald ratio | 1 | 514.571(1.815-145846.665) | 0.030 | - | - | - | - | - | - | - |
| EDAR | Diabetic polyneuropathy | Wald ratio | 1 | 1.852(1.129-3.036) | 0.015 | - | - | - | - | - | - | - |
| DKK3 | Diabetic polyneuropathy | Wald ratio | 1 | 1.611(1.058-2.452) | 0.026 | - | - | - | - | - | - | - |
| DDC | Diabetic polyneuropathy | Wald ratio | 1 | 1.702(1.030-2.811) | 0.038 | - | - | - | - | - | - | - |
| CRNN | Diabetic polyneuropathy | Wald ratio | 1 | 0.772(0.597-0.998) | 0.049 | - | - | - | - | - | - | - |
| CKM_CKB | Diabetic polyneuropathy | Wald ratio | 1 | 0.192(0.080-0.461) | <0.001 | - | - | - | - | - | - | - |
| CKM | Diabetic polyneuropathy | Wald ratio | 1 | 0.192(0.080-0.461) | <0.001 | - | - | - | - | - | - | - |
| CD72 | Diabetic polyneuropathy | Wald ratio | 1 | 4.767(1.375-16.530) | 0.014 | - | - | - | - | - | - | - |
| CD6 | Diabetic polyneuropathy | Wald ratio | 1 | 1.294(1.046-1.601) | 0.017 | - | - | - | - | - | - | - |
| CD58 | Diabetic polyneuropathy | Wald ratio | 1 | 0.450(0.212-0.952) | 0.037 | - | - | - | - | - | - | - |
| CD163 | Diabetic polyneuropathy | Wald ratio | 1 | 0.270(0.073-0.991) | 0.048 | - | - | - | - | - | - | - |
| CD14 | Diabetic polyneuropathy | Inverse variance weighted | 2 | 2.338(1.475-3.707) | <0.001 | 0 | 0.205 | 0.650 | - | - | - | - |
| CCL5 | Diabetic polyneuropathy | Wald ratio | 1 | 0.409(0.168-0.993) | 0.048 | - | - | - | - | - | - | - |
| CCL24 | Diabetic polyneuropathy | Wald ratio | 1 | 1.282(1.040-1.580) | 0.020 | - | - | - | - | - | - | - |
| CASP3 | Diabetic polyneuropathy | Wald ratio | 1 | 2.003(1.158-3.465) | 0.013 | - | - | - | - | - | - | - |
| CA12 | Diabetic polyneuropathy | Wald ratio | 1 | 0.541(0.314-0.935) | 0.028 | - | - | - | - | - | - | - |
| BDNF | Diabetic polyneuropathy | Wald ratio | 1 | 4.956(1.011-24.305) | 0.048 | - | - | - | - | - | - | - |
| ART3 | Diabetic polyneuropathy | Wald ratio | 1 | 0.541(0.322-0.909) | 0.020 | - | - | - | - | - | - | - |
| APOF | Diabetic polyneuropathy | Wald ratio | 1 | 0.293(0.103-0.831) | 0.021 | - | - | - | - | - | - | - |
| ACADM | Diabetic polyneuropathy | Wald ratio | 1 | 0.706(0.507-0.983) | 0.039 | - | - | - | - | - | - | - |

Note: Nsnp, Number of Single Nucleotide Polymorphisms; OR, Odds Ratio; SE, Standard Error of β
