# Supplementary material for: Identification of potential drug targets for diabetic polyneuropathy through Mendelian randomization analysis
Source: Cell Biosci. 2024 Dec 5;14:147. doi: 10.1186/s13578-024-01323-4 (PMC11619124; doi:10.1186/s13578-024-01323-4)
Supplement: Supplementary file 5 — Supplementary Material 5. [file 13578_2024_1323_MOESM5_ESM.docx]

**Association Between Circulating Plasma Proteome and Diabetic Polyneuropathy Identified Using cis-pQTLs + trans-pQTLs through Mendelian Randomization**

| **Exposure** | **Outcome** | **Method** | **Nsnp** | **MR** |  |  | **Heterogeneity** |  |  | **Horizontal pleiotropy** |  | **MR-PRESSO** |
| --- | --- | --- | --- | --- | --- | --- | --- | --- | --- | --- | --- | --- |
|  |  |  |  | **OR(95%CI)** | **P value** | **I^2^(%)** | **Cochran's Q** | **P-value** | **Egger intercept** | **SE** | **P-value** | **P-value** |
| ZHX3 | Diabetic polyneuropathy | Wald ratio | 1 | 0.504(0.279-0.908) | 0.023 | - | - | - | - | - | - | - |
| YWHAQ | Diabetic polyneuropathy | Inverse variance weighted | 2 | 0.537(0.325-0.887) | 0.015 | 0 | <0.001 | 0.991 | - | - | - | - |
| VNN2 | Diabetic polyneuropathy | Inverse variance weighted | 2 | 0.746(0.584-0.952) | 0.019 | 24 | 1.314 | 0.252 | - | - | - | - |
| UPP1 | Diabetic polyneuropathy | Wald ratio | 1 | 2.951(1.109-7.855) | 0.030 | - | - | - | - | - | - | - |
| ULBP2 | Diabetic polyneuropathy | Inverse variance weighted | 3 | 2.307(1.156-4.602) | 0.018 | 0 | 0.115 | 0.944 | - | - | - | 0.923 |
| ULBP2 | Diabetic polyneuropathy | MR Egger | 3 | 2.823(0.563-14.150) | 0.427 | 0 | 0.041 | 0.840 | -0.033 | 0.123 | 0.831 | - |
| ULBP2 | Diabetic polyneuropathy | Weighted median | 3 | 2.385(1.124-5.060) | 0.024 | - | - | - | - | - | - | - |
| ULBP2 | Diabetic polyneuropathy | Simple mode | 3 | 2.385(0.824-6.902) | 0.250 | - | - | - | - | - | - | - |
| ULBP2 | Diabetic polyneuropathy | Weighted mode | 3 | 2.385(1.079-5.269) | 0.165 | - | - | - | - | - | - | - |
| UBE2G1 | Diabetic polyneuropathy | Inverse variance weighted | 2 | 5.279(1.750-15.923) | 0.003 | 0 | 0.786 | 0.375 | - | - | - | - |
| TRAPPC3 | Diabetic polyneuropathy | Wald ratio | 1 | 0.504(0.279-0.908) | 0.023 | - | - | - | - | - | - | - |
| TP53I11 | Diabetic polyneuropathy | Inverse variance weighted | 2 | 0.515(0.315-0.845) | 0.009 | 0 | 0.280 | 0.597 | - | - | - | - |
| TNFSF14 | Diabetic polyneuropathy | Wald ratio | 1 | 0.115(0.016-0.832) | 0.032 | - | - | - | - | - | - | - |
| TNFRSF19 | Diabetic polyneuropathy | Inverse variance weighted | 3 | 0.531(0.293-0.964) | 0.037 | 0 | 1.100 | 0.577 | - | - | - | 0.871 |
| TNFRSF19 | Diabetic polyneuropathy | MR Egger | 3 | 0.691(0.224-2.132) | 0.636 | 0 | 0.810 | 0.368 | -0.052 | 0.096 | 0.686 | - |
| TNFRSF19 | Diabetic polyneuropathy | Weighted median | 3 | 0.557(0.298-1.042) | 0.067 | - | - | - | - | - | - | - |
| TNFRSF19 | Diabetic polyneuropathy | Simple mode | 3 | 0.652(0.239-1.781) | 0.492 | - | - | - | - | - | - | - |
| TNFRSF19 | Diabetic polyneuropathy | Weighted mode | 3 | 0.560(0.297-1.056) | 0.215 | - | - | - | - | - | - | - |
| TNFAIP3 | Diabetic polyneuropathy | Wald ratio | 1 | 0.283(0.106-0.754) | 0.012 | - | - | - | - | - | - | - |
| TMCC3 | Diabetic polyneuropathy | Inverse variance weighted | 2 | 0.515(0.315-0.845) | 0.009 | 0 | 0.280 | 0.597 | - | - | - | - |
| TGFA | Diabetic polyneuropathy | Inverse variance weighted | 2 | 4.911(1.510-15.964) | 0.008 | 0 | 0.064 | 0.800 | - | - | - | - |
| TG | Diabetic polyneuropathy | Wald ratio | 1 | 24.862(1.883-328.324) | 0.015 | - | - | - | - | - | - | - |
| TBXAS1 | Diabetic polyneuropathy | Wald ratio | 1 | 17.462(1.208-252.447) | 0.036 | - | - | - | - | - | - | - |
| TBCA | Diabetic polyneuropathy | Wald ratio | 1 | 0.536(0.320-0.898) | 0.018 | - | - | - | - | - | - | - |
| SUMF1 | Diabetic polyneuropathy | Inverse variance weighted | 2 | 0.559(0.354-0.883) | 0.013 | 0 | 0.300 | 0.584 | - | - | - | - |
| SRI | Diabetic polyneuropathy | Wald ratio | 1 | 0.252(0.088-0.721) | 0.010 | - | - | - | - | - | - | - |
| SRA1 | Diabetic polyneuropathy | Inverse variance weighted | 2 | 3.947(1.773-8.787) | 0.001 | 0 | 0.324 | 0.569 | - | - | - | - |
| SPINT2 | Diabetic polyneuropathy | Wald ratio | 1 | 0.455(0.220-0.944) | 0.034 | - | - | - | - | - | - | - |
| SMOC2 | Diabetic polyneuropathy | Inverse variance weighted | 5 | 0.633(0.408-0.984) | 0.042 | 22 | 5.116 | 0.276 | - | - | - | 0.453 |
| SMOC2 | Diabetic polyneuropathy | MR Egger | 5 | 0.956(0.541-1.687) | 0.886 | 0 | 1.327 | 0.723 | -0.114 | 0.058 | 0.147 | - |
| SMOC2 | Diabetic polyneuropathy | Weighted median | 5 | 0.702(0.463-1.063) | 0.094 | - | - | - | - | - | - | - |
| SMOC2 | Diabetic polyneuropathy | Simple mode | 5 | 0.168(0.031-0.915) | 0.108 | - | - | - | - | - | - | - |
| SMOC2 | Diabetic polyneuropathy | Weighted mode | 5 | 0.716(0.467-1.097) | 0.200 | - | - | - | - | - | - | - |
| SHBG | Diabetic polyneuropathy | Inverse variance weighted | 5 | 0.455(0.219-0.942) | 0.034 | 0 | 2.305 | 0.680 | - | - | - | 0.794 |
| SHBG | Diabetic polyneuropathy | MR Egger | 5 | 0.575(0.091-3.636) | 0.598 | 0 | 2.231 | 0.526 | -0.029 | 0.105 | 0.803 | - |
| SHBG | Diabetic polyneuropathy | Weighted median | 5 | 0.425(0.193-0.937) | 0.034 | - | - | - | - | - | - | - |
| SHBG | Diabetic polyneuropathy | Simple mode | 5 | 0.380(0.115-1.250) | 0.187 | - | - | - | - | - | - | - |
| SHBG | Diabetic polyneuropathy | Weighted mode | 5 | 0.421(0.184-0.965) | 0.111 | - | - | - | - | - | - | - |
| SFXN5 | Diabetic polyneuropathy | Wald ratio | 1 | 1.832(1.146-2.929) | 0.011 | - | - | - | - | - | - | - |
| SDC1 | Diabetic polyneuropathy | Inverse variance weighted | 10 | 0.467(0.219-0.992) | 0.048 | 0 | 4.378 | 0.885 | - | - | - | 0.914 |
| SDC1 | Diabetic polyneuropathy | MR Egger | 10 | 0.116(0.004-3.454) | 0.248 | 0 | 3.696 | 0.883 | 0.113 | 0.136 | 0.433 | - |
| SDC1 | Diabetic polyneuropathy | Weighted median | 10 | 0.575(0.212-1.563) | 0.279 | - | - | - | - | - | - | - |
| SDC1 | Diabetic polyneuropathy | Simple mode | 10 | 0.666(0.148-3.007) | 0.610 | - | - | - | - | - | - | - |
| SDC1 | Diabetic polyneuropathy | Weighted mode | 10 | 0.638(0.164-2.479) | 0.532 | - | - | - | - | - | - | - |
| SARS | Diabetic polyneuropathy | Wald ratio | 1 | 0.536(0.320-0.898) | 0.018 | - | - | - | - | - | - | - |
| RSPO3 | Diabetic polyneuropathy | Inverse variance weighted | 2 | 1.426(1.005-2.023) | 0.047 | 0 | 0.226 | 0.635 | - | - | - | 0.153 |
| RNASET2 | Diabetic polyneuropathy | Inverse variance weighted | 5 | 0.693(0.496-0.969) | 0.032 | 26 | 5.370 | 0.251 | - | - | - | 0.488 |
| RNASET2 | Diabetic polyneuropathy | MR Egger | 5 | 0.976(0.560-1.701) | 0.938 | 6 | 3.187 | 0.364 | -0.131 | 0.091 | 0.247 | - |
| RNASET2 | Diabetic polyneuropathy | Weighted median | 5 | 0.704(0.505-0.981) | 0.038 | - | - | - | - | - | - | - |
| RNASET2 | Diabetic polyneuropathy | Simple mode | 5 | 0.442(0.196-0.996) | 0.120 | - | - | - | - | - | - | - |
| RNASET2 | Diabetic polyneuropathy | Weighted mode | 5 | 0.772(0.547-1.091) | 0.216 | - | - | - | - | - | - | - |
| RGS7 | Diabetic polyneuropathy | Wald ratio | 1 | 0.536(0.320-0.898) | 0.018 | - | - | - | - | - | - | - |
| RET | Diabetic polyneuropathy | Inverse variance weighted | 5 | 1.647(1.102-2.460) | 0.015 | 0 | 3.211 | 0.523 | - | - | - | 0.778 |
| RET | Diabetic polyneuropathy | MR Egger | 5 | 1.529(0.884-2.644) | 0.226 | 2 | 3.052 | 0.384 | 0.021 | 0.053 | 0.719 | - |
| RET | Diabetic polyneuropathy | Weighted median | 5 | 1.603(1.049-2.451) | 0.029 | - | - | - | - | - | - | - |
| RET | Diabetic polyneuropathy | Simple mode | 5 | 1.369(0.459-4.089) | 0.603 | - | - | - | - | - | - | - |
| RET | Diabetic polyneuropathy | Weighted mode | 5 | 1.606(1.036-2.490) | 0.101 | - | - | - | - | - | - | - |
| RARRES3 | Diabetic polyneuropathy | Wald ratio | 1 | 1.707(1.128-2.583) | 0.011 | - | - | - | - | - | - | - |
| RACGAP1 | Diabetic polyneuropathy | Wald ratio | 1 | 0.029(0.001-0.812) | 0.037 | - | - | - | - | - | - | - |
| RAB31 | Diabetic polyneuropathy | Wald ratio | 1 | 2.617(1.138-6.017) | 0.024 | - | - | - | - | - | - | - |
| RAB22A | Diabetic polyneuropathy | Inverse variance weighted | 3 | 2.324(1.187-4.549) | 0.014 | 0 | 0.238 | 0.888 | - | - | - | - |
| RAB22A | Diabetic polyneuropathy | MR Egger | 3 | 2.417(0.446-13.107) | 0.493 | 0 | 0.235 | 0.628 | -0.007 | 0.134 | 0.968 | - |
| RAB22A | Diabetic polyneuropathy | Weighted median | 3 | 2.345(1.142-4.813) | 0.020 | - | - | - | - | - | - | - |
| RAB22A | Diabetic polyneuropathy | Simple mode | 3 | 2.011(0.770-5.255) | 0.290 | - | - | - | - | - | - | - |
| RAB22A | Diabetic polyneuropathy | Weighted mode | 3 | 2.581(1.205-5.529) | 0.135 | - | - | - | - | - | - | - |
| PVRL4 | Diabetic polyneuropathy | Wald ratio | 1 | 0.043(0.003-0.667) | 0.024 | - | - | - | - | - | - | - |
| PVR | Diabetic polyneuropathy | Wald ratio | 1 | 0.728(0.580-0.914) | 0.006 | - | - | - | - | - | - | - |
| PSAP | Diabetic polyneuropathy | Wald ratio | 1 | 0.208(0.047-0.929) | 0.040 | - | - | - | - | - | - | - |
| PRKCG | Diabetic polyneuropathy | Wald ratio | 1 | 0.504(0.279-0.908) | 0.023 | - | - | - | - | - | - | - |
| PPP2R3A | Diabetic polyneuropathy | Wald ratio | 1 | 0.062(0.005-0.761) | 0.030 | - | - | - | - | - | - | - |
| PPP1R12A | Diabetic polyneuropathy | Inverse variance weighted | 3 | 6.109(1.381-27.015) | 0.017 | 0 | 1.490 | 0.475 | - | - | - | 0.630 |
| PPP1R12A | Diabetic polyneuropathy | MR Egger | 3 | 0.178(0.000-7599554144.478) | 0.913 | 27 | 1.379 | 0.240 | 0.236 | 0.833 | 0.824 | - |
| PPP1R12A | Diabetic polyneuropathy | Weighted median | 3 | 6.991(1.165-41.952) | 0.033 | - | - | - | - | - | - | - |
| PPP1R12A | Diabetic polyneuropathy | Simple mode | 3 | 11.947(1.146-124.597) | 0.174 | - | - | - | - | - | - | - |
| PPP1R12A | Diabetic polyneuropathy | Weighted mode | 3 | 11.947(1.171-121.837) | 0.171 | - | - | - | - | - | - | - |
| PPA2 | Diabetic polyneuropathy | Inverse variance weighted | 2 | 4.581(1.387-15.128) | 0.013 | 0 | 0.593 | 0.441 | - | - | - | - |
| PLXNA4 | Diabetic polyneuropathy | Inverse variance weighted | 4 | 3.891(1.368-11.065) | 0.011 | 0 | 0.718 | 0.869 | - | - | - | 0.911 |
| PLXNA4 | Diabetic polyneuropathy | MR Egger | 4 | 2.573(0.037-181.050) | 0.706 | 0 | 0.679 | 0.712 | 0.036 | 0.185 | 0.862 | - |
| PLXNA4 | Diabetic polyneuropathy | Weighted median | 4 | 3.755(1.073-13.140) | 0.038 | - | - | - | - | - | - | - |
| PLXNA4 | Diabetic polyneuropathy | Simple mode | 4 | 3.970(0.811-19.438) | 0.187 | - | - | - | - | - | - | - |
| PLXNA4 | Diabetic polyneuropathy | Weighted mode | 4 | 3.554(0.822-15.363) | 0.188 | - | - | - | - | - | - | - |
| PKP2 | Diabetic polyneuropathy | Inverse variance weighted | 3 | 2.472(1.108-5.512) | 0.027 | 0 | 0.342 | 0.843 | - | - | - | - |
| PKP2 | Diabetic polyneuropathy | MR Egger | 3 | 2.120(0.496-9.054) | 0.495 | 0 | 0.280 | 0.597 | 0.026 | 0.103 | 0.845 | - |
| PKP2 | Diabetic polyneuropathy | Weighted median | 3 | 2.328(0.927-5.847) | 0.072 | - | - | - | - | - | - | - |
| PKP2 | Diabetic polyneuropathy | Simple mode | 3 | 2.127(0.628-7.209) | 0.349 | - | - | - | - | - | - | - |
| PKP2 | Diabetic polyneuropathy | Weighted mode | 3 | 2.149(0.768-6.014) | 0.282 | - | - | - | - | - | - | - |
| PDZK1IP1 | Diabetic polyneuropathy | Wald ratio | 1 | 0.536(0.320-0.898) | 0.018 | - | - | - | - | - | - | - |
| PDZD11 | Diabetic polyneuropathy | Wald ratio | 1 | 7.352(1.458-37.076) | 0.016 | - | - | - | - | - | - | - |
| PDE3A | Diabetic polyneuropathy | Wald ratio | 1 | 4.015(1.373-11.737) | 0.011 | - | - | - | - | - | - | - |
| PCSK9 | Diabetic polyneuropathy | Inverse variance weighted | 6 | 0.407(0.191-0.870) | 0.020 | 0 | 3.795 | 0.579 | - | - | - | 0.635 |
| PCSK9 | Diabetic polyneuropathy | MR Egger | 6 | 0.403(0.053-3.079) | 0.431 | 0 | 3.795 | 0.435 | 0.001 | 0.111 | 0.992 | - |
| PCSK9 | Diabetic polyneuropathy | Weighted median | 6 | 0.297(0.114-0.776) | 0.013 | - | - | - | - | - | - | - |
| PCSK9 | Diabetic polyneuropathy | Simple mode | 6 | 0.229(0.055-0.959) | 0.100 | - | - | - | - | - | - | - |
| PCSK9 | Diabetic polyneuropathy | Weighted mode | 6 | 0.242(0.058-1.014) | 0.110 | - | - | - | - | - | - | - |
| PAEP | Diabetic polyneuropathy | Wald ratio | 1 | 0.719(0.559-0.924) | 0.010 | - | - | - | - | - | - | - |
| OBP2B | Diabetic polyneuropathy | Inverse variance weighted | 2 | 0.764(0.605-0.964) | 0.023 | 0 | 0.935 | 0.333 | - | - | - | - |
| NUDCD3 | Diabetic polyneuropathy | Inverse variance weighted | 2 | 2.174(1.054-4.484) | 0.036 | 0 | 0.001 | 0.974 | - | - | - | - |
| NPC2 | Diabetic polyneuropathy | Wald ratio | 1 | 0.364(0.171-0.774) | 0.009 | - | - | - | - | - | - | - |
| NHEJ1 | Diabetic polyneuropathy | Inverse variance weighted | 2 | 2.737(1.207-6.210) | 0.016 | 0 | 0.058 | 0.810 | - | - | - | - |
| NEFL | Diabetic polyneuropathy | Wald ratio | 1 | 0.536(0.320-0.898) | 0.018 | - | - | - | - | - | - | - |
| NECTIN4 | Diabetic polyneuropathy | Inverse variance weighted | 5 | 0.530(0.341-0.822) | 0.005 | 0 | 1.040 | 0.904 | - | - | - | 0.865 |
| NECTIN4 | Diabetic polyneuropathy | MR Egger | 5 | 0.537(0.286-1.006) | 0.147 | 0 | 1.036 | 0.792 | -0.003 | 0.061 | 0.958 | - |
| NECTIN4 | Diabetic polyneuropathy | Weighted median | 5 | 0.554(0.338-0.908) | 0.019 | - | - | - | - | - | - | - |
| NECTIN4 | Diabetic polyneuropathy | Simple mode | 5 | 0.426(0.176-1.028) | 0.130 | - | - | - | - | - | - | - |
| NECTIN4 | Diabetic polyneuropathy | Weighted mode | 5 | 0.544(0.336-0.882) | 0.069 | - | - | - | - | - | - | - |
| MGMT | Diabetic polyneuropathy | Inverse variance weighted | 2 | 4.111(1.163-14.536) | 0.028 | 0 | 0.460 | 0.498 | - | - | - | - |
| LRRN1 | Diabetic polyneuropathy | Inverse variance weighted | 2 | 0.569(0.385-0.842) | 0.005 | 0 | 0.121 | 0.728 | - | - | - | - |
| LRRC25 | Diabetic polyneuropathy | Inverse variance weighted | 6 | 0.472(0.228-0.980) | 0.044 | 12 | 5.680 | 0.339 | - | - | - | 0.206 |
| LRRC25 | Diabetic polyneuropathy | MR Egger | 6 | 0.248(0.089-0.689) | 0.056 | 0 | 2.915 | 0.572 | 0.103 | 0.062 | 0.172 | - |
| LRRC25 | Diabetic polyneuropathy | Weighted median | 6 | 0.346(0.160-0.751) | 0.007 | - | - | - | - | - | - | - |
| LRRC25 | Diabetic polyneuropathy | Simple mode | 6 | 0.402(0.109-1.487) | 0.230 | - | - | - | - | - | - | - |
| LRRC25 | Diabetic polyneuropathy | Weighted mode | 6 | 0.342(0.151-0.778) | 0.051 | - | - | - | - | - | - | - |
| LEFTY2 | Diabetic polyneuropathy | Inverse variance weighted | 3 | 0.487(0.281-0.845) | 0.010 | 0 | 0.490 | 0.783 | - | - | - | - |
| LEFTY2 | Diabetic polyneuropathy | MR Egger | 3 | 0.496(0.253-0.974) | 0.291 | 0 | 0.481 | 0.488 | -0.008 | 0.090 | 0.940 | - |
| LEFTY2 | Diabetic polyneuropathy | Weighted median | 3 | 0.504(0.275-0.925) | 0.027 | - | - | - | - | - | - | - |
| LEFTY2 | Diabetic polyneuropathy | Simple mode | 3 | 0.562(0.210-1.510) | 0.372 | - | - | - | - | - | - | - |
| LEFTY2 | Diabetic polyneuropathy | Weighted mode | 3 | 0.509(0.275-0.941) | 0.164 | - | - | - | - | - | - | - |
| JPH4 | Diabetic polyneuropathy | Wald ratio | 1 | 0.029(0.001-0.812) | 0.037 | - | - | - | - | - | - | - |
| ITM2B | Diabetic polyneuropathy | Inverse variance weighted | 4 | 0.594(0.376-0.940) | 0.026 | 0 | 1.428 | 0.699 | - | - | - | 0.710 |
| ITM2B | Diabetic polyneuropathy | MR Egger | 4 | 0.418(0.194-0.900) | 0.156 | 0 | 0.168 | 0.920 | 0.094 | 0.083 | 0.378 | - |
| ITM2B | Diabetic polyneuropathy | Weighted median | 4 | 0.550(0.339-0.890) | 0.015 | - | - | - | - | - | - | - |
| ITM2B | Diabetic polyneuropathy | Simple mode | 4 | 0.602(0.259-1.404) | 0.325 | - | - | - | - | - | - | - |
| ITM2B | Diabetic polyneuropathy | Weighted mode | 4 | 0.548(0.335-0.894) | 0.095 | - | - | - | - | - | - | - |
| IPCEF1 | Diabetic polyneuropathy | Inverse variance weighted | 2 | 5.104(1.134-22.972) | 0.034 | 0 | 0.974 | 0.324 | - | - | - | - |
| INHBC | Diabetic polyneuropathy | Wald ratio | 1 | 0.528(0.338-0.826) | 0.005 | - | - | - | - | - | - | - |
| INHBB | Diabetic polyneuropathy | Inverse variance weighted | 4 | 0.562(0.368-0.860) | 0.008 | 0 | 2.549 | 0.466 | - | - | - | 0.467 |
| INHBB | Diabetic polyneuropathy | MR Egger | 4 | 0.477(0.250-0.908) | 0.153 | 3 | 2.072 | 0.355 | 0.048 | 0.071 | 0.567 | - |
| INHBB | Diabetic polyneuropathy | Weighted median | 4 | 0.543(0.343-0.860) | 0.009 | - | - | - | - | - | - | - |
| INHBB | Diabetic polyneuropathy | Simple mode | 4 | 0.588(0.174-1.990) | 0.456 | - | - | - | - | - | - | - |
| INHBB | Diabetic polyneuropathy | Weighted mode | 4 | 0.533(0.346-0.823) | 0.065 | - | - | - | - | - | - | - |
| INHBA_INHBC | Diabetic polyneuropathy | Wald ratio | 1 | 0.528(0.338-0.826) | 0.005 | - | - | - | - | - | - | - |
| IDUA | Diabetic polyneuropathy | Wald ratio | 1 | 1.343(1.023-1.764) | 0.034 | - | - | - | - | - | - | - |
| IDI2 | Diabetic polyneuropathy | Wald ratio | 1 | 1.755(1.003-3.072) | 0.049 | - | - | - | - | - | - | - |
| HNF4A | Diabetic polyneuropathy | Wald ratio | 1 | 0.504(0.279-0.908) | 0.023 | - | - | - | - | - | - | - |
| HAVCR2 | Diabetic polyneuropathy | Wald ratio | 1 | 1.945(1.099-3.442) | 0.022 | - | - | - | - | - | - | - |
| HADH | Diabetic polyneuropathy | Wald ratio | 1 | 7.487(1.554-36.064) | 0.012 | - | - | - | - | - | - | - |
| GPN1 | Diabetic polyneuropathy | Inverse variance weighted | 3 | 1.593(1.005-2.524) | 0.048 | 0 | 1.218 | 0.544 | - | - | - | - |
| GPN1 | Diabetic polyneuropathy | MR Egger | 3 | 1.194(0.413-3.450) | 0.799 | 0 | 0.869 | 0.351 | 0.065 | 0.111 | 0.660 | - |
| GPN1 | Diabetic polyneuropathy | Weighted median | 3 | 1.591(0.987-2.564) | 0.056 | - | - | - | - | - | - | - |
| GPN1 | Diabetic polyneuropathy | Simple mode | 3 | 2.249(1.045-4.842) | 0.174 | - | - | - | - | - | - | - |
| GPN1 | Diabetic polyneuropathy | Weighted mode | 3 | 1.352(0.826-2.215) | 0.353 | - | - | - | - | - | - | - |
| GPC1 | Diabetic polyneuropathy | Inverse variance weighted | 11 | 0.624(0.404-0.964) | 0.034 | 22 | 12.878 | 0.231 | - | - | - | 0.460 |
| GPC1 | Diabetic polyneuropathy | MR Egger | 11 | 0.703(0.373-1.323) | 0.303 | 28 | 12.498 | 0.187 | -0.026 | 0.050 | 0.613 | - |
| GPC1 | Diabetic polyneuropathy | Weighted median | 11 | 0.677(0.439-1.041) | 0.076 | - | - | - | - | - | - | - |
| GPC1 | Diabetic polyneuropathy | Simple mode | 11 | 1.233(0.356-4.271) | 0.748 | - | - | - | - | - | - | - |
| GPC1 | Diabetic polyneuropathy | Weighted mode | 11 | 0.658(0.405-1.069) | 0.122 | - | - | - | - | - | - | - |
| GPA33 | Diabetic polyneuropathy | Inverse variance weighted | 8 | 0.558(0.315-0.989) | 0.046 | 0 | 3.817 | 0.801 | - | - | - | 0.818 |
| GPA33 | Diabetic polyneuropathy | MR Egger | 8 | 0.502(0.216-1.168) | 0.161 | 0 | 3.705 | 0.717 | 0.018 | 0.055 | 0.749 | - |
| GPA33 | Diabetic polyneuropathy | Weighted median | 8 | 0.510(0.252-1.031) | 0.061 | - | - | - | - | - | - | - |
| GPA33 | Diabetic polyneuropathy | Simple mode | 8 | 0.526(0.168-1.646) | 0.306 | - | - | - | - | - | - | - |
| GPA33 | Diabetic polyneuropathy | Weighted mode | 8 | 0.513(0.236-1.116) | 0.136 | - | - | - | - | - | - | - |
| GOLM2 | Diabetic polyneuropathy | Inverse variance weighted | 4 | 0.771(0.600-0.989) | 0.041 | 0 | 1.541 | 0.673 | - | - | - | 0.575 |
| GOLM2 | Diabetic polyneuropathy | MR Egger | 4 | 1.005(0.586-1.724) | 0.988 | 0 | 0.361 | 0.835 | -0.133 | 0.123 | 0.391 | - |
| GOLM2 | Diabetic polyneuropathy | Weighted median | 4 | 0.774(0.595-1.008) | 0.058 | - | - | - | - | - | - | - |
| GOLM2 | Diabetic polyneuropathy | Simple mode | 4 | 0.513(0.246-1.068) | 0.172 | - | - | - | - | - | - | - |
| GOLM2 | Diabetic polyneuropathy | Weighted mode | 4 | 0.798(0.625-1.018) | 0.167 | - | - | - | - | - | - | - |
| GHR | Diabetic polyneuropathy | Inverse variance weighted | 2 | 0.515(0.315-0.845) | 0.009 | 0 | 0.280 | 0.597 | - | - | - | - |
| GGT2 | Diabetic polyneuropathy | Inverse variance weighted | 2 | 0.515(0.315-0.845) | 0.009 | 0 | 0.280 | 0.597 | - | - | - | - |
| GALNT13 | Diabetic polyneuropathy | Inverse variance weighted | 2 | 0.070(0.008-0.636) | 0.018 | 0 | 0.026 | 0.872 | - | - | - | - |
| FJX1 | Diabetic polyneuropathy | Inverse variance weighted | 2 | 0.308(0.111-0.852) | 0.023 | 0 | 0.489 | 0.484 | - | - | - | - |
| FGFBP1 | Diabetic polyneuropathy | Inverse variance weighted | 7 | 0.387(0.182-0.822) | 0.013 | 0 | 3.749 | 0.711 | - | - | - | 0.711 |
| FGFBP1 | Diabetic polyneuropathy | MR Egger | 7 | 0.070(0.008-0.647) | 0.066 | 0 | 1.182 | 0.947 | 0.160 | 0.100 | 0.170 | - |
| FGFBP1 | Diabetic polyneuropathy | Weighted median | 7 | 0.333(0.129-0.859) | 0.023 | - | - | - | - | - | - | - |
| FGFBP1 | Diabetic polyneuropathy | Simple mode | 7 | 0.321(0.082-1.259) | 0.154 | - | - | - | - | - | - | - |
| FGFBP1 | Diabetic polyneuropathy | Weighted mode | 7 | 0.284(0.088-0.914) | 0.079 | - | - | - | - | - | - | - |
| ENPP7 | Diabetic polyneuropathy | Wald ratio | 1 | 1.224(1.048-1.428) | 0.011 | - | - | - | - | - | - | - |
| ENG | Diabetic polyneuropathy | Inverse variance weighted | 6 | 0.443(0.214-0.920) | 0.029 | 0 | 4.577 | 0.470 | - | - | - | 0.356 |
| ENG | Diabetic polyneuropathy | MR Egger | 6 | 0.787(0.176-3.521) | 0.770 | 0 | 3.838 | 0.428 | -0.081 | 0.094 | 0.438 | - |
| ENG | Diabetic polyneuropathy | Weighted median | 6 | 0.587(0.240-1.435) | 0.243 | - | - | - | - | - | - | - |
| ENG | Diabetic polyneuropathy | Simple mode | 6 | 0.479(0.132-1.741) | 0.314 | - | - | - | - | - | - | - |
| ENG | Diabetic polyneuropathy | Weighted mode | 6 | 0.508(0.188-1.371) | 0.239 | - | - | - | - | - | - | - |
| EMC8 | Diabetic polyneuropathy | Wald ratio | 1 | 4.243(1.349-13.347) | 0.013 | - | - | - | - | - | - | - |
| EFNA3 | Diabetic polyneuropathy | Wald ratio | 1 | 514.571(1.815-145846.665) | 0.030 | - | - | - | - | - | - | - |
| EDIL3 | Diabetic polyneuropathy | Wald ratio | 1 | 6.915(1.971-24.263) | 0.003 | - | - | - | - | - | - | - |
| EDAR | Diabetic polyneuropathy | Inverse variance weighted | 14 | 1.526(1.038-2.245) | 0.032 | 0 | 11.707 | 0.552 | - | - | - | 0.635 |
| EDAR | Diabetic polyneuropathy | MR Egger | 14 | 1.707(1.026-2.839) | 0.062 | 0 | 11.272 | 0.506 | -0.027 | 0.041 | 0.522 | - |
| EDAR | Diabetic polyneuropathy | Weighted median | 14 | 1.826(1.137-2.933) | 0.013 | - | - | - | - | - | - | - |
| EDAR | Diabetic polyneuropathy | Simple mode | 14 | 1.771(0.560-5.594) | 0.348 | - | - | - | - | - | - | - |
| EDAR | Diabetic polyneuropathy | Weighted mode | 14 | 1.890(1.188-3.007) | 0.019 | - | - | - | - | - | - | - |
| DLK1 | Diabetic polyneuropathy | Wald ratio | 1 | 6.915(1.971-24.263) | 0.003 | - | - | - | - | - | - | - |
| DEFB104A | Diabetic polyneuropathy | Wald ratio | 1 | 0.062(0.005-0.761) | 0.030 | - | - | - | - | - | - | - |
| DDC | Diabetic polyneuropathy | Inverse variance weighted | 4 | 1.614(1.012-2.574) | 0.044 | 0 | 2.419 | 0.490 | - | - | - | 0.753 |
| DDC | Diabetic polyneuropathy | MR Egger | 4 | 1.678(0.947-2.974) | 0.218 | 14 | 2.329 | 0.312 | -0.016 | 0.057 | 0.807 | - |
| DDC | Diabetic polyneuropathy | Weighted median | 4 | 1.616(0.987-2.644) | 0.056 | - | - | - | - | - | - | - |
| DDC | Diabetic polyneuropathy | Simple mode | 4 | 0.860(0.265-2.794) | 0.818 | - | - | - | - | - | - | - |
| DDC | Diabetic polyneuropathy | Weighted mode | 4 | 1.673(1.042-2.688) | 0.123 | - | - | - | - | - | - | - |
| DCUN1D5 | Diabetic polyneuropathy | Inverse variance weighted | 2 | 0.515(0.315-0.845) | 0.009 | 0 | 0.280 | 0.597 | - | - | - | - |
| DCUN1D1 | Diabetic polyneuropathy | Inverse variance weighted | 2 | 0.568(0.324-0.995) | 0.048 | 40 | 1.679 | 0.195 | - | - | - | - |
| DCK | Diabetic polyneuropathy | Wald ratio | 1 | 0.536(0.320-0.898) | 0.018 | - | - | - | - | - | - | - |
| CTSH | Diabetic polyneuropathy | Wald ratio | 1 | 0.171(0.033-0.887) | 0.036 | - | - | - | - | - | - | - |
| CTF1 | Diabetic polyneuropathy | Wald ratio | 1 | 0.536(0.320-0.898) | 0.018 | - | - | - | - | - | - | - |
| CRYZL1 | Diabetic polyneuropathy | Inverse variance weighted | 2 | 0.263(0.072-0.960) | 0.043 | 0 | 0.150 | 0.698 | - | - | - | - |
| CRP | Diabetic polyneuropathy | Inverse variance weighted | 3 | 0.633(0.415-0.966) | 0.034 | 0 | 1.354 | 0.508 | - | - | - | 0.441 |
| CRP | Diabetic polyneuropathy | MR Egger | 3 | 0.390(0.155-0.983) | 0.296 | 0 | 0.023 | 0.881 | 0.125 | 0.108 | 0.455 | - |
| CRP | Diabetic polyneuropathy | Weighted median | 3 | 0.626(0.397-0.988) | 0.044 | - | - | - | - | - | - | - |
| CRP | Diabetic polyneuropathy | Simple mode | 3 | 0.883(0.435-1.789) | 0.762 | - | - | - | - | - | - | - |
| CRP | Diabetic polyneuropathy | Weighted mode | 3 | 0.570(0.354-0.917) | 0.147 | - | - | - | - | - | - | - |
| CRLF1 | Diabetic polyneuropathy | Wald ratio | 1 | 6.915(1.971-24.263) | 0.003 | - | - | - | - | - | - | - |
| CRISPLD2 | Diabetic polyneuropathy | Wald ratio | 1 | 0.143(0.025-0.809) | 0.028 | - | - | - | - | - | - | - |
| CREG1 | Diabetic polyneuropathy | Inverse variance weighted | 5 | 0.541(0.389-0.751) | <0.001 | 0 | 0.603 | 0.963 | - | - | - | 0.960 |
| CREG1 | Diabetic polyneuropathy | MR Egger | 5 | 0.459(0.221-0.955) | 0.129 | 0 | 0.362 | 0.948 | 0.053 | 0.108 | 0.657 | - |
| CREG1 | Diabetic polyneuropathy | Weighted median | 5 | 0.521(0.362-0.750) | <0.001 | - | - | - | - | - | - | - |
| CREG1 | Diabetic polyneuropathy | Simple mode | 5 | 0.564(0.341-0.931) | 0.089 | - | - | - | - | - | - | - |
| CREG1 | Diabetic polyneuropathy | Weighted mode | 5 | 0.518(0.348-0.770) | 0.031 | - | - | - | - | - | - | - |
| CPQ | Diabetic polyneuropathy | Wald ratio | 1 | 0.529(0.288-0.973) | 0.040 | - | - | - | - | - | - | - |
| CLSPN | Diabetic polyneuropathy | Wald ratio | 1 | 5.458(1.211-24.589) | 0.027 | - | - | - | - | - | - | - |
| CKM | Diabetic polyneuropathy | Wald ratio | 1 | 0.192(0.080-0.461) | <0.001 | - | - | - | - | - | - | - |
| CDHR2 | Diabetic polyneuropathy | Inverse variance weighted | 5 | 0.559(0.345-0.906) | 0.018 | 0 | 1.413 | 0.842 | - | - | - | 0.523 |
| CDHR2 | Diabetic polyneuropathy | MR Egger | 5 | 0.508(0.259-0.996) | 0.143 | 0 | 1.253 | 0.740 | 0.024 | 0.061 | 0.716 | - |
| CDHR2 | Diabetic polyneuropathy | Weighted median | 5 | 0.536(0.321-0.896) | 0.017 | - | - | - | - | - | - | - |
| CDHR2 | Diabetic polyneuropathy | Simple mode | 5 | 0.527(0.174-1.594) | 0.320 | - | - | - | - | - | - | - |
| CDHR2 | Diabetic polyneuropathy | Weighted mode | 5 | 0.532(0.319-0.888) | 0.073 | - | - | - | - | - | - | - |
| CDH6 | Diabetic polyneuropathy | Inverse variance weighted | 2 | 18.340(1.901-176.938) | 0.012 | 0 | 0.107 | 0.744 | - | - | - | - |
| CD72 | Diabetic polyneuropathy | Wald ratio | 1 | 4.767(1.375-16.530) | 0.014 | - | - | - | - | - | - | - |
| CD6 | Diabetic polyneuropathy | Inverse variance weighted | 7 | 1.274(1.034-1.569) | 0.023 | 0 | 5.374 | 0.497 | - | - | - | 0.591 |
| CD6 | Diabetic polyneuropathy | MR Egger | 7 | 1.305(1.010-1.687) | 0.097 | 5 | 5.256 | 0.385 | -0.014 | 0.042 | 0.751 | - |
| CD6 | Diabetic polyneuropathy | Weighted median | 7 | 1.294(1.050-1.595) | 0.016 | - | - | - | - | - | - | - |
| CD6 | Diabetic polyneuropathy | Simple mode | 7 | 1.803(0.478-6.794) | 0.417 | - | - | - | - | - | - | - |
| CD6 | Diabetic polyneuropathy | Weighted mode | 7 | 1.285(1.045-1.581) | 0.055 | - | - | - | - | - | - | - |
| CD14 | Diabetic polyneuropathy | Inverse variance weighted | 2 | 2.338(1.475-3.707) | <0.001 | 0 | 0.205 | 0.650 | - | - | - | 0.107 |
| CCL26 | Diabetic polyneuropathy | Inverse variance weighted | 3 | 2.981(1.079-8.235) | 0.035 | 3 | 2.071 | 0.355 | - | - | - | 0.423 |
| CCL26 | Diabetic polyneuropathy | MR Egger | 3 | 1.273(0.174-9.310) | 0.851 | 5 | 1.058 | 0.304 | 0.105 | 0.107 | 0.507 | - |
| CCL26 | Diabetic polyneuropathy | Weighted median | 3 | 2.537(0.853-7.547) | 0.094 | - | - | - | - | - | - | - |
| CCL26 | Diabetic polyneuropathy | Simple mode | 3 | 3.572(0.590-21.645) | 0.300 | - | - | - | - | - | - | - |
| CCL26 | Diabetic polyneuropathy | Weighted mode | 3 | 2.081(0.690-6.277) | 0.323 | - | - | - | - | - | - | - |
| CASP3 | Diabetic polyneuropathy | Wald ratio | 1 | 2.003(1.158-3.465) | 0.013 | - | - | - | - | - | - | - |
| CA12 | Diabetic polyneuropathy | Inverse variance weighted | 6 | 0.478(0.301-0.759) | 0.002 | 0 | 0.778 | 0.978 | - | - | - | 0.857 |
| CA12 | Diabetic polyneuropathy | MR Egger | 6 | 0.631(0.263-1.510) | 0.359 | 0 | 0.238 | 0.993 | -0.053 | 0.073 | 0.503 | - |
| CA12 | Diabetic polyneuropathy | Weighted median | 6 | 0.512(0.306-0.859) | 0.011 | - | - | - | - | - | - | - |
| CA12 | Diabetic polyneuropathy | Simple mode | 6 | 0.328(0.138-0.778) | 0.053 | - | - | - | - | - | - | - |
| CA12 | Diabetic polyneuropathy | Weighted mode | 6 | 0.541(0.319-0.917) | 0.071 | - | - | - | - | - | - | - |
| ATOX1 | Diabetic polyneuropathy | Inverse variance weighted | 2 | 0.341(0.117-0.992) | 0.048 | 0 | 0.016 | 0.899 | - | - | - | - |
| ART3 | Diabetic polyneuropathy | Wald ratio | 1 | 0.541(0.322-0.909) | 0.020 | - | - | - | - | - | - | - |
| ARL8B | Diabetic polyneuropathy | Wald ratio | 1 | 0.249(0.083-0.743) | 0.013 | - | - | - | - | - | - | - |
| ARL1 | Diabetic polyneuropathy | Wald ratio | 1 | 0.536(0.320-0.898) | 0.018 | - | - | - | - | - | - | - |
| APOL3 | Diabetic polyneuropathy | Wald ratio | 1 | 6.915(1.971-24.263) | 0.003 | - | - | - | - | - | - | - |
| AMY2B | Diabetic polyneuropathy | Inverse variance weighted | 9 | 0.800(0.644-0.995) | 0.045 | 0 | 4.764 | 0.782 | - | - | - | 0.853 |
| AMY2B | Diabetic polyneuropathy | MR Egger | 9 | 0.864(0.657-1.135) | 0.328 | 0 | 3.952 | 0.785 | -0.033 | 0.037 | 0.397 | - |
| AMY2B | Diabetic polyneuropathy | Weighted median | 9 | 0.820(0.649-1.035) | 0.094 | - | - | - | - | - | - | - |
| AMY2B | Diabetic polyneuropathy | Simple mode | 9 | 0.921(0.462-1.835) | 0.820 | - | - | - | - | - | - | - |
| AMY2B | Diabetic polyneuropathy | Weighted mode | 9 | 0.826(0.652-1.045) | 0.149 | - | - | - | - | - | - | - |
| AMY2A | Diabetic polyneuropathy | Inverse variance weighted | 2 | 0.788(0.639-0.972) | 0.026 | 0 | 0.122 | 0.727 | - | - | - | - |
| AGR3 | Diabetic polyneuropathy | Inverse variance weighted | 5 | 0.326(0.127-0.840) | 0.020 | 0 | 2.585 | 0.630 | - | - | - | 0.782 |
| AGR3 | Diabetic polyneuropathy | MR Egger | 5 | 0.003(0.000-19.895) | 0.287 | 0 | 1.488 | 0.685 | 0.426 | 0.407 | 0.372 | - |
| AGR3 | Diabetic polyneuropathy | Weighted median | 5 | 0.316(0.101-0.996) | 0.049 | - | - | - | - | - | - | - |
| AGR3 | Diabetic polyneuropathy | Simple mode | 5 | 0.277(0.060-1.269) | 0.174 | - | - | - | - | - | - | - |
| AGR3 | Diabetic polyneuropathy | Weighted mode | 5 | 0.291(0.068-1.246) | 0.172 | - | - | - | - | - | - | - |
| ACADM | Diabetic polyneuropathy | Wald ratio | 1 | 0.706(0.507-0.983) | 0.039 | - | - | - | - | - | - | - |

Note: Nsnp, Number of Single Nucleotide Polymorphisms; OR, Odds Ratio; SE, Standard Error of β
