# Supplementary material for: Identification of potential drug targets for diabetic polyneuropathy through Mendelian randomization analysis
Source: Cell Biosci. 2024 Dec 5;14:147. doi: 10.1186/s13578-024-01323-4 (PMC11619124; doi:10.1186/s13578-024-01323-4)
Supplement: Supplementary file 6 — Supplementary Material 6. [file 13578_2024_1323_MOESM6_ESM.docx]

**Steiger Filtering Test Results for cis-pQTLs and Diabetic Polyneuropathy**

| **Exposure** | **Outcome** | **Correct_causal_direction** | **Steiger_pval** |
| --- | --- | --- | --- |
| ACADM | Diabetic polyneuropathy | TRUE | 2.1905E-188 |
| APOF | Diabetic polyneuropathy | TRUE | 5.57895E-81 |
| ART3 | Diabetic polyneuropathy | TRUE | 1.47195E-30 |
| BDNF | Diabetic polyneuropathy | TRUE | 6.86142E-18 |
| CA12 | Diabetic polyneuropathy | TRUE | 1.2281E-163 |
| CASP3 | Diabetic polyneuropathy | TRUE | 5.31553E-11 |
| CCL24 | Diabetic polyneuropathy | TRUE | 4.4393E-163 |
| CCL5 | Diabetic polyneuropathy | TRUE | 8.00898E-88 |
| CD14 | Diabetic polyneuropathy | TRUE | 6.3233E-113 |
| CD163 | Diabetic polyneuropathy | TRUE | 6.75341E-42 |
| CD58 | Diabetic polyneuropathy | TRUE | 1.59878E-95 |
| CD6 | Diabetic polyneuropathy | TRUE | 7.5017E-163 |
| CD72 | Diabetic polyneuropathy | TRUE | 4.77634E-32 |
| CKM | Diabetic polyneuropathy | TRUE | 4.68628E-14 |
| CKM_CKB | Diabetic polyneuropathy | TRUE | 4.68628E-14 |
| CRNN | Diabetic polyneuropathy | TRUE | 1.1909E-164 |
| DDC | Diabetic polyneuropathy | TRUE | 3.4312E-164 |
| DKK3 | Diabetic polyneuropathy | TRUE | 1.5637E-163 |
| EDAR | Diabetic polyneuropathy | TRUE | 1.4478E-162 |
| EFNA3 | Diabetic polyneuropathy | TRUE | 4.32276E-07 |
| ENO3 | Diabetic polyneuropathy | TRUE | 3.3886E-162 |
| ENPP7 | Diabetic polyneuropathy | TRUE | 8.88E-203 |
| FGFBP1 | Diabetic polyneuropathy | TRUE | 4.06082E-31 |
| GGH | Diabetic polyneuropathy | TRUE | 3.6492E-188 |
| HADH | Diabetic polyneuropathy | TRUE | 6.43342E-11 |
| IDI2 | Diabetic polyneuropathy | TRUE | 1.1417E-164 |
| IDUA | Diabetic polyneuropathy | TRUE | 1.1442E-147 |
| IL5RA | Diabetic polyneuropathy | TRUE | 8.039E-164 |
| INHBA_INHBC | Diabetic polyneuropathy | TRUE | 1.76886E-10 |
| INHBC | Diabetic polyneuropathy | TRUE | 1.76886E-10 |
| IPCEF1 | Diabetic polyneuropathy | TRUE | 2.74558E-21 |
| KL | Diabetic polyneuropathy | TRUE | 4.03936E-41 |
| LIFR | Diabetic polyneuropathy | TRUE | 1.58524E-79 |
| LTBR | Diabetic polyneuropathy | TRUE | 1.5672E-164 |
| MGMT | Diabetic polyneuropathy | TRUE | 1.3463E-163 |
| NECTIN4 | Diabetic polyneuropathy | TRUE | 7.3414E-163 |
| NHEJ1 | Diabetic polyneuropathy | TRUE | 1.55808E-36 |
| NUDCD3 | Diabetic polyneuropathy | TRUE | 1.64722E-52 |
| PAEP | Diabetic polyneuropathy | TRUE | 5.5577E-162 |
| PARP1 | Diabetic polyneuropathy | TRUE | 6.13402E-43 |
| PDE3A | Diabetic polyneuropathy | TRUE | 4.66463E-23 |
| PLXNA1 | Diabetic polyneuropathy | TRUE | 4.2369E-141 |
| PODXL | Diabetic polyneuropathy | TRUE | 1.781E-164 |
| PTPRM | Diabetic polyneuropathy | TRUE | 2.93352E-16 |
| PVR | Diabetic polyneuropathy | TRUE | 2.8918E-161 |
| PVRL4 | Diabetic polyneuropathy | TRUE | 2.99362E-09 |
| RAB31 | Diabetic polyneuropathy | TRUE | 4.7058E-124 |
| RET | Diabetic polyneuropathy | TRUE | 1.7967E-163 |
| ROR1 | Diabetic polyneuropathy | TRUE | 5.8793E-163 |
| SERPINE1 | Diabetic polyneuropathy | TRUE | 1.35894E-28 |
| SERPINE2 | Diabetic polyneuropathy | TRUE | 3.6752E-188 |
| SLITRK6 | Diabetic polyneuropathy | TRUE | 8.9498E-162 |
| SPINT2 | Diabetic polyneuropathy | TRUE | 1.7345E-203 |
| SRA1 | Diabetic polyneuropathy | TRUE | 6.01555E-41 |
| SRI | Diabetic polyneuropathy | TRUE | 7.82774E-32 |
| TGFA | Diabetic polyneuropathy | TRUE | 2.94489E-42 |
| TIGIT | Diabetic polyneuropathy | TRUE | 8.4023E-14 |
| TNFAIP3 | Diabetic polyneuropathy | TRUE | 8.98009E-70 |
| TNFSF14 | Diabetic polyneuropathy | TRUE | 2.8789E-116 |
| UPP1 | Diabetic polyneuropathy | TRUE | 3.24061E-26 |
| VAV1 | Diabetic polyneuropathy | TRUE | 4.78864E-12 |
| VNN2 | Diabetic polyneuropathy | TRUE | 3.0166E-187 |
