# Supplementary material for: Identification of potential drug targets for diabetic polyneuropathy through Mendelian randomization analysis
Source: Cell Biosci. 2024 Dec 5;14:147. doi: 10.1186/s13578-024-01323-4 (PMC11619124; doi:10.1186/s13578-024-01323-4)
Supplement: Supplementary file 7 — Supplementary Material 7. [file 13578_2024_1323_MOESM7_ESM.docx]

**Steiger Filtering Test Results for cis-pQTLs + trans-pQTLs and Diabetic Polyneuropathy**

| exposure | outcome | correct_causal_direction | steiger_pval |
| --- | --- | --- | --- |
| ACADM | Diabetic polyneuropathy | TRUE | 2.1905E-188 |
| AGR3 | Diabetic polyneuropathy | TRUE | 2.3846E-74 |
| AMY2A | Diabetic polyneuropathy | TRUE | 0 |
| AMY2B | Diabetic polyneuropathy | TRUE | 0 |
| APOL3 | Diabetic polyneuropathy | TRUE | 4.6499E-200 |
| ARL1 | Diabetic polyneuropathy | TRUE | 3.00822E-28 |
| ARL8B | Diabetic polyneuropathy | TRUE | 4.30414E-39 |
| ART3 | Diabetic polyneuropathy | TRUE | 1.47195E-30 |
| ATOX1 | Diabetic polyneuropathy | TRUE | 4.82266E-21 |
| CA12 | Diabetic polyneuropathy | TRUE | 6.65E-252 |
| CASP3 | Diabetic polyneuropathy | TRUE | 5.31553E-11 |
| CCL26 | Diabetic polyneuropathy | TRUE | 2.98E-221 |
| CD14 | Diabetic polyneuropathy | TRUE | 8.9915E-151 |
| CD6 | Diabetic polyneuropathy | TRUE | 3.27E-291 |
| CD72 | Diabetic polyneuropathy | TRUE | 4.77634E-32 |
| CDH6 | Diabetic polyneuropathy | TRUE | 4.1856E-199 |
| CDHR2 | Diabetic polyneuropathy | TRUE | 0 |
| CKM | Diabetic polyneuropathy | TRUE | 4.68628E-14 |
| CLSPN | Diabetic polyneuropathy | TRUE | 2.9181E-29 |
| CPQ | Diabetic polyneuropathy | TRUE | 2.6202E-164 |
| CREG1 | Diabetic polyneuropathy | TRUE | 0 |
| CRISPLD2 | Diabetic polyneuropathy | TRUE | 5.92164E-37 |
| CRLF1 | Diabetic polyneuropathy | TRUE | 8.7866E-115 |
| CRP | Diabetic polyneuropathy | TRUE | 2.4738E-157 |
| CRYZL1 | Diabetic polyneuropathy | TRUE | 6.66684E-64 |
| CTF1 | Diabetic polyneuropathy | TRUE | 3.00822E-28 |
| CTSH | Diabetic polyneuropathy | TRUE | 1.18652E-63 |
| DCK | Diabetic polyneuropathy | TRUE | 3.00822E-28 |
| DCUN1D1 | Diabetic polyneuropathy | TRUE | 1.5209E-152 |
| DCUN1D5 | Diabetic polyneuropathy | TRUE | 6.26176E-93 |
| DDC | Diabetic polyneuropathy | TRUE | 6.15E-215 |
| DEFB104A | Diabetic polyneuropathy | TRUE | 5.35021E-09 |
| DLK1 | Diabetic polyneuropathy | TRUE | 3.0812E-200 |
| EDAR | Diabetic polyneuropathy | TRUE | 0 |
| EDIL3 | Diabetic polyneuropathy | TRUE | 8.54077E-36 |
| EFNA3 | Diabetic polyneuropathy | TRUE | 4.32276E-07 |
| EMC8 | Diabetic polyneuropathy | TRUE | 1.66297E-13 |
| ENG | Diabetic polyneuropathy | TRUE | 0 |
| ENPP7 | Diabetic polyneuropathy | TRUE | 8.88E-203 |
| FGFBP1 | Diabetic polyneuropathy | TRUE | 1.1436E-154 |
| FJX1 | Diabetic polyneuropathy | TRUE | 1.1785E-127 |
| GALNT13 | Diabetic polyneuropathy | TRUE | 3.25332E-31 |
| GGT2 | Diabetic polyneuropathy | TRUE | 1.8793E-109 |
| GHR | Diabetic polyneuropathy | TRUE | 6.18E-241 |
| GOLM2 | Diabetic polyneuropathy | TRUE | 0 |
| GPA33 | Diabetic polyneuropathy | TRUE | 2.05E-285 |
| GPC1 | Diabetic polyneuropathy | TRUE | 0 |
| GPN1 | Diabetic polyneuropathy | TRUE | 7.80E-222 |
| HADH | Diabetic polyneuropathy | TRUE | 6.43342E-11 |
| HAVCR2 | Diabetic polyneuropathy | TRUE | 2.5008E-165 |
| HNF4A | Diabetic polyneuropathy | TRUE | 4.53795E-16 |
| IDI2 | Diabetic polyneuropathy | TRUE | 1.1417E-164 |
| IDUA | Diabetic polyneuropathy | TRUE | 9.1348E-172 |
| INHBA_INHBC | Diabetic polyneuropathy | TRUE | 6.59E-292 |
| INHBB | Diabetic polyneuropathy | TRUE | 0 |
| INHBC | Diabetic polyneuropathy | TRUE | 4.5611E-139 |
| IPCEF1 | Diabetic polyneuropathy | TRUE | 9.88758E-37 |
| ITM2B | Diabetic polyneuropathy | TRUE | 2.5001E-194 |
| JPH4 | Diabetic polyneuropathy | TRUE | 3.83025E-20 |
| LEFTY2 | Diabetic polyneuropathy | TRUE | 8.62E-291 |
| LRRC25 | Diabetic polyneuropathy | TRUE | 0 |
| LRRN1 | Diabetic polyneuropathy | TRUE | 5.68784E-49 |
| MGMT | Diabetic polyneuropathy | TRUE | 7.58202E-54 |
| NECTIN4 | Diabetic polyneuropathy | TRUE | 1.32E-217 |
| NEFL | Diabetic polyneuropathy | TRUE | 3.00822E-28 |
| NHEJ1 | Diabetic polyneuropathy | TRUE | 5.17611E-84 |
| NPC2 | Diabetic polyneuropathy | TRUE | 9.3647E-162 |
| NUDCD3 | Diabetic polyneuropathy | TRUE | 8.2063E-117 |
| OBP2B | Diabetic polyneuropathy | TRUE | 1.26E-215 |
| PAEP | Diabetic polyneuropathy | TRUE | 1.9757E-189 |
| PCSK9 | Diabetic polyneuropathy | TRUE | 1.8862E-133 |
| PDE3A | Diabetic polyneuropathy | TRUE | 4.64082E-72 |
| PDZD11 | Diabetic polyneuropathy | TRUE | 8.16116E-10 |
| PDZK1IP1 | Diabetic polyneuropathy | TRUE | 3.00822E-28 |
| PKP2 | Diabetic polyneuropathy | TRUE | 4.33085E-51 |
| PLXNA4 | Diabetic polyneuropathy | TRUE | 2.16516E-75 |
| PPA2 | Diabetic polyneuropathy | TRUE | 6.0096E-39 |
| PPP1R12A | Diabetic polyneuropathy | TRUE | 2.96894E-41 |
| PPP2R3A | Diabetic polyneuropathy | TRUE | 5.35021E-09 |
| PRKCG | Diabetic polyneuropathy | TRUE | 2.98743E-41 |
| PSAP | Diabetic polyneuropathy | TRUE | 1.7399E-175 |
| PVR | Diabetic polyneuropathy | TRUE | 2.8918E-161 |
| PVRL4 | Diabetic polyneuropathy | TRUE | 2.99362E-09 |
| RAB22A | Diabetic polyneuropathy | TRUE | 2.54322E-96 |
| RAB31 | Diabetic polyneuropathy | TRUE | 3.99928E-36 |
| RACGAP1 | Diabetic polyneuropathy | TRUE | 1.39242E-06 |
| RARRES3 | Diabetic polyneuropathy | TRUE | 1.3755E-174 |
| RET | Diabetic polyneuropathy | TRUE | 4.11E-223 |
| RGS7 | Diabetic polyneuropathy | TRUE | 3.00822E-28 |
| RNASET2 | Diabetic polyneuropathy | TRUE | 0 |
| RSPO3 | Diabetic polyneuropathy | TRUE | 0 |
| SARS | Diabetic polyneuropathy | TRUE | 3.00822E-28 |
| SDC1 | Diabetic polyneuropathy | TRUE | 3.5561E-176 |
| SFXN5 | Diabetic polyneuropathy | TRUE | 2.5221E-165 |
| SHBG | Diabetic polyneuropathy | TRUE | 8.81E-113 |
| SMOC2 | Diabetic polyneuropathy | TRUE | 1.9869E-201 |
| SPINT2 | Diabetic polyneuropathy | TRUE | 1.3906E-126 |
| SRA1 | Diabetic polyneuropathy | TRUE | 5.62062E-93 |
| SRI | Diabetic polyneuropathy | TRUE | 7.82774E-32 |
| SUMF1 | Diabetic polyneuropathy | TRUE | 1.9722E-186 |
| TBCA | Diabetic polyneuropathy | TRUE | 3.00822E-28 |
| TBXAS1 | Diabetic polyneuropathy | TRUE | 3.39931E-09 |
| TG | Diabetic polyneuropathy | TRUE | 1.87387E-10 |
| TGFA | Diabetic polyneuropathy | TRUE | 1.36E-265 |
| TMCC3 | Diabetic polyneuropathy | TRUE | 9.8147E-173 |
| TNFAIP3 | Diabetic polyneuropathy | TRUE | 8.98009E-70 |
| TNFRSF19 | Diabetic polyneuropathy | TRUE | 4.81E-212 |
| TNFSF14 | Diabetic polyneuropathy | TRUE | 5.63339E-27 |
| TP53I11 | Diabetic polyneuropathy | TRUE | 9.8147E-173 |
| TRAPPC3 | Diabetic polyneuropathy | TRUE | 4.53795E-16 |
| UBE2G1 | Diabetic polyneuropathy | TRUE | 4.10512E-35 |
| ULBP2 | Diabetic polyneuropathy | TRUE | 6.25E-241 |
| UPP1 | Diabetic polyneuropathy | TRUE | 3.24061E-26 |
| VNN2 | Diabetic polyneuropathy | TRUE | 1.95E-224 |
| YWHAQ | Diabetic polyneuropathy | TRUE | 3.6024E-155 |
| ZHX3 | Diabetic polyneuropathy | TRUE | 1.28936E-65 |
